# Integrative Analysis Unveils the Correlation of Aminoacyl-tRNA Biosynthesis Metabolites with the Methylation of the *SEPSECS* Gene in Huntington’s Disease Brain Tissue

**DOI:** 10.3390/genes14091752

**Published:** 2023-09-02

**Authors:** Sangeetha Vishweswaraiah, Ali Yilmaz, Nazia Saiyed, Abdullah Khalid, Purvesh R. Koladiya, Xiaobei Pan, Shirin Macias, Andrew C. Robinson, David Mann, Brian D. Green, Ieva Kerševičiūte, Juozas Gordevičius, Uppala Radhakrishna, Stewart F. Graham

**Affiliations:** 1 Department of Obstetrics and Gynecology, Corewell Health William Beaumont University Hospital, 3601 W. 13 Mile Road, Royal Oak, MI 48073, USA; sangeetha.vishweswaraiah@corewellhealth.org (S.V.); uppalar99@gmail.com (U.R.); 2 Metabolomics Department, Corewell Health Research Institute, 3811 W. 13 Mile Road, Royal Oak, MI 48073, USA; ali.yilmaz@corewellhealth.org (A.Y.); nazia.saiyed@corewellhealth.org (N.S.); abdullah.khalid@corewellhealth.org (A.K.); purveshrohitkumar.koladiya@corewellhealth.org (P.R.K.); 3 Advanced Asset Technology Centre, Institute for Global Food Security, Queen’s University Belfast, Belfast BT9 5DL, UK; x.pan@qub.ac.uk (X.P.); smacias01@qub.ac.uk (S.M.); b.green@qub.ac.uk (B.D.G.); 4 Faculty of Biology, Medicine and Health, School of Biological Sciences, Division of Neuroscience, The University of Manchester, Salford Royal Hospital, Salford M6 8HD, UK; andrew.c.robinson@manchester.ac.uk (A.C.R.); davidmann1948@hotmail.com (D.M.); 5 VUGENE, LLC, 625 Kenmoor Ave Suite 301 PMB 96578, Grand Rapids, MI 49546, USA; ieva@vugene.us (I.K.); juozas@vugene.us (J.G.); 6 Department of Obstetrics and Gynecology, Oakland University-William Beaumont School of Medicine, Rochester, MI 48309, USA

**Keywords:** metabolomics, epigenetics, integrative omics, epi-metabolomics, Huntington’s disease, brain

## Abstract

The impact of environmental factors on epigenetic changes is well established, and cellular function is determined not only by the genome but also by interacting partners such as metabolites. Given the significant impact of metabolism on disease progression, exploring the interaction between the metabolome and epigenome may offer new insights into Huntington’s disease (HD) diagnosis and treatment. Using fourteen post-mortem HD cases and fourteen control subjects, we performed metabolomic profiling of human postmortem brain tissue (striatum and frontal lobe), and we performed DNA methylome profiling using the same frontal lobe tissue. Along with finding several perturbed metabolites and differentially methylated loci, Aminoacyl-tRNA biosynthesis (adj *p*-value = 0.0098) was the most significantly perturbed metabolic pathway with which two CpGs of the *SEPSECS* gene were correlated. This study improves our understanding of molecular biomarker connections and, importantly, increases our knowledge of metabolic alterations driving HD progression.

## 1. Introduction

Huntington’s disease (HD) is a neurodegenerative disorder that results in a decline of neurological function, characterized by worsening motor skills, cognitive abilities, and psychological symptoms [1]. The root cause of HD is the expansion of a CAG trinucleotide repeat in the huntingtin (*HTT*) gene, producing a toxic protein that adversely affects the brain’s cells [2]. The average age for symptom onset in HD is between 30 and 50 years, although, in certain instances, symptoms may begin before the age of 20. The early indications of HD include irritability, depression, small uncontrolled movements, impaired coordination, and difficulty with learning or decision-making. In advanced stages, many individuals with HD experience involuntary twitching movements that become more severe over time. Walking, speaking, and swallowing may also become difficult [3,4]. The global prevalence of HD is estimated to be around 5–10 cases per 100,000 individuals, and it is inherited in an autosomal dominant pattern [5]. The onset and severity of HD symptoms can vary significantly among individuals, even within families with identical genetic mutations. Presently, no cure exists for HD, and available treatments only provide relief for symptoms associated with the disease [6].

HD involves neurodegeneration, mainly in the basal ganglia, particularly the striatum, impacting movement and reward-related neural activity [7]. The neocortex, which connects to the striatum, shrinks during HD, affecting other brain areas later [8]. Disease development involves complex mechanisms like transcription dysfunction and abnormal trafficking [9]. Mood and behavior changes related to frontal lobe dysfunction serve as early HD indicators [10,11,12,13].

In the pursuit of comprehending complex biological systems, multi-omics studies hold significant importance. Such studies involve the integration and analysis of data from various “-omics” fields, such as genomics, transcriptomics, proteomics, and metabolomics, to obtain a holistic view of biological processes. By exploring the interactions between different molecular levels, multi-omics studies can reveal novel pathways and mechanisms that contribute to complex diseases such as neurodegenerative diseases [14,15]. Multi-omics studies can aid in identifying new drug targets and developing more effective therapeutic strategies [16].

DNA methylation is an epigenetic mechanism that plays a crucial role in regulating gene expression, and numerous studies have revealed the involvement of altered DNA methylation in the pathogenesis of neurodegenerative disorders, including HD [17,18]. In recent years, DNA methylation has emerged as a promising biomarker for HD diagnosis and prognosis [18]. For instance, a study has indicated that differences in DNA methylation levels of the *HTT* gene may contribute to the tissue-specific variation of its expression. Additionally, the mutant *HTT* gene appears to impact the epigenetic age of individuals with HD [18]. Another study reported that DNA methylation levels in certain genes correlated with the advancement and severity of the disease [19]. Furthermore, some studies have explored the therapeutic potential of targeting DNA methylation in HD [18,20,21].

Metabolomics possesses the capability to reveal the underlying mechanisms of disease progression that have the potential to function as prognostic biomarkers for risk assessment [22]. The discovery of metabolic biomarkers and their corresponding pathways linked to the progression of HD can enhance comprehension of the disease’s pathophysiological mechanisms. Moreover, this can stimulate the development of efficacious treatments by providing precise assessments of the disease advancement [23,24]. Several metabolomics investigations involving human subjects and other models have produced intriguing outcomes, indicating significant alterations in metabolic pathways associated with HD [23,25]. Several -omics studies, including some in the field of metabolomics, have showcased metabolic modifications, encompassing alterations in protein, carbohydrate, cholesterol, lipid, and amino acid metabolism [26,27,28,29]. 

Given the potential impact of both DNA methylation and metabolomics on the pathogenesis of HD, and the potential for interplay between these two -omics technologies, we proposed that investigating them in tandem could reveal potential signaling mechanisms for therapeutic targeting. To this end, we conducted DNA methylation profiling of the frontal lobe, as well as metabolite profiling in both the striatum and frontal lobe of postmortem individuals with HD. The implications of dysregulated pathways are presented and discussed.

## 2. Methods

### 2.1. Study Samples

This research was carried out by utilizing brain tissue samples collected posthumously from HD-affected patients and from normal controls. The Manchester Brain Bank provided the brain tissue [30]. The use of post-mortem human brain samples was approved by the Manchester Brain Bank Management Committee working under conditions agreed upon with the Newcastle and North Tyneside 1 Research Ethics Committee (REC Reference# 09/H0906/52 + 5). Further ethical approval was granted by the Beaumont Institutional Review Board (IRB# 2014–353). Fourteen post-mortem HD cases and fourteen controls were used for metabolomics profiling, and the same fourteen cases, and thirteen controls, were considered for the methylation analysis. Control subjects had no evidence of Huntington’s pathology and there was no recent history of dementia-associated medication use among them. Both striatum and frontal lobe tissues were metabolically profiled, whereas only frontal lobe tissue was available for epigenetic analysis. The HD cases all exhibited a moderate to severely atrophied corpus striatum consistent with grades 2 or 3. The exact CAG repeat numbers were not available, but the diagnosis of HD was confirmed using genetic testing except for in two cases, where the diagnosis was based on the presence of ubiquitinated/p62 positive intra-nuclear inclusions within cortical and striatal neurons, as observed in all other HD cases but not in control cases. The demographic characteristics of the study group are provided in Table 1. 

### 2.2. ^1^H NMR Analysis

The samples were first stored at −80 °C before being prepared. To minimize the amount of heat produced, the samples were subsequently lyophilized and milled into a fine powder under liquid nitrogen. To analyze the samples using ^1^H NMR, previously optimized methods were employed [31]. The 50 mg samples of the lyophilized and milled tissue were extracted in 50% methanol/water (1 mg per 10 µL; 0.1 g/mL) in a sterile 2 mL Eppendorf tube. The samples were mixed and sonicated for 20 min at a frequency of 50–60 Hz to achieve homogenization, cell disruption, and compound extraction, and centrifuged at 13,000× *g* at 4 °C for 30 min to remove any macro molecules that may affect the NMR signal. After collecting the supernatants, they were dried under vacuum using a Savant DNA Speedvac (Thermo Scientific, USA) and reconstituted in 285 µL of 50 mM potassium phosphate buffer (pH 7.0), 30 µL of Sodium 2,2-dimethyl-2-silapentane-5-sulfonate (DSS), and 35 µL of D_2_O. A volume of 200 µL of the reconstituted sample was then transferred to a 3 mm Bruker NMR tube for analysis. All samples were stored at 4 °C in a thermostatically controlled SampleJet autosampler (Bruker-Biospin, Billerica, MA, USA) and heated to room temperature over 3 min prior to NMR analysis.

To record all 1D ^1^H NMR data, a randomized running order was utilized on a Bruker ASCEND HD 600 MHz spectrometer (Bruker-Biospin, Billerica, MA, USA) coupled with a 5 mm TCI cryoprobe at a temperature of 300 (±0.5) K. For each sample, 256 transients were collected as 64k data points with a spectral width of 12 kHz (20 ppm) using a pulse sequence called CPP WaterSupp (Bruker pulse program: pusenoesypr1d), which was developed by Mercier et al. (2011) [32], with an inter-pulse delay of 9.65 s. The data collection protocol involved a 180 s temperature equilibration period, fast 3D shimming utilizing the z-axis profile of the ^2^H NMR solvent signal, receiver gain adjustment, and acquisition. The free induction decay signal was zero-filled to 128 k points and exponentially multiplied with a 0.1 Hz line broadening factor. After Fourier transformation, the zero and first-order phase constants were manually optimized, and a polynomial baseline correction of the FID (degree 5) was applied for precise quantitation. Chenomx NMR Suite (ver. 8.1, Chenomx, Edmonton, AB, Canada) was used for processing and analyzing all spectra.

### 2.3. Direct Injection/Liquid Chromatography–Mass Spectral Analysis (DI/LC-MS/MS)

Post-mortem brain samples were prepared and analyzed using a method previously reported by Urban et al. and our group [33,34]. Post-mortem brain samples were subjected to freeze drying using a Christ α 1-4LD Plus freeze dryer (IMA Life) and milled to a fine powder under liquid nitrogen using a 6870-freezer mill (SpexSamplePrep, Metuchen, NJ, USA). Subsequently, 10 mg of tissue was mixed with 300 µL of 85% ethanol and 15% phosphate-buffered saline. Samples were sonicated for 5 min in ice water, mixed for 30 s, and then centrifuged at 10,000× *g* for 5 min at 4 °C. 

A total of 10 µL of the subsequent supernatant was analyzed using the targeted, commercially available quantitative DI/LC-MS/MS AbsoluteIDQ p180 kit (Biocrates, Innsbruck, Austria), as previously described [35], and all sample preparatory steps were completed as detailed by the manufacturer. Metabolites were separated and quantified using a reverse phase column (Waters ACQUITY UPLC BEH C18 2.1 × 50 mm, 1.7 µm; Wexford, Ireland) on a UPLC system (I-Class, Waters Corporation, Budapest, Hungary) coupled to a triple quadrupole mass spectrometer (Xevo TQ-S, Waters Corporation) operating in the multiple reaction monitoring (MRM) mode. All remaining compounds (acylcarnitines, hexoses, glycerophospholipids, and sphingolipids) were quantified using the same mass spectrometer using flow injection analysis, operating in multiple reaction monitoring mode. Metabolite concentrations were calculated and expressed in micromolar (µM). 

### 2.4. Genome-Wide DNA Methylation Assay

The QIAamp DNA Mini Kit (Qiagen) was used to extract genomic DNA from the lyophilized and milled brain tissue for the genome-wide DNA methylation assay. The Illumina Infinium Epic HumanMethylation850K arrays were employed to profile DNA methylation (Illumina, Inc., San Diego, CA, USA), which covers >850,000 methylation sites per sample, including translation start sites, enhancers, gene bodies, promoters, intronic regions, and also the CpG islands all over the genome at a single-nucleotide resolution. A total of 500 ng of genomic DNA was utilized from each sample that was subjected to bisulfite conversion using the EZ DNA Methylation-Direct Kit (Zymo Research, Orange, CA, USA). The BeadChips were handled in accordance with the manufacturer’s guidelines, and the Illumina iScan (Illumina, USA) was used to image the fluorescently stained BeadChips.

### 2.5. Data Analysis

Metabolomic profiling: The raw metabolomic data underwent sum normalization, autoscaling, and log transformation. Principal Component Analysis (PCA) was employed to detect outliers; any sample deviating by more than 3 standard deviations away from the center of the first three principal components was considered an outlier (i.e., 99.7% confidence). No outliers were identified. To estimate sample diagnosis, a logistic regression model was applied, using the first five principal components, as well as sample age, sex, and post-mortem interval as inputs. To identify differentially expressed metabolites, a robust linear regression model was fitted to each metabolite, with sample diagnosis, age, sex, and PMI as covariates. *p*-values for diagnosis were calculated using empirical Bayes treatment of fitted models. Metabolites with FDR q < 0.05 were deemed significantly differentially expressed in the disease. To estimate metabolite pathway enrichment, the metabolites were ranked based on their log-transformed *p* value multiplied by the sign of fold change. This ranking method ensured that metabolites with a significant increase in abundance were at the top of the list, while those with the most significant decrease in abundance were at the bottom. Any metabolites without an HMDB ID were removed from the ranked list. Metabolomic pathways were downloaded from the KEGG database using the R package multiGSEA, and then metabolite set enrichment was calculated with 10,000 permutations using the fgsea package (version 1.16.0).

DNA methylation profiling: The raw *idat files were read using *minfi* package of bioconductor, which was also considered to mark the failed methylation probes. To identify outliers in the data, several quality control measures were performed. First, samples with more than 20% of failed probes were considered outliers based on the proportion of failed probes. The median probe intensity of U and M probes was then calculated, and samples in the lower-left corner of the plot were marked as outliers. Gender prediction was performed by comparing the median signal in X and Y chromosomes, and samples with a mismatch between the predicted and known gender were marked as outliers. Principal component analysis of the centered, unnormalized β value matrix was used to identify samples deviating by more than 2 standard deviations from the mean of the first three principal components and marked as outliers. Noob normalization was used to normalize the samples, and methylation β values were extracted for the remaining probes. To adjust for the effect of sample position on the EPIC array, the empiricalBayesLM function from the WGCNA package was used, and the effect was modeled as a second-degree polynomial. The proportion of neuronal cells in each sample was estimated using the flow-sorted PFC samples and the estimateCellCount function from the minfi package. The sample DNA methylation age was estimated using the ENmix package. Age acceleration was defined as the residuals of a linear model, where DNAmAge was the dependent variable, and sample chronological age, sex, postmortem interval, and proportion of neuronal cells were the independent variables. To identify differentially methylated CpGs, robust linear regression was employed using the R package limma with sample diagnosis, age, sex, postmortem interval, and proportion of neuronal nuclei as covariates. *p*-value estimates for diagnosis were obtained after the empirical Bayes treatment of the fitted models. Cytosines with a false discovery rate (FDR) q-value of less than 0.05 were considered significantly altered in HD individuals.

To conduct an epigenome enrichment analysis, cytosines were mapped to gene names using the UCSC_RefGene_Name column specified in the Bioconductor EPIC array annotation package IlluminaHumanMethylationEPICanno.ilm10b4.hg19. When a CpG locus was annotated with multiple genes multiple times, the one that was most frequently associated with the locus was chosen. The genes were ranked based on the significance of the affected cytosines multiplied by the sign of fold change. For genes that mapped to multiple cytosines, the one with the smallest *p*-value was selected. To conduct the pathway enrichment analysis, KEGG pathways were downloaded and the fgsea function was used. 

### 2.6. Epigenome–Metabolome Interactions

To establish epigenome–metabolome interactions within each pathway, metabolites and transcripts pertaining to the pathway were selected. Pathway–protein associations were obtained from SMPDB 63 (https://smpdb.ca). Only CpGs that were associated with any of the transcripts in the pathway were further considered. The concordance of each metabolite–CpG pair was established by fitting a robust linear regression model without an intercept, where the standardized methylation value was the response variable, and standardized metabolite abundance, as well as diagnosis, age, and sex were the independent variables. Concordance *p* values were adjusted using FDR, and those with FDR q < 0.2 were reported. 

### 2.7. Diagnostic Models

To evaluate the diagnostic models based on metabolome, the normalized data were used, and no other additional data preprocessing was performed. Two basic diagnostic models were trained: *glmnet* (LASSO), and Random Forest (rf). In addition, an ensemble of those models was built using either linear (ensemble linear) or glmnet-weighted (ensemble glmnet) combination of basic predictors. The area under the curve (AUC) metric was used as the training objective. The data were randomly split into ten training and validation sets. Within each training set, bootstrapping was performed to train and select the best models. The models were then evaluated on the validation set.

For the methylome, we transformed the normalized β values into M values and applied batch correction to remove Sentrix array ID and Position effects from the data. We randomly split the data into a training (N = 20) and validation (N = 4) sets. For each such split, we used the training data to filter the loci by applying a t-test and selecting top 50.000 loci for further use in training the models. We used glmnet, random forest, and SVM with linear and radial kernel algorithms to build and evaluate the models. In addition, we combined these models into ensembles using either linear weighting of predictors (ensemble linear) or glmnet inferred weights of predictors (ensemble glmnet). Finally, we trained a classifier using each of the above algorithms on the full dataset. 

## 3. Results

### 3.1. HD Brain Metabolomic Profile

To study any underlying metabolic alterations in the brain of people who died from HD, we profiled the striatum and the frontal lobe of these individuals and compared them with controls. In both the striatum and frontal lobes, the variance inflation analysis showed that the control subjects were more likely to be younger, which correlates with the chronological age. However, age was controlled for in all linear models. Variance inflation for the striatum and frontal lobe are shown in Supplementary Figures S1 and S2, respectively. Normalized intensities using the striatum and frontal lobe data are provided in Supplementary Figures S3 and S4, respectively.

Targeted metabolomic profiling revealed that, of the 166 metabolites, 4 were significantly upregulated in the striatum of HD brains when compared to controls (FDR adjusted q-value < 0.05). This included taurine, SM.C16.0, PC.aa.C40.3, and SM.C18.1 (Supplementary Table S1). In the frontal lobe, we identified 14 metabolites that were significantly different between HD and control brains (FDR adjusted q-value < 0.05). Eight were upregulated and the remaining six were downregulated in HD brains. The top four were PC.aa.C38.4, lysoPC.a.C18.2, C18.1.OH, and C2 (Supplementary Table S2).

**Figure 1:**
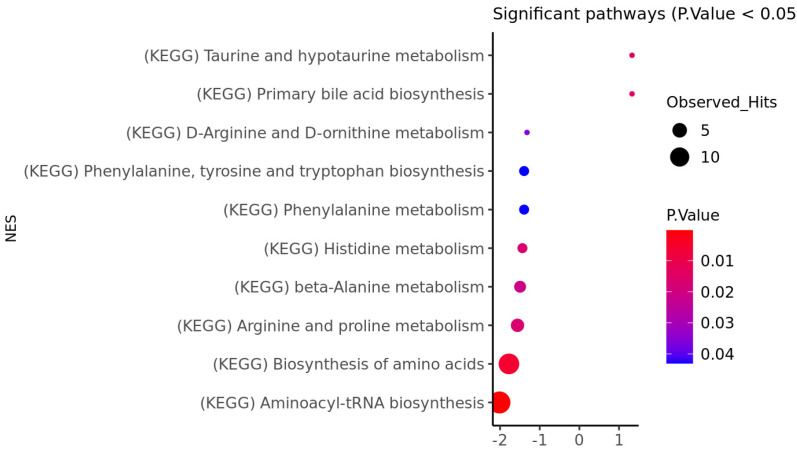
The metabolic pathways that are significantly enriched with metabolites have been identified using the striatum region of HD patients. The *x*-axis represents the hyper-methylated (+) and hypo-methylated (−) status of pathways, whereas the *y*-axis provides the pathways that were perturbed. The node size indicates the number of observed hits that are associated with significant pathways. The node color indicates the significance level based on the *p*-value (<0.05).

**Figure 2:**
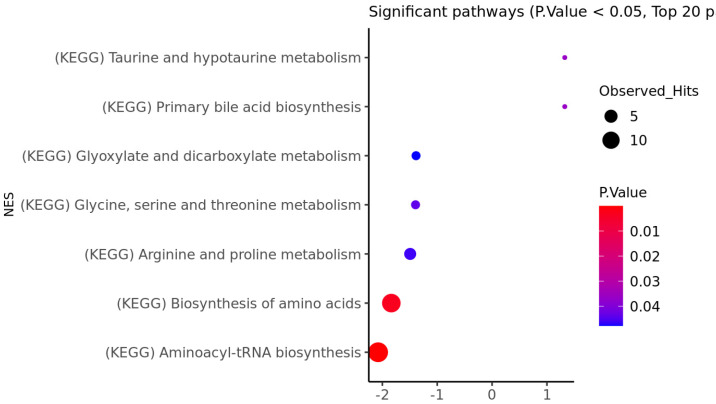
The metabolic pathways that are significantly enriched with metabolites have been identified using the frontal lobe region of HD patients.

We also performed metabolomic set enrichment analysis for both the striatum (Figure 1) and the frontal lobe (Figure 2). Interestingly, both analyses highlighted the same two pathways as being significantly perturbed. These include aminoacyl-tRNA biosynthesis and the biosynthesis of amino acids (FDR adjusted q-value less than 0.05). 

### 3.2. HD Brain Methylation Profile

The Sentrix ID of the sample wells was found to be inflated and adjusted (Supplementary Figure S5). Further, the enrichment of genomic regions by methylation markers using the frontal lobe of HD patients vs. the control group revealed enrichment of hypomethylated markers in the intron and 3′ UTRs. CGI shores are enriched with hyper-methylated loci, and the open sea areas are enriched with hypo-methylated loci. In terms of genomic features, hypo-methylated loci tend to overlap introns and 3′UTR regions of genes. The enrichment of genomic regions is depicted in Supplementary Figure S6, and the details are provided in Supplementary Table S3. There were 11,955 significantly differentially methylated CpGs identified (FDR adjusted q-value = 0.05) when the frontal lobes of HD patients were compared with those of control subjects. Among them, 11,292 CpGs were hypomethylated, and the remaining 663 were significantly hypermethylated (Supplementary Table S4).

### 3.3. Epigenome and Metabolome Correlation

This study found correlations between DNA methylation and the metabolome, specifically identifying several genes (Trafficking Protein Particle Complex Subunit 10 [*TRAPPC10*], CUB And Sushi Multiple Domains 3 [*CSMD3*], 5-Methyltetrahydrofolate-Homocysteine Methyltransferase Reductase [*MTRR*], Pecanex 1 [*PCNX*], PC-Esterase Domain Containing 1B [*PCED1B*], Glucose-6-Phosphatase Catalytic Subunit 2 [*G6PC2*], Protocadherin 7 [*PCDH7*], Glypican 6 [*GPC6*], Ribosomal Protein S25 [*RPS25*], and Casein Kinase 1 γ 3 [*CSNK1G3*]) that are correlated with the metabolites, phenylalanine, and methionine. Further details are available in Supplementary Table S5.

**Figure 3:**
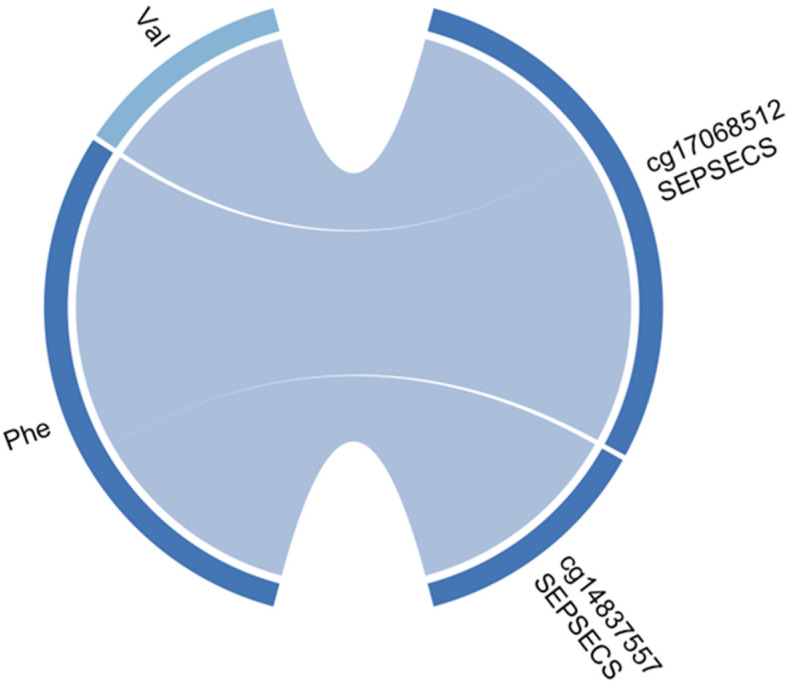
The “circos plot” displays the negative correlation between significantly differentially methylated cytosines and metabolites related to the aminoacyl-tRNA biosynthesis pathway. Metabolites Val and Phe have been downregulated in metabolome analysis, as indicated by the blue color on the edge of the circos plot. The cytosines pertaining to *SEPSECS* gene have been hypo-methylated as well. The blue strips connecting metabolites and cytosines indicate a negative correlation between methylation and metabolite abundance when adjusted for age, sex, postmortem delay, and condition.

The linear model interaction network between CpGs and metabolites was established. The most significantly affected pathway in this study was the Aminoacyl-tRNA biosynthesis pathway. Within this pathway, two metabolites, valine and phenylalanine, were found to interact significantly with two CpGs of the same gene, *SEPSECS*. Specifically, HMDB0000159 (phenylalanine) interacted with cg17068512 (*p*-value = 0.0005), HMDB0000159 interacted with cg14837557 (*p*-value = 0.004), and HMDB0000883 (valine) interacted with cg17068512 (*p*-value = 0.007). These CpGs were located on different transcripts of the *SEPSECS* gene body. Interestingly, both metabolites were found to be negatively correlated with the methylation of sites belonging to the *SEPSECS* gene (see Figure 3 and Supplementary Table S6).

### 3.4. Diagnostic Models

Using metabolomics data acquired from striatal tissue (Supplementary Figure S7), we were able to develop a predictive model with an AUC of 0.82 on glmnet. This was followed by ensemble Linear AUC with 0.79, rf AUC with 0.75, and ensembleGlmnet AUC with 0.74. Using frontal lobe metabolomics data (Supplementary Figure S8), we developed a predictive model with an AUC of 0.84 on glmnet, while both ensembleLinear and ensembleGlmnet had an AUC of 0.8. The rf analysis achieved an AUC of 0.74.

Based on the methylome data, we evaluated the performance of several models. Our results indicate that the svmRadial model achieved the highest AUC value of 0.95. Additionally, both ensembleGlmnet and ensembleLinear models performed well, with an AUC value of 0.9. The glmnet, random forest, and svmLinear models achieved an AUC of 0.85 (Supplementary Figure S9).

## 4. Discussion

Earlier studies [36] have shown that alterations in DNA methylation can contribute to the development of Huntington’s disease (HD). However, the relationship between methylation and the metabolome in HD has not been extensively studied. The goal of our study was to investigate the potential interaction between DNA methylation and the metabolome in the human brain of HD individuals compared to controls. We analyzed the metabolome in two brain regions, the striatum and frontal lobe, and performed methylome profiling only in the frontal lobe. 

Environmental factors can lead to epigenetic changes, and metabolites can act as interacting partners that determine cellular function. Even minor and short-term changes in nutrition can have significant and long-lasting effects on gene expression, possibly by interacting with epigenetic factors. This “memory” of past metabolic conditions may also contribute to the development of certain diseases over time [37]. We hypothesized that there exists a robust connection between DNA methylation patterns and metabolic measures. We aimed to discover diagnostic indicators and understand the methylome–metabolome interplay in HD. Our study reveals the complex relationship between the epigenome and metabolome, potentially impacting HD development.

Our study highlights the complex interplay between the epigenome and metabolome, which may play a role in the development of HD.

Analysis of the acquired data showed that there was a significant correlation between phenylalanine and methionine, and the CpGs of *GPC6*, *CSNK1G3*, and *PCNX* genes. While phenylalanine is not directly involved in the development of HD or the formation of protein aggregates, it can influence the levels of neurotransmitters, which are critical in the pathogenesis of HD [38,39]. Similarly, while methionine is not directly implicated in the development of HD, it may play a role in the neurodegeneration [40,41] and the pathogenesis of HD through its involvement in the generation of reactive oxygen species [42] and impaired homocysteine metabolism [43,44]. According to previously reported research, GPC6 is a significant protein involved in synapse formation [45]. *CSNK1G3* is a member of the casein kinase family of genes, which play a critical role in neuronal and synaptic network functions. It has been studied in relation to neurodegenerative disorders, including HD [46]. Meanwhile, *PCNX* is a member of the pecanex family of genes, which act as maternal-effect neurogenic genes studied in Drosophila. This gene is thought to potentially contribute to nervous system development [47]. Additionally, genes including TRAPPC10, CSMD3, MTRR, G6PC2, PCDH7, PCED1B, RPS25, and TRAPPC4 were found to be highly correlated with phenylalanine. Specifically, CSMD3 has been linked to synaptogenesis and neurogenesis in the context of neurodevelopmental disorders. This gene may also play a role in regulating a network of genes involved in synaptic organization and immune activity [48]. The *MTRR* gene encodes for 5-methyltetrahydrofolate-homocysteine methyltransferase reductase, which is necessary for the normal function of the *MTHFR* gene. Dysregulation of S-adenosyl methionine, methionine, and homocysteine, which are all involved with the *MTHFR* gene, can lead to neurotoxicity [18,49]. *PCDH7* has been shown to play a role in regulating dendritic spine morphology and synaptic function [50]. *RPS25* participates in nucleotide repeat expansions through the regulation of an unconventional translation process that is commonly associated with the pathogenesis of Huntington’s disease [51,52]. Our study identified methylation on the promoter region of the *TRAPPC4* gene, which has been associated with splice variants linked to a neurodegenerative syndrome in infants. This finding suggests a possible involvement of TRAPPC4 in the neurodegenerative process [53].

### Aminoacyl-tRNA Biosynthesis

Metabolic enrichment analysis highlighted the aminoacyl-tRNA biosynthesis pathway as being significantly perturbed. This signaling mechanism plays a crucial role in protein synthesis and in ensuring the precision of translation [54,55]. It is noteworthy that a study utilizing cerebrospinal fluid identified a strong association of aminoacyl-tRNA biosynthesis with HD [39]. Several studies have also suggested that cytosolic tRNAs play a crucial role in HD [56,57]. Elongated Gln repeat in the huntingtin protein, consisting of 40–100 repeated CAG codons, is known to cause HD. However, shorter CAG repeats can also contribute to HD, indicating the involvement of additional disease modifiers. The constant process of translation of the repeat sequence depletes charged tRNA^Gln^–CUG, resulting in more frequent frameshifting translation of the huntingtin gene, which can exacerbate HD pathogenesis. The process also signifies the role of tRNA in the disease process [56,57,58]. Our study investigated the potential impact of epigenetic modifications, specifically DNA methylation, on the above-mentioned metabolomic changes in the striatum and frontal lobe regions of the brain in individuals with HD.

In our study, the aminoacyl-tRNA biosynthesis pathway was found to show a correlation between valine and phenylalanine with two CpGs of *SEPSECS* gene. Valine plays a vital role in protein synthesis and various cellular functions and has been found to be associated with HD. A study observed lower levels of valine in the HD patient group compared to the control group. The study also found a correlation between valine levels, weight loss, and the number of CAG repeats [59]. Also, the decreased levels of phenylalanine were identified, along with valine, in postmortem brain tissues, CSF, and serum samples of HD patients [39].

The *SEPSECS* gene codes for the enzyme Sep tRNA:Sec tRNA Synthase, which synthesizes selenocysteine. This unique amino acid has antioxidants and appears in proteins for oxidative stress defense and redox signaling [60,61]. Several studies have suggested that oxidative stress and damage contribute to the pathogenesis of HD, especially in the striatum and cerebral cortex [62,63,64]. In this context, it has been proposed that selenocysteine and other antioxidants might have neuroprotective effects against the oxidative stress that occurs in HD.

Moreover, recent evidence suggests that alterations in the expression of the *SEPSECS* gene and the levels of selenocysteine in the brain might be involved in the pathogenesis of HD [65,66,67]. Selenoproteins have been found to participate in various biological processes, such as antioxidant defense, anti-inflammation, anti-apoptosis, and immune response regulation, as well as the regulation of oxidative stress and endoplasmic reticulum stress [68]. A study by Lu et al., 2014 suggests that, when sodium selenite was given, mouse models of HD showed marked improvements in motor endurance, decreased loss of brain weight, reduced burden of mutant huntingtin aggregates, and decreased levels of brain oxidized glutathione [69]. In summary, the *SEPSECS* gene and its product selenocysteine might play an important role in the pathogenesis of HD by regulating oxidative stress and neuroprotection. Further research is needed to fully understand the mechanisms underlying the association of the metabolites noted in our study with this gene and to explore their potential therapeutic applications in HD. Notably, there were perturbations in the aminoacyl-tRNA biosynthesis pathway, which is linked to protein synthesis and translation precision [54,55]. A previous study, which used cerebrospinal fluid, also connected this pathway with HD [39]. Cytosolic tRNAs are implicated in HD, with the elongated Gln repeat in the huntingtin protein driving the disease process [56,57,58]. One of the genes, the *SEPSECS* gene, responsible for producing the Sep (O-Phosphoserine) tRNA:Sec (Selenocysteine) tRNA Synthase enzyme, is a focal point. This enzyme facilitates selenocysteine synthesis, a rare amino acid with antioxidant properties [60,61]. Given the oxidative stress implications in HD pathogenesis, exploring selenocysteine’s neuroprotective potential is significant [62,63,64]. Our study sheds light on these interconnected mechanisms and their contribution to HD’s complex pathology.

In conclusion, we have used an epimetabolomics approach to highlight the significance of the aminoacyl-tRNA biosynthesis pathway in the development of HD. However, this study was performed on a limited number of samples, and this is due to the difficulty of obtaining PM brain tissue. However, as a proof-of-concept study, we aimed to demonstrate the feasibility of the proposed methodology combining metabolomics and DNA methylation, which is the first of its kind in studying the pathogenesis of HD. For the analysis of methylome, we have considered known, potentially confounding, factors, such as sample age, gender, estimated proportion of neurons, and postmortem delay. The effects of nutrition or stress are assumed to be equally distributed between sample groups, and they should not have a significant impact on the outcome. We have also experimented with the use of RUV [70], the method used to estimate unknown confounding variables, but that did not impact the outcome. Further, we emphasize that the regulation of metabolites and genes is tightly intertwined and plays a critical role in maintaining cellular homeostasis in the brain. Dysregulation of these processes can lead to the accumulation of toxic metabolites, oxidative stress, and inflammation, which contribute to the development and progression of HD. Understanding the complex interplay between metabolites and genes in HD has the potential to uncover novel therapeutic targets for this devastating disease. We suggest that these biomarkers of HD should be the focus of future translational research efforts.

## Figures and Tables

**Table 1:** A comparison of demographic features between individuals diagnosed with Huntington’s disease and control subjects without the condition.

	HD Patients	Controls	*p*-Value
Number of subjects	14	14	n/a
Age, Mean (SD)	54.64 (12.39)	78.5 (13.46)	<0.0001
Individual age in years:			
Patient/control–1	70	84	
Patient/control–2	57	84	
Patient/control–3	51	81	
Patient/control–4	52	87	
Patient/control–5	67	90	
Patient/control–6	51	89	
Patient/control–7	33	89	
Patient/control–8	47	54	
Patient/control–9	48	53	
Patient/control–10	na	84	
Patient/control–11	50	60	
Patient/control–12	68	89	
Patient/control–13	72	83	
Patient/control–14	75	90	
Sex			
Males	8 (57.1)	8 (57.1)	0.45
Females	6 (42.8)	6 (42.8)	
Postmortem delay (PMD)-Minutes			
Mean (SD)	77.35 (71.63)	69.28 (38.09)	0.65

## Data Availability

Data will be provided upon request to corresponding author.

## Supplementary Materials

The following supporting information can be downloaded at: https://www.mdpi.com/article/10.3390/genes14091752/s1. Supplementary Table S1: Striatum-Metabolites. Supplementary Table S2: Frontal-Metabolites. Supplementary Table S3: EPIC-Enrichment of genomic region. Supplementary Table S4: EPIC-DMCs. Supplementary Table S5: Epimetabolomics-Pathway-Interactions. Supplementary Table S6: Interaction of metabolites and CpGs of sig pathway. Supplementary Figure S1: Metabolomics-Striatum-Variance inflation. Supplementary Figure S2: Metabolomics-Frontal-Variance inflation. Supplementary Figure S3: Metabolomics-Striatum-Normalization. Supplementary Figure S4: Metabolomics-Frontal-Normalization. Supplementary Figure S5: Epic array variance inflation. Supplementary Figure S6: EPIC-Enrichment of genomic region. Supplementary Figure S7: Striatum-Metabolomics-Predictor. Supplementary Figure S8: Striatum-Metabolomics-Predictor. Supplementary Figure S9: Frontal lobe-Methylation-Predictor.
