# Supplementary material for: Integrative Analysis Unveils the Correlation of Aminoacyl-tRNA Biosynthesis Metabolites with the Methylation of the SEPSECS Gene in Huntington’s Disease Brain Tissue
Source: Genes (Basel). 2023 Sep 2;14(9):1752. doi: 10.3390/genes14091752 (PMC10530570; doi:10.3390/genes14091752)
Supplement: Supplementary file 1 [file genes-14-01752-s001.zip › 9-HD-Supple Table 4- EPIC-DMCs.pdf]

ID	logFC	AveExpr	t	P.Value	adj.P.Val
cg17214100	-0.886204884	1.304366858	-8.52980544	1.29829E-08	0.009196867
cg21114836	-0.791815647	3.441244047	-7.487396329	1.23215E-07	0.030923513
cg11513962	-0.660219501	4.107970995	-7.218211067	2.25538E-07	0.030923513
cg03437106	-1.116279939	-0.33275447	-7.184186113	2.43613E-07	0.030923513
cg12653894	-0.669674057	3.026992036	-6.997344113	3.73008E-07	0.030923513
cg20901257	-0.759577762	3.507830884	-6.920582581	4.44933E-07	0.030923513
cg16243402	-0.833306269	1.420232909	-6.872860976	4.96667E-07	0.030923513
cg02921735	-0.716123508	3.371230505	-6.832257006	5.45519E-07	0.030923513
cg15938442	-0.811364533	1.665682702	-6.782963363	6.11498E-07	0.030923513
cg16496450	-0.69538502	3.017761083	-6.717538615	7.11886E-07	0.030923513
cg02974898	-0.643692956	-0.268220083	-6.715911836	7.14586E-07	0.030923513
cg04689698	-0.991549557	-1.040191709	-6.629335242	8.7453E-07	0.030923513
cg03309025	-0.912331182	3.005910054	-6.622143034	8.89364E-07	0.030923513
cg04769423	-0.530253593	3.097850592	-6.603172147	9.29741E-07	0.030923513
cg22265078	-0.994117173	2.744555035	-6.591035092	9.56551E-07	0.030923513
cg11406453	-0.900550878	-1.890098128	-6.589655484	9.59648E-07	0.030923513
cg06126727	-0.57929999	3.208708015	-6.558204365	1.0331E-06	0.030923513
cg14982506	-0.666349671	3.561206401	-6.51958523	1.13122E-06	0.030923513
cg00403661	-0.774730354	2.957438072	-6.515311468	1.14264E-06	0.030923513
cg01417946	-0.760604037	2.672105059	-6.48377424	1.2307E-06	0.030923513
cg06605063	-0.669724016	4.066616968	-6.480065548	1.2415E-06	0.030923513
cg02706742	-1.105302462	-3.197911588	-6.461639932	1.29662E-06	0.030923513
cg11541225	-0.813262803	3.14714646	-6.457232597	1.31017E-06	0.030923513
cg10244976	-0.702551173	-0.998312668	-6.442856431	1.35538E-06	0.030923513
cg15935247	-0.71279227	-1.596302928	-6.440368471	1.36336E-06	0.030923513
cg13552681	-1.139373895	0.329046204	-6.403407181	1.48781E-06	0.030923513
cg02488942	-0.65353809	-2.06574673	-6.384597792	1.55553E-06	0.030923513
cg20770936	-0.605461055	4.276964208	-6.38227128	1.56412E-06	0.030923513
cg09085411	-1.040987319	1.884806454	-6.327892045	1.77937E-06	0.030923513
cg20046743	-0.703444729	-0.915744928	-6.301585834	1.89413E-06	0.030923513
cg14300418	-0.796302689	2.384097266	-6.298095193	1.90991E-06	0.030923513
cg18165914	-0.580786094	-0.120475767	-6.278293221	2.00203E-06	0.030923513
cg03591606	-1.118822338	-2.708802101	-6.274877569	2.01837E-06	0.030923513
cg17844690	-0.749969338	2.546785263	-6.263679155	2.07291E-06	0.030923513
cg05779818	-0.654567548	3.398836582	-6.259702839	2.09263E-06	0.030923513
cg03404502	-1.042659598	3.141557707	-6.23812919	2.20304E-06	0.030923513
cg06859734	-0.541384106	3.93157464	-6.232394695	2.23338E-06	0.030923513
cg07463167	-0.8778201	1.754436516	-6.229411406	2.24932E-06	0.030923513
cg10164186	-1.001487306	2.475278126	-6.228709292	2.2531E-06	0.030923513
cg21529827	-0.875194667	2.039869063	-6.228282949	2.25539E-06	0.030923513
cg18733925	-0.607331417	3.336745627	-6.218531137	2.30848E-06	0.030923513
cg14415559	-0.864479286	2.051453831	-6.212702645	2.34083E-06	0.030923513
cg20576989	-0.601065347	3.942620706	-6.192983615	2.45371E-06	0.030923513
cg05297929	-0.617376772	4.380491553	-6.176015366	2.55528E-06	0.030923513
cg12157852	-0.748636396	0.662016022	-6.172173271	2.57887E-06	0.030923513
cg09470907	1.850944973	1.520469316	6.166400686	2.61473E-06	0.030923513

cg00798167	-0.673980945	3.452778383	-6.145987268	2.74566E-06	0.030923513
cg08413196	-0.460100975	4.064793615	-6.13985971	2.78625E-06	0.030923513
cg08418508	-0.603245499	3.241757868	-6.135107447	2.81816E-06	0.030923513
cg22934200	-0.631656929	-0.512663218	-6.131024353	2.84586E-06	0.030923513
cg03321196	-0.830941287	-0.17754014	-6.129826529	2.85404E-06	0.030923513
cg19208718	-0.564944564	3.924102033	-6.122014297	2.90799E-06	0.030923513
cg22882201	0.771417481	-4.378689343	6.111507197	2.98219E-06	0.030923513
cg22747531	-0.680026536	3.060622832	-6.110635397	2.98843E-06	0.030923513
cg16650125	-0.598608053	4.316589824	-6.110040089	2.9927E-06	0.030923513
cg16141839	-0.903798946	2.456317244	-6.105500288	3.02547E-06	0.030923513
cg06014765	-0.760057117	3.267761421	-6.10500261	3.02909E-06	0.030923513
cg10406360	-0.519802653	0.023716641	-6.094413339	3.10705E-06	0.030923513
cg02534444	-0.57967434	3.874457116	-6.090615927	3.1355E-06	0.030923513
cg17080740	-0.604591374	0.118866845	-6.086251559	3.16853E-06	0.030923513
cg19762178	-0.611782556	3.731935006	-6.085351998	3.17538E-06	0.030923513
cg05324590	-0.949204863	2.117329003	-6.078720765	3.22636E-06	0.030923513
cg08998414	-0.648636047	2.791463961	-6.073389538	3.26795E-06	0.030923513
cg23483562	-0.520613614	3.547053517	-6.072020149	3.27872E-06	0.030923513
cg09784342	0.78683536	-2.541109964	6.063991886	3.34259E-06	0.030923513
cg19427807	-0.8323054	2.568366825	-6.06013783	3.3737E-06	0.030923513
cg00833747	-0.61232684	-0.692159337	-6.057861911	3.39221E-06	0.030923513
cg26570804	-0.802723275	0.938432522	-6.052771805	3.43399E-06	0.030923513
cg10411510	-0.732817445	0.995213611	-6.050547097	3.45241E-06	0.030923513
cg14066626	-0.681716126	-1.76399012	-6.049194916	3.46366E-06	0.030923513
cg01726273	-0.788663207	-1.446587963	-6.046603957	3.48531E-06	0.030923513
cg19177422	-0.768656355	3.076720063	-6.035369709	3.5808E-06	0.030923513
cg13535449	-0.608805684	3.803138795	-6.033310692	3.59859E-06	0.030923513
cg06910290	-0.634674543	-0.043490268	-6.025372893	3.66802E-06	0.030923513
cg22084647	-0.667627535	3.828461882	-6.02282471	3.6906E-06	0.030923513
cg20237890	-0.649535676	3.668374111	-6.022456672	3.69387E-06	0.030923513
cg04639988	-0.660905092	-2.193142079	-6.011126577	3.79606E-06	0.030923513
cg01759026	-0.686262562	2.13389608	-6.007694075	3.82759E-06	0.030923513
cg24602838	-0.697726417	0.565576575	-5.990326134	3.99124E-06	0.030923513
cg14799129	-0.795138386	2.334153908	-5.981206876	4.08E-06	0.030923513
cg11541587	-0.826256559	3.459594024	-5.979685759	4.095E-06	0.030923513
cg22843026	-0.546014845	3.792121447	-5.979650219	4.09535E-06	0.030923513
cg17625635	0.791411808	2.88421883	5.974685884	4.1447E-06	0.030923513
cg15443378	-0.631231439	3.506200715	-5.967681808	4.21536E-06	0.030923513
cg02947772	-0.547757911	3.637183465	-5.964598693	4.24685E-06	0.030923513
cg06636431	-0.632948835	3.958610845	-5.963438002	4.25877E-06	0.030923513
cg00159230	-0.6758577	3.541359487	-5.958899196	4.30569E-06	0.030923513
cg07632939	-0.644345882	-0.101698673	-5.958495646	4.30989E-06	0.030923513
cg08943895	-0.678742082	2.602067061	-5.95748696	4.3204E-06	0.030923513
cg04261937	-0.942935761	1.454907622	-5.954960859	4.34684E-06	0.030923513
cg12869362	-0.584850182	-3.081725417	-5.951409365	4.38429E-06	0.030923513
cg19083491	-0.782474126	3.160264117	-5.950171076	4.39742E-06	0.030923513
cg27596715	-0.704986316	3.223426305	-5.94947787	4.40479E-06	0.030923513

cg01929407	-0.628332407	3.125614965	-5.943413484	4.4698E-06	0.030923513
cg24450692	-0.692425045	-1.10444255	-5.930667683	4.60965E-06	0.030923513
cg21485602	-0.815059684	2.182353134	-5.929724134	4.62017E-06	0.030923513
cg02611479	-0.750776039	1.785060815	-5.927340023	4.64688E-06	0.030923513
cg00387738	-0.584329481	3.540074834	-5.924507907	4.67882E-06	0.030923513
cg25829942	-0.570727498	3.981503487	-5.923633235	4.68872E-06	0.030923513
cg02800607	-0.747423572	-2.902519114	-5.91404416	4.79875E-06	0.030923513
cg04800555	-1.058098396	2.681286405	-5.913193351	4.80864E-06	0.030923513
cg03763457	-0.682953994	3.115864766	-5.907662823	4.87342E-06	0.030923513
cg14099387	-0.797710797	2.551079256	-5.905645001	4.89728E-06	0.030923513
cg15758896	-0.513408588	-0.580798972	-5.904281041	4.91347E-06	0.030923513
cg25969318	-0.712034129	3.963538189	-5.898912735	4.97774E-06	0.030923513
cg08371532	-0.619892201	-0.758052064	-5.898536365	4.98227E-06	0.030923513
cg06261400	-0.897879556	2.247125707	-5.897439677	4.99552E-06	0.030923513
cg14297991	-0.558072829	3.640714536	-5.893376815	5.0449E-06	0.030923513
cg00105080	-0.957962619	2.201731671	-5.893090355	5.0484E-06	0.030923513
cg23791672	-0.796157344	0.552430897	-5.889988797	5.08646E-06	0.030923513
cg03807888	-0.589502971	3.99683858	-5.879843007	5.21302E-06	0.030923513
cg26740375	-0.646547014	2.30617629	-5.874700948	5.27838E-06	0.030923513
cg15973546	-0.494092954	-0.212341562	-5.87097619	5.32624E-06	0.030923513
cg25275452	-0.55206308	3.540695682	-5.868208939	5.36209E-06	0.030923513
cg18106045	-0.951149798	2.256858834	-5.867326639	5.37357E-06	0.030923513
cg08708389	-0.75069575	3.008783794	-5.866499163	5.38436E-06	0.030923513
cg10106639	-1.021016094	1.874247298	-5.866152352	5.38889E-06	0.030923513
cg26055718	-0.7589398	1.507717417	-5.858466026	5.49025E-06	0.030923513
cg00472411	-0.712072246	3.365321729	-5.849357784	5.61289E-06	0.030923513
cg06047629	-0.796193811	2.188972659	-5.846155357	5.65667E-06	0.030923513
cg12876349	-1.078397043	-4.492423541	-5.843893297	5.6878E-06	0.030923513
cg14931396	-0.786222625	1.231446688	-5.834463792	5.81946E-06	0.030923513
cg00556627	-0.658110713	0.645214572	-5.829496857	5.89005E-06	0.030923513
cg09976979	-0.580593937	3.893174542	-5.827430489	5.91968E-06	0.030923513
cg18792689	-1.1606698	2.622537973	-5.827413583	5.91992E-06	0.030923513
cg19511263	-0.586536661	3.354637473	-5.825619208	5.94577E-06	0.030923513
cg05357137	-0.63475567	3.768760132	-5.82215469	5.996E-06	0.030923513
cg08194369	-0.715210052	1.325292666	-5.818860977	6.04416E-06	0.030923513
cg06334689	5.368465261	-1.318044504	5.813705437	6.12033E-06	0.030923513
cg05508408	-0.645952638	0.745467354	-5.810514323	6.16797E-06	0.030923513
cg08436419	-0.618333203	-1.564148731	-5.810360153	6.17028E-06	0.030923513
cg14242526	-0.656121005	4.003456739	-5.80820708	6.20264E-06	0.030923513
cg23527183	-0.913050629	2.410388807	-5.798990449	6.34315E-06	0.030923513
cg08595782	-0.677250725	-0.388565717	-5.797490446	6.36632E-06	0.030923513
cg16354717	-0.62600971	3.114887681	-5.797485497	6.3664E-06	0.030923513
cg21916555	-0.748360838	2.821121898	-5.797049369	6.37315E-06	0.030923513
cg09414827	-0.670440712	-0.276704266	-5.792554687	6.44317E-06	0.030923513
cg17242574	-0.637411535	0.500467773	-5.788962033	6.49971E-06	0.030923513
cg03426940	-0.529795787	0.839478376	-5.787920221	6.5162E-06	0.030923513
cg00896578	-0.604534851	4.012636332	-5.786000873	6.54668E-06	0.030923513

cg19523639	-0.710028927	2.795806515	-5.784888907	6.56441E-06	0.030923513
cg27107110	-0.739217761	1.506412488	-5.784259148	6.57447E-06	0.030923513
cg24657503	-0.750809921	-1.560136907	-5.779797241	6.64622E-06	0.030923513
cg24407907	-0.707444468	3.44903697	-5.777158407	6.68902E-06	0.030923513
cg21737306	-0.847254105	1.712310494	-5.77669769	6.69652E-06	0.030923513
cg24128226	-0.828857474	2.03164695	-5.772184191	6.77047E-06	0.030923513
cg23852964	-0.74938958	3.602197091	-5.7715365	6.78115E-06	0.030923513
cg17138594	-1.013587575	2.254967235	-5.769069847	6.82198E-06	0.030923513
cg01193159	-1.014634273	1.814366848	-5.768611361	6.8296E-06	0.030923513
cg21963023	-0.644799042	2.37439282	-5.757818278	7.01141E-06	0.030923513
cg02093477	-0.714328698	2.673982618	-5.75768308	7.01372E-06	0.030923513
cg18729133	-0.653910768	3.27854986	-5.755471959	7.05158E-06	0.030923513
cg06094512	-0.636499036	3.400401707	-5.754119365	7.07484E-06	0.030923513
cg17673914	-0.849997076	0.061709469	-5.75130221	7.12355E-06	0.030923513
cg03764900	-0.705981941	2.264926648	-5.74763431	7.18747E-06	0.030923513
cg24289966	-0.548541984	-1.202658113	-5.741383565	7.29775E-06	0.030923513
cg18479241	-0.791493619	3.526737426	-5.74053639	7.31283E-06	0.030923513
cg07354850	-0.734173591	2.899304588	-5.739874667	7.32463E-06	0.030923513
cg14229516	-0.54248423	4.01894308	-5.735828465	7.3972E-06	0.030923513
cg12263409	0.450520387	0.418431087	5.735700306	7.39951E-06	0.030923513
cg03053370	-0.869058453	2.334249078	-5.733282118	7.44325E-06	0.030923513
cg13868001	-0.802638547	-0.969365198	-5.731444253	7.47666E-06	0.030923513
cg04296137	-0.709324499	3.167374747	-5.726258164	7.57178E-06	0.030923513
cg18012213	-0.602484796	3.574876285	-5.724150103	7.61079E-06	0.030923513
cg22311408	-0.784468797	-0.021459255	-5.720022993	7.68776E-06	0.030923513
cg18833779	-0.835611077	2.328274576	-5.717982837	7.7261E-06	0.030923513
cg01369276	-0.489951674	3.658504668	-5.715991869	7.76371E-06	0.030923513
cg19503307	-0.607324475	0.528637019	-5.715157863	7.77951E-06	0.030923513
cg13272108	0.747514185	-0.841205213	5.707860514	7.91922E-06	0.030923513
cg01775781	-0.821146732	1.737870464	-5.707827606	7.91986E-06	0.030923513
cg17677381	-0.587640973	3.71718982	-5.703875513	7.99659E-06	0.030923513
cg08786470	-0.903574444	3.359988493	-5.698781511	8.09661E-06	0.030923513
cg14812743	-0.66371979	3.573743449	-5.698049816	8.11108E-06	0.030923513
cg27208806	-0.879243301	1.903794793	-5.692875447	8.21416E-06	0.030923513
cg04500903	-0.765813859	2.982913825	-5.686817815	8.33653E-06	0.030923513
cg09894683	-1.19710998	-1.088412178	-5.67801535	8.51766E-06	0.030923513
cg05823545	-0.755315224	2.257532887	-5.677331856	8.53189E-06	0.030923513
cg20180538	-0.663300448	3.714687049	-5.675979285	8.56012E-06	0.030923513
cg04505845	-0.614913771	-0.343126466	-5.671549311	8.65325E-06	0.030923513
cg13921308	-0.795392925	1.014961967	-5.669519532	8.69626E-06	0.030923513
cg02331902	-1.222467054	-3.660751342	-5.669290504	8.70113E-06	0.030923513
cg27022171	-0.655145762	-0.46372579	-5.667742537	8.7341E-06	0.030923513
cg22636442	-0.486193262	3.105183077	-5.666910691	8.75187E-06	0.030923513
cg23687909	-0.818860134	2.79263086	-5.666737943	8.75556E-06	0.030923513
cg20712946	-0.617784444	3.657742247	-5.663958996	8.81522E-06	0.030923513
cg12480424	-0.559524955	3.553518407	-5.660317043	8.89402E-06	0.030923513
cg13880897	-0.643731356	2.438923862	-5.654396162	9.02367E-06	0.030923513

cg04424246	-0.681485655	3.315267885	-5.650766618	9.10409E-06	0.030923513
cg01287200	-0.56062863	3.717716201	-5.6470019	9.18827E-06	0.030923513
cg13150094	-0.639860672	3.138714267	-5.646723237	9.19453E-06	0.030923513
cg21147040	-0.626811154	1.162798786	-5.646194419	9.20643E-06	0.030923513
cg13029013	-0.597718456	-0.601346074	-5.646115709	9.2082E-06	0.030923513
cg26453456	-0.808960094	-2.141778695	-5.641868346	9.30434E-06	0.030923513
cg07868561	-0.563689055	0.3712308	-5.641834254	9.30511E-06	0.030923513
cg25363192	-0.711028685	2.918299227	-5.640800491	9.32867E-06	0.030923513
cg11661454	-0.951569845	2.216564378	-5.640631474	9.33252E-06	0.030923513
cg11546830	-0.574395349	3.975170987	-5.638198321	9.38823E-06	0.030923513
cg08247527	-0.677848516	3.574740956	-5.634868653	9.465E-06	0.030923513
cg03520833	-0.711634584	-0.377627191	-5.633201961	9.50367E-06	0.030923513
cg01536306	-0.634487955	3.715148707	-5.632059429	9.53027E-06	0.030923513
cg20284699	-0.609215329	-1.813708033	-5.630121155	9.57557E-06	0.030923513
cg22157320	-0.572743366	3.754633458	-5.627390425	9.63976E-06	0.030923513
cg07823855	-0.716789222	3.339391525	-5.626913221	9.65102E-06	0.030923513
cg02839514	-0.779038148	2.787041372	-5.62616752	9.66865E-06	0.030923513
cg05704719	-0.65394037	3.250810922	-5.625292293	9.68938E-06	0.030923513
cg20322908	-0.58832496	3.09005152	-5.624510972	9.70792E-06	0.030923513
cg22788747	-0.583204555	-0.868427485	-5.622023574	9.7672E-06	0.030923513
cg14332653	-0.837678155	1.69879111	-5.621428173	9.78144E-06	0.030923513
cg24368124	-0.830482691	1.783742552	-5.618176431	9.8596E-06	0.030923513
cg14432334	-0.739777962	3.525395468	-5.614481058	9.94919E-06	0.030923513
cg21180839	-0.86261876	1.430245221	-5.606681159	1.0141E-05	0.030923513
cg26794993	-0.898148994	3.004193232	-5.606413639	1.01477E-05	0.030923513
cg17608635	-0.896108524	-0.038702713	-5.60520198	1.01778E-05	0.030923513
cg07786995	-0.880480057	-1.507085169	-5.600884872	1.0286E-05	0.030923513
cg08571020	-0.749069305	0.762900226	-5.600668429	1.02914E-05	0.030923513
cg23390381	-0.799733097	1.962835172	-5.597168847	1.038E-05	0.030923513
cg08244182	-0.711370397	3.168323219	-5.596351333	1.04008E-05	0.030923513
cg19844101	0.734720102	3.08846748	5.595301063	1.04276E-05	0.030923513
cg00877691	-0.703212312	0.999116947	-5.59465029	1.04443E-05	0.030923513
cg12575659	-0.677321067	-2.282489824	-5.591325898	1.05297E-05	0.030923513
cg09371284	-0.604220239	-0.346594719	-5.590047107	1.05627E-05	0.030923513
cg23051063	-0.599454723	3.543803747	-5.587475207	1.06295E-05	0.030923513
cg10052687	-0.59948262	-0.208510893	-5.586883151	1.06449E-05	0.030923513
cg16889782	-0.798333273	2.78766942	-5.58565944	1.06769E-05	0.030923513
cg09370405	0.776451194	-2.803711453	5.583322471	1.07382E-05	0.030923513
cg09836433	-0.866792049	1.495549638	-5.580096297	1.08235E-05	0.030923513
cg03289264	-0.595069575	3.425314112	-5.579676352	1.08346E-05	0.030923513
cg23689129	-0.614510628	3.47360168	-5.577024339	1.09053E-05	0.030923513
cg14308466	-0.676271619	-1.071997393	-5.576139712	1.09289E-05	0.030923513
cg11086428	-0.623785674	3.298680047	-5.574889052	1.09625E-05	0.030923513
cg05302350	-0.955767759	-1.466402782	-5.572819151	1.10183E-05	0.030923513
cg11546574	-0.472547014	3.290504457	-5.569850229	1.10988E-05	0.030923513
cg02430558	-0.534153401	-0.977357915	-5.569643845	1.11044E-05	0.030923513
cg07011981	-0.800534678	0.968677693	-5.569450298	1.11097E-05	0.030923513

cg11576468	-0.576889061	1.647168336	-5.568392574	1.11385E-05	0.030923513
cg17918348	-0.60258713	3.373563994	-5.56658321	1.1188E-05	0.030923513
cg12390678	-0.833323247	3.096645395	-5.565109418	1.12285E-05	0.030923513
cg10031008	-0.605323815	-0.811797839	-5.564458408	1.12465E-05	0.030923513
cg17969913	-0.506598193	3.627600547	-5.564175754	1.12543E-05	0.030923513
cg15800340	-0.569839483	3.692873464	-5.562811954	1.1292E-05	0.030923513
cg23368416	-0.70387724	3.417009971	-5.562252388	1.13075E-05	0.030923513
cg04891601	-0.826766554	2.72519792	-5.561955722	1.13157E-05	0.030923513
cg20095390	-0.578147987	2.644440896	-5.556482595	1.14687E-05	0.030923513
cg27507372	-0.723607705	0.223963886	-5.555733792	1.14897E-05	0.030923513
cg06105694	-0.501658729	-1.404198489	-5.555421905	1.14985E-05	0.030923513
cg13111622	-0.668404024	0.287371921	-5.553819275	1.15438E-05	0.030923513
cg23585102	-0.563744316	2.780852696	-5.553080887	1.15648E-05	0.030923513
cg16962259	-0.65845692	3.434675339	-5.552929252	1.15691E-05	0.030923513
cg15187139	-0.592082853	3.538460105	-5.552019667	1.15949E-05	0.030923513
cg27382588	-0.633911057	3.898997315	-5.550026435	1.16518E-05	0.030923513
cg16954761	-0.61616484	3.811500116	-5.548351392	1.16997E-05	0.030923513
cg27510497	-0.636422092	3.259370945	-5.54823954	1.1703E-05	0.030923513
cg12969108	-0.753766859	3.344271227	-5.547306143	1.17298E-05	0.030923513
cg03487391	-0.658162406	-1.754657293	-5.547239453	1.17317E-05	0.030923513
cg06899018	-0.572220207	3.729600389	-5.542393749	1.1872E-05	0.030923513
cg20961014	-0.549274024	3.511080337	-5.539564021	1.19548E-05	0.030923513
cg03976244	-0.564371682	-0.172599077	-5.537596523	1.20127E-05	0.030923513
cg10659171	-0.748029942	1.009523987	-5.537575829	1.20133E-05	0.030923513
cg19282236	-0.519125179	0.607750239	-5.536752333	1.20376E-05	0.030923513
cg17507646	-0.840361377	2.259981919	-5.536716056	1.20387E-05	0.030923513
cg15258175	-0.670240034	1.002376202	-5.534128631	1.21154E-05	0.030923513
cg19308375	-0.623122065	0.511142922	-5.534100157	1.21162E-05	0.030923513
cg00762354	-0.72845831	0.854405198	-5.534092621	1.21164E-05	0.030923513
cg00182781	-0.683453248	2.962182466	-5.533933782	1.21212E-05	0.030923513
cg00010743	-0.753632546	2.779575347	-5.533286712	1.21404E-05	0.030923513
cg00773582	-0.737293626	2.677029564	-5.531616826	1.21903E-05	0.030923513
cg02267341	-0.531647492	2.909477108	-5.531245489	1.22014E-05	0.030923513
cg26718363	-0.670639085	3.610791337	-5.530971665	1.22096E-05	0.030923513
cg27496965	-0.623709978	3.801869845	-5.528918736	1.22713E-05	0.030923513
cg16776821	0.634822458	4.021170449	5.52843404	1.22859E-05	0.030923513
cg22930677	-0.715059527	3.133880442	-5.528383116	1.22875E-05	0.030923513
cg18851108	-1.059616048	2.500733244	-5.527609211	1.23108E-05	0.030923513
cg11490667	-0.684578645	3.439870304	-5.527524095	1.23134E-05	0.030923513
cg21057130	-0.701823137	3.535346343	-5.527296302	1.23203E-05	0.030923513
cg07869283	-0.722000288	3.632596936	-5.526560408	1.23426E-05	0.030923513
cg18771955	0.570074672	2.226050354	5.524766024	1.23971E-05	0.030923513
cg15105899	-0.719578897	3.433677047	-5.523415365	1.24383E-05	0.030923513
cg07448221	-0.742776975	3.039234806	-5.52190792	1.24844E-05	0.030923513
cg05209405	-0.672743164	-0.925139796	-5.521124179	1.25085E-05	0.030923513
cg11060910	-0.986213888	1.805041565	-5.518710323	1.25829E-05	0.030923513
cg18347227	-0.834861335	-0.322157665	-5.518274445	1.25963E-05	0.030923513

cg23043162	-0.821937167	2.972656551	-5.51701336	1.26354E-05	0.030923513
cg08604181	-0.52098258	3.644673773	-5.512986278	1.2761E-05	0.030923513
cg03774848	-0.677632583	3.201807399	-5.512209477	1.27854E-05	0.030923513
cg09871711	-0.668524356	4.16301501	-5.511496001	1.28078E-05	0.030923513
cg06214728	-0.866932443	2.326179606	-5.511242629	1.28158E-05	0.030923513
cg15725890	-0.767949273	2.267643115	-5.509643168	1.28663E-05	0.030923513
cg21231550	-0.578495656	3.115875684	-5.507848174	1.29231E-05	0.030923513
cg17001457	-0.611804217	3.244820605	-5.506528225	1.29651E-05	0.030923513
cg15955354	-0.717093511	1.876138484	-5.504052589	1.30442E-05	0.030923513
cg21050001	-0.859731102	-1.220995918	-5.503368365	1.30662E-05	0.030923513
cg11727132	-0.79023252	2.819986002	-5.503106379	1.30746E-05	0.030923513
cg07491396	-0.741430843	2.875140931	-5.501810213	1.31163E-05	0.030923513
cg11494887	-0.61753713	3.95803942	-5.499824115	1.31805E-05	0.030923513
cg01775523	-0.613307203	3.157633004	-5.499128516	1.32031E-05	0.030923513
cg00122434	-0.582516686	3.182909498	-5.49678208	1.32794E-05	0.030923513
cg17619799	-0.659466184	3.536661862	-5.496210067	1.32981E-05	0.030923513
cg06743576	-0.593681442	3.52093783	-5.494892954	1.33412E-05	0.030923513
cg00938069	-0.914229602	0.672806654	-5.494074408	1.33681E-05	0.030923513
cg20103222	-0.655419819	3.325256133	-5.492366439	1.34244E-05	0.030923513
cg00701946	-0.722738427	-1.387522429	-5.490648856	1.34812E-05	0.030923513
cg15988727	-0.762332926	3.002898764	-5.489684782	1.35132E-05	0.030923513
cg24931324	-0.825200088	2.555202065	-5.487916679	1.3572E-05	0.030923513
cg27423627	-0.640234832	-0.897983389	-5.487321356	1.35919E-05	0.030923513
cg00223867	-0.654527059	2.569614221	-5.486756368	1.36108E-05	0.030923513
cg14981189	-1.127325017	-0.951017219	-5.484448105	1.36883E-05	0.030923513
cg12756897	-0.830599346	-1.683832586	-5.483858905	1.37081E-05	0.030923513
cg24603573	-0.701455168	3.489918568	-5.477580986	1.39214E-05	0.030923513
cg07710335	-0.477149845	0.104194012	-5.476628875	1.39541E-05	0.030923513
cg20786733	-0.541430079	3.619265201	-5.474276597	1.4035E-05	0.030923513
cg15634474	-0.844010016	3.099472019	-5.473232611	1.40711E-05	0.030923513
cg06561144	-0.681492723	-0.28756387	-5.472155889	1.41085E-05	0.030923513
cg00864346	-0.744753322	2.815492521	-5.469510223	1.42006E-05	0.030923513
cg13238200	-0.736286102	3.886052256	-5.469476403	1.42018E-05	0.030923513
cg01020556	-0.625445059	4.008899675	-5.468769687	1.42265E-05	0.030923513
cg15327692	-0.641128287	2.772032394	-5.467974963	1.42543E-05	0.030923513
cg26076850	-0.629573123	2.224273715	-5.467485361	1.42715E-05	0.030923513
cg17490428	-0.768813724	2.990269468	-5.467206776	1.42813E-05	0.030923513
cg13024807	-0.589408002	-0.285239618	-5.467061006	1.42864E-05	0.030923513
cg14607962	-0.559604225	3.428967276	-5.465103434	1.43554E-05	0.030923513
cg13197283	-0.532926609	-0.952673768	-5.464893208	1.43628E-05	0.030923513
cg17950169	-0.621641721	0.134984686	-5.464842523	1.43646E-05	0.030923513
cg22971407	-0.546269952	2.8810217	-5.463528231	1.44111E-05	0.030923513
cg18947879	-1.110746985	1.649580328	-5.462691199	1.44409E-05	0.030923513
cg19199275	-0.581320501	-0.001425949	-5.462387996	1.44516E-05	0.030923513
cg02135170	-0.878383132	2.797332767	-5.461964965	1.44667E-05	0.030923513
cg26238686	-0.835235598	2.569744846	-5.461433476	1.44856E-05	0.030923513
cg10193350	-0.517244464	3.554480172	-5.459337149	1.45606E-05	0.030923513

cg12219870	-0.562367632	3.740534002	-5.459289572	1.45623E-05	0.030923513
cg00354054	-0.637202482	3.766094157	-5.457415874	1.46296E-05	0.030923513
cg17865653	-0.510660042	4.22595202	-5.452900327	1.47931E-05	0.030923513
cg24844295	-0.49451916	0.949754298	-5.452855352	1.47947E-05	0.030923513
cg20074963	-0.628994907	1.881337344	-5.449781135	1.49071E-05	0.030923513
cg07418518	-0.74976849	2.971813555	-5.44914358	1.49305E-05	0.030923513
cg16865768	-0.683638012	2.690157731	-5.447389584	1.49951E-05	0.030923513
cg15286975	-0.609467277	2.941408656	-5.445912567	1.50498E-05	0.030923513
cg15147658	-0.6881419	2.69429778	-5.445309501	1.50721E-05	0.030923513
cg19538115	-0.550571129	-0.415043375	-5.444193416	1.51136E-05	0.030923513
cg25744610	-0.585599137	4.085728598	-5.444082085	1.51178E-05	0.030923513
cg13297148	-0.548332673	3.515829232	-5.443008631	1.51578E-05	0.030923513
cg23445482	-0.735498128	2.861180976	-5.442896604	1.51619E-05	0.030923513
cg02889110	-0.709072079	2.678728605	-5.440438569	1.5254E-05	0.030923513
cg06535100	-0.592595468	3.623905502	-5.439786698	1.52785E-05	0.030923513
cg07100374	-0.855265294	1.708323917	-5.437196291	1.53763E-05	0.030923513
cg12692440	-0.618595617	3.761780059	-5.436178343	1.54149E-05	0.030923513
cg12760968	-0.69843143	0.730803583	-5.435487209	1.54412E-05	0.030923513
cg03194786	-0.914021444	2.052433718	-5.434484922	1.54793E-05	0.030923513
cg23858471	-0.589433602	3.296018574	-5.43376756	1.55067E-05	0.030923513
cg21961499	-0.626019875	2.561024747	-5.433271815	1.55256E-05	0.030923513
cg19135052	-0.60565483	-0.321284769	-5.429769668	1.56602E-05	0.030923513
cg06631763	-0.753963361	-0.80454613	-5.428741585	1.56999E-05	0.030923513
cg16098545	-0.854173826	0.443866182	-5.427063752	1.57649E-05	0.030923513
cg05584737	-0.795087933	3.379823772	-5.424899534	1.58492E-05	0.030923513
cg24904576	-0.596676393	3.022601291	-5.424805962	1.58528E-05	0.030923513
cg23544066	-0.678749995	3.130690777	-5.418760488	1.60908E-05	0.030923513
cg10775753	-1.025647844	1.973948103	-5.417273441	1.61498E-05	0.030923513
cg23098127	-0.670999926	3.842619828	-5.41583102	1.62074E-05	0.030923513
cg05857143	-0.714254907	-2.063829293	-5.415417186	1.62239E-05	0.030923513
cg10763406	-0.53737599	4.173229462	-5.4146501	1.62546E-05	0.030923513
cg08885892	-0.545328591	4.073530681	-5.413871177	1.62858E-05	0.030923513
cg15120454	-0.634855134	-2.093488266	-5.413266996	1.63101E-05	0.030923513
cg10630087	-1.01406459	2.054038412	-5.411744661	1.63714E-05	0.030923513
cg02637078	-0.775628515	-2.996096692	-5.410832521	1.64083E-05	0.030923513
cg15989892	-0.651791192	-0.745725242	-5.409504945	1.6462E-05	0.030923513
cg00318737	-0.548048375	3.508554322	-5.409024932	1.64815E-05	0.030923513
cg08677613	-0.645124448	2.77531729	-5.405738337	1.66156E-05	0.030923513
cg11141503	-0.704442324	1.54590655	-5.404879854	1.66508E-05	0.030923513
cg14958756	-0.618415885	3.805361104	-5.404834802	1.66527E-05	0.030923513
cg07313980	-0.505520138	3.462227644	-5.404167778	1.66801E-05	0.030923513
cg04102784	-0.531902554	3.661879988	-5.402027834	1.67683E-05	0.030923513
cg23225180	-0.730610261	1.420282658	-5.401345703	1.67965E-05	0.030923513
cg22161131	0.621226631	-3.296392589	5.400723726	1.68223E-05	0.030923513
cg06593811	-0.573664959	3.616902864	-5.400460452	1.68332E-05	0.030923513
cg10320312	-0.794926652	0.317302595	-5.39893752	1.68966E-05	0.030923513
cg16225663	-0.436434877	-0.14938339	-5.3963293	1.70056E-05	0.030923513

cg10799802	-0.711618397	2.572137898	-5.396050714	1.70173E-05	0.030923513
cg11544398	-0.780501319	2.905574032	-5.394036125	1.71021E-05	0.030923513
cg25617519	-0.549944918	3.150045807	-5.393676272	1.71172E-05	0.030923513
cg23609036	-0.777811436	2.565898134	-5.39327287	1.71343E-05	0.030923513
cg20253855	-0.62943145	0.379585425	-5.392225669	1.71786E-05	0.030923513
cg23759367	-0.669587093	2.675485602	-5.392155865	1.71815E-05	0.030923513
cg17014879	-0.681579305	3.750126965	-5.391298568	1.72179E-05	0.030923513
cg18068731	-0.502001923	3.669034044	-5.391005635	1.72304E-05	0.030923513
cg20869637	-0.624128659	2.840489304	-5.390575618	1.72486E-05	0.030923513
cg13730905	-0.677164647	3.419372255	-5.388936843	1.73185E-05	0.030923513
cg22855923	-0.570132482	2.87293177	-5.388644155	1.7331E-05	0.030923513
cg14990116	-0.579600538	3.508906533	-5.388526322	1.73361E-05	0.030923513
cg26759551	-0.691998162	-2.519853007	-5.386751931	1.74121E-05	0.030923513
cg01197090	-0.654406539	3.148598621	-5.386683658	1.7415E-05	0.030923513
cg09677626	-0.796965606	1.416519347	-5.38564199	1.74598E-05	0.030923513
cg01076937	-0.696794553	2.730798443	-5.384951309	1.74896E-05	0.030923513
cg15080232	-0.586425501	3.445556087	-5.384056165	1.75283E-05	0.030923513
cg08128105	-0.578470587	-1.142293288	-5.383996309	1.75309E-05	0.030923513
cg20070029	-0.697283296	3.274029188	-5.383583413	1.75487E-05	0.030923513
cg11776930	-0.581973924	-0.179018577	-5.382211996	1.76082E-05	0.030923513
cg05790038	-1.298716751	1.292947028	-5.381651714	1.76326E-05	0.030923513
cg03911701	-0.54795737	3.372749493	-5.381438382	1.76418E-05	0.030923513
cg24650672	-0.571163775	2.822254029	-5.381034675	1.76594E-05	0.030923513
cg05590053	-0.636466338	0.367522513	-5.380921693	1.76644E-05	0.030923513
cg04962865	-0.72580377	-0.522723773	-5.379715843	1.7717E-05	0.030923513
cg18127823	-0.612029257	0.330723279	-5.379456993	1.77283E-05	0.030923513
cg14159673	-0.602371715	4.494581052	-5.378820192	1.77562E-05	0.030923513
cg08837866	-0.768534537	0.803794881	-5.378696746	1.77616E-05	0.030923513
cg12387480	-0.737885359	3.00308298	-5.378429864	1.77733E-05	0.030923513
cg15770150	-0.613178598	3.576606038	-5.378429521	1.77733E-05	0.030923513
cg19868864	-0.723272196	2.685945141	-5.378354752	1.77766E-05	0.030923513
cg04572706	-0.512715332	0.067864415	-5.376824443	1.78438E-05	0.030923513
cg22235407	-0.769739201	-1.97822351	-5.376396231	1.78627E-05	0.030923513
cg03551199	-0.972691748	1.792055483	-5.375580875	1.78987E-05	0.030923513
cg04106128	-0.781486408	2.215562411	-5.374994856	1.79246E-05	0.030923513
cg16084244	-0.477998149	4.021276872	-5.374609005	1.79417E-05	0.030923513
cg20858324	-0.615237706	3.062653922	-5.373163224	1.80058E-05	0.030958716
cg01011846	-0.519296602	3.955014428	-5.371923796	1.80609E-05	0.030973813
cg05699748	-0.740590483	2.764527406	-5.370261321	1.81352E-05	0.030973813
cg17682316	0.776181548	2.939946407	5.369582432	1.81656E-05	0.030973813
cg20429722	-0.613377409	3.740745414	-5.36782912	1.82444E-05	0.030973813
cg14239477	-0.891818698	2.295409265	-5.367358164	1.82656E-05	0.030973813
cg16602871	-0.485221528	0.078761996	-5.36710765	1.82769E-05	0.030973813
cg04098270	-0.507015863	-3.215989689	-5.365992508	1.83273E-05	0.030984593
cg13445938	-1.067012819	0.135330959	-5.365032897	1.83708E-05	0.030984593
cg17992876	-0.535477767	3.498749391	-5.363892604	1.84225E-05	0.030998127
cg15526954	-0.525104069	-1.58013401	-5.362029225	1.85075E-05	0.031067252

cg06850315	-0.648598079	0.466546815	-5.360586235	1.85735E-05	0.031104414
cg22741129	-0.579794804	3.026509758	-5.358561824	1.86666E-05	0.031186536
cg09302575	-0.636014502	-0.100257296	-5.356835042	1.87463E-05	0.031230302
cg17396522	-0.657719179	3.997928287	-5.356087879	1.8781E-05	0.031230302
cg05750822	-0.592822004	3.73859774	-5.354754467	1.88429E-05	0.031256718
cg19701195	-0.700933437	2.838030632	-5.353848565	1.88851E-05	0.031256718
cg08089583	-0.863380932	1.546676902	-5.352855686	1.89314E-05	0.031260407
cg19079357	-0.533231012	3.650924001	-5.351589589	1.89907E-05	0.03128537
cg06222181	-0.516873731	3.510947703	-5.349375167	1.90949E-05	0.03138394
cg13191285	-0.766164755	1.996993246	-5.346747095	1.92192E-05	0.03139025
cg07532183	-0.680565358	-0.819746561	-5.346313945	1.92398E-05	0.03139025
cg05069950	-0.887781994	0.051269893	-5.346140921	1.9248E-05	0.03139025
cg20754324	-0.606579022	-0.073472847	-5.344573313	1.93227E-05	0.03139025
cg23348610	-0.532116418	4.126537312	-5.344433151	1.93294E-05	0.03139025
cg12599431	-0.608026572	2.834670193	-5.343696182	1.93646E-05	0.03139025
cg11657317	-0.612254136	3.508855009	-5.341248986	1.9482E-05	0.031409697
cg05308141	-0.565052756	-1.792489621	-5.340854298	1.9501E-05	0.031409697
cg25449870	-0.491960433	4.10946035	-5.340675779	1.95096E-05	0.031409697
cg23246901	-1.158727326	-4.129625512	-5.337997605	1.96391E-05	0.031431836
cg12248872	-0.739273034	2.967120496	-5.337647977	1.96561E-05	0.031431836
cg01071052	-0.736129139	3.364931633	-5.337253794	1.96752E-05	0.031431836
cg13055487	-0.784613684	2.064205369	-5.334923905	1.97888E-05	0.031431836
cg23671701	-0.597404819	3.390281391	-5.333665068	1.98504E-05	0.031431836
cg10272637	-0.626851071	2.909663625	-5.333261301	1.98703E-05	0.031431836
cg08206271	-0.689790892	3.033188494	-5.332463184	1.99095E-05	0.031431836
cg18809953	-0.970652058	-3.370278193	-5.331917562	1.99363E-05	0.031431836
cg18097228	-0.712779574	3.310823429	-5.330528875	2.00049E-05	0.031431836
cg00092687	-0.867094331	2.191112445	-5.330362203	2.00131E-05	0.031431836
cg05058652	-0.611496757	3.308353219	-5.329509452	2.00553E-05	0.031431836
cg00052976	-0.578115735	3.718471807	-5.329223396	2.00695E-05	0.031431836
cg21613901	-0.754493891	2.542202655	-5.325560209	2.0252E-05	0.031431836
cg07501414	-0.567346224	3.420133681	-5.325258555	2.02671E-05	0.031431836
cg25681728	-0.519933109	0.700633992	-5.324884656	2.02859E-05	0.031431836
cg03138206	-0.629526162	2.936079908	-5.324190327	2.03207E-05	0.031431836
cg24619797	-0.812674331	2.901607092	-5.324073667	2.03266E-05	0.031431836
cg00275479	0.791832815	3.622199775	5.323594843	2.03506E-05	0.031431836
cg24446454	-0.664054227	3.288785249	-5.322536965	2.04039E-05	0.031431836
cg20412255	-0.562663196	3.811130972	-5.319716863	2.05466E-05	0.031431836
cg18083764	-0.514968854	-0.485396001	-5.319601009	2.05525E-05	0.031431836
cg00605523	-0.56318409	3.268498882	-5.319581093	2.05535E-05	0.031431836
cg12796085	-0.717834018	3.390137079	-5.316875386	2.06914E-05	0.031431836
cg10740880	-0.580455812	3.428642818	-5.316308406	2.07205E-05	0.031431836
cg08225009	-0.539784778	-1.095010991	-5.315331219	2.07706E-05	0.031431836
cg24594002	-0.98221795	1.669350692	-5.315324392	2.07709E-05	0.031431836
cg11912202	-0.603697434	-0.119204523	-5.313243461	2.08781E-05	0.031431836
cg06060681	-0.662528259	3.068899683	-5.310265466	2.10324E-05	0.031431836
cg00150774	-0.606657883	2.480128865	-5.309360379	2.10795E-05	0.031431836

cg06947461	0.559499271	1.880333544	5.309276109	2.10839E-05	0.031431836
cg04584635	-0.694115155	2.995778911	-5.308294406	2.11351E-05	0.031431836
cg11227979	-1.007586972	1.588557702	-5.308008529	2.115E-05	0.031431836
cg01124048	-0.691492175	-0.011408486	-5.307690235	2.11667E-05	0.031431836
cg25844590	-0.86068023	1.8086775	-5.307224695	2.11911E-05	0.031431836
cg03536711	-0.704645046	-0.076468857	-5.307201296	2.11923E-05	0.031431836
cg08623623	-0.800682183	1.781466406	-5.305594527	2.12767E-05	0.031431836
cg14730919	-0.543594618	0.089306994	-5.305398358	2.1287E-05	0.031431836
cg14415410	-0.608135935	3.361077229	-5.305383069	2.12878E-05	0.031431836
cg01655210	-0.69743017	2.782370099	-5.305245266	2.12951E-05	0.031431836
cg24066561	-0.73231384	3.099023301	-5.305185629	2.12982E-05	0.031431836
cg01409736	-0.792916714	1.87187422	-5.30223665	2.14541E-05	0.031534769
cg12486630	-0.609629758	3.343254618	-5.302182135	2.1457E-05	0.031534769
cg13442200	-0.610391771	3.404819822	-5.301142113	2.15122E-05	0.031550526
cg25498731	-1.327747652	0.18294011	-5.299790848	2.15843E-05	0.031562977
cg22368180	-0.656218882	3.060996484	-5.299301965	2.16104E-05	0.031562977
cg21005546	-0.944517402	2.644702659	-5.298478974	2.16544E-05	0.031562977
cg09180903	-0.710956642	3.535718721	-5.297220998	2.17219E-05	0.031596323
cg15290913	-0.617921515	3.108284155	-5.295308216	2.18249E-05	0.031622743
cg16443716	0.774126133	2.883474242	5.291420521	2.20358E-05	0.031622743
cg18456139	-0.772379869	3.067780245	-5.289854118	2.21213E-05	0.031622743
cg23649326	-0.905627489	2.286197988	-5.289275313	2.2153E-05	0.031622743
cg25622155	0.849073684	-3.644615574	5.28919685	2.21573E-05	0.031622743
cg12337525	-0.604231727	-2.063014482	-5.288776855	2.21804E-05	0.031622743
cg12738800	-0.608785266	3.465570581	-5.288163711	2.22141E-05	0.031622743
cg04513819	-0.669963418	3.235722926	-5.285178118	2.23788E-05	0.031622743
cg25146230	-0.628550751	3.223024603	-5.284346778	2.24248E-05	0.031622743
cg20715291	-0.566364496	3.387206455	-5.283928071	2.24481E-05	0.031622743
cg16408679	0.723087029	1.905770384	5.283572488	2.24678E-05	0.031622743
cg08730315	-0.444553034	0.898626526	-5.282753223	2.25134E-05	0.031622743
cg02611605	-0.758950394	2.230672068	-5.282507189	2.25271E-05	0.031622743
cg25259085	-0.802233653	2.937134499	-5.282494086	2.25279E-05	0.031622743
cg10154953	-0.750188865	2.607325381	-5.281997961	2.25555E-05	0.031622743
cg17388712	-0.671639152	0.530985379	-5.280118478	2.26607E-05	0.031622743
cg19571979	-0.563206892	-0.5906001	-5.280045143	2.26648E-05	0.031622743
cg07218995	-0.905653625	2.609475001	-5.279883065	2.26739E-05	0.031622743
cg22551243	0.558514376	2.533686586	5.279206001	2.27119E-05	0.031622743
cg12287196	-0.651054789	3.630263864	-5.278907086	2.27287E-05	0.031622743
cg04072135	-0.746445041	3.366355927	-5.278564346	2.2748E-05	0.031622743
cg08383122	-0.516545861	3.817912752	-5.278280813	2.2764E-05	0.031622743
cg00547312	-0.699749121	2.309599473	-5.278231087	2.27668E-05	0.031622743
cg17313245	-0.97483465	1.616654949	-5.275065898	2.29458E-05	0.031635765
cg01513623	-0.768316188	-0.737295833	-5.274906269	2.29549E-05	0.031635765
cg06740667	-0.781612308	2.724309952	-5.274325997	2.29879E-05	0.031635765
cg04543823	-0.608328462	3.013898621	-5.274257812	2.29918E-05	0.031635765
cg25594319	-0.447548536	-0.039527971	-5.274122652	2.29994E-05	0.031635765
cg21969993	-0.596312073	3.471449183	-5.272862102	2.30713E-05	0.031639983

cg10298896	-0.75481317	2.794015093	-5.271884712	2.31272E-05	0.031639983
cg04934763	-0.552580519	4.147259251	-5.271510791	2.31486E-05	0.031639983
cg07165256	-0.656579505	2.920322292	-5.270942901	2.31812E-05	0.031639983
cg24631800	-0.781381663	2.677911814	-5.269368047	2.32717E-05	0.031682862
cg08348615	-0.485125105	3.897386573	-5.268841975	2.3302E-05	0.031682862
cg11721178	-0.520013994	3.336628071	-5.267734094	2.3366E-05	0.031709009
cg12782294	-0.941984001	0.704361963	-5.265632125	2.34879E-05	0.031751888
cg06566419	-0.775464873	2.785266576	-5.264875198	2.3532E-05	0.031751888
cg05709091	-0.595419595	3.566338856	-5.264873326	2.35321E-05	0.031751888
cg17195693	-0.714587937	2.834003099	-5.263646143	2.36037E-05	0.031787953
cg04100107	0.665699098	-3.988302743	5.261430939	2.37335E-05	0.031836085
cg07199536	-0.993073457	1.204991178	-5.259783716	2.38305E-05	0.031836085
cg16372630	-0.642312868	2.794520398	-5.257560975	2.3962E-05	0.031836085
cg24264042	-0.537891639	2.572036286	-5.256124588	2.40474E-05	0.031836085
cg18700744	-0.547862144	-0.2761864	-5.255508251	2.40841E-05	0.031836085
cg05439262	-0.997847153	2.542739182	-5.255172261	2.41042E-05	0.031836085
cg05128437	-0.514149486	0.481342103	-5.255084792	2.41094E-05	0.031836085
cg12445374	0.499593158	3.523370268	5.254725637	2.41309E-05	0.031836085
cg09940082	-0.600254438	2.811212671	-5.253758238	2.41887E-05	0.031836085
cg22336874	-0.667911675	3.588229135	-5.253465679	2.42063E-05	0.031836085
cg10317175	-0.821896946	-1.868468004	-5.252933685	2.42382E-05	0.031836085
cg05202700	-0.543319776	2.428724177	-5.25264348	2.42556E-05	0.031836085
cg19233790	-0.748584654	2.659671365	-5.252556259	2.42608E-05	0.031836085
cg18139075	-0.718930775	2.928161661	-5.252426647	2.42686E-05	0.031836085
cg14937228	-0.635860987	0.504489231	-5.250338776	2.43944E-05	0.031848682
cg06143047	-0.573940904	3.065868427	-5.249570897	2.44409E-05	0.031848682
cg14051606	-0.680598037	-2.587624059	-5.247371187	2.45744E-05	0.031848682
cg13537111	-0.565404834	4.044801298	-5.246378471	2.46349E-05	0.031848682
cg07099640	-0.513564434	-2.081095444	-5.245804697	2.46699E-05	0.031848682
cg16149437	-0.691351457	2.187190392	-5.245355233	2.46974E-05	0.031848682
cg03530006	-0.506720634	-0.567198691	-5.245115675	2.47121E-05	0.031848682
cg13325904	-0.537933122	-0.72386513	-5.244241148	2.47657E-05	0.031848682
cg22562234	-0.576260756	3.810415416	-5.243671224	2.48007E-05	0.031848682
cg12802773	-0.870136408	2.635055948	-5.243600826	2.4805E-05	0.031848682
cg17164401	-0.905457263	0.869222537	-5.243457248	2.48138E-05	0.031848682
cg27317493	-1.069299837	-1.322115724	-5.242369869	2.48807E-05	0.031848682
cg07099118	-0.84233171	2.74660396	-5.241526316	2.49328E-05	0.031848682
cg11085309	-0.675139416	2.833911082	-5.240313095	2.50078E-05	0.031848682
cg08331840	-0.473447662	3.537524575	-5.23990171	2.50333E-05	0.031848682
cg05504252	-0.748314629	3.173146538	-5.239787139	2.50404E-05	0.031848682
cg26429266	-0.66698181	-0.907069545	-5.237655199	2.5173E-05	0.031848682
cg10475928	0.611067263	1.172152463	5.236155258	2.52668E-05	0.031848682
cg01182171	-0.521415805	3.746536868	-5.235807685	2.52885E-05	0.031848682
cg13371883	-0.561384705	0.215626771	-5.23560879	2.5301E-05	0.031848682
cg22565309	-0.603594563	3.44521666	-5.235479282	2.53091E-05	0.031848682
cg09075968	-0.502825288	0.094720777	-5.233811735	2.54139E-05	0.031848682
cg20743720	-0.689549684	3.18988086	-5.233661823	2.54233E-05	0.031848682

cg20951949	-0.51347101	3.789580063	-5.233331214	2.54442E-05	0.031848682
cg00275886	-0.670855467	3.445529418	-5.232855768	2.54742E-05	0.031848682
cg25350011	-0.883269159	-2.500268603	-5.230639826	2.56144E-05	0.031848682
cg23034784	-0.537614256	3.811271507	-5.229288654	2.57003E-05	0.031848682
cg10363271	-0.51106035	3.664773964	-5.229277103	2.57011E-05	0.031848682
cg25599924	-0.794834961	-1.298531447	-5.228683203	2.57389E-05	0.031848682
cg01071385	-0.676102683	3.904751906	-5.228005251	2.57822E-05	0.031848682
cg19259203	-0.726880697	0.505312639	-5.227819081	2.57941E-05	0.031848682
cg16382030	-0.596007031	-0.439871498	-5.227559136	2.58107E-05	0.031848682
cg04499254	-0.584615037	-0.059768015	-5.227250914	2.58305E-05	0.031848682
cg14514716	-0.435366166	-0.581659869	-5.226986617	2.58474E-05	0.031848682
cg26354531	-0.44782088	4.041104366	-5.226546909	2.58756E-05	0.031848682
cg08026772	-0.812613897	2.329580635	-5.225935493	2.59148E-05	0.031848682
cg08368148	0.707412275	-4.175295478	5.22506373	2.59708E-05	0.031848682
cg21893193	-0.795075313	2.934583617	-5.224454813	2.60101E-05	0.031848682
cg20899034	-0.686863458	2.975379282	-5.223883361	2.60469E-05	0.031848682
cg14153481	-0.491276197	-0.782960948	-5.220297815	2.62794E-05	0.031848682
cg10947408	-0.550947064	-2.346813444	-5.217506215	2.64619E-05	0.031848682
cg22427411	-0.969394845	1.598905767	-5.216919932	2.65004E-05	0.031848682
cg26255194	-0.541796025	3.320293862	-5.216097975	2.65545E-05	0.031848682
cg11467262	-0.602588659	-2.622949124	-5.215580732	2.65885E-05	0.031848682
cg14998926	-0.666233056	3.332457351	-5.215045579	2.66238E-05	0.031848682
cg21241222	-0.575260741	3.201911305	-5.214344329	2.66701E-05	0.031848682
cg22337220	-0.628249778	3.618197181	-5.213428443	2.67308E-05	0.031848682
cg16914533	-1.007417756	2.355116472	-5.21241534	2.6798E-05	0.031848682
cg24901282	-0.593335587	0.695638443	-5.212172214	2.68141E-05	0.031848682
cg09853301	-0.548443641	3.272347116	-5.211079224	2.68869E-05	0.031848682
cg02004203	-0.890419529	1.890686531	-5.210826003	2.69038E-05	0.031848682
cg22666612	-0.678720937	3.207683181	-5.210389507	2.69329E-05	0.031848682
cg09138965	-0.672035956	3.837247303	-5.210325842	2.69372E-05	0.031848682
cg20697575	-0.498347525	2.852761201	-5.210172526	2.69474E-05	0.031848682
cg00857512	-0.604104146	-1.644397324	-5.2093707	2.7001E-05	0.031848682
cg24345261	-0.729222594	0.258983606	-5.206813225	2.71728E-05	0.031848682
cg03797628	-0.514833421	3.78029942	-5.206716337	2.71793E-05	0.031848682
cg13046608	-0.541149961	-0.176635652	-5.206198157	2.72143E-05	0.031848682
cg09837799	-0.646390303	3.686026795	-5.206131774	2.72188E-05	0.031848682
cg02286270	-0.581934269	0.949431986	-5.205048103	2.7292E-05	0.031848682
cg25768895	-0.72798476	3.349780708	-5.204926796	2.73002E-05	0.031848682
cg08403513	-0.687189834	4.018537947	-5.204892682	2.73025E-05	0.031848682
cg20833599	-0.569908912	3.512359935	-5.204560368	2.7325E-05	0.031848682
cg07068709	-0.570092019	0.583405017	-5.204525319	2.73274E-05	0.031848682
cg24770798	-0.561920102	-0.832180746	-5.20450195	2.7329E-05	0.031848682
cg04089974	-0.968032559	2.264182322	-5.204194175	2.73498E-05	0.031848682
cg10634424	-0.866177096	2.475031853	-5.204005358	2.73627E-05	0.031848682
cg26483805	-0.539348638	3.793367447	-5.200434425	2.7606E-05	0.031848682
cg13860928	-0.614042772	-0.621196305	-5.200185639	2.76231E-05	0.031848682
cg19160686	-0.682804597	3.014635026	-5.199824036	2.76479E-05	0.031848682

cg01501737	-0.662027236	3.097906945	-5.199092995	2.7698E-05	0.031848682
cg19556572	-0.58790857	3.458822009	-5.198374126	2.77475E-05	0.031848682
cg15272286	-0.630256019	3.593193262	-5.197583185	2.78019E-05	0.031848682
cg26692791	-0.723170149	-0.234022021	-5.197493537	2.78081E-05	0.031848682
cg22731763	-0.544746353	0.526055364	-5.197383932	2.78157E-05	0.031848682
cg13627968	-0.626002647	-1.225063818	-5.197206359	2.78279E-05	0.031848682
cg09648366	-0.49419079	0.570222134	-5.196857974	2.7852E-05	0.031848682
cg19295399	-0.55293339	3.739630007	-5.196522016	2.78752E-05	0.031848682
cg06846903	-0.650623635	3.760230541	-5.196050101	2.79079E-05	0.031848682
cg01068906	-0.509064498	-0.969279641	-5.19574517	2.7929E-05	0.031848682
cg07595547	-0.670388605	2.509090219	-5.195728164	2.79301E-05	0.031848682
cg13392874	-0.593995932	3.615259589	-5.1936719	2.8073E-05	0.031848682
cg03519805	-0.670749015	0.052574548	-5.193475355	2.80866E-05	0.031848682
cg00567492	-1.026404966	-2.210523203	-5.19335014	2.80954E-05	0.031848682
cg06133910	-0.556606418	-1.373483723	-5.193286489	2.80998E-05	0.031848682
cg19628390	-0.514225584	3.836749858	-5.192382048	2.81629E-05	0.031869221
cg00264291	-0.463121796	4.466128939	-5.191723608	2.8209E-05	0.031870403
cg02406289	-0.537879376	3.29440603	-5.190234485	2.83133E-05	0.031930949
cg19588259	-0.536276506	4.306644658	-5.189674626	2.83527E-05	0.031930949
cg12264517	-0.759514954	-2.155215622	-5.188419048	2.84411E-05	0.031979717
cg01944899	-0.662769137	-0.716056087	-5.187065358	2.85368E-05	0.031988313
cg17736819	-0.645782972	3.510706272	-5.186898527	2.85486E-05	0.031988313
cg08643930	0.553498818	2.045690432	5.185922146	2.86179E-05	0.031988313
cg21752295	-0.463140158	4.506336383	-5.185759484	2.86294E-05	0.031988313
cg01223833	-0.617842595	1.865830718	-5.183389719	2.87982E-05	0.032069162
cg05077566	-0.657125664	2.754873678	-5.183196525	2.8812E-05	0.032069162
cg26293014	-0.810744044	1.145556887	-5.182439134	2.88662E-05	0.032069162
cg18276368	-0.642729401	2.98896813	-5.181556595	2.89295E-05	0.032069162
cg12276348	-0.665295489	-0.506536161	-5.181486827	2.89345E-05	0.032069162
cg18209323	-0.777655332	-2.285569675	-5.179975451	2.90432E-05	0.032069162
cg26891537	-0.921897097	3.33590262	-5.179916834	2.90475E-05	0.032069162
cg02835779	-0.598928687	4.118278295	-5.17968813	2.90639E-05	0.032069162
cg03827311	-0.575711392	1.614953262	-5.176074826	2.93257E-05	0.032135701
cg10791778	-0.72665298	1.196648892	-5.175669588	2.93552E-05	0.032135701
cg23147243	-0.623217578	-0.831471119	-5.175109908	2.9396E-05	0.032135701
cg03859846	-0.662367238	0.583821644	-5.174809431	2.94179E-05	0.032135701
cg01795204	-0.87950692	1.8190199	-5.174610004	2.94325E-05	0.032135701
cg17609282	-0.459595154	3.820580213	-5.174439216	2.9445E-05	0.032135701
cg01285877	-0.586844666	0.78669963	-5.173467102	2.95161E-05	0.032135701
cg02005249	-0.617161603	3.77682709	-5.17255931	2.95827E-05	0.032135701
cg08388519	-0.738921186	2.865959523	-5.172009618	2.9623E-05	0.032135701
cg02674039	-0.53176482	1.034431884	-5.17170656	2.96453E-05	0.032135701
cg27629134	-0.71404235	3.066669897	-5.170556686	2.97301E-05	0.032135701
cg26207372	-0.630291455	2.91312974	-5.169707042	2.97928E-05	0.032135701
cg09524422	-0.567464852	0.101095302	-5.168993403	2.98456E-05	0.032135701
cg14279345	-0.487740602	3.577777845	-5.16764593	2.99456E-05	0.032135701
cg17078152	-0.528836712	3.754636313	-5.167286351	2.99724E-05	0.032135701

cg16096176	-0.646224234	-0.157282109	-5.164471885	3.01825E-05	0.032135701
cg17822735	-0.655910584	0.181573751	-5.163505679	3.02549E-05	0.032135701
cg20262306	-0.568699963	1.399770213	-5.163027303	3.02909E-05	0.032135701
cg24634408	-0.744305558	3.48461322	-5.161841402	3.03802E-05	0.032135701
cg03819767	-0.929939651	2.48934439	-5.161769459	3.03856E-05	0.032135701
cg15852986	-0.418688215	4.250497657	-5.161289631	3.04218E-05	0.032135701
cg04004056	-0.475975108	3.641231293	-5.161222459	3.04269E-05	0.032135701
cg04357925	-0.569566292	-1.105514905	-5.160560724	3.04769E-05	0.032135701
cg02011981	-0.577577183	0.352178257	-5.159902923	3.05267E-05	0.032135701
cg03882100	-0.660370571	3.128490577	-5.159075158	3.05895E-05	0.032135701
cg06844213	4.699591059	-1.446867578	5.159031928	3.05928E-05	0.032135701
cg05886720	-0.552500322	0.090131063	-5.158813758	3.06094E-05	0.032135701
cg17833539	-0.715256964	2.477527971	-5.158226212	3.06541E-05	0.032135701
cg17131410	0.843141049	1.500270874	5.157564278	3.07045E-05	0.032135701
cg11165254	-0.878652035	2.194059085	-5.156667242	3.0773E-05	0.032135701
cg25463742	-0.695631853	1.959174414	-5.156629918	3.07758E-05	0.032135701
cg06800565	0.558462944	-4.042440674	5.155485595	3.08634E-05	0.032135701
cg20210151	-0.585985616	3.70920575	-5.154958353	3.09038E-05	0.032135701
cg06986754	-0.678141869	1.15098078	-5.153901	3.0985E-05	0.032135701
cg06036449	-0.72969858	2.354518591	-5.153125287	3.10448E-05	0.032135701
cg08063891	-0.538370114	3.289202513	-5.152340229	3.11053E-05	0.032135701
cg03672752	-0.678544037	5.079957541	-5.150930096	3.12144E-05	0.032135701
cg23293720	-0.541055002	3.274602677	-5.150789846	3.12253E-05	0.032135701
cg04339177	-0.52714748	-0.703445959	-5.150747159	3.12286E-05	0.032135701
cg23176214	-0.53144884	1.063400074	-5.150606728	3.12395E-05	0.032135701
cg21238327	-0.536261304	-1.140327021	-5.150314269	3.12622E-05	0.032135701
cg08877049	-0.59368893	3.68160167	-5.149685254	3.13111E-05	0.032135701
cg01339351	-0.470936551	3.450479887	-5.149266715	3.13436E-05	0.032135701
cg17238970	-0.782127604	3.047163395	-5.148010432	3.14416E-05	0.032135701
cg18559561	-0.920698921	1.819309305	-5.147076124	3.15146E-05	0.032135701
cg11681913	-0.660743558	3.076535901	-5.146472311	3.15619E-05	0.032135701
cg01607143	-0.748103862	2.806117412	-5.145941862	3.16035E-05	0.032135701
cg24295122	-0.706487027	3.39979392	-5.145170109	3.16641E-05	0.032135701
cg13755396	-0.596861368	2.041337681	-5.144296049	3.17329E-05	0.032135701
cg26916006	-0.598153268	2.858700386	-5.143899059	3.17642E-05	0.032135701
cg22613078	-0.628134003	3.083442181	-5.143592826	3.17884E-05	0.032135701
cg21177832	-0.483062566	0.049855878	-5.142560026	3.187E-05	0.032135701
cg19615684	-0.599815982	-1.082123995	-5.142454727	3.18784E-05	0.032135701
cg11284625	-0.644993821	3.067766593	-5.142395528	3.18831E-05	0.032135701
cg12595444	-0.709851107	-0.038082845	-5.141293959	3.19704E-05	0.032135701
cg02378329	-0.541934066	0.929496162	-5.140804867	3.20093E-05	0.032135701
cg16562126	-0.753502577	-0.021275167	-5.140665674	3.20203E-05	0.032135701
cg23410343	-0.574126768	4.23618623	-5.139825809	3.20872E-05	0.032135701
cg27260984	-0.477063863	3.923249925	-5.138703378	3.21768E-05	0.032135701
cg26231350	-0.464118218	3.665589696	-5.138258043	3.22124E-05	0.032135701
cg27227159	-0.645902933	-0.828488877	-5.137765501	3.22518E-05	0.032135701
cg02656323	-0.590742404	0.232119746	-5.137103662	3.23049E-05	0.032135701

cg02236094	-0.815964469	3.002744104	-5.137015316	3.2312E-05	0.032135701
cg11621632	-0.715483854	-1.749462974	-5.136556781	3.23488E-05	0.032135701
cg19939732	-0.706432863	3.323807648	-5.13640732	3.23608E-05	0.032135701
cg15656824	-0.442523614	4.075762458	-5.135996032	3.23939E-05	0.032135701
cg15993408	-0.468938703	3.862343391	-5.135575497	3.24278E-05	0.032135701
cg27434995	-0.741173768	0.763865897	-5.134071559	3.25491E-05	0.032135701
cg09031957	-0.710158148	2.60400471	-5.132770693	3.26545E-05	0.032135701
cg02774854	-0.528834824	4.055395464	-5.132205083	3.27004E-05	0.032135701
cg26877270	-0.755724953	1.646754002	-5.131466258	3.27605E-05	0.032135701
cg02718752	-0.735814263	-0.452195305	-5.131343232	3.27705E-05	0.032135701
cg16385474	-0.497555707	3.497434206	-5.130335331	3.28526E-05	0.032135701
cg11204897	-0.811071141	2.633157872	-5.129807749	3.28957E-05	0.032135701
cg24158307	-0.561243371	0.490706549	-5.129807142	3.28958E-05	0.032135701
cg25674438	-0.649253994	3.060725233	-5.129415366	3.29278E-05	0.032135701
cg06899522	-0.669671253	-0.127577408	-5.128948337	3.2966E-05	0.032135701
cg06800806	-0.659271215	3.375804306	-5.128494509	3.30032E-05	0.032135701
cg20931223	-0.453647181	3.366941769	-5.128045156	3.30401E-05	0.032135701
cg20600515	-0.78344806	2.951897642	-5.12803916	3.30406E-05	0.032135701
cg02258002	-0.591216478	2.797020695	-5.127606385	3.30761E-05	0.032135701
cg04398218	-0.580917651	3.336204679	-5.127590029	3.30775E-05	0.032135701
cg21988302	-0.556234061	4.234841788	-5.127303834	3.3101E-05	0.032135701
cg13053595	-0.742839278	3.281230514	-5.125217997	3.3273E-05	0.032135701
cg09054474	-0.523873731	3.438465938	-5.124710757	3.3315E-05	0.032135701
ch.10.1020819F	-0.848550162	-1.719600143	-5.124611384	3.33232E-05	0.032135701
cg22726219	-0.624220705	-1.003567106	-5.124577217	3.3326E-05	0.032135701
cg14162011	-0.598353197	3.402923071	-5.124358654	3.33441E-05	0.032135701
cg22216302	-0.710749325	0.55670275	-5.124022829	3.33719E-05	0.032135701
cg06537680	-0.581751568	-2.302082661	-5.123847151	3.33865E-05	0.032135701
cg09035925	-0.700050337	-2.04997649	-5.123132632	3.34458E-05	0.032135701
cg17126523	-0.516207494	3.767440904	-5.123127566	3.34463E-05	0.032135701
cg16161447	-0.58567255	4.062565695	-5.12285074	3.34693E-05	0.032135701
cg25072090	-0.501573448	4.255473121	-5.121561088	3.35767E-05	0.032135701
cg13411923	-0.746792442	0.317334893	-5.121382547	3.35916E-05	0.032135701
cg03405302	-0.753670982	1.996830387	-5.120872057	3.36342E-05	0.032135701
cg03654707	-0.632008769	-0.829051849	-5.11945494	3.37529E-05	0.032135701
cg06437030	-0.733096863	1.447952004	-5.119264253	3.37689E-05	0.032135701
cg01093898	-0.484859122	3.302522395	-5.117352743	3.39297E-05	0.032135701
cg13947385	-0.664146391	3.509103761	-5.11680308	3.39761E-05	0.032135701
cg14660710	-0.744333521	0.735782603	-5.114827361	3.41433E-05	0.032135701
cg09947611	-0.458275562	-1.291240513	-5.11398609	3.42147E-05	0.032135701
cg01338157	-0.597889817	3.536191067	-5.113331694	3.42704E-05	0.032135701
cg19294383	-0.741435923	3.23766136	-5.113241493	3.42781E-05	0.032135701
cg03624563	-0.685028787	2.890189272	-5.113058709	3.42937E-05	0.032135701
cg14665781	-0.609102579	2.809226268	-5.112546743	3.43374E-05	0.032135701
cg17280723	-0.56670732	3.337466914	-5.112178975	3.43688E-05	0.032135701
cg04683300	-0.885421177	2.548036143	-5.111965232	3.4387E-05	0.032135701
cg20751398	-0.478336508	-0.148492021	-5.109740967	3.45777E-05	0.032135701

cg25826143	-0.801773905	-2.18373387	-5.109243275	3.46205E-05	0.032135701
cg20511962	-0.683083518	2.58182704	-5.108474583	3.46867E-05	0.032135701
cg00626624	-0.881936394	-0.581935828	-5.107572089	3.47646E-05	0.032135701
cg02095572	-0.666465902	3.540379007	-5.106352546	3.48701E-05	0.032135701
cg06794293	-0.575841521	-1.639014669	-5.105542496	3.49404E-05	0.032135701
cg17476223	-0.830253133	2.95062983	-5.105490258	3.49449E-05	0.032135701
cg06561001	-0.454253234	3.669799225	-5.105417291	3.49513E-05	0.032135701
cg12965833	-0.817017072	1.348181667	-5.105038614	3.49842E-05	0.032135701
cg05747234	-0.611785326	2.770882846	-5.10495191	3.49917E-05	0.032135701
cg27401755	-0.803119274	2.410279808	-5.104571307	3.50248E-05	0.032135701
cg19363920	-0.622037016	3.679280619	-5.10409119	3.50667E-05	0.032135701
cg12691765	-0.587943992	3.779161505	-5.103563474	3.51127E-05	0.032135701
cg13665823	-0.47088155	3.419524271	-5.103556316	3.51133E-05	0.032135701
cg26502057	-0.900712318	2.41093025	-5.103127331	3.51508E-05	0.032135701
cg24784036	-0.558153262	-0.827490553	-5.1031269	3.51508E-05	0.032135701
cg18791395	-0.564598236	3.266670094	-5.102880867	3.51723E-05	0.032135701
cg12041684	-0.726676583	2.889190201	-5.100656765	3.53673E-05	0.032135701
cg10190744	-0.534752909	4.103508215	-5.100472334	3.53836E-05	0.032135701
cg26123532	-0.667966412	0.43583242	-5.099711082	3.54506E-05	0.032135701
cg01549663	-0.690361185	-1.684418049	-5.098514367	3.55562E-05	0.032135701
cg03440426	-0.669469001	2.167577031	-5.097368897	3.56576E-05	0.032135701
cg00534472	-0.73995001	2.963512563	-5.097111739	3.56804E-05	0.032135701
cg18721807	-0.704760394	2.885444709	-5.09708731	3.56826E-05	0.032135701
cg17540765	-0.458304884	-0.332597903	-5.096419401	3.57419E-05	0.032135701
cg25046661	-0.925549188	2.851026726	-5.096385577	3.57449E-05	0.032135701
cg15260981	-0.856110689	1.942202456	-5.096199274	3.57615E-05	0.032135701
cg14930335	-0.62148732	-0.611737107	-5.095971258	3.57818E-05	0.032135701
cg00320861	-0.840176652	1.781477953	-5.095218125	3.58488E-05	0.032135701
cg23105907	-0.591886881	0.900374054	-5.094673662	3.58974E-05	0.032135701
cg23323457	0.783176581	-4.948786404	5.094401278	3.59217E-05	0.032135701
cg22506548	-0.407403025	-0.246162773	-5.092243889	3.61149E-05	0.032135701
cg27327253	-0.666359422	1.741739142	-5.091484971	3.61831E-05	0.032135701
cg20758171	-0.68646692	-1.137491534	-5.091268803	3.62026E-05	0.032135701
cg02687109	-0.722923766	3.276067929	-5.090330637	3.62871E-05	0.032135701
cg16142112	-0.767303088	0.277125999	-5.089492445	3.63629E-05	0.032135701
cg03335128	-0.661029798	1.477815112	-5.089391596	3.6372E-05	0.032135701
cg20237960	-0.660607156	3.049945211	-5.089301297	3.63801E-05	0.032135701
cg11717782	-0.561860491	3.269707833	-5.088737177	3.64312E-05	0.032135701
cg09835686	-0.684059678	3.634421676	-5.087275346	3.65639E-05	0.032135701
cg17573714	-0.561885405	3.739542922	-5.086745986	3.66121E-05	0.032135701
cg02843359	-0.735437311	2.793947931	-5.086078198	3.66729E-05	0.032135701
cg23889476	-0.925076599	2.666798373	-5.085777727	3.67003E-05	0.032135701
cg07428713	-0.60307939	3.652134666	-5.085378375	3.67368E-05	0.032135701
cg13388822	-0.651779673	3.690939578	-5.085253967	3.67482E-05	0.032135701
cg18548400	-0.553770595	1.224714598	-5.085076166	3.67644E-05	0.032135701
cg17857235	0.679933393	2.36573496	5.084868271	3.67834E-05	0.032135701
cg12711407	-0.601483301	3.224982595	-5.084046565	3.68587E-05	0.032135701

cg11066131	-0.733804992	3.156307949	-5.083669653	3.68932E-05	0.032135701
cg09671092	-0.550153157	0.323756055	-5.082745201	3.69782E-05	0.032135701
cg21553916	-0.786780021	2.681331537	-5.082578984	3.69934E-05	0.032135701
cg20452252	-0.546134822	3.590127698	-5.082468763	3.70036E-05	0.032135701
cg22240998	-0.654256255	2.708326809	-5.082438374	3.70064E-05	0.032135701
cg04011253	-0.621296166	-3.851101744	-5.081917562	3.70544E-05	0.032135701
cg17173891	-0.574299745	3.873420172	-5.081907532	3.70553E-05	0.032135701
cg23352483	-0.573459158	-1.353840013	-5.081681404	3.70761E-05	0.032135701
cg03094728	-0.741069855	2.275042859	-5.081615401	3.70822E-05	0.032135701
cg16440564	-0.535103875	3.592942623	-5.081221313	3.71186E-05	0.032135701
cg18207109	-0.656168109	3.413407899	-5.08069294	3.71674E-05	0.032135701
cg08517799	-0.746612062	2.389296865	-5.080505546	3.71847E-05	0.032135701
cg16255198	-0.60990115	3.799659171	-5.080100815	3.72222E-05	0.032135701
cg13126387	-0.635271008	3.216951304	-5.079370624	3.72898E-05	0.032135701
cg14793544	-0.82622985	-1.833823105	-5.079105594	3.73144E-05	0.032135701
cg09437691	-0.577312122	3.435981768	-5.07841071	3.7379E-05	0.032135701
cg01145653	-0.75754307	1.639873994	-5.078154159	3.74028E-05	0.032135701
cg09690103	-0.662157636	-0.122442411	-5.077369373	3.74759E-05	0.032135701
cg24318365	-0.498772776	4.62800364	-5.077243727	3.74876E-05	0.032135701
cg13803067	-0.558886949	2.585057431	-5.077205273	3.74912E-05	0.032135701
cg10807105	-0.694013175	-0.111947501	-5.077066035	3.75042E-05	0.032135701
cg19961312	-0.530373689	3.886928358	-5.076501597	3.75569E-05	0.032135701
cg11041636	-0.614518661	3.157219186	-5.075622186	3.76391E-05	0.032135701
cg22616124	-1.024766638	2.321049579	-5.075541103	3.76467E-05	0.032135701
cg26842332	-0.724006271	2.87117386	-5.075298785	3.76694E-05	0.032135701
cg07754381	-0.536407741	3.686361913	-5.074646475	3.77306E-05	0.032135701
cg09008589	-0.528694654	0.362769222	-5.073933242	3.77976E-05	0.032135701
cg01522161	-0.700081329	2.939788894	-5.073695067	3.782E-05	0.032135701
cg00513032	-0.685126912	1.273352376	-5.073384071	3.78493E-05	0.032135701
cg22155218	-0.623688017	1.490400638	-5.073374849	3.78501E-05	0.032135701
cg18107144	-0.729132788	-2.296611391	-5.073180797	3.78684E-05	0.032135701
cg18712792	-0.566601898	-1.200938117	-5.072093728	3.7971E-05	0.032135701
cg19624955	-0.692994011	2.491922104	-5.071735321	3.80048E-05	0.032135701
cg10612751	-0.609236753	3.521543972	-5.07172397	3.80059E-05	0.032135701
cg09879989	-0.485433655	3.70115089	-5.071401845	3.80364E-05	0.032135701
cg17120366	-0.713491805	2.059369638	-5.070767566	3.80964E-05	0.032135701
cg16316720	-0.518532688	3.76127282	-5.070376846	3.81335E-05	0.032135701
cg25023260	-0.721584745	3.292208421	-5.070126276	3.81573E-05	0.032135701
cg14802771	-0.56400969	-0.623273553	-5.069639574	3.82035E-05	0.032135701
cg19031044	-0.662824517	3.488801533	-5.069516855	3.82152E-05	0.032135701
cg13394713	-0.531828611	3.650036326	-5.069005718	3.82638E-05	0.032135701
cg21628673	-0.691801763	2.883180877	-5.068906837	3.82732E-05	0.032135701
cg23668476	-0.557738037	0.280520691	-5.068370886	3.83243E-05	0.032135701
cg13198116	-1.400345433	2.025220983	-5.068020586	3.83577E-05	0.032135701
cg11974480	-1.216701162	1.508372735	-5.067989932	3.83606E-05	0.032135701
cg08637691	-0.623721163	-1.217058653	-5.0671394	3.84419E-05	0.032135701
cg25705734	-0.786559075	2.309974698	-5.065927683	3.85579E-05	0.032135701

cg25752539	-0.621034062	4.042483549	-5.06585529	3.85649E-05	0.032135701
cg16040911	-0.570542806	3.594814894	-5.065620292	3.85874E-05	0.032135701
cg07047256	-1.074835915	-0.326083368	-5.065434322	3.86053E-05	0.032135701
cg12650480	-0.533620149	3.483289996	-5.064418716	3.8703E-05	0.032135701
cg11904435	-0.551414004	0.08002931	-5.06434291	3.87103E-05	0.032135701
cg14276286	-0.693132052	0.054287603	-5.063786515	3.87639E-05	0.032135701
cg04171909	-0.829053142	2.246843161	-5.062102137	3.89267E-05	0.032135701
cg00036976	-0.494582324	-1.024178976	-5.061775508	3.89583E-05	0.032135701
cg00609661	-0.626015506	3.106476424	-5.060567704	3.90756E-05	0.032135701
cg07695047	-0.527382829	-2.278481851	-5.060246819	3.91068E-05	0.032135701
cg25087601	-0.575044862	3.490273786	-5.059913453	3.91393E-05	0.032135701
cg19928955	-0.926154942	-1.473380438	-5.059168646	3.92119E-05	0.032135701
cg26398793	-0.589311905	3.277992403	-5.058955815	3.92326E-05	0.032135701
cg20536617	-0.604835936	0.768898471	-5.058732215	3.92545E-05	0.032135701
cg17781772	0.491253847	1.794480084	5.058713796	3.92563E-05	0.032135701
cg10576646	-0.590279676	3.815752961	-5.058101024	3.93162E-05	0.032135701
cg10769985	-0.482887105	4.07391458	-5.057603404	3.93649E-05	0.032135701
cg00955895	-0.645503533	3.624613781	-5.057242221	3.94003E-05	0.032135701
cg04252710	-0.828444914	2.363118773	-5.056625821	3.94608E-05	0.032135701
cg25279395	-0.652268071	3.342832528	-5.055866114	3.95355E-05	0.032135701
cg18871289	-0.561845961	-1.957694018	-5.055246143	3.95965E-05	0.032135701
cg12697764	-0.567890188	3.618043968	-5.054964246	3.96243E-05	0.032135701
cg08850719	-0.887616221	2.196067505	-5.054574895	3.96627E-05	0.032135701
cg12170574	-0.750197822	3.255640719	-5.053530648	3.97659E-05	0.032135701
cg25654380	-0.831600718	3.013200331	-5.053085518	3.981E-05	0.032135701
cg06956648	-0.541358024	2.780669816	-5.053075784	3.98109E-05	0.032135701
cg23077682	-0.676974162	3.06982779	-5.051566654	3.99608E-05	0.032135701
cg05184395	-0.524553735	0.341220044	-5.051526915	3.99647E-05	0.032135701
cg11661375	-0.664065206	2.659383546	-5.051009649	4.00162E-05	0.032135701
cg11641097	-0.841936742	2.232284372	-5.050888203	4.00283E-05	0.032135701
cg00879872	-0.56093396	3.600938552	-5.049576557	4.01592E-05	0.032135701
cg01035598	-0.647675213	2.941340986	-5.04898687	4.02182E-05	0.032135701
cg21432711	-0.52173665	4.303103906	-5.048979659	4.02189E-05	0.032135701
cg13647309	-0.550926481	4.105480968	-5.048867002	4.02302E-05	0.032135701
cg05306831	-0.603357598	3.108917142	-5.048748383	4.0242E-05	0.032135701
cg11823449	-0.739617645	2.72104253	-5.048670325	4.02499E-05	0.032135701
cg06838703	-0.757516301	2.490477481	-5.0471044	4.0407E-05	0.032135701
cg17626965	-0.905566004	2.775053476	-5.046019015	4.05163E-05	0.032135701
cg20920951	-0.585247906	3.680606909	-5.04537826	4.0581E-05	0.032135701
cg06512476	-0.598245743	3.268483316	-5.045212287	4.05978E-05	0.032135701
cg02272278	-0.509819424	0.595072164	-5.044588308	4.06609E-05	0.032135701
cg24431362	-0.920943228	2.299010515	-5.044520132	4.06678E-05	0.032135701
cg02693498	-0.70855988	-0.119972564	-5.044123781	4.07079E-05	0.032135701
cg02366018	-0.623428345	2.513357148	-5.043717831	4.07491E-05	0.032135701
cg16023600	-0.542846357	3.621902021	-5.043637398	4.07573E-05	0.032135701
cg23528787	-0.633460001	3.08485164	-5.043276285	4.07939E-05	0.032135701
cg01954957	-0.556544663	3.382008168	-5.041998297	4.09239E-05	0.032135701

cg10021494	-0.578941484	3.549553618	-5.041874342	4.09365E-05	0.032135701
cg21524530	-0.834055209	2.695539282	-5.040668253	4.10596E-05	0.032135701
cg05248081	-0.492599746	4.134345622	-5.040605072	4.10661E-05	0.032135701
cg20221707	-0.440083718	-0.17223857	-5.039654841	4.11633E-05	0.032135701
cg18553213	-0.761094609	0.265868699	-5.039625617	4.11663E-05	0.032135701
cg25768944	-0.65323051	3.518303942	-5.038488083	4.12831E-05	0.032135701
cg07685390	-0.751922018	4.372419874	-5.03773441	4.13606E-05	0.032135701
cg16793757	-0.598978544	2.369532118	-5.03734586	4.14006E-05	0.032135701
cg08988698	-0.688398097	2.486553185	-5.036849878	4.14518E-05	0.032135701
cg19027726	-0.532800102	3.438740679	-5.036654911	4.14719E-05	0.032135701
cg27530628	-0.692148702	0.644919326	-5.036519077	4.14859E-05	0.032135701
cg17723122	-0.7110426	0.320326919	-5.036194076	4.15195E-05	0.032135701
cg13760881	-0.920237935	2.771305443	-5.035361465	4.16057E-05	0.032135701
cg05423727	-0.789847975	-2.703100294	-5.034614167	4.16831E-05	0.032135701
cg19100992	-0.701708461	3.118394701	-5.033815664	4.17661E-05	0.032135701
cg02528150	-0.568139134	3.163576843	-5.032483725	4.19048E-05	0.032135701
cg15772133	-0.633356189	2.523469832	-5.031835857	4.19725E-05	0.032135701
cg08169794	-0.681551449	1.190870229	-5.031077244	4.20518E-05	0.032135701
cg26638716	0.819242784	-0.550914849	5.029982337	4.21666E-05	0.032135701
cg15000031	-0.884692983	1.0192135	-5.029813574	4.21844E-05	0.032135701
cg01887322	-0.569106207	0.180535294	-5.029045414	4.22651E-05	0.032135701
cg12327749	-0.724138575	3.023792139	-5.028900069	4.22804E-05	0.032135701
cg21220614	-0.460617325	0.158235391	-5.028691963	4.23023E-05	0.032135701
cg16598679	0.710786962	1.82854956	5.027965569	4.23789E-05	0.032135701
cg10320027	-0.714682899	2.869316876	-5.027825492	4.23937E-05	0.032135701
cg10807894	-1.086861498	-4.647810546	-5.027726973	4.24041E-05	0.032135701
cg05477401	-0.624756839	-0.725121465	-5.027586777	4.24189E-05	0.032135701
cg05760937	-0.498487947	-0.365587453	-5.027365549	4.24423E-05	0.032135701
cg25463688	-0.523394307	0.89809259	-5.027342934	4.24447E-05	0.032135701
cg26994192	-0.696544739	3.067688626	-5.027244349	4.24551E-05	0.032135701
cg24439121	-0.512022693	3.65467031	-5.0270075	4.24801E-05	0.032135701
cg11280087	-0.533467948	3.534322864	-5.026307323	4.25543E-05	0.032135701
cg23879481	-0.523629035	3.723781874	-5.026298805	4.25552E-05	0.032135701
cg09999560	-0.585004627	-0.448583797	-5.026097252	4.25765E-05	0.032135701
cg24478733	-0.48496092	3.824834598	-5.025571752	4.26323E-05	0.032135701
cg09395955	-0.745226568	-0.255052791	-5.025473824	4.26427E-05	0.032135701
cg15781504	-0.671562593	3.393701261	-5.025464455	4.26437E-05	0.032135701
cg13108609	-0.568242523	2.896606098	-5.025282649	4.2663E-05	0.032135701
cg00068519	-0.520422278	3.38063125	-5.024898716	4.27038E-05	0.032135701
cg13023030	-0.667758655	2.409115654	-5.024407909	4.2756E-05	0.032135701
cg03859668	-0.594013614	3.432311463	-5.023544879	4.2848E-05	0.032135701
cg04796642	-0.520302712	4.169690593	-5.023402372	4.28632E-05	0.032135701
cg15672365	-0.964411129	2.554594678	-5.023160794	4.2889E-05	0.032135701
cg17218344	-0.439012802	3.939482257	-5.022963488	4.29101E-05	0.032135701
cg13735193	-0.608584633	2.927099223	-5.022819873	4.29254E-05	0.032135701
cg06897370	-0.583311663	3.1143053	-5.022206997	4.2991E-05	0.032135701
cg09941995	-0.444255061	0.722401947	-5.021737479	4.30413E-05	0.032135701

cg14102577	-0.622354163	1.940279098	-5.02085543	4.3136E-05	0.032135701
cg02727828	-0.56896376	4.123346918	-5.020830554	4.31386E-05	0.032135701
cg06105935	-0.61617416	3.057600537	-5.020700888	4.31526E-05	0.032135701
cg05369791	-0.541694935	-0.781561931	-5.020567411	4.31669E-05	0.032135701
cg05715353	-0.706736489	2.671628516	-5.019024059	4.33331E-05	0.032135701
cg02217975	-0.735438266	-1.876354479	-5.018825551	4.33546E-05	0.032135701
cg10958598	-0.658331195	3.261962044	-5.018554857	4.33838E-05	0.032135701
cg15035590	-0.779179517	0.028210248	-5.018433303	4.33969E-05	0.032135701
cg09201906	-0.676161831	2.798824876	-5.017551316	4.34924E-05	0.032135701
cg19735532	-0.543854009	2.938951069	-5.016111123	4.36487E-05	0.032135701
cg25236277	0.569832253	2.456309509	5.015922735	4.36691E-05	0.032135701
cg02620935	-0.742083055	1.842124363	-5.015799019	4.36826E-05	0.032135701
cg14009504	-0.627357167	2.636170339	-5.015657227	4.3698E-05	0.032135701
cg01126380	-0.539061827	3.198021299	-5.015555946	4.37091E-05	0.032135701
cg18398009	-0.553681185	3.905700966	-5.015513679	4.37137E-05	0.032135701
cg15780078	-0.630319077	-1.091069397	-5.015126463	4.37558E-05	0.032135701
cg11804948	-0.614541562	3.410482285	-5.014702115	4.38021E-05	0.032135701
cg20841436	-0.757516459	0.579807267	-5.013936143	4.38858E-05	0.032135701
cg24213954	-0.648860489	0.147742457	-5.013764205	4.39046E-05	0.032135701
cg06978626	-0.487940311	4.250934874	-5.013611473	4.39213E-05	0.032135701
cg25347499	0.380348849	-0.329753693	5.013441648	4.39398E-05	0.032135701
cg15748376	-0.730270056	1.616111923	-5.013410833	4.39432E-05	0.032135701
cg24677444	-0.651191107	0.771237954	-5.012887872	4.40005E-05	0.032135701
cg21672524	-0.805717826	0.820528145	-5.012717752	4.40191E-05	0.032135701
cg20812837	-0.727408554	3.203742051	-5.012632272	4.40285E-05	0.032135701
cg05097801	-0.61264558	3.375185886	-5.012593603	4.40328E-05	0.032135701
cg15129971	-0.497172497	3.755057361	-5.010842073	4.42253E-05	0.032135701
cg27473073	-0.671218555	3.442712029	-5.01073269	4.42373E-05	0.032135701
cg11282156	-0.592431368	-2.096650328	-5.009984812	4.43198E-05	0.032135701
cg09143713	-0.510527572	-0.667586731	-5.009243206	4.44018E-05	0.032135701
cg27207809	-0.62748997	-0.958550195	-5.008503575	4.44837E-05	0.032135701
cg25889035	-0.507705734	-0.23645783	-5.008472639	4.44871E-05	0.032135701
cg13123425	-0.874778315	2.581719743	-5.007948946	4.45452E-05	0.032135701
cg26220781	-0.625587576	3.191336885	-5.007435652	4.46022E-05	0.032135701
cg16931554	-0.904563039	-1.36392608	-5.006208554	4.47387E-05	0.032135701
cg11854463	-0.63850989	3.454685493	-5.006117513	4.47489E-05	0.032135701
cg12089028	-0.552220751	-0.458341661	-5.005886197	4.47747E-05	0.032135701
cg02674055	-0.602290362	0.806063966	-5.005554977	4.48116E-05	0.032135701
cg18987912	-0.721966683	3.302098434	-5.005047738	4.48683E-05	0.032135701
cg14438189	-0.701127711	-0.189010741	-5.004434436	4.49369E-05	0.032135701
cg12699784	-0.791235934	2.801648873	-5.004331836	4.49484E-05	0.032135701
cg15750238	-0.642885494	3.462049579	-5.003995071	4.49861E-05	0.032135701
cg07341237	-0.664036401	3.516882259	-5.003105959	4.50858E-05	0.032135701
cg08766289	-0.632380772	3.258816104	-5.002929888	4.51056E-05	0.032135701
cg08201041	-0.776109947	2.660740862	-5.002706375	4.51308E-05	0.032135701
cg11098970	-0.575025835	3.213408186	-5.002352402	4.51706E-05	0.032135701
cg12339785	-0.563452373	2.899879546	-5.002065752	4.52028E-05	0.032135701

cg08209365	-0.594429201	3.013320826	-5.001919663	4.52193E-05	0.032135701
cg15999104	-0.851610171	3.729687799	-5.001199518	4.53005E-05	0.032135701
cg10044303	-0.653494333	2.354271924	-5.000817864	4.53436E-05	0.032135701
cg22162932	-0.869545129	2.605061262	-5.000762714	4.53498E-05	0.032135701
cg02757270	-0.505609083	3.679796635	-5.000623072	4.53656E-05	0.032135701
cg15421639	-0.632470217	3.53716436	-5.000566714	4.5372E-05	0.032135701
cg22594692	-0.710593351	0.590832318	-4.999804148	4.54583E-05	0.032135701
cg22345048	-0.691502533	-1.35240828	-4.999777144	4.54613E-05	0.032135701
cg05868449	-1.01692526	1.48232991	-4.999190274	4.55278E-05	0.032135701
cg02160323	-0.639407821	3.771073078	-4.999034499	4.55455E-05	0.032135701
cg08101375	-0.760661815	-2.345764258	-4.999011617	4.55481E-05	0.032135701
cg03124510	-0.526939028	-0.060291873	-4.998740423	4.55789E-05	0.032135701
cg14577406	-0.482133549	0.18667989	-4.997969931	4.56665E-05	0.032135701
cg05964000	-0.533002738	3.524542367	-4.99758715	4.57101E-05	0.032135701
cg08439787	-0.656971473	2.928425471	-4.997215952	4.57524E-05	0.032135701
cg01673485	-0.614322517	3.294936454	-4.996725968	4.58082E-05	0.032135701
cg24965026	-0.817363898	0.466327813	-4.996417027	4.58435E-05	0.032135701
cg19167683	-1.015706045	-3.429376069	-4.995590635	4.5938E-05	0.032135701
cg09488258	-0.805453259	-0.991084232	-4.995367722	4.59635E-05	0.032135701
cg20032501	-0.595282547	3.48100148	-4.995187445	4.59842E-05	0.032135701
cg01960160	-0.957546227	1.237991862	-4.995074077	4.59972E-05	0.032135701
cg16460860	-0.561135075	-1.074408405	-4.99467411	4.6043E-05	0.032135701
cg18610978	-0.556106356	0.730775386	-4.994524971	4.60602E-05	0.032135701
cg08466075	-0.469305175	2.712159137	-4.994379685	4.60768E-05	0.032135701
cg11136343	-0.74612353	1.810895859	-4.993329539	4.61976E-05	0.032135701
cg26986713	-0.540636164	3.688359302	-4.993322289	4.61984E-05	0.032135701
cg03472357	-0.574127354	3.306108179	-4.99288506	4.62488E-05	0.032135701
cg08280475	-0.769136339	-4.887660438	-4.992285899	4.63179E-05	0.032135701
cg02362017	-1.127313329	0.676021774	-4.991209821	4.64422E-05	0.032135701
cg08833631	-0.467816452	2.744129068	-4.990689716	4.65025E-05	0.032135701
cg04772328	-0.626233495	-1.101650381	-4.990584643	4.65146E-05	0.032135701
cg26791879	-0.999680264	-1.431530512	-4.989771046	4.6609E-05	0.032135701
cg21409029	-0.494657965	3.775362264	-4.988992764	4.66995E-05	0.032135701
cg17126573	-0.506064729	3.781850738	-4.988810171	4.67208E-05	0.032135701
cg26846943	-0.505411502	0.451418098	-4.988440559	4.67638E-05	0.032135701
cg16972496	-0.634584257	3.436751304	-4.987663124	4.68545E-05	0.032135701
cg08636886	-0.554789392	3.780714453	-4.986702752	4.69668E-05	0.032135701
cg23053977	-0.65466074	1.231422812	-4.985681309	4.70865E-05	0.032135701
cg23087877	-0.649746656	3.178606958	-4.985461196	4.71123E-05	0.032135701
cg11013329	-0.671645555	3.123928777	-4.98385184	4.73017E-05	0.032135701
cg07783576	-0.61210723	3.396761356	-4.983596748	4.73318E-05	0.032135701
cg19413958	-0.417472047	4.057832531	-4.983411973	4.73536E-05	0.032135701
cg22557012	-0.64592606	2.233238381	-4.982719872	4.74353E-05	0.032135701
cg23650385	-0.616672355	3.218965688	-4.982695606	4.74382E-05	0.032135701
cg01231611	-0.530435024	3.848107209	-4.982073273	4.75118E-05	0.032135701
cg00088135	-0.471420343	3.769122579	-4.981704683	4.75555E-05	0.032135701
cg00652189	-0.728754475	3.521987147	-4.981688242	4.75574E-05	0.032135701

cg10643162	-0.580508794	4.030830493	-4.98144727	4.7586E-05	0.032135701
cg07159131	-0.540083476	3.511400486	-4.980299185	4.77224E-05	0.032135701
cg11392922	-0.766510984	2.53677602	-4.979086434	4.78668E-05	0.032135701
cg00951800	-0.618315629	2.718044073	-4.97865325	4.79186E-05	0.032135701
cg02538667	-0.435090873	3.33283605	-4.978475458	4.79398E-05	0.032135701
cg01183713	-0.647237316	3.202769282	-4.977910701	4.80073E-05	0.032135701
cg05308867	-0.641773859	-0.826018593	-4.976444233	4.81831E-05	0.032135701
cg16162648	-0.835289249	0.447693031	-4.976407125	4.81876E-05	0.032135701
cg23751035	-0.743155058	0.740295479	-4.976272603	4.82037E-05	0.032135701
cg06509814	-0.554228879	-0.212666232	-4.974513423	4.84156E-05	0.032135701
cg11506483	-0.578753692	4.270096499	-4.973804007	4.85013E-05	0.032135701
cg00658820	-0.503603054	3.460923515	-4.972920068	4.86083E-05	0.032135701
cg23700491	-0.55519599	0.496476952	-4.972779052	4.86254E-05	0.032135701
cg05168279	0.543045187	0.866900262	4.972635795	4.86427E-05	0.032135701
cg01070197	0.699207267	3.386533138	4.972525347	4.86561E-05	0.032135701
cg21318481	-0.594860979	2.836103754	-4.972436838	4.86669E-05	0.032135701
cg11048637	-0.743979488	3.065267264	-4.971990921	4.8721E-05	0.032135701
cg12755507	-0.539628646	2.807228367	-4.971727905	4.87529E-05	0.032135701
cg08192891	-0.741214863	3.311958137	-4.971585302	4.87703E-05	0.032135701
cg24451843	-0.827705116	2.263668526	-4.970805682	4.88652E-05	0.032135701
cg12267933	-0.517426602	3.277114874	-4.969972442	4.89668E-05	0.032135701
cg05563022	-1.103576989	2.210691184	-4.969916702	4.89736E-05	0.032135701
cg09810730	-0.548682047	3.772992701	-4.969848266	4.89819E-05	0.032135701
cg25948328	-0.539267321	3.509832978	-4.969314437	4.90472E-05	0.032135701
cg13530751	-0.425698445	0.246774177	-4.969298707	4.90491E-05	0.032135701
cg13558754	-0.666210419	-2.926853275	-4.969099204	4.90735E-05	0.032135701
cg06094757	-0.72685325	3.176157778	-4.969090246	4.90746E-05	0.032135701
cg01064363	-0.501285642	3.341466778	-4.968453862	4.91525E-05	0.032135701
cg00074313	-0.514081709	3.309793195	-4.96825636	4.91767E-05	0.032135701
cg18320290	-0.68636164	2.916884723	-4.968254384	4.91769E-05	0.032135701
cg20469666	-0.5781214	4.005496464	-4.968155124	4.91891E-05	0.032135701
cg09474959	-0.654771358	2.873054135	-4.967976706	4.9211E-05	0.032135701
cg00866540	1.504836109	2.139041934	4.967904612	4.92198E-05	0.032135701
cg15472735	-0.506021256	4.267496994	-4.967763833	4.92371E-05	0.032135701
cg08074830	-0.751687807	0.440291385	-4.967523402	4.92666E-05	0.032135701
cg06031797	-0.638592948	-2.358483101	-4.966825866	4.93524E-05	0.032135701
cg25029696	-0.806919737	2.357359172	-4.966710475	4.93666E-05	0.032135701
cg26455201	-0.452492188	3.896295353	-4.966572135	4.93836E-05	0.032135701
cg10716548	-0.669380926	3.052652754	-4.965776652	4.94817E-05	0.032135701
cg02769615	-0.766297474	1.18127326	-4.965726542	4.94878E-05	0.032135701
cg05840218	-0.598655312	4.086523971	-4.965715658	4.94892E-05	0.032135701
cg25422766	-0.73149564	3.09442807	-4.964787416	4.96038E-05	0.032135701
cg05645109	-0.437014353	4.132446702	-4.963008986	4.98243E-05	0.032135701
cg19335711	-0.475194195	3.914977451	-4.96291609	4.98358E-05	0.032135701
cg24276053	-0.680934101	2.272498719	-4.962876823	4.98407E-05	0.032135701
cg03398194	-0.638672576	2.727913059	-4.962753187	4.98561E-05	0.032135701
cg13506496	-0.449636782	0.484541272	-4.962738952	4.98578E-05	0.032135701

cg01354090	-0.688228682	3.007042034	-4.962679518	4.98652E-05	0.032135701
cg19116204	-0.757577082	2.374137275	-4.962508708	4.98865E-05	0.032135701
cg14715508	-0.745345061	-0.367312174	-4.962365212	4.99043E-05	0.032135701
cg18014789	-0.69862991	-3.213333774	-4.962352217	4.99059E-05	0.032135701
cg11849213	-0.526629338	3.604639821	-4.962321504	4.99098E-05	0.032135701
cg25734842	-0.626346025	-1.271973719	-4.961813349	4.9973E-05	0.032135701
cg10747028	-0.757606123	3.179513996	-4.961436595	5.002E-05	0.032135701
cg24773234	-0.720533962	2.81971658	-4.961361145	5.00294E-05	0.032135701
cg03546284	-0.674003233	-0.72472814	-4.960349221	5.01558E-05	0.032135701
cg13553509	-0.518905962	3.342013659	-4.959137471	5.03076E-05	0.032135701
cg23401088	-0.652281753	0.305319065	-4.95909133	5.03134E-05	0.032135701
cg09321133	-0.542573976	3.629794139	-4.958593773	5.03758E-05	0.032135701
cg24530107	-0.516769657	3.688308408	-4.958438198	5.03954E-05	0.032135701
cg14866151	-0.693980011	2.509297868	-4.958198651	5.04255E-05	0.032135701
cg20675950	-0.705734599	3.372873839	-4.957996414	5.04509E-05	0.032135701
cg20437132	-0.923408024	2.42843901	-4.957963615	5.0455E-05	0.032135701
cg26351679	0.594091477	2.517400814	4.95788965	5.04643E-05	0.032135701
cg26245142	-0.546688744	4.23584156	-4.957765431	5.048E-05	0.032135701
cg09233300	-0.934024436	2.141661302	-4.957591141	5.05019E-05	0.032135701
cg15818534	-0.576261375	3.812747317	-4.957164636	5.05557E-05	0.032135701
cg25702500	0.697087869	2.612308055	4.957097649	5.05641E-05	0.032135701
cg19360064	-0.759416174	-2.10915411	-4.957063362	5.05684E-05	0.032135701
cg17389149	-0.657347421	3.21772121	-4.957038753	5.05715E-05	0.032135701
cg04074353	-0.829192606	2.740976429	-4.956893902	5.05898E-05	0.032135701
cg21350111	-0.689452713	3.260295048	-4.956844603	5.0596E-05	0.032135701
cg03062196	-0.506364337	3.56108738	-4.956748542	5.06081E-05	0.032135701
cg02927058	-0.573606186	0.604956664	-4.956146749	5.06841E-05	0.032135701
cg07684583	-0.970308892	1.379306626	-4.95604045	5.06976E-05	0.032135701
cg19325597	-0.538003349	2.137224929	-4.955151831	5.081E-05	0.032135701
cg26294177	-0.641702087	2.622000317	-4.954856315	5.08475E-05	0.032135701
cg24426778	-0.507377093	0.774258117	-4.954351117	5.09116E-05	0.032135701
cg03365937	-0.626153467	-0.545356872	-4.954243372	5.09253E-05	0.032135701
cg07794083	-0.621048302	2.983298301	-4.954189939	5.09321E-05	0.032135701
cg01961092	-0.502260652	2.075742947	-4.954090294	5.09447E-05	0.032135701
cg27087428	-0.51131324	-0.456396994	-4.953864517	5.09734E-05	0.032135701
cg18923673	-0.509617491	3.315780907	-4.953750753	5.09879E-05	0.032135701
cg07148407	-0.592541477	0.152630622	-4.953324245	5.10421E-05	0.032135701
cg07878481	-0.697095273	2.746058199	-4.953232468	5.10538E-05	0.032135701
cg14697542	-0.508971823	-0.241841077	-4.953040496	5.10783E-05	0.032135701
cg17526301	-0.637955408	-2.418475279	-4.952907027	5.10953E-05	0.032135701
cg04155644	-0.74197002	2.325615036	-4.952707045	5.11207E-05	0.032135701
cg00590881	-0.744262903	1.537531423	-4.951997884	5.12112E-05	0.032135701
cg21131711	-0.488677365	3.027773508	-4.95156101	5.12671E-05	0.032135701
cg27491818	-0.661533341	0.16607107	-4.951021249	5.13361E-05	0.032135701
cg24997941	-0.718561664	2.073128874	-4.949672006	5.15091E-05	0.032135701
cg20525861	-0.771694093	-1.890339609	-4.949646931	5.15124E-05	0.032135701
cg15158642	-0.631500866	3.262841208	-4.949616806	5.15162E-05	0.032135701

cg05341234	-1.269579968	2.334769821	-4.949117849	5.15804E-05	0.032135701
cg03738656	-0.630290115	-0.388731667	-4.948922649	5.16055E-05	0.032135701
cg19031981	-0.713292027	2.528121505	-4.948702681	5.16338E-05	0.032135701
cg05431964	-0.542726194	3.918385254	-4.948233664	5.16942E-05	0.032135701
cg03501131	-0.565999969	3.509191116	-4.947959733	5.17296E-05	0.032135701
cg24907183	-0.722694369	2.151595488	-4.947822108	5.17473E-05	0.032135701
cg15305791	-0.48258669	0.609813676	-4.947542884	5.17834E-05	0.032135701
cg25575520	-0.615649628	3.211519166	-4.947016092	5.18515E-05	0.032135701
cg07860110	-0.570668286	3.063167381	-4.946677724	5.18952E-05	0.032135701
cg07251280	-0.545608044	0.062324601	-4.946441803	5.19258E-05	0.032135701
cg09933108	-0.529254906	3.408885979	-4.946438749	5.19262E-05	0.032135701
cg12840245	-0.582432537	3.023815674	-4.946351085	5.19375E-05	0.032135701
cg19588506	-0.837382462	3.189192336	-4.946317622	5.19419E-05	0.032135701
cg20667822	-0.880641212	3.062574853	-4.946315129	5.19422E-05	0.032135701
cg12144121	-0.513113883	0.574261894	-4.946222993	5.19541E-05	0.032135701
cg13708378	-0.983321929	-0.542736974	-4.946145604	5.19641E-05	0.032135701
cg24444839	-0.624282254	0.28055504	-4.946115763	5.1968E-05	0.032135701
cg26821758	-0.614562452	2.928738985	-4.945366609	5.20652E-05	0.032135701
cg17768123	-0.504548302	3.241593601	-4.945298212	5.20741E-05	0.032135701
cg12064581	-0.655104064	3.338695067	-4.945255198	5.20797E-05	0.032135701
cg18731761	-0.510960682	3.806339823	-4.945024301	5.21097E-05	0.032135701
cg27528859	-0.62923465	3.179579312	-4.94463127	5.21608E-05	0.032135701
cg10860713	-0.905454779	2.929360106	-4.944307396	5.22029E-05	0.032135701
cg07890600	-0.455341905	-1.59982251	-4.943823565	5.2266E-05	0.032135701
cg23694028	-0.75574689	3.150903236	-4.943713711	5.22803E-05	0.032135701
cg07089813	-0.456329406	-1.777240954	-4.943518954	5.23057E-05	0.032135701
cg16319436	-0.718728174	3.092106947	-4.943147295	5.23542E-05	0.032137631
cg05740473	-0.857269738	1.940568421	-4.942286938	5.24667E-05	0.032140709
cg13695286	-0.626795641	-0.144514042	-4.942212739	5.24764E-05	0.032140709
cg21364568	-0.467119832	3.806405635	-4.941644756	5.25508E-05	0.032140709
cg01729652	-0.683825051	2.591711024	-4.941493037	5.25707E-05	0.032140709
cg16086021	-0.73726801	3.296009129	-4.941375545	5.25861E-05	0.032140709
cg14404316	-0.651684041	3.557783932	-4.940886681	5.26502E-05	0.032152183
cg22623776	-0.440516026	0.554244072	-4.940380802	5.27167E-05	0.032163364
cg01022801	-0.595339729	-2.185047162	-4.939831421	5.2789E-05	0.032163364
cg22403365	-0.502424931	-0.068962962	-4.939711788	5.28048E-05	0.032163364
cg27547329	-0.613141728	3.265273844	-4.939116781	5.28832E-05	0.032176409
cg00172225	-0.591560713	3.340736759	-4.938860388	5.2917E-05	0.032176409
cg19384905	-0.65258512	-0.591053125	-4.938222606	5.30013E-05	0.032199999
cg09870559	-0.453872254	3.793427619	-4.93722094	5.31339E-05	0.032212196
cg12700606	-0.645851783	3.578039972	-4.937157965	5.31422E-05	0.032212196
cg19889495	-0.506175205	3.956002786	-4.937040658	5.31578E-05	0.032212196
cg01991859	-0.649202636	3.623218535	-4.935481724	5.33649E-05	0.032310066
cg07259163	-0.493612614	3.785529282	-4.934450737	5.35023E-05	0.032365607
cg14532664	-0.556684527	3.088963857	-4.933868443	5.35801E-05	0.032367861
cg27088726	-0.500998405	-0.637292647	-4.93347495	5.36327E-05	0.032367861
cg08678000	-0.697854123	2.236087064	-4.933030868	5.36922E-05	0.032367861

cg24598158	-0.770125028	0.146800319	-4.932654652	5.37426E-05	0.032367861
cg24837192	-0.550619671	4.21043443	-4.932605581	5.37491E-05	0.032367861
cg03317075	-0.491978624	3.143419033	-4.932096777	5.38174E-05	0.032367861
cg00890249	-0.836266218	2.437072619	-4.931356801	5.39169E-05	0.032367861
cg12518360	-0.829021171	-4.654138666	-4.930850179	5.3985E-05	0.032367861
cg15688980	-0.430851689	4.206302673	-4.930565781	5.40234E-05	0.032367861
cg27565457	-0.636599539	3.801997698	-4.929511932	5.41656E-05	0.032367861
cg04537184	-0.669246416	-0.891858632	-4.928372678	5.43197E-05	0.032367861
cg16912009	-0.802671225	1.749886717	-4.928338977	5.43243E-05	0.032367861
cg05877899	-0.552222743	3.944013932	-4.928260137	5.4335E-05	0.032367861
cg20646420	-0.434311331	4.505772461	-4.928133951	5.43521E-05	0.032367861
cg05548334	-0.645409254	3.338242056	-4.927867438	5.43883E-05	0.032367861
cg01167029	-0.606425985	3.137230704	-4.927835852	5.43925E-05	0.032367861
cg24859006	-0.673124397	3.014993263	-4.927792417	5.43984E-05	0.032367861
cg00636308	-0.556196261	-2.082729716	-4.927672205	5.44148E-05	0.032367861
cg18315007	-0.577180024	3.537741672	-4.927538865	5.44329E-05	0.032367861
cg13702833	0.749980792	1.778926953	4.927059548	5.4498E-05	0.032367861
cg23746283	-1.043177938	1.802856929	-4.92699164	5.45072E-05	0.032367861
cg01477493	-0.623844515	3.300958291	-4.926961942	5.45113E-05	0.032367861
cg22479441	-0.709772756	3.285770427	-4.925853926	5.46622E-05	0.03243028
cg14524764	-0.635295963	0.807650706	-4.925166372	5.4756E-05	0.032442738
cg26374986	-0.553223726	2.953107322	-4.925029184	5.47748E-05	0.032442738
cg24354784	-0.670397097	2.820423904	-4.924652735	5.48262E-05	0.032446095
cg12302983	-0.519863733	3.217746601	-4.923948015	5.49227E-05	0.032468922
cg25823893	-0.595789678	0.323302421	-4.923592502	5.49715E-05	0.032468922
cg21413173	-0.647118858	-2.026090111	-4.923022256	5.50497E-05	0.032468922
cg01249337	-0.710868861	2.944737945	-4.923000364	5.50527E-05	0.032468922
cg13808473	-0.489281008	3.819187015	-4.922303985	5.51485E-05	0.032468922
cg01665554	-0.648564563	2.417728492	-4.92214298	5.51706E-05	0.032468922
cg16573346	-0.535097759	-0.336649392	-4.92191413	5.52022E-05	0.032468922
cg09960211	-0.445927133	-0.635393368	-4.921701162	5.52315E-05	0.032468922
cg07038689	-0.774632301	3.157920266	-4.920913474	5.53402E-05	0.032470249
cg16548780	-0.430707692	-0.852844806	-4.920719082	5.5367E-05	0.032470249
cg25947994	-0.590816918	4.009979055	-4.920325848	5.54214E-05	0.032470249
cg19970014	-0.577475225	3.42632071	-4.920236792	5.54337E-05	0.032470249
cg21835089	-0.64554836	3.253459865	-4.919405736	5.55487E-05	0.032470249
cg08064269	-0.445231909	3.98764896	-4.919240388	5.55717E-05	0.032470249
cg11322076	-0.545662895	3.699652443	-4.918821687	5.56298E-05	0.032470249
cg05203607	-0.575771941	3.825462261	-4.918622083	5.56575E-05	0.032470249
cg21484515	-0.654108567	0.234408734	-4.918181484	5.57187E-05	0.032470249
cg19137288	-0.534637276	3.250801931	-4.918111616	5.57284E-05	0.032470249
cg22124751	-0.543830336	2.710572338	-4.918042951	5.5738E-05	0.032470249
cg18914863	-0.586871193	0.336421226	-4.9169076	5.58961E-05	0.032535614
cg13676759	-0.702877605	2.825298401	-4.915849016	5.60439E-05	0.032536877
cg03937203	-0.805165983	-0.084092971	-4.915811472	5.60492E-05	0.032536877
cg14705173	-0.706566747	3.242676522	-4.915461294	5.60982E-05	0.032536877
cg26413163	-0.552266743	2.932791527	-4.914697928	5.62052E-05	0.032536877

cg20332660	-0.908119294	3.199984532	-4.914602566	5.62185E-05	0.032536877
cg19110014	-0.717142248	2.924347047	-4.914578598	5.62219E-05	0.032536877
cg08675147	-0.778055439	2.856050036	-4.914475845	5.62363E-05	0.032536877
cg25300624	-0.54009513	0.729969604	-4.913218861	5.6413E-05	0.032536877
cg11283152	-0.791362255	2.805928906	-4.912331227	5.65381E-05	0.032536877
cg05424239	-0.728574501	3.332269138	-4.912147389	5.6564E-05	0.032536877
cg26894722	-0.64968613	2.516284541	-4.911921356	5.6596E-05	0.032536877
cg13393588	-0.714630074	0.251347109	-4.911828311	5.66091E-05	0.032536877
cg24357149	-0.479941302	2.746261742	-4.910648629	5.6776E-05	0.032536877
cg17321476	-0.556619995	0.415109926	-4.909997011	5.68684E-05	0.032536877
cg07649835	-0.592676888	3.737408393	-4.909928107	5.68782E-05	0.032536877
cg03516599	-0.656266057	3.590179115	-4.909316424	5.69651E-05	0.032536877
cg03065516	-0.616518717	3.268906749	-4.908464622	5.70863E-05	0.032536877
cg23028728	-0.602601058	2.829549032	-4.908307189	5.71087E-05	0.032536877
cg01349007	-0.493760688	3.786797243	-4.907801656	5.71808E-05	0.032536877
cg05169578	-0.785107222	2.63096479	-4.907766096	5.71859E-05	0.032536877
cg10430362	-0.734553629	2.938597103	-4.907623588	5.72063E-05	0.032536877
cg06574960	-0.585881592	-1.288205123	-4.907314584	5.72504E-05	0.032536877
cg15495648	-0.479826246	4.153111363	-4.907222109	5.72636E-05	0.032536877
cg09952891	-0.585301612	4.007104775	-4.907170912	5.72709E-05	0.032536877
cg02927485	-0.671086163	-2.912068375	-4.906365205	5.73862E-05	0.032536877
cg16661114	-0.592294133	0.938052412	-4.90568221	5.74841E-05	0.032536877
cg05206321	-0.536710353	4.076714222	-4.905612803	5.74941E-05	0.032536877
cg14711743	-0.633367681	2.45112877	-4.90561259	5.74941E-05	0.032536877
cg09859617	-0.597279856	2.702686281	-4.905464965	5.75153E-05	0.032536877
cg05727926	-0.459935931	3.882250531	-4.90536584	5.75295E-05	0.032536877
cg26404235	-0.534068972	3.719187354	-4.904892606	5.75975E-05	0.032536877
cg16029900	-0.609660711	3.664272617	-4.90463202	5.7635E-05	0.032536877
cg16608357	-1.031711913	2.903263555	-4.904565703	5.76445E-05	0.032536877
cg25451303	-0.76021007	-4.225884166	-4.904087554	5.77134E-05	0.032536877
cg20411792	-0.690419491	2.866456369	-4.903635902	5.77785E-05	0.032536877
cg16390380	-1.104739048	0.293476139	-4.903576885	5.7787E-05	0.032536877
cg05832094	-0.550345455	0.329094024	-4.903141542	5.78498E-05	0.032536877
cg04374059	-0.745673784	2.395334691	-4.903045113	5.78637E-05	0.032536877
cg06284762	-0.795433006	3.079940947	-4.902940052	5.78789E-05	0.032536877
cg20749792	-0.696889684	-2.296181387	-4.901864003	5.80346E-05	0.032536877
cg08653633	-0.725737629	3.240769727	-4.901047861	5.81529E-05	0.032536877
cg01534818	0.433757013	2.590105318	4.900548666	5.82254E-05	0.032536877
cg01116942	-0.676699202	2.992111065	-4.900205039	5.82754E-05	0.032536877
cg15014137	-0.809585152	2.435057255	-4.8997962	5.83349E-05	0.032536877
cg08482307	-0.471582542	-0.991820706	-4.899117538	5.84338E-05	0.032536877
cg22954978	-0.510020216	0.036485569	-4.898827409	5.84761E-05	0.032536877
cg10256726	-0.509693082	-1.067194863	-4.898741111	5.84887E-05	0.032536877
cg03462249	-0.758296118	2.856415645	-4.898231359	5.85632E-05	0.032536877
cg19145429	-0.629709509	3.641722933	-4.897981944	5.85996E-05	0.032536877
cg23266597	-0.701767247	3.14115907	-4.897685945	5.8643E-05	0.032536877
cg18800748	-0.458417976	-1.395896041	-4.897657526	5.86471E-05	0.032536877

cg09595044	-0.47558956	3.916645393	-4.896231152	5.88563E-05	0.032536877
cg03041055	-0.560173503	3.372533863	-4.896175822	5.88644E-05	0.032536877
cg10318641	-0.544215862	0.614451606	-4.895307494	5.89922E-05	0.032536877
cg07397913	-0.435028546	-0.03086751	-4.894519651	5.91083E-05	0.032536877
cg16656041	-0.672877895	-1.018329456	-4.894338169	5.91351E-05	0.032536877
cg16911487	-0.637100028	2.795781945	-4.894278003	5.9144E-05	0.032536877
cg05868019	-0.581982522	3.518810354	-4.893702443	5.9229E-05	0.032536877
cg25417335	-0.64308461	-0.137896126	-4.893558174	5.92503E-05	0.032536877
cg06598634	-0.729616969	0.776664757	-4.893001432	5.93327E-05	0.032536877
cg10333212	-0.757350119	2.745785409	-4.892875825	5.93513E-05	0.032536877
cg12003289	-0.490910823	3.5690524	-4.892436839	5.94164E-05	0.032536877
cg22628512	-0.521165753	-0.158253432	-4.892120618	5.94633E-05	0.032536877
cg21640312	-0.930038009	2.672894016	-4.891990686	5.94826E-05	0.032536877
cg08868601	-0.696470535	1.546188202	-4.891672691	5.95299E-05	0.032536877
cg05548535	-0.65516022	3.425113317	-4.891551637	5.95479E-05	0.032536877
cg13473086	-0.800003233	0.30770842	-4.891544059	5.9549E-05	0.032536877
cg01475926	-0.396949193	4.082482511	-4.891520207	5.95525E-05	0.032536877
cg06247422	-0.662594205	0.190054305	-4.891099432	5.96151E-05	0.032536877
cg07089617	-0.541315012	-0.278653024	-4.890674944	5.96783E-05	0.032536877
cg12772372	-0.67451608	2.300120254	-4.889931676	5.97892E-05	0.032536877
cg21138486	-0.660533809	2.943021317	-4.889897392	5.97943E-05	0.032536877
cg19960165	-0.550639225	4.120889266	-4.889011178	5.99267E-05	0.032536877
cg09237172	-0.621589528	3.836204935	-4.888042766	6.00718E-05	0.032536877
cg14081465	-0.570750189	-1.149423109	-4.887913023	6.00913E-05	0.032536877
cg12062505	-0.413062663	-0.705019149	-4.887744427	6.01166E-05	0.032536877
cg13917614	-0.470711019	-1.114984393	-4.887562321	6.01439E-05	0.032536877
cg01743793	-0.700000775	-0.15492984	-4.887509057	6.01519E-05	0.032536877
cg03288217	-0.70127641	0.827856432	-4.887439174	6.01624E-05	0.032536877
cg17660251	-0.541911725	3.368899835	-4.886878462	6.02467E-05	0.032536877
cg05855157	-0.602108567	3.568283948	-4.886568901	6.02932E-05	0.032536877
cg14282050	-0.612694088	3.532105077	-4.886567417	6.02935E-05	0.032536877
cg18366199	-0.735222886	2.95402157	-4.886379625	6.03217E-05	0.032536877
cg15234410	-0.519006462	2.926510597	-4.886305663	6.03329E-05	0.032536877
cg03615630	-0.518415248	-0.720080553	-4.886107264	6.03628E-05	0.032536877
cg01566282	-0.898228541	2.4971074	-4.886094617	6.03647E-05	0.032536877
cg22836912	-0.623693591	3.359114458	-4.884975394	6.05336E-05	0.032536877
cg19644750	-0.512960965	-0.764138526	-4.884922966	6.05415E-05	0.032536877
cg14177623	-0.661856007	-0.742657859	-4.884908837	6.05437E-05	0.032536877
cg14196395	-0.693768011	-0.36963029	-4.884705328	6.05744E-05	0.032536877
cg05403383	-0.580417341	-0.629976335	-4.884227775	6.06467E-05	0.032536877
cg08870337	0.453005154	2.408913956	4.8838933	6.06974E-05	0.032536877
cg11246948	-0.730315957	-1.910424175	-4.88361301	6.07399E-05	0.032536877
cg13504410	-0.605581029	2.61557663	-4.883355242	6.0779E-05	0.032536877
cg24003503	-0.836523328	3.226546801	-4.883168391	6.08073E-05	0.032536877
cg12234099	-0.741584541	1.822388232	-4.882748964	6.0871E-05	0.032536877
cg19189688	-0.59573159	-1.048324859	-4.882168443	6.09593E-05	0.032536877
cg02145259	-0.446146468	1.573296095	-4.881732306	6.10257E-05	0.032536877

cg22510134	-0.55619084	-0.193660148	-4.881143326	6.11156E-05	0.032536877
cg14612684	-0.498739171	3.35595897	-4.880585289	6.12008E-05	0.032536877
cg02125941	-1.035842913	2.054550727	-4.880578755	6.12018E-05	0.032536877
cg12712409	1.541344067	3.363617267	4.88028698	6.12464E-05	0.032536877
cg01999476	-0.597084313	3.430352644	-4.880071516	6.12793E-05	0.032536877
cg03756034	-0.992935208	2.089871255	-4.879442997	6.13756E-05	0.032536877
cg14575449	-0.448390949	3.75773232	-4.87912911	6.14237E-05	0.032536877
cg01072612	-0.612742479	3.38421094	-4.87904825	6.14361E-05	0.032536877
cg03256424	-0.737422854	0.075024075	-4.878951856	6.14509E-05	0.032536877
cg03813471	-0.703938885	-0.612740661	-4.878781893	6.1477E-05	0.032536877
cg25800379	-0.840660499	-2.94040159	-4.878310454	6.15494E-05	0.032536877
cg11914037	-0.480280348	0.69605732	-4.878046407	6.159E-05	0.032536877
cg15417875	-0.732346468	0.554696065	-4.877799422	6.1628E-05	0.032536877
cg17140186	-0.630854186	3.247412927	-4.877682017	6.1646E-05	0.032536877
cg00175987	-0.431044165	4.216603142	-4.877508755	6.16727E-05	0.032536877
cg08647621	-0.667161764	3.066789632	-4.877150437	6.17279E-05	0.032536877
cg25431990	-0.713614752	3.118395728	-4.877068592	6.17405E-05	0.032536877
cg17248521	-0.684420876	3.165388674	-4.876946772	6.17593E-05	0.032536877
cg16074241	-0.942464464	2.12579535	-4.876590409	6.18143E-05	0.032536877
cg10568207	-0.409482107	3.438490643	-4.87653269	6.18232E-05	0.032536877
cg04837157	-0.580513778	-1.414254699	-4.875782301	6.19392E-05	0.032536877
cg08623051	-0.737228723	-3.07113576	-4.875340259	6.20076E-05	0.032536877
cg06015379	-0.697201892	3.261922361	-4.875335922	6.20082E-05	0.032536877
cg04168675	-1.114052277	0.448246322	-4.875310193	6.20122E-05	0.032536877
cg02130329	-0.801328305	1.327119602	-4.875003439	6.20597E-05	0.032536877
cg01211496	-0.610693854	3.026280004	-4.874951336	6.20678E-05	0.032536877
cg00762317	0.632140508	3.390914858	4.874321381	6.21655E-05	0.032536877
cg24184528	-0.467208586	3.949670172	-4.874160117	6.21906E-05	0.032536877
cg17052675	-0.815954732	-0.254110686	-4.874039071	6.22094E-05	0.032536877
cg13745182	-0.468689354	3.173861582	-4.873965985	6.22207E-05	0.032536877
cg18537383	-0.599879158	0.006837464	-4.872835457	6.23966E-05	0.032536877
cg14866338	-0.728064751	2.086791281	-4.872680184	6.24208E-05	0.032536877
cg10398186	-0.566955864	3.975908499	-4.87213778	6.25054E-05	0.032536877
cg07747254	-0.444437229	3.839187528	-4.872100451	6.25113E-05	0.032536877
cg14518796	-0.55023701	-1.047420819	-4.871796276	6.25588E-05	0.032536877
cg00594137	-0.500136118	3.747793985	-4.87167929	6.2577E-05	0.032536877
cg02641420	-0.658757124	2.499302465	-4.871665321	6.25792E-05	0.032536877
cg24632827	-0.617874672	3.526006814	-4.871464481	6.26106E-05	0.032536877
cg02702688	-0.426750019	0.073793012	-4.871086859	6.26697E-05	0.032536877
cg14160911	-0.997748146	2.023954892	-4.870912156	6.2697E-05	0.032536877
cg04331429	-0.61284609	-0.540031712	-4.870551029	6.27536E-05	0.032536877
cg16612566	-0.952833716	2.683683648	-4.870509678	6.27601E-05	0.032536877
cg17599241	-0.773368251	-0.333047557	-4.869808588	6.28701E-05	0.032536877
cg19410636	-0.559646399	-0.489731512	-4.869797552	6.28718E-05	0.032536877
cg25728477	-0.480966306	0.126477675	-4.869665292	6.28926E-05	0.032536877
cg01334366	-0.497442853	3.09211104	-4.869590369	6.29043E-05	0.032536877
cg04937388	-0.573109679	4.296599222	-4.86863516	6.30546E-05	0.032536877

cg10769416	-0.931844333	2.692942742	-4.868603241	6.30596E-05	0.032536877
cg27549186	-0.583974522	0.559336427	-4.86856336	6.30659E-05	0.032536877
cg19778582	-0.457255935	3.690712025	-4.868370747	6.30962E-05	0.032536877
cg15485511	-0.607159617	3.765565563	-4.868175647	6.3127E-05	0.032536877
cg25547630	-0.795375392	1.492968099	-4.868138661	6.31328E-05	0.032536877
cg00641391	-0.672021448	3.039560672	-4.867731164	6.31971E-05	0.032536877
cg14521435	-0.627687515	3.172792733	-4.867265475	6.32706E-05	0.032536877
cg10275328	-0.878741663	2.563765923	-4.867140885	6.32903E-05	0.032536877
cg02641941	-0.726823304	2.905776756	-4.866890736	6.33299E-05	0.032536877
cg11207969	-0.60055517	3.437258608	-4.866888237	6.33303E-05	0.032536877
cg07696519	-0.882981568	2.877764025	-4.866357075	6.34143E-05	0.032536877
cg07010337	-0.580040516	3.40624928	-4.866145206	6.34479E-05	0.032536877
cg17911021	-0.586605808	-1.856416826	-4.866002834	6.34705E-05	0.032536877
cg01160409	-0.868173271	1.373951077	-4.865846358	6.34953E-05	0.032536877
cg26229208	-0.585442477	0.552047056	-4.865771582	6.35071E-05	0.032536877
cg10445545	-0.706035242	-0.754814751	-4.865674742	6.35225E-05	0.032536877
cg02459037	-0.53536753	3.267762639	-4.865216772	6.35952E-05	0.032536877
cg21777304	-0.889020885	2.468126712	-4.8643155	6.37385E-05	0.032536877
cg26586163	-0.853853661	2.196514223	-4.86425306	6.37484E-05	0.032536877
cg21476837	-0.530634666	3.649839889	-4.864073981	6.3777E-05	0.032536877
cg23953034	-0.713970633	3.165209111	-4.863753646	6.3828E-05	0.032536877
cg24026331	-0.564723157	3.903217755	-4.863457078	6.38753E-05	0.032536877
cg19960114	-0.460490846	-1.224346053	-4.863402821	6.3884E-05	0.032536877
cg08638965	-0.599029934	1.30073738	-4.862382557	6.40469E-05	0.032536877
cg12556802	-0.776442266	-0.072724423	-4.862074428	6.40963E-05	0.032536877
cg04174382	-0.686110737	3.178463543	-4.861851622	6.41319E-05	0.032536877
cg15123761	-0.534986657	0.304783293	-4.861097479	6.42528E-05	0.032536877
cg09660387	-0.590303666	3.679827995	-4.860629949	6.43279E-05	0.032536877
cg13542073	-0.820885051	0.740847935	-4.86022408	6.43932E-05	0.032536877
cg09701422	-0.852370655	2.194258628	-4.860148755	6.44053E-05	0.032536877
cg25370180	-0.468857673	4.442767542	-4.859565147	6.44992E-05	0.032536877
cg20325293	-0.824393431	-0.157289084	-4.859372295	6.45303E-05	0.032536877
cg13992929	-0.848643496	2.23075276	-4.85901317	6.45882E-05	0.032536877
cg02403899	-0.59323813	3.345533724	-4.858974295	6.45945E-05	0.032536877
cg09059789	-0.836881392	2.697507363	-4.858825678	6.46185E-05	0.032536877
cg18260919	-0.818596273	2.809272296	-4.858784387	6.46251E-05	0.032536877
cg03725688	-0.635945404	2.89799452	-4.858628333	6.46503E-05	0.032536877
cg08217336	-0.567721997	3.967637783	-4.858609857	6.46533E-05	0.032536877
cg19299912	-0.573758517	0.536697981	-4.858568569	6.466E-05	0.032536877
cg27133206	-0.568167509	3.174179554	-4.858412208	6.46852E-05	0.032536877
cg17474785	-0.586967642	2.642144398	-4.858200722	6.47194E-05	0.032536877
cg06024391	-0.553568425	0.189770933	-4.858072425	6.47402E-05	0.032536877
cg19724154	-0.553855879	3.667195486	-4.857983634	6.47545E-05	0.032536877
cg13480043	-0.729349369	2.475647448	-4.857410457	6.48473E-05	0.032536877
cg21583440	-0.52218153	1.849354526	-4.857151852	6.48892E-05	0.032536877
cg00023948	-0.685979863	2.621233341	-4.856362464	6.50173E-05	0.032536877
cg08018572	-1.139189914	-1.359587433	-4.856277305	6.50311E-05	0.032536877

cg03213549	-0.563536398	3.29707608	-4.855734109	6.51194E-05	0.032536877
cg07722359	-0.66851945	2.881629689	-4.855428236	6.51692E-05	0.032536877
cg25150155	-0.464078121	0.552493168	-4.855344431	6.51828E-05	0.032536877
cg20802692	-0.700400617	3.030086204	-4.854705538	6.52869E-05	0.032536877
cg21861305	-0.489999279	3.443089317	-4.854508078	6.53191E-05	0.032536877
cg00773446	-0.866143915	2.564006198	-4.854266789	6.53585E-05	0.032536877
cg11774469	-0.853370755	0.825102185	-4.854104047	6.53851E-05	0.032536877
cg24114822	-0.603691409	2.463263842	-4.854032438	6.53968E-05	0.032536877
cg23732557	-0.657717057	3.706976058	-4.852736624	6.56088E-05	0.032536877
cg18372136	-0.582154811	3.163552548	-4.852369146	6.56691E-05	0.032536877
cg24582365	-0.442777251	4.20058164	-4.85211893	6.57101E-05	0.032536877
cg03686111	-0.622581278	0.56811101	-4.852102934	6.57127E-05	0.032536877
cg18901519	-0.681346204	2.599697906	-4.851828201	6.57579E-05	0.032536877
cg01998667	-0.65997683	0.557551023	-4.851543356	6.58047E-05	0.032536877
cg04135778	-0.816755672	2.329426266	-4.850695297	6.59442E-05	0.032536877
cg23076903	-0.429387244	3.880958196	-4.849542924	6.61343E-05	0.032536877
cg00253269	-0.464302864	2.912098122	-4.849461395	6.61478E-05	0.032536877
cg23528975	-0.568721607	0.030406837	-4.849287916	6.61765E-05	0.032536877
cg04613576	-0.818565637	1.857677517	-4.848987233	6.62262E-05	0.032536877
cg09892459	-0.605519767	3.957207142	-4.848747193	6.62659E-05	0.032536877
cg19961800	-0.82228698	-1.192606785	-4.848558282	6.62972E-05	0.032536877
cg16284553	-0.598360583	-0.125470664	-4.848157622	6.63636E-05	0.032536877
cg15806588	-0.606177598	0.426717564	-4.848020422	6.63863E-05	0.032536877
cg21069700	-0.713960395	2.344924862	-4.848017719	6.63868E-05	0.032536877
cg11628864	-0.762142033	2.766869238	-4.847955895	6.6397E-05	0.032536877
cg13115910	-0.625843352	0.049250268	-4.84784023	6.64162E-05	0.032536877
cg19911006	-0.580295639	0.959324486	-4.847528787	6.64679E-05	0.032536877
cg14709069	-0.696022518	2.766248908	-4.847338622	6.64995E-05	0.032536877
cg19647197	-0.623972513	3.437276868	-4.847057102	6.65463E-05	0.032536877
cg06171671	-0.82889337	2.739706608	-4.84674451	6.65983E-05	0.032536877
cg26122676	-0.570321508	3.217619138	-4.846126283	6.67012E-05	0.032536877
cg05576754	-0.498406277	3.757689746	-4.846118337	6.67025E-05	0.032536877
cg01009798	-0.907497667	3.336697943	-4.845018946	6.6886E-05	0.032536877
cg17956903	-0.986786032	0.382468503	-4.844878944	6.69094E-05	0.032536877
cg10919488	-0.669011788	3.590242614	-4.844682709	6.69422E-05	0.032536877
cg02953769	-0.718972931	2.7218966	-4.844510244	6.69711E-05	0.032536877
cg13293266	-0.533810585	0.784789398	-4.844378389	6.69931E-05	0.032536877
cg02063727	-0.819705254	1.369470099	-4.844261475	6.70127E-05	0.032536877
cg16769113	-0.602880145	3.284678968	-4.844131904	6.70344E-05	0.032536877
cg01055868	-0.769780558	1.139810974	-4.84380571	6.7089E-05	0.032536877
ch.4.698479R	-0.732500109	-2.825145951	-4.843781451	6.70931E-05	0.032536877
cg04933490	-0.632120796	-1.414547407	-4.843548075	6.71322E-05	0.032536877
cg19947357	-0.705401749	2.445485523	-4.843456886	6.71475E-05	0.032536877
cg08125539	-0.629956659	2.950017441	-4.84336484	6.7163E-05	0.032536877
cg27660807	-0.641360967	0.215757929	-4.843318945	6.71707E-05	0.032536877
cg16070908	-0.474313246	3.615346656	-4.84324734	6.71827E-05	0.032536877
cg10307762	-0.616213048	3.059202633	-4.843234117	6.71849E-05	0.032536877

cg03724891	-0.652319973	3.398887767	-4.842727399	6.727E-05	0.032536877
cg21564289	-0.517719218	3.169018474	-4.842098611	6.73758E-05	0.032536877
cg08513039	-1.01986935	1.771380678	-4.842030605	6.73872E-05	0.032536877
cg08449717	-0.75552899	2.052050967	-4.841849837	6.74177E-05	0.032536877
cg04359602	-0.801512537	0.065243252	-4.841683187	6.74457E-05	0.032536877
cg08985601	-0.589181363	-0.233139056	-4.841599193	6.74599E-05	0.032536877
cg03856773	-0.365813917	4.188577884	-4.84135467	6.75011E-05	0.032536877
cg13408606	-0.706725108	3.441944723	-4.841013795	6.75586E-05	0.032536877
cg08192831	-0.634648095	0.522516537	-4.840875413	6.7582E-05	0.032536877
cg14928149	-0.521361868	3.805539394	-4.840852305	6.75859E-05	0.032536877
cg13521687	0.612219966	2.161672627	4.840767457	6.76002E-05	0.032536877
cg13956518	-0.790814841	2.826488954	-4.840492337	6.76467E-05	0.032536877
cg03056087	-0.699663709	2.887366997	-4.840422113	6.76586E-05	0.032536877
cg19106809	-0.546408746	0.301265698	-4.840320415	6.76758E-05	0.032536877
cg02989450	-0.734104237	3.126372285	-4.839817157	6.77609E-05	0.032536877
cg16156662	-0.599618069	3.427612084	-4.839673049	6.77853E-05	0.032536877
cg22890825	-0.464594748	-0.161972891	-4.839534649	6.78088E-05	0.032536877
cg02589899	-0.603148765	0.370721403	-4.839451437	6.78229E-05	0.032536877
cg22103319	-0.517530398	3.87339366	-4.839266896	6.78541E-05	0.032536877
cg24963454	-0.626199153	3.230851777	-4.839167406	6.7871E-05	0.032536877
cg25470023	-0.608708025	3.367817704	-4.838806539	6.79322E-05	0.032536877
cg11145461	-0.849358123	-2.008882734	-4.838774645	6.79376E-05	0.032536877
cg07988609	-0.980626289	0.784370574	-4.83857204	6.7972E-05	0.032536877
cg01757942	-0.956343101	2.374172343	-4.838264582	6.80243E-05	0.032536877
cg21243146	-0.650790427	2.874183902	-4.838037913	6.80628E-05	0.032536877
cg02386178	-0.71060726	2.777056345	-4.837995471	6.807E-05	0.032536877
cg09916228	-0.562946334	2.864322962	-4.837230877	6.82002E-05	0.032539807
cg15099060	-0.633307114	2.785515217	-4.837102592	6.82221E-05	0.032539807
cg06582460	-0.659234083	3.465826936	-4.836995994	6.82402E-05	0.032539807
cg24482389	-0.665122153	2.719665969	-4.836880628	6.82599E-05	0.032539807
cg21646823	-0.703907273	3.151323689	-4.836525322	6.83205E-05	0.032546806
cg08910658	-0.767877554	3.154493428	-4.835459101	6.85028E-05	0.032598351
cg06581176	-0.655358012	2.814353104	-4.835354036	6.85208E-05	0.032598351
cg17707104	-0.855983552	3.139877972	-4.834919117	6.85953E-05	0.032607055
cg07479492	-0.755100881	2.482263167	-4.834709985	6.86311E-05	0.032607055
cg27419360	-0.807479407	1.051580214	-4.83408844	6.87378E-05	0.032635846
cg11838818	-0.591453373	2.070447184	-4.8336952	6.88054E-05	0.032643173
cg11742981	-0.701614619	3.018569564	-4.833238341	6.8884E-05	0.032643173
cg20854570	-0.519695657	3.265302284	-4.832799269	6.89596E-05	0.032643173
cg15208491	-0.626612093	2.765374924	-4.83265911	6.89837E-05	0.032643173
cg05035406	-0.605573294	3.813857603	-4.832154858	6.90707E-05	0.032643173
cg19759051	-0.499198931	-0.645924152	-4.832068959	6.90855E-05	0.032643173
cg13668207	-0.596212191	-0.848359484	-4.83168828	6.91513E-05	0.032643173
cg17291213	-0.859705169	2.59973239	-4.83124654	6.92276E-05	0.032643173
cg14538256	-0.466107935	3.986227898	-4.831220439	6.92322E-05	0.032643173
cg19486070	-0.660955441	-1.798992125	-4.831015594	6.92676E-05	0.032643173
cg19750031	-0.580329491	3.479757574	-4.830856845	6.92951E-05	0.032643173

cg14640555	-0.428050929	-1.027952431	-4.830370471	6.93794E-05	0.032643173
cg20594304	-0.563230397	-1.72800388	-4.82991655	6.94581E-05	0.032643173
cg27467591	-0.968889924	2.657537062	-4.829480277	6.95339E-05	0.032643173
cg10845890	-0.600336444	-1.42469348	-4.829418855	6.95445E-05	0.032643173
cg01605931	-0.507363432	0.05782497	-4.829303814	6.95645E-05	0.032643173
cg20695529	-0.442533957	3.507691685	-4.82910423	6.95992E-05	0.032643173
cg24615926	-0.524344486	4.059840597	-4.828765261	6.96582E-05	0.032643173
cg18582408	-0.685176684	3.01073875	-4.828695033	6.96704E-05	0.032643173
cg10056799	-1.054557671	0.792376117	-4.828669658	6.96748E-05	0.032643173
cg16477662	-0.505966492	-0.134080169	-4.827900115	6.9809E-05	0.032681843
cg06636080	-0.507323347	3.448699939	-4.82766689	6.98497E-05	0.032681843
cg18954633	-0.525847849	-0.385399527	-4.827182135	6.99343E-05	0.032699858
cg18687492	-0.74623556	3.812554604	-4.826617982	7.0033E-05	0.032715414
cg22548804	-0.788127424	2.539078423	-4.826450048	7.00624E-05	0.032715414
cg26355640	-0.59994826	2.89923132	-4.82620018	7.01061E-05	0.032715414
cg22594071	-0.613284157	1.555569657	-4.825919441	7.01553E-05	0.032716821
cg13697735	-0.683274635	-1.339749174	-4.825484009	7.02317E-05	0.032730893
cg09971864	-0.54850046	3.416317479	-4.824782739	7.03549E-05	0.032764758
cg13101948	-0.596343419	1.902968379	-4.824504584	7.04038E-05	0.032764758
cg05831237	-1.064315932	2.684895113	-4.824171676	7.04624E-05	0.032764758
cg15824303	-0.679323252	1.718395354	-4.823511971	7.05787E-05	0.032764758
cg01611522	-0.603490083	0.424481832	-4.82324817	7.06252E-05	0.032764758
cg07390411	-0.671016482	2.060067954	-4.822971309	7.06741E-05	0.032764758
cg27464514	-0.88946016	2.676993494	-4.822591362	7.07413E-05	0.032764758
cg09230454	-0.595523494	2.619597451	-4.822566001	7.07457E-05	0.032764758
cg16891213	-0.644687073	2.835710711	-4.822483618	7.07603E-05	0.032764758
cg10556222	-0.755234688	3.187285457	-4.822446229	7.07669E-05	0.032764758
cg21859880	-0.533605117	0.894715453	-4.821832634	7.08755E-05	0.032788374
cg10476989	-0.572074921	3.841569524	-4.820997607	7.10236E-05	0.032788374
cg10668814	-0.654440399	-0.378404944	-4.820951513	7.10318E-05	0.032788374
cg02999082	-0.422212908	3.965591381	-4.820862544	7.10476E-05	0.032788374
cg17050062	-0.803394329	1.983718231	-4.820076764	7.11872E-05	0.032788374
cg07996037	-0.507199761	0.211645136	-4.819772739	7.12413E-05	0.032788374
cg16685239	-0.559341658	3.856937641	-4.81955663	7.12798E-05	0.032788374
cg12244668	-0.493604813	0.452353532	-4.819058578	7.13686E-05	0.032788374
cg20707969	-0.494412499	0.614533696	-4.818401601	7.14859E-05	0.032788374
cg20244202	-0.523438985	-1.403024205	-4.817757971	7.16009E-05	0.032788374
cg11030620	-0.644766202	3.34598108	-4.817700161	7.16113E-05	0.032788374
cg22224736	-0.57058005	3.363124164	-4.817607181	7.16279E-05	0.032788374
cg21820652	-0.443587812	-0.868294173	-4.817392544	7.16664E-05	0.032788374
cg11956562	-0.749617191	2.970294905	-4.817124804	7.17143E-05	0.032788374
cg03424137	-0.59090466	3.448887751	-4.817076074	7.17231E-05	0.032788374
cg04226884	-0.466276412	-0.470012275	-4.816902073	7.17543E-05	0.032788374
cg21556182	-0.543884192	-0.049441595	-4.81670711	7.17892E-05	0.032788374
cg20136009	-0.783195067	2.660800237	-4.816181679	7.18836E-05	0.032788374
cg17711463	-0.760364212	1.653263029	-4.815961843	7.19231E-05	0.032788374
cg21498120	-0.932823012	1.258412752	-4.815771368	7.19573E-05	0.032788374

cg04083226	0.633395605	3.460107067	4.815527656	7.20012E-05	0.032788374
cg18498096	-0.59802527	-1.112895205	-4.81529749	7.20426E-05	0.032788374
cg26963320	-0.483376681	3.790421718	-4.815216678	7.20572E-05	0.032788374
cg06814136	-0.827511154	3.084205847	-4.815042541	7.20885E-05	0.032788374
cg10524582	-0.678214055	2.811945878	-4.814534138	7.21802E-05	0.032788374
cg24320018	-0.636731262	4.03558587	-4.814436365	7.21978E-05	0.032788374
cg03249171	-0.620569462	3.208195527	-4.814348746	7.22136E-05	0.032788374
cg07424435	-0.652381911	0.77304102	-4.814240812	7.22331E-05	0.032788374
cg15223494	-0.696365171	2.938377	-4.813660732	7.23379E-05	0.032788374
cg26441893	-0.721005402	3.143994336	-4.813441095	7.23777E-05	0.032788374
cg12098658	-0.468910431	-1.325340923	-4.813179595	7.2425E-05	0.032788374
cg25195373	-0.65810622	2.381250451	-4.812547363	7.25395E-05	0.032788374
cg10576242	-0.571504676	2.672413384	-4.812391639	7.25678E-05	0.032788374
cg02784486	-0.615493267	3.097507571	-4.812326395	7.25796E-05	0.032788374
cg21636989	-0.541824222	2.570238762	-4.812322361	7.25803E-05	0.032788374
cg18862260	-0.691224671	0.901888308	-4.812261993	7.25913E-05	0.032788374
cg03959186	-0.748206605	-1.814932827	-4.812193329	7.26037E-05	0.032788374
cg12399793	-0.7888205	-2.115987479	-4.811907221	7.26557E-05	0.032788374
cg15837109	-0.479023126	0.138318457	-4.811835039	7.26688E-05	0.032788374
cg23084441	-0.636155786	2.622511157	-4.811779072	7.26789E-05	0.032788374
cg04408104	-0.485397323	-1.297631886	-4.811344858	7.27579E-05	0.032788374
cg27454408	-0.828018955	2.435198823	-4.811295123	7.27669E-05	0.032788374
cg10103036	-0.786885172	2.639565683	-4.810257638	7.29559E-05	0.032788374
cg09026137	-0.834010472	1.295583271	-4.810122776	7.29805E-05	0.032788374
cg25271413	-0.651676613	3.045326444	-4.809761317	7.30464E-05	0.032788374
cg14364460	-0.487980356	-1.042311863	-4.809663181	7.30643E-05	0.032788374
cg15989318	-0.433115632	-0.490417714	-4.808307958	7.33123E-05	0.032788374
cg16222773	-0.634148977	3.247100404	-4.808286235	7.33162E-05	0.032788374
cg21621344	-0.495634216	4.156279784	-4.807922479	7.33829E-05	0.032788374
cg22482658	-0.453182197	3.682125357	-4.807229257	7.35102E-05	0.032788374
cg12235788	-0.628550042	0.110888803	-4.806808269	7.35876E-05	0.032788374
cg20297468	-0.608208018	2.843540813	-4.806738295	7.36005E-05	0.032788374
cg21348526	-0.646172979	3.237579274	-4.806614388	7.36233E-05	0.032788374
cg07908679	-0.568501639	-0.452859604	-4.806267327	7.36872E-05	0.032788374
cg11028600	-0.579996821	2.631679249	-4.806238522	7.36925E-05	0.032788374
cg22530449	-0.760008255	3.044259907	-4.805997806	7.37368E-05	0.032788374
cg02907950	-0.61209291	3.172999342	-4.805952631	7.37452E-05	0.032788374
cg01647384	-0.599488411	2.924338057	-4.805900097	7.37548E-05	0.032788374
cg08642671	-0.770417401	2.770414471	-4.805331154	7.38598E-05	0.032788374
cg07278329	-0.83141142	2.188667089	-4.805107335	7.39011E-05	0.032788374
cg17138880	-0.775458033	3.038113251	-4.805064265	7.39091E-05	0.032788374
cg00970757	-0.485165034	3.584013698	-4.804753773	7.39665E-05	0.032788374
cg27330561	-0.543549228	3.594107544	-4.804658076	7.39842E-05	0.032788374
cg11160572	-0.510112596	0.362505274	-4.804259082	7.4058E-05	0.032788374
cg08919084	-0.480724668	-1.421979303	-4.804114955	7.40847E-05	0.032788374
cg18455490	-0.529811753	-2.280702533	-4.804080654	7.4091E-05	0.032788374
cg18395354	-0.706249754	0.200622807	-4.803902839	7.4124E-05	0.032788374

cg19159488	-0.535407132	3.269950255	-4.803760989	7.41503E-05	0.032788374
cg20995142	-0.523528889	2.666338293	-4.803495728	7.41995E-05	0.032788374
cg07449080	-0.518917308	3.621766531	-4.803111334	7.42708E-05	0.032788374
cg01566127	-0.638422588	-3.824330784	-4.803104516	7.42721E-05	0.032788374
cg04689876	-0.553385216	3.539827032	-4.802741965	7.43394E-05	0.032788374
cg03018162	-0.56714935	3.982672919	-4.802718613	7.43437E-05	0.032788374
cg23502924	-0.489288461	-0.099761268	-4.802573152	7.43708E-05	0.032788374
cg17411455	-0.509734736	-0.938685212	-4.802546323	7.43758E-05	0.032788374
cg07160402	-0.690779564	2.98225089	-4.802464215	7.4391E-05	0.032788374
cg10108729	0.477996723	2.690960813	4.801995424	7.44783E-05	0.032788374
cg06991584	-0.449407174	3.848315331	-4.801034191	7.46574E-05	0.032788374
cg09337948	-0.673468255	3.069182702	-4.800791453	7.47028E-05	0.032788374
cg02946483	0.528941287	2.709621915	4.800676124	7.47243E-05	0.032788374
cg05198178	-0.507274621	2.920316437	-4.800667509	7.47259E-05	0.032788374
cg26009180	-0.577647623	3.019391926	-4.800534704	7.47507E-05	0.032788374
cg20693840	-0.822528571	2.252665003	-4.800289566	7.47965E-05	0.032788374
cg13390441	-0.528352553	-0.838959513	-4.798477795	7.51361E-05	0.032788374
cg13871684	-0.906508545	1.813780785	-4.79846198	7.5139E-05	0.032788374
cg04794720	-0.79245471	-0.17724815	-4.798201899	7.51879E-05	0.032788374
cg02449225	-0.666271987	-0.442924635	-4.798118791	7.52035E-05	0.032788374
cg07983930	-0.42204664	-0.658531334	-4.797685994	7.52849E-05	0.032788374
cg15013199	0.570024031	2.454342892	4.79744471	7.53304E-05	0.032788374
cg06008302	-0.681515406	3.360667231	-4.797340251	7.535E-05	0.032788374
cg22034627	-0.557618169	-1.105563061	-4.797329554	7.53521E-05	0.032788374
cg22819824	-0.647426766	2.917208402	-4.797188853	7.53786E-05	0.032788374
cg05626921	-0.477608408	-0.015777993	-4.797174023	7.53814E-05	0.032788374
cg16744911	-0.463130065	-0.343977085	-4.797028306	7.54088E-05	0.032788374
cg20363751	-0.432535535	4.057722763	-4.796786396	7.54544E-05	0.032788374
cg01446171	-1.010763248	1.938518366	-4.796715361	7.54679E-05	0.032788374
cg01208787	-0.928278675	1.494705166	-4.796690908	7.54725E-05	0.032788374
cg24276974	-0.674622158	3.631084957	-4.796217766	7.55618E-05	0.032788374
cg17783968	-0.647913624	2.025018524	-4.795797053	7.56413E-05	0.032788374
cg16456371	-0.573009816	3.47628151	-4.795664175	7.56664E-05	0.032788374
cg21820890	-0.512272535	2.568406309	-4.795571308	7.5684E-05	0.032788374
cg11349552	-0.653434833	3.231709029	-4.795414915	7.57136E-05	0.032788374
cg05904364	-1.052232535	-2.853004806	-4.795317201	7.57321E-05	0.032788374
cg14484681	-0.986754787	1.700177165	-4.795074316	7.57781E-05	0.032788374
cg08203162	-0.532157106	3.658410678	-4.794987861	7.57945E-05	0.032788374
cg13011928	-0.652371522	2.918112802	-4.794981912	7.57956E-05	0.032788374
cg01213651	-0.665408151	3.785928274	-4.79490244	7.58107E-05	0.032788374
cg21046954	-1.062613918	1.849527463	-4.794618926	7.58644E-05	0.032788374
cg03474172	-0.555226247	-0.507001011	-4.794567501	7.58742E-05	0.032788374
cg18121434	-0.629462972	-2.172781101	-4.794381821	7.59094E-05	0.032788374
cg04808930	-0.852374737	1.80985203	-4.793750887	7.60292E-05	0.032803674
cg23354075	-0.588944272	3.555955551	-4.793434822	7.60893E-05	0.032803674
cg19961545	-0.525109524	-0.592603486	-4.793210332	7.61321E-05	0.032803674
cg07527547	-0.483458085	3.957445608	-4.792979511	7.6176E-05	0.032803674

cg17212902	-0.601666858	3.152651025	-4.792977587	7.61764E-05	0.032803674
cg04041046	-0.463700411	3.962972746	-4.792630004	7.62426E-05	0.032812246
cg22786836	-0.423545869	3.638088641	-4.791451271	7.64676E-05	0.032889105
cg10222682	-0.577092045	2.65872937	-4.790619112	7.66269E-05	0.032894198
cg06341047	-0.573543946	0.247157019	-4.790396796	7.66695E-05	0.032894198
cg09450120	-0.755572475	2.28161531	-4.790386208	7.66715E-05	0.032894198
cg26458142	-0.613202721	3.120142414	-4.790347857	7.66789E-05	0.032894198
cg17718545	-0.598232873	0.607909482	-4.789893789	7.6766E-05	0.032894198
cg17012555	-0.618138804	3.233967534	-4.789291054	7.68817E-05	0.032894198
cg09964207	-0.607315729	2.497688852	-4.789193934	7.69004E-05	0.032894198
cg09034580	-0.610430478	2.664751668	-4.789137442	7.69113E-05	0.032894198
cg00712146	-0.46371076	3.333824728	-4.789029414	7.6932E-05	0.032894198
cg25178497	-0.508392069	4.251316719	-4.788775235	7.69809E-05	0.032894198
cg11761443	-0.558074424	3.576200389	-4.787727602	7.71828E-05	0.032894198
cg16326512	-0.507186654	4.227813807	-4.787646607	7.71985E-05	0.032894198
cg22420623	-0.467718123	4.264580899	-4.787473355	7.72319E-05	0.032894198
cg06291988	-0.483422489	3.52674102	-4.787377562	7.72504E-05	0.032894198
cg05210373	-0.464589881	-0.794197289	-4.787353358	7.72551E-05	0.032894198
cg26952136	-0.65182138	0.76813028	-4.787339353	7.72578E-05	0.032894198
cg21986679	-0.730662789	0.570841982	-4.787282122	7.72689E-05	0.032894198
cg25083484	-0.50639485	4.009078122	-4.78661045	7.73987E-05	0.032912295
cg05388137	-0.506668345	2.992077252	-4.786581727	7.74043E-05	0.032912295
cg03674311	-0.473709379	4.050206855	-4.786265069	7.74656E-05	0.032918604
cg17037640	-0.596808647	3.299977545	-4.785728215	7.75697E-05	0.032925757
cg09142090	-0.727166439	2.432815618	-4.785698611	7.75754E-05	0.032925757
cg05171643	-0.514196615	-1.980003524	-4.78517156	7.76777E-05	0.032934059
cg13733984	-0.424324467	2.982303448	-4.785118803	7.76879E-05	0.032934059
cg12433196	-0.86805185	0.061840724	-4.783996125	7.79063E-05	0.032964541
cg09484593	-0.564582342	3.381569107	-4.783855779	7.79337E-05	0.032964541
cg00013301	-0.681441486	4.138815308	-4.783534949	7.79962E-05	0.032964541
cg05891614	-0.550165325	2.975864729	-4.783365299	7.80293E-05	0.032964541
cg23806708	-0.62610668	1.833110645	-4.783120157	7.80771E-05	0.032964541
cg11760320	-0.762209776	2.692566137	-4.782851451	7.81296E-05	0.032964541
cg13733394	-0.488993398	3.619681504	-4.782534095	7.81916E-05	0.032964541
cg17056988	0.949823121	2.047149086	4.782197655	7.82574E-05	0.032964541
cg24980696	-0.500717005	3.728134038	-4.781708729	7.83532E-05	0.032964541
cg25782003	0.564798834	-4.364948994	4.781594076	7.83756E-05	0.032964541
cg02662823	-0.687624768	-0.875958996	-4.781099452	7.84726E-05	0.032964541
cg21408197	-0.568826574	-0.773185303	-4.781096695	7.84732E-05	0.032964541
cg26754071	-0.478759632	3.17311118	-4.780667875	7.85574E-05	0.032964541
cg14933010	-0.611568398	3.308209163	-4.780591645	7.85723E-05	0.032964541
cg22220380	-0.633618698	3.271852428	-4.78057538	7.85755E-05	0.032964541
cg16144590	-0.574184099	4.365574172	-4.780525739	7.85853E-05	0.032964541
cg21099163	-0.695533308	3.695978578	-4.780256332	7.86383E-05	0.032964541
cg11751613	-0.576474642	0.494979648	-4.780030042	7.86828E-05	0.032964541
cg24914219	-0.701262679	2.894426228	-4.7795623	7.87748E-05	0.032964541
cg20731677	-0.791933847	3.254972339	-4.779437538	7.87994E-05	0.032964541

cg25330016	-0.467461284	-0.711815696	-4.779064266	7.8873E-05	0.032964541
cg08334575	-0.435400527	0.846806305	-4.778913998	7.89026E-05	0.032964541
cg21951459	-0.405074079	3.654326373	-4.778754703	7.89341E-05	0.032964541
cg16374225	-0.527067388	3.191575201	-4.778751535	7.89347E-05	0.032964541
cg10084484	-0.490592398	0.320370557	-4.778743261	7.89363E-05	0.032964541
cg22558265	-0.540425943	3.709442635	-4.778451318	7.8994E-05	0.032964541
cg07201717	-0.5435389	-0.697830728	-4.777994611	7.90843E-05	0.032964541
cg21930788	-0.683613711	-0.149413625	-4.777677176	7.91471E-05	0.032964541
cg10171557	-0.487791884	4.026885964	-4.777144093	7.92526E-05	0.032964541
cg00305774	-0.577490507	-0.036235802	-4.776870897	7.93068E-05	0.032964541
cg05764238	-0.593669689	-0.581548343	-4.776634044	7.93538E-05	0.032964541
cg03885270	-0.49896099	-0.988276253	-4.776453118	7.93897E-05	0.032964541
cg19927020	-0.505448845	3.454290558	-4.775929622	7.94937E-05	0.032964541
cg02283964	-0.669391675	3.103079225	-4.775878953	7.95037E-05	0.032964541
cg01839898	-0.405795901	-0.865462228	-4.775851619	7.95092E-05	0.032964541
cg05355766	-0.616355261	0.450556722	-4.77526389	7.96261E-05	0.032964541
cg06729532	-0.631779375	-1.107039755	-4.775053333	7.96681E-05	0.032964541
cg01112125	-0.501517517	3.770948566	-4.77505108	7.96685E-05	0.032964541
cg13796226	-0.688444426	3.366502361	-4.775046856	7.96694E-05	0.032964541
cg21037057	-0.718080003	3.216791432	-4.774925952	7.96934E-05	0.032964541
cg17295878	-0.615480006	-0.475807048	-4.774841211	7.97103E-05	0.032964541
cg00549398	-0.498817916	0.292473172	-4.774821277	7.97143E-05	0.032964541
cg06458741	-0.788821532	1.933212792	-4.774318124	7.98147E-05	0.032986788
cg07309120	-0.720752778	2.783531098	-4.77323118	8.00319E-05	0.033031701
cg13856236	-0.693595108	0.627612379	-4.773181767	8.00418E-05	0.033031701
cg07630825	-0.747329335	2.942602832	-4.773074718	8.00632E-05	0.033031701
cg11979138	-0.510400124	3.206263966	-4.772730196	8.01322E-05	0.033040929
cg20316986	-0.700736995	2.306321217	-4.771865133	8.03058E-05	0.033052555
cg19786648	-0.485153517	0.170523997	-4.771387152	8.04018E-05	0.033052555
cg08613981	-0.640427956	2.367177775	-4.770997781	8.04802E-05	0.033052555
cg25182665	-0.680931206	2.943114616	-4.770681654	8.05438E-05	0.033052555
cg01336845	-0.524074864	-1.143056991	-4.770620108	8.05562E-05	0.033052555
cg09610332	-0.591419861	2.84264558	-4.770184746	8.0644E-05	0.033052555
cg09313841	-0.96179303	2.544376741	-4.769874475	8.07066E-05	0.033052555
cg07123625	-0.628114528	3.462733033	-4.76886348	8.09109E-05	0.033052555
cg21415292	-0.445360334	-0.624852136	-4.768644582	8.09552E-05	0.033052555
cg22256082	-0.602428048	2.443333114	-4.768562522	8.09718E-05	0.033052555
cg09203047	-0.862365723	1.513976844	-4.768322493	8.10204E-05	0.033052555
cg06465756	-0.539385464	3.787127965	-4.768216348	8.10419E-05	0.033052555
cg18542842	-0.584388198	3.190523014	-4.768066418	8.10723E-05	0.033052555
cg15691016	-0.573353316	3.846741698	-4.768033363	8.1079E-05	0.033052555
cg20315554	-0.537409273	3.222718071	-4.767839656	8.11183E-05	0.033052555
cg07063336	-0.538686222	3.074547649	-4.767697634	8.11471E-05	0.033052555
cg25311335	-0.868698462	0.03393258	-4.767610535	8.11648E-05	0.033052555
cg16935646	-0.455532132	4.045514897	-4.767540519	8.1179E-05	0.033052555
cg12110537	-0.512926313	3.633540561	-4.767269202	8.12341E-05	0.033052555
cg06725695	-0.572113689	3.357166935	-4.766966309	8.12957E-05	0.033052555

cg07696502	-0.576163693	-0.5908846	-4.766925415	8.1304E-05	0.033052555
cg09863133	-0.633540669	2.828732831	-4.766702549	8.13493E-05	0.033052555
cg11607910	-0.579040455	3.581796869	-4.766549949	8.13804E-05	0.033052555
cg02258230	-0.460986457	3.869302148	-4.766376596	8.14157E-05	0.033052555
cg23385218	0.707050009	-3.346696207	4.765960977	8.15003E-05	0.033052555
cg25556230	-0.611849118	2.650426531	-4.765912429	8.15102E-05	0.033052555
cg03418370	-0.541059566	3.523146922	-4.765777296	8.15378E-05	0.033052555
cg23651872	-0.630303202	-1.417210278	-4.76553535	8.15871E-05	0.033052555
cg03629516	-0.891174315	1.708228649	-4.764840055	8.17291E-05	0.033052555
cg27533767	-0.392572587	0.050066297	-4.764528778	8.17928E-05	0.033052555
cg00795416	-0.614766824	4.089336204	-4.764272335	8.18452E-05	0.033052555
cg10012517	-0.788597408	-2.29526577	-4.763898942	8.19217E-05	0.033052555
cg10141830	-0.480474094	3.296872673	-4.763715494	8.19593E-05	0.033052555
cg15314164	-0.750361411	3.189878956	-4.763657085	8.19713E-05	0.033052555
cg06615754	0.656865869	-2.196824389	4.763471679	8.20093E-05	0.033052555
cg26420566	-0.520105199	4.085462798	-4.763292759	8.2046E-05	0.033052555
cg03202213	-0.382365138	3.909014774	-4.763218503	8.20612E-05	0.033052555
cg21524784	-0.568480766	2.640554131	-4.762946308	8.21171E-05	0.033052555
cg16662043	-1.016575583	0.966831705	-4.762774833	8.21524E-05	0.033052555
cg18327385	-0.520077498	1.2150525	-4.762641393	8.21798E-05	0.033052555
cg12849484	-0.65383675	3.539033599	-4.762527908	8.22031E-05	0.033052555
cg02612766	-0.494726387	-1.112956636	-4.762316798	8.22465E-05	0.033052555
cg12308577	-0.635670986	1.257146714	-4.762195824	8.22714E-05	0.033052555
cg20479009	-0.72517969	-0.405980403	-4.761817505	8.23493E-05	0.033052555
cg21157232	-0.649005792	3.033227495	-4.761514164	8.24118E-05	0.033052555
cg01321839	-0.586989627	3.718805194	-4.761486875	8.24174E-05	0.033052555
cg18887228	-0.533446365	4.189870674	-4.76124974	8.24663E-05	0.033052555
cg17361885	0.594915282	1.475729792	4.761177744	8.24812E-05	0.033052555
cg26187477	-0.47543924	-0.363824927	-4.761160383	8.24847E-05	0.033052555
cg14617524	-0.385805462	-0.695335185	-4.760948464	8.25285E-05	0.033052555
cg07869457	-0.489587775	3.43343381	-4.760892393	8.25401E-05	0.033052555
cg01211852	-0.755262688	1.944250068	-4.76063375	8.25935E-05	0.033055258
cg13010299	-0.568601574	-4.194052169	-4.76011702	8.27003E-05	0.033079317
cg00058291	-0.483302625	0.322219549	-4.759345645	8.286E-05	0.033112541
cg11875744	-0.43478952	3.462148395	-4.758939199	8.29443E-05	0.033112541
cg05789595	-0.550288961	-0.103184792	-4.758905604	8.29512E-05	0.033112541
cg09381333	-0.821336558	2.331441833	-4.758813636	8.29703E-05	0.033112541
cg09470758	-0.497359393	4.087204445	-4.758434946	8.30489E-05	0.033123559
cg06253910	-0.523442196	3.32946228	-4.757553306	8.32323E-05	0.033123559
cg09989714	-0.560911501	3.078411632	-4.757266933	8.32919E-05	0.033123559
cg08497894	-0.644205742	2.704230276	-4.757069347	8.33331E-05	0.033123559
cg10633103	2.20021319	3.247437209	4.756880242	8.33725E-05	0.033123559
cg23914535	-1.019509456	2.120261901	-4.756820259	8.3385E-05	0.033123559
cg03075287	-0.720900117	0.164249532	-4.756594579	8.34321E-05	0.033123559
cg11354933	-0.602333071	2.802912257	-4.756284701	8.34968E-05	0.033123559
cg02505933	-0.549238602	3.575814963	-4.75610654	8.3534E-05	0.033123559
cg15443266	-1.057241933	2.240284261	-4.756097095	8.3536E-05	0.033123559

cg25728306	-0.590837429	3.431022291	-4.756026635	8.35507E-05	0.033123559
cg01008365	-0.534657925	3.490894325	-4.755986808	8.3559E-05	0.033123559
cg05956531	-0.751635433	3.281187239	-4.754856213	8.37957E-05	0.033189115
cg21488113	0.823785863	-3.308648371	4.754587981	8.38519E-05	0.033189115
cg13443503	-0.465752282	3.965530058	-4.754525722	8.3865E-05	0.033189115
cg07339310	-0.752748465	2.45114497	-4.754219863	8.39291E-05	0.03319597
cg02454350	-0.694417003	3.032415763	-4.75337962	8.41057E-05	0.033247248
cg22071015	-0.478122648	-0.725215919	-4.752610008	8.42678E-05	0.033292734
cg21808433	-1.139125958	2.041121622	-4.752017163	8.43928E-05	0.033298586
cg01962471	-0.559091366	-0.313971914	-4.751947966	8.44075E-05	0.033298586
cg01176915	-0.639769782	3.922657331	-4.751470191	8.45084E-05	0.033298586
cg11325800	-0.66721727	0.675236606	-4.751449779	8.45127E-05	0.033298586
cg03977394	0.863307602	-2.902485687	4.751426459	8.45176E-05	0.033298586
cg08426604	-0.765476239	2.117769335	-4.751199884	8.45656E-05	0.033298943
cg10727879	-0.504502937	3.891704707	-4.750904799	8.4628E-05	0.033305018
cg12134559	-0.521376637	0.271039978	-4.750571186	8.46986E-05	0.033314315
cg14847947	-0.571242206	0.506313539	-4.749979856	8.4824E-05	0.033345113
cg05528266	-0.547630527	3.86483995	-4.749553817	8.49145E-05	0.033362154
cg06015378	-0.517560777	4.296006045	-4.749054278	8.50206E-05	0.033385352
cg20765594	-0.404062688	4.147612778	-4.748465958	8.51459E-05	0.033415738
cg17221235	-0.426893054	0.859103572	-4.747932916	8.52595E-05	0.033415738
cg20277356	-0.807756951	-0.065895977	-4.747875928	8.52716E-05	0.033415738
cg17792742	-0.453715537	3.563156184	-4.747805185	8.52867E-05	0.033415738
cg18530920	-0.634417043	0.838337049	-4.747574308	8.5336E-05	0.033416559
cg08021815	-0.445909172	4.368098622	-4.747117266	8.54336E-05	0.033421475
cg26098816	-0.632702066	3.084464758	-4.747073783	8.54429E-05	0.033421475
cg01672987	-0.716390775	1.741253858	-4.746481754	8.55695E-05	0.033452536
cg05112969	-0.574440608	3.74099807	-4.746093128	8.56527E-05	0.033454564
cg17362026	-0.60280695	2.4729298	-4.745917753	8.56903E-05	0.033454564
cg11619648	-1.049612276	0.769936481	-4.745711972	8.57345E-05	0.033454564
cg02005589	-0.674039238	2.602586599	-4.74552121	8.57754E-05	0.033454564
cg22997193	-0.510621521	3.952787974	-4.745219334	8.58402E-05	0.033454564
cg16999359	-0.717852196	2.208646629	-4.745136016	8.58581E-05	0.033454564
cg16425946	0.742082964	2.643133901	4.744767371	8.59373E-05	0.033454777
cg14838738	-0.531973312	3.432775938	-4.744427277	8.60104E-05	0.033454777
cg14856698	-0.714982457	-3.359415724	-4.744207664	8.60577E-05	0.033454777
cg20921240	-0.566921305	-0.682375817	-4.74411617	8.60774E-05	0.033454777
cg16349846	-0.621273289	3.518907327	-4.743974363	8.61079E-05	0.033454777
cg19932853	-0.551953638	3.7918107	-4.743711555	8.61646E-05	0.033454777
cg00805973	-0.594795333	2.906283281	-4.743597254	8.61892E-05	0.033454777
cg27633458	-0.564639757	-0.290185925	-4.742706339	8.63815E-05	0.033476104
cg02048501	-0.596930584	3.631544103	-4.742665734	8.63903E-05	0.033476104
cg19219065	-0.539282474	3.549828277	-4.742464477	8.64338E-05	0.033476104
cg01202671	-0.808442934	2.680318806	-4.742260878	8.64778E-05	0.033476104
cg07958926	0.571013011	-4.173483388	4.742129387	8.65063E-05	0.033476104
cg13876366	-0.605243549	3.405229717	-4.742030442	8.65277E-05	0.033476104
cg19364765	-0.55046315	3.232962148	-4.741167983	8.67146E-05	0.033506733

cg12762851	-0.483801219	3.38870316	-4.741083333	8.6733E-05	0.033506733
cg27237834	-0.61312029	2.546785353	-4.740749706	8.68054E-05	0.033506733
cg10826999	-0.509670561	4.421042636	-4.74053315	8.68524E-05	0.033506733
cg03424643	-0.748905039	2.279874946	-4.740161054	8.69333E-05	0.033506733
cg16946833	-0.678263551	2.916705965	-4.740086079	8.69496E-05	0.033506733
cg18694483	-0.525247871	-0.821913656	-4.739957386	8.69776E-05	0.033506733
cg19931913	-0.60576147	3.334444541	-4.739677215	8.70386E-05	0.033506733
cg14306953	-0.796123774	2.496862512	-4.739520414	8.70727E-05	0.033506733
cg20967514	0.611345701	1.21538416	4.738776249	8.7235E-05	0.033506733
cg08078514	-0.561607059	-0.155402484	-4.738699001	8.72518E-05	0.033506733
cg27397824	-0.780181121	0.69925661	-4.73846901	8.73021E-05	0.033506733
cg00133970	-0.626532125	4.111802652	-4.737973428	8.74104E-05	0.033506733
cg08633789	-0.691658524	3.001519237	-4.737702315	8.74697E-05	0.033506733
cg00527307	-0.795841147	0.472371525	-4.737508173	8.75122E-05	0.033506733
cg22954037	-0.434931166	4.418274297	-4.737315079	8.75545E-05	0.033506733
cg10957029	-0.566811351	-0.217326263	-4.73656347	8.77193E-05	0.033506733
cg03919779	-0.446657425	-1.251000755	-4.736389824	8.77574E-05	0.033506733
cg24313472	-0.575658646	0.387765774	-4.736356763	8.77646E-05	0.033506733
cg21731721	-0.765385021	2.546729746	-4.736159469	8.7808E-05	0.033506733
cg12024649	-0.687088889	2.794801075	-4.735938919	8.78564E-05	0.033506733
cg01115920	-0.760243818	3.007825539	-4.735712991	8.79061E-05	0.033506733
cg16599577	-0.774565064	2.345270543	-4.735640819	8.7922E-05	0.033506733
cg14295534	-0.887839047	2.744514927	-4.735440371	8.79661E-05	0.033506733
cg04092447	-0.51244085	3.321430914	-4.735331916	8.79899E-05	0.033506733
cg16320737	0.48720916	3.663091491	4.735224276	8.80136E-05	0.033506733
cg03093801	-0.77675293	-1.301886048	-4.734946901	8.80747E-05	0.033506733
cg22449766	-0.519667054	-3.4699087	-4.734439665	8.81866E-05	0.033506733
cg16710348	-0.429850891	4.087515447	-4.73432978	8.82108E-05	0.033506733
cg22576398	-0.564940935	3.204053317	-4.734259372	8.82264E-05	0.033506733
cg17141408	-0.393631429	-0.657290005	-4.734084407	8.8265E-05	0.033506733
cg19405834	-0.683101398	3.640542289	-4.734021618	8.82789E-05	0.033506733
cg06388363	-0.491247707	-0.297693293	-4.733955436	8.82935E-05	0.033506733
cg02328375	-0.91375798	2.535866004	-4.733839935	8.8319E-05	0.033506733
cg26888807	-0.714750894	2.140607169	-4.733646564	8.83617E-05	0.033506733
cg08147187	-0.630230276	0.202923589	-4.733026136	8.8499E-05	0.033506733
cg19055196	-0.52707342	-0.816732219	-4.732798454	8.85494E-05	0.033506733
cg15278577	-0.372015834	4.318802037	-4.732594156	8.85947E-05	0.033506733
cg19862235	-0.555388481	-0.127386206	-4.731969923	8.87332E-05	0.033506733
cg15654627	-0.72767185	2.527165281	-4.731868917	8.87556E-05	0.033506733
cg22377482	-0.568311131	3.62629147	-4.731838055	8.87625E-05	0.033506733
cg17324843	-0.940916394	1.769100066	-4.731801048	8.87707E-05	0.033506733
cg23459805	-0.562151827	3.271281093	-4.731740457	8.87841E-05	0.033506733
cg05598763	-0.471392197	3.291451293	-4.731541352	8.88284E-05	0.033506733
cg22382430	-0.523048636	3.162338103	-4.731460316	8.88464E-05	0.033506733
cg18217284	-0.811790613	0.762550194	-4.731376349	8.8865E-05	0.033506733
cg05396119	-0.589373175	2.927907602	-4.731224155	8.88989E-05	0.033506733
cg23207835	-0.621501827	2.504045504	-4.731153893	8.89145E-05	0.033506733

cg24453153	-0.931628921	2.209851076	-4.730791594	8.89952E-05	0.033506733
cg01821225	-0.568771974	3.15224377	-4.730492056	8.90619E-05	0.033506733
cg17219928	-0.788028891	2.095873081	-4.73015617	8.91368E-05	0.033506733
cg20913253	-0.503403227	-0.170643746	-4.730017478	8.91677E-05	0.033506733
cg13076784	-0.651230537	2.549020017	-4.729934948	8.91861E-05	0.033506733
cg15586979	-0.555001544	3.492098079	-4.72990683	8.91924E-05	0.033506733
cg05336056	-0.645975389	-0.510047969	-4.729756786	8.92259E-05	0.033506733
cg06412349	-0.555303511	3.685260475	-4.729488865	8.92857E-05	0.033506733
cg17312067	-0.598920582	3.41461545	-4.729411516	8.9303E-05	0.033506733
cg24827032	0.794328047	2.072346008	4.728978678	8.93997E-05	0.033525282
cg19821404	-0.531846434	3.354273627	-4.728588671	8.9487E-05	0.033540257
cg26691059	-0.637554385	-0.424006332	-4.728142839	8.95869E-05	0.033559935
cg10944441	-0.587685269	-0.30839492	-4.727215139	8.97951E-05	0.033620144
cg23017722	-1.006272212	2.665766796	-4.726992344	8.98452E-05	0.03362112
cg08102508	-0.481827485	-1.597130661	-4.726129251	9.00394E-05	0.03364766
cg26798738	-0.768317094	2.151663402	-4.725930838	9.00841E-05	0.03364766
cg21645290	-0.769553541	1.988590263	-4.725848727	9.01026E-05	0.03364766
cg11203293	-0.785210271	-1.181677607	-4.725833356	9.01061E-05	0.03364766
cg08076018	-0.572795764	-1.058278923	-4.72501661	9.02904E-05	0.033674961
cg00818868	-0.800754219	2.518896342	-4.724934397	9.0309E-05	0.033674961
cg09032059	-0.57970524	3.270460847	-4.724877666	9.03218E-05	0.033674961
cg08835962	-0.540852378	4.50234429	-4.724643642	9.03747E-05	0.03367696
cg11857368	-0.48872423	3.653823191	-4.723939252	9.05341E-05	0.033677756
cg15074728	-0.543643667	4.035346925	-4.723217607	9.06977E-05	0.033677756
cg22583808	-0.62276873	0.058022245	-4.723140405	9.07153E-05	0.033677756
cg15317061	-0.513620977	-1.371643977	-4.722827236	9.07864E-05	0.033677756
cg05286376	-0.560249131	3.803112524	-4.72226262	9.09147E-05	0.033677756
cg13128740	-0.548407806	3.409008929	-4.722088823	9.09543E-05	0.033677756
cg10066697	-0.635715695	2.893406713	-4.721689763	9.10451E-05	0.033677756
cg23088510	-0.597698218	-0.681208424	-4.721603616	9.10648E-05	0.033677756
cg25794730	-0.626957363	-1.669866414	-4.721135739	9.11714E-05	0.033677756
cg00038590	-0.571291753	3.008063915	-4.720949744	9.12139E-05	0.033677756
cg25178683	-0.527952628	-1.586584134	-4.720852813	9.1236E-05	0.033677756
cg08972882	-0.77025038	3.180086011	-4.720835115	9.12401E-05	0.033677756
cg00561281	-0.644745282	3.520829376	-4.720674464	9.12767E-05	0.033677756
cg06397427	-0.814940519	0.283351956	-4.720670863	9.12776E-05	0.033677756
cg11797364	-0.534852982	0.500504869	-4.720656843	9.12808E-05	0.033677756
cg12611488	-0.762506655	-2.332787688	-4.72042724	9.13332E-05	0.033677756
cg20050484	-0.718525367	0.146112047	-4.720414811	9.13361E-05	0.033677756
cg22692767	-0.472526377	3.481218755	-4.720366179	9.13472E-05	0.033677756
cg11734727	-0.52050603	3.836143639	-4.719935859	9.14456E-05	0.033677756
cg08462941	-0.580894083	0.103262595	-4.719395942	9.15692E-05	0.033677756
cg05155793	-0.581415626	-0.096266353	-4.719166341	9.16218E-05	0.033677756
cg05756019	-0.989513521	3.167668816	-4.719151615	9.16252E-05	0.033677756
cg04498511	-0.589123555	1.169597605	-4.719090288	9.16393E-05	0.033677756
cg23288398	-0.530104925	3.988421837	-4.718770313	9.17127E-05	0.033677756
cg27337752	-0.654299565	3.228428755	-4.718698035	9.17293E-05	0.033677756

cg08343834	-0.493188489	-0.867138792	-4.718582317	9.17558E-05	0.033677756
cg14723977	-0.626342612	-0.609925205	-4.718489421	9.17772E-05	0.033677756
cg06719018	-0.508748269	4.230993543	-4.718387645	9.18005E-05	0.033677756
cg27419138	-0.557131459	3.357213262	-4.717954634	9.19001E-05	0.033677756
cg06456840	-0.547392718	-0.251102729	-4.71706171	9.21056E-05	0.033677756
cg06994786	-0.702254081	2.861750814	-4.716975773	9.21254E-05	0.033677756
cg19160964	-0.484468074	3.221813989	-4.716751323	9.21772E-05	0.033677756
cg19447782	-0.733449433	2.982381415	-4.716599922	9.22121E-05	0.033677756
cg17549755	-0.7475094	2.372863104	-4.716411675	9.22556E-05	0.033677756
cg08540100	-0.493233061	-1.915575323	-4.716297983	9.22818E-05	0.033677756
cg03553826	-0.658187976	2.459429672	-4.716090983	9.23296E-05	0.033677756
cg01712742	-0.42352997	-0.186279239	-4.715594285	9.24445E-05	0.033677756
cg13098379	-0.54402125	-0.38031327	-4.715575323	9.24489E-05	0.033677756
cg13151100	-0.71001705	3.557033787	-4.714810936	9.26259E-05	0.033677756
cg15577262	-0.724672894	3.018769064	-4.714630699	9.26676E-05	0.033677756
cg05731859	-0.715289532	3.237733118	-4.71454446	9.26876E-05	0.033677756
cg02204989	-0.550672648	0.04003386	-4.714178416	9.27726E-05	0.033677756
cg25759020	-0.440985882	0.230078088	-4.714169515	9.27746E-05	0.033677756
cg13518677	-0.509824124	4.231252769	-4.713689248	9.28862E-05	0.033677756
cg14727110	-0.831589469	3.349933478	-4.713437166	9.29448E-05	0.033677756
cg01265068	-0.828319646	-0.082061397	-4.713304034	9.29758E-05	0.033677756
cg02934034	-0.503823175	3.15872847	-4.713303292	9.2976E-05	0.033677756
cg09588476	-0.837355498	2.563741243	-4.712775419	9.30989E-05	0.033677756
cg09060789	-0.501504185	-1.799514415	-4.712418176	9.31821E-05	0.033677756
cg09757359	-0.596681266	-0.365633591	-4.712256871	9.32198E-05	0.033677756
cg17235987	-0.56413208	3.894339975	-4.712055186	9.32668E-05	0.033677756
cg16342620	-0.504665157	0.208205002	-4.711718436	9.33454E-05	0.033677756
cg06983551	-0.508261413	-2.199352899	-4.711711849	9.3347E-05	0.033677756
cg23382784	-0.583996086	3.255025311	-4.711576802	9.33785E-05	0.033677756
cg20566475	-0.554510921	-0.672428162	-4.711537872	9.33876E-05	0.033677756
cg25703604	-0.602547608	-0.386527358	-4.711269822	9.34503E-05	0.033677756
cg25267808	0.701170781	-1.943180827	4.711239885	9.34573E-05	0.033677756
cg09144480	-0.553516436	3.687443766	-4.710741134	9.3574E-05	0.033677756
cg10338504	-0.593188402	-0.618620507	-4.710412545	9.3651E-05	0.033677756
cg07230890	-0.51869653	3.972906197	-4.710126014	9.37182E-05	0.033677756
cg24627325	-0.650224956	-0.746133937	-4.710056483	9.37345E-05	0.033677756
cg22583599	-0.554722052	3.462518069	-4.709953348	9.37587E-05	0.033677756
cg14918548	-0.495370623	-0.740682441	-4.709904632	9.37701E-05	0.033677756
cg00869372	-0.537188073	-1.000325192	-4.709862507	9.378E-05	0.033677756
cg03715057	-0.418072475	0.300020605	-4.709727474	9.38117E-05	0.033677756
cg18812697	-0.504848813	4.092516929	-4.709562625	9.38504E-05	0.033677756
cg01114846	-0.705492468	2.457542509	-4.70952928	9.38582E-05	0.033677756
cg13670099	-0.570970063	-0.437467314	-4.709255312	9.39226E-05	0.033677756
cg23725246	-0.859945311	2.389034248	-4.709098274	9.39595E-05	0.033677756
cg05083363	-0.546303053	2.602010676	-4.708815766	9.4026E-05	0.033677756
cg12395526	-0.522756284	3.747769444	-4.708523425	9.40948E-05	0.033677756
cg19392994	-0.586828163	3.276931407	-4.708179257	9.41759E-05	0.033677756

cg15923548	-0.563243101	1.680861177	-4.708071092	9.42014E-05	0.033677756
cg19493665	-0.488075083	4.104177465	-4.708063498	9.42031E-05	0.033677756
cg07827139	-0.516607859	0.537494038	-4.708040917	9.42085E-05	0.033677756
cg12771751	-0.605447201	3.432154326	-4.707879591	9.42465E-05	0.033677756
cg04299357	-0.484024537	-0.019312049	-4.707694668	9.42901E-05	0.033677756
cg09776122	-0.742454175	1.658851095	-4.707551225	9.4324E-05	0.033677756
cg03437096	-0.555548114	3.113443775	-4.707308267	9.43814E-05	0.033677756
cg15944684	-0.524742756	-2.322275561	-4.707252635	9.43945E-05	0.033677756
cg25851222	-0.461763039	4.048268556	-4.706987782	9.44571E-05	0.033677756
cg02073054	-0.715329785	2.557292085	-4.706840438	9.44919E-05	0.033677756
cg23139797	-0.571941269	-1.129805618	-4.706837368	9.44926E-05	0.033677756
cg03148084	-0.520912121	0.246116084	-4.706796869	9.45022E-05	0.033677756
cg23276502	-0.59062265	-0.187763554	-4.706762689	9.45103E-05	0.033677756
cg18420655	-0.669082481	2.525356341	-4.706472421	9.4579E-05	0.033677756
cg15663705	-0.558973607	3.590442911	-4.705919578	9.47099E-05	0.033677756
cg25337647	-0.610031718	2.864147014	-4.705537136	9.48006E-05	0.033677756
cg11641450	0.560443154	2.685869963	4.705404841	9.4832E-05	0.033677756
cg04522714	0.458859034	-2.93635239	4.704756383	9.4986E-05	0.033677756
cg11366422	-0.57695436	-0.536412962	-4.704750491	9.49874E-05	0.033677756
cg09607452	-0.808266366	0.74049257	-4.704601278	9.50229E-05	0.033677756
cg19290700	-0.937889716	1.581740703	-4.704544304	9.50365E-05	0.033677756
cg11828581	-0.620306096	2.937165987	-4.704517629	9.50428E-05	0.033677756
cg08797491	-0.535417757	-0.99358414	-4.70448693	9.50501E-05	0.033677756
cg11312284	-0.41395908	4.003217826	-4.70436595	9.50789E-05	0.033677756
cg18964021	-0.495027461	3.823081968	-4.704225552	9.51123E-05	0.033677756
cg15242463	-0.45596855	3.898574749	-4.704147609	9.51309E-05	0.033677756
cg18772739	-0.566229503	-1.686625883	-4.703849514	9.52019E-05	0.033677756
cg12418779	-0.827525665	2.943665265	-4.703818476	9.52093E-05	0.033677756
cg02505728	-0.544609415	-0.187424841	-4.703511813	9.52824E-05	0.033677756
cg04530613	-0.586069401	2.896735112	-4.703158772	9.53666E-05	0.033677756
cg11828510	-0.534157458	3.427779856	-4.702809672	9.54499E-05	0.033677756
cg22676038	-0.727364285	2.474358043	-4.702801931	9.54518E-05	0.033677756
cg10780638	-0.583159613	2.533070302	-4.702569938	9.55072E-05	0.033677756
cg18365765	-0.869932284	0.531321009	-4.702493923	9.55254E-05	0.033677756
cg04516932	-0.663738354	2.921427305	-4.702366044	9.5556E-05	0.033677756
cg17775034	-0.556157365	3.174223523	-4.702287318	9.55748E-05	0.033677756
cg12899775	-0.70206349	3.450606231	-4.702177828	9.5601E-05	0.033677756
cg11199506	-0.530067756	-0.701071182	-4.702155118	9.56064E-05	0.033677756
cg09760210	-0.58882649	3.431575979	-4.70190042	9.56674E-05	0.033681095
cg17685905	-0.675765832	2.752706483	-4.701545696	9.57524E-05	0.033681095
cg09807033	-0.810685697	1.948917403	-4.701495209	9.57645E-05	0.033681095
cg11401606	-0.487753759	3.665030968	-4.701256739	9.58216E-05	0.033681095
cg10185478	-0.449019755	-1.498507016	-4.700630598	9.59719E-05	0.033681095
cg24329798	-0.782729947	1.49709403	-4.700228593	9.60685E-05	0.033681095
cg12965934	0.704578991	-4.845837267	4.69994355	9.61371E-05	0.033681095
cg12416929	-0.494433235	-0.805460925	-4.699858359	9.61576E-05	0.033681095
cg26737330	-0.562250626	3.350354673	-4.699834075	9.61634E-05	0.033681095

cg11713064	-0.521059645	-0.074430918	-4.699801826	9.61712E-05	0.033681095
cg03827804	-0.50966753	2.65987285	-4.699753395	9.61828E-05	0.033681095
cg02311879	-0.674157686	3.379016451	-4.699727022	9.61892E-05	0.033681095
cg22122331	-0.461487672	-0.298632467	-4.699383641	9.62719E-05	0.033681095
cg05640540	-0.581374389	3.051011267	-4.699162764	9.63251E-05	0.033681095
cg07914576	-0.638840418	2.440336278	-4.698823994	9.64068E-05	0.033681095
cg26619570	-0.514570028	3.826258755	-4.698734719	9.64284E-05	0.033681095
cg03940005	-0.51980301	3.478957192	-4.698691456	9.64388E-05	0.033681095
cg06118056	-0.528215678	0.297192304	-4.698555036	9.64717E-05	0.033681095
cg23640339	-0.610463325	0.554436346	-4.698331932	9.65256E-05	0.033683306
cg01201295	0.654379234	3.053229742	4.697729601	9.66712E-05	0.033712342
cg04122493	-0.510635107	3.504040476	-4.697594209	9.6704E-05	0.033712342
cg06936388	-0.572651652	4.049162403	-4.696964387	9.68566E-05	0.033748917
cg12542106	-0.597788605	1.918776744	-4.69653038	9.69618E-05	0.033768986
cg19812432	-0.548088055	-0.738379818	-4.696052733	9.70778E-05	0.033781581
cg03828080	-0.685926463	1.196959564	-4.695599825	9.71879E-05	0.033781581
cg26476972	-0.691348787	2.447467589	-4.69481848	9.73782E-05	0.033781581
cg14626628	-0.632097872	2.576770611	-4.694729459	9.73999E-05	0.033781581
cg23036194	-0.588264003	3.755320303	-4.694676542	9.74128E-05	0.033781581
cg20555985	-0.516455807	3.970925599	-4.69456859	9.74391E-05	0.033781581
cg10052319	-0.598363611	2.673763463	-4.694311835	9.75017E-05	0.033781581
cg00786909	-0.656635779	-0.140408664	-4.694236335	9.75202E-05	0.033781581
cg00215587	-0.366587474	0.272743951	-4.694176159	9.75349E-05	0.033781581
cg10582860	-0.716904231	-2.872585379	-4.694126501	9.7547E-05	0.033781581
cg21059054	-0.680157222	2.016583903	-4.6941159	9.75496E-05	0.033781581
cg21827683	-0.685051083	2.60723697	-4.694031139	9.75703E-05	0.033781581
cg08481633	-0.565641137	-0.502531759	-4.693591757	9.76776E-05	0.033786929
cg24197288	-0.734885076	2.284591008	-4.693355724	9.77353E-05	0.033786929
cg19240985	-0.421213269	-0.010482262	-4.693157367	9.77839E-05	0.033786929
cg13073030	-0.805861707	3.101381218	-4.693017296	9.78182E-05	0.033786929
cg03215160	-0.603307371	0.488042407	-4.692636507	9.79114E-05	0.033786929
cg01256256	-0.550554471	3.714109049	-4.692398709	9.79697E-05	0.033786929
cg27647852	-0.82691063	0.599892488	-4.69230844	9.79919E-05	0.033786929
cg01273672	-0.486918411	3.939697291	-4.692089166	9.80457E-05	0.033786929
cg09493837	-0.627237312	2.763147276	-4.691910685	9.80895E-05	0.033786929
cg15325044	-0.889761503	2.213463387	-4.691831475	9.81089E-05	0.033786929
cg22210209	-0.664277528	0.296005458	-4.69182562	9.81104E-05	0.033786929
cg01773642	-0.515873736	3.972972994	-4.691423519	9.82092E-05	0.033804517
cg10606942	-0.447325341	3.975433448	-4.690964749	9.8322E-05	0.033819641
cg16817189	-0.486110392	0.196613793	-4.690249457	9.84982E-05	0.033819641
cg01679871	-0.605675288	3.556644153	-4.690079788	9.854E-05	0.033819641
cg22118524	-0.381301127	-2.524484979	-4.689959433	9.85697E-05	0.033819641
cg17424433	-0.580606334	0.317209458	-4.689873425	9.85909E-05	0.033819641
cg20736887	-0.598359137	3.150896556	-4.689756747	9.86197E-05	0.033819641
cg06569993	-0.543573376	3.754705288	-4.689658183	9.86441E-05	0.033819641
cg19181996	-0.610255546	3.847834373	-4.689503002	9.86824E-05	0.033819641
cg06881239	-0.570368962	-0.804664345	-4.68950143	9.86828E-05	0.033819641

cg17885507	-0.597612726	-0.656582161	-4.688597891	9.89062E-05	0.033870321
cg14282126	-0.520948615	3.828236151	-4.688228998	9.89976E-05	0.033870321
cg23230349	-0.922519697	2.024668107	-4.688052197	9.90414E-05	0.033870321
cg25982561	-0.608228427	3.762962706	-4.687130854	9.92701E-05	0.033870321
cg11315107	-0.569260069	0.008345663	-4.687128611	9.92706E-05	0.033870321
cg07321497	-0.631953946	0.964611618	-4.687064811	9.92865E-05	0.033870321
cg26893181	-0.564041491	3.484899888	-4.687055469	9.92888E-05	0.033870321
cg06100608	-0.568577302	3.056852387	-4.686980434	9.93074E-05	0.033870321
cg03469660	-0.911260031	1.226758147	-4.686812998	9.93491E-05	0.033870321
cg01549936	-0.660484363	2.438191524	-4.686636506	9.9393E-05	0.033870321
cg02322661	-0.678534179	2.817821064	-4.686386639	9.94552E-05	0.033870321
cg25476797	-0.567186277	3.100968644	-4.686225146	9.94954E-05	0.033870321
cg05864715	0.797881663	-4.976303785	4.686098103	9.9527E-05	0.033870321
cg02814033	-0.48982039	0.358672485	-4.686067543	9.95346E-05	0.033870321
cg10595532	-0.682206821	3.131118766	-4.685529411	9.96688E-05	0.033870321
cg07683385	-0.523772701	-0.996120328	-4.68552164	9.96707E-05	0.033870321
cg12171458	-0.662405043	2.917831067	-4.685371642	9.97082E-05	0.033870321
cg03619635	-0.524971898	3.928822913	-4.685195606	9.97521E-05	0.033870321
cg18628686	-0.816382676	1.659996795	-4.684921801	9.98205E-05	0.033870321
cg03049507	-0.562595251	3.764187592	-4.684882921	9.98302E-05	0.033870321
cg25835568	-0.503727511	3.245560143	-4.684864781	9.98347E-05	0.033870321
cg24040563	-0.709247164	0.959480097	-4.684264019	9.9985E-05	0.033904594
cg01890888	-0.70768385	-1.124330638	-4.684063062	0.000100035	0.033904594
cg22429100	-0.74079634	2.429931661	-4.68327758	0.000100232	0.033904594
cg07216436	-0.450153569	-0.519278867	-4.683154325	0.000100263	0.033904594
cg02623936	-0.52811243	3.304715653	-4.682912318	0.000100324	0.033904594
cg25248097	-0.767449694	2.742273914	-4.682713775	0.000100374	0.033904594
cg20030274	-0.904332681	2.279547072	-4.682561737	0.000100412	0.033904594
cg01973317	-0.549805043	3.180653101	-4.682503661	0.000100427	0.033904594
cg27118102	-0.578170017	4.039519733	-4.682433774	0.000100444	0.033904594
cg06252669	-0.543964978	-0.624986717	-4.682165211	0.000100512	0.033904594
cg04065725	-0.532269114	3.677727436	-4.681931983	0.00010057	0.033904594
cg25752864	-0.53076632	-0.670307144	-4.68146628	0.000100688	0.033904594
cg14995457	-0.47864705	3.586482517	-4.681212435	0.000100752	0.033904594
cg04348857	-0.513044289	4.064703182	-4.681155016	0.000100766	0.033904594
cg11505026	-0.898592693	-1.306505399	-4.680653318	0.000100893	0.033904594
cg01546378	-0.745773424	-2.273377311	-4.680594766	0.000100908	0.033904594
cg27530921	-0.590984764	3.739088925	-4.680511586	0.000100929	0.033904594
cg06576136	-0.834144278	2.29053827	-4.680109851	0.00010103	0.033904594
cg18912530	-0.510170856	0.124936765	-4.679614581	0.000101155	0.033904594
cg01475476	-0.520094839	3.716320813	-4.679410093	0.000101207	0.033904594
cg18360825	-0.394503291	0.461153623	-4.679159944	0.000101271	0.033904594
cg16346032	-0.519609152	0.706191229	-4.679125864	0.000101279	0.033904594
cg24951364	-0.68689449	2.5029203	-4.67867723	0.000101393	0.033904594
cg03746015	-0.53582437	2.927751496	-4.678428416	0.000101456	0.033904594
cg00411413	-0.584121245	0.050259652	-4.677763332	0.000101625	0.033904594
cg11672158	-0.523741397	2.739081768	-4.677594675	0.000101668	0.033904594

cg09881005	-0.48289937	4.065302354	-4.677572451	0.000101674	0.033904594
cg23079307	-0.588317296	-1.219862152	-4.677382057	0.000101722	0.033904594
cg23253243	-0.732876479	2.420561648	-4.677220521	0.000101763	0.033904594
cg08005872	-0.478154158	-0.612430578	-4.677198713	0.000101769	0.033904594
cg14771689	-0.562968817	-0.105508963	-4.677059823	0.000101804	0.033904594
cg10907062	-0.479410489	-1.502114591	-4.677016745	0.000101815	0.033904594
cg15865525	-0.577415158	3.149877861	-4.676874634	0.000101852	0.033904594
cg19523486	-0.708850298	2.529621661	-4.676705613	0.000101895	0.033904594
cg17609057	0.417030434	0.328410526	4.676192059	0.000102026	0.033904594
cg25934198	-0.52610711	0.507208635	-4.676026709	0.000102068	0.033904594
cg16550656	-0.701963018	3.042734861	-4.675986234	0.000102078	0.033904594
cg23210369	-0.60139318	3.809192464	-4.675732012	0.000102143	0.033904594
cg18273817	-0.627998603	2.870014647	-4.675566258	0.000102186	0.033904594
cg04707299	-0.538941259	3.813518705	-4.67542599	0.000102222	0.033904594
cg13125421	-0.514890648	0.586655138	-4.675420145	0.000102223	0.033904594
cg06079852	-0.583873547	3.732004119	-4.675384663	0.000102232	0.033904594
cg09017289	-0.56256138	3.628109941	-4.675329817	0.000102246	0.033904594
cg09952332	-0.523633841	2.825756592	-4.675246754	0.000102268	0.033904594
cg23101261	-0.522880402	-1.162447622	-4.675183138	0.000102284	0.033904594
cg21972733	-0.420437588	-1.052611922	-4.675045252	0.000102319	0.033904594
cg07340599	-0.617218911	2.961691691	-4.674693455	0.000102409	0.033904594
cg11464517	-0.573412966	3.157943544	-4.674687867	0.000102411	0.033904594
cg15009294	-0.437621357	4.134007477	-4.674599689	0.000102433	0.033904594
cg22272697	-0.481104526	-0.819962954	-4.674438495	0.000102475	0.033904594
cg26633191	-0.621823517	2.901426382	-4.674011815	0.000102584	0.033904594
cg17015744	-0.517045637	0.013624152	-4.673756275	0.00010265	0.033904594
cg06073570	-0.700746074	3.379665004	-4.673684618	0.000102668	0.033904594
cg18917803	-0.607126093	3.711580143	-4.673623935	0.000102684	0.033904594
cg23229584	-0.505938172	0.011876019	-4.673594012	0.000102692	0.033904594
cg16029260	-0.628903733	-0.665892129	-4.673467172	0.000102724	0.033904594
cg07349477	-0.758714165	-1.089375865	-4.672772728	0.000102903	0.033904594
cg10420716	-0.433424056	-0.197780335	-4.672648925	0.000102935	0.033904594
cg12956890	-0.857223773	1.60932973	-4.672645339	0.000102936	0.033904594
cg04670359	0.791622784	-5.020767464	4.67264433	0.000102936	0.033904594
cg09771998	-0.559499711	3.511428756	-4.672319294	0.00010302	0.033904594
cg24810932	-0.532321674	2.795690759	-4.672197542	0.000103051	0.033904594
cg18256118	-0.484899987	3.81627632	-4.672086929	0.00010308	0.033904594
cg18167944	-0.716861288	0.992665533	-4.671998834	0.000103102	0.033904594
cg14815878	-0.788649584	2.238291363	-4.671978828	0.000103108	0.033904594
cg08960006	-0.748364948	2.875255575	-4.671944986	0.000103116	0.033904594
cg04076337	-0.769279532	2.009048704	-4.671843575	0.000103143	0.033904594
cg11974120	-0.580412189	3.05577631	-4.671499349	0.000103231	0.033918084
cg25486438	-0.757984468	1.783923723	-4.669885042	0.000103649	0.033957989
cg26372885	-0.684189818	3.130957515	-4.669777845	0.000103677	0.033957989
cg24880680	-0.453409943	0.588099538	-4.66945254	0.000103762	0.033957989
cg05516619	-0.502747947	2.668355253	-4.669432924	0.000103767	0.033957989
cg03323911	-0.540617479	3.114960577	-4.66938912	0.000103778	0.033957989

cg16983361	-0.510004665	0.124104284	-4.669340697	0.000103791	0.033957989
cg12016862	-0.58706938	3.018147225	-4.669316679	0.000103797	0.033957989
cg21300422	-0.474880146	-1.13337334	-4.669192284	0.000103829	0.033957989
cg04141141	-0.673526393	3.034570624	-4.668669964	0.000103965	0.033957989
cg21122604	-0.453697471	3.908676616	-4.668584147	0.000103988	0.033957989
cg12139707	-0.499102584	0.131839106	-4.668540575	0.000103999	0.033957989
cg02540338	-0.710296228	3.011692335	-4.668465386	0.000104018	0.033957989
cg01012295	-0.451528852	4.003118423	-4.668314765	0.000104058	0.033957989
cg22174405	-0.491394812	-0.682214777	-4.668282452	0.000104066	0.033957989
cg13561503	-0.541195731	2.806314764	-4.668260193	0.000104072	0.033957989
cg03671054	-0.62682088	4.024312614	-4.668035066	0.000104131	0.033958235
cg16995606	-0.483190879	4.316625789	-4.667586531	0.000104248	0.033958235
cg00844901	-0.511484298	-0.776394744	-4.667413272	0.000104293	0.033958235
cg00025197	-0.506950638	0.149748097	-4.667254083	0.000104334	0.033958235
cg21790461	-0.532033352	0.838860589	-4.667163223	0.000104358	0.033958235
cg19115088	-0.495521788	-2.057135412	-4.667154876	0.00010436	0.033958235
cg14630933	-0.618303885	-0.788202963	-4.666659893	0.00010449	0.033982156
cg21533897	0.486649898	2.67115692	4.666424295	0.000104551	0.033982156
cg04097046	-0.604855578	2.67688852	-4.666231434	0.000104602	0.033982156
cg04734587	-0.579000987	3.41443174	-4.665628506	0.00010476	0.033982156
cg14797950	-0.571543819	-0.363866793	-4.665620231	0.000104762	0.033982156
cg08137638	-0.524766497	3.636512022	-4.665396822	0.000104821	0.033982156
cg05517456	-0.66993856	3.235711207	-4.665117183	0.000104894	0.033982156
cg11517848	-0.706737103	2.774972065	-4.664993538	0.000104927	0.033982156
cg03610123	-0.602941338	2.616134474	-4.664578374	0.000105036	0.033982156
cg17148134	-0.610594295	3.545729069	-4.664508582	0.000105054	0.033982156
cg03238185	-0.531055882	0.289269868	-4.664125351	0.000105155	0.033982156
cg16093863	-0.460368839	3.646028239	-4.663995723	0.000105189	0.033982156
cg16446041	-0.644880168	1.730568995	-4.663418844	0.000105341	0.033982156
cg15496771	-0.591247343	3.010457876	-4.663416074	0.000105342	0.033982156
cg04963089	-0.449877276	3.492047498	-4.663199145	0.000105399	0.033982156
cg03169191	-0.834482137	2.151174714	-4.662919562	0.000105473	0.033982156
cg13908076	-0.728825268	3.010284488	-4.66291191	0.000105475	0.033982156
cg00878274	-0.65562729	-1.381654486	-4.662673413	0.000105538	0.033982156
cg05266796	-0.574095546	4.202849647	-4.662520972	0.000105578	0.033982156
cg24702713	-0.705760088	1.323723816	-4.662215934	0.000105659	0.033982156
cg18537203	-0.620712338	0.490685358	-4.661960731	0.000105726	0.033982156
cg16264358	-0.491847883	4.400825376	-4.661895131	0.000105744	0.033982156
cg06218523	0.553273397	2.770306985	4.661763685	0.000105778	0.033982156
cg10413475	-0.570087695	1.795798046	-4.661708558	0.000105793	0.033982156
cg04294207	0.455142576	0.985382126	4.661664307	0.000105805	0.033982156
cg04300553	-0.977883662	0.885993721	-4.661623746	0.000105816	0.033982156
cg22996308	-0.434452474	3.68752545	-4.661222796	0.000105922	0.033982156
cg08202575	-0.789010852	3.212795251	-4.661076524	0.000105961	0.033982156
cg21332701	-0.519802989	3.216974318	-4.660961059	0.000105991	0.033982156
cg11148561	-0.702603401	1.974981824	-4.660776487	0.00010604	0.033982156
cg18973237	-0.720598099	2.972896721	-4.660674924	0.000106067	0.033982156

cg27006030	-0.488222294	1.204228609	-4.660510112	0.000106111	0.033982156
cg18862215	-0.584237455	-0.671742881	-4.660394543	0.000106142	0.033982156
cg13660126	-0.643876099	-0.950206837	-4.660311389	0.000106164	0.033982156
cg11411943	-0.989497169	2.173035286	-4.660234605	0.000106184	0.033982156
cg07776734	-0.796898387	-1.442115516	-4.660221484	0.000106188	0.033982156
cg13126097	-0.930577311	2.32189646	-4.659818135	0.000106295	0.033982156
cg04690793	-0.476163252	3.631376494	-4.659664546	0.000106336	0.033982156
cg27461835	-0.593742725	3.244879105	-4.659263174	0.000106443	0.033982156
cg12902410	-0.838170575	2.529870395	-4.659180351	0.000106465	0.033982156
cg09487757	-0.627398403	2.584436299	-4.659169999	0.000106468	0.033982156
cg24416186	-0.631166631	-0.155467174	-4.659104163	0.000106485	0.033982156
cg27560137	-0.720424159	2.584830493	-4.659061052	0.000106497	0.033982156
cg06943925	-0.709873609	1.269922833	-4.65869517	0.000106594	0.033992731
cg25204319	-0.460703704	-0.931760845	-4.658577116	0.000106626	0.033992731
cg21006923	-0.782521462	1.391501275	-4.657669753	0.000106868	0.034040733
cg23312158	-0.720998524	3.342807484	-4.657654145	0.000106872	0.034040733
cg20824932	-0.539611277	4.096659978	-4.657019569	0.000107042	0.034068059
cg11920708	-0.557502277	4.031459511	-4.656853087	0.000107087	0.034068059
cg03962463	-0.573036724	3.573741756	-4.656707061	0.000107126	0.034068059
cg13031943	-0.527404552	3.636012383	-4.656544464	0.00010717	0.034068059
cg00323962	-0.73339613	2.828746935	-4.656398511	0.000107209	0.034068059
cg24445405	-0.520636201	3.071657828	-4.65623534	0.000107253	0.034068059
cg19382584	-0.556094142	3.601379083	-4.656078502	0.000107295	0.034068059
cg01469409	-0.452870588	-1.079824918	-4.655371285	0.000107485	0.034110435
cg04274445	-0.534296303	-0.56408059	-4.654889597	0.000107615	0.034110435
cg25149136	-0.567934108	3.713255239	-4.654779815	0.000107644	0.034110435
cg06525698	-0.743827406	2.604184826	-4.654321286	0.000107768	0.034110435
cg27380552	-0.498164469	3.56518641	-4.654179359	0.000107806	0.034110435
cg01190084	-0.847353488	2.880599037	-4.65415272	0.000107813	0.034110435
cg03540403	-0.636235997	0.377042336	-4.653970812	0.000107863	0.034110435
cg16527909	-0.631089774	3.298215447	-4.653863803	0.000107891	0.034110435
cg17822633	-0.676060629	0.414613887	-4.653827494	0.000107901	0.034110435
cg11123847	-0.611623961	2.718881652	-4.653760321	0.000107919	0.034110435
cg18460809	-0.546752483	2.771119261	-4.653617607	0.000107958	0.034110435
cg20541376	-0.445065995	-1.080397327	-4.653125789	0.000108091	0.034137238
cg16392409	-0.87440068	1.686573081	-4.652940874	0.000108141	0.034137827
cg12181907	-0.698664435	2.580764705	-4.652666889	0.000108215	0.034141397
cg11860639	-0.531311448	4.401896217	-4.652361011	0.000108298	0.034141397
cg10037633	-0.501422352	-1.070135005	-4.652290054	0.000108317	0.034141397
cg15057043	-0.55973376	3.622795277	-4.652187815	0.000108345	0.034141397
cg12988850	-0.596641006	3.346141801	-4.651988598	0.000108399	0.034143243
cg03317433	-0.646676949	3.361607263	-4.651314665	0.000108582	0.034164757
cg23837774	-0.799467251	2.041348945	-4.65122213	0.000108607	0.034164757
cg07061975	0.512208042	1.904999961	4.650714707	0.000108746	0.034164757
cg04820094	-0.509245923	3.685398429	-4.650317789	0.000108854	0.034164757
cg18058538	-0.508111334	2.820096682	-4.650250386	0.000108872	0.034164757
cg19513484	-0.509506281	3.929511746	-4.650023016	0.000108934	0.034164757

cg04634514	-0.721137563	-1.451697408	-4.649916005	0.000108963	0.034164757
cg04790356	-0.730506625	1.583493032	-4.649811691	0.000108992	0.034164757
cg08234439	-0.68332986	2.735582306	-4.649753045	0.000109008	0.034164757
cg04193901	-0.643897081	-1.709265604	-4.64907876	0.000109192	0.034164757
cg16513933	-0.636035756	3.437050058	-4.648964323	0.000109223	0.034164757
cg02151535	-0.568757543	3.422284216	-4.648944459	0.000109229	0.034164757
cg03782845	-0.624245159	3.79978761	-4.648824913	0.000109261	0.034164757
cg24544594	-0.735933409	2.645112813	-4.648735007	0.000109286	0.034164757
cg02144870	-0.443241665	3.762016115	-4.648026786	0.00010948	0.034164757
cg12812788	-0.533748296	3.56511712	-4.647973679	0.000109494	0.034164757
cg21860329	-0.427883707	3.478113858	-4.647869157	0.000109523	0.034164757
cg05493358	-0.557704373	-1.058731495	-4.647307777	0.000109677	0.034164757
cg17814030	-0.606996537	-0.272119319	-4.646905146	0.000109788	0.034164757
cg03876225	-0.489495204	4.110671976	-4.646642506	0.00010986	0.034164757
cg10080497	-0.523983771	0.304494292	-4.646602193	0.000109871	0.034164757
cg06994884	-0.798671506	0.772343088	-4.646512393	0.000109896	0.034164757
cg27593778	-0.40022426	0.558701517	-4.64647031	0.000109907	0.034164757
cg16524207	-0.787278052	-0.394674324	-4.646235458	0.000109972	0.034164757
cg02668261	-0.449965079	3.818515139	-4.646219568	0.000109976	0.034164757
cg24034154	-0.837624093	1.480214438	-4.646087105	0.000110013	0.034164757
cg18344168	-0.887131247	1.726159596	-4.645956439	0.000110049	0.034164757
cg26684801	-0.480396517	4.251213505	-4.645935751	0.000110055	0.034164757
cg14437446	-0.550508433	3.695359816	-4.645852033	0.000110078	0.034164757
cg14173476	-0.499605601	-0.624754404	-4.645759429	0.000110103	0.034164757
cg19037598	-0.778400194	2.821526379	-4.645563273	0.000110157	0.034164757
cg14067880	-0.757657103	2.650564299	-4.6454	0.000110202	0.034164757
cg01965939	-0.568615435	-1.5904477	-4.645232094	0.000110249	0.034164757
cg25367075	-0.567756606	-0.718407402	-4.645165442	0.000110267	0.034164757
cg06440590	-0.881317476	1.982266424	-4.644613827	0.000110419	0.034164757
cg18085517	-0.54648653	-0.652001472	-4.644396241	0.00011048	0.034164757
cg24660073	-0.549835569	3.649616627	-4.644363024	0.000110489	0.034164757
cg08762432	-0.610062546	-1.994359092	-4.643935041	0.000110607	0.034164757
cg14335435	-0.416634916	4.055220269	-4.643812561	0.000110641	0.034164757
cg16368095	-0.574154742	3.35250865	-4.643612653	0.000110697	0.034164757
cg11502565	-0.41161393	3.589030672	-4.643551651	0.000110714	0.034164757
cg01322290	-0.644336673	3.683195253	-4.643538452	0.000110717	0.034164757
cg00267746	-0.448836399	-0.4662413	-4.6435234	0.000110721	0.034164757
cg27100953	-1.00455344	0.513762317	-4.643354782	0.000110768	0.034164757
cg08467539	-0.718135924	0.82173256	-4.643259498	0.000110795	0.034164757
cg09210935	-0.675837731	2.809725852	-4.643198128	0.000110812	0.034164757
cg01481283	-0.598154799	3.955715291	-4.642887502	0.000110898	0.034164757
cg18749404	-0.550662311	-1.257719911	-4.6428303	0.000110914	0.034164757
cg27309098	-0.740997303	-1.355446816	-4.642626669	0.00011097	0.034164757
cg00430155	-0.525696429	-2.057420665	-4.642608216	0.000110975	0.034164757
cg12654412	-0.457480318	-1.863249253	-4.642563414	0.000110988	0.034164757
cg03822593	-0.463572107	3.528154967	-4.642476435	0.000111012	0.034164757
cg12318676	-0.523505283	3.31373553	-4.642329964	0.000111053	0.034164757

cg14814103	-0.784141559	2.53241427	-4.641941921	0.000111161	0.034164757
cg07937999	-0.389746331	4.1903992	-4.641912178	0.000111169	0.034164757
cg17499294	-0.504175643	4.316537444	-4.641902441	0.000111172	0.034164757
cg08295429	-0.643355208	3.739460287	-4.641741163	0.000111217	0.034164757
cg05877591	-0.585817293	-1.314497572	-4.640942203	0.000111439	0.034204674
cg14553338	-0.69205362	-0.680964633	-4.640928629	0.000111443	0.034204674
cg03501725	-0.440697234	3.729678926	-4.640616574	0.00011153	0.034206082
cg20880520	-0.587015452	3.456950418	-4.64056627	0.000111544	0.034206082
cg02681115	-0.446000693	-0.725892735	-4.639817172	0.000111754	0.034255476
cg23964699	-0.758782167	2.897366071	-4.639270561	0.000111907	0.034277574
cg05889139	-0.679840689	2.845049626	-4.639037204	0.000111972	0.034277574
cg13469457	-0.704012974	-0.449764671	-4.638819412	0.000112033	0.034277574
cg08544453	-0.506927555	0.18785279	-4.638770861	0.000112047	0.034277574
cg02822089	-0.870442254	2.170706311	-4.63868968	0.00011207	0.034277574
cg20713510	-0.560626647	3.287065604	-4.638401871	0.00011215	0.034277574
cg03400808	-0.802236246	2.856807969	-4.638351829	0.000112164	0.034277574
cg24313993	-0.827810719	2.706222825	-4.637893601	0.000112293	0.03430213
cg10520307	-0.576386878	4.018765914	-4.63735863	0.000112444	0.034332272
cg16477869	-0.532578638	4.15744958	-4.637035869	0.000112535	0.034332272
cg16061863	-0.586199591	2.790032721	-4.63697397	0.000112552	0.034332272
cg22981898	-0.493379956	2.879909054	-4.636854618	0.000112586	0.034332272
cg18085821	-0.76708064	3.145402474	-4.636499993	0.000112686	0.034341804
cg00615485	-0.706118728	-1.773008035	-4.636400087	0.000112714	0.034341804
cg07456546	-0.509289033	3.570205363	-4.635966127	0.000112837	0.034359444
cg01973844	-0.931741179	1.728799381	-4.635822075	0.000112877	0.034359444
cg00088428	-0.55390078	3.518368548	-4.635393346	0.000112999	0.034359444
cg04605696	-0.691322553	1.029606968	-4.635393092	0.000112999	0.034359444
cg26905261	-0.513457746	-0.458815972	-4.635300127	0.000113025	0.034359444
cg24765791	-0.930083341	1.588436568	-4.635165778	0.000113063	0.034359444
cg13916056	-0.577711759	-2.286876211	-4.634788818	0.00011317	0.034369464
cg20847914	-0.52662082	3.638132247	-4.634346545	0.000113295	0.034369464
cg25911090	-0.571478306	3.904729245	-4.63427652	0.000113315	0.034369464
cg19474801	-0.593669252	3.954826432	-4.634243582	0.000113324	0.034369464
cg09157736	-0.487739717	3.998297446	-4.63419367	0.000113339	0.034369464
cg01877404	-0.743652534	2.609063271	-4.633813198	0.000113447	0.034387505
cg17444638	-0.56718105	3.169241583	-4.633592135	0.000113509	0.03439183
cg13075014	-0.731561641	3.196231859	-4.633135794	0.000113639	0.034400102
cg13571698	-0.809000108	2.399300799	-4.632909141	0.000113704	0.034400102
cg12818499	-0.592194618	1.778654196	-4.632811325	0.000113732	0.034400102
cg06079062	-0.505567834	3.414853121	-4.632310138	0.000113874	0.034400102
cg21762957	-0.545691751	3.23187951	-4.631987963	0.000113966	0.034400102
cg24465845	-0.552730616	3.168254168	-4.631695399	0.00011405	0.034400102
cg21243154	-0.532453043	3.329545056	-4.63149404	0.000114107	0.034400102
cg16083202	-0.65478321	3.120014762	-4.631404436	0.000114133	0.034400102
cg16453878	-0.854864154	0.975227402	-4.631173993	0.000114199	0.034400102
cg15373660	-0.593019382	2.828773338	-4.631127077	0.000114212	0.034400102
cg12640631	-0.66654362	2.8663974	-4.63102104	0.000114243	0.034400102

cg20696578	-0.485329517	-0.023374047	-4.6308072	0.000114304	0.034400102
cg26218229	-0.499167993	3.29323949	-4.630734784	0.000114324	0.034400102
cg10034863	-0.573154718	4.055230855	-4.630386268	0.000114424	0.034400102
cg11729734	-0.544434851	3.887632217	-4.63024401	0.000114465	0.034400102
cg02832056	-0.822958648	-1.755373864	-4.630233817	0.000114468	0.034400102
cg18107400	-0.665441736	2.834084101	-4.630081448	0.000114512	0.034400102
cg00496126	-0.479043529	3.738047495	-4.629912346	0.00011456	0.034400102
cg11782789	-0.386939888	-0.463902488	-4.629872418	0.000114572	0.034400102
cg05750936	-0.67625488	3.969203324	-4.629452572	0.000114692	0.034400102
cg17306624	-0.832483792	3.404979723	-4.629102633	0.000114793	0.034400102
cg26132715	-0.774752161	0.120151283	-4.628774062	0.000114887	0.034400102
cg08909148	-0.597315951	2.962808874	-4.628600237	0.000114937	0.034400102
cg20950090	-0.459621382	2.796062317	-4.628352433	0.000115009	0.034400102
cg12885203	-0.603195939	3.592043481	-4.628109552	0.000115079	0.034400102
cg07703643	-0.483918956	4.243536441	-4.627867673	0.000115148	0.034400102
cg06454983	-1.053656511	1.631771426	-4.627806364	0.000115166	0.034400102
cg14675124	-0.786352273	2.663933854	-4.627801604	0.000115167	0.034400102
cg15358855	-0.552517372	3.358033831	-4.627796103	0.000115169	0.034400102
cg19640026	-0.784657714	0.889525853	-4.627764426	0.000115178	0.034400102
cg02888474	-0.634352837	3.498604076	-4.627361005	0.000115294	0.034400102
cg06455851	-0.709266798	3.178617081	-4.627294135	0.000115314	0.034400102
cg13466917	-0.540535625	0.539747546	-4.627230489	0.000115332	0.034400102
cg06616221	-0.615442004	3.235279396	-4.627163648	0.000115351	0.034400102
cg14620117	-0.490311129	3.404544085	-4.627093434	0.000115372	0.034400102
cg08264335	-0.789484638	-3.909000242	-4.627023689	0.000115392	0.034400102
cg20933261	-0.462728455	3.588367819	-4.626686418	0.000115489	0.034400102
cg00738741	-0.5093032	3.691997598	-4.626557747	0.000115527	0.034400102
cg17892892	-0.722615825	2.400493301	-4.626203918	0.000115629	0.034400102
cg12268400	-0.660968117	2.668447702	-4.62603508	0.000115678	0.034400102
cg15139641	-0.864952326	0.80816969	-4.62570903	0.000115772	0.034400102
cg18216575	-0.462380429	-0.366098618	-4.625630739	0.000115795	0.034400102
cg09983196	-0.550642508	3.67045725	-4.625181705	0.000115925	0.034400102
cg23301376	-0.567003807	-0.223881247	-4.625135709	0.000115939	0.034400102
cg20804467	-0.391292172	4.105646194	-4.624709019	0.000116063	0.034400102
cg19773671	-0.584610801	3.720661092	-4.624602191	0.000116094	0.034400102
cg16214693	-0.501447514	0.200908546	-4.624394212	0.000116154	0.034400102
cg03264156	-0.552006642	3.481499794	-4.624343132	0.000116169	0.034400102
cg05270928	-0.485509743	2.896877857	-4.624326394	0.000116174	0.034400102
cg13038236	-0.502051329	3.837495938	-4.624322663	0.000116175	0.034400102
cg05165900	-0.52018011	0.284137257	-4.624076908	0.000116247	0.034400102
cg26753560	-0.639208795	2.624986526	-4.623955701	0.000116282	0.034400102
cg06726118	-0.48978983	3.951840803	-4.623864864	0.000116308	0.034400102
cg10331500	-0.54352671	2.915321047	-4.623750792	0.000116342	0.034400102
cg11301794	-0.597511179	-0.709028846	-4.623614391	0.000116381	0.034400102
cg27165761	-0.516827826	3.384083254	-4.623499287	0.000116415	0.034400102
cg26535070	-0.531377142	3.432591111	-4.623497225	0.000116415	0.034400102
cg03492827	-0.448577253	-2.153352959	-4.623312245	0.000116469	0.034400102

cg27224143	-0.580241961	0.250949395	-4.623302521	0.000116472	0.034400102
cg21104686	-0.633311559	0.361504072	-4.623184644	0.000116507	0.034400102
cg15389392	-0.632726224	1.923098102	-4.623166492	0.000116512	0.034400102
cg11328704	-0.597144051	-3.020236587	-4.623044735	0.000116547	0.034400102
cg09434586	0.686412673	-4.586633044	4.622388056	0.000116739	0.034422441
cg16284053	-0.86758591	2.175563495	-4.622356246	0.000116749	0.034422441
cg01974249	-0.692767185	-1.310492956	-4.622218701	0.000116789	0.034422441
cg16933320	-0.497001885	3.554524274	-4.621991816	0.000116855	0.034422441
cg27369502	-0.58277853	3.25976959	-4.621954487	0.000116866	0.034422441
cg05215605	-0.84220603	0.043429974	-4.621738442	0.000116929	0.034426756
cg10579279	-0.788667617	-1.808977377	-4.621454299	0.000117013	0.034430926
cg00048370	-0.661181885	-0.878012278	-4.621358295	0.000117041	0.034430926
cg20100014	-0.559661374	0.23305914	-4.620903745	0.000117174	0.034455835
cg26312252	-0.591380747	-1.532457155	-4.620032186	0.00011743	0.034516796
cg05948757	-0.897246286	3.104381953	-4.619792296	0.000117501	0.034523214
cg13631966	-1.132812603	1.541180277	-4.619475951	0.000117594	0.034525158
cg13792757	-0.626208336	2.539206713	-4.619438724	0.000117605	0.034525158
cg23740652	-0.694022747	-0.829371538	-4.618569874	0.000117861	0.034577087
cg07467532	-0.468005588	3.871857856	-4.61850778	0.000117879	0.034577087
cg08313618	-0.63863487	3.409588318	-4.618309747	0.000117938	0.034579921
cg18479249	-0.663315714	-0.632889281	-4.618061225	0.000118011	0.034587135
cg08270157	-0.604274998	3.514581046	-4.617572631	0.000118156	0.034608963
cg25961601	-0.676295453	3.22751143	-4.617479044	0.000118183	0.034608963
cg04326353	-0.444448265	3.042399891	-4.617190772	0.000118269	0.034615151
cg22848967	-0.521255971	3.102297693	-4.616993785	0.000118327	0.034615151
cg14106587	-0.674787266	3.009091158	-4.616783424	0.000118389	0.034615151
cg15317129	-0.678249968	0.017603037	-4.616747944	0.0001184	0.034615151
cg15050103	-0.484748106	3.387571782	-4.616318139	0.000118527	0.034628385
cg02908942	-0.658446103	1.27341021	-4.616157346	0.000118575	0.034628385
cg03263792	-0.389461161	0.939067943	-4.616101253	0.000118592	0.034628385
cg01940181	-0.824541783	-1.791984942	-4.615782981	0.000118686	0.034641719
cg04911518	-0.879790841	2.819283006	-4.615369976	0.000118809	0.034663287
cg17184927	-0.489369098	0.044433074	-4.615020471	0.000118913	0.034679359
cg22224666	-0.789093363	1.575517483	-4.614104092	0.000119187	0.034742435
cg04281401	-0.457423848	-0.155842481	-4.613729556	0.000119298	0.034742435
cg13058868	-0.626211207	3.254651691	-4.613653769	0.000119321	0.034742435
cg25005356	-0.478432924	3.703835592	-4.613637911	0.000119326	0.034742435
cg27474223	-0.588944813	3.309035392	-4.61317641	0.000119464	0.03476145
cg19309504	-0.504461051	4.187506479	-4.612886807	0.00011955	0.03476145
cg05003364	-0.634699596	2.513515523	-4.612878472	0.000119553	0.03476145
cg22786486	-0.545097186	3.320989402	-4.612466268	0.000119676	0.03476145
cg17228824	-0.634590163	3.325502238	-4.612461996	0.000119678	0.03476145
cg04378659	-0.691964587	2.169303533	-4.61243599	0.000119686	0.03476145
cg02085786	-0.677091454	3.051130946	-4.612175582	0.000119764	0.034769873
cg09218117	-0.540353777	3.540685312	-4.611611376	0.000119933	0.034791897
cg19649172	-0.538093824	-0.478529868	-4.611506977	0.000119964	0.034791897
cg07661480	-0.397160403	-0.332357141	-4.611432131	0.000119987	0.034791897

cg17336337	-0.691525101	-0.173960428	-4.611119472	0.000120081	0.034804906
cg05046589	-0.600342088	-0.23263763	-4.610943852	0.000120134	0.034805976
cg22450974	-0.432152756	2.874721259	-4.6103703	0.000120306	0.034830085
cg05109096	-0.570088815	3.155196833	-4.610271557	0.000120336	0.034830085
cg06941127	-0.547531581	-0.882296326	-4.609686267	0.000120513	0.034830085
cg05049822	-3.851976385	4.35858227	-4.609333599	0.000120619	0.034830085
cg06399992	-0.972527893	1.148357763	-4.609146201	0.000120676	0.034830085
cg16565890	-0.560331254	3.401184712	-4.608908747	0.000120748	0.034830085
cg07213264	-0.483736943	4.130595389	-4.608880961	0.000120756	0.034830085
cg23283216	-0.475419338	3.952741857	-4.608704872	0.000120809	0.034830085
cg21593496	-0.562570075	3.608391876	-4.608395525	0.000120903	0.034830085
cg13158989	-0.565568946	3.648963573	-4.608297409	0.000120933	0.034830085
cg08968935	-0.647034286	2.923628463	-4.608279399	0.000120938	0.034830085
cg26177041	-0.489170934	-1.160448071	-4.608134397	0.000120982	0.034830085
cg05758931	-0.641746924	2.030233372	-4.608025799	0.000121015	0.034830085
cg13777511	-0.661576268	3.089446	-4.608014295	0.000121018	0.034830085
cg23248184	-0.775526765	3.479267261	-4.607854145	0.000121067	0.034830085
cg03240700	-0.572727722	3.142653838	-4.607800698	0.000121083	0.034830085
cg00020617	-0.386624191	4.327055964	-4.607587983	0.000121148	0.034830085
cg25087750	-0.658568606	2.298083912	-4.607348592	0.00012122	0.034830085
cg09786062	-0.550810347	2.753277889	-4.606948324	0.000121342	0.034830085
cg20138486	-0.504523918	2.59911864	-4.606861187	0.000121368	0.034830085
cg05743798	-0.698466682	2.152367305	-4.606858308	0.000121369	0.034830085
cg12883152	-0.395354138	-0.89407602	-4.606841056	0.000121374	0.034830085
cg04947397	-0.672961968	3.255269461	-4.606792772	0.000121389	0.034830085
cg19090360	-0.587709288	0.069927432	-4.606578217	0.000121454	0.034830085
cg09856045	-0.871890381	2.359311376	-4.606480052	0.000121484	0.034830085
cg26577320	-0.427595009	-0.886296628	-4.606280631	0.000121545	0.034830085
cg15877238	-0.507883172	-1.248611279	-4.606169142	0.000121579	0.034830085
cg05535321	-0.718072529	2.415486617	-4.606120975	0.000121594	0.034830085
cg22317443	-0.585065401	-0.851143354	-4.604869658	0.000121975	0.034925294
cg08308834	-0.614434725	3.0360673	-4.603913695	0.000122268	0.034968915
cg18333653	-0.825217002	1.204568539	-4.603803023	0.000122302	0.034968915
cg11043888	0.496571957	1.124666049	4.603693599	0.000122335	0.034968915
cg02826180	-0.6141491	3.364561221	-4.603634618	0.000122353	0.034968915
cg06932691	-0.683005417	1.990197587	-4.603449263	0.00012241	0.034968915
cg01028226	-0.59063128	-2.189928204	-4.603404179	0.000122424	0.034968915
cg21774861	-0.464112532	3.62590476	-4.603183744	0.000122491	0.034974125
cg10896276	-0.559273427	3.647611823	-4.602839603	0.000122597	0.034990181
cg00964055	-0.83618237	-4.933466238	-4.602649538	0.000122655	0.034992743
cg20754964	-0.497386472	3.751227264	-4.602101006	0.000122824	0.035026746
cg13809660	-0.497486032	-1.021685764	-4.601934523	0.000122875	0.035027253
cg26573923	-0.504244059	0.200386388	-4.601602286	0.000122978	0.035042312
cg16544246	-0.786983482	2.783926907	-4.601280386	0.000123077	0.035056475
cg14171052	-0.474436385	-1.033887439	-4.601031683	0.000123153	0.03506422
cg13714636	-0.53855731	3.275106541	-4.600613908	0.000123282	0.03507131
cg03813721	-0.827001963	2.714072825	-4.600488659	0.000123321	0.03507131

cg16711597	-0.56266579	-0.185676725	-4.600441662	0.000123336	0.03507131
cg26034145	-0.44230629	4.246235533	-4.600309601	0.000123376	0.03507131
cg01127207	-0.715950691	3.443867636	-4.599986759	0.000123476	0.035072037
cg18730882	-0.505166364	3.919282666	-4.599981027	0.000123478	0.035072037
cg01937267	-0.549384666	3.343416116	-4.599520463	0.00012362	0.035098446
cg03922783	-0.850740839	2.023485153	-4.598593094	0.000123908	0.035117519
cg09845296	-0.6797811	2.75307527	-4.598446014	0.000123954	0.035117519
cg24457647	-0.742461168	0.738144459	-4.598345066	0.000123985	0.035117519
cg16941302	-0.517960028	-2.813572182	-4.598336278	0.000123988	0.035117519
cg11073717	-0.420349496	-0.276232028	-4.598309286	0.000123996	0.035117519
cg08122234	-0.680038431	0.070598704	-4.598246688	0.000124015	0.035117519
cg20899927	-0.700441071	3.298319326	-4.598185002	0.000124035	0.035117519
cg01869388	-0.639741722	2.63686737	-4.597791017	0.000124157	0.035119396
cg05380935	-0.518581688	-0.019067127	-4.597494444	0.000124249	0.035119396
cg16172672	-0.651469247	2.150648794	-4.59748987	0.000124251	0.035119396
cg03705571	-0.643226216	-0.236763045	-4.597392526	0.000124281	0.035119396
cg09512331	-0.571194759	3.380847613	-4.596911182	0.000124431	0.035119396
cg07224380	-0.495645954	0.568616844	-4.596844472	0.000124452	0.035119396
cg03435735	-0.595615268	3.400566912	-4.596834559	0.000124455	0.035119396
cg15408855	-0.523455946	-1.288915206	-4.596768197	0.000124476	0.035119396
cg03569214	-0.867636223	2.857539388	-4.59650932	0.000124556	0.035119396
cg23156071	-0.457112193	0.171480239	-4.596390834	0.000124593	0.035119396
cg19536713	-0.433910401	-0.933809974	-4.596269522	0.000124631	0.035119396
cg14067578	-0.531614541	3.346480157	-4.595726046	0.000124801	0.035119396
cg24011341	-0.543292705	3.400244501	-4.595636087	0.000124829	0.035119396
cg23678918	-0.568788856	-0.055897141	-4.59549451	0.000124873	0.035119396
cg15717840	-0.471834251	0.300847797	-4.595181237	0.000124971	0.035119396
cg06899312	-0.435210737	0.305904814	-4.595112868	0.000124993	0.035119396
cg16936421	-0.444993939	0.008944154	-4.594981299	0.000125034	0.035119396
cg12801066	-0.46808296	3.926184193	-4.594963658	0.000125039	0.035119396
cg17775180	-0.676929994	1.129670833	-4.594905464	0.000125058	0.035119396
cg05697693	-0.572083264	2.911339135	-4.594764059	0.000125102	0.035119396
cg20827629	-0.63350347	4.008904842	-4.594744142	0.000125108	0.035119396
cg08977091	-0.52321934	1.719056063	-4.594668507	0.000125132	0.035119396
cg08979261	-0.628120551	4.484174867	-4.5940587	0.000125323	0.035159149
cg19572170	-0.581410397	3.40473055	-4.593373815	0.000125538	0.035163564
cg09790284	-0.564273453	3.53614809	-4.593118989	0.000125619	0.035163564
cg16632994	0.789720192	-5.041780486	4.593097587	0.000125625	0.035163564
cg19281706	-0.669090787	0.720502812	-4.593096907	0.000125626	0.035163564
cg26828835	-0.741017125	-1.271684458	-4.592683771	0.000125756	0.035163564
cg17305215	-0.833036733	2.785052455	-4.592561426	0.000125794	0.035163564
cg05888518	-0.6035413	-0.361053795	-4.592517228	0.000125808	0.035163564
cg11793646	-0.5667796	0.014758264	-4.592517132	0.000125808	0.035163564
cg04172297	-0.484779516	4.068141407	-4.592495052	0.000125815	0.035163564
cg14234912	-0.685012448	0.539513326	-4.59193605	0.000125991	0.035163564
cg15711240	-0.486524559	3.340978234	-4.591552711	0.000126112	0.035163564
cg24427649	-0.4872406	0.051848084	-4.591403818	0.000126159	0.035163564

cg18082894	-0.481808793	-1.245704819	-4.591307027	0.00012619	0.035163564
cg10841979	-0.401171249	3.446649203	-4.591111692	0.000126252	0.035163564
cg03694841	-0.522986352	-1.690747241	-4.590861983	0.000126331	0.035163564
cg12165664	-0.520188514	0.164754729	-4.590803806	0.000126349	0.035163564
cg16134127	-0.54040909	3.365772746	-4.590591093	0.000126417	0.035163564
cg03732749	-0.61860783	3.253541616	-4.590392429	0.000126479	0.035163564
cg19371646	-0.553469538	3.430275476	-4.590391229	0.00012648	0.035163564
cg09657382	-0.432980578	-0.990553233	-4.590275303	0.000126517	0.035163564
cg13455257	-0.484000904	1.833752136	-4.590088709	0.000126576	0.035163564
cg08735991	-0.750259823	2.941088082	-4.589926482	0.000126627	0.035163564
cg15754752	-0.596606759	-0.42132039	-4.589854411	0.00012665	0.035163564
cg07048375	-0.502067633	3.957472726	-4.589584325	0.000126736	0.035163564
cg16434331	-0.726237496	-1.145822785	-4.589475131	0.00012677	0.035163564
cg05606878	-0.43269622	0.07977845	-4.589364195	0.000126806	0.035163564
cg22614027	-0.462156755	3.027534937	-4.589346956	0.000126811	0.035163564
cg13031608	-0.716313247	2.730110046	-4.589279027	0.000126833	0.035163564
cg04843688	-0.471201831	3.686369191	-4.589260794	0.000126838	0.035163564
cg13306195	-0.734277657	3.70624111	-4.589233774	0.000126847	0.035163564
cg24860036	-0.558493408	2.804724152	-4.589045304	0.000126907	0.035163564
cg15613991	-0.567252036	-2.927477334	-4.5888525	0.000126968	0.035163564
cg03881527	-0.431079986	3.180292042	-4.588774147	0.000126993	0.035163564
cg02643883	-0.569250319	3.689621228	-4.588036039	0.000127228	0.035163564
cg20432810	-0.684324655	1.953548868	-4.588031895	0.000127229	0.035163564
cg18417622	-0.718361386	-1.9420379	-4.587904005	0.00012727	0.035163564
cg18222195	-0.516505834	4.092233615	-4.587392635	0.000127433	0.035163564
cg26424649	-0.522462048	-0.776333249	-4.587313518	0.000127459	0.035163564
cg10842318	-0.474890697	4.013986476	-4.587118348	0.000127521	0.035163564
cg14060364	-0.744904492	2.837182154	-4.586954291	0.000127573	0.035163564
cg02648943	-0.489597917	-0.901433327	-4.586572353	0.000127695	0.035163564
cg05071691	-0.584404086	3.626869522	-4.586414993	0.000127746	0.035163564
cg11699531	-0.654015137	-0.137227291	-4.586161925	0.000127827	0.035163564
cg14295188	-0.396317436	4.108863439	-4.586069002	0.000127857	0.035163564
cg10019593	-0.502460085	4.020952256	-4.585622878	0.000128	0.035163564
cg18556803	-0.428061829	3.772190742	-4.585181483	0.000128141	0.035163564
cg12692673	-0.418205453	4.375538456	-4.58494148	0.000128218	0.035163564
cg04350236	-0.704046314	3.369476734	-4.584854648	0.000128246	0.035163564
cg09500171	-0.622089188	-0.125245457	-4.584767918	0.000128274	0.035163564
cg25635022	-0.543467744	-0.202093464	-4.58476647	0.000128274	0.035163564
cg01481569	-0.576057999	3.631585254	-4.584697403	0.000128297	0.035163564
cg18770728	-0.700734298	2.812146292	-4.584437485	0.00012838	0.035163564
cg06528466	-0.812094976	2.326686883	-4.58422958	0.000128447	0.035163564
cg10883123	-0.507934685	-0.811145779	-4.584194861	0.000128458	0.035163564
cg13457276	-0.570533597	0.794125358	-4.584061445	0.000128501	0.035163564
cg25226141	-0.742306068	2.316813634	-4.584050018	0.000128505	0.035163564
cg15484904	-0.392117624	3.941554804	-4.583771602	0.000128595	0.035163564
cg04969657	-0.48181694	0.270063688	-4.583437934	0.000128702	0.035163564
cg04603972	0.8066132	-3.196647012	4.58329335	0.000128749	0.035163564

cg09019635	-0.649903593	0.193947085	-4.583018403	0.000128837	0.035163564
cg04382991	-0.633816127	3.15393345	-4.582932809	0.000128865	0.035163564
cg21315468	-0.694109649	2.780926126	-4.582844622	0.000128893	0.035163564
cg12066683	-0.406517716	4.045369023	-4.582814998	0.000128903	0.035163564
cg24297839	-0.801561644	1.739329484	-4.582714864	0.000128935	0.035163564
cg10441250	-0.543575935	-0.606499133	-4.582680828	0.000128946	0.035163564
cg12367874	-0.510373446	4.167390616	-4.582631694	0.000128962	0.035163564
cg07855449	-0.443675517	3.366051232	-4.582387293	0.000129041	0.035163564
cg05294459	-0.447873608	-0.394674242	-4.582131846	0.000129124	0.035163564
cg15788572	-0.516698898	0.258734632	-4.582063242	0.000129146	0.035163564
cg25949033	-0.418486241	-0.092352442	-4.581997506	0.000129167	0.035163564
cg02237755	-0.716478487	-0.386200688	-4.581827175	0.000129222	0.035163564
cg25826013	-0.869452022	1.907814718	-4.581794983	0.000129233	0.035163564
cg10872106	-0.74559919	2.21839073	-4.581713395	0.000129259	0.035163564
cg03977009	-0.600566314	3.599346985	-4.581472336	0.000129337	0.035163564
cg06964876	-0.502337899	-0.265206883	-4.581435718	0.000129349	0.035163564
cg23465967	-0.42679212	-2.16334116	-4.581434992	0.000129349	0.035163564
cg04026154	-0.549868612	3.344266793	-4.581364578	0.000129372	0.035163564
cg17562874	-0.699895958	3.13212339	-4.580922627	0.000129516	0.035163564
cg25840608	-0.565215092	-1.117881585	-4.580261961	0.00012973	0.035163564
cg20202874	-0.695952745	3.285189723	-4.579839512	0.000129867	0.035163564
cg23174663	-0.70482318	2.298506418	-4.579802657	0.000129879	0.035163564
cg17609359	-0.574443642	3.113199168	-4.5795974	0.000129946	0.035163564
cg06717875	-0.492370358	3.848892016	-4.579382177	0.000130016	0.035163564
cg10907484	-0.822126855	3.838538447	-4.579328369	0.000130034	0.035163564
cg19966444	-0.531912194	3.460013809	-4.578957661	0.000130155	0.035163564
cg25265010	-0.600444179	3.075919687	-4.578782308	0.000130212	0.035163564
cg00523547	-0.450616343	-0.411891984	-4.578448644	0.000130321	0.035163564
cg09099242	-0.411836145	-0.804935901	-4.57798262	0.000130473	0.035163564
cg10665117	-0.545522155	3.621652878	-4.577843176	0.000130518	0.035163564
cg09576364	-0.537564201	3.529792278	-4.577815349	0.000130528	0.035163564
cg17620300	-0.514919834	3.344128604	-4.577789576	0.000130536	0.035163564
cg04971825	-0.581969338	1.435241009	-4.577580231	0.000130604	0.035163564
cg05885012	-0.441883802	-2.008707877	-4.577493937	0.000130633	0.035163564
cg02967055	-0.549987803	3.353795047	-4.577443874	0.000130649	0.035163564
cg27138518	-0.786737842	1.906622117	-4.577179131	0.000130736	0.035163564
cg19979384	-0.543005639	3.341061566	-4.577100264	0.000130762	0.035163564
cg07629109	-0.538318492	3.507097179	-4.577074452	0.00013077	0.035163564
cg20134805	-0.60895961	3.124884203	-4.57694754	0.000130812	0.035163564
cg05477953	-0.748411483	2.531025028	-4.576868671	0.000130837	0.035163564
cg13553498	-0.663673361	3.199166207	-4.576774686	0.000130868	0.035163564
cg13939296	-0.516411883	3.414369143	-4.57677447	0.000130868	0.035163564
cg09978047	-0.639164402	2.584760399	-4.576684135	0.000130898	0.035163564
cg10347456	-0.598577205	-0.559029799	-4.576579309	0.000130932	0.035163564
cg07224636	-0.550680957	4.057297994	-4.576378927	0.000130998	0.035163564
cg10562478	-0.568884729	-0.278945138	-4.576340226	0.000131011	0.035163564
cg10445599	-0.630957282	-1.510363081	-4.576192656	0.000131059	0.035163564

cg02537342	-0.824651097	0.970680695	-4.575782515	0.000131194	0.035163564
cg13858885	-0.589677542	3.013323229	-4.575728252	0.000131212	0.035163564
cg05279413	-0.648333275	-2.596849009	-4.575696376	0.000131222	0.035163564
cg18485992	-0.526985494	3.684088041	-4.575001958	0.000131451	0.035163564
cg11369729	-0.449532727	3.939131469	-4.574925748	0.000131476	0.035163564
cg13902777	-0.420014464	-0.470430594	-4.57491007	0.000131481	0.035163564
cg16979165	-0.510375536	-0.425816928	-4.574909043	0.000131481	0.035163564
cg01010839	-0.633451762	2.428058148	-4.574830227	0.000131507	0.035163564
cg16819272	-0.561277912	3.899787717	-4.57477778	0.000131525	0.035163564
cg26391958	-0.589715803	3.768895209	-4.574541524	0.000131602	0.035163564
cg10003526	-0.563649649	3.075856729	-4.574299902	0.000131682	0.035163564
cg23680134	-0.505858049	3.484282913	-4.573999323	0.000131781	0.035163564
cg22791307	0.746752573	-2.801926824	4.573791829	0.00013185	0.035163564
cg11183602	-0.58256284	3.326820807	-4.573739833	0.000131867	0.035163564
cg21929681	-0.585209145	2.581669723	-4.573241605	0.000132032	0.035163564
cg27413600	-0.663538772	3.280280332	-4.573143872	0.000132064	0.035163564
cg04190745	-0.554891642	3.579405548	-4.573084214	0.000132084	0.035163564
cg08304325	-0.448630927	3.977935668	-4.572725464	0.000132202	0.035163564
cg21843573	-0.741688059	2.10581654	-4.572576943	0.000132252	0.035163564
cg13676902	-0.847301502	1.094603733	-4.572362667	0.000132323	0.035163564
cg09089239	-0.572384875	3.22487982	-4.572249236	0.00013236	0.035163564
cg09994906	0.690722109	1.526438624	4.572119026	0.000132403	0.035163564
cg03572619	-0.593027295	3.758544413	-4.572117059	0.000132404	0.035163564
cg05349788	-0.474154436	-1.70870164	-4.572089526	0.000132413	0.035163564
cg04499792	-0.492817085	-0.194350771	-4.572075452	0.000132418	0.035163564
cg22273223	-0.577944754	2.792516735	-4.57206284	0.000132422	0.035163564
cg13108681	-0.523417675	-1.414638805	-4.572058607	0.000132424	0.035163564
cg02066547	-0.47547191	3.75316865	-4.572022432	0.000132436	0.035163564
cg09366475	-0.568917	1.212608343	-4.571688443	0.000132546	0.035163564
cg09401623	-0.499906614	0.826251066	-4.571679659	0.000132549	0.035163564
cg14074539	-0.51958301	4.016350597	-4.571662112	0.000132555	0.035163564
cg10177585	-0.716217515	3.229330054	-4.571636861	0.000132563	0.035163564
cg12675852	-0.468640241	-1.033152984	-4.571602243	0.000132575	0.035163564
cg25495341	-0.538989363	3.972460819	-4.571479051	0.000132616	0.035163564
cg04497015	0.674321428	2.60902682	4.57145694	0.000132623	0.035163564
cg20359654	-0.461171828	4.08695752	-4.571218806	0.000132702	0.035163564
cg00763172	-0.789708624	2.380256964	-4.571167481	0.000132719	0.035163564
cg09836940	-0.712630431	0.651356716	-4.571109888	0.000132739	0.035163564
cg16598670	-0.464831476	3.501421443	-4.571102399	0.000132741	0.035163564
cg01292545	-0.49296542	3.422455864	-4.571058893	0.000132756	0.035163564
cg23807580	-0.776385506	1.669517132	-4.571041825	0.000132761	0.035163564
cg02704594	-0.486048996	3.625109768	-4.570894284	0.00013281	0.035163564
cg26592247	-0.418129344	-0.868967891	-4.569815793	0.00013317	0.035163564
cg07963361	-0.659937109	-3.955350084	-4.569731276	0.000133198	0.035163564
cg02620403	-0.558641626	3.613101449	-4.569604142	0.00013324	0.035163564
cg10938593	-0.627018139	3.595605962	-4.569455265	0.00013329	0.035163564
cg11705747	-0.662290161	2.47189377	-4.569385692	0.000133313	0.035163564

cg04001210	-0.65821467	1.196210618	-4.569383359	0.000133314	0.035163564
cg09419185	-0.702474716	2.92953024	-4.569121281	0.000133402	0.035163564
cg01582937	-0.756516133	-1.58342501	-4.569012806	0.000133438	0.035163564
cg10863113	-0.523375476	-0.940359887	-4.568635292	0.000133564	0.035163564
cg22996577	-0.583558829	3.188586788	-4.568529922	0.000133599	0.035163564
cg13042511	-0.653838102	2.632795035	-4.568522147	0.000133602	0.035163564
cg17508816	-0.356188266	-0.691750184	-4.568486732	0.000133614	0.035163564
cg17905632	-0.633704909	2.734994639	-4.568450156	0.000133626	0.035163564
cg12093229	-0.873213472	1.923676073	-4.568372974	0.000133652	0.035163564
cg05066250	-0.5378825	-0.169634903	-4.56835917	0.000133656	0.035163564
cg08545909	-0.531620118	1.912901225	-4.56833053	0.000133666	0.035163564
cg12651154	-0.610098969	2.790638486	-4.568277657	0.000133684	0.035163564
cg14201389	-0.561895395	2.511360928	-4.568067678	0.000133754	0.035163564
cg20522673	-0.732426974	0.623712487	-4.567477991	0.000133952	0.035163564
cg26591958	-0.771735713	3.933709894	-4.566708578	0.00013421	0.035163564
cg17840481	-0.455192372	0.708980737	-4.566574117	0.000134255	0.035163564
cg12918604	-0.456362225	3.561529106	-4.566510325	0.000134277	0.035163564
cg18036068	-0.621354671	3.546142423	-4.56642772	0.000134305	0.035163564
cg04006953	-0.901189809	2.645763792	-4.566368763	0.000134325	0.035163564
cg26789284	-0.572091867	-0.965299788	-4.56627621	0.000134356	0.035163564
cg24471024	-0.540259288	0.192643931	-4.566218496	0.000134375	0.035163564
cg05559643	-0.478621937	3.869063615	-4.565395922	0.000134652	0.035163564
cg09001369	-0.726359139	2.237346645	-4.565232682	0.000134707	0.035163564
cg20865048	-0.539418244	3.144192364	-4.565202665	0.000134718	0.035163564
cg21520050	-0.448426189	3.345144383	-4.565169939	0.000134729	0.035163564
cg22117079	-0.751726986	3.2229049	-4.565111494	0.000134748	0.035163564
cg24137268	-0.623140793	3.2649712	-4.565103569	0.000134751	0.035163564
cg12666469	-0.522407638	3.641274477	-4.565102934	0.000134751	0.035163564
cg25797694	-0.872205433	2.542963394	-4.565101257	0.000134752	0.035163564
cg01415058	-0.470346353	3.07226803	-4.564987198	0.00013479	0.035163564
cg24307636	-0.38660205	-0.531078408	-4.564887909	0.000134824	0.035163564
cg21329470	-0.492148645	3.996645315	-4.564630723	0.000134911	0.035163564
cg21225319	-0.715289262	-3.314712863	-4.56444458	0.000134974	0.035163564
cg02178784	-0.61622881	1.002564338	-4.564245913	0.000135041	0.035163564
cg08513075	-0.527950042	-0.785450783	-4.564159255	0.00013507	0.035163564
cg25223390	-0.515967043	3.602280018	-4.564124684	0.000135082	0.035163564
cg13519549	-0.616750362	2.93308792	-4.564040348	0.00013511	0.035163564
cg04521027	-0.490986793	3.731559816	-4.563866414	0.000135169	0.035163564
cg08837449	-0.690442379	-0.887515235	-4.563750462	0.000135209	0.035163564
cg02794358	-0.81246794	-0.216734917	-4.563560871	0.000135273	0.035163564
cg03199390	-0.639010515	2.506076727	-4.563493641	0.000135296	0.035163564
cg16577533	-0.539550628	2.683038034	-4.563248491	0.000135379	0.035163564
ch.11.94541071R	0.606545946	-4.039148559	4.563173528	0.000135404	0.035163564
cg04600961	-0.797853263	2.557230062	-4.563097408	0.00013543	0.035163564
cg12515229	-0.447547711	3.655932458	-4.562960106	0.000135476	0.035163564
cg18561658	-0.606997394	3.312761822	-4.562930544	0.000135487	0.035163564
cg09672954	-0.847200278	0.243881618	-4.562568762	0.000135609	0.035163564

cg06895831	-0.592449137	2.258668172	-4.562508373	0.00013563	0.035163564
cg12365411	-0.55170073	2.651705169	-4.562444069	0.000135652	0.035163564
cg07160420	-0.428221027	0.073790236	-4.562245584	0.000135719	0.035163564
cg21386549	-0.569201055	3.015582866	-4.562177722	0.000135742	0.035163564
cg10946681	-0.484609409	3.172391273	-4.562094905	0.00013577	0.035163564
cg03468827	-0.575860958	-2.207118489	-4.561933717	0.000135825	0.035163564
cg23322812	-0.602760043	0.554661561	-4.561799099	0.000135871	0.035163564
cg26194843	-0.603996501	3.283191434	-4.561704816	0.000135903	0.035163564
cg19312076	-0.523689338	3.605474424	-4.561690985	0.000135908	0.035163564
cg02238031	-0.627818285	2.711249706	-4.561641692	0.000135925	0.035163564
cg13691257	-0.726162038	1.685165747	-4.561560502	0.000135952	0.035163564
cg09311056	-0.489109493	3.438314373	-4.561271365	0.000136051	0.035163564
cg15411952	-0.583898252	2.424358788	-4.561104903	0.000136108	0.035163564
cg00520380	-0.424431851	0.759255894	-4.560845226	0.000136196	0.035163564
cg00336791	-0.52913714	3.969800441	-4.560767905	0.000136223	0.035163564
cg00973424	-0.511915485	3.471753687	-4.560642174	0.000136265	0.035163564
cg11612140	-0.549088856	2.533910969	-4.56062697	0.000136271	0.035163564
cg24524029	-0.432659848	-0.560004116	-4.560548314	0.000136298	0.035163564
cg25960218	-0.764691165	2.050382829	-4.56040234	0.000136347	0.035163564
cg12688894	0.460263402	1.938257882	4.560397212	0.000136349	0.035163564
cg25712611	-0.653700194	3.176649063	-4.56030014	0.000136382	0.035163564
cg15721102	-0.605777019	2.875775645	-4.560249295	0.0001364	0.035163564
cg12776896	-0.528824736	1.010730283	-4.560223364	0.000136409	0.035163564
cg20286147	0.453097972	2.599091374	4.559913311	0.000136515	0.035164507
cg17678297	-0.512627203	-0.533550527	-4.559885518	0.000136524	0.035164507
cg26354926	-0.668504945	3.343832285	-4.559565219	0.000136634	0.035164507
cg23868250	-0.567050724	3.088645123	-4.559421062	0.000136683	0.035164507
cg18806815	-0.782624201	2.025488428	-4.558969116	0.000136838	0.035164507
cg20643991	-0.509378498	-0.36758483	-4.558629014	0.000136954	0.035164507
cg16398289	-0.550757098	4.03275866	-4.558600004	0.000136964	0.035164507
cg00767573	-0.78280317	2.193507801	-4.55859006	0.000136968	0.035164507
cg25886665	-0.643975349	3.546949546	-4.558412397	0.000137029	0.035164507
cg14757199	-0.583257316	3.296958706	-4.558341366	0.000137053	0.035164507
cg23920293	-0.661164317	2.628711675	-4.557985939	0.000137175	0.035164507
cg14610335	-0.51277129	-1.255380939	-4.557976271	0.000137179	0.035164507
cg05300553	-0.689940232	2.8286846	-4.557955702	0.000137186	0.035164507
cg14474293	-0.368077007	3.349075878	-4.557790808	0.000137242	0.035164507
cg13045635	-0.75672081	3.237898247	-4.55742296	0.000137369	0.035164507
cg18382401	-0.645257064	3.090231532	-4.557112431	0.000137476	0.035164507
cg08773757	-0.476170194	3.564438329	-4.55701353	0.00013751	0.035164507
cg10301445	0.429874984	1.660264454	4.55699378	0.000137517	0.035164507
cg13276981	-0.615003313	3.715180443	-4.556956119	0.00013753	0.035164507
cg18356477	-0.553444354	3.131428379	-4.556854306	0.000137565	0.035164507
cg00339695	-0.517933662	1.056284973	-4.556587277	0.000137657	0.035164507
cg17449339	-0.816585215	0.643955915	-4.556564276	0.000137665	0.035164507
cg13975336	-0.540048617	-2.76367274	-4.55653112	0.000137676	0.035164507
cg07834276	-0.667273579	2.842290925	-4.556401416	0.000137721	0.035164507

cg14040897	-0.506133887	3.605339581	-4.555928161	0.000137884	0.035164507
cg07815324	-0.553967979	3.060101562	-4.555853227	0.00013791	0.035164507
cg03448286	-0.649280755	3.015728213	-4.555771854	0.000137938	0.035164507
cg16435343	-0.496952504	-0.018843744	-4.555744528	0.000137948	0.035164507
cg08340378	-0.576404893	2.680439522	-4.555603761	0.000137996	0.035164507
cg08510995	-0.617401845	2.653578908	-4.555545394	0.000138017	0.035164507
cg14188346	-0.422898279	0.056129274	-4.555524346	0.000138024	0.035164507
cg08523780	-0.515399442	3.793405651	-4.555509173	0.000138029	0.035164507
cg21632230	0.67905951	1.291209059	4.555426347	0.000138058	0.035164507
cg23878490	-0.491359821	2.892614451	-4.555304239	0.0001381	0.035164507
cg07177661	-0.557092065	-0.387162921	-4.554736608	0.000138296	0.035193301
cg20928596	-0.910647139	2.690650615	-4.554690673	0.000138312	0.035193301
cg10105341	-0.469817178	3.00332606	-4.553996202	0.000138553	0.035228127
cg09941405	-0.545051462	3.160362336	-4.553883681	0.000138592	0.035228127
cg12151614	-0.523454549	4.07998644	-4.553473698	0.000138735	0.035228127
cg03716161	-0.43533956	-1.978272311	-4.553263689	0.000138808	0.035228127
cg20585916	-0.478134859	3.706418686	-4.553234306	0.000138818	0.035228127
cg08320989	-0.744200007	-0.152230012	-4.553211198	0.000138826	0.035228127
cg12958443	-0.420699881	-0.491157406	-4.553077575	0.000138872	0.035228127
cg17562277	-0.573049454	2.890944019	-4.552963293	0.000138912	0.035228127
cg03092494	-0.567434323	3.517178158	-4.552832746	0.000138958	0.035228127
cg21129041	-0.495602507	3.728677046	-4.55282564	0.00013896	0.035228127
cg02938774	-0.825430311	2.428676737	-4.552259225	0.000139157	0.035228127
cg03451204	-0.404101219	-1.03408745	-4.552129937	0.000139203	0.035228127
cg02189048	-0.348302883	-0.437333308	-4.552071772	0.000139223	0.035228127
cg06587664	-0.908101216	2.967841193	-4.5518465	0.000139301	0.035228127
cg18383585	-0.687342376	-1.704575403	-4.551747396	0.000139336	0.035228127
cg01445952	-0.51242854	-0.906998444	-4.551741849	0.000139338	0.035228127
cg02208249	-0.697016139	-2.115788714	-4.551628807	0.000139377	0.035228127
cg24707189	-0.473357361	4.245382306	-4.551582393	0.000139394	0.035228127
cg04667114	-0.71570519	-2.11211049	-4.551580998	0.000139394	0.035228127
cg27498782	-0.551682793	2.866616952	-4.551052879	0.000139579	0.035244486
cg05943455	-0.41848334	-1.277948553	-4.550738562	0.000139689	0.035244486
cg12412070	-0.517192724	-1.022834952	-4.550634031	0.000139725	0.035244486
cg08005915	-0.769587263	1.91895221	-4.55052881	0.000139762	0.035244486
cg17324671	-0.693119995	2.686747824	-4.550302653	0.000139841	0.035244486
cg25397519	-0.854286298	2.708973869	-4.550028595	0.000139937	0.035244486
cg09410612	-0.607692837	0.206235412	-4.549944259	0.000139967	0.035244486
cg26831709	-0.438939934	4.229485677	-4.549746908	0.000140036	0.035244486
cg04944654	-0.435148898	3.440125238	-4.549242016	0.000140213	0.035244486
cg02313013	-0.518744431	-0.580851276	-4.549104464	0.000140262	0.035244486
cg23141745	-0.479871123	4.395669159	-4.549020049	0.000140291	0.035244486
cg23803981	-0.503451492	0.174464357	-4.548992724	0.000140301	0.035244486
cg16420720	-0.727166225	2.856802679	-4.548771326	0.000140379	0.035244486
cg12829524	-0.519973643	3.33539381	-4.548700485	0.000140404	0.035244486
cg13870198	0.573861555	2.202286749	4.548547756	0.000140457	0.035244486
cg03097169	-0.62472899	-3.079933079	-4.548518908	0.000140468	0.035244486

cg27255388	0.933705416	3.137521047	4.548432071	0.000140498	0.035244486
cg10970856	0.867808998	-4.08767968	4.548389296	0.000140513	0.035244486
cg08789421	-0.531677959	-0.576773544	-4.548208419	0.000140577	0.035244486
cg02554737	-0.632860385	2.918619765	-4.548205206	0.000140578	0.035244486
cg17231980	-0.526597482	-0.352379885	-4.548135773	0.000140603	0.035244486
cg24085438	-0.55971072	2.918460978	-4.547862021	0.000140699	0.035244486
cg00377173	-0.476626832	-1.057946326	-4.547752494	0.000140738	0.035244486
cg04265429	-0.607183173	3.196449193	-4.547709336	0.000140753	0.035244486
cg23674806	-0.776129058	2.03802747	-4.547644396	0.000140776	0.035244486
cg26621192	-0.465379465	3.373524677	-4.547593369	0.000140794	0.035244486
cg02979010	-0.551971523	3.335051148	-4.547279082	0.000140905	0.035244486
cg22315746	-0.535745465	-0.156013944	-4.547193889	0.000140935	0.035244486
cg12262409	-0.453565515	0.123758804	-4.54706032	0.000140982	0.035244486
cg08108165	-0.828800927	-1.914129009	-4.547057626	0.000140983	0.035244486
cg06640662	-0.859125689	2.034933663	-4.546764011	0.000141087	0.035244486
cg12114513	-0.576662704	3.78100173	-4.546616194	0.000141139	0.035244486
cg20708103	-0.441016572	3.263960346	-4.54605891	0.000141336	0.035244486
cg03582371	-0.448244317	0.306391277	-4.545755483	0.000141443	0.035244486
cg10831366	-0.483419447	4.141382517	-4.545705859	0.000141461	0.035244486
cg19680308	-0.614394391	3.237663244	-4.545552116	0.000141516	0.035244486
cg17064535	-0.597518275	3.067441962	-4.545341916	0.00014159	0.035244486
cg26417244	-0.465585828	3.928476213	-4.545330505	0.000141594	0.035244486
cg21830914	-0.768298288	2.942109496	-4.545310071	0.000141601	0.035244486
cg14593451	-0.668394538	2.921464768	-4.545200891	0.00014164	0.035244486
cg08021244	-0.554613378	3.190209519	-4.545032051	0.0001417	0.035244486
cg05266271	-0.449029605	3.494296291	-4.544864356	0.00014176	0.035244486
cg02401312	-0.631463739	3.163982294	-4.544667965	0.000141829	0.035244486
cg22962356	-0.494663812	3.285169526	-4.544576622	0.000141862	0.035244486
cg22463799	-0.758308178	3.154757294	-4.544545668	0.000141873	0.035244486
cg05684274	-0.630606827	2.355845544	-4.544521587	0.000141881	0.035244486
cg11912239	-0.440228666	-1.115026974	-4.54443065	0.000141914	0.035244486
cg05478885	-0.835110456	3.136182926	-4.544291065	0.000141963	0.035244486
cg24822465	-0.692708486	2.86936425	-4.544267423	0.000141972	0.035244486
cg04131403	-0.483856458	4.105566048	-4.544021729	0.000142059	0.035244486
cg04485399	-0.51056205	1.046775218	-4.543963152	0.00014208	0.035244486
cg10897651	-0.484018753	0.431533924	-4.543831286	0.000142127	0.035244486
cg07665877	-0.416171932	3.977653576	-4.543603177	0.000142208	0.035244486
cg05165779	-0.880931364	1.248702073	-4.543534512	0.000142233	0.035244486
cg16042652	0.516114733	3.118388924	4.543512121	0.000142241	0.035244486
cg13077413	-0.522019699	-1.920273996	-4.543499882	0.000142245	0.035244486
cg12559119	-0.464437038	-0.331420273	-4.543260887	0.00014233	0.035253265
cg11583791	-0.653935131	3.448703756	-4.542830689	0.000142484	0.035273663
cg05677756	-0.449121141	4.195672707	-4.542703881	0.000142529	0.035273663
cg02534459	-0.529686769	3.134008934	-4.542323785	0.000142665	0.035273663
cg20251591	-0.411614806	3.857952383	-4.54223291	0.000142697	0.035273663
cg27360305	-0.63326397	0.209267869	-4.542119811	0.000142738	0.035273663
cg18189173	-0.52501502	-0.342010944	-4.542043684	0.000142765	0.035273663

cg11128944	-0.495441502	3.986358388	-4.541930058	0.000142806	0.035273663
cg19147499	-0.637318696	2.245100504	-4.541901713	0.000142816	0.035273663
cg00920864	-0.800500963	2.024151795	-4.541775946	0.000142861	0.035273663
cg02234400	-0.531439512	1.856333662	-4.541410624	0.000142992	0.035283679
cg26552233	-0.529249254	3.560482528	-4.541064858	0.000143116	0.035283679
cg08741063	-0.634469073	3.33126039	-4.540973413	0.000143148	0.035283679
cg21272827	-0.670026055	2.992003084	-4.540956405	0.000143154	0.035283679
cg24311237	-0.557306879	3.826698498	-4.540846991	0.000143194	0.035283679
cg18994940	-0.55941182	3.559854687	-4.540828767	0.0001432	0.035283679
cg08061218	-0.533166265	3.143309059	-4.540607822	0.000143279	0.035290941
cg02489677	-0.482092411	0.190878024	-4.540027638	0.000143488	0.035324667
cg11853366	-0.876845263	1.254929447	-4.539949093	0.000143516	0.035324667
cg20989968	-0.733540291	2.729047746	-4.539561512	0.000143656	0.035332555
cg14096799	-0.654088885	2.734131628	-4.539548425	0.00014366	0.035332555
cg26455970	-0.508048349	-0.627501532	-4.539444132	0.000143698	0.035332555
cg20869070	0.489159943	3.193468071	4.53926644	0.000143762	0.035336023
cg07568716	0.51520906	-4.516156001	4.538745277	0.00014395	0.03536992
cg23166710	-0.644563789	3.169747919	-4.538446533	0.000144057	0.03538413
cg01868577	-0.581211764	-0.316751149	-4.538147077	0.000144166	0.035398413
cg10365362	-0.562152608	3.377164768	-4.537944056	0.000144239	0.035404152
cg15523980	-0.629408821	0.707695719	-4.537613605	0.000144358	0.035421202
cg04500740	-0.651550864	3.784405849	-4.537083109	0.00014455	0.035443294
cg25451194	-0.479755037	-1.368030462	-4.537022007	0.000144572	0.035443294
cg21272363	-0.5662731	3.278249331	-4.536950209	0.000144598	0.035443294
cg26905406	-0.438591073	4.118799186	-4.536789618	0.000144657	0.035445281
cg20380380	-0.565482585	3.57451314	-4.536619818	0.000144718	0.035445281
cg06701210	-0.606888662	-0.348709461	-4.536513724	0.000144757	0.035445281
cg20237731	-0.565392973	3.168485699	-4.536069891	0.000144918	0.035452779
cg07632208	-0.844065119	2.939300658	-4.535962504	0.000144957	0.035452779
cg25680015	-0.51662081	3.481868145	-4.535904733	0.000144978	0.035452779
cg24495004	-0.585877109	2.153145781	-4.535761136	0.00014503	0.035452779
cg01206640	0.46974878	1.875624359	4.535704628	0.00014505	0.035452779
cg19078851	-0.548977634	-1.328646061	-4.535466487	0.000145137	0.035452779
cg18788116	-0.468282853	3.915270277	-4.535217491	0.000145228	0.035452779
cg05962763	-0.690251952	2.322922322	-4.535154689	0.00014525	0.035452779
cg00415971	-0.576991457	-1.213565385	-4.534957836	0.000145322	0.035452779
cg09637100	-0.541963681	1.006381101	-4.534938263	0.000145329	0.035452779
cg06143663	-0.532768935	2.908910174	-4.534914566	0.000145338	0.035452779
cg06678477	-0.60637294	3.67442741	-4.534736892	0.000145403	0.035456355
cg23760494	-0.598986862	2.553309531	-4.534544958	0.000145473	0.035461202
cg12990174	-0.502051682	3.140600805	-4.534304543	0.00014556	0.035468636
cg20322104	-0.804740976	1.811876383	-4.534113689	0.00014563	0.035468636
cg17055895	-0.51816645	0.517733474	-4.534049468	0.000145653	0.035468636
cg19194095	-0.523682196	3.645449377	-4.53376241	0.000145758	0.035481957
cg11750851	-0.873092028	-2.489051226	-4.533300426	0.000145927	0.035504632
cg06663283	-0.399327368	3.887644145	-4.533188612	0.000145968	0.035504632
cg00921219	-0.54098258	3.152707278	-4.532848584	0.000146092	0.035504632

cg26192279	-0.534838484	3.776060312	-4.532802054	0.000146109	0.035504632
cg13065203	-0.542372614	2.933238026	-4.532422465	0.000146248	0.035504632
cg18332814	-0.790583401	-1.004665514	-4.53236347	0.00014627	0.035504632
cg17940200	-0.532058902	4.031917614	-4.532353782	0.000146273	0.035504632
cg09546889	-0.463448078	3.563797876	-4.532347218	0.000146276	0.035504632
cg11646240	-0.517382131	-0.411367337	-4.532274915	0.000146302	0.035504632
cg20654611	-0.732087308	2.720106287	-4.532135097	0.000146354	0.035504908
cg17212127	-0.986062421	-2.559910424	-4.531924735	0.000146431	0.035511319
cg04001997	-0.77228177	2.18408809	-4.531789751	0.00014648	0.035511319
cg17756860	-0.435940745	3.564396652	-4.531494026	0.000146589	0.035525483
cg24829718	-0.479255806	-0.763386871	-4.531261286	0.000146674	0.035534048
cg06708315	-0.503434572	-0.984224117	-4.530923213	0.000146799	0.035537436
cg09723536	-0.608380776	3.726117511	-4.530913312	0.000146802	0.035537436
cg19002100	-0.626606761	3.237763415	-4.530692788	0.000146883	0.035537436
cg17830370	-0.638861941	3.074927878	-4.530677609	0.000146889	0.035537436
cg18571447	0.96546431	-3.274283759	4.530518088	0.000146948	0.035539504
cg17176609	-0.80253269	-1.584251394	-4.53015169	0.000147083	0.035560004
cg00765623	0.699079186	3.378359073	4.529636929	0.000147272	0.035592181
cg01686960	-0.704019994	2.975703907	-4.529390002	0.000147364	0.035592181
cg16396822	-0.48853056	-0.419689229	-4.529382243	0.000147366	0.035592181
cg19878971	-0.499889457	3.60584492	-4.529178411	0.000147442	0.035598226
cg09342185	-0.791782008	3.417145759	-4.528924451	0.000147536	0.035608747
cg11149952	-0.86702045	0.126314197	-4.528451139	0.000147711	0.035630266
cg20243900	-0.724162267	-0.305884228	-4.528411451	0.000147725	0.035630266
cg15884789	0.545723026	1.28085917	4.527923041	0.000147906	0.035661751
cg01946401	-0.491467718	3.399704245	-4.527428958	0.000148089	0.035682728
cg02636380	-0.677727228	2.874190122	-4.527416739	0.000148094	0.035682728
cg12374672	-0.422525542	-0.559379402	-4.527279613	0.000148145	0.035682852
cg08419507	-0.587376075	3.182944225	-4.526704686	0.000148358	0.035718352
cg09499849	-0.610195447	-1.832146703	-4.526611409	0.000148393	0.035718352
cg09423651	-0.916199599	0.966866078	-4.525974339	0.00014863	0.035743589
cg02858706	-0.580311307	2.613973382	-4.525905485	0.000148656	0.035743589
ch.6.122375039R	-0.737203412	-1.585111841	-4.52585753	0.000148674	0.035743589
cg06507137	-0.749004121	1.608004611	-4.525787413	0.0001487	0.035743589
cg06955182	-0.549086797	3.444751823	-4.525399098	0.000148844	0.035766245
cg14688588	-0.424014753	-0.695473925	-4.524779312	0.000149076	0.035776547
cg14737571	-0.758300529	-0.454174527	-4.524531441	0.000149168	0.035776547
cg27426824	-0.708758427	1.774858785	-4.524510714	0.000149176	0.035776547
cg20384211	-1.022041698	0.805611199	-4.52450746	0.000149177	0.035776547
cg25307288	-0.587913921	2.811887771	-4.524193833	0.000149295	0.035776547
cg03703911	-0.495015586	3.190008397	-4.524036946	0.000149353	0.035776547
cg21653097	-0.637236028	3.079474591	-4.523885104	0.00014941	0.035776547
cg07545014	-0.585789012	-2.170296233	-4.523865872	0.000149417	0.035776547
cg14220104	0.501272045	2.877542001	4.523525248	0.000149545	0.035776547
cg16726875	-0.615908674	2.951362249	-4.523452572	0.000149572	0.035776547
cg10973093	-0.598246037	1.338787425	-4.523240574	0.000149652	0.035776547
cg26339919	-0.428572819	-0.183145799	-4.52319835	0.000149667	0.035776547

cg02438950	-0.692935393	-0.319634742	-4.523092754	0.000149707	0.035776547
cg01363334	-0.55783943	2.894743779	-4.522962574	0.000149756	0.035776547
cg02896808	-0.742378906	1.744046258	-4.522756622	0.000149833	0.035776547
cg09848943	-1.018653318	-2.886501719	-4.522612046	0.000149887	0.035776547
cg26960452	-0.559637915	2.878850963	-4.522461855	0.000149944	0.035776547
cg26032021	-0.54752058	2.846554339	-4.52239122	0.00014997	0.035776547
cg16452467	-0.734478109	0.788864533	-4.522311803	0.00015	0.035776547
cg18792385	-0.671256311	2.826080429	-4.522302593	0.000150004	0.035776547
cg08717983	-0.555487521	3.577203972	-4.522281789	0.000150012	0.035776547
cg04267605	-0.612405015	3.465678134	-4.522260336	0.00015002	0.035776547
cg21083928	-0.51897756	3.522030398	-4.522076945	0.000150089	0.035776547
cg10835876	-0.540600139	3.436032296	-4.52204805	0.000150099	0.035776547
cg22007788	-0.550291716	0.038737137	-4.52172661	0.00015022	0.035793329
cg10615150	-0.39698491	-1.150424344	-4.521049073	0.000150476	0.035825398
cg16593899	-0.496913562	-1.786629038	-4.520981936	0.000150501	0.035825398
cg27372537	-0.5136784	2.944608995	-4.520898911	0.000150532	0.035825398
cg26722544	-0.680841483	3.775877243	-4.520790985	0.000150573	0.035825398
cg15977148	-0.534646643	2.91640412	-4.520574551	0.000150655	0.035825398
cg07254166	-0.603473762	4.075014917	-4.520497773	0.000150684	0.035825398
cg23751564	-0.482574701	-0.007890694	-4.520430563	0.000150709	0.035825398
cg04329349	-0.467810418	-1.843074294	-4.519895161	0.000150911	0.035849971
cg23262914	-0.871194651	2.988229647	-4.519889122	0.000150914	0.035849971
cg22435764	-0.68163237	2.006984025	-4.519573239	0.000151033	0.035866329
cg15206889	-0.483607771	-0.82515946	-4.519310751	0.000151132	0.035868243
cg26637557	-0.691004555	3.223769272	-4.519145861	0.000151195	0.035868243
cg17171500	-0.505147695	3.886211914	-4.518806303	0.000151324	0.035868243
cg19004325	-0.506438488	3.911736912	-4.51870958	0.00015136	0.035868243
cg03367716	-0.628761315	0.281174822	-4.518660586	0.000151379	0.035868243
cg05375309	-0.539133694	2.666564112	-4.518616857	0.000151395	0.035868243
cg03822645	-0.447113908	0.17594494	-4.518616466	0.000151396	0.035868243
cg15551710	-0.478983519	0.080121745	-4.518478407	0.000151448	0.035868656
cg16362458	-0.478773607	3.431347413	-4.517653694	0.000151761	0.035905668
cg03401963	-0.386134755	3.596133305	-4.517652612	0.000151762	0.035905668
cg03101972	-0.894705526	2.619631769	-4.517572264	0.000151792	0.035905668
cg27636379	-0.46387559	3.091758759	-4.517533391	0.000151807	0.035905668
cg00190393	-0.587599083	2.525633878	-4.516651715	0.000152143	0.035943657
cg21921829	-0.773565752	2.530718573	-4.516598328	0.000152163	0.035943657
cg19317600	-0.731329566	-1.55094062	-4.516484037	0.000152207	0.035943657
cg04869336	-0.740581482	0.171609808	-4.516439564	0.000152224	0.035943657
cg17392044	-0.665066016	3.246781443	-4.516256732	0.000152293	0.035943657
cg26477387	-0.615067823	3.728403185	-4.515978149	0.0001524	0.035943657
cg15760630	-0.929575524	1.965610615	-4.515904597	0.000152428	0.035943657
cg02817143	-0.646033284	3.508813962	-4.515864731	0.000152443	0.035943657
cg12484453	-0.705576699	0.494629685	-4.51581225	0.000152463	0.035943657
cg15196139	-0.456361303	-1.791466424	-4.515739295	0.000152491	0.035943657
cg20067415	-0.540625363	3.401535511	-4.515648183	0.000152526	0.035943657
cg22645421	-0.534781515	3.682996986	-4.515426457	0.00015261	0.035951671

cg23289954	-0.606831229	2.924021934	-4.51492108	0.000152804	0.035985257
cg13295333	-0.444182331	3.693713783	-4.514500123	0.000152965	0.036011261
cg07379779	-0.478784349	3.114057738	-4.513755955	0.000153251	0.036045866
cg22274628	-0.575401633	3.58056234	-4.513745018	0.000153255	0.036045866
cg04307655	-0.633699839	2.86598279	-4.513527045	0.000153339	0.036045866
cg15824600	-0.454402353	-1.633082922	-4.51329791	0.000153427	0.036045866
cg25619136	-0.791954952	2.024670784	-4.513191147	0.000153468	0.036045866
cg06125456	-0.684434291	2.880752536	-4.512949471	0.000153561	0.036045866
cg13529479	-0.479965534	-0.762575175	-4.512880816	0.000153587	0.036045866
cg03065308	-0.562684536	-0.237713125	-4.512846583	0.0001536	0.036045866
cg01861019	-0.575900188	0.328289623	-4.512600801	0.000153695	0.036045866
cg13427594	-1.278478474	1.91606676	-4.512593836	0.000153697	0.036045866
cg19618555	-0.959185425	2.095687503	-4.512548427	0.000153715	0.036045866
cg09368302	-0.772005652	1.986786855	-4.512138608	0.000153873	0.036045866
cg06698432	-0.672701478	1.008678739	-4.511934158	0.000153952	0.036045866
cg09388871	-0.691160971	1.727943599	-4.511909717	0.000153961	0.036045866
cg00263248	-0.604890361	-1.013032962	-4.511896094	0.000153966	0.036045866
cg20070619	-0.554261724	3.748074912	-4.511880546	0.000153972	0.036045866
cg12156155	-0.427582195	0.242692194	-4.511793062	0.000154006	0.036045866
cg12292630	-0.521886125	3.856477329	-4.511639931	0.000154065	0.036045866
cg01427300	0.641348008	3.263668266	4.511490057	0.000154123	0.036045866
cg20838818	-0.705665133	1.371861603	-4.511445044	0.000154141	0.036045866
cg15328131	-0.729425875	-1.302539646	-4.51121156	0.000154231	0.036045866
cg10151019	-0.582567784	3.603833412	-4.511209371	0.000154232	0.036045866
cg19116349	-0.880189973	-0.355371177	-4.510861097	0.000154366	0.036057038
cg04551025	-0.547969363	3.404634897	-4.510580658	0.000154475	0.036057038
cg00725356	-0.511418925	-2.369464982	-4.510572901	0.000154478	0.036057038
cg02022431	-0.440667222	3.806991384	-4.510297454	0.000154584	0.036057038
cg02119938	-0.524313582	-1.565558559	-4.510280496	0.000154591	0.036057038
cg15301871	-0.424031768	4.144750239	-4.5102506	0.000154603	0.036057038
cg15146441	-0.753623926	0.55865401	-4.510165034	0.000154636	0.036057038
cg06670786	-0.514800561	3.290598658	-4.509276271	0.00015498	0.036067244
cg11828871	-0.507911065	3.824022837	-4.50925251	0.00015499	0.036067244
cg01907042	-0.539351678	4.075157447	-4.509200733	0.00015501	0.036067244
cg01439560	-0.430304222	2.782253063	-4.509102054	0.000155048	0.036067244
cg22645624	-0.821212122	2.226861048	-4.509002648	0.000155087	0.036067244
cg23024358	-0.652914847	2.700921019	-4.508780757	0.000155173	0.036067244
cg15482000	-0.630819235	3.285229799	-4.508745697	0.000155187	0.036067244
cg06770076	-0.404976105	3.789006146	-4.508689653	0.000155208	0.036067244
cg22011581	-0.698498341	3.525061263	-4.508509844	0.000155278	0.036067244
cg00084432	-0.69961893	-0.028609025	-4.508309279	0.000155356	0.036067244
cg16931825	-1.042197253	-4.77154792	-4.508218421	0.000155392	0.036067244
cg05181098	-0.534821787	3.334903274	-4.508026724	0.000155466	0.036067244
cg20111163	-0.854536648	2.27151671	-4.507942696	0.000155499	0.036067244
cg22251211	-0.477576932	0.917460721	-4.507785344	0.00015556	0.036067244
cg07787563	-0.466617742	3.871464429	-4.507781718	0.000155562	0.036067244
cg27506390	0.594191541	1.485223182	4.50747894	0.00015568	0.036067244

cg23418383	-0.594482167	3.244864224	-4.507423169	0.000155702	0.036067244
cg03400587	-0.607649205	3.367008054	-4.507422445	0.000155702	0.036067244
cg13254203	-0.395219814	3.874578965	-4.507301713	0.000155749	0.036067244
cg08628459	-0.486361825	4.027340091	-4.507133449	0.000155815	0.036067244
cg23393139	-0.486566297	3.9721766	-4.506988172	0.000155871	0.036067244
cg04728774	-0.96279272	3.763207495	-4.506880135	0.000155914	0.036067244
cg10065739	-0.674118538	0.795722929	-4.506675227	0.000155994	0.036067244
cg03683612	-0.713976564	2.927546335	-4.506572677	0.000156034	0.036067244
cg16692561	-0.49364537	3.70130152	-4.506428549	0.00015609	0.036067244
cg06674089	-0.801150469	1.317268413	-4.506340156	0.000156125	0.036067244
cg14201196	-0.846769249	0.835892418	-4.506291505	0.000156144	0.036067244
cg05146966	-0.669316174	3.430298642	-4.506232126	0.000156167	0.036067244
cg18768862	-0.496493273	-1.671859104	-4.505852075	0.000156316	0.036067244
cg02878831	-0.549543443	-0.340556391	-4.505739017	0.00015636	0.036067244
cg15721802	-0.612617288	2.917670553	-4.505616288	0.000156408	0.036067244
cg27525902	-0.782479116	1.222338188	-4.505611023	0.00015641	0.036067244
cg02926868	-0.380911827	-1.565191623	-4.505550305	0.000156434	0.036067244
cg12688965	-0.645258155	-1.236746154	-4.505509788	0.00015645	0.036067244
cg13470063	-0.416450431	0.271881641	-4.505480414	0.000156461	0.036067244
cg24258234	-0.601454151	-0.807999524	-4.50532864	0.000156521	0.036067488
cg11473757	-0.459769302	4.26808841	-4.505218052	0.000156564	0.036067488
cg01222719	-0.747006271	0.823094172	-4.504821118	0.00015672	0.03606889
cg24474852	-0.437220126	-1.917371573	-4.504782563	0.000156735	0.03606889
cg23705245	-0.457868375	-0.738693837	-4.504618025	0.0001568	0.03606889
cg06572713	-0.499564432	-1.500256587	-4.504290225	0.000156929	0.03606889
cg20264491	-0.784857798	2.192377572	-4.504286924	0.00015693	0.03606889
cg06534197	-0.466403239	0.844276555	-4.504034299	0.000157029	0.03606889
cg09295252	-0.642449765	-2.515276986	-4.504000741	0.000157043	0.03606889
cg05389848	-0.619966288	3.42706597	-4.50396866	0.000157055	0.03606889
cg14040679	-0.466673789	0.192109273	-4.503715189	0.000157155	0.03606889
cg13203009	-0.831804507	2.194732146	-4.50333077	0.000157306	0.03606889
cg10747963	-0.498639805	-0.244748582	-4.503309964	0.000157315	0.03606889
cg23613143	-0.584999362	-2.039349342	-4.503227023	0.000157347	0.03606889
cg24067952	0.429125752	2.417902538	4.5031153	0.000157391	0.03606889
cg17075155	-0.47974519	-0.497255578	-4.503039879	0.000157421	0.03606889
cg27204197	-0.42660829	0.048814651	-4.502979048	0.000157445	0.03606889
cg00241097	-0.668740474	2.845238373	-4.502749749	0.000157536	0.03606889
cg01278508	0.428878067	1.949425108	4.502741758	0.000157539	0.03606889
cg08123100	-0.43328461	3.643499872	-4.502730966	0.000157543	0.03606889
cg06772933	-0.746034334	0.869698822	-4.502702159	0.000157554	0.03606889
cg07765328	-0.548549758	3.581756881	-4.502615173	0.000157589	0.03606889
cg15642290	-0.509938778	4.125600028	-4.502393923	0.000157676	0.036072102
cg17848900	-0.919555314	2.142596879	-4.502321815	0.000157705	0.036072102
cg01409498	-0.583445032	3.232135849	-4.501486584	0.000158035	0.036117931
cg19442437	-0.45780184	3.214925987	-4.501466322	0.000158043	0.036117931
cg08399422	-0.491813167	2.810586994	-4.501347524	0.00015809	0.036117931
cg07558672	-0.545176242	-0.644077635	-4.501234915	0.000158135	0.036117931

cg25242729	-0.609895185	1.537215076	-4.501136	0.000158174	0.036117931
cg00203698	-0.584505387	0.227753196	-4.501042643	0.000158211	0.036117931
cg05630996	-0.423898994	3.419724838	-4.500863702	0.000158282	0.036122487
cg19355763	-0.491067935	3.668035204	-4.500627053	0.000158376	0.036125131
cg17140208	-0.835414184	2.924108736	-4.500577432	0.000158395	0.036125131
cg07887753	-0.558322248	3.639980179	-4.500164259	0.00015856	0.036135026
cg13231791	-0.782427115	2.673341764	-4.500105144	0.000158583	0.036135026
cg12697352	-0.449930324	3.674762242	-4.500082842	0.000158592	0.036135026
cg23380274	-0.59414579	2.963032383	-4.499912654	0.00015866	0.036138814
cg13170462	-0.416618989	4.202958132	-4.499748029	0.000158725	0.036142103
cg18567268	-0.539503242	-0.454535557	-4.499483739	0.00015883	0.036154423
cg26575140	-0.46541733	4.180033451	-4.499045866	0.000159004	0.036166556
cg02758612	-1.317328737	-4.495075663	-4.498859879	0.000159079	0.036166556
cg19425477	0.644040701	3.565453915	4.498789146	0.000159107	0.036166556
cg07616866	-0.571309001	4.173782363	-4.498766554	0.000159116	0.036166556
cg06677621	-0.45457631	4.057606884	-4.498547397	0.000159203	0.036166556
cg25605773	-0.516848381	-0.04567698	-4.498067016	0.000159395	0.036166556
cg22483175	-0.641315929	3.414131349	-4.498049365	0.000159402	0.036166556
cg02319946	-0.728594035	3.350943536	-4.497957282	0.000159439	0.036166556
cg06214217	-0.847706707	2.328138609	-4.497932328	0.000159449	0.036166556
cg09301445	0.620701108	1.373775469	4.497781171	0.000159509	0.036166556
cg05064936	0.54497334	2.709588226	4.497533131	0.000159608	0.036166556
cg22954906	-0.517773029	0.377376965	-4.497030193	0.00015981	0.036166556
cg13600494	-0.596356572	3.12787072	-4.496836403	0.000159887	0.036166556
cg15004860	-0.443848163	0.400384026	-4.496788246	0.000159907	0.036166556
cg20050420	-0.712003222	1.768255654	-4.496653849	0.00015996	0.036166556
cg21829255	-0.508745906	3.339413534	-4.496400657	0.000160062	0.036166556
cg24992741	-0.713455403	2.693668746	-4.496386116	0.000160068	0.036166556
cg13673570	-0.376035478	-2.211565765	-4.496259185	0.000160119	0.036166556
cg14583414	-0.643093587	2.550291757	-4.496239588	0.000160126	0.036166556
cg21735808	-0.75716586	2.682189068	-4.496238512	0.000160127	0.036166556
cg12209861	-0.522934705	3.862388327	-4.496064159	0.000160197	0.036166556
cg22424122	-0.71311166	1.88909817	-4.49599515	0.000160225	0.036166556
cg13346940	-0.371156595	0.503591917	-4.495929774	0.000160251	0.036166556
cg01839565	-0.518897683	-1.474722593	-4.495771146	0.000160315	0.036166556
cg01131170	-0.46964952	0.029444591	-4.49576366	0.000160318	0.036166556
cg15941779	-0.516919077	3.856608357	-4.495749029	0.000160323	0.036166556
cg04169243	-0.467720413	3.842482396	-4.4956612	0.000160359	0.036166556
cg12707710	0.544321412	3.280124951	4.495596862	0.000160385	0.036166556
cg00750225	-0.648671884	-0.491803538	-4.495538699	0.000160408	0.036166556
cg10943980	-0.621411292	2.658399571	-4.495520947	0.000160415	0.036166556
cg00400217	-0.447115764	-0.93776739	-4.495329912	0.000160492	0.036172359
cg20883137	-0.566189384	3.733434725	-4.495095487	0.000160586	0.036176723
cg12534855	-0.727910885	1.984810908	-4.494833988	0.000160691	0.036176723
cg05544693	-0.926559971	2.735762391	-4.494706097	0.000160743	0.036176723
cg11251399	-0.606495439	4.230608007	-4.494650276	0.000160765	0.036176723
cg12890561	-0.758312729	2.979011914	-4.494647384	0.000160767	0.036176723

cg03495652	-0.802717709	1.614998567	-4.494208091	0.000160944	0.036179315
cg25529661	-0.483960008	4.190895065	-4.494151286	0.000160967	0.036179315
cg21178143	-0.875247949	2.603226421	-4.494126525	0.000160977	0.036179315
cg01879365	-0.620041421	2.57044177	-4.494097932	0.000160988	0.036179315
cg26829261	-0.481132646	3.424254088	-4.493877292	0.000161077	0.036179315
cg13668359	-0.478521968	0.145645166	-4.493797305	0.000161109	0.036179315
cg18355353	-0.59077166	3.754268762	-4.493732332	0.000161136	0.036179315
cg04948529	-0.377449518	-0.995298928	-4.493594968	0.000161191	0.036180302
cg24161812	0.649006056	3.210092779	4.493171256	0.000161362	0.036199195
cg16443977	-0.673043436	1.594998778	-4.49304344	0.000161414	0.036199195
cg06424706	-0.649350043	2.120325625	-4.493007441	0.000161429	0.036199195
cg20186713	-0.541800567	2.636780188	-4.492846521	0.000161494	0.036202334
cg14535569	-0.686702471	1.334845993	-4.492670297	0.000161565	0.036202993
cg05876692	-0.599827794	3.182327362	-4.492473525	0.000161645	0.036202993
cg08129992	-0.435407568	4.012808721	-4.492371953	0.000161686	0.036202993
cg21643547	-0.513530513	-0.41468122	-4.4923344	0.000161701	0.036202993
cg11542316	-0.54617383	3.312042716	-4.491915816	0.000161871	0.03622341
cg19594258	-0.738892903	-0.216399764	-4.491842846	0.0001619	0.03622341
cg06002983	-0.659837083	2.602260362	-4.491731159	0.000161946	0.03622341
ch.12.2515819F	-0.561573331	-1.695635478	-4.49115652	0.000162179	0.036240067
cg22288769	-0.466141134	-1.622261903	-4.491069787	0.000162214	0.036240067
cg26409336	-0.565270634	0.827167828	-4.490849142	0.000162304	0.036240067
cg10223549	-1.005108009	1.608933572	-4.490455656	0.000162464	0.036240067
cg07394499	-0.551777903	-1.793600069	-4.490420552	0.000162478	0.036240067
cg26099738	-0.680588346	2.14705818	-4.490376512	0.000162496	0.036240067
cg10320449	-0.508230449	-0.197300701	-4.490257527	0.000162545	0.036240067
cg06391165	-0.413389383	3.740484747	-4.490246376	0.000162549	0.036240067
cg05928297	-0.585137573	3.15701753	-4.490222366	0.000162559	0.036240067
cg07214164	-0.516355157	-2.280469638	-4.490151179	0.000162588	0.036240067
cg10034028	-0.511210183	4.152914002	-4.490078964	0.000162617	0.036240067
cg03718416	-0.700957026	3.162113898	-4.490030228	0.000162637	0.036240067
cg01443071	-0.434083172	0.628412202	-4.489912831	0.000162685	0.036240067
cg01358940	-0.545832714	3.292298916	-4.48966802	0.000162785	0.036250249
cg26695291	-0.610003795	-2.449962763	-4.489549804	0.000162833	0.036250249
cg01923891	-0.50937166	0.960183291	-4.489192017	0.000162979	0.036259057
cg12053891	-0.542714227	-1.441296316	-4.489166814	0.00016299	0.036259057
cg17133295	-0.869855208	2.390346219	-4.489050534	0.000163037	0.036259057
cg23882086	-0.681565296	2.584958053	-4.488770892	0.000163151	0.036259057
cg13105046	-0.73336469	2.771963728	-4.48872082	0.000163172	0.036259057
cg10991809	-0.6217547	2.805669069	-4.488526213	0.000163251	0.036259057
cg08087714	-0.474697599	3.667892969	-4.488470032	0.000163274	0.036259057
cg12889998	-0.550809533	-0.935404875	-4.488450734	0.000163282	0.036259057
cg00808305	-0.536439308	-1.29211016	-4.48813588	0.000163411	0.036260326
cg10330410	-0.470297817	-0.394146619	-4.488101168	0.000163425	0.036260326
cg05473796	-0.799012466	1.99063793	-4.48806162	0.000163442	0.036260326
cg08245249	-0.488418571	3.767769932	-4.487669262	0.000163602	0.036284629
cg01669150	-0.808135277	-1.027866822	-4.487495066	0.000163674	0.036286952

cg11921583	-0.736124919	1.903270239	-4.487393891	0.000163715	0.036286952
cg19320294	-0.593356839	0.318399598	-4.486938965	0.000163902	0.036316976
cg14665062	-0.740756289	1.716238217	-4.486773966	0.00016397	0.036320633
cg02138992	-0.697427446	2.350792823	-4.48648688	0.000164088	0.036323262
cg05271660	-0.444715312	3.575046113	-4.486423452	0.000164114	0.036323262
cg01857262	0.764751485	3.071214589	4.486253219	0.000164184	0.036323262
cg26592432	-0.620585172	2.927679872	-4.486246219	0.000164187	0.036323262
cg25205993	-0.428975724	3.834362216	-4.486004535	0.000164286	0.03632431
cg10785155	-0.536010127	3.320460056	-4.485976518	0.000164298	0.03632431
cg10681093	-0.711198867	1.646427641	-4.48586097	0.000164345	0.03632431
cg11673660	-0.464098349	3.359480887	-4.485408258	0.000164532	0.036349035
cg06252055	-0.605780017	-0.192729329	-4.485340452	0.00016456	0.036349035
cg26992566	-0.431936321	1.053683866	-4.484793433	0.000164785	0.036387545
cg23330167	-0.470373839	-0.285199293	-4.484120468	0.000165064	0.036436818
cg00418037	0.582409397	2.218024099	4.483659849	0.000165254	0.036436818
cg14065930	-0.687445105	3.144364757	-4.483206586	0.000165442	0.036436818
cg04570669	-0.402287665	3.787901763	-4.483187346	0.00016545	0.036436818
cg16572410	-0.575799077	3.27967105	-4.483157345	0.000165462	0.036436818
cg19889291	-0.707036407	2.791504367	-4.483056016	0.000165504	0.036436818
cg00874605	-0.392847777	-0.496854544	-4.48292303	0.00016556	0.036436818
cg13292958	-0.510537438	3.328825341	-4.482718314	0.000165645	0.036436818
cg15859496	-0.776422811	-0.152947522	-4.482516566	0.000165728	0.036436818
cg04608253	-0.538875594	-0.639931306	-4.482464282	0.00016575	0.036436818
cg25634312	-0.518188235	3.578042023	-4.482455954	0.000165753	0.036436818
cg04566744	-0.659024684	1.249334132	-4.482315944	0.000165812	0.036436818
cg06041866	-0.591082406	2.180941269	-4.48221829	0.000165852	0.036436818
cg00108210	-0.607709637	1.644551469	-4.482164345	0.000165875	0.036436818
cg15005677	-0.557955575	-2.317412307	-4.482053613	0.000165921	0.036436818
cg01012080	-0.552632333	2.872368792	-4.482012045	0.000165938	0.036436818
cg14800929	-0.465978957	3.568336941	-4.481999741	0.000165943	0.036436818
cg08246644	-0.670864885	-1.55934956	-4.481977326	0.000165952	0.036436818
cg00514873	-0.414869301	4.372765191	-4.481896683	0.000165986	0.036436818
cg01820767	-0.739472615	2.118457801	-4.481660128	0.000166084	0.036447128
cg12308723	-0.552354098	3.291172566	-4.481505733	0.000166149	0.036449939
cg27344018	-0.693464159	3.179925161	-4.481304223	0.000166233	0.03645089
cg14129266	-0.616928125	2.828917635	-4.481248208	0.000166256	0.03645089
cg06316114	-0.510888996	3.672885687	-4.480960447	0.000166376	0.036465896
cg11523350	-0.548747305	-0.622593366	-4.480608035	0.000166523	0.036480762
cg24536410	0.60589266	-3.475947976	4.480499172	0.000166568	0.036480762
cg13308349	-0.632262417	2.988751852	-4.480427515	0.000166598	0.036480762
cg18162549	-0.763746764	2.39500016	-4.48029268	0.000166654	0.036481813
cg21034416	-0.40602412	-0.988804067	-4.479985615	0.000166783	0.036490874
cg15184749	-0.978031995	2.374378115	-4.47983921	0.000166844	0.036490874
cg03086078	-0.642661203	0.126069118	-4.479823753	0.00016685	0.036490874
cg26207102	-0.551439023	3.76920624	-4.479656786	0.00016692	0.036494877
cg20975401	-0.58861905	3.382613222	-4.479327304	0.000167058	0.03651375
cg02341953	-0.494649816	3.356703496	-4.478874678	0.000167248	0.036535432

cg19472302	-1.219149349	1.243638805	-4.478713614	0.000167315	0.036535432
cg01470839	-0.439871828	3.781329343	-4.478637864	0.000167347	0.036535432
cg25055392	-0.503012472	2.521291266	-4.478363983	0.000167462	0.036535432
cg21432801	-0.727913976	2.653625715	-4.478357703	0.000167464	0.036535432
cg09532575	-0.475228053	3.225229259	-4.478152228	0.000167551	0.036535432
cg13632520	-0.439495922	0.444639603	-4.478098222	0.000167573	0.036535432
cg05115552	-0.508573601	3.19394257	-4.478020209	0.000167606	0.036535432
cg21191843	-0.621629211	2.698432222	-4.477869805	0.000167669	0.036535432
cg11818555	-0.592051689	2.331912758	-4.477758666	0.000167716	0.036535432
cg14071799	-0.475629164	1.094270139	-4.477556607	0.000167801	0.036535432
cg25965156	-0.698990703	2.23470522	-4.477538594	0.000167808	0.036535432
cg21803927	-0.549484703	2.922136071	-4.477402991	0.000167865	0.036535432
cg00328411	-0.559483797	3.681269178	-4.477370195	0.000167879	0.036535432
cg21208580	-0.53627645	4.165952564	-4.47702414	0.000168025	0.036555895
cg06343288	-0.576111596	2.288703866	-4.476750962	0.00016814	0.036569695
cg25202000	-0.452295433	3.898307973	-4.476461833	0.000168262	0.036584966
cg03680898	-0.539610284	3.780526708	-4.476149814	0.000168393	0.036602346
cg25855710	-0.983246815	1.988129949	-4.475829191	0.000168529	0.036620527
cg23243995	-0.579259346	3.2934349	-4.475522774	0.000168658	0.036637417
cg18834156	-0.545592345	3.820603151	-4.475233475	0.000168781	0.036652746
cg12626381	-0.640948692	2.68928034	-4.474834985	0.000168949	0.036663053
cg03918426	-0.513586254	3.789809953	-4.474820553	0.000168955	0.036663053
cg04658278	-0.55827692	0.688703736	-4.474754402	0.000168983	0.036663053
cg21701806	-0.536234147	3.23353267	-4.474569088	0.000169062	0.036664773
cg03314137	-0.552414686	3.618210676	-4.474062665	0.000169276	0.036664773
cg03807590	-0.443401628	3.829653473	-4.473740444	0.000169413	0.036664773
cg05448077	-0.70154863	-1.359088386	-4.473623931	0.000169463	0.036664773
cg05984332	-0.478672596	3.082284771	-4.473518105	0.000169508	0.036664773
cg27051729	-0.542398254	2.758819078	-4.473397879	0.000169559	0.036664773
cg07954193	-0.586671078	2.300773514	-4.473310628	0.000169596	0.036664773
cg09771226	-0.491640311	-0.602013308	-4.473301874	0.000169599	0.036664773
cg03846960	-0.76999124	2.580007862	-4.473089526	0.00016969	0.036664773
cg19888881	-0.574557487	3.625993376	-4.47305853	0.000169703	0.036664773
cg09250897	-0.420394008	3.830292973	-4.47297745	0.000169737	0.036664773
cg07353119	-0.722410655	2.512197519	-4.472792468	0.000169816	0.036664773
cg14857399	-0.665894555	3.216068527	-4.47263726	0.000169882	0.036664773
cg17563818	-0.758043027	2.283729379	-4.472336865	0.00017001	0.036664773
cg14172090	-0.469194752	-0.600778112	-4.472214467	0.000170062	0.036664773
cg04702572	-0.425189628	3.860337706	-4.472143686	0.000170092	0.036664773
cg14152587	-0.619860073	2.933999086	-4.472109986	0.000170107	0.036664773
cg06583013	-0.86363899	1.736163188	-4.47190433	0.000170194	0.036664773
cg26273715	-0.631590875	3.447809746	-4.471869627	0.000170209	0.036664773
cg15390986	-0.63113668	3.058031613	-4.47165691	0.0001703	0.036664773
cg19189010	-0.838564566	1.606923083	-4.471604545	0.000170322	0.036664773
cg12720514	-0.709890834	2.192351058	-4.471574523	0.000170335	0.036664773
cg24034552	-0.608458266	2.817575993	-4.471551289	0.000170345	0.036664773
cg20930082	-0.789341648	3.063429677	-4.471360983	0.000170426	0.036664773

cg23380947	-0.520492908	3.736248659	-4.471168456	0.000170508	0.036664773
cg14258243	-0.717381988	-5.099154049	-4.471013255	0.000170575	0.036664773
cg19651642	-0.800458662	2.237987329	-4.470824302	0.000170656	0.036664773
cg18482690	-0.72700123	1.664801167	-4.470813052	0.00017066	0.036664773
cg25584787	-0.550982748	3.675158714	-4.470590721	0.000170756	0.036664773
cg08057786	-1.031304765	-0.190905458	-4.470586664	0.000170757	0.036664773
cg07567677	-0.608580478	3.592787717	-4.469976012	0.000171019	0.036664773
cg05142047	-0.587698621	0.341629948	-4.469567325	0.000171194	0.036664773
cg23723775	-0.602791504	3.67871742	-4.469560184	0.000171197	0.036664773
cg01632220	-0.694572927	3.490575819	-4.469490168	0.000171227	0.036664773
cg06666590	-0.913019447	1.389947913	-4.469473704	0.000171234	0.036664773
cg06531226	-0.454365458	-0.983527891	-4.469453832	0.000171243	0.036664773
cg01283201	-1.021020538	-3.285732576	-4.469303404	0.000171307	0.036664773
cg05519451	-0.579189052	2.981701204	-4.46929314	0.000171312	0.036664773
cg04850875	-0.38698544	0.350558413	-4.469270581	0.000171321	0.036664773
cg13189121	-0.700428713	2.67074532	-4.469066183	0.000171409	0.036664773
cg19712386	-0.920302196	2.816201403	-4.469057648	0.000171413	0.036664773
cg07808039	-0.479016762	3.902389178	-4.468884561	0.000171487	0.036664773
cg18375152	-0.48783998	3.912011392	-4.468848109	0.000171503	0.036664773
cg03555299	-0.583906983	3.102483716	-4.468679123	0.000171575	0.036664773
cg02041593	-0.444197044	0.626700883	-4.468616044	0.000171603	0.036664773
cg03101058	-0.397891287	-0.389308656	-4.468456072	0.000171671	0.036664773
cg03113919	-0.539521152	-2.34267865	-4.468274881	0.000171749	0.036664773
cg15657925	-0.91736433	-3.309037965	-4.468206055	0.000171779	0.036664773
cg10649802	-0.645361511	3.679329339	-4.468166922	0.000171796	0.036664773
cg23583267	-0.581948501	3.232356418	-4.468085383	0.000171831	0.036664773
cg14151317	-0.584873559	0.717748033	-4.467966528	0.000171882	0.036664773
cg16269457	-0.491594014	3.5040894	-4.467961137	0.000171884	0.036664773
cg21860045	-0.557876433	-0.754679833	-4.467950377	0.000171889	0.036664773
cg26673722	-0.565481571	3.314319972	-4.467920828	0.000171902	0.036664773
cg00418313	-0.785604454	2.768832142	-4.467917628	0.000171903	0.036664773
cg24504318	-0.557549677	3.354757719	-4.467894259	0.000171913	0.036664773
cg13357530	-0.592881445	3.494564345	-4.46745961	0.0001721	0.036664773
cg14047911	-0.599379836	3.409020597	-4.467439689	0.000172109	0.036664773
cg26060536	-0.56427815	3.432024362	-4.466879113	0.000172351	0.036664773
cg01729108	0.695433548	2.832969894	4.466739977	0.000172411	0.036664773
cg15714470	-0.569412447	3.518885299	-4.466634413	0.000172457	0.036664773
cg02173540	-0.760460317	3.414446476	-4.466616019	0.000172465	0.036664773
cg14778021	-0.427131421	4.001660802	-4.466585667	0.000172478	0.036664773
cg24635930	-0.802791989	0.967125769	-4.466584988	0.000172478	0.036664773
cg07439216	-0.596850062	3.386259567	-4.466446433	0.000172538	0.036664773
cg21096818	-0.876361902	3.011712186	-4.466015523	0.000172724	0.036664773
cg25481252	-0.735321546	2.870075769	-4.465909484	0.00017277	0.036664773
cg11848967	-0.538491149	0.321108507	-4.465872316	0.000172786	0.036664773
cg13089661	-0.575430464	3.221252907	-4.465799319	0.000172818	0.036664773
cg23549892	-0.659197307	3.24660591	-4.465745215	0.000172841	0.036664773
cg12918812	-0.526529095	3.445707317	-4.46544454	0.000172972	0.036664773

cg14013712	-0.462754425	4.060593358	-4.465276019	0.000173045	0.036664773
cg01982170	-0.766578489	0.612144714	-4.465274885	0.000173045	0.036664773
cg19202986	-0.466949785	-0.747592458	-4.465237069	0.000173062	0.036664773
cg02558993	-0.433214187	3.244089784	-4.465192471	0.000173081	0.036664773
cg19780920	-0.650228176	3.077085878	-4.465175231	0.000173088	0.036664773
cg05542646	-0.574501223	-0.84604759	-4.465120652	0.000173112	0.036664773
cg22540162	-0.703327451	2.958046251	-4.46512006	0.000173112	0.036664773
cg27120228	-0.455087786	3.385427667	-4.465106611	0.000173118	0.036664773
cg09423735	-0.936333261	2.127471137	-4.465075077	0.000173132	0.036664773
cg23214199	-0.740579663	2.884220945	-4.464652376	0.000173315	0.036692383
cg20060109	-0.631108174	3.095387563	-4.464495213	0.000173384	0.036692383
cg27110005	0.687757155	2.445009438	4.464416913	0.000173418	0.036692383
cg15549849	-0.587837445	0.197844208	-4.464126375	0.000173544	0.036703638
cg06426818	-0.539419505	4.02852407	-4.464056185	0.000173574	0.036703638
cg13154009	-0.485696678	-1.185759977	-4.463511882	0.000173811	0.036742764
cg19071976	-0.473610647	-0.338569139	-4.463335324	0.000173888	0.036748056
cg09000827	-0.412270588	-1.401360109	-4.462854116	0.000174098	0.03676794
cg10991910	-0.451004141	-0.575369441	-4.46283433	0.000174107	0.03676794
cg11949518	-0.814986407	3.587201661	-4.462513744	0.000174247	0.03676794
cg08198574	-0.655552579	0.550241164	-4.462444956	0.000174277	0.03676794
cg23473374	-0.590898911	2.771852323	-4.462379034	0.000174305	0.03676794
cg07211884	0.666367725	0.921738935	4.462181201	0.000174392	0.03676794
cg05317405	0.441241717	0.799763197	4.462079294	0.000174436	0.03676794
cg04451259	-0.580026633	-1.436719838	-4.462062321	0.000174444	0.03676794
cg16652347	-0.532157968	2.559207155	-4.462049352	0.000174449	0.03676794
cg19399653	-0.484617307	2.940076206	-4.461669887	0.000174615	0.036786236
cg15066885	-0.643972875	2.448808542	-4.461613412	0.00017464	0.036786236
cg11734411	-0.448583431	3.970796593	-4.461284639	0.000174784	0.036793948
cg15032493	-0.77846503	2.19172288	-4.460970566	0.000174922	0.036793948
cg27541570	-0.905894633	-0.870684566	-4.46089763	0.000174954	0.036793948
cg18324215	-0.697820827	1.895662874	-4.460866122	0.000174967	0.036793948
cg15823896	-0.894240413	1.85168611	-4.460696783	0.000175042	0.036793948
cg26373662	-0.474860489	2.898954586	-4.460624962	0.000175073	0.036793948
cg18769929	-0.56193247	3.22273831	-4.460610932	0.000175079	0.036793948
cg02951876	-0.481197256	3.748908245	-4.46058153	0.000175092	0.036793948
cg12597444	-0.631089813	3.209017201	-4.460309762	0.000175212	0.036808094
cg02610157	-0.658099063	3.330486926	-4.460085747	0.00017531	0.036811427
cg17437621	-0.629175508	3.698226564	-4.460029681	0.000175335	0.036811427
cg23372016	-0.830477452	2.045228344	-4.459894757	0.000175394	0.036811427
cg27608402	-0.683650927	1.267942919	-4.459600527	0.000175523	0.036811427
cg03908391	-0.531192612	0.156107254	-4.459327054	0.000175644	0.036811427
cg10197297	-0.512485457	4.155813051	-4.459242318	0.000175681	0.036811427
cg03182620	-0.620321356	0.434178215	-4.458995401	0.00017579	0.036811427
cg24077869	-0.628237754	3.18452437	-4.458911938	0.000175826	0.036811427
cg19593184	-0.502374473	2.96318202	-4.458806707	0.000175873	0.036811427
cg12742491	-0.678475767	3.167909685	-4.458802487	0.000175875	0.036811427
cg14279245	-0.545457821	-0.04151674	-4.458515013	0.000176001	0.036811427

cg05503484	-0.998998568	2.231902753	-4.458397173	0.000176053	0.036811427
cg00468091	-0.529795465	3.484576098	-4.458256457	0.000176115	0.036811427
cg09135441	0.459321443	1.569499827	4.458165993	0.000176155	0.036811427
cg23741939	-0.450058635	-0.708318614	-4.458121842	0.000176175	0.036811427
cg02270765	-0.58722344	2.353962876	-4.457903593	0.000176271	0.036811427
cg04512340	-0.90159043	1.64065348	-4.457875914	0.000176283	0.036811427
cg06009329	-0.442167653	3.525003913	-4.457830569	0.000176303	0.036811427
cg11605890	-0.420835242	3.717593231	-4.457663639	0.000176377	0.036811427
cg02400139	-0.482192536	4.164339347	-4.457586793	0.000176411	0.036811427
cg20590195	-0.466432687	3.012278695	-4.457505222	0.000176447	0.036811427
cg03346523	-0.550014219	2.891503016	-4.457483896	0.000176457	0.036811427
cg06331557	-0.553928518	3.318855143	-4.457436759	0.000176477	0.036811427
cg20122981	-0.611948822	3.207098891	-4.457382352	0.000176502	0.036811427
cg13573530	-0.528887072	-0.840384173	-4.457325734	0.000176527	0.036811427
cg27645492	-0.511714452	0.172550655	-4.457159009	0.0001766	0.036815972
cg21631439	-0.513577222	-0.600329746	-4.456630597	0.000176834	0.036853905
cg01602666	-0.740169978	1.204696632	-4.456456133	0.000176912	0.03685718
cg27358375	-0.523407204	-1.11912848	-4.456360382	0.000176954	0.03685718
cg02389050	-0.695582396	3.47749935	-4.456062647	0.000177086	0.036873845
cg25289656	-0.711453581	3.315524712	-4.455889691	0.000177163	0.036878989
cg24455985	-0.584362598	3.279625576	-4.455530846	0.000177322	0.036894403
cg07367791	0.503010913	-1.233212544	4.45548844	0.000177341	0.036894403
cg11144775	-0.526664654	3.762674738	-4.455208457	0.000177466	0.036895418
cg07132072	-0.957306155	2.630990301	-4.455136347	0.000177498	0.036895418
cg13790618	-0.433700694	-1.251285553	-4.454771646	0.00017766	0.036895418
cg02533281	-0.579872679	3.126716133	-4.454766952	0.000177662	0.036895418
cg11341604	-0.538605824	4.044175504	-4.45465869	0.00017771	0.036895418
cg15472485	-0.726784279	2.27397035	-4.454651391	0.000177713	0.036895418
cg25416878	-0.794516062	2.290429113	-4.454457222	0.0001778	0.036895418
cg12814766	-0.546889027	3.085827763	-4.454298165	0.000177871	0.036895418
cg01995265	-0.839042746	3.038452543	-4.454083363	0.000177967	0.036895418
cg25485388	-0.523517167	3.881616231	-4.454082366	0.000177967	0.036895418
cg16301894	-0.580558144	-1.688270808	-4.453965013	0.000178019	0.036895418
cg08022070	-0.669374702	2.648648852	-4.453571398	0.000178195	0.036895418
cg00874817	-1.237917787	-4.41505464	-4.453274921	0.000178327	0.036895418
cg12753845	-0.487890556	4.173434498	-4.453225457	0.00017835	0.036895418
cg15804669	-0.619650693	3.145204508	-4.453200007	0.000178361	0.036895418
cg16767323	-0.608472176	3.323662103	-4.453127134	0.000178394	0.036895418
cg12360666	-0.68162608	3.337792285	-4.453074508	0.000178417	0.036895418
cg16441347	-0.575396635	2.72585035	-4.452791526	0.000178544	0.036895418
cg02714148	-0.443496118	4.063733264	-4.452743424	0.000178565	0.036895418
cg21701184	-0.658988361	3.181366237	-4.452477091	0.000178684	0.036895418
cg12923242	-1.188469115	1.429886043	-4.452442683	0.0001787	0.036895418
cg13576605	-0.488033789	3.441858572	-4.452431213	0.000178705	0.036895418
cg13397057	0.672957618	-3.726803421	4.452424543	0.000178708	0.036895418
cg06350590	-0.474602066	4.019015188	-4.452338366	0.000178746	0.036895418
cg03337482	0.739730476	3.470531295	4.452267947	0.000178778	0.036895418

cg19611602	-0.654174982	3.421672295	-4.452105924	0.000178851	0.036895418
cg25055520	-0.380426597	3.47095291	-4.451903626	0.000178941	0.036895418
cg08699899	-0.539359542	4.001122541	-4.451712815	0.000179027	0.036895418
cg07029002	-0.507566138	-0.883598592	-4.451706612	0.00017903	0.036895418
cg06871246	-0.789028229	1.88540977	-4.451671342	0.000179045	0.036895418
cg15324917	-0.526477485	-0.710754788	-4.451540854	0.000179104	0.036895418
cg06904008	-0.568196257	3.269067978	-4.451527658	0.00017911	0.036895418
cg11440716	-0.423366757	0.051110516	-4.451473998	0.000179134	0.036895418
cg11597754	-0.47742175	-0.585359704	-4.451407381	0.000179164	0.036895418
cg06727131	-0.532519556	-0.235752325	-4.451311693	0.000179207	0.036895418
cg26450149	-0.466376588	-0.052318679	-4.451280232	0.000179221	0.036895418
cg22478038	-0.833317737	-0.858635631	-4.450682821	0.00017949	0.036931717
cg08379987	-0.671523889	-2.948400975	-4.450655902	0.000179502	0.036931717
cg02606469	-0.642977596	2.742205394	-4.45043889	0.000179599	0.036934513
cg07553297	0.767772896	-2.951635288	4.450218785	0.000179698	0.036934513
cg04310193	-0.53140986	-0.528338061	-4.450068796	0.000179766	0.036934513
cg05764393	0.586584589	1.641867579	4.45005055	0.000179774	0.036934513
cg11132198	-0.668585573	2.799544253	-4.449947251	0.000179821	0.036934513
cg22432627	-0.485074455	-1.565343859	-4.449930822	0.000179828	0.036934513
cg03479706	-0.452669065	3.498525726	-4.449589252	0.000179982	0.036950491
cg07599830	-0.567465215	3.971373375	-4.449412566	0.000180062	0.036950491
cg00242039	-0.449455914	1.177047221	-4.449411234	0.000180062	0.036950491
cg01737284	-0.840576516	2.278664586	-4.449213714	0.000180151	0.036958078
cg19623397	0.692891579	2.526951948	4.449092765	0.000180206	0.036958577
cg02555663	-0.836890048	-3.033300016	-4.448795461	0.00018034	0.036966988
cg24300446	-0.448313973	-0.064165442	-4.448770933	0.000180351	0.036966988
cg05956185	-0.676886513	-0.634607053	-4.448387653	0.000180525	0.036981049
cg15393390	-0.838559298	1.014655778	-4.448239486	0.000180592	0.036981049
cg21758525	-0.627561601	1.998684375	-4.448183341	0.000180617	0.036981049
cg08163568	-0.640865069	2.63239399	-4.448074403	0.000180667	0.036981049
cg27372920	-0.470149473	-1.641365722	-4.448042198	0.000180681	0.036981049
cg07977260	-0.581137071	-0.29511292	-4.447600253	0.000180881	0.037011331
cg14338893	0.710418146	-4.09480387	4.447153744	0.000181084	0.037037548
cg04353365	-0.579376508	-0.724346809	-4.447087176	0.000181114	0.037037548
cg10151953	-0.466081546	0.296561096	-4.446784684	0.000181251	0.037050004
cg03863606	-0.604531718	3.006601166	-4.446495839	0.000181383	0.037050004
cg24157509	-0.468033091	3.365851625	-4.44645875	0.000181399	0.037050004
cg05409848	-0.500998626	0.018020476	-4.446391715	0.00018143	0.037050004
cg26545167	-0.866304858	2.170506358	-4.446319013	0.000181463	0.037050004
cg26501729	-0.610923962	0.549880995	-4.446262323	0.000181489	0.037050004
cg12074633	-0.518561231	4.173016554	-4.445600286	0.00018179	0.037052241
cg27296835	-0.504763144	3.375349142	-4.445521989	0.000181826	0.037052241
cg21895182	-0.530792181	3.590466181	-4.445212597	0.000181967	0.037052241
cg07172755	-0.574294645	0.734205757	-4.445148182	0.000181996	0.037052241
cg11601120	-0.681170664	2.819758515	-4.445121332	0.000182008	0.037052241
cg07699740	-0.635710891	2.366338378	-4.444918467	0.000182101	0.037052241
cg20770201	-0.575828941	1.907076146	-4.444868059	0.000182124	0.037052241

cg08044345	-0.774291113	2.094367191	-4.444678347	0.00018221	0.037052241
cg14542120	-0.53627533	-1.772467042	-4.444495677	0.000182294	0.037052241
cg18568859	-0.481762829	3.728171966	-4.444221898	0.000182419	0.037052241
cg01729999	0.566342583	3.102323725	4.44415314	0.00018245	0.037052241
cg16969274	-0.511279385	3.201198884	-4.444131345	0.00018246	0.037052241
cg11187807	-0.417585448	-0.491912248	-4.444129834	0.000182461	0.037052241
cg11774116	-0.501146377	-0.359451008	-4.444106363	0.000182472	0.037052241
cg09192881	-0.477907692	-0.229031595	-4.443956478	0.00018254	0.037052241
cg19431140	-0.560438521	3.358672068	-4.443887148	0.000182572	0.037052241
cg27332131	-0.632333078	2.567295252	-4.443806381	0.000182609	0.037052241
cg11878146	-0.456482099	2.93880704	-4.443726516	0.000182646	0.037052241
cg07486252	-0.46037173	-1.556762633	-4.4436237	0.000182693	0.037052241
cg01607771	-0.43350213	-0.11016586	-4.443602074	0.000182703	0.037052241
cg16425355	-0.685549127	2.988122493	-4.443446824	0.000182774	0.037052241
cg06790450	-0.569086083	2.960655119	-4.443389214	0.0001828	0.037052241
cg17043823	-0.527872306	3.9763681	-4.443336464	0.000182824	0.037052241
cg00145863	-0.604823157	1.520262239	-4.44332341	0.00018283	0.037052241
cg14095525	-0.572244637	0.349580981	-4.442972622	0.000182991	0.037052241
cg11734254	-0.569747261	3.976276946	-4.44286742	0.000183039	0.037052241
cg09374028	-0.633933195	-0.159941836	-4.442852789	0.000183046	0.037052241
cg06424232	-0.529449821	3.894570252	-4.442819984	0.000183061	0.037052241
cg08925998	-0.500169152	3.786462631	-4.442630201	0.000183148	0.037052241
cg17701285	-0.502429139	4.12683286	-4.442498867	0.000183208	0.037052241
cg12129577	-0.673368553	3.159601065	-4.442485153	0.000183215	0.037052241
cg06254176	-0.415308181	3.573064585	-4.442412028	0.000183248	0.037052241
cg10774231	-0.706589714	2.565373341	-4.442308765	0.000183296	0.037052241
cg04314092	-0.612464004	0.477503702	-4.442188431	0.000183351	0.037052241
cg01085165	-0.521078053	0.07957249	-4.4421435	0.000183371	0.037052241
cg10013798	-0.994419441	2.318237364	-4.442119194	0.000183383	0.037052241
cg00336168	-0.528457479	3.667689528	-4.441910803	0.000183478	0.037059848
cg22509053	-0.663249338	2.683286052	-4.44170183	0.000183575	0.037059848
cg07317983	-0.644611763	-0.654614355	-4.44165675	0.000183595	0.037059848
cg27396403	-0.503211339	2.389173916	-4.441572192	0.000183634	0.037059848
cg03694644	-0.69349885	3.735148887	-4.441468533	0.000183682	0.037059848
cg18978196	-0.528382115	3.233546273	-4.441171306	0.000183819	0.037076046
cg01078695	-0.69814154	-0.354798314	-4.441057457	0.000183871	0.037076046
cg02021596	-0.495810155	3.921067722	-4.440953279	0.000183919	0.037076046
cg23882573	-0.777056729	3.276000146	-4.440401496	0.000184174	0.037116781
cg23043091	0.349373805	1.172066042	4.439780535	0.00018446	0.037164005
cg18311413	-0.477637306	3.591418951	-4.439579465	0.000184553	0.037164748
cg03700622	-0.478199504	2.9931042	-4.43954561	0.000184569	0.037164748
cg16119643	-0.527142956	3.33319621	-4.439132005	0.00018476	0.037178972
cg27437083	-0.616062435	3.553487705	-4.439061722	0.000184793	0.037178972
cg24421216	-0.796780235	-0.950903985	-4.439014905	0.000184815	0.037178972
cg02661431	-0.512836771	-0.390532711	-4.438939392	0.00018485	0.037178972
cg06047881	-0.457045784	-0.810084916	-4.43861192	0.000185001	0.037181517
cg17823067	-0.612345907	2.878253104	-4.43858632	0.000185013	0.037181517

cg03711942	-0.480225958	3.642595761	-4.438525064	0.000185042	0.037181517
cg27389242	-0.649246207	-1.052982839	-4.438256106	0.000185166	0.037181517
cg25844265	-0.714586339	1.999594293	-4.438201343	0.000185192	0.037181517
cg01663008	-0.716835664	-0.105765562	-4.43818396	0.0001852	0.037181517
cg23794135	-0.565074698	3.4283644	-4.438119676	0.00018523	0.037181517
cg12857064	-0.505910613	2.962583359	-4.437896877	0.000185333	0.037185046
cg02885516	-0.647800486	3.359459109	-4.437855684	0.000185352	0.037185046
cg05577736	-0.505827103	-0.307582214	-4.43773127	0.00018541	0.037186109
cg23295985	-0.651699767	1.669486939	-4.437371204	0.000185577	0.037197279
cg13709186	-0.474101835	3.957133891	-4.437230625	0.000185643	0.037197279
cg12399389	-0.590400056	3.847927945	-4.437198153	0.000185658	0.037197279
cg00855685	-0.692353104	-0.107601105	-4.436989462	0.000185755	0.037197279
cg10022194	-0.461765686	3.272560306	-4.436945284	0.000185776	0.037197279
cg21604742	-0.411712058	4.37970712	-4.436880047	0.000185806	0.037197279
cg22408586	-0.462833823	0.71337091	-4.436729509	0.000185876	0.037197279
cg12626957	-0.55809009	-1.101800102	-4.436708492	0.000185886	0.037197279
cg00123317	-0.471708206	4.348684223	-4.436332655	0.000186061	0.03721124
cg17995347	-0.612392938	3.136559637	-4.436316114	0.000186069	0.03721124
cg05079689	-0.725383455	2.156983886	-4.436108622	0.000186165	0.03721124
cg12639923	-0.468951199	4.400233643	-4.436101057	0.000186169	0.03721124
cg22292151	-0.759553693	2.174130583	-4.435995444	0.000186218	0.03721124
cg04038724	-0.563580945	3.370779423	-4.43555473	0.000186424	0.037232927
cg05766901	0.613620051	1.630821248	4.435346901	0.000186521	0.037232927
cg16509592	-0.5537279	2.836243009	-4.435142761	0.000186617	0.037232927
cg04055795	-0.710584141	-0.517508167	-4.435041108	0.000186664	0.037232927
cg23881653	-0.528957559	3.618497525	-4.435016885	0.000186675	0.037232927
cg23228587	-0.455642404	4.021888473	-4.43495728	0.000186703	0.037232927
cg17215172	-0.405532676	-0.095541063	-4.43480599	0.000186774	0.037232927
cg11565512	-0.627673744	-0.830521561	-4.434773484	0.000186789	0.037232927
cg27464144	-0.536310764	-0.591320562	-4.43475101	0.0001868	0.037232927
cg19878348	-0.498377621	3.564207057	-4.434369938	0.000186978	0.037258013
cg12235111	-0.662405054	1.699120462	-4.433990356	0.000187156	0.037259847
cg18737081	-0.441273731	0.182884522	-4.433965148	0.000187168	0.037259847
cg07195211	-0.601548715	1.290064874	-4.433938597	0.00018718	0.037259847
cg16364155	-0.644011164	3.10490835	-4.433901495	0.000187198	0.037259847
cg08912554	-0.855571853	2.398040014	-4.433568678	0.000187354	0.037280458
cg05769891	-0.456198865	4.152495688	-4.433313066	0.000187474	0.037289558
cg01465878	-0.44164323	3.798866652	-4.432937474	0.000187651	0.037289558
cg08749605	-0.679446448	3.499035904	-4.43237616	0.000187915	0.037289558
cg20033307	-0.498015991	-0.856554259	-4.432349821	0.000187927	0.037289558
cg19531391	-0.656776674	2.876955985	-4.432310065	0.000187946	0.037289558
cg09832180	-0.492034733	3.703252884	-4.432264027	0.000187968	0.037289558
cg07451520	-0.610421255	3.723725059	-4.43211654	0.000188037	0.037289558
cg16577567	-0.548201379	3.419187612	-4.432054018	0.000188066	0.037289558
cg09432964	-0.473972356	3.750247691	-4.431980068	0.000188101	0.037289558
cg01412300	-0.724688918	1.329821813	-4.431690687	0.000188238	0.037289558
cg18913923	-0.496909167	0.029828971	-4.43167226	0.000188246	0.037289558

cg19296262	-0.544905326	-0.106403307	-4.43161274	0.000188275	0.037289558
cg13420273	-0.634580758	3.748546216	-4.431472618	0.000188341	0.037289558
cg14008998	-0.783320347	2.087966817	-4.431398141	0.000188376	0.037289558
cg08692903	-0.368439493	-0.567564476	-4.431103744	0.000188515	0.037289558
cg22684787	-0.425461452	-1.912523175	-4.431016444	0.000188556	0.037289558
cg18712566	-0.5174257	0.766154471	-4.43076333	0.000188676	0.037289558
cg17392043	-0.617224817	2.343752609	-4.430760842	0.000188677	0.037289558
cg26879637	-0.829780817	2.294497654	-4.430711421	0.0001887	0.037289558
cg09310575	-0.585376259	2.460829089	-4.430702065	0.000188705	0.037289558
cg04752945	-0.510550202	0.237529321	-4.430549475	0.000188777	0.037289558
cg19654323	-2.071437952	-2.915098102	-4.430426895	0.000188835	0.037289558
cg05634679	-0.537890348	-0.371122983	-4.430418717	0.000188839	0.037289558
cg05687603	-0.492260024	3.842371384	-4.430166136	0.000188958	0.037289558
cg01868752	-0.681196699	2.635721798	-4.430154121	0.000188964	0.037289558
cg12913175	0.878428538	3.190718961	4.430134859	0.000188973	0.037289558
cg11520308	-0.600141433	0.108698985	-4.430105906	0.000188987	0.037289558
cg12512180	-0.467631326	4.024319929	-4.430098354	0.00018899	0.037289558
cg06840100	-0.590973069	3.143055704	-4.430093258	0.000188993	0.037289558
cg02493939	-0.442437823	0.098764685	-4.429897642	0.000189085	0.037289558
cg07396062	-0.809641363	2.055864252	-4.429770557	0.000189146	0.037289558
cg10674569	-0.790825365	0.807594584	-4.429697555	0.00018918	0.037289558
cg09714950	-0.434865034	3.795779757	-4.429599883	0.000189227	0.037289558
cg13845363	-0.457814531	3.922833629	-4.429592318	0.00018923	0.037289558
cg20452230	0.816498058	-3.270205618	4.429566794	0.000189242	0.037289558
cg24148415	-0.555178622	3.29977847	-4.429409856	0.000189317	0.037293851
cg12069042	-0.473276512	-0.738184653	-4.429065848	0.00018948	0.037312378
cg26713664	-0.503544591	3.142889865	-4.428665465	0.00018967	0.037312378
cg15064387	-0.497873171	3.373510064	-4.428541393	0.000189729	0.037312378
cg27041299	-0.617880153	3.309123697	-4.42851325	0.000189742	0.037312378
cg03098848	-0.585612993	2.006172792	-4.428468081	0.000189764	0.037312378
cg18177826	-0.567683284	3.9075995	-4.428395447	0.000189798	0.037312378
cg24376286	-0.378235808	-0.068744059	-4.427663632	0.000190147	0.037312378
cg04383208	-0.402465534	3.925864192	-4.427648115	0.000190154	0.037312378
cg00120897	0.586698712	3.009917334	4.42757509	0.000190189	0.037312378
cg10519882	-0.523041294	0.395551443	-4.42752536	0.000190213	0.037312378
cg05607531	-0.468837901	-0.023098615	-4.427206528	0.000190365	0.037312378
cg26350919	-0.683922241	2.776740023	-4.427189026	0.000190373	0.037312378
cg11526347	-0.524184444	2.245682453	-4.427022022	0.000190453	0.037312378
cg19640754	-0.44921764	3.634785864	-4.42694828	0.000190488	0.037312378
cg09262882	0.414206777	2.099289622	4.426868403	0.000190526	0.037312378
cg05002165	-0.619922383	1.478325749	-4.426678141	0.000190617	0.037312378
cg00546844	0.618120138	2.88968943	4.42667235	0.00019062	0.037312378
cg12966553	-0.601140434	2.901879714	-4.426416845	0.000190742	0.037312378
cg09043768	-0.49030396	-1.58636797	-4.42620859	0.000190841	0.037312378
cg21338373	-0.501908088	3.403518968	-4.425725735	0.000191072	0.037312378
cg04021557	-0.564041951	3.332810716	-4.42560308	0.000191131	0.037312378
cg05902968	-0.484380807	-0.08946526	-4.425565777	0.000191149	0.037312378

cg09059395	-0.524041349	0.526957148	-4.425480472	0.00019119	0.037312378
cg14527333	-0.986992566	-1.011396269	-4.425425546	0.000191216	0.037312378
cg08893641	-0.665361484	-4.539764314	-4.425394696	0.000191231	0.037312378
cg07549115	-0.63982734	3.359932647	-4.425337	0.000191259	0.037312378
cg25611344	-0.49293054	3.479726557	-4.425292051	0.00019128	0.037312378
cg18871813	-0.535016589	3.469698456	-4.425170533	0.000191338	0.037312378
cg08254353	-0.547291368	-1.701950244	-4.425120467	0.000191362	0.037312378
cg20484417	-0.474317343	0.62828198	-4.425080703	0.000191381	0.037312378
cg21618948	-0.419414859	-2.193198754	-4.42491774	0.00019146	0.037312378
cg18877558	-0.678804657	2.616917057	-4.424095657	0.000191854	0.037312378
cg22072966	-0.933636279	2.277671424	-4.423959541	0.00019192	0.037312378
cg02874061	-0.433396238	-0.479544807	-4.423875505	0.00019196	0.037312378
cg27161809	-0.591144347	2.673625317	-4.42344415	0.000192168	0.037312378
cg15466595	-0.776241552	3.287109968	-4.423360535	0.000192208	0.037312378
cg22233808	-0.501359358	3.81523262	-4.423338068	0.000192219	0.037312378
cg00137607	-0.587172355	2.770563582	-4.42325631	0.000192258	0.037312378
cg14080063	-0.422150515	3.373636537	-4.42324784	0.000192262	0.037312378
cg19259421	-0.560584847	3.06876609	-4.423237613	0.000192267	0.037312378
cg27183454	-0.525523998	3.697330557	-4.423203481	0.000192284	0.037312378
cg13179445	-0.39484495	3.438732599	-4.423153438	0.000192308	0.037312378
cg09725202	-0.494557155	4.036025703	-4.423030688	0.000192367	0.037312378
cg18737468	-0.731619633	2.165305082	-4.42297119	0.000192396	0.037312378
cg22107525	-0.760593126	-0.012743707	-4.422927434	0.000192417	0.037312378
cg23096663	-0.48910083	4.368896726	-4.422825866	0.000192466	0.037312378
cg07749136	-0.466937127	-1.444901725	-4.422713755	0.00019252	0.037312378
cg14918782	0.493292469	2.644811542	4.422680071	0.000192536	0.037312378
cg16489926	-0.729974552	-0.383058152	-4.42267938	0.000192536	0.037312378
cg05439503	-0.455504395	3.939939892	-4.422638028	0.000192556	0.037312378
cg09149974	-0.60919112	3.329015027	-4.422615056	0.000192567	0.037312378
cg11144986	-4.020777171	2.145361416	-4.422554659	0.000192597	0.037312378
cg00404703	-0.461802355	-0.137367748	-4.422458192	0.000192643	0.037312378
cg06882900	-0.594170571	4.356970435	-4.422403223	0.00019267	0.037312378
cg13510487	-0.545955861	3.018099736	-4.422333713	0.000192703	0.037312378
cg16491571	-0.443795503	4.031275689	-4.42232962	0.000192705	0.037312378
cg00255726	0.412222287	4.047484731	4.422305887	0.000192717	0.037312378
cg14021880	-0.413927526	-0.418981739	-4.422169938	0.000192782	0.037312378
cg15370654	-0.691753871	2.371248363	-4.422123833	0.000192805	0.037312378
cg14881923	-0.738113628	-0.45468501	-4.422088383	0.000192822	0.037312378
cg07133969	-0.622635686	3.038155879	-4.421922401	0.000192902	0.037312378
cg19685491	-0.506428844	-1.301104526	-4.421576613	0.000193069	0.037312378
cg13373080	-0.473957734	-0.071876479	-4.421575818	0.00019307	0.037312378
cg01850278	-0.593033819	2.734057788	-4.421528425	0.000193092	0.037312378
cg02437346	-0.623732538	3.411076953	-4.421418722	0.000193146	0.037312378
cg11813658	-0.808283574	1.791019211	-4.421332908	0.000193187	0.037312378
cg00682038	-0.450981216	-1.263359893	-4.421254073	0.000193225	0.037312378
cg14585907	-0.640493152	3.02273028	-4.421213853	0.000193245	0.037312378
cg08243301	-0.504874059	-0.499170835	-4.42094111	0.000193377	0.037312378

cg18960406	-0.422465528	3.908984994	-4.420841395	0.000193425	0.037312378
cg16054606	-0.695736639	1.329063796	-4.420653131	0.000193516	0.037312378
cg24037916	0.80198	-2.909607749	4.420620067	0.000193532	0.037312378
cg09931111	-0.620822258	3.215711425	-4.42049047	0.000193595	0.037312378
cg09589796	-0.613907924	3.411138934	-4.420461417	0.000193609	0.037312378
cg18647095	-0.439211888	3.756564694	-4.420451163	0.000193614	0.037312378
cg03016500	-0.420372082	4.255270254	-4.420346042	0.000193665	0.037312378
cg09682551	-0.653327906	-1.457845916	-4.420208871	0.000193732	0.037312378
cg02105950	-0.594887564	3.014221928	-4.420083911	0.000193793	0.037312378
cg04202900	-0.420657389	4.179443524	-4.420069999	0.000193799	0.037312378
cg17916980	-0.517849263	0.075352982	-4.42004048	0.000193814	0.037312378
cg00211337	-0.689870417	2.664288502	-4.419903561	0.00019388	0.037312378
cg00448578	-0.586445381	1.558450637	-4.419603125	0.000194026	0.037312378
cg05026786	-0.648728545	0.869212914	-4.41957302	0.000194041	0.037312378
cg15499264	-0.535230769	3.392888582	-4.419473368	0.000194089	0.037312378
cg05423738	-0.62735202	3.043169637	-4.419433564	0.000194109	0.037312378
cg21295207	-0.599426747	3.356583581	-4.419389467	0.00019413	0.037312378
cg17161697	-0.727419886	1.323227316	-4.419272348	0.000194187	0.037312378
cg17785937	-0.592832882	3.307150987	-4.419162538	0.000194241	0.037312378
cg09887867	-0.447434927	-2.436479571	-4.41883564	0.0001944	0.037312378
cg14336112	-0.598178461	4.296152298	-4.41882327	0.000194406	0.037312378
cg07074891	-0.389437342	-0.402767793	-4.418803885	0.000194415	0.037312378
cg02230774	-0.840835378	2.639529614	-4.418761022	0.000194436	0.037312378
cg27377493	-0.684692141	2.681963436	-4.41853288	0.000194547	0.037312378
cg08538509	-0.422287501	3.932226859	-4.418416662	0.000194604	0.037312378
cg08999124	-0.474991672	-0.112591928	-4.418406088	0.000194609	0.037312378
cg03969079	-0.394238001	4.240949223	-4.418224393	0.000194698	0.037312378
cg02670516	-0.473132443	0.154767648	-4.418189491	0.000194715	0.037312378
cg13977545	-0.687518588	2.835743558	-4.418181175	0.000194719	0.037312378
cg12951341	-0.694523963	3.205832666	-4.418075181	0.00019477	0.037312378
cg22169750	-0.994433034	0.825148359	-4.418036713	0.000194789	0.037312378
cg20003050	-0.594794868	2.917200528	-4.418013729	0.0001948	0.037312378
cg25322596	-0.516010088	2.830711317	-4.417581721	0.000195011	0.037312378
cg25664878	-0.709956344	2.124915259	-4.417551978	0.000195026	0.037312378
cg20693126	-0.434347293	-0.77440586	-4.417444656	0.000195078	0.037312378
cg09386315	-0.556924793	-0.747561713	-4.41737946	0.00019511	0.037312378
cg20689661	-0.514433176	-0.169738306	-4.41718561	0.000195205	0.037312378
cg16195245	-0.445336765	-0.559752399	-4.417020074	0.000195286	0.037312378
cg02854877	-0.427129338	4.300209952	-4.417013555	0.000195289	0.037312378
cg20415435	-0.752660517	2.052540001	-4.417009579	0.000195291	0.037312378
cg22305564	-0.55289603	3.279009033	-4.416909279	0.00019534	0.037312378
cg23501196	-0.723639492	2.690555287	-4.416790395	0.000195399	0.037312378
cg14207507	-0.470425928	3.245049818	-4.416720096	0.000195433	0.037312378
cg10612815	-0.519602599	3.644039011	-4.416681316	0.000195452	0.037312378
cg03553125	-0.428002987	3.685948469	-4.416507034	0.000195537	0.037312378
cg04653887	-0.403531147	0.178341482	-4.416351975	0.000195613	0.037312378
cg06775073	-0.429089857	-0.786623654	-4.416342308	0.000195618	0.037312378

cg00002926	-0.643648872	3.147291345	-4.416335894	0.000195621	0.037312378
cg15721577	-0.691810368	-0.891313843	-4.416304139	0.000195637	0.037312378
cg23618114	-0.603443427	3.319341149	-4.416195766	0.00019569	0.037312378
cg09299668	-0.428963089	2.787317502	-4.416111068	0.000195731	0.037312378
cg22539390	-0.530490785	3.819965237	-4.415999205	0.000195786	0.03731238
cg25445808	-0.602351416	3.311136491	-4.415880648	0.000195844	0.03731238
cg19605359	-0.588792271	2.634172576	-4.415614313	0.000195975	0.03731238
cg01288933	-0.795925285	2.409442004	-4.41557075	0.000195997	0.03731238
cg24596259	-0.666714905	2.909283184	-4.415433345	0.000196064	0.03731238
cg15050139	-0.41330101	1.162746478	-4.415406226	0.000196077	0.03731238
cg17347650	-0.600333268	3.03433352	-4.415340767	0.00019611	0.03731238
cg13883966	-0.591405534	2.604100673	-4.415252761	0.000196153	0.03731238
cg10231862	-0.613766463	3.270282891	-4.415019492	0.000196267	0.037322053
cg18103325	-0.513766803	3.519837642	-4.414921978	0.000196315	0.037322053
cg22795059	-0.622740538	2.096988094	-4.414807948	0.000196372	0.037322053
cg13171643	-0.550878344	2.701936441	-4.414415247	0.000196565	0.037322053
cg26659082	-0.872950847	2.058921749	-4.414298929	0.000196622	0.037322053
cg19784428	-0.48137908	-0.38383596	-4.41424415	0.000196649	0.037322053
cg15966720	-0.58743068	2.944495204	-4.414214162	0.000196664	0.037322053
cg09971289	-0.427434047	3.686754029	-4.414120137	0.00019671	0.037322053
cg14378149	-0.922509426	2.277032034	-4.414105572	0.000196717	0.037322053
cg11383275	-0.60134182	3.69332345	-4.414009511	0.000196765	0.037322053
cg03522510	-0.519857598	3.638495043	-4.413967152	0.000196786	0.037322053
cg01211627	-0.644679675	3.238663284	-4.413865342	0.000196836	0.037322053
cg25302172	-0.467886466	4.116390029	-4.413130002	0.000197199	0.037352873
cg06693166	-0.675261791	2.948829743	-4.413109991	0.000197209	0.037352873
cg24482226	-0.376591169	-1.693582917	-4.413044711	0.000197241	0.037352873
cg06120764	-0.446924449	0.824095704	-4.413015813	0.000197255	0.037352873
cg11035319	-0.754998836	2.865100815	-4.412996928	0.000197265	0.037352873
cg22086454	-0.719878132	2.850691906	-4.41280374	0.00019736	0.037352873
cg25631694	-0.590721666	2.324758431	-4.412757442	0.000197383	0.037352873
cg03966746	-0.657835335	3.106774108	-4.412607937	0.000197457	0.037352873
cg09674400	-0.660889571	3.229644262	-4.412561387	0.00019748	0.037352873
cg06279648	-0.749342234	2.79962748	-4.41240799	0.000197556	0.037352873
cg12985297	-0.749636736	2.33888703	-4.412218705	0.00019765	0.037352873
cg10688799	-0.467807009	-1.711894286	-4.412032616	0.000197742	0.037352873
cg03047383	-0.674222509	-0.024228516	-4.41196571	0.000197775	0.037352873
cg12892666	-1.014918059	0.47052232	-4.411945509	0.000197785	0.037352873
cg25923786	-0.480287662	3.287688291	-4.411917199	0.000197799	0.037352873
cg09840472	-0.493215126	3.852407856	-4.411830334	0.000197842	0.037352873
cg24347263	-0.557138058	-2.454371587	-4.411694025	0.00019791	0.037355677
cg10032289	-0.462955961	3.716424482	-4.411432545	0.000198039	0.037360997
cg04925574	-0.496663243	0.135254883	-4.411424563	0.000198043	0.037360997
cg11657741	-0.549411632	3.03284627	-4.41130952	0.0001981	0.037361818
cg07745808	-0.723974271	3.206797379	-4.41092303	0.000198292	0.037388062
cg10051161	-0.531981261	3.76679763	-4.410590692	0.000198458	0.037406771
cg24895862	0.52655228	1.286010834	4.410510962	0.000198497	0.037406771

cg21726662	-0.703131065	0.305412763	-4.410378141	0.000198563	0.037408799
cg12656805	-0.566258435	1.036869241	-4.410277036	0.000198614	0.037408799
cg08210468	1.151755664	3.138876833	4.410084502	0.000198709	0.037409616
cg18771050	-0.621148949	0.446021738	-4.410056141	0.000198724	0.037409616
cg19133921	-0.355675533	-0.322915653	-4.40947199	0.000199015	0.037446838
cg26602666	-0.868042997	3.06162402	-4.40944717	0.000199027	0.037446838
cg09861583	-0.4950392	-0.391762855	-4.409279477	0.000199111	0.037452342
cg23560388	0.617402775	3.318054763	4.409176562	0.000199162	0.037452342
cg13799628	0.795479003	1.83846592	4.408950406	0.000199275	0.037463626
cg06146802	-0.562673915	0.191471072	-4.40876779	0.000199366	0.037470827
cg05569767	-0.474166852	2.971322559	-4.40856679	0.000199467	0.037472181
cg15121978	-0.681082609	3.159739832	-4.408541646	0.000199479	0.037472181
cg04501607	-0.583524413	3.704604239	-4.408431076	0.000199534	0.037472627
cg22383292	-0.599710659	3.403102024	-4.408274725	0.000199613	0.037477374
cg04449239	-0.557842331	-1.706600044	-4.408043036	0.000199728	0.03748124
cg27117701	-0.473491028	3.9454016	-4.407986602	0.000199757	0.03748124
cg00077904	-0.585978051	-0.59735467	-4.407854762	0.000199823	0.03748124
cg03397483	-0.48382418	4.278321759	-4.407810666	0.000199845	0.03748124
cg13927504	-0.936895942	-0.358834084	-4.407205844	0.000200148	0.03751784
cg03348365	0.781815766	2.340181186	4.407093022	0.000200204	0.03751784
cg12853654	-0.674759497	3.398114838	-4.406995833	0.000200253	0.03751784
cg15082470	-0.497696277	2.583918215	-4.40698896	0.000200257	0.03751784
cg04142890	-0.467179932	-0.306685801	-4.406893136	0.000200305	0.03751784
cg01832757	-0.52605125	-0.630397479	-4.406708699	0.000200397	0.03752526
cg13329352	-0.40742716	3.656899767	-4.40656644	0.000200469	0.037528718
cg26808583	-0.460441134	1.38551448	-4.406300892	0.000200602	0.03753499
cg15415787	-0.435574012	-2.446097605	-4.406288848	0.000200608	0.03753499
cg19696891	-0.422458267	-0.522195137	-4.405930705	0.000200788	0.037549971
cg26295061	-0.450007061	3.754094711	-4.405918818	0.000200794	0.037549971
cg13781167	-0.420602996	0.38208325	-4.405778747	0.000200865	0.037553238
cg21135516	-0.405938338	4.085183596	-4.40566205	0.000200924	0.037554308
cg00081238	-0.562321223	3.12769555	-4.405414976	0.000201048	0.037563326
cg15900159	-0.464983931	3.800330655	-4.405255066	0.000201129	0.037563326
cg13723763	-0.544026973	2.664195615	-4.405248745	0.000201132	0.037563326
cg21684505	-0.484738373	3.530482922	-4.405145229	0.000201184	0.037563326
cg01016518	-0.633168138	3.296446025	-4.404747065	0.000201385	0.037590911
cg08054515	-0.523545944	3.819812083	-4.404536679	0.000201491	0.037600353
cg02529459	-0.678393265	2.511789383	-4.404436544	0.000201541	0.037600353
cg21247229	-0.527427028	3.401773789	-4.404155804	0.000201683	0.037615435
cg21140290	-0.700561769	3.113511264	-4.404066323	0.000201728	0.037615435
cg04331840	-0.468089539	2.920357028	-4.403790564	0.000201868	0.037621912
cg05386697	-0.652265809	3.045265094	-4.403787551	0.000201869	0.037621912
cg04858987	-0.447346044	3.646217134	-4.403660557	0.000201934	0.037623986
cg24869651	-0.372477101	3.229118257	-4.403355927	0.000202088	0.037630298
cg05958352	-0.450894969	-0.096662023	-4.403247865	0.000202143	0.037630298
cg23730696	-0.465552245	2.126014497	-4.403186819	0.000202174	0.037630298
cg25004385	-0.794807031	2.059672995	-4.403173933	0.00020218	0.037630298

cg01961682	-0.486762688	-0.074036132	-4.403039526	0.000202248	0.037633085
cg24422202	-0.510855648	3.514032619	-4.402917716	0.00020231	0.037634687
cg08222943	-0.500237049	3.927926808	-4.402701082	0.00020242	0.037645234
cg00685116	-0.743378562	-1.974253556	-4.402590323	0.000202476	0.037645799
cg09246668	-0.422892024	3.144375594	-4.402105437	0.000202722	0.037662502
cg16951105	-0.592843077	3.173694307	-4.402065482	0.000202742	0.037662502
cg15475255	-0.387371433	4.153106507	-4.401982647	0.000202784	0.037662502
cg20656951	-0.694543323	3.29492827	-4.401851157	0.000202851	0.037662502
cg02164502	-0.530563032	3.011443475	-4.401612436	0.000202973	0.037662502
cg08886318	-0.456175874	3.949213805	-4.401569749	0.000202994	0.037662502
cg22194885	-0.721839398	1.101355813	-4.401513834	0.000203023	0.037662502
cg04356440	-0.463762144	-0.220160645	-4.401473765	0.000203043	0.037662502
cg08710921	-0.487974682	-0.448888262	-4.401437148	0.000203062	0.037662502
cg14557842	-0.503221564	3.27329542	-4.401273787	0.000203145	0.037662502
cg25642172	-0.629741592	3.05840393	-4.401160093	0.000203203	0.037662502
cg24383674	-0.494025257	3.687911869	-4.40109025	0.000203238	0.037662502
cg09644422	-0.480118277	3.252563865	-4.400887009	0.000203342	0.037662502
cg04132851	0.720490022	-4.327093236	4.400820262	0.000203376	0.037662502
cg13666392	-0.539725464	-0.654351098	-4.400799451	0.000203386	0.037662502
cg12385305	-0.648743409	3.478301073	-4.400740729	0.000203416	0.037662502
cg17427666	0.698190806	-3.994060668	4.400577344	0.0002035	0.037668078
cg05607040	-0.47129909	3.903008596	-4.400392693	0.000203594	0.037670945
cg09635768	-0.533422058	2.703890452	-4.400338445	0.000203622	0.037670945
cg22346868	0.4107273	0.809481075	4.399299824	0.000204152	0.037704251
cg11020821	-0.461335727	3.831158259	-4.399270564	0.000204167	0.037704251
cg00723395	-0.571615838	-0.930185356	-4.398907754	0.000204353	0.037704251
cg16934684	-0.643962457	2.720196976	-4.398752538	0.000204432	0.037704251
cg05570237	-0.469705882	3.543288202	-4.398638983	0.00020449	0.037704251
cg13561532	-0.512708416	3.263226621	-4.398490929	0.000204566	0.037704251
cg00855299	-0.417685526	0.00565684	-4.398443277	0.000204591	0.037704251
cg00737548	-0.764489	2.922179352	-4.398014689	0.000204811	0.037704251
cg09095196	-0.600122293	3.119337134	-4.397871475	0.000204884	0.037704251
cg08194621	-0.494510324	4.192403908	-4.397753592	0.000204945	0.037704251
cg10072168	-0.560838634	-0.147304511	-4.39773867	0.000204952	0.037704251
cg11061150	-0.53925798	4.025814111	-4.397697652	0.000204973	0.037704251
cg07208267	-0.505446053	3.813887105	-4.39767162	0.000204987	0.037704251
cg01587411	-0.430598864	3.613932327	-4.397620698	0.000205013	0.037704251
cg14528315	-0.591916162	2.946241255	-4.397604778	0.000205021	0.037704251
cg27270451	-0.593210645	2.807291616	-4.397292825	0.000205181	0.037704251
cg27504302	-0.444896807	3.561698967	-4.397146586	0.000205257	0.037704251
cg24852203	0.443981438	2.002317516	4.39711025	0.000205275	0.037704251
cg02571944	-0.402155072	3.604181174	-4.397057551	0.000205302	0.037704251
cg23173416	-0.463782799	4.116022238	-4.397007808	0.000205328	0.037704251
cg11314141	-0.791781534	2.639480026	-4.396984567	0.00020534	0.037704251
cg26394870	-0.521792263	-1.017921482	-4.396943585	0.000205361	0.037704251
cg10375300	-0.521976705	3.86206734	-4.396861723	0.000205403	0.037704251
cg24694501	-0.733498072	-0.320775297	-4.396740016	0.000205466	0.037704251

cg06632790	-0.379350026	3.248854622	-4.396646952	0.000205514	0.037704251
cg15046909	-0.697375715	2.850876232	-4.396644389	0.000205515	0.037704251
cg15885139	-0.491797768	0.096765536	-4.39659877	0.000205538	0.037704251
cg04912563	-0.460673522	-0.293223232	-4.396416088	0.000205633	0.037704251
cg08436449	-0.399767314	1.020622238	-4.396295406	0.000205695	0.037704251
cg26223117	-0.512261752	2.938150304	-4.396064062	0.000205814	0.037704251
cg01912166	0.677687079	3.228802958	4.395965097	0.000205865	0.037704251
cg04062868	-1.040210288	1.861593355	-4.395884286	0.000205907	0.037704251
cg25957804	-0.390681858	-0.46737732	-4.395709509	0.000205997	0.037704251
cg20122656	-0.52120705	4.178445883	-4.395513176	0.000206098	0.037704251
cg20812378	-0.530433768	-1.096807976	-4.395438894	0.000206137	0.037704251
cg15722631	-0.494709426	3.114101088	-4.394940446	0.000206394	0.037704251
cg05819613	-0.43680055	0.695673671	-4.394907993	0.000206411	0.037704251
cg20538594	-0.574233079	3.068607842	-4.394887223	0.000206422	0.037704251
cg01593345	-0.585031698	-1.43569435	-4.3948426	0.000206445	0.037704251
cg19515398	-0.562101638	-0.12096867	-4.394680012	0.000206529	0.037704251
cg06701531	-0.628787806	2.792237087	-4.394647426	0.000206546	0.037704251
cg00313452	-0.579396894	-1.897940457	-4.394551689	0.000206595	0.037704251
cg01597973	-0.619838697	3.752018404	-4.394348084	0.000206701	0.037704251
cg21997164	-0.453595104	3.458615005	-4.394314082	0.000206718	0.037704251
cg26309054	-0.508470254	4.10994997	-4.394294363	0.000206729	0.037704251
cg06787912	-0.81479962	-1.023827102	-4.394135772	0.000206811	0.037704251
cg00589737	-0.482654325	3.795012312	-4.394064524	0.000206848	0.037704251
cg08250938	-0.437503956	3.926132622	-4.393914947	0.000206925	0.037704251
cg27572854	-0.565504605	3.255263256	-4.393820804	0.000206974	0.037704251
cg20165746	-0.543139006	3.764890877	-4.393808074	0.000206981	0.037704251
cg10843037	-0.529175728	0.318208312	-4.393720186	0.000207026	0.037704251
cg09349018	-0.577313706	0.276560134	-4.393643068	0.000207066	0.037704251
cg20424182	-0.658581304	2.4955885	-4.393508171	0.000207136	0.037704251
cg03518728	0.791237523	-3.091759567	4.393442555	0.00020717	0.037704251
cg21017348	-0.752533336	3.273180267	-4.393399512	0.000207193	0.037704251
cg15917640	-0.675679401	2.545214656	-4.393188943	0.000207302	0.037704251
cg03551062	-0.503007596	-0.838665944	-4.393130131	0.000207332	0.037704251
cg08277543	-0.458239001	2.808091468	-4.393080952	0.000207358	0.037704251
cg17952808	-0.636157947	-1.802147847	-4.393030875	0.000207384	0.037704251
cg07891543	-0.516555573	3.240789052	-4.393010943	0.000207394	0.037704251
cg22891003	-0.420261009	1.023487569	-4.392739032	0.000207536	0.037704251
cg17440336	-0.472966038	3.985730357	-4.392551592	0.000207633	0.037704251
cg20494738	-0.484489992	0.073407818	-4.39250621	0.000207657	0.037704251
cg24024511	-0.68238065	3.008621493	-4.392353277	0.000207736	0.037704251
cg23400921	-0.55724734	3.172347075	-4.392222439	0.000207805	0.037704251
cg13214474	-0.46771529	3.789150671	-4.392210991	0.000207811	0.037704251
cg00092427	-0.527993221	0.180522296	-4.392193042	0.00020782	0.037704251
cg01778565	-0.809791996	1.754811999	-4.392188134	0.000207822	0.037704251
cg03659680	-0.762899079	1.630501036	-4.392100328	0.000207868	0.037704251
cg01493978	-0.454837487	3.487134095	-4.392098126	0.000207869	0.037704251
cg13432560	-0.576060185	4.026670015	-4.392084084	0.000207877	0.037704251

cg09243721	-0.441765675	3.242552969	-4.392029555	0.000207905	0.037704251
cg27588356	-0.905440728	1.05833312	-4.392029456	0.000207905	0.037704251
cg23807661	-0.580410716	3.723188053	-4.391702675	0.000208075	0.037704251
cg21564045	-0.604674174	2.997714792	-4.391702108	0.000208076	0.037704251
cg08127131	-0.5406748	-1.882616671	-4.39161885	0.000208119	0.037704251
cg23606392	0.683343995	2.865758925	4.391418127	0.000208224	0.037704251
cg13563329	0.608438807	2.967564259	4.391322049	0.000208274	0.037704251
cg09558975	-0.504161578	3.674722932	-4.391294697	0.000208288	0.037704251
cg16172329	-0.497937995	3.4451748	-4.391151034	0.000208363	0.037704251
cg14113958	-0.890768492	0.262877575	-4.391069848	0.000208406	0.037704251
cg23729070	-0.506622309	3.332090447	-4.390843176	0.000208524	0.037704251
cg22599270	-0.471086136	3.770340245	-4.390826112	0.000208533	0.037704251
cg08903077	-0.64662214	0.530811714	-4.390824186	0.000208534	0.037704251
cg00301531	-0.506926089	0.636736493	-4.390821294	0.000208535	0.037704251
cg07872264	-0.586813012	3.256310613	-4.390804887	0.000208544	0.037704251
cg16734637	-0.458071682	0.031970972	-4.390605229	0.000208648	0.037704251
cg16739209	-0.724802415	2.85792834	-4.39054702	0.000208679	0.037704251
cg13597330	-0.516766351	3.976403403	-4.390496836	0.000208705	0.037704251
cg04827268	-0.569610399	3.491682464	-4.390387104	0.000208762	0.037704251
cg04122955	-0.580738853	2.784046562	-4.39032203	0.000208796	0.037704251
cg19407682	-0.620235832	3.327127122	-4.390279365	0.000208819	0.037704251
cg09683085	-0.403311451	4.082422472	-4.390151084	0.000208886	0.037704251
cg10911328	-0.443250718	4.132885011	-4.39001851	0.000208955	0.037704251
cg04458053	-0.681560745	-2.618032935	-4.390000158	0.000208965	0.037704251
cg26133339	-0.542042964	3.62533855	-4.389961438	0.000208985	0.037704251
cg23173301	-0.429205129	-0.058619246	-4.389828594	0.000209055	0.037704251
cg10802555	-0.614963939	2.525439004	-4.389821396	0.000209058	0.037704251
cg07601093	-0.661585545	2.816354721	-4.389797499	0.000209071	0.037704251
cg21805138	-0.549552256	0.745821654	-4.389669205	0.000209138	0.037706773
cg09137301	-0.482157955	-1.117591958	-4.389102404	0.000209435	0.037745495
cg06791083	-0.683112502	-0.483935022	-4.389007088	0.000209485	0.037745495
cg26226306	-0.807102524	1.91931643	-4.388954912	0.000209513	0.037745495
cg26657018	-0.504405228	0.062937348	-4.388690648	0.000209652	0.037758632
cg06784867	-0.419013796	0.291074005	-4.388324772	0.000209844	0.037758632
cg06461306	-0.624039113	-1.68651848	-4.388297324	0.000209858	0.037758632
cg20499126	-0.746526275	2.350839725	-4.388277958	0.000209868	0.037758632
cg06121332	-0.641489235	2.722728648	-4.388145761	0.000209938	0.037758632
cg00036585	0.594876293	4.408816439	4.3881169	0.000209953	0.037758632
cg10147237	-0.475394482	3.663428921	-4.387880115	0.000210078	0.037758632
cg01546472	0.705355856	2.555834086	4.387859658	0.000210088	0.037758632
cg13360215	-0.494952648	2.999355271	-4.387664132	0.000210191	0.037758632
cg14742920	-0.465900574	3.420076214	-4.387657757	0.000210195	0.037758632
cg12668824	-0.562508937	3.295835971	-4.387528463	0.000210263	0.037758632
cg14842825	-0.511951266	3.827119963	-4.387519958	0.000210267	0.037758632
cg15451825	-0.379732068	4.194043598	-4.387498599	0.000210279	0.037758632
cg12170649	-0.552647079	2.743453321	-4.387194579	0.000210439	0.037777828
cg04272684	-0.671558209	1.942710494	-4.386901487	0.000210593	0.037780678

cg14618583	-0.524651442	2.657763049	-4.386838525	0.000210627	0.037780678
cg06216505	-0.888616759	2.030473791	-4.386763566	0.000210666	0.037780678
cg02335363	-0.55624768	3.917328798	-4.386751239	0.000210673	0.037780678
cg19213045	-0.592640257	3.286221233	-4.386659051	0.000210721	0.037780678
cg15071140	-0.393950353	-0.537493921	-4.386390936	0.000210863	0.037796499
cg06533253	-0.785187513	2.001343272	-4.385986958	0.000211077	0.037825203
cg10653532	-0.556965077	3.014093896	-4.385752848	0.0002112	0.037835283
cg15627442	-0.459364377	4.310719641	-4.385535659	0.000211315	0.037835283
cg05292376	-0.412261108	0.137833106	-4.385280547	0.00021145	0.037835283
cg18959169	-0.557211447	2.864753867	-4.385105643	0.000211543	0.037835283
cg26569316	1.268177411	3.291892748	4.385042834	0.000211576	0.037835283
cg16116970	-0.562579661	3.649167641	-4.384876017	0.000211665	0.037835283
cg14997389	-0.513012199	-0.541657587	-4.384580523	0.000211822	0.037835283
cg07520292	-0.604397109	2.243794195	-4.384560198	0.000211832	0.037835283
cg04463911	-0.698331273	1.576601266	-4.384539723	0.000211843	0.037835283
cg09185632	-0.562604238	3.951392398	-4.384525546	0.000211851	0.037835283
cg27264051	-0.577123499	3.781071524	-4.384408221	0.000211913	0.037835283
cg19139370	-0.508881716	3.697094355	-4.384146893	0.000212052	0.037835283
cg04716977	-0.522846046	3.497342822	-4.383963934	0.000212149	0.037835283
cg20400915	-0.664142824	-2.125704814	-4.383958317	0.000212152	0.037835283
cg27592542	-0.58442492	3.605735329	-4.383888754	0.000212189	0.037835283
cg09461630	-0.630496166	3.368795808	-4.383791312	0.000212241	0.037835283
cg13872123	-0.552606878	0.694310616	-4.383790487	0.000212241	0.037835283
cg25792462	-0.538499168	2.512371184	-4.383779181	0.000212247	0.037835283
cg10213800	-0.639987702	3.464844102	-4.38374743	0.000212264	0.037835283
cg03332224	-0.556340792	3.324812968	-4.3837321	0.000212272	0.037835283
cg05147886	-0.532592943	-0.421304677	-4.383532814	0.000212378	0.037835283
cg05922773	-0.561189662	3.174395437	-4.383528181	0.000212381	0.037835283
cg13451882	-0.563710076	3.255253547	-4.383463301	0.000212415	0.037835283
cg01094918	-0.644051592	3.247948025	-4.383455573	0.000212419	0.037835283
cg04112602	-0.619267776	3.114151514	-4.383293603	0.000212506	0.037835283
cg13391521	-0.684557654	2.720835582	-4.383251928	0.000212528	0.037835283
cg16350729	-0.536577616	3.121514824	-4.383163693	0.000212575	0.037835283
cg25136868	-0.500133047	2.877646075	-4.382985092	0.00021267	0.037842709
cg16414530	-0.454107438	-0.496230344	-4.382767727	0.000212786	0.037846886
cg11077643	-0.640065487	2.556968483	-4.382738378	0.000212802	0.037846886
cg21799607	-0.402973034	0.013741527	-4.382640365	0.000212854	0.037846886
cg06696584	-0.781895402	2.870402465	-4.382276906	0.000213048	0.037856389
cg19589824	-0.450533431	3.459419268	-4.382169436	0.000213105	0.037856389
cg10531055	-1.051706545	2.78068365	-4.382081098	0.000213152	0.037856389
cg01644599	-0.441706073	0.83808432	-4.382022224	0.000213184	0.037856389
cg18348274	-0.712041214	-0.299795081	-4.382000572	0.000213195	0.037856389
cg16961528	-0.589016327	3.257457782	-4.38189856	0.00021325	0.037856389
cg05371502	-0.744813585	2.275094515	-4.381664319	0.000213375	0.037856389
cg04538695	-0.571133094	2.978838434	-4.381636948	0.00021339	0.037856389
cg09688971	-0.450040322	3.765125434	-4.381332839	0.000213552	0.037856389
cg21637072	-0.465380996	3.754106327	-4.381126304	0.000213663	0.037856389

cg14303079	-0.559643462	3.164114405	-4.381076354	0.000213689	0.037856389
cg21225096	-0.78946644	2.383465266	-4.381059692	0.000213698	0.037856389
cg24853977	-0.644062774	1.427796827	-4.381027208	0.000213716	0.037856389
cg07486487	-0.77353301	2.446255764	-4.380977691	0.000213742	0.037856389
cg16551026	-0.523809006	3.887911063	-4.380920458	0.000213773	0.037856389
cg05202777	-0.483598623	1.03750498	-4.380705093	0.000213888	0.037856389
cg18827501	-0.430157909	4.127265222	-4.380652985	0.000213916	0.037856389
cg21466781	0.970323563	2.596446355	4.380524083	0.000213985	0.037856389
cg03707970	-0.396415308	-0.718027002	-4.380522287	0.000213986	0.037856389
cg05162812	-0.464446595	3.356165738	-4.380493291	0.000214002	0.037856389
cg02614923	-0.397779626	0.102930963	-4.380395473	0.000214054	0.037856389
cg08023890	-0.616597121	2.55884269	-4.380295707	0.000214108	0.037856389
cg09295050	-0.393049927	0.296124195	-4.380160195	0.000214181	0.037856389
cg13038078	-0.695794587	2.845905625	-4.380058717	0.000214235	0.037856389
cg05825212	-0.524550509	3.168584394	-4.379983421	0.000214275	0.037856389
cg27039008	-0.624411235	2.497124222	-4.379943813	0.000214297	0.037856389
cg00337668	-0.74237099	2.466134542	-4.379598435	0.000214482	0.037864856
cg04522024	-0.807797948	2.077734427	-4.379521554	0.000214524	0.037864856
cg07679834	-0.58037195	2.915410721	-4.379436846	0.000214569	0.037864856
cg23778117	-0.64239523	3.438283336	-4.379365538	0.000214607	0.037864856
cg10365337	-0.509954846	3.691764034	-4.379329554	0.000214627	0.037864856
cg00626702	-0.481492843	-0.14532178	-4.379257784	0.000214665	0.037864856
cg00505280	-0.532700835	3.094378345	-4.379042342	0.000214781	0.037866148
cg05008457	-0.437805215	3.844778447	-4.378935029	0.000214839	0.037866148
cg23362229	-0.902721287	2.199478184	-4.37888704	0.000214865	0.037866148
cg05266786	-0.536701433	-0.478226898	-4.378815732	0.000214903	0.037866148
cg25298189	-0.359978649	-0.591929806	-4.37850079	0.000215073	0.037866148
cg15568266	-0.864714004	1.918956797	-4.378469402	0.00021509	0.037866148
cg17347386	-0.61044781	3.075379958	-4.378306734	0.000215177	0.037866148
cg15572396	-0.487765934	-0.431244052	-4.378210265	0.000215229	0.037866148
cg07200348	-0.358402424	-1.563858671	-4.378185725	0.000215243	0.037866148
cg07981080	-0.329407052	-0.750533957	-4.378088802	0.000215295	0.037866148
cg12429241	-0.89106763	1.3477175	-4.378086474	0.000215296	0.037866148
cg19111287	0.768918275	0.726669017	4.378053284	0.000215314	0.037866148
cg07043494	-0.50865009	3.424848263	-4.377862784	0.000215417	0.037871561
cg15508470	-0.484272172	3.775618124	-4.377737907	0.000215484	0.037871561
cg03179979	-0.811231853	1.461269119	-4.377597779	0.00021556	0.037871561
cg10938899	-0.430590542	3.494735485	-4.377520465	0.000215602	0.037871561
cg02222561	-0.712724761	2.559113448	-4.377501083	0.000215612	0.037871561
cg24337081	-0.529889397	0.475116677	-4.377370009	0.000215683	0.037874608
cg08688498	-0.718520153	2.738590348	-4.377242664	0.000215752	0.037875285
cg00385246	-0.533231719	-0.296336095	-4.377165039	0.000215794	0.037875285
cg23305420	-0.496856121	-0.217709074	-4.377016276	0.000215874	0.037880018
cg19702569	-0.710073455	-4.679731929	-4.376406445	0.000216204	0.037928542
cg19580576	-0.711860726	3.297934797	-4.376105479	0.000216367	0.037941366
cg09959355	-0.613052829	1.533531236	-4.375898235	0.00021648	0.037941366
cg07585257	-0.623309222	-0.511821681	-4.375816833	0.000216524	0.037941366

cg24841193	-0.715101042	1.759716602	-4.375683112	0.000216596	0.037941366
cg14484149	-0.591505857	3.330090637	-4.375581602	0.000216652	0.037941366
cg26690672	-0.507310709	2.966209098	-4.375488753	0.000216702	0.037941366
cg23854567	-0.485924064	-1.338344454	-4.375169532	0.000216875	0.037941366
cg14803804	-0.500487582	3.217741105	-4.375131304	0.000216896	0.037941366
cg19307584	-0.872710855	0.914915469	-4.374976539	0.00021698	0.037941366
cg12253698	-0.647430382	3.190547213	-4.374972911	0.000216982	0.037941366
cg14266611	-0.476936223	-0.624923162	-4.37490418	0.00021702	0.037941366
cg05955831	0.609007214	3.110336944	4.374898635	0.000217023	0.037941366
cg08492753	0.462536592	1.892312172	4.374833769	0.000217058	0.037941366
cg20668812	-0.555570214	2.840245872	-4.374806566	0.000217073	0.037941366
cg18475832	-0.650328814	2.396532702	-4.374763322	0.000217096	0.037941366
cg23985641	-0.775622978	0.590469996	-4.374632414	0.000217167	0.037941366
cg08397456	-0.604097781	4.093858386	-4.374568409	0.000217202	0.037941366
cg18180609	-0.569694216	3.817866946	-4.374475807	0.000217253	0.037941366
cg10136885	-0.611306935	-1.771091635	-4.37439782	0.000217295	0.037941366
cg22945982	-0.716358503	3.229545112	-4.374292689	0.000217352	0.037942008
cg03475101	-0.607186848	3.286520389	-4.374069395	0.000217474	0.037949257
cg15627993	-0.427472779	0.070367643	-4.37401977	0.000217501	0.037949257
cg09377308	-0.689251365	1.51175418	-4.373580124	0.000217741	0.037962141
cg19854744	-0.469639767	-0.81203753	-4.37354203	0.000217761	0.037962141
cg02039858	-0.552318823	-0.771546736	-4.373381535	0.000217849	0.037962141
cg12466037	-0.649623493	-0.216782442	-4.373165225	0.000217967	0.037962141
cg12008501	-0.506600261	-0.206226025	-4.373163098	0.000217968	0.037962141
cg25900943	-0.573384902	-1.156776957	-4.373149485	0.000217976	0.037962141
cg00518973	-0.560448508	2.742015418	-4.373120842	0.000217991	0.037962141
cg19487865	-0.568749502	-0.479299635	-4.373098557	0.000218004	0.037962141
cg03736807	-0.973828018	0.284773057	-4.372758721	0.000218189	0.037967109
cg02659138	-0.615674129	3.407175988	-4.372649009	0.000218249	0.037967109
cg21614161	0.676371268	2.46006457	4.372629283	0.00021826	0.037967109
cg01350636	-0.488761731	3.330487409	-4.372600386	0.000218276	0.037967109
cg17759616	-0.434612435	3.788526193	-4.372405657	0.000218382	0.037967109
cg00937691	1.050510107	-3.50459113	4.372369056	0.000218402	0.037967109
cg20429242	-0.700858541	2.688343108	-4.372360076	0.000218407	0.037967109
cg25886345	-0.443219342	3.957804283	-4.371972447	0.000218619	0.037992745
cg05425666	-0.598314612	3.592305766	-4.37173036	0.000218752	0.037992745
cg17554494	-0.602953694	3.587915121	-4.371634383	0.000218805	0.037992745
cg19462116	-0.641977758	2.965796741	-4.371630106	0.000218807	0.037992745
cg26609516	-0.568998266	3.97547713	-4.371399169	0.000218934	0.037992745
cg06121748	-0.523591194	3.918868675	-4.371391442	0.000218938	0.037992745
cg08996334	-0.540810834	3.184486198	-4.3713288	0.000218972	0.037992745
cg18546182	0.617452795	3.40058552	4.371256588	0.000219012	0.037992745
cg00552502	-0.803042403	2.940018112	-4.371210061	0.000219037	0.037992745
cg22454313	-0.687075075	-1.058172006	-4.370872937	0.000219222	0.038012675
cg14261271	-0.691384285	0.196525781	-4.37080531	0.00021926	0.038012675
cg23049907	-0.6241257	3.134323197	-4.370494623	0.00021943	0.038016
cg04461800	-0.595418298	3.605784465	-4.370417154	0.000219473	0.038016

cg04509954	-0.694039906	3.262616665	-4.370406921	0.000219479	0.038016
cg02069524	-0.671607326	-1.226840536	-4.370190891	0.000219597	0.038016
cg25424293	-0.602375337	3.129568202	-4.369958244	0.000219725	0.038016
cg24120717	-0.524944461	3.269534824	-4.369725055	0.000219854	0.038016
cg22475353	-0.506820399	-1.361916178	-4.36966946	0.000219884	0.038016
cg11284329	-0.735648344	-2.711951098	-4.369568544	0.00021994	0.038016
cg11291445	-0.637646559	3.461388205	-4.369567076	0.000219941	0.038016
cg18899355	-0.532941457	-0.757940444	-4.369554419	0.000219948	0.038016
cg16942595	-0.553168496	3.823888291	-4.369514716	0.00021997	0.038016
cg25740342	-0.521948926	3.119563622	-4.369486795	0.000219985	0.038016
cg04263575	-0.444820589	3.656543948	-4.369443949	0.000220009	0.038016
cg02345692	-0.712413735	1.228337177	-4.369309765	0.000220083	0.038016
cg08008105	-0.60534169	1.921642951	-4.369307759	0.000220084	0.038016
cg25946752	0.365522866	0.612761608	4.369104789	0.000220196	0.038021377
cg18085086	-0.464057942	4.056375582	-4.369056687	0.000220222	0.038021377
cg04095800	0.792749185	2.034225128	4.368899082	0.000220309	0.038027124
cg05276071	-0.69890521	3.352675243	-4.368732458	0.000220401	0.038031095
cg20663432	-0.540172075	2.728446132	-4.368651524	0.000220446	0.038031095
cg19249918	-0.481494356	3.08916705	-4.368565736	0.000220493	0.038031095
cg01271485	-0.499587061	0.23682537	-4.368312372	0.000220633	0.038038109
cg17709458	-0.487995045	-1.045310588	-4.368297796	0.000220641	0.038038109
cg01657761	-0.520110358	-0.544919617	-4.368184977	0.000220704	0.038039604
cg04552799	-0.468276976	4.116815116	-4.368070966	0.000220767	0.038041215
cg08500510	-0.506496326	-0.266020883	-4.367894586	0.000220864	0.038048773
cg16784215	-0.830609097	-1.347422248	-4.367511298	0.000221077	0.038067609
cg23690785	-0.512936135	3.822772056	-4.367502938	0.000221081	0.038067609
cg10070640	-0.657534121	2.965877671	-4.367285633	0.000221202	0.038078415
cg02550673	-0.739964821	0.365002565	-4.367195644	0.000221251	0.038078415
cg10564060	-1.001692064	2.565211891	-4.366663153	0.000221547	0.038088737
cg17975663	-0.463082226	-0.083721108	-4.366644239	0.000221557	0.038088737
cg00214140	-0.72725564	-1.09908359	-4.366597377	0.000221583	0.038088737
cg09479330	-0.643244287	-1.382170227	-4.366574536	0.000221596	0.038088737
cg15569292	-0.470907708	-1.421038116	-4.366534703	0.000221618	0.038088737
cg07048592	-0.433566087	0.289384492	-4.366377911	0.000221705	0.038088737
cg22837714	-0.481314913	-1.551622918	-4.366284791	0.000221757	0.038088737
cg09207377	-0.535321688	3.252700871	-4.366248858	0.000221777	0.038088737
cg11655410	-0.599645142	3.504750824	-4.366215612	0.000221795	0.038088737
cg23507980	-0.530177838	-0.837919215	-4.366054693	0.000221885	0.03809486
cg20731316	-0.506435323	4.027724184	-4.365693528	0.000222086	0.038120105
cg22951316	-0.825115338	3.31243271	-4.36555566	0.000222162	0.038124036
cg13809964	-0.505331082	3.568465227	-4.365446959	0.000222223	0.038125184
cg07242524	-0.51747065	-1.502429818	-4.365331052	0.000222287	0.038127022
cg09908110	-0.853304414	-1.862922603	-4.36510029	0.000222416	0.038139836
cg24426924	-0.515490989	4.387266759	-4.364987743	0.000222479	0.038141358
cg16048522	-0.601486001	-0.944118756	-4.364695782	0.000222641	0.038150837
cg25014676	-0.528133964	3.02006226	-4.364695397	0.000222642	0.038150837
cg00910222	-0.659490708	0.191813563	-4.364478939	0.000222762	0.038152694

cg01376795	-0.554821352	2.296022421	-4.36447396	0.000222765	0.038152694
cg08223225	-0.556794823	0.149837917	-4.364386399	0.000222814	0.038152694
cg00714758	-0.699053102	2.362854368	-4.364263392	0.000222883	0.038155231
cg13277131	-0.543541432	-0.655396576	-4.363894149	0.000223089	0.038174119
cg18078217	-0.352126849	-0.430402167	-4.36381164	0.000223135	0.038174119
cg23661026	-0.63391722	3.356298839	-4.363776553	0.000223155	0.038174119
cg15350946	-0.518000043	2.421269893	-4.363382848	0.000223375	0.038187622
cg03421507	-0.605757672	-1.952699763	-4.363350476	0.000223393	0.038187622
cg21537915	-0.547682034	3.970532502	-4.363345675	0.000223396	0.038187622
cg00044497	-0.647952193	3.395016418	-4.36322171	0.000223465	0.038187622
cg15573406	-0.505946295	4.255869251	-4.363147225	0.000223507	0.038187622
cg00442704	-0.46064512	3.212713601	-4.363057449	0.000223557	0.038187622
cg12111442	-0.751327516	-0.477170402	-4.362942991	0.000223621	0.038189364
cg12686731	-0.755240733	3.321734827	-4.362599088	0.000223814	0.038210099
cg12499271	-0.682417363	2.877074866	-4.362533909	0.000223851	0.038210099
cg22087540	-0.489847459	-0.43056009	-4.36215905	0.000224061	0.038214791
cg21888978	-0.808632974	3.284224248	-4.362114493	0.000224086	0.038214791
cg07079914	-0.559018415	4.041961282	-4.362103436	0.000224092	0.038214791
cg27349276	-0.571579495	2.987743053	-4.362031573	0.000224132	0.038214791
cg03737787	-0.931277326	0.093510726	-4.362004261	0.000224148	0.038214791
cg23331241	-0.686159377	3.121080451	-4.361819905	0.000224251	0.038219121
cg22125214	-0.543143764	3.646383428	-4.361766941	0.000224281	0.038219121
cg26056522	-0.635316532	3.016307564	-4.361348698	0.000224516	0.038245414
cg22109433	-0.543713535	-1.102124659	-4.361300428	0.000224543	0.038245414
cg08548542	-0.711364215	3.025966507	-4.360822594	0.000224812	0.038282019
cg22403060	-0.49692097	3.836036677	-4.360712395	0.000224874	0.038283386
cg08893117	-0.543608205	2.532609139	-4.360255843	0.000225132	0.038298064
cg16536513	-0.599415891	3.727074242	-4.360221563	0.000225151	0.038298064
cg25460263	-0.903119856	1.55245032	-4.360176761	0.000225176	0.038298064
cg01019082	-0.832022321	3.562013189	-4.360037829	0.000225255	0.038298064
cg03477789	-0.652120667	2.798338938	-4.359707592	0.000225441	0.038298064
cg09921497	-0.593207968	3.537103956	-4.359622082	0.000225489	0.038298064
cg19287711	-0.599298917	4.678651742	-4.35961677	0.000225492	0.038298064
cg15659527	-0.64285185	0.332323724	-4.359536398	0.000225538	0.038298064
cg09912525	-0.530018447	1.804752488	-4.359531609	0.000225541	0.038298064
cg21583016	-0.669600157	-0.601670769	-4.359376864	0.000225628	0.038298064
cg10213756	-0.564155597	3.733296075	-4.359309853	0.000225666	0.038298064
cg09403144	-0.570521703	2.938774249	-4.359222009	0.000225716	0.038298064
cg20738553	-0.539851156	3.943135879	-4.359179304	0.00022574	0.038298064
cg27583988	0.555996893	2.76160804	4.359132688	0.000225766	0.038298064
cg00954255	-0.554301591	3.542174892	-4.358940774	0.000225875	0.038298064
cg23331837	-0.496071384	3.576826821	-4.35893192	0.00022588	0.038298064
cg10811485	-0.557192446	-2.309221619	-4.358931873	0.00022588	0.038298064
cg18845937	-0.794529924	2.602468759	-4.358670974	0.000226027	0.038313933
cg06720747	-0.585906849	3.698408321	-4.358512067	0.000226117	0.038320019
cg21775245	-0.92840419	-2.180614305	-4.358223755	0.000226281	0.038327282
cg16361444	-0.601340829	3.513315069	-4.35809279	0.000226355	0.038327282

cg21810086	-0.690846971	2.760516739	-4.357990807	0.000226413	0.038327282
cg02249398	-0.53438662	3.729827191	-4.357878909	0.000226476	0.038327282
cg26940620	-0.382823994	0.090419483	-4.357805863	0.000226518	0.038327282
cg01359165	-0.523579114	-0.03658381	-4.357691859	0.000226582	0.038327282
cg15385778	-0.404339339	3.970187522	-4.357668595	0.000226596	0.038327282
cg02300890	-0.469028077	-0.147608139	-4.35748652	0.000226699	0.038327282
cg00755146	-0.512565086	-0.281586684	-4.357216043	0.000226853	0.038327282
cg27103048	-0.511097415	2.886853944	-4.357131671	0.000226901	0.038327282
cg18728109	-0.486972801	4.376263288	-4.357120788	0.000226907	0.038327282
cg02335656	-0.722134567	-1.02980755	-4.357027772	0.00022696	0.038327282
cg16957923	-0.425484149	-0.525249988	-4.3570265	0.00022696	0.038327282
cg22966887	-0.627122134	3.633899038	-4.356974761	0.00022699	0.038327282
cg09473494	-0.590525096	3.501403859	-4.356914811	0.000227024	0.038327282
cg25530613	-0.470449912	2.886236397	-4.356795579	0.000227092	0.038327282
cg21927177	-0.574554336	-1.493377969	-4.356733034	0.000227127	0.038327282
cg16885600	-0.666509166	-0.918971278	-4.356721171	0.000227134	0.038327282
cg05049980	-0.475837669	4.197255087	-4.356532557	0.000227241	0.038333434
cg00283409	-0.881329473	0.217474787	-4.356344491	0.000227349	0.038333434
cg05097308	-0.533098438	3.732533995	-4.356077798	0.0002275	0.038333434
cg27571061	-0.570673866	2.00149718	-4.355996396	0.000227547	0.038333434
cg24820250	-0.496918891	3.700672386	-4.355994971	0.000227548	0.038333434
cg08749095	-0.463934946	3.22396268	-4.355985013	0.000227553	0.038333434
cg17576960	-0.555719976	-0.157859482	-4.355829425	0.000227642	0.038333434
cg24053343	-0.675797782	3.253748639	-4.355467785	0.000227848	0.038333434
cg27411301	-0.657496916	3.081812211	-4.355451413	0.000227858	0.038333434
cg26082707	-0.559631067	3.78322173	-4.355428939	0.000227871	0.038333434
cg17536465	-0.317368506	-0.471213795	-4.355320828	0.000227932	0.038333434
cg08448262	-0.582572154	3.486469059	-4.355316082	0.000227935	0.038333434
cg24414383	-0.517599325	3.749191108	-4.355282932	0.000227954	0.038333434
cg18060849	0.48704745	3.637931736	4.355215841	0.000227992	0.038333434
cg14199187	-0.433777813	-1.664262851	-4.355149534	0.00022803	0.038333434
cg04401043	-0.394085315	-1.432512093	-4.355138588	0.000228036	0.038333434
cg18593660	-0.663201016	0.593462405	-4.354699386	0.000228287	0.038366529
cg09014973	-0.440891202	2.594013198	-4.354482518	0.000228411	0.038378274
cg26675933	-0.624121132	2.715354477	-4.354200286	0.000228573	0.038380524
cg15961533	-0.828129016	2.064393031	-4.354007351	0.000228684	0.038380524
cg17258226	-0.586679466	3.687649342	-4.353963127	0.000228709	0.038380524
cg15718555	-0.524346046	3.51615488	-4.353904717	0.000228742	0.038380524
cg11182322	-0.611520103	3.020498575	-4.353868669	0.000228763	0.038380524
cg16000097	-0.523377306	-1.093346527	-4.353809556	0.000228797	0.038380524
cg12760533	-0.745816317	0.76397632	-4.353722268	0.000228847	0.038380524
cg12458020	-0.863831413	1.125811785	-4.353702374	0.000228858	0.038380524
cg13731777	-0.528166685	2.894988022	-4.35348889	0.000228981	0.038387133
cg02454884	0.641816242	-4.473622638	4.353444674	0.000229006	0.038387133
cg27237756	-0.387884806	4.090177084	-4.353053166	0.000229231	0.038409108
cg08244422	-0.410399469	3.654325078	-4.353016658	0.000229252	0.038409108
cg19047698	-0.786407138	2.959443161	-4.352932952	0.0002293	0.038409108

cg22034261	-0.605209823	1.806573749	-4.352670385	0.000229451	0.038410189
cg14712766	-0.44621846	3.81035595	-4.352592928	0.000229495	0.038410189
cg10859566	-0.717169561	1.106224173	-4.352538952	0.000229526	0.038410189
cg04855748	-0.395294422	-1.032154653	-4.352198205	0.000229722	0.038410189
cg07497042	-0.549166702	3.593939419	-4.352185305	0.00022973	0.038410189
cg05572930	-0.532952709	3.698220344	-4.352147951	0.000229751	0.038410189
cg00503269	-0.468002857	3.553071715	-4.35209836	0.00022978	0.038410189
cg02917683	-0.601216206	0.511821183	-4.35187721	0.000229907	0.038410189
cg13861413	-0.663945581	2.322068922	-4.351835389	0.000229931	0.038410189
cg25023822	-0.453171409	-2.001415638	-4.351773086	0.000229967	0.038410189
cg00202913	-0.742751288	2.501033806	-4.351763082	0.000229973	0.038410189
cg06328829	-0.542820022	0.022765835	-4.351686073	0.000230017	0.038410189
cg00845239	-0.439312162	0.400436036	-4.351595247	0.00023007	0.038410189
cg20369378	-0.656903544	3.525107182	-4.351586353	0.000230075	0.038410189
cg16030541	-0.503882638	3.254574318	-4.35137202	0.000230198	0.038410189
cg06722917	-0.461444543	3.674775001	-4.351321689	0.000230227	0.038410189
cg13306208	-0.381783624	3.680420412	-4.351267616	0.000230258	0.038410189
cg26326061	0.507467635	3.446973354	4.351198698	0.000230298	0.038410189
cg02362016	-0.550720019	3.785680592	-4.351077955	0.000230368	0.038410189
cg07933106	-0.463379622	3.719884321	-4.351038314	0.000230391	0.038410189
cg04271164	-0.533724053	3.790594213	-4.350802292	0.000230527	0.038423863
cg06617335	-0.482812019	-1.261069278	-4.350451123	0.00023073	0.038441391
cg21593554	-0.599519263	3.104707027	-4.350278055	0.00023083	0.038441391
cg03620729	0.589285139	2.490136422	4.350257826	0.000230842	0.038441391
cg08714076	-0.43307046	-0.43524478	-4.350213528	0.000230867	0.038441391
cg17990967	-0.52569267	3.733706632	-4.350096239	0.000230935	0.038441391
cg11927951	-0.937626861	2.153149869	-4.350048218	0.000230963	0.038441391
cg12274479	-0.665312119	2.320580204	-4.349866904	0.000231068	0.038441391
cg02097259	-0.577070866	3.046049818	-4.349788379	0.000231113	0.038441391
cg23755506	-0.438107311	4.136491129	-4.349693076	0.000231168	0.038441391
cg01171579	-0.415194277	3.721621767	-4.349611607	0.000231216	0.038441391
cg24779843	-0.478361471	-4.010783528	-4.349445317	0.000231312	0.038441391
cg26518932	-0.575527539	4.188939392	-4.349407991	0.000231334	0.038441391
cg17588181	-0.714293978	3.026700903	-4.349401044	0.000231338	0.038441391
cg03735233	0.507392767	1.613896623	4.349289602	0.000231402	0.038443106
cg12203044	-0.435560531	-0.559619687	-4.34893422	0.000231608	0.038463568
cg16383005	-0.510200097	-0.530346033	-4.348889996	0.000231634	0.038463568
cg05350384	-0.469269149	4.269639668	-4.348699263	0.000231745	0.038468382
cg05161357	-0.557133246	3.58786403	-4.348574074	0.000231817	0.038468382
cg18732064	-0.437503847	-1.254454392	-4.348469661	0.000231878	0.038468382
cg13915886	-0.389859495	0.586020759	-4.348457994	0.000231885	0.038468382
cg22521416	-0.590032782	2.823414373	-4.348372434	0.000231935	0.038468382
cg01872024	-0.409894604	-1.576085363	-4.348157601	0.000232059	0.03848008
cg10283179	-0.472450649	4.242945965	-4.347960298	0.000232174	0.038483478
cg21825026	-0.793222889	2.185478108	-4.347701923	0.000232324	0.038483478
cg03170099	-0.501566682	3.87339409	-4.347661239	0.000232348	0.038483478
cg22209723	-0.577901671	1.007624565	-4.347549977	0.000232413	0.038483478

cg06159593	-0.410445244	3.281054285	-4.347465516	0.000232462	0.038483478
cg02673323	-0.515699953	4.067937447	-4.347438167	0.000232478	0.038483478
cg18396668	-0.601633061	2.397603231	-4.347372967	0.000232516	0.038483478
cg07774964	-0.491386152	-1.464747867	-4.34734416	0.000232533	0.038483478
cg11298535	-0.623252273	3.152138075	-4.347282232	0.000232569	0.038483478
cg16339745	-0.352001616	4.089024047	-4.346943574	0.000232766	0.038498525
cg25845898	0.687811408	-3.56426811	4.346939732	0.000232768	0.038498525
cg18675416	-0.500004816	1.862678481	-4.346103934	0.000233256	0.038564321
cg18467053	-0.934509296	2.007748112	-4.346016337	0.000233307	0.038564321
cg12079279	-0.527118747	2.314087246	-4.345799324	0.000233434	0.038564321
cg03729676	-0.791075093	-0.021039952	-4.345754515	0.000233461	0.038564321
cg03240727	-0.97170242	2.546516031	-4.345711972	0.000233485	0.038564321
cg05184101	-0.411539168	-0.047672779	-4.345699219	0.000233493	0.038564321
cg06462301	-0.52264168	3.501972777	-4.345200625	0.000233785	0.038584332
cg07834510	-0.541592261	3.889886149	-4.345200086	0.000233785	0.038584332
cg16771103	-0.584845078	3.145326888	-4.344939733	0.000233938	0.038584332
cg06619035	-1.145361367	0.400947935	-4.344284496	0.000234322	0.038584332
cg24159511	-0.52045494	3.187129084	-4.344248648	0.000234343	0.038584332
cg25644624	-0.615848213	3.17677056	-4.344145087	0.000234404	0.038584332
cg15894683	-0.737968367	3.158839505	-4.344136662	0.000234409	0.038584332
cg05511024	-0.479539813	3.098345999	-4.344067834	0.000234449	0.038584332
cg08268031	-0.562435261	2.720568376	-4.343796404	0.000234608	0.038584332
cg17575972	0.474689435	1.094145874	4.343711105	0.000234659	0.038584332
cg16234890	-0.609003312	3.017672936	-4.343657407	0.00023469	0.038584332
cg02867709	-0.490031405	-0.3236869	-4.34362066	0.000234712	0.038584332
cg06005913	-0.588057782	0.763967642	-4.343547442	0.000234755	0.038584332
cg19289607	-0.581100965	2.346246441	-4.343503715	0.000234781	0.038584332
cg22014026	0.633016061	3.924681495	4.343285162	0.000234909	0.038584332
cg13850024	-0.666471134	3.178410135	-4.34304377	0.000235051	0.038584332
cg13785068	-0.559297166	-0.883356533	-4.343039814	0.000235054	0.038584332
cg10071822	-0.680838991	4.17056612	-4.342972534	0.000235093	0.038584332
cg01817009	-0.511849481	-0.622968165	-4.342934724	0.000235115	0.038584332
cg04091926	0.554795304	3.215359475	4.342732264	0.000235235	0.038584332
cg08578312	-0.758073015	2.514395213	-4.342719222	0.000235242	0.038584332
cg14948804	-0.475215249	3.013183432	-4.342660877	0.000235277	0.038584332
cg27634130	-0.601244783	3.682412065	-4.342658088	0.000235278	0.038584332
cg02336364	-0.577036534	-0.267273385	-4.342408311	0.000235426	0.038584332
cg11179537	-0.574210082	3.38133915	-4.342367392	0.00023545	0.038584332
cg20779334	-0.470378138	-0.896459393	-4.342338307	0.000235467	0.038584332
cg03053291	0.502098188	-0.719126212	4.342179982	0.00023556	0.038584332
cg04599589	-0.602484512	2.713327399	-4.342161494	0.000235571	0.038584332
cg02889672	0.976696918	-1.629618953	4.342130262	0.00023559	0.038584332
cg05521874	-0.541031739	3.788139231	-4.342084316	0.000235617	0.038584332
cg15029438	-0.462834496	-0.574276548	-4.342048368	0.000235638	0.038584332
cg20155876	-0.439181603	1.169564349	-4.341943972	0.0002357	0.038584332
cg00204743	-0.488953141	-0.579077626	-4.341801615	0.000235784	0.038584332
cg02561081	-0.660121835	2.409032846	-4.341770659	0.000235802	0.038584332

cg27438949	-0.579151976	3.678963549	-4.341713052	0.000235836	0.038584332
cg09029704	-0.799003828	2.206589435	-4.341618181	0.000235892	0.038584332
cg06297160	-0.926227479	2.133919743	-4.341566778	0.000235922	0.038584332
cg10809560	-0.54616599	-0.410330455	-4.341562718	0.000235925	0.038584332
cg05478128	-0.648750673	3.196982121	-4.34148816	0.000235969	0.038584332
cg07520341	-0.435871336	4.120949081	-4.341430129	0.000236003	0.038584332
cg11297399	-0.494665881	-0.76633683	-4.341406248	0.000236017	0.038584332
cg18067749	0.683613225	3.237912776	4.341362361	0.000236043	0.038584332
cg07075061	-0.537480612	4.171024257	-4.341149745	0.000236169	0.038584332
cg19697663	-0.944822448	3.304025799	-4.340998353	0.000236259	0.038584332
cg25528822	-0.491187419	1.630570711	-4.3409676	0.000236277	0.038584332
cg11757326	-0.429782353	3.911465587	-4.340884202	0.000236326	0.038584332
cg26529437	-0.637427582	1.616596328	-4.340850911	0.000236346	0.038584332
cg25162270	-0.627637841	3.168975662	-4.340778251	0.000236389	0.038584332
cg17936229	-0.80030913	1.024164316	-4.340636027	0.000236473	0.038584332
cg20698406	-0.614840668	2.656155044	-4.340601321	0.000236494	0.038584332
cg13907089	-0.61298396	2.697308262	-4.340578182	0.000236507	0.038584332
cg09488450	-0.443589686	-0.771196073	-4.340517297	0.000236544	0.038584332
cg25386707	-0.51948173	-1.462190049	-4.340472758	0.00023657	0.038584332
cg02929350	-0.837606326	2.040686517	-4.340452195	0.000236582	0.038584332
cg13240235	-0.807094654	-0.196474198	-4.340391826	0.000236618	0.038584332
cg01662578	-0.715283155	-0.719643607	-4.340268869	0.000236691	0.038584332
cg00803368	-0.775618764	0.307814209	-4.340194001	0.000236735	0.038584332
cg20185964	-0.732205977	1.377464734	-4.340129876	0.000236773	0.038584332
cg09141610	-0.565155874	0.26122232	-4.339833244	0.000236949	0.038604132
cg16300263	-0.360741989	3.857281023	-4.339734328	0.000237008	0.03860482
cg14912033	-0.502704524	-1.285176474	-4.339444427	0.00023718	0.038617098
cg02107173	-0.459822414	3.455229494	-4.339413844	0.000237198	0.038617098
cg26290885	-0.616309103	0.284820066	-4.339165629	0.000237346	0.038617098
cg12609322	-0.493325341	-0.211125909	-4.339104766	0.000237382	0.038617098
cg07671622	-0.381155292	-0.533010808	-4.339058388	0.00023741	0.038617098
cg18173388	-0.549856976	3.46732723	-4.339057002	0.00023741	0.038617098
cg05358326	-0.494479768	-0.141553212	-4.338849182	0.000237534	0.038627489
cg14766524	-0.534410308	3.886221885	-4.338625965	0.000237667	0.038627489
cg03554194	-0.613397722	3.427467052	-4.338513936	0.000237734	0.038627489
cg07507599	-0.783686866	1.05424778	-4.338502801	0.00023774	0.038627489
cg11661479	-0.60076949	3.549414321	-4.338491515	0.000237747	0.038627489
cg17542592	-0.732760798	2.591181734	-4.338239723	0.000237897	0.038629252
cg24207750	-0.86741079	3.184104638	-4.338119327	0.000237969	0.038629252
cg09961896	-0.444722107	3.969744333	-4.337984518	0.000238049	0.038629252
cg23689426	-0.517105024	3.105636856	-4.337796711	0.000238161	0.038629252
cg16858144	-0.632427405	2.857384169	-4.337655241	0.000238245	0.038629252
cg16866520	-0.546349022	3.624615677	-4.337581941	0.000238289	0.038629252
cg12595439	-0.466853092	3.517184288	-4.337528369	0.000238321	0.038629252
cg24342987	-0.700763831	1.837958308	-4.337289046	0.000238464	0.038629252
cg11025439	-0.450526041	3.158841487	-4.337273253	0.000238474	0.038629252
cg01945532	-0.518749422	3.692684147	-4.337246568	0.000238489	0.038629252

cg09852411	-0.468701777	3.892019919	-4.337175131	0.000238532	0.038629252
cg17561096	-0.54739074	1.082915542	-4.337133219	0.000238557	0.038629252
cg14914887	-0.484672467	3.997113793	-4.336953655	0.000238665	0.038629252
cg10578504	-0.617901311	2.684388346	-4.336952386	0.000238665	0.038629252
cg26035961	-0.465170929	3.26617446	-4.336816377	0.000238747	0.038629252
cg19079351	-1.082923096	0.34163396	-4.336805727	0.000238753	0.038629252
cg17448641	-0.476902333	4.17178645	-4.336783656	0.000238766	0.038629252
cg08172581	-0.564117145	-0.14744769	-4.336760614	0.00023878	0.038629252
cg08192812	-0.518144054	-0.244870981	-4.336713042	0.000238808	0.038629252
cg01204185	-0.442060175	4.095196422	-4.336415146	0.000238987	0.038629252
cg06504624	-0.737647335	2.70244579	-4.336394478	0.000238999	0.038629252
cg18057688	-0.764626134	2.332022416	-4.336387757	0.000239003	0.038629252
cg27411273	-0.468067095	0.690528335	-4.336353487	0.000239024	0.038629252
cg12865252	-0.523067793	3.124285062	-4.336281805	0.000239066	0.038629252
cg23741330	-0.489156999	-1.054685982	-4.336185033	0.000239124	0.038629805
cg02243249	-0.466186307	-0.389095922	-4.33601576	0.000239226	0.038637377
cg22731164	-0.532517718	3.659705663	-4.33579668	0.000239357	0.038644213
cg06992342	-0.630688375	2.765063031	-4.335763131	0.000239377	0.038644213
cg00969319	0.496679029	1.135306946	4.33547117	0.000239552	0.038646122
cg05073233	-0.443693471	3.688579177	-4.335412046	0.000239588	0.038646122
cg06389267	-0.692681001	3.150223452	-4.335404281	0.000239593	0.038646122
cg10135682	-0.401217453	3.507374627	-4.335379659	0.000239607	0.038646122
cg10790394	-0.468869723	3.252800029	-4.334997088	0.000239837	0.038665998
cg14101998	-0.403120169	-0.256313806	-4.334802454	0.000239954	0.038665998
cg01618923	-0.650710795	-0.8141585	-4.334769182	0.000239974	0.038665998
cg11635155	-0.43274952	3.779675358	-4.334682991	0.000240026	0.038665998
cg03215909	-0.491806167	3.185274924	-4.33446576	0.000240156	0.038665998
cg15864152	-0.838022725	2.310823492	-4.334387969	0.000240203	0.038665998
cg11925907	-0.558277096	3.755297038	-4.33435879	0.000240221	0.038665998
cg12348705	-0.878014548	-0.616491961	-4.334341696	0.000240231	0.038665998
cg24881472	-0.58520894	3.825739115	-4.334314321	0.000240248	0.038665998
cg22763593	-0.559788075	-1.420120772	-4.33419871	0.000240317	0.038665998
cg03432278	-0.43138595	4.230948668	-4.334161484	0.00024034	0.038665998
cg05372370	-0.530757139	3.688383901	-4.334085098	0.000240386	0.038665998
cg02527578	-0.553783793	3.580909267	-4.333946332	0.000240469	0.03867066
cg09048128	-0.580235039	2.727682779	-4.333638904	0.000240654	0.038682317
cg10124928	-0.785760717	2.628299025	-4.333612044	0.000240671	0.038682317
cg23378722	-0.551881477	-1.807577595	-4.333496711	0.00024074	0.038682317
cg00432369	-0.627957718	2.747781402	-4.33342646	0.000240782	0.038682317
cg17601288	-0.454009477	1.178618538	-4.333291509	0.000240864	0.038682317
cg15621651	-0.804048794	-0.972344191	-4.333253327	0.000240887	0.038682317
cg23417673	0.614435924	2.608535306	4.333192105	0.000240924	0.038682317
cg19115205	-0.512933982	4.280154984	-4.332873089	0.000241116	0.038696611
cg23800415	-0.682378417	2.375318173	-4.332738118	0.000241198	0.038696611
cg10704012	-0.779082093	2.529652795	-4.332717808	0.00024121	0.038696611
cg07360518	-0.723567951	2.900791187	-4.332682821	0.000241231	0.038696611
cg13504933	0.602034227	3.320940483	4.332520067	0.00024133	0.03870127

cg02915920	-0.525084902	-1.357501882	-4.33242142	0.000241389	0.03870127
cg05253370	0.50756077	2.552920732	4.332363639	0.000241424	0.03870127
cg16371838	-0.543226004	3.427736845	-4.331976634	0.000241659	0.038714292
cg06865309	-0.518360136	3.60104142	-4.331906362	0.000241701	0.038714292
cg00538691	-0.471500189	-0.16637933	-4.331807979	0.000241761	0.038714292
cg16829258	-0.61128583	3.789584096	-4.331798748	0.000241766	0.038714292
cg20899151	-0.537085613	3.886688598	-4.331608782	0.000241881	0.038714292
cg13057506	-0.5413388	1.584408464	-4.331395673	0.00024201	0.038714292
cg08271852	-0.450546326	-1.157283077	-4.331286403	0.000242077	0.038714292
cg18147900	-0.519192613	3.383204516	-4.3312255	0.000242114	0.038714292
cg07204486	-0.500689908	3.877972389	-4.33115297	0.000242158	0.038714292
cg11648212	-0.563667921	3.073892329	-4.330855874	0.000242338	0.038714292
cg04470564	-0.530170005	3.351169615	-4.330700932	0.000242432	0.038714292
cg08985853	-0.517398703	3.110002927	-4.330655455	0.00024246	0.038714292
cg00652926	-0.400278088	4.04422482	-4.330558576	0.000242518	0.038714292
cg01463702	-1.043369894	2.207444296	-4.330522798	0.00024254	0.038714292
cg22383447	-0.673162601	3.71710041	-4.330500054	0.000242554	0.038714292
cg18690301	-0.646620027	3.15627886	-4.330477071	0.000242568	0.038714292
cg15575683	-0.43271199	-1.798445845	-4.330413888	0.000242606	0.038714292
cg20408707	-0.487216282	4.249234762	-4.329969258	0.000242877	0.038714292
cg04890780	0.62296792	1.641037168	4.329869309	0.000242937	0.038714292
cg18903413	-0.563972115	3.319461364	-4.329790042	0.000242986	0.038714292
cg07702548	-0.663678799	-2.872233116	-4.329739035	0.000243017	0.038714292
cg01874933	-0.495988845	3.909881416	-4.329701328	0.00024304	0.038714292
cg17835106	-0.49082079	2.595107883	-4.329683173	0.000243051	0.038714292
cg21733927	-0.353930803	-0.694546434	-4.329580095	0.000243114	0.038714292
cg02994670	-0.624260275	2.830414171	-4.329536969	0.00024314	0.038714292
cg17129519	1.552045021	3.236919843	4.329444759	0.000243196	0.038714292
cg05902191	-0.483604951	-0.050026517	-4.329383665	0.000243233	0.038714292
cg11979205	-0.468390896	-0.285744057	-4.32936735	0.000243243	0.038714292
cg24470792	-0.630670039	3.116182601	-4.329309662	0.000243278	0.038714292
cg26572776	-0.683700575	-0.209232296	-4.32906105	0.00024343	0.038714292
cg03642549	-0.469634394	3.56353875	-4.328996148	0.000243469	0.038714292
cg22117688	-0.569915357	4.02009195	-4.328905929	0.000243524	0.038714292
cg05263725	-0.782442641	2.040338594	-4.328902074	0.000243527	0.038714292
cg07640566	-0.525476981	-0.223118601	-4.328840247	0.000243564	0.038714292
cg10953415	-0.830232867	1.34370756	-4.328777579	0.000243603	0.038714292
cg21861901	-0.548545907	-0.193568566	-4.328294421	0.000243898	0.038714292
cg22910462	-0.665951189	-0.61262056	-4.327860387	0.000244163	0.038714292
cg01020840	-0.482503819	3.988728615	-4.327848741	0.00024417	0.038714292
cg05541607	-0.759604152	2.320646218	-4.327502438	0.000244382	0.038714292
cg01522820	-0.389662192	3.802380823	-4.327469279	0.000244402	0.038714292
cg17190161	-0.423051955	4.435705721	-4.327423281	0.00024443	0.038714292
cg05317931	-0.486126389	3.430810977	-4.327396553	0.000244447	0.038714292
cg02607779	-0.769054753	-3.35341286	-4.327349155	0.000244476	0.038714292
cg21301879	-0.573926134	3.661819151	-4.327345403	0.000244478	0.038714292
cg08656172	-0.550894106	3.80860634	-4.327295316	0.000244509	0.038714292

cg19370097	-0.532481162	-1.945946702	-4.327255415	0.000244533	0.038714292
cg12990411	-0.653886691	2.925589046	-4.327241347	0.000244542	0.038714292
cg21334045	-0.626524745	2.827436711	-4.327224069	0.000244552	0.038714292
cg10277864	-0.922070052	3.420270419	-4.327186365	0.000244576	0.038714292
cg02898904	-0.615890601	2.648755347	-4.326976627	0.000244704	0.038714292
cg21306949	-0.77503335	-1.613200375	-4.326813111	0.000244804	0.038714292
cg08758916	-0.864459113	-5.117455695	-4.326762437	0.000244835	0.038714292
cg10793252	-0.521860349	3.655231771	-4.326741824	0.000244848	0.038714292
cg08318371	-0.518044693	3.422454227	-4.326683126	0.000244884	0.038714292
cg15527872	-0.987375543	0.784210291	-4.326515934	0.000244987	0.038714292
cg11390255	-0.467032769	4.05874129	-4.326463908	0.000245019	0.038714292
cg26088614	-0.559085862	3.47960965	-4.326274709	0.000245135	0.038714292
cg05032281	-0.583002438	2.778436984	-4.32627236	0.000245136	0.038714292
cg18944274	-0.755393925	2.156113913	-4.32608064	0.000245254	0.038714292
cg21152922	-0.628882956	3.155934307	-4.326038346	0.00024528	0.038714292
cg19574297	-0.435900844	-0.65376581	-4.326010799	0.000245297	0.038714292
cg10338697	-0.508650609	-0.496588414	-4.326009496	0.000245298	0.038714292
cg06451918	-0.460821436	4.074028939	-4.325872146	0.000245382	0.038714292
cg18349405	-0.455206394	3.884429716	-4.325613041	0.000245541	0.038714292
cg11266511	-0.640695825	2.813511739	-4.325436363	0.00024565	0.038714292
cg14633276	-0.537177746	3.332211611	-4.325379887	0.000245685	0.038714292
cg18232597	-0.689232244	-1.023314109	-4.325356625	0.000245699	0.038714292
cg08959397	-0.655903142	3.244994722	-4.325332346	0.000245714	0.038714292
cg01144053	-0.447319896	-1.927873646	-4.325180449	0.000245807	0.038714292
cg16386961	-0.74206001	2.529089837	-4.325164095	0.000245818	0.038714292
cg09461293	-0.63846265	2.577095413	-4.325073753	0.000245873	0.038714292
cg00753353	-0.737584599	2.492660849	-4.324772021	0.000246059	0.038714292
cg14781826	-0.876605021	-0.011339719	-4.324711071	0.000246097	0.038714292
cg21864056	-0.522597264	2.839596027	-4.324696907	0.000246105	0.038714292
cg08311343	-0.764763568	-1.777587532	-4.324491035	0.000246232	0.038714292
cg21961096	-0.611329784	2.596195942	-4.324336454	0.000246328	0.038714292
cg17438633	-0.543535977	-0.179578669	-4.324207947	0.000246407	0.038714292
cg25834802	0.670422641	2.674101226	4.324153564	0.000246441	0.038714292
cg05046829	-0.471407361	3.960860239	-4.324117462	0.000246463	0.038714292
cg24245620	-0.446418378	0.680779562	-4.323908755	0.000246592	0.038714292
cg08579247	-0.445217876	3.840264545	-4.323719935	0.000246708	0.038714292
cg22302226	-0.552458042	3.550379038	-4.323512243	0.000246837	0.038714292
cg19293832	-0.726788008	3.207081	-4.323319913	0.000246956	0.038714292
cg04570934	-0.547349883	0.147707624	-4.323184389	0.000247039	0.038714292
cg25798632	-0.479113484	-0.644067616	-4.32318161	0.000247041	0.038714292
cg08392576	-0.708144241	3.166101372	-4.323156474	0.000247057	0.038714292
cg10854850	-0.707852967	1.690907102	-4.323145131	0.000247064	0.038714292
cg08831401	-0.536431409	-0.763034575	-4.323100757	0.000247091	0.038714292
cg16905489	-0.61128472	3.408691164	-4.323090933	0.000247097	0.038714292
cg22847494	-0.400508725	4.108048672	-4.323087065	0.0002471	0.038714292
cg02234734	-0.563187054	0.895340556	-4.322992708	0.000247158	0.038714292
cg23358661	-0.720262677	2.067923045	-4.322916411	0.000247205	0.038714292

cg09024920	-0.429288331	3.933423322	-4.322909681	0.00024721	0.038714292
cg08789253	-0.583078063	3.833772463	-4.322899595	0.000247216	0.038714292
cg11880551	-0.48134912	-1.842968368	-4.322788768	0.000247284	0.038714292
cg01757101	-0.467199337	3.8938683	-4.322781402	0.000247289	0.038714292
cg24237339	-0.389184821	3.899411103	-4.322569172	0.00024742	0.038714292
cg27269190	-0.551104347	3.468527164	-4.322475499	0.000247478	0.038714292
cg05353702	-0.550834378	3.293773494	-4.322468047	0.000247483	0.038714292
cg03180134	-0.474839473	-2.34132817	-4.322401095	0.000247525	0.038714292
cg00610603	-0.488371682	4.233783348	-4.322378547	0.000247539	0.038714292
cg22297750	-0.640352235	2.461623638	-4.322377395	0.000247539	0.038714292
cg05226279	-0.620304709	2.860408192	-4.322298477	0.000247588	0.038714292
cg25372522	-0.426881712	3.7775506	-4.322228616	0.000247632	0.038714292
cg04479217	-0.406320077	4.158239918	-4.322160654	0.000247674	0.038714292
cg06747919	-0.437598172	-0.477956184	-4.321931854	0.000247816	0.038714292
cg02914050	-0.546653276	3.290371349	-4.321927626	0.000247818	0.038714292
cg26495760	-0.83080814	2.438330607	-4.321680214	0.000247972	0.038714292
cg03017005	-0.672345215	2.372442269	-4.321420245	0.000248133	0.038714292
cg08047577	-0.544372772	3.050031378	-4.321265459	0.00024823	0.038714292
cg02808203	0.711112454	3.618662433	4.321196126	0.000248273	0.038714292
cg23947614	-0.387051215	3.722642503	-4.321130862	0.000248313	0.038714292
cg03938704	-0.619085969	2.768309335	-4.321075101	0.000248348	0.038714292
cg16065219	-0.533302433	3.217060694	-4.321054685	0.000248361	0.038714292
cg06065150	-0.689534695	2.372366547	-4.321046277	0.000248366	0.038714292
cg15134801	-0.522339273	-1.489940171	-4.321039107	0.00024837	0.038714292
cg05875516	-0.578881479	3.351361029	-4.320991913	0.0002484	0.038714292
cg08825448	-0.536280154	3.671638872	-4.320858561	0.000248483	0.038714292
cg23668450	-0.568537191	-0.400642097	-4.320853514	0.000248486	0.038714292
cg04082020	-0.431929025	3.476091806	-4.320812601	0.000248511	0.038714292
cg02722330	-0.510027688	3.31851824	-4.3207777	0.000248533	0.038714292
cg23251279	-0.632708453	3.67871843	-4.320774903	0.000248535	0.038714292
cg21091189	-0.655566668	3.803835543	-4.320662161	0.000248605	0.038714292
cg02026447	-0.623915544	3.309629693	-4.320585545	0.000248653	0.038714292
cg15026099	-0.54496985	3.837532571	-4.320448862	0.000248738	0.038714292
cg19044794	-0.356247249	4.406737008	-4.320356734	0.000248795	0.038714292
cg08524734	-0.565543164	2.870460942	-4.320320436	0.000248818	0.038714292
cg06745684	-0.553070588	-0.30196716	-4.320228164	0.000248875	0.038714292
cg07370087	-0.506084286	3.556815818	-4.319758496	0.000249168	0.038714292
cg20750619	-0.568348959	3.238903329	-4.319665432	0.000249226	0.038714292
cg18243542	-0.406863749	3.918895627	-4.319662959	0.000249228	0.038714292
cg04124295	-0.473438166	3.210330836	-4.31955711	0.000249294	0.038714292
cg10651121	-0.46001903	-1.969524893	-4.319523151	0.000249315	0.038714292
cg03354077	-0.491463248	3.769378509	-4.319359101	0.000249418	0.038714292
cg03596777	-0.621725515	-0.786935611	-4.319318063	0.000249443	0.038714292
cg02479278	0.526870383	2.783285868	4.319051538	0.00024961	0.038714292
cg12705627	-0.466667603	2.141596211	-4.319038365	0.000249618	0.038714292
cg05112200	-0.675528169	3.3511388	-4.318918103	0.000249693	0.038714292
cg27000059	-0.609619466	2.509154463	-4.318841584	0.000249741	0.038714292

cg25092881	-0.602254405	2.811031306	-4.318823902	0.000249752	0.038714292
cg06343183	-0.513710114	3.384010133	-4.318785043	0.000249777	0.038714292
cg06614336	-0.551902123	4.143815336	-4.318737741	0.000249806	0.038714292
cg21714849	-0.415938246	4.281395367	-4.318668416	0.00024985	0.038714292
cg06538278	-0.747880292	1.4627965	-4.318666655	0.000249851	0.038714292
cg06333233	-0.555691422	-1.010874619	-4.318591233	0.000249898	0.038714292
cg06257798	-0.449786148	-1.938771992	-4.318549724	0.000249924	0.038714292
cg26131576	-0.440187584	3.775752282	-4.318510601	0.000249948	0.038714292
cg03636082	-0.497250379	3.652987337	-4.318504973	0.000249952	0.038714292
cg21786027	-0.743831437	2.92940577	-4.318498086	0.000249956	0.038714292
cg27503740	-0.802970798	2.44591098	-4.318318561	0.000250069	0.038714292
cg25436344	-0.443202394	-0.631973157	-4.318228164	0.000250125	0.038714292
cg06051442	0.804678112	1.771620526	4.317954346	0.000250297	0.038714292
cg23774155	-0.806327023	2.642231245	-4.317772869	0.000250411	0.038714292
cg15547794	-0.500146313	3.554272806	-4.31762412	0.000250504	0.038714292
cg27154192	-0.560888373	1.282469548	-4.317614329	0.00025051	0.038714292
cg16850027	-0.499431681	-0.823641338	-4.317593694	0.000250523	0.038714292
cg06943491	-0.663016995	2.921109655	-4.317589493	0.000250526	0.038714292
cg12270947	-0.578431298	1.018714493	-4.317474914	0.000250598	0.038714292
cg12963332	-0.632969342	2.869501901	-4.317473772	0.000250598	0.038714292
cg15708127	-0.430971005	-0.035691589	-4.31746444	0.000250604	0.038714292
cg11401820	-0.457368923	0.541226375	-4.317407313	0.00025064	0.038714292
cg16696727	-0.617250592	-0.590479397	-4.317279423	0.00025072	0.038714292
cg15158754	-0.797289355	-1.185321127	-4.317236352	0.000250747	0.038714292
cg22226311	-0.405195869	-0.785033879	-4.31717578	0.000250785	0.038714292
cg11587926	-0.917610141	1.732050777	-4.31717534	0.000250786	0.038714292
cg23622616	-0.409172605	-0.190987532	-4.317158684	0.000250796	0.038714292
cg21205282	-0.462930345	-0.853027335	-4.317156595	0.000250797	0.038714292
cg07700972	-0.649025355	3.376302389	-4.317078284	0.000250847	0.038714292
cg02170784	-0.511455602	-1.224046838	-4.316907325	0.000250954	0.038714292
cg04511182	-0.599246122	1.709430358	-4.316838516	0.000250997	0.038714292
cg09426571	-0.414534157	-0.103174263	-4.316813021	0.000251013	0.038714292
cg23433405	-0.557356132	3.575025755	-4.316780705	0.000251034	0.038714292
cg01504485	-0.510792261	3.651646069	-4.316761804	0.000251046	0.038714292
cg01916664	-0.969626278	1.66929427	-4.316755846	0.000251049	0.038714292
cg21216618	-0.696781746	2.717876006	-4.316537464	0.000251187	0.038714292
cg17342243	-0.548885361	3.345706077	-4.316450274	0.000251242	0.038714292
cg13830791	-0.493320019	3.406963601	-4.316399766	0.000251273	0.038714292
cg13161456	-0.469271234	1.122428418	-4.316390468	0.000251279	0.038714292
cg23368410	-0.583714499	2.744965592	-4.316328509	0.000251318	0.038714292
cg27307298	-0.439906296	-1.077411568	-4.316289213	0.000251343	0.038714292
cg03384047	-0.491279166	-0.666446727	-4.316168425	0.000251419	0.038717587
cg07200421	1.321835839	2.795520067	4.316001416	0.000251524	0.038725367
cg06898502	-0.576533462	3.563827731	-4.315866013	0.000251609	0.038730084
cg11759378	-0.487396903	3.815580814	-4.315763392	0.000251674	0.038731624
cg25554530	-0.784592528	2.303770567	-4.315555296	0.000251805	0.038733312
cg21150503	-0.566749397	2.756735094	-4.315553543	0.000251806	0.038733312

cg12865904	-0.53744052	3.860504375	-4.31534799	0.000251936	0.038733312
cg13460439	-0.511374218	3.608891378	-4.315182521	0.000252041	0.038733312
cg19625237	-0.592489383	3.179533417	-4.315103665	0.00025209	0.038733312
cg06799952	-0.366501004	3.61933458	-4.314959331	0.000252181	0.038733312
cg04398282	-0.523331334	3.284509643	-4.314940759	0.000252193	0.038733312
cg17948986	-0.477433574	3.355157124	-4.314933336	0.000252198	0.038733312
cg26671966	-0.623766878	1.565434388	-4.314813718	0.000252273	0.038733312
cg19996420	-0.492318677	-0.942555455	-4.31476284	0.000252306	0.038733312
cg03275396	-0.491260879	4.318196569	-4.314700426	0.000252345	0.038733312
cg27449158	-0.483686644	3.349756378	-4.314672229	0.000252363	0.038733312
cg16952631	-0.494273822	3.225743716	-4.314619942	0.000252396	0.038733312
cg27315109	-0.431664938	3.923369759	-4.314388544	0.000252542	0.038747373
cg21861532	-0.552687089	0.40159243	-4.314134695	0.000252703	0.038763619
cg05955006	-0.552185066	-0.470696518	-4.313708757	0.000252973	0.038789457
cg10456769	-0.651624155	3.411242587	-4.31369577	0.000252981	0.038789457
cg07229001	-0.574135487	-0.981349947	-4.31344683	0.000253139	0.038797529
cg07776130	-0.623787658	2.744224408	-4.313421162	0.000253155	0.038797529
cg08992471	-0.435874976	-0.938165418	-4.31329743	0.000253233	0.038797529
cg13133297	-0.961713083	2.07110109	-4.313212537	0.000253287	0.038797529
cg11148483	-0.395414082	4.21512015	-4.313118373	0.000253347	0.038797529
cg21679730	-0.398272467	-0.460655627	-4.313029674	0.000253403	0.038797529
cg12469164	-0.462913148	-0.733414262	-4.313008182	0.000253417	0.038797529
cg06928134	-0.434012769	3.155865617	-4.312844637	0.000253521	0.038805038
cg10166915	-0.598071141	-0.856181062	-4.312654852	0.000253641	0.0388151
cg03450180	-0.411614585	-0.346805623	-4.312506894	0.000253735	0.0388211
cg05040360	-0.59758009	3.439980394	-4.312363153	0.000253827	0.038826692
cg24859528	-0.570438501	4.182685719	-4.311923137	0.000254106	0.038843157
cg16595042	-0.525690514	0.164966774	-4.31191606	0.000254111	0.038843157
cg16508903	-0.581264269	0.843401199	-4.311861989	0.000254145	0.038843157
cg14852923	-0.383479301	-1.737153729	-4.311725222	0.000254232	0.038843157
cg20772995	-0.556754905	4.443073055	-4.311704671	0.000254245	0.038843157
cg15768810	-0.488537924	3.349535114	-4.311676891	0.000254263	0.038843157
cg14665413	-0.678752283	-1.855330552	-4.31130282	0.000254502	0.03886483
cg26950369	-0.691438444	1.793102737	-4.3112223	0.000254553	0.03886483
cg10415188	-0.712748214	2.681612624	-4.310856944	0.000254786	0.03886483
cg13838057	-0.469920059	0.784795413	-4.310853494	0.000254788	0.03886483
cg09045787	-0.510545825	3.414935272	-4.310681166	0.000254898	0.03886483
cg07841059	-0.457685858	-0.003738628	-4.310642375	0.000254923	0.03886483
cg13910020	-0.673383186	-2.040874539	-4.310617988	0.000254938	0.03886483
cg20638113	0.882912364	-4.609154049	4.310541117	0.000254987	0.03886483
cg16490805	3.718253341	1.883768965	4.310485699	0.000255023	0.03886483
cg02171024	-0.462123666	3.299299221	-4.310362156	0.000255102	0.03886483
cg19249097	-0.598003863	-0.202047153	-4.310252454	0.000255172	0.03886483
cg15832727	-0.685783645	1.607846776	-4.310227394	0.000255188	0.03886483
cg21093945	-0.594458972	-0.851358603	-4.310089271	0.000255276	0.03886483
cg07619967	-0.537999337	3.852916481	-4.309987156	0.000255341	0.03886483
cg14818279	-0.637070073	-0.342007383	-4.309911862	0.00025539	0.03886483

cg05057352	-0.584179191	-1.198445992	-4.309870645	0.000255416	0.03886483
cg03664527	-0.40602025	0.048104331	-4.309852991	0.000255427	0.03886483
cg09426333	-0.884484184	2.138474426	-4.309835468	0.000255439	0.03886483
cg24203155	-0.471778065	4.475809733	-4.309821469	0.000255447	0.03886483
cg03843572	-0.705307088	3.501301333	-4.309458685	0.00025568	0.038869538
cg13080042	-0.565092211	2.468902691	-4.309351846	0.000255748	0.038869538
cg21717810	-0.443388256	4.039707363	-4.309279103	0.000255795	0.038869538
cg03494790	-0.399044815	-0.964954202	-4.309245251	0.000255816	0.038869538
cg14957855	-0.923054505	2.931504392	-4.309233549	0.000255824	0.038869538
cg06461903	-0.795258169	2.394215225	-4.308838762	0.000256077	0.038869538
cg00968638	-0.575671946	-1.53708108	-4.308819837	0.000256089	0.038869538
cg24764114	-0.565186999	3.478566008	-4.308692289	0.000256171	0.038869538
cg00473929	-0.470586195	3.081551503	-4.308608025	0.000256225	0.038869538
cg21346680	-0.389734526	4.118110473	-4.30859331	0.000256234	0.038869538
cg24885246	-0.782722954	-0.709697222	-4.308576916	0.000256245	0.038869538
cg09547820	-0.51086077	0.484400953	-4.308557127	0.000256258	0.038869538
cg22261158	-0.595717051	3.450236493	-4.30850228	0.000256293	0.038869538
cg18156135	-0.50044029	-2.36094467	-4.308475778	0.00025631	0.038869538
cg24663170	-0.584404485	2.742633373	-4.308434009	0.000256337	0.038869538
cg11126904	-0.588094915	3.733009033	-4.308403332	0.000256356	0.038869538
cg06210576	-0.740143285	2.296427591	-4.308223048	0.000256472	0.038878771
cg00009737	0.484043107	2.583374277	4.307959198	0.000256642	0.038890328
cg16544737	-0.477703646	3.527991482	-4.307933517	0.000256658	0.038890328
cg22455206	-0.461990163	3.499301502	-4.307739913	0.000256783	0.038897963
cg19357431	-0.756115979	3.089887546	-4.307684348	0.000256818	0.038897963
cg00220654	0.549535881	1.856597526	4.307129308	0.000257176	0.038909994
cg21470594	-0.415321763	4.217924919	-4.30699541	0.000257262	0.038909994
cg02060732	-0.40782094	-0.383273338	-4.306975524	0.000257275	0.038909994
cg03808580	-0.582672643	2.832576958	-4.306969772	0.000257278	0.038909994
cg12482501	-0.570487831	2.569942056	-4.306958778	0.000257285	0.038909994
cg14557807	-0.731062833	1.567659965	-4.306899696	0.000257324	0.038909994
cg01939741	-0.615653038	2.901462513	-4.306721707	0.000257438	0.038909994
cg19319417	-0.590841708	3.94741356	-4.306600695	0.000257516	0.038909994
cg00794673	-0.580454538	-1.882801483	-4.306387712	0.000257654	0.038909994
cg09976669	0.494302428	2.047137555	4.306293429	0.000257715	0.038909994
cg09860921	-0.46584871	-1.184317741	-4.306208839	0.000257769	0.038909994
cg23327011	-0.530429056	0.37433354	-4.306203681	0.000257772	0.038909994
cg22331840	-0.529513321	3.681542514	-4.306029026	0.000257885	0.038909994
cg02268370	-0.489831033	3.671157071	-4.305997906	0.000257905	0.038909994
cg00399162	-0.529206546	3.527269803	-4.305826285	0.000258016	0.038909994
cg02924628	-0.482339909	4.108966457	-4.305798694	0.000258034	0.038909994
cg07507115	0.487164802	1.453186918	4.305756439	0.000258061	0.038909994
cg16325280	0.597877852	-4.377340444	4.305694197	0.000258102	0.038909994
cg12589350	-0.520306682	3.503018586	-4.305563783	0.000258186	0.038909994
cg13826364	-0.470114415	4.178128353	-4.305506782	0.000258223	0.038909994
cg02629470	-0.376862334	1.233483978	-4.305399069	0.000258292	0.038909994
cg16599804	-0.408830232	3.852900388	-4.305318819	0.000258344	0.038909994

cg07524685	-0.531642778	2.895542029	-4.305284071	0.000258367	0.038909994
cg16735361	-0.423637403	0.157157001	-4.305198975	0.000258422	0.038909994
cg01844755	-0.517405088	3.430042995	-4.304940109	0.00025859	0.038909994
cg26485613	-0.426121927	3.745637434	-4.304919472	0.000258603	0.038909994
cg22250332	-0.611267852	3.071122739	-4.304915693	0.000258605	0.038909994
cg09418268	-0.660768317	2.799784706	-4.304897686	0.000258617	0.038909994
cg06773488	-0.486907165	-1.60365974	-4.304892905	0.00025862	0.038909994
cg00367584	-0.647675313	3.590506319	-4.30486428	0.000258639	0.038909994
cg19690879	-0.549373229	0.568149274	-4.304839782	0.000258654	0.038909994
cg00322919	-0.519776988	3.906473936	-4.304838213	0.000258656	0.038909994
cg02861124	-0.582543909	3.183145255	-4.304344502	0.000258976	0.038949862
cg18808765	-0.694529973	1.865708554	-4.304178095	0.000259083	0.038957827
cg12547959	-0.426387353	3.600518007	-4.303939938	0.000259238	0.038972797
cg25052216	-0.675498638	3.26952971	-4.303502339	0.000259522	0.038998813
cg17446583	-0.854191792	1.570781828	-4.303458705	0.000259551	0.038998813
cg13508208	-0.656989758	2.983221267	-4.303409953	0.000259582	0.038998813
cg20337297	-0.549592729	-0.423634453	-4.303334652	0.000259631	0.038998813
cg16065434	-0.546368775	3.59546983	-4.303050593	0.000259816	0.039008012
cg26272723	-0.675335102	3.543182511	-4.303025128	0.000259833	0.039008012
cg24829450	-0.413914313	4.273242631	-4.302878165	0.000259928	0.039008012
cg13691567	-0.575534973	0.156668058	-4.302841297	0.000259952	0.039008012
cg17943175	-0.395966362	-2.136555242	-4.302722706	0.00026003	0.039008012
cg22463117	-0.621849118	3.643194577	-4.302574925	0.000260126	0.039008012
cg21763827	-0.460502657	3.743537921	-4.302531579	0.000260154	0.039008012
cg09065396	-0.737118951	2.294576492	-4.30246792	0.000260195	0.039008012
cg10617396	-0.412520321	2.992961063	-4.302426134	0.000260223	0.039008012
cg11619434	-0.477739089	3.699172663	-4.302309114	0.000260299	0.039008012
cg09121243	-0.498039653	3.672701196	-4.302130215	0.000260416	0.039008012
cg24479776	-0.599092319	1.181564542	-4.302010608	0.000260494	0.039008012
cg09577739	-0.579699424	3.618826173	-4.301890264	0.000260572	0.039008012
cg13789435	-0.484344828	3.771476761	-4.301819883	0.000260618	0.039008012
cg09273683	-0.371872692	-0.57148711	-4.301792483	0.000260636	0.039008012
cg04741379	-0.470936592	3.502465829	-4.301773311	0.000260648	0.039008012
cg14573769	-0.743117743	2.59001132	-4.301690852	0.000260702	0.039008012
cg01009386	-0.449575939	3.876870603	-4.3016298	0.000260742	0.039008012
cg11069446	-0.482735069	3.540995903	-4.301607474	0.000260757	0.039008012
cg25748057	-0.481969926	3.855610161	-4.301461531	0.000260852	0.039008012
cg23729005	-0.712372501	-1.564479525	-4.301449013	0.00026086	0.039008012
cg21294557	-0.530263084	3.734563794	-4.301382064	0.000260904	0.039008012
cg08466143	-0.742828565	2.7680883	-4.301007655	0.000261149	0.039036366
cg09405265	-0.432910829	-0.650971876	-4.300803794	0.000261282	0.03904806
cg08419350	-0.821582651	-0.976840216	-4.30062321	0.0002614	0.039050533
cg25924408	-0.514172675	0.870991917	-4.300610061	0.000261409	0.039050533
cg21893059	-0.861312508	1.571340145	-4.300326638	0.000261595	0.039070021
cg04800041	-0.514167771	3.354550391	-4.300168954	0.000261698	0.039077213
cg03308120	-0.557838799	3.013380297	-4.300066743	0.000261765	0.03907898
cg18547845	-0.534576266	3.961324796	-4.299830994	0.000261919	0.039085632

cg02265379	-0.485824234	-1.118395733	-4.299830498	0.00026192	0.039085632
cg14772660	-1.03895054	2.176608942	-4.299738559	0.00026198	0.039086399
cg12967384	-0.456259298	0.345187347	-4.299468792	0.000262157	0.039090251
cg20084434	-0.620721302	3.371652047	-4.299326755	0.00026225	0.039090251
cg19277926	-0.465918324	3.741265392	-4.299300679	0.000262268	0.039090251
cg13346355	-0.528217501	-0.072288776	-4.299262455	0.000262293	0.039090251
cg04057956	-0.553619416	-1.125843932	-4.299156457	0.000262362	0.039090251
cg20230784	-0.575543436	3.814664681	-4.299139878	0.000262373	0.039090251
cg09621610	-0.463991377	1.39064788	-4.299110925	0.000262392	0.039090251
cg21555240	-0.645945726	3.109742291	-4.298966608	0.000262487	0.039093038
cg26077761	-0.604732181	3.68038318	-4.298914537	0.000262521	0.039093038
cg25609327	-0.44340548	3.1801217	-4.298697917	0.000262664	0.039103992
cg26171924	-0.440291592	3.601391879	-4.298634814	0.000262705	0.039103992
cg25279351	-0.466517073	3.281871893	-4.298389471	0.000262867	0.039119805
cg18480726	-0.498626788	3.429364143	-4.298280485	0.000262938	0.039122265
cg02959688	-0.569988121	3.972783659	-4.29804862	0.000263091	0.039130665
cg14494808	-0.586343755	3.280284451	-4.297996959	0.000263125	0.039130665
cg04987833	-0.466827514	4.197716848	-4.297943212	0.000263161	0.039130665
cg17580261	-0.425881564	3.686961634	-4.29770723	0.000263316	0.03914558
cg15713625	-0.409880421	-1.0652608	-4.297571175	0.000263406	0.039150703
cg10017597	0.739009547	3.729084016	4.297481919	0.000263465	0.039151241
cg14862228	-0.64142738	3.226049566	-4.297350975	0.000263551	0.039152759
cg08616431	-0.402592632	-1.293463949	-4.297298934	0.000263586	0.039152759
cg17723924	-0.458973352	-0.776795805	-4.296993789	0.000263787	0.039172499
cg16404064	-0.779492626	-1.795464884	-4.296877379	0.000263864	0.039172499
cg07968094	-0.711850734	1.454389176	-4.296846542	0.000263884	0.039172499
cg16887335	-0.646790468	2.680761497	-4.296678644	0.000263995	0.039180762
cg13546240	-0.394496092	-0.914876942	-4.296412935	0.000264171	0.039185246
cg08077392	-0.528096103	3.313058011	-4.296323104	0.00026423	0.039185246
cg07323991	-0.509851421	3.643234164	-4.296317791	0.000264234	0.039185246
cg08344146	-0.649351871	2.606434334	-4.296298438	0.000264247	0.039185246
cg20126681	-0.499615247	3.372821283	-4.296018515	0.000264432	0.039204517
cg14611218	-0.532110058	0.319521106	-4.295544136	0.000264746	0.039220311
cg01282921	-0.47228016	3.765010688	-4.295311315	0.000264901	0.039220311
cg02642924	-0.532237924	3.468071132	-4.29527794	0.000264923	0.039220311
cg00394901	-0.624117274	0.15067363	-4.295272053	0.000264927	0.039220311
cg18247764	-1.165409312	2.424963455	-4.295108267	0.000265036	0.039220311
cg24191571	-0.754902265	2.293605428	-4.295063949	0.000265065	0.039220311
cg00289249	0.331313268	3.020328227	4.294914943	0.000265164	0.039220311
cg22294319	-0.605279105	2.510159991	-4.294892186	0.000265179	0.039220311
cg19603202	-0.41557155	0.21541998	-4.294774776	0.000265257	0.039220311
cg17543123	-0.616099947	-2.829735777	-4.294579545	0.000265387	0.039220311
cg05412784	-0.639067077	3.400426132	-4.294441693	0.000265478	0.039220311
cg27070962	-0.620070991	0.030467451	-4.294370936	0.000265525	0.039220311
cg16513334	-0.86173099	1.063522971	-4.294331723	0.000265551	0.039220311
cg20148068	-0.759470666	2.366243534	-4.29432925	0.000265553	0.039220311
cg03277161	-0.361677088	4.34373564	-4.294294779	0.000265576	0.039220311

cg11792522	-0.684431096	2.878125891	-4.294180673	0.000265652	0.039220311
cg16040017	-0.425078817	3.843167743	-4.294161816	0.000265664	0.039220311
cg07472795	0.532685951	2.452628122	4.294060754	0.000265732	0.039220311
cg11725435	-0.512223591	3.507212776	-4.293965161	0.000265795	0.039220311
cg03363829	-0.373512842	4.095496206	-4.293840492	0.000265878	0.039220311
cg26051028	-0.419920598	3.202429482	-4.293824237	0.000265889	0.039220311
cg26481783	-0.53101653	3.555276251	-4.293784992	0.000265915	0.039220311
cg19288676	-0.504652226	-1.533563721	-4.293740925	0.000265945	0.039220311
cg10879114	-0.456456015	3.572259069	-4.293685576	0.000265981	0.039220311
cg05890373	-0.508286611	4.173385351	-4.293681664	0.000265984	0.039220311
cg16345171	0.631452985	1.800086765	4.293662442	0.000265997	0.039220311
cg13130493	-0.502146198	3.444038535	-4.293607474	0.000266033	0.039220311
cg07502874	-0.406686136	0.347542812	-4.293302356	0.000266237	0.039227213
cg06802774	-0.499531607	-0.283804731	-4.293209006	0.000266299	0.039227213
cg26109889	-0.472860831	0.119319122	-4.293147703	0.00026634	0.039227213
cg15380511	-0.604999979	-1.224278265	-4.293127407	0.000266353	0.039227213
cg14184180	-0.507356674	2.610690252	-4.293084511	0.000266382	0.039227213
cg04870555	-0.504306961	3.850211161	-4.292931949	0.000266484	0.039227213
cg24964793	-0.684108638	3.071727353	-4.292842966	0.000266543	0.039227213
cg09971846	-0.625173937	3.205744221	-4.292780231	0.000266585	0.039227213
cg11612727	-0.577622453	-2.197258776	-4.292665535	0.000266662	0.039227213
cg21192716	-0.862373642	1.260594917	-4.292575814	0.000266722	0.039227213
cg27325965	0.756246237	-2.770412691	4.292267913	0.000266927	0.039227213
cg08822511	-0.689766429	3.327023275	-4.292130841	0.000267019	0.039227213
cg22433802	-0.486938343	-0.792211052	-4.292115629	0.000267029	0.039227213
cg10297722	-0.580071301	2.688877406	-4.29210663	0.000267035	0.039227213
cg05280501	-0.544242457	-2.100944645	-4.292082927	0.000267051	0.039227213
cg25068967	-0.480163582	3.564266936	-4.29202822	0.000267088	0.039227213
cg11460208	-0.462904001	3.981550707	-4.291910842	0.000267166	0.039227213
cg02605874	-0.669891872	3.052051142	-4.291769453	0.000267261	0.039227213
cg07097502	-0.693575306	2.539521804	-4.291757898	0.000267269	0.039227213
cg24032872	-0.433928574	3.906444864	-4.291726311	0.00026729	0.039227213
cg01035644	-0.484329306	3.782956899	-4.291716305	0.000267296	0.039227213
cg05880770	-0.40426922	3.165567972	-4.291532487	0.000267419	0.039227213
cg05235126	-0.398277746	3.613009376	-4.291403144	0.000267506	0.039227213
cg14513264	-0.576178049	3.815007088	-4.291402523	0.000267506	0.039227213
cg03526756	-0.542115634	4.179408668	-4.291388622	0.000267516	0.039227213
cg01210167	-0.352077289	3.799026691	-4.291382221	0.00026752	0.039227213
cg08885391	-0.485577806	4.260681816	-4.291251673	0.000267608	0.039231378
cg25681629	-0.629762867	2.835928993	-4.291043826	0.000267747	0.039231378
cg10031303	-0.675889497	2.820138592	-4.29099305	0.000267781	0.039231378
cg09514197	-0.558127048	0.198013788	-4.290956669	0.000267805	0.039231378
cg14183760	-0.599835342	0.850705967	-4.290859672	0.00026787	0.039231378
cg18393905	-0.490612046	3.67126851	-4.290824193	0.000267894	0.039231378
cg04837020	-0.516834685	3.545693372	-4.290732123	0.000267956	0.039231378
cg09712893	-0.669054517	2.681069925	-4.290598448	0.000268046	0.039231378
cg16021364	-0.734669617	2.42393748	-4.290534336	0.000268089	0.039231378

cg23328210	-0.662405221	0.057826579	-4.290271054	0.000268265	0.039231378
cg20963047	-0.408655618	-0.21182087	-4.29026543	0.000268269	0.039231378
cg24335366	-0.413573465	4.094565071	-4.290243603	0.000268284	0.039231378
cg27270264	-0.605076339	-1.0717852	-4.290206461	0.000268309	0.039231378
cg19789315	-0.510086136	3.762162375	-4.290184229	0.000268324	0.039231378
cg04800883	-0.590910516	3.7614839	-4.290013172	0.000268439	0.039240087
cg18641057	-0.366258845	4.016202042	-4.289822062	0.000268567	0.039250771
cg22247531	-0.66707844	2.384368626	-4.289566797	0.000268739	0.039258372
cg17348243	-0.629599912	3.156221668	-4.28951694	0.000268773	0.039258372
cg15354877	-0.499198256	2.723816259	-4.289497642	0.000268786	0.039258372
cg15326613	-0.512346794	3.544988664	-4.289395237	0.000268854	0.039260346
cg25579620	-0.470731636	3.452188472	-4.289074643	0.00026907	0.039264001
cg02732941	0.555867147	3.213166851	4.288955959	0.00026915	0.039264001
cg13849424	-0.601727012	2.91722884	-4.288939576	0.000269161	0.039264001
cg11911286	-0.581310981	0.624167558	-4.28888462	0.000269198	0.039264001
cg12251448	-0.556825275	3.398925742	-4.288865069	0.000269212	0.039264001
cg10273151	-0.642428027	-0.874017216	-4.288864432	0.000269212	0.039264001
cg23877008	-0.477922928	0.050783965	-4.288688688	0.000269331	0.039265028
cg02885095	-0.491224962	3.491342436	-4.288651114	0.000269356	0.039265028
cg20778669	-0.47904287	3.956329107	-4.288477699	0.000269473	0.039265028
cg13640414	0.434028182	-1.111751382	4.288477049	0.000269473	0.039265028
cg17323984	-0.702016949	2.245204342	-4.288423594	0.000269509	0.039265028
cg16276965	-0.672859574	3.11229369	-4.287906278	0.000269859	0.039265028
cg09820244	-0.66640203	-1.438330565	-4.28790093	0.000269862	0.039265028
cg07396182	-0.622848564	3.400192976	-4.287863449	0.000269888	0.039265028
cg11303667	-0.393119278	4.302426088	-4.287799769	0.000269931	0.039265028
cg27532187	-0.971252142	-3.81984106	-4.287798716	0.000269931	0.039265028
cg25535544	-0.443596542	3.451906289	-4.287782733	0.000269942	0.039265028
cg19078878	1.217295432	2.654499176	4.287724759	0.000269981	0.039265028
cg17325500	-0.390151999	3.8694145	-4.28763221	0.000270044	0.039265028
cg26828599	-0.771994166	-0.862033662	-4.287586112	0.000270075	0.039265028
cg16262479	-0.513362836	3.647661276	-4.287507821	0.000270128	0.039265028
cg08398073	-0.542913976	3.28151511	-4.287467707	0.000270155	0.039265028
cg10724315	-0.415354038	4.02666728	-4.287407651	0.000270196	0.039265028
cg00110223	-0.421495931	3.569648008	-4.287265007	0.000270292	0.039265028
cg06105925	-1.798522874	-4.39067244	-4.287178988	0.000270351	0.039265028
cg25576682	-0.600964585	3.421942099	-4.28716686	0.000270359	0.039265028
cg20871205	-0.467960711	3.683670173	-4.287002848	0.00027047	0.039265028
cg01330075	-0.506049868	0.73324104	-4.286926656	0.000270522	0.039265028
cg25143975	-0.573192565	2.767113479	-4.286823981	0.000270591	0.039265028
cg03704645	-0.512052454	3.80476336	-4.286732673	0.000270653	0.039265028
cg19258556	-0.490880693	3.357685429	-4.286590362	0.000270749	0.039265028
cg24555935	-0.390828468	2.463024765	-4.286507405	0.000270806	0.039265028
cg24901960	-0.70639959	3.174144879	-4.286350955	0.000270912	0.039265028
cg05484606	-0.502014606	-0.045601254	-4.286272209	0.000270965	0.039265028
cg03661929	-0.542956885	0.59246664	-4.286087981	0.00027109	0.039265028
cg11426922	-0.461940718	4.054170831	-4.286031422	0.000271129	0.039265028

cg20777082	-0.565817203	3.029916837	-4.286025725	0.000271133	0.039265028
cg12372706	-0.513643304	3.693412083	-4.285975753	0.000271166	0.039265028
cg05972059	-0.49443447	-0.64198067	-4.285905879	0.000271214	0.039265028
cg21822490	-0.496872348	3.368713012	-4.28582972	0.000271266	0.039265028
cg09651899	-0.483869762	3.994629176	-4.285816639	0.000271275	0.039265028
cg11017445	-0.576400317	3.81251227	-4.285767061	0.000271308	0.039265028
cg14051210	-0.772228969	1.551766416	-4.285726136	0.000271336	0.039265028
cg06104859	-0.523609151	4.051016392	-4.285278062	0.000271641	0.039265028
cg06469883	-0.419954212	-1.545372764	-4.285077431	0.000271777	0.039265028
cg24641427	-0.544537965	2.133068643	-4.284995049	0.000271833	0.039265028
cg26268896	-0.456263876	3.446730012	-4.28497507	0.000271847	0.039265028
cg10263494	-0.849826314	0.577253745	-4.284890142	0.000271905	0.039265028
cg08996726	-0.625230541	3.530374716	-4.284871808	0.000271917	0.039265028
cg25317724	-0.597976752	3.653100843	-4.284677755	0.000272049	0.039265028
cg16421264	-0.712116109	3.374394139	-4.284633608	0.000272079	0.039265028
cg19524316	-0.719645967	2.740159544	-4.284571707	0.000272121	0.039265028
cg09756121	-0.504052837	-0.706362107	-4.28450625	0.000272166	0.039265028
cg25338531	-0.560804794	3.272086242	-4.28438146	0.000272251	0.039265028
cg10898115	-0.47386381	0.037983292	-4.284333893	0.000272284	0.039265028
cg11041042	-0.857652401	2.415637632	-4.28429933	0.000272307	0.039265028
cg09191574	-0.504603386	3.207111732	-4.2842669	0.000272329	0.039265028
cg03310127	-0.4963111	4.087600886	-4.284145394	0.000272412	0.039265028
cg09018554	-0.537199967	3.571735296	-4.284077694	0.000272458	0.039265028
cg19149114	-0.547715199	2.497785567	-4.284013424	0.000272502	0.039265028
cg10626896	-0.512693923	0.333062655	-4.283957413	0.00027254	0.039265028
cg05577388	-0.456277809	2.970825467	-4.28386179	0.000272606	0.039265028
cg19255693	0.375931462	0.31378667	4.283759022	0.000272676	0.039265028
cg17831554	-0.554924654	3.327546455	-4.283641366	0.000272756	0.039265028
cg01639438	-0.585804478	3.450831359	-4.283521146	0.000272838	0.039265028
cg04619437	-0.477292145	4.232829576	-4.283490091	0.00027286	0.039265028
cg09204531	-0.524539417	3.366248134	-4.283468037	0.000272875	0.039265028
cg07122460	-0.592625308	2.412988999	-4.283443523	0.000272891	0.039265028
cg25226066	-0.983815449	-0.414694465	-4.283320326	0.000272976	0.039265028
cg26788659	-0.613186834	3.040101379	-4.283309017	0.000272983	0.039265028
cg00445142	-0.629417126	0.835257632	-4.283201475	0.000273057	0.039265028
cg21074911	-0.688160027	3.260221411	-4.283168963	0.000273079	0.039265028
cg15006396	-0.830351254	-3.088564938	-4.283157998	0.000273086	0.039265028
cg00143308	-0.55404982	3.114379397	-4.283017386	0.000273183	0.039265028
cg21573329	-0.538409757	-0.982682505	-4.282918161	0.000273251	0.039265028
cg04093208	-0.498483987	-1.241783373	-4.282902344	0.000273261	0.039265028
cg27583266	0.518185654	1.786308651	4.282861091	0.00027329	0.039265028
cg20707758	-0.487339339	3.638937468	-4.2828577	0.000273292	0.039265028
cg03353560	-0.605242	3.500095426	-4.282831085	0.00027331	0.039265028
cg02960488	-0.537146319	3.357922108	-4.282815407	0.000273321	0.039265028
cg09927477	-0.55740759	-0.085594557	-4.282599378	0.000273469	0.039278307
cg24015263	-0.671026039	2.075938672	-4.282424001	0.000273589	0.039287594
cg06462074	-0.616278706	3.68242634	-4.282124201	0.000273794	0.039301838

cg21856963	-0.518133682	0.214091415	-4.281899817	0.000273948	0.039301838
cg07218959	-0.468857274	3.797462943	-4.281871156	0.000273968	0.039301838
cg05403754	-0.484945533	4.072440666	-4.281768531	0.000274038	0.039301838
cg25281674	-0.880654897	-2.946486825	-4.281624625	0.000274137	0.039301838
cg11360546	-0.569063871	-0.8277008	-4.281494742	0.000274226	0.039301838
cg16585872	-0.4361111	3.128206468	-4.281386451	0.000274301	0.039301838
cg05702032	-0.413347818	3.502343217	-4.28136321	0.000274317	0.039301838
cg24993869	-0.655527823	2.073031272	-4.281296115	0.000274363	0.039301838
cg01834840	-0.472147285	3.987159199	-4.281291654	0.000274366	0.039301838
cg19617033	-0.789998925	2.518001845	-4.281278943	0.000274374	0.039301838
cg05117982	-0.478972522	-2.048768001	-4.281247252	0.000274396	0.039301838
cg24872610	-0.614402871	3.054354124	-4.281145918	0.000274466	0.039301838
cg05461734	-0.517904609	0.126108204	-4.281089027	0.000274505	0.039301838
cg16595745	-0.499477248	4.054310518	-4.2810667	0.00027452	0.039301838
cg10157234	-0.595411806	2.86689579	-4.280748791	0.000274739	0.039306412
cg02173484	0.414020304	1.352682462	4.280745302	0.000274741	0.039306412
cg03563307	0.583241657	1.992013154	4.280692391	0.000274778	0.039306412
cg18554492	-0.748306183	2.474598575	-4.280626852	0.000274823	0.039306412
cg25268697	-0.730186206	2.30981303	-4.280616863	0.00027483	0.039306412
cg02659355	0.572386288	2.66409402	4.280297734	0.000275049	0.039329892
cg21913376	-0.592201497	1.078329112	-4.280207753	0.000275111	0.039330816
cg05620787	-0.631300984	3.969188135	-4.279948297	0.00027529	0.039347689
cg21207205	-0.807618039	2.558293364	-4.279875285	0.00027534	0.039347689
cg05767035	-0.649671609	2.27204206	-4.279737364	0.000275436	0.039353341
cg26182842	-0.413333187	4.027765947	-4.279438754	0.000275642	0.039374834
cg15103180	-0.38303966	3.973004242	-4.278766345	0.000276106	0.039422746
cg15633396	-0.503002936	3.389424603	-4.278706649	0.000276147	0.039422746
cg18616409	-0.720193778	2.310577488	-4.27870596	0.000276148	0.039422746
cg26111107	-0.770287707	1.678883505	-4.278631072	0.0002762	0.039422746
cg16900632	-0.548165073	3.704899823	-4.278166896	0.000276521	0.03942553
cg08534512	-0.689054306	0.701392947	-4.278133336	0.000276544	0.03942553
cg10907257	-0.646868287	3.500483415	-4.278062804	0.000276593	0.03942553
cg01227628	-0.459971955	3.639581271	-4.278048202	0.000276603	0.03942553
cg21342557	-0.51578116	3.749003035	-4.277958134	0.000276665	0.03942553
cg16863382	-0.469839463	0.518397058	-4.277946206	0.000276674	0.03942553
cg25414709	-0.688292326	-0.087926012	-4.277923317	0.000276689	0.03942553
cg20716915	-0.44622179	4.423654367	-4.277908086	0.0002767	0.03942553
cg01999914	-0.409304275	3.869359402	-4.277824323	0.000276758	0.03942553
cg26924825	-0.431946419	-1.120463871	-4.277697225	0.000276846	0.03942553
cg12418173	-0.457563903	3.544088956	-4.277654533	0.000276876	0.03942553
cg09166546	-0.672744452	2.819345368	-4.27763838	0.000276887	0.03942553
cg22834409	-0.564206258	3.53085441	-4.277468782	0.000277005	0.039431802
cg20673929	-0.436764861	3.481952818	-4.277328789	0.000277102	0.039431802
cg01513576	-0.509305473	0.354481162	-4.277303195	0.000277119	0.039431802
cg03954045	-0.534339164	3.564617443	-4.277253863	0.000277154	0.039431802
cg26808738	-0.556375453	3.537109346	-4.277160178	0.000277219	0.039433133
cg09681910	-0.63609147	2.650095134	-4.27673761	0.000277512	0.039466771

cg09089006	-0.570701401	3.822615522	-4.276472362	0.000277697	0.039466771
cg22231516	-0.545733831	3.441604902	-4.276402991	0.000277745	0.039466771
cg26953086	-0.478278004	3.535207457	-4.276358779	0.000277776	0.039466771
cg02200717	-0.378912372	0.199607174	-4.276357118	0.000277777	0.039466771
cg05668926	-0.486873355	3.749954199	-4.276238029	0.00027786	0.039466771
cg23490425	-0.421776583	4.097354937	-4.276105442	0.000277952	0.039466771
cg18532070	-0.51939705	3.143305609	-4.275999083	0.000278026	0.039466771
cg06887454	-0.582197649	3.48082339	-4.275898823	0.000278096	0.039466771
cg23526504	-0.497667636	-1.166053918	-4.275888103	0.000278103	0.039466771
cg07849052	-0.764631835	2.637928688	-4.275861961	0.000278121	0.039466771
cg03505817	-0.473739357	-0.181615007	-4.275824871	0.000278147	0.039466771
cg06690989	-0.476643007	3.200595536	-4.275755818	0.000278195	0.039466771
cg16277405	-0.745333175	2.907857003	-4.275591537	0.00027831	0.039466771
cg19055178	-0.430556107	3.821306413	-4.275552102	0.000278337	0.039466771
cg00901816	-0.627179861	3.064591461	-4.275491861	0.000278379	0.039466771
cg05333636	-0.617218416	3.477650479	-4.275458588	0.000278402	0.039466771
cg13233074	-0.513962209	3.236748878	-4.275239165	0.000278555	0.039480559
cg14375632	-0.423546921	-0.157212491	-4.274783585	0.000278873	0.039515871
cg07041795	-0.591328705	3.644386139	-4.274722322	0.000278916	0.039515871
cg20443766	-0.483126168	-0.262903385	-4.274484863	0.000279082	0.039531465
cg17672752	-0.456537534	-1.152712415	-4.274320057	0.000279197	0.039539133
cg06757178	-0.64212303	-0.498227404	-4.274201979	0.00027928	0.039539133
cg17548578	-0.668159388	2.483338389	-4.274047618	0.000279388	0.039539133
cg17821079	0.595890015	-4.183036387	4.274000415	0.000279421	0.039539133
cg00783291	0.676384068	2.16083525	4.273918121	0.000279478	0.039539133
cg10773540	-0.765925482	2.386336247	-4.273912517	0.000279482	0.039539133
cg09357934	-0.419991343	-1.503892361	-4.273848756	0.000279527	0.039539133
cg13444151	-0.535858089	0.512308565	-4.273684572	0.000279642	0.039547493
cg03721403	-0.602847498	3.404006541	-4.273240148	0.000279953	0.039583621
cg22895198	-0.574337431	3.396602152	-4.273034297	0.000280097	0.039596125
cg10934068	-0.583498923	3.240313738	-4.272743785	0.000280301	0.03961005
cg15819914	-0.737901824	-2.440681791	-4.272734483	0.000280308	0.03961005
cg21550257	-0.565768738	3.348435204	-4.272579242	0.000280417	0.039617546
cg10368655	-0.44456895	4.249567451	-4.272278517	0.000280628	0.039632055
cg21162961	-0.544106486	2.617120025	-4.272263395	0.000280638	0.039632055
cg18356198	-0.360404222	4.210474687	-4.272004039	0.000280821	0.039632055
cg19524229	-0.602513123	2.573236811	-4.271997743	0.000280825	0.039632055
cg10059756	-0.514039707	-2.500092934	-4.271961325	0.000280851	0.039632055
cg19835482	-0.636472829	1.785718986	-4.271946429	0.000280861	0.039632055
cg17025642	-0.507927763	3.331067557	-4.271875786	0.000280911	0.039632055
cg11714477	-0.461511406	3.539575519	-4.271627656	0.000281086	0.039645001
cg06635397	-0.505272361	3.376336826	-4.27152056	0.000281161	0.039645001
cg21477285	-0.409966717	-1.340011449	-4.271506779	0.000281171	0.039645001
cg06333542	-0.603836602	2.038070004	-4.271317009	0.000281304	0.039649149
cg13071438	-0.485972787	-0.914081925	-4.271165321	0.000281411	0.039649149
cg07041565	-0.464594436	3.730928361	-4.271088186	0.000281465	0.039649149
cg24246984	-0.549041196	3.288890169	-4.271082848	0.000281469	0.039649149

cg03967501	0.631893167	-4.550315295	4.271067691	0.00028148	0.039649149
cg24843263	-0.489644899	4.072719435	-4.270824117	0.000281652	0.03966459
cg11123981	-0.411728805	0.402350121	-4.270745927	0.000281707	0.03966459
cg24796645	-0.595867503	2.334217254	-4.270673992	0.000281757	0.03966459
cg20533754	-0.773333923	3.782084717	-4.270514883	0.00028187	0.039665171
cg24374636	-0.685719952	-1.961418998	-4.270509428	0.000281874	0.039665171
cg12303247	-0.51394789	1.619257323	-4.270305704	0.000282017	0.039665426
cg15380772	-0.538141666	-1.552496402	-4.270263757	0.000282047	0.039665426
cg15774976	-0.651666833	3.053306823	-4.27009174	0.000282169	0.039665426
cg15118846	-0.535021768	1.018875291	-4.269938221	0.000282277	0.039665426
cg13131370	-0.845523586	3.032776409	-4.269883307	0.000282316	0.039665426
cg23626039	-0.423998124	-1.502423518	-4.269875023	0.000282322	0.039665426
cg03996913	-0.434680246	4.344103501	-4.269840328	0.000282346	0.039665426
cg24783019	-0.756423374	1.953417847	-4.26983394	0.000282351	0.039665426
cg09579651	-0.542783886	3.233677776	-4.26979342	0.000282379	0.039665426
cg11792007	-0.506177473	3.425705623	-4.26947827	0.000282602	0.039688866
cg13910601	-0.548252819	3.679237781	-4.26936	0.000282686	0.039692751
cg27027723	-0.535107375	2.702658629	-4.269189852	0.000282806	0.039701794
cg19813207	-0.596540708	0.347736222	-4.269025455	0.000282923	0.03970907
cg22954513	-0.559127318	-0.292408113	-4.268958382	0.00028297	0.03970907
cg01228854	-0.498401692	0.739155146	-4.268835841	0.000283057	0.039713387
cg16699728	-0.451053388	-0.580862188	-4.268638381	0.000283197	0.039714387
cg19246549	-0.538244704	3.863569651	-4.268633192	0.000283201	0.039714387
cg02567932	-0.499034793	3.610937651	-4.268588533	0.000283232	0.039714387
cg05609780	-0.513912339	-1.061309945	-4.268275715	0.000283454	0.039722005
cg19532814	-0.519047676	3.532018142	-4.268216708	0.000283496	0.039722005
cg06943029	-0.61763607	-0.615325917	-4.267939802	0.000283693	0.039722005
cg07739303	-0.514842042	-0.193718936	-4.267915547	0.00028371	0.039722005
cg24833225	-0.541648519	0.338525855	-4.267799024	0.000283793	0.039722005
cg09931140	-0.422786209	3.526211205	-4.267791298	0.000283798	0.039722005
cg11363743	-0.659151985	2.71915122	-4.267781096	0.000283806	0.039722005
cg00462515	-0.470174831	3.706118829	-4.267769687	0.000283814	0.039722005
cg05281304	-0.559464321	-1.491413061	-4.267705119	0.00028386	0.039722005
cg14929320	-0.54263404	3.287943815	-4.26769751	0.000283865	0.039722005
cg03539051	0.361183765	1.606821406	4.267643209	0.000283904	0.039722005
cg19966146	-0.734929818	2.629471207	-4.267514161	0.000283995	0.039726994
cg05228338	-0.888740134	2.115505809	-4.267397357	0.000284078	0.039730598
cg13957432	-0.566322035	3.376666778	-4.267316918	0.000284136	0.039730598
cg17111849	-0.882630707	1.985592491	-4.267241382	0.000284189	0.039730598
cg04739880	-0.692259249	-1.593257408	-4.26714903	0.000284255	0.039730984
cg07285814	-0.489784692	3.389875663	-4.267018987	0.000284348	0.039730984
cg06083990	-0.513214909	-0.334261792	-4.26700109	0.00028436	0.039730984
cg08284985	-0.537979722	3.640642261	-4.266765528	0.000284528	0.039736008
cg18239484	-0.47629644	-0.187487801	-4.266757661	0.000284534	0.039736008
cg11059341	-0.603214688	3.695517488	-4.26657534	0.000284664	0.039736008
cg01340229	-0.681322561	3.329196233	-4.266529296	0.000284697	0.039736008
cg04559279	-0.594165092	3.128694934	-4.266491137	0.000284724	0.039736008

cg21346679	-0.466224528	3.246718158	-4.2663018	0.000284859	0.039736008
cg04114767	-0.482314662	3.640576075	-4.266291674	0.000284866	0.039736008
cg17871011	-0.456007448	-0.573415677	-4.266253454	0.000284893	0.039736008
cg10988894	-0.376664888	-0.390426729	-4.266080031	0.000285017	0.039736008
cg07501957	-0.661380229	2.565606859	-4.266045083	0.000285042	0.039736008
cg18685911	-0.397298511	3.851182733	-4.266029731	0.000285053	0.039736008
cg22688969	-0.650516513	3.255630079	-4.26592568	0.000285127	0.039736008
cg04745458	-0.529258196	3.661733012	-4.265914345	0.000285135	0.039736008
cg04465052	-0.520228534	3.657716002	-4.265839509	0.000285189	0.039736008
cg18340250	-0.818352248	1.649972346	-4.265643839	0.000285328	0.039736008
cg09229115	-0.77928414	1.774737067	-4.265512968	0.000285422	0.039736008
cg26385798	-0.689158143	2.518086774	-4.265416597	0.000285491	0.039736008
cg16935203	0.714678727	-3.06721835	4.265403084	0.0002855	0.039736008
cg25142260	-0.591265796	3.459568635	-4.265397793	0.000285504	0.039736008
cg06210915	-0.518088111	3.651672895	-4.265378063	0.000285518	0.039736008
cg16419361	-0.708238925	1.683978455	-4.265203012	0.000285643	0.039745618
cg03577017	-0.564845045	2.249262259	-4.264965785	0.000285813	0.03976142
cg07282184	-0.497922445	3.157376419	-4.26475723	0.000285962	0.039774375
cg03495053	-0.603932867	-0.516483633	-4.264602338	0.000286073	0.039781991
cg22540410	-0.491474754	3.571864887	-4.264362771	0.000286245	0.039798046
cg16024028	-0.406142534	4.265736446	-4.264220278	0.000286347	0.039803501
cg12099990	-0.583360022	3.560600266	-4.264093247	0.000286438	0.039803501
cg04424070	-0.547708215	4.206949079	-4.264072902	0.000286453	0.039803501
cg14873567	0.685977001	1.77418527	4.263679619	0.000286735	0.03981785
cg06026331	-0.738201396	2.759480198	-4.263676749	0.000286737	0.03981785
cg07277618	-0.567692461	2.747263953	-4.263586553	0.000286802	0.03981785
cg04609889	-0.580523861	0.060825097	-4.263490299	0.000286871	0.03981785
cg18409312	0.828244194	-2.870871078	4.263437625	0.000286909	0.03981785
cg07109430	-0.45775519	4.015926649	-4.263410597	0.000286928	0.03981785
cg07347148	-0.372431687	3.907601733	-4.263380867	0.000286949	0.03981785
cg21940640	-0.445359636	-1.6412238	-4.263162229	0.000287107	0.039821738
cg24337863	0.577639396	2.770663857	4.26314142	0.000287122	0.039821738
cg21251563	-0.661885439	-1.476076373	-4.263064013	0.000287177	0.039821738
cg18905886	-0.561707184	2.905690357	-4.26294145	0.000287265	0.039821738
cg10678718	-0.479200958	4.450069235	-4.262900872	0.000287294	0.039821738
cg11324910	-0.480221755	3.444018797	-4.262872684	0.000287315	0.039821738
cg07669814	-0.595907684	2.872997346	-4.262662501	0.000287466	0.039823425
cg17688440	-0.472301913	-0.832741254	-4.262578215	0.000287527	0.039823425
cg06550183	-0.510007456	-0.373449044	-4.262562677	0.000287538	0.039823425
cg23637093	-0.716287814	0.368229672	-4.262543277	0.000287552	0.039823425
cg23946319	-0.682084381	3.284169725	-4.262350129	0.000287691	0.039832753
cg08602276	-0.456079006	4.423029034	-4.262293571	0.000287732	0.039832753
cg10592563	-0.472043511	3.983737831	-4.262104983	0.000287868	0.039841751
cg16731104	-0.522844108	3.608865807	-4.262047268	0.000287909	0.039841751
cg05836902	-0.496377763	3.951843856	-4.26185893	0.000288045	0.039852755
cg07954054	-0.622155034	2.70436745	-4.261691695	0.000288166	0.039861659
cg05869631	0.660257825	1.910564439	4.261479591	0.000288319	0.039867586

cg17711217	-0.717359409	4.02860561	-4.261249682	0.000288485	0.039867586
cg11151995	-0.475700399	3.921785795	-4.26123974	0.000288492	0.039867586
cg18978680	-0.48993464	0.134507518	-4.261199991	0.00028852	0.039867586
cg04334564	-0.391491258	3.03196871	-4.261154363	0.000288553	0.039867586
cg24908618	-0.688357023	3.403195274	-4.261066771	0.000288617	0.039867586
cg26089892	-0.59688854	3.34142934	-4.261020341	0.00028865	0.039867586
cg17150074	-0.446477164	2.730602927	-4.261008788	0.000288659	0.039867586
cg07640187	-0.543538043	3.247904889	-4.260840706	0.00028878	0.039876591
cg15104906	-0.506599931	0.336384185	-4.2606228	0.000288938	0.039884387
cg26473189	-0.603214396	-0.273659495	-4.26046937	0.000289049	0.039884387
cg15238404	-0.408550761	4.12123	-4.260417122	0.000289086	0.039884387
cg11252331	-0.480057184	3.751349534	-4.260295565	0.000289174	0.039884387
cg04592197	0.605397238	3.057769741	4.260294633	0.000289175	0.039884387
cg07657456	-0.458807576	-0.483034456	-4.260260291	0.0002892	0.039884387
cg15538101	-0.562404815	-2.627433056	-4.260171389	0.000289264	0.039884387
cg07326692	-0.786029368	3.401892916	-4.260140199	0.000289287	0.039884387
cg26019654	-0.642008908	1.849642744	-4.259901519	0.00028946	0.03989138
cg26505319	-0.615507705	4.234939096	-4.259887183	0.00028947	0.03989138
cg18649438	-0.53337005	-2.289899722	-4.259837011	0.000289507	0.03989138
cg04763558	-0.52680172	-0.902180579	-4.259639628	0.00028965	0.039897958
cg17506734	-0.76470826	-0.443243477	-4.259615784	0.000289667	0.039897958
cg27255505	-0.551391339	3.751441869	-4.259400312	0.000289823	0.039911725
cg17314407	-0.868016693	2.787801402	-4.259125636	0.000290023	0.03992657
cg13041080	-0.466541571	3.576475492	-4.259096499	0.000290044	0.03992657
cg27500229	0.659343939	4.028850459	4.258970992	0.000290135	0.03992942
cg18003659	-0.498479596	0.337081611	-4.258764017	0.000290285	0.03992942
cg11917415	-0.713209068	2.869789662	-4.258672713	0.000290352	0.03992942
cg15458254	-0.504956647	3.42269872	-4.258602362	0.000290403	0.03992942
cg21548672	-0.539025248	3.609967864	-4.25855056	0.000290441	0.03992942
cg13845800	-0.462913941	-1.065164211	-4.25851566	0.000290466	0.03992942
cg15744368	-0.632340045	3.235996304	-4.258422915	0.000290533	0.03992942
cg00501869	-0.498139826	-1.365321096	-4.258367673	0.000290574	0.03992942
cg23291280	-0.617346672	-2.315536514	-4.258339734	0.000290594	0.03992942
cg04127075	-0.664245336	-1.3027786	-4.258292502	0.000290628	0.03992942
cg17623793	-0.356696927	-0.292076909	-4.258066646	0.000290793	0.039944256
cg24554777	-0.723076359	1.145353657	-4.257831158	0.000290964	0.039960063
cg11669124	-0.539808356	3.661485075	-4.257678684	0.000291075	0.03996757
cg02480396	-0.578590585	3.217647656	-4.257503212	0.000291203	0.039970521
cg11199066	0.859506494	1.412667653	4.257433362	0.000291254	0.039970521
cg19172949	-0.517697395	3.350433885	-4.257242615	0.000291393	0.039970521
cg00765852	-0.401199291	-0.299475917	-4.257195892	0.000291427	0.039970521
cg14913547	-0.39000376	3.333287864	-4.257030254	0.000291548	0.039970521
cg16536560	-0.637324808	2.938403777	-4.256991785	0.000291576	0.039970521
cg02994477	-0.631727997	3.651333001	-4.256901115	0.000291642	0.039970521
cg21974679	-0.479558899	-0.153953233	-4.256755431	0.000291749	0.039970521
cg06646724	-0.532308287	0.744620013	-4.256727033	0.000291769	0.039970521
cg01691328	-0.758731777	2.650358603	-4.25671947	0.000291775	0.039970521

cg00312217	-0.515342912	4.025185926	-4.256717172	0.000291777	0.039970521
cg27442871	-0.59332362	2.327019644	-4.256712489	0.00029178	0.039970521
cg21742071	-0.564711211	0.131408557	-4.256621072	0.000291847	0.039970521
cg08493904	-0.436056902	3.632459759	-4.256566664	0.000291887	0.039970521
cg10361345	-0.417216078	4.288715269	-4.256359078	0.000292038	0.039980976
cg20578356	-0.488649694	3.815159768	-4.256200584	0.000292154	0.039980976
cg26325286	-0.512404932	0.156075161	-4.256187459	0.000292164	0.039980976
cg20915760	-0.475791048	-1.293179476	-4.256153434	0.000292189	0.039980976
cg20520804	-0.679050807	-1.305232034	-4.25602068	0.000292286	0.039986541
cg15020502	0.5345893	1.458948899	4.255771909	0.000292468	0.040002501
cg01312999	-0.57085554	1.008879631	-4.25570702	0.000292515	0.040002501
cg01933946	0.639547316	-4.296633498	4.255496673	0.000292669	0.040002591
cg19220584	-0.508436613	-1.724191385	-4.255461373	0.000292695	0.040002591
cg05025239	-0.537727576	2.58049485	-4.255431305	0.000292717	0.040002591
cg21594043	-0.526929526	-2.289588268	-4.255307833	0.000292808	0.040002591
cg27010449	-0.675410884	1.242729319	-4.2552754	0.000292832	0.040002591
cg05315334	-0.705972261	-2.889416057	-4.255243695	0.000292855	0.040002591
cg16136487	-0.547822298	3.458743114	-4.25515838	0.000292917	0.040003421
cg02437493	-0.400755365	3.824065773	-4.255071887	0.000292981	0.040004372
cg26951340	-0.64941886	-1.312455832	-4.254973781	0.000293053	0.040006486
cg24523306	-0.728156871	1.980000413	-4.2548785	0.000293123	0.04000832
cg01446692	0.536533078	3.352569037	4.254574747	0.000293346	0.04002377
cg00472914	-0.570261589	2.97085748	-4.254536823	0.000293374	0.04002377
cg09254171	-0.447284364	-0.888641818	-4.254468664	0.000293424	0.04002377
cg16767895	-0.441249319	-0.279344749	-4.254292136	0.000293553	0.04002377
cg16585333	-0.505155141	-2.663452398	-4.254291985	0.000293553	0.04002377
cg20342255	-0.544375439	2.703926211	-4.254138274	0.000293666	0.04002377
cg10622643	-0.766076923	-2.177822364	-4.254023365	0.000293751	0.04002377
cg16149927	-0.692962567	0.171010945	-4.254013458	0.000293758	0.04002377
cg08140751	-0.446010291	-0.173548364	-4.253887161	0.000293851	0.04002377
cg13496421	-0.789205325	2.449891118	-4.253882925	0.000293854	0.04002377
cg24486867	-0.456563481	3.978275898	-4.253839027	0.000293887	0.04002377
cg18509719	-0.768694195	2.297328883	-4.2537331	0.000293964	0.04002377
cg12566844	-0.423292881	-0.13185943	-4.253724927	0.00029397	0.04002377
cg16350225	-0.398184243	2.75597963	-4.253439756	0.00029418	0.040044656
cg00931877	-0.503294128	3.914922234	-4.253101071	0.00029443	0.040069036
cg27467076	-0.619742711	1.378293743	-4.252945587	0.000294545	0.040069036
cg13546664	-0.598664427	2.853681094	-4.252928794	0.000294557	0.040069036
cg03876697	-0.395934812	0.712858132	-4.252876995	0.000294595	0.040069036
cg18780435	-0.503803794	3.543698314	-4.252813004	0.000294642	0.040069036
cg20054786	-0.401079546	3.720437901	-4.252547274	0.000294838	0.040078868
cg09746018	-0.440002987	3.268722302	-4.25250607	0.000294869	0.040078868
cg17406249	-0.35774417	3.809442975	-4.252484991	0.000294884	0.040078868
cg04832291	-0.715878997	-0.478624323	-4.252336346	0.000294994	0.040086094
cg27383418	-0.592717774	-1.58187685	-4.252186395	0.000295105	0.040088084
cg27038049	-0.672536402	1.992295369	-4.252163275	0.000295122	0.040088084
cg11619797	-0.568740992	2.812291588	-4.251957963	0.000295274	0.040101004

cg07823305	-0.61122133	2.70419913	-4.251771852	0.000295411	0.040102247
cg04012830	-0.458045594	-0.61530507	-4.251553135	0.000295573	0.040102247
cg20988852	-0.668395434	2.795452321	-4.251547451	0.000295577	0.040102247
cg23803253	-0.544910561	3.702547502	-4.251520444	0.000295597	0.040102247
cg03579446	-0.718179012	2.172838991	-4.251453349	0.000295647	0.040102247
cg05785081	-0.587145713	-1.601422249	-4.251282767	0.000295773	0.040102247
ch.3.2059622R	-0.841424175	-2.911929435	-4.25121797	0.000295821	0.040102247
cg21437591	-0.598874451	3.027861378	-4.25117448	0.000295853	0.040102247
cg19505184	-0.455261328	3.692845123	-4.251113511	0.000295898	0.040102247
cg20331691	-0.549534257	3.748284657	-4.251091963	0.000295914	0.040102247
cg04621022	-0.485360375	-0.847424342	-4.250998606	0.000295984	0.040102247
cg01424562	-0.945498118	-4.058041424	-4.250921108	0.000296041	0.040102247
cg02319043	0.671792087	-2.787457461	4.250898246	0.000296058	0.040102247
cg07969095	-0.689979801	3.068745377	-4.250802961	0.000296129	0.040102247
cg01645729	-0.514694261	-3.270113186	-4.250798428	0.000296132	0.040102247
cg01601949	-0.548222255	-1.035751365	-4.250408305	0.000296421	0.040122107
cg26620978	-0.615488413	0.378633065	-4.250385702	0.000296438	0.040122107
cg24945346	-0.376924944	3.835156271	-4.250293614	0.000296506	0.040122107
cg04015122	-0.910054573	2.561018104	-4.250286147	0.000296512	0.040122107
cg25787654	-0.523458712	3.71506027	-4.25021899	0.000296562	0.040122107
cg25837944	-0.547415225	-1.907483366	-4.249952005	0.00029676	0.040128439
cg01314658	-0.757592362	-0.247531829	-4.249895743	0.000296802	0.040128439
cg18432790	-0.361946311	-1.047230269	-4.249849838	0.000296836	0.040128439
cg18997075	-0.586953869	3.72950845	-4.249784787	0.000296884	0.040128439
cg14876009	-0.83043157	1.196011459	-4.249774655	0.000296892	0.040128439
cg03663880	-0.729773536	2.1120326	-4.249682917	0.00029696	0.040129998
cg02916102	-0.551786795	-4.283864912	-4.249523319	0.000297079	0.040138377
cg08472398	-1.25564033	-5.677262535	-4.249384707	0.000297182	0.04013864
cg25021279	-0.520952319	-0.944639787	-4.249368341	0.000297194	0.04013864
cg03087142	0.830994852	-4.652420379	4.248998919	0.000297469	0.040161377
cg24824917	-0.478373615	-2.550199534	-4.248989805	0.000297476	0.040161377
cg23098844	-0.4405366	-0.051840024	-4.248824551	0.000297599	0.040164109
cg02379352	-0.815691798	1.35630749	-4.248741649	0.000297661	0.040164109
cg21106462	-0.649798003	2.576759645	-4.248712629	0.000297682	0.040164109
cg21763793	-0.585058967	3.514985008	-4.248450007	0.000297878	0.040164109
cg13775490	-0.498149551	3.783259561	-4.248380682	0.00029793	0.040164109
cg05275603	-0.713938823	1.695133328	-4.248326134	0.00029797	0.040164109
cg04804796	-0.4696155	3.778039588	-4.248130605	0.000298116	0.040164109
cg00993127	-0.398237297	3.821287339	-4.248119713	0.000298124	0.040164109
cg18786018	-0.513191525	2.469687232	-4.248048294	0.000298178	0.040164109
cg21666996	-0.810402794	0.500533625	-4.247996725	0.000298216	0.040164109
cg03983544	-0.677763156	3.013576366	-4.247937762	0.00029826	0.040164109
cg20026466	-0.419217735	0.586431269	-4.24791938	0.000298274	0.040164109
cg17102674	-0.641674305	-0.096499824	-4.247854123	0.000298323	0.040164109
cg21035374	-0.59077594	-0.584715669	-4.247708152	0.000298432	0.040164109
cg17390675	-0.718497328	0.992847186	-4.247661024	0.000298467	0.040164109
cg08512167	-0.425581071	-1.958382814	-4.247590707	0.000298519	0.040164109

cg15189393	-0.454891158	3.679049981	-4.247500651	0.000298587	0.040164109
cg15500594	-0.653582055	3.516150173	-4.247445589	0.000298628	0.040164109
cg22365139	-0.618152554	1.792586438	-4.24743044	0.000298639	0.040164109
cg04721764	-0.574786052	3.71261824	-4.247307163	0.000298731	0.040164109
cg12813797	-0.825769744	0.636396993	-4.247254608	0.000298771	0.040164109
cg25927551	-0.585319404	3.243821453	-4.247246058	0.000298777	0.040164109
cg23673974	-0.464862556	-0.069588782	-4.24721532	0.0002988	0.040164109
cg08623971	-0.45749678	3.780200843	-4.246957984	0.000298993	0.040182365
cg05380205	-0.665494976	2.757128163	-4.246746877	0.000299151	0.040194537
cg09163652	-0.730985208	2.903574538	-4.246569298	0.000299284	0.040194537
cg25389269	-0.729900122	-3.929023574	-4.246520047	0.000299321	0.040194537
cg13647527	-0.527077504	2.035600112	-4.246188209	0.000299569	0.040194537
cg09504658	-0.431318213	4.456709318	-4.246063475	0.000299663	0.040194537
cg12017434	-0.467121927	0.647480344	-4.246056041	0.000299668	0.040194537
cg13867646	-0.594569571	1.820711323	-4.246007867	0.000299704	0.040194537
cg01764979	-0.629700543	-0.925035115	-4.24598426	0.000299722	0.040194537
cg02327789	-0.486648461	4.44007371	-4.245849492	0.000299823	0.040194537
cg10650299	-0.896243048	1.475173281	-4.245793193	0.000299866	0.040194537
cg21619340	-0.665376194	2.644718571	-4.245772388	0.000299881	0.040194537
cg19769877	-0.75323378	2.072999052	-4.245732467	0.000299911	0.040194537
cg05548325	-0.54008236	3.152009762	-4.245723158	0.000299918	0.040194537
cg00065419	-0.425026828	-1.409369264	-4.245655609	0.000299969	0.040194537
cg03220564	-0.592974953	0.423998292	-4.245641891	0.000299979	0.040194537
cg02700106	-0.631367809	-2.184285535	-4.245626164	0.000299991	0.040194537
cg12025832	-0.438164424	0.105200781	-4.245547653	0.00030005	0.040194834
cg13977600	-0.511219798	4.092268573	-4.245464902	0.000300112	0.04019556
cg08719515	-0.455242707	0.309255603	-4.245257544	0.000300268	0.040206128
cg22958881	-0.466374744	3.90193775	-4.245100746	0.000300386	0.040206128
cg05825121	-0.403193415	0.337769435	-4.245079482	0.000300402	0.040206128
cg17757514	-0.530291594	2.968132856	-4.245057855	0.000300418	0.040206128
cg08041408	-0.477437321	-0.136714109	-4.244856245	0.00030057	0.040218825
cg04776542	-0.478025759	4.248642923	-4.244753973	0.000300647	0.040219531
cg08518792	-0.516350473	2.729133629	-4.244591094	0.000300769	0.040219531
cg17121254	-0.66153275	2.806107354	-4.244587675	0.000300772	0.040219531
cg23766360	-0.414141532	-1.137568537	-4.244484651	0.000300849	0.040219531
cg26717322	-0.608339939	-0.731803144	-4.244472093	0.000300859	0.040219531
cg05397739	-0.334071617	4.32865741	-4.243935554	0.000301263	0.040260784
cg20883701	-0.661122557	2.63198377	-4.243856406	0.000301323	0.040260784
cg04700229	-0.723911227	2.703973014	-4.24383642	0.000301338	0.040260784
cg04813697	-0.548753615	0.237130106	-4.243758547	0.000301397	0.040261039
cg24440300	-0.566611041	3.171262754	-4.243589494	0.000301524	0.040266119
cg24110396	-0.507855862	0.026349126	-4.24351247	0.000301582	0.040266119
cg03622604	-0.476540562	3.590306173	-4.243482205	0.000301605	0.040266119
cg07395436	-0.656190957	3.271835996	-4.243316737	0.00030173	0.040271579
cg22950458	-0.583112598	3.944328771	-4.243252589	0.000301779	0.040271579
cg15790649	0.550969646	3.256554868	4.243202215	0.000301817	0.040271579
cg11070119	-0.489198112	3.894802464	-4.242880644	0.00030206	0.040292572

cg16570791	-0.573768526	3.021176901	-4.24284354	0.000302088	0.040292572
cg14224760	-0.717870208	2.627091141	-4.242747737	0.00030216	0.040294648
cg26931213	-0.431377746	3.604882182	-4.242575407	0.000302291	0.040297166
cg17867769	-0.428169181	3.998731365	-4.242544031	0.000302314	0.040297166
cg00475790	-0.588309959	4.061722532	-4.242497211	0.00030235	0.040297166
cg00460179	-0.560964788	2.940572472	-4.242356617	0.000302456	0.040301244
cg00537210	-0.550205284	-0.444758908	-4.242202106	0.000302573	0.040301244
cg08762628	-0.49833677	-0.545847064	-4.242170513	0.000302597	0.040301244
cg13085643	-0.431238969	3.443217497	-4.242130586	0.000302627	0.040301244
cg09987806	-0.494930708	3.53126044	-4.242081129	0.000302665	0.040301244
cg08846079	-0.459152131	-1.540382942	-4.24185172	0.000302839	0.040316814
cg24012574	-0.581116563	3.120930579	-4.241607736	0.000303024	0.04033134
cg07113870	-0.679440138	2.960373326	-4.241557661	0.000303062	0.04033134
cg18618195	-0.60900299	2.645210255	-4.241467624	0.00030313	0.040332854
cg23315282	-0.534902364	4.24824934	-4.241195086	0.000303337	0.04034524
cg00289254	-0.48111339	-1.125836962	-4.241194897	0.000303337	0.04034524
cg13416026	-0.498030746	2.981549831	-4.240954143	0.00030352	0.04034856
cg22602830	-0.577647106	-0.159297043	-4.240825032	0.000303618	0.04034856
cg23159236	-0.527915316	-0.848985565	-4.240818191	0.000303623	0.04034856
cg16317553	-0.490834994	0.685506574	-4.240804993	0.000303633	0.04034856
cg26442961	-0.45469629	3.747932881	-4.240767826	0.000303661	0.04034856
cg07920109	-0.692490142	3.123509152	-4.240655956	0.000303746	0.04034856
cg10737847	-0.504447443	3.798673935	-4.240637274	0.000303761	0.04034856
cg12809842	0.699382912	-4.392964205	4.240512077	0.000303856	0.04035364
cg05130541	-0.529279812	1.04021145	-4.240257791	0.000304049	0.040354227
cg23603305	-0.526997863	4.012233037	-4.240176234	0.000304111	0.040354227
cg02867407	-0.443611528	4.021179844	-4.240060439	0.0003042	0.040354227
cg00120332	-0.592982146	0.344971209	-4.240013926	0.000304235	0.040354227
cg25933594	-0.512570247	3.779157588	-4.23998054	0.00030426	0.040354227
ch.2.1685112F	-0.49655479	-2.381509215	-4.239954836	0.00030428	0.040354227
cg07181472	-0.562383421	3.372261694	-4.239944518	0.000304288	0.040354227
cg11557886	-0.560889584	3.076194468	-4.239907501	0.000304316	0.040354227
cg19255113	-0.631213636	-1.132776329	-4.239741635	0.000304442	0.040356388
cg15147120	-0.525135218	3.922161957	-4.239569995	0.000304573	0.040356388
cg12211471	-0.439346731	3.72213495	-4.239554115	0.000304585	0.040356388
cg07910075	-0.553315558	-0.865933817	-4.239551021	0.000304588	0.040356388
cg16770048	-0.505000938	0.329366499	-4.239503305	0.000304624	0.040356388
cg02450219	-0.389542584	-0.660292886	-4.239435575	0.000304676	0.040356388
cg04300159	-0.595936817	-0.856454187	-4.239356936	0.000304736	0.040356388
cg12741065	-0.767993267	-4.790699879	-4.238928419	0.000305063	0.040356388
cg04458562	-0.51212259	-0.881311454	-4.238909798	0.000305077	0.040356388
cg16975406	0.772183874	1.542823212	4.238888517	0.000305093	0.040356388
cg16929959	-0.585302664	2.244666899	-4.238779367	0.000305177	0.040356388
cg14168246	-0.642839023	1.042872501	-4.238660591	0.000305267	0.040356388
cg03204787	-0.58630729	0.519542877	-4.238655927	0.000305271	0.040356388
cg09802689	-0.757052034	2.2485078	-4.238600472	0.000305313	0.040356388
cg27618701	-0.724179515	1.891758251	-4.238392026	0.000305473	0.040356388

cg16542473	-0.46102921	3.359215295	-4.238372072	0.000305488	0.040356388
cg20808750	-0.68831365	2.561135164	-4.238300241	0.000305543	0.040356388
cg15472815	-0.513011116	3.651802257	-4.238297651	0.000305545	0.040356388
cg17001881	-0.535172119	0.355482148	-4.23825524	0.000305577	0.040356388
cg06672736	-0.629432185	1.761349224	-4.238248461	0.000305582	0.040356388
cg13466629	-0.451030566	3.211748525	-4.238171707	0.000305641	0.040356388
cg24570371	-0.48638731	-0.606921188	-4.23806898	0.00030572	0.040356388
cg14895968	-0.392682817	-0.195482834	-4.238044997	0.000305738	0.040356388
cg23586440	-0.476515962	0.627887425	-4.238034982	0.000305746	0.040356388
cg05903821	-0.486851249	3.591530575	-4.237948116	0.000305812	0.040356388
cg11355761	-0.457814607	3.098796297	-4.237946305	0.000305814	0.040356388
cg20867820	-0.381716613	0.036740386	-4.237471634	0.000306177	0.040367782
cg15671767	-0.691860254	2.868896285	-4.237441442	0.0003062	0.040367782
cg13163839	-0.522744438	3.081612881	-4.237365455	0.000306258	0.040367782
cg25652656	-0.424027156	-0.082863396	-4.237254471	0.000306344	0.040367782
cg18952170	-0.527915504	-2.369753541	-4.237052561	0.000306498	0.040367782
cg18417502	-0.622597425	1.139418763	-4.237023417	0.000306521	0.040367782
cg17280172	-0.825883548	2.282452614	-4.236950595	0.000306577	0.040367782
cg12575520	-0.483271767	-0.152226885	-4.236727005	0.000306748	0.040367782
cg20673820	-0.722396141	2.873943421	-4.236710509	0.000306761	0.040367782
cg19949797	-0.379452712	4.014431378	-4.236515779	0.00030691	0.040367782
cg20963903	-0.358719667	3.746618037	-4.236444268	0.000306965	0.040367782
cg23615483	0.338087079	2.126829113	4.236437256	0.000306971	0.040367782
cg05800782	-0.705587171	2.0039454	-4.236435893	0.000306972	0.040367782
cg00220905	0.555205173	-3.95373657	4.236325264	0.000307057	0.040367782
cg03014950	-0.461533802	3.517530422	-4.236293948	0.000307081	0.040367782
cg01454482	-0.947121954	1.865151736	-4.236200647	0.000307153	0.040367782
cg11947915	-0.620314976	3.010261376	-4.236157683	0.000307186	0.040367782
cg12758444	-0.837140126	3.222451213	-4.236106202	0.000307225	0.040367782
cg13469427	-0.441370652	4.369567968	-4.236054878	0.000307265	0.040367782
cg24416660	-0.463994964	-1.183263444	-4.236041161	0.000307275	0.040367782
cg03375703	-0.736270743	2.21443233	-4.235955596	0.000307341	0.040367782
cg03662501	0.630416592	2.911319306	4.23593706	0.000307355	0.040367782
cg05107094	-0.473903555	3.292908561	-4.235928064	0.000307362	0.040367782
cg01243312	0.611463057	2.590532761	4.235927199	0.000307363	0.040367782
cg14092259	0.485704418	2.241918535	4.235830131	0.000307438	0.040367782
cg21955446	-0.616280096	0.623696244	-4.235811783	0.000307452	0.040367782
cg13204189	-0.433009	-0.683259839	-4.235704652	0.000307534	0.040367782
cg10744426	-0.391673226	-0.290181715	-4.235619485	0.0003076	0.040367782
cg06927323	0.552599801	1.025646519	4.235608932	0.000307608	0.040367782
cg27468419	-0.618161338	2.520466463	-4.235606865	0.000307609	0.040367782
cg26462130	-0.510087715	0.061098387	-4.235400994	0.000307768	0.040377033
cg12326923	-0.413097848	-0.784186015	-4.235351302	0.000307806	0.040377033
cg21200501	-0.486547484	3.502893552	-4.235277431	0.000307863	0.040377033
cg00121868	-0.791359396	2.002589431	-4.235219362	0.000307908	0.040377033
cg15018391	-0.530069255	3.439925877	-4.234893384	0.000308159	0.040393975
cg04548688	-0.617194145	2.471397217	-4.23472995	0.000308285	0.040393975

cg19957337	-0.520001735	3.983845923	-4.234687495	0.000308318	0.040393975
cg19290447	-0.807524953	1.920998786	-4.23461331	0.000308375	0.040393975
cg09821226	-0.455650335	-1.206834085	-4.234545828	0.000308427	0.040393975
cg15160118	-0.449116891	4.176951274	-4.23437846	0.000308557	0.040393975
cg05598808	-0.542718773	3.452737216	-4.234343438	0.000308584	0.040393975
cg17840843	-0.358887197	-0.732195902	-4.234301151	0.000308616	0.040393975
cg05147343	-0.587965022	2.820627855	-4.234255952	0.000308651	0.040393975
cg25385318	-0.449125358	4.190021356	-4.234242012	0.000308662	0.040393975
cg20162240	-0.472446969	-0.288843497	-4.234238995	0.000308664	0.040393975
cg19646021	-0.518406465	3.361881717	-4.234074415	0.000308792	0.040397906
cg26447219	-0.536897644	3.086263657	-4.234052523	0.000308808	0.040397906
cg11751848	-0.475901901	-0.892679196	-4.233511762	0.000309227	0.040443435
cg12940993	-0.396492124	-1.187881902	-4.233454938	0.000309271	0.040443435
cg20511797	-0.459927988	1.038406271	-4.233268951	0.000309415	0.040449325
cg06557624	-0.534506635	3.364858752	-4.233249259	0.00030943	0.040449325
cg04393471	-0.37693605	3.994648775	-4.233175365	0.000309487	0.040449343
cg10139765	-0.471130638	-0.405360314	-4.232907714	0.000309695	0.04045038
cg16563203	-0.466607066	3.899913345	-4.232898675	0.000309702	0.04045038
cg22753985	-0.551809653	3.767626276	-4.232896319	0.000309703	0.04045038
cg02471028	-0.614554712	-1.27504905	-4.232804872	0.000309774	0.04045038
cg17755269	-0.437814399	-0.428167422	-4.232796698	0.000309781	0.04045038
cg17588400	-0.436473193	0.31971284	-4.232636199	0.000309905	0.040454981
cg24337173	-0.605857603	3.666378347	-4.232582738	0.000309947	0.040454981
cg22139637	-0.611061661	3.486720947	-4.232492268	0.000310017	0.040454981
cg11610614	-0.618364817	-0.863722499	-4.232456763	0.000310044	0.040454981
cg06287897	-0.72943786	-0.089672342	-4.232135179	0.000310294	0.040459679
cg25772092	-0.371881324	0.4248179	-4.231930611	0.000310453	0.040459679
cg15823375	-0.45296936	2.90323976	-4.231834071	0.000310528	0.040459679
cg26675326	0.688065648	-4.095926973	4.231823197	0.000310536	0.040459679
cg03244496	-0.545391963	0.750715356	-4.231806564	0.000310549	0.040459679
cg20528695	-0.457420278	-0.114122272	-4.231802745	0.000310552	0.040459679
cg25777422	-0.572319554	-0.28831713	-4.231696454	0.000310635	0.040459679
cg16991637	-0.601483773	-1.417244816	-4.231646719	0.000310673	0.040459679
cg07690824	-0.374251022	-0.442863487	-4.231600605	0.000310709	0.040459679
cg14418231	0.614848165	3.649138081	4.231559771	0.000310741	0.040459679
cg17436460	-0.668487239	-0.637984238	-4.231547391	0.000310751	0.040459679
cg02731443	-0.618461439	-1.475117553	-4.231528191	0.000310766	0.040459679
cg03127584	-0.73059331	1.671013738	-4.231405041	0.000310861	0.040461461
cg23611970	-0.605898155	2.909045526	-4.231238016	0.000310991	0.040461461
cg01766844	-0.388143555	4.009754245	-4.231192053	0.000311027	0.040461461
cg18197575	-0.73953196	2.25729176	-4.231188587	0.00031103	0.040461461
cg05596755	0.61871564	2.660378251	4.231143585	0.000311065	0.040461461
cg04348273	-0.702552221	1.39047481	-4.231043157	0.000311143	0.040462693
cg24788873	-0.469162021	2.22687682	-4.230984715	0.000311189	0.040462693
cg16810065	-0.649882295	3.392519497	-4.230515823	0.000311554	0.040495786
cg22681495	-0.852615232	0.21783838	-4.230097224	0.000311881	0.040495786
cg23150965	-0.667741346	2.645299191	-4.230069795	0.000311902	0.040495786

cg04365696	-0.645158555	2.726653179	-4.229997947	0.000311958	0.040495786
cg17554647	-0.589644097	-0.503915893	-4.22997376	0.000311977	0.040495786
cg04171808	-0.448741497	-0.316823865	-4.229818668	0.000312098	0.040495786
cg02978938	-0.417536795	0.106677015	-4.229753674	0.000312149	0.040495786
cg17899492	-0.68213027	0.8102774	-4.229623906	0.00031225	0.040495786
cg21253943	-0.571726394	3.397747543	-4.229618127	0.000312255	0.040495786
cg20548631	-0.447815161	-1.831237321	-4.229585978	0.00031228	0.040495786
cg15377328	-0.499852878	4.24120386	-4.229532834	0.000312321	0.040495786
cg20083388	-0.463654516	4.085561926	-4.22948025	0.000312363	0.040495786
cg14521546	-0.780912251	3.796520413	-4.229264719	0.000312531	0.040495786
cg22212938	-0.503044304	-0.158377847	-4.229248586	0.000312544	0.040495786
cg04521697	-0.659693185	2.388126739	-4.229240692	0.00031255	0.040495786
cg02531821	-0.402307042	3.573984047	-4.229210779	0.000312573	0.040495786
cg17322375	-0.570516699	3.125553781	-4.229116248	0.000312647	0.040495786
cg02785578	-0.547190445	-0.044483179	-4.228975371	0.000312758	0.040495786
cg16637194	-0.549176369	-1.962829708	-4.228933538	0.00031279	0.040495786
cg21581313	-0.413434609	2.943967738	-4.228862258	0.000312846	0.040495786
cg21476738	-0.417907697	-0.223029296	-4.228853656	0.000312853	0.040495786
cg23491387	-0.420653941	4.0125051	-4.22880929	0.000312888	0.040495786
cg02380840	-0.539786792	-0.81067771	-4.228793923	0.0003129	0.040495786
cg27228611	-0.414879842	-0.668567029	-4.22873176	0.000312948	0.040495786
cg03328201	-0.370132833	1.571547973	-4.228660974	0.000313004	0.040495786
cg12044130	-0.612351011	2.793547438	-4.228590421	0.000313059	0.040495786
cg27652345	-0.655980848	3.029384763	-4.228589182	0.00031306	0.040495786
cg03529189	-0.584719885	3.009584834	-4.228262044	0.000313316	0.040495786
cg00119670	-0.533410029	3.672762689	-4.228237082	0.000313336	0.040495786
cg21353493	-0.588342102	3.532622072	-4.228234208	0.000313338	0.040495786
cg14569423	-0.816926869	2.3429229	-4.228133585	0.000313417	0.040495786
cg05180258	-0.605760468	2.920737042	-4.228116394	0.000313431	0.040495786
cg04364764	-0.529474685	2.711688475	-4.228058971	0.000313476	0.040495786
cg22877494	-0.53230471	3.771591435	-4.228030546	0.000313498	0.040495786
cg26337954	-0.603628218	3.252939951	-4.228010871	0.000313513	0.040495786
cg20858727	-0.492690419	3.635494639	-4.227989154	0.00031353	0.040495786
cg20021470	-0.373155674	4.256362235	-4.227937647	0.000313571	0.040495786
cg23155625	-0.79093027	0.828730329	-4.227846743	0.000313642	0.040495786
cg07825983	-0.624520263	2.064166471	-4.227729755	0.000313734	0.040495786
cg08658667	-0.458678626	3.789628095	-4.227696592	0.00031376	0.040495786
cg15057359	-0.385094249	4.064858289	-4.227662304	0.000313787	0.040495786
cg21400562	-0.677594881	2.835781104	-4.227547255	0.000313877	0.040500069
cg21384940	-0.464314989	4.032045492	-4.227215875	0.000314138	0.040501427
cg14664089	-0.55880608	-1.268085154	-4.227211463	0.000314141	0.040501427
cg17447811	-0.583051252	2.394334257	-4.227203894	0.000314147	0.040501427
cg24916688	-0.665143843	3.108728063	-4.227097761	0.000314231	0.040501427
cg05447100	-0.474296452	0.799934523	-4.227053216	0.000314266	0.040501427
cg24457947	-0.416296163	3.522337593	-4.22702195	0.00031429	0.040501427
cg22731975	-0.875300818	1.245677002	-4.226967044	0.000314333	0.040501427
cg27190145	-0.355047016	-0.872576472	-4.226907317	0.00031438	0.040501427

cg23697275	-0.497501963	3.383362777	-4.226859781	0.000314418	0.040501427
cg10857341	-0.524272734	2.004554843	-4.22675101	0.000314503	0.040501427
cg12565693	-0.386258182	3.793065143	-4.226734059	0.000314517	0.040501427
cg22821358	-0.681200515	1.5484897	-4.226609191	0.000314615	0.040506722
cg08364820	-0.488343028	2.961743063	-4.226340176	0.000314827	0.040524121
cg02732777	-0.767216865	2.794401171	-4.226292372	0.000314865	0.040524121
cg11622516	-0.425509389	3.866896686	-4.226060309	0.000315048	0.040534625
cg25331127	0.701628356	2.985917358	4.225985699	0.000315106	0.040534625
cg01631162	-0.656399995	-4.319144435	-4.225900928	0.000315173	0.040534625
cg17180122	-0.42201691	3.844154302	-4.225889533	0.000315182	0.040534625
cg01064725	-0.745040225	-1.697470209	-4.225791783	0.000315259	0.040534625
cg09997171	-0.692326874	3.255211901	-4.225753464	0.00031529	0.040534625
cg12652229	-0.770098759	3.037565383	-4.225559111	0.000315443	0.040545273
cg24076474	-0.713816105	-4.464046478	-4.225503496	0.000315487	0.040545273
cg11550950	-0.453250489	-1.169070618	-4.225166691	0.000315753	0.040572103
cg17651628	-0.611680456	2.178886346	-4.224830114	0.000316019	0.040594009
cg07470162	-0.598322709	3.30802149	-4.224806059	0.000316038	0.040594009
cg10727661	-0.503493763	3.233948301	-4.22457639	0.00031622	0.040597488
cg04019060	-0.572118622	2.846433809	-4.224518014	0.000316266	0.040597488
cg12424652	-0.416806502	-0.357502917	-4.224378477	0.000316376	0.040597488
cg19086344	-0.538403108	3.691122609	-4.224339486	0.000316407	0.040597488
cg27327359	-0.454835842	3.319465609	-4.224325098	0.000316419	0.040597488
cg06078068	-0.424951738	3.219783712	-4.224201803	0.000316516	0.040597488
cg02265874	-0.97641142	1.90876637	-4.224194761	0.000316522	0.040597488
cg05833610	-0.504719834	0.087001837	-4.224143191	0.000316563	0.040597488
cg06173520	-0.534730754	2.810336277	-4.224114027	0.000316586	0.040597488
cg06343687	-0.575368557	3.067007068	-4.223990512	0.000316684	0.040597488
cg17876645	-0.614311304	-1.551778019	-4.223792823	0.00031684	0.040597488
cg07062156	-0.577958232	3.808129816	-4.22368284	0.000316928	0.040597488
cg14562851	-0.518428878	2.750142571	-4.223527733	0.000317051	0.040597488
cg18780779	-0.522174121	2.948465707	-4.223497477	0.000317075	0.040597488
cg14887576	-1.367899026	1.232318599	-4.2234594	0.000317105	0.040597488
cg07053715	-0.40624568	3.601934002	-4.22340534	0.000317148	0.040597488
cg24653707	-0.577495659	3.08451348	-4.223396138	0.000317155	0.040597488
cg17722012	-0.874785149	0.170085592	-4.223148555	0.000317352	0.040597488
cg15449419	-0.674637932	2.767442203	-4.223119888	0.000317374	0.040597488
cg13438476	-0.561630525	3.517593331	-4.223068449	0.000317415	0.040597488
cg21479418	-0.464209245	3.606452118	-4.223067679	0.000317416	0.040597488
cg02035366	-0.921113423	1.390026967	-4.222988667	0.000317479	0.040597488
cg04230325	-0.772432432	-1.076584963	-4.22284166	0.000317596	0.040597488
cg16971976	-0.47631133	3.334553505	-4.222751913	0.000317667	0.040597488
cg16838772	-0.667969568	1.92940023	-4.222746309	0.000317671	0.040597488
cg19192065	-0.488483594	-0.687797181	-4.222677784	0.000317726	0.040597488
cg01739304	-0.740442979	2.992466678	-4.222670225	0.000317732	0.040597488
cg17128799	-0.463678182	-0.382235904	-4.222469869	0.000317891	0.040597488
cg12298241	-0.406503127	4.26081806	-4.222355211	0.000317982	0.040597488
cg03764027	-0.502545889	3.58949098	-4.222328472	0.000318004	0.040597488

cg26959303	-0.575291727	3.273090095	-4.222317386	0.000318012	0.040597488
cg06476450	-0.724539923	3.072419502	-4.222251926	0.000318065	0.040597488
cg03217619	-0.614668853	3.441080424	-4.222243371	0.000318071	0.040597488
cg17264730	-0.590581501	3.399763275	-4.222213632	0.000318095	0.040597488
cg05131696	-0.476802351	3.052501127	-4.222117162	0.000318172	0.040597488
cg23460362	-0.678178213	2.195820007	-4.222050718	0.000318225	0.040597488
cg00475194	-0.405547135	3.926987687	-4.221990018	0.000318273	0.040597488
cg21944491	0.739345028	0.126851016	4.221986379	0.000318276	0.040597488
cg03704960	-0.538206364	3.964306726	-4.221926725	0.000318324	0.040597488
cg16887292	-0.849604377	0.739192136	-4.221884069	0.000318358	0.040597488
cg23629959	-0.714827731	2.764806387	-4.221671037	0.000318527	0.040601593
cg14647880	-0.737060192	3.124404083	-4.221564182	0.000318612	0.040601593
cg25764137	-0.495952215	4.195626961	-4.22151065	0.000318655	0.040601593
cg03442592	-0.422140277	3.336406807	-4.221497076	0.000318666	0.040601593
cg00886958	-0.548211107	2.845656239	-4.221484149	0.000318676	0.040601593
cg11056945	-0.649047223	3.444062016	-4.221364529	0.000318772	0.040602616
cg08350516	-0.454793593	0.746187042	-4.221311606	0.000318814	0.040602616
cg07263509	-0.549494754	3.190835208	-4.221227467	0.000318881	0.040602616
cg18403754	-0.561369918	3.914019435	-4.221162311	0.000318933	0.040602616
cg15208856	-0.542180099	3.537928401	-4.221114894	0.000318971	0.040602616
cg09037422	-0.420079709	3.873444361	-4.220895303	0.000319146	0.040606184
cg06196597	-0.493016545	-0.153511154	-4.220869386	0.000319167	0.040606184
cg18025450	-0.446151536	3.726086263	-4.220849989	0.000319182	0.040606184
cg25022205	-0.702515441	2.726221628	-4.220792653	0.000319228	0.040606184
cg24617171	-0.613666719	-1.347612336	-4.2204001	0.000319542	0.040634206
cg09949300	-0.375099846	4.291891111	-4.2203735	0.000319563	0.040634206
cg06192755	-0.396123133	4.025065153	-4.220217056	0.000319688	0.040636157
cg17950165	-0.510815045	3.899686124	-4.220210885	0.000319693	0.040636157
cg11216550	-0.402703965	-0.683406261	-4.220136887	0.000319753	0.040636391
cg25613065	-0.50055723	3.809031054	-4.219984074	0.000319875	0.040639007
cg23307744	-0.489280401	1.460337239	-4.219967799	0.000319888	0.040639007
cg22841318	-0.52820816	3.235975701	-4.219776522	0.000320041	0.040646517
cg17887588	-0.562437991	-2.266706731	-4.219750657	0.000320062	0.040646517
cg25104476	-0.731064314	2.420084916	-4.219555614	0.000320218	0.040651341
cg04378133	-0.570629004	-1.312031073	-4.219500236	0.000320262	0.040651341
cg04772833	-0.535141302	3.466923026	-4.219470238	0.000320286	0.040651341
cg16468565	-0.666516356	0.58768099	-4.219368222	0.000320368	0.040651341
cg11294103	-0.673381123	2.794030827	-4.219106259	0.000320578	0.040651341
cg01325599	0.421527363	2.351268157	4.219049489	0.000320624	0.040651341
cg04182611	-0.347388355	4.02830252	-4.219027234	0.000320642	0.040651341
cg14222093	0.493395806	2.548980285	4.21902636	0.000320642	0.040651341
cg08162226	-0.634527879	2.934463174	-4.218960751	0.000320695	0.040651341
cg14467990	-0.723356627	2.11635477	-4.218927132	0.000320722	0.040651341
cg14769192	0.80898218	-4.436467983	4.21874265	0.00032087	0.040651341
cg00077302	-0.552087006	4.028045005	-4.218734966	0.000320876	0.040651341
cg21094724	-0.498498791	3.635790136	-4.218717262	0.00032089	0.040651341
cg17263363	-0.621136188	-2.285426201	-4.218701525	0.000320903	0.040651341

cg11339582	-0.667209246	2.757191486	-4.218577911	0.000321002	0.040656648
cg14331609	-0.649435978	1.490817418	-4.218491493	0.000321072	0.040658171
cg17008375	-0.661867405	-1.418331559	-4.218328497	0.000321203	0.040664847
cg10320547	-0.553245531	3.442732395	-4.218283035	0.000321239	0.040664847
cg21030133	-0.691438265	2.887666391	-4.218081778	0.000321401	0.040676602
cg07701911	-0.489065317	3.819514656	-4.218024737	0.000321447	0.040676602
cg18244892	-0.740858266	1.918791703	-4.217728684	0.000321685	0.040689181
cg25862072	-0.520545635	0.296108112	-4.217694219	0.000321713	0.040689181
cg15428001	-0.814943813	2.358548018	-4.217687081	0.000321719	0.040689181
cg21032217	-0.534838761	3.792530532	-4.217542655	0.000321835	0.040691904
cg04637429	-0.503058912	3.696610215	-4.217318823	0.000322015	0.040691904
cg13552373	-0.552409468	0.232607069	-4.21728832	0.00032204	0.040691904
cg11394247	-0.583098972	4.23639734	-4.21718693	0.000322122	0.040691904
cg22633427	-0.49636552	3.703686536	-4.217163239	0.000322141	0.040691904
cg11395768	-0.551200581	3.854539179	-4.216952883	0.00032231	0.040691904
cg05767392	-0.485483945	3.960063137	-4.2169333	0.000322326	0.040691904
cg10633230	-0.659140262	2.598121096	-4.216865438	0.000322381	0.040691904
cg27284962	-0.47049967	3.646332394	-4.216835226	0.000322405	0.040691904
cg13779050	-0.48736431	2.984117066	-4.216762171	0.000322464	0.040691904
cg10513480	-0.76823765	-3.366531528	-4.216679326	0.000322531	0.040691904
cg05573378	-0.436828011	-0.199241421	-4.216674286	0.000322535	0.040691904
cg14136612	-0.463382709	3.510195798	-4.21666994	0.000322539	0.040691904
cg16079104	0.647476718	3.446963857	4.216633873	0.000322568	0.040691904
cg04583116	-0.519393231	3.632760145	-4.216591551	0.000322602	0.040691904
cg11080540	-0.591874091	-2.761740142	-4.216226551	0.000322897	0.040703044
cg23374902	-0.473027622	3.916157794	-4.216220717	0.000322901	0.040703044
cg01531840	-0.684928125	2.332825751	-4.216198776	0.000322919	0.040703044
cg26429783	-0.409959412	-0.258994714	-4.216150214	0.000322958	0.040703044
cg18548100	-0.691566321	3.541014703	-4.216126539	0.000322978	0.040703044
cg13552389	-0.562558009	3.687469146	-4.215892385	0.000323167	0.040719656
cg13184880	-0.453148006	-0.694960746	-4.215798003	0.000323243	0.040720357
cg22309983	-0.897653657	-0.131351906	-4.215729753	0.000323298	0.040720357
cg19309928	-1.081253345	0.646978342	-4.215672315	0.000323345	0.040720357
cg03980424	0.548428293	0.91920544	4.215590644	0.000323411	0.04072144
cg03663749	-0.815197806	2.746509303	-4.215164827	0.000323756	0.040757609
cg09962036	-0.616735211	3.232087621	-4.214816392	0.000324038	0.040762164
cg00589760	-0.626955111	3.324725098	-4.214771695	0.000324074	0.040762164
cg10821813	-0.525675185	3.367463263	-4.214717869	0.000324118	0.040762164
cg00244959	-0.518551028	3.342977856	-4.214688338	0.000324142	0.040762164
cg15721211	-0.582112911	3.556623027	-4.214457887	0.000324329	0.040762164
cg17248182	-0.574267939	3.025382206	-4.214279077	0.000324474	0.040762164
cg13758290	-0.487484748	-0.411348467	-4.214271016	0.000324481	0.040762164
cg22045206	-0.490420152	-1.131732747	-4.214244515	0.000324502	0.040762164
cg18489526	-0.471348055	4.262450586	-4.214206147	0.000324533	0.040762164
cg12957442	0.482493554	2.504130494	4.214194889	0.000324542	0.040762164
cg00351735	-0.523794548	3.92761133	-4.214184929	0.000324551	0.040762164
cg10664223	-0.379076876	3.855869381	-4.214164914	0.000324567	0.040762164

cg17815567	-0.468271847	-2.7846551	-4.21413303	0.000324593	0.040762164
cg14381171	-0.609863527	2.999504985	-4.214127121	0.000324598	0.040762164
cg23923340	-0.601829904	2.885551944	-4.214041571	0.000324667	0.040763665
cg04243822	-0.498230391	-1.002569399	-4.213830036	0.000324839	0.04077802
cg07424805	-0.534400734	3.455372173	-4.213687817	0.000324955	0.040779315
cg14379939	-0.700720576	-0.852129062	-4.21360563	0.000325021	0.040779315
cg20674490	-0.32935503	-0.389754567	-4.213570462	0.00032505	0.040779315
cg00033191	-0.45628313	3.978419243	-4.213534168	0.000325079	0.040779315
cg16636114	-0.497501747	0.957313259	-4.213188018	0.000325361	0.040786788
cg00300876	-0.612377512	2.733285134	-4.213151675	0.000325391	0.040786788
cg18025691	-0.571648504	2.895648409	-4.213126589	0.000325411	0.040786788
cg21081878	-0.416115864	0.436855507	-4.21308554	0.000325445	0.040786788
cg10566252	-0.689672537	-1.388823852	-4.213050942	0.000325473	0.040786788
cg12602145	-0.665315102	-0.042264959	-4.212955207	0.000325551	0.040786788
cg16801161	-0.996336014	0.858884193	-4.212926476	0.000325574	0.040786788
cg04896165	-0.537669325	3.136649879	-4.21289518	0.0003256	0.040786788
cg25043328	-0.533266552	4.003983518	-4.212764243	0.000325706	0.040791596
cg26781321	-2.721095543	3.211850566	-4.21270676	0.000325753	0.040791596
cg26970316	-0.622700617	1.16839028	-4.212605883	0.000325835	0.040792055
cg10693587	0.785399774	0.05313314	4.212560994	0.000325872	0.040792055
cg23811512	-0.436678495	3.591611498	-4.212410049	0.000325995	0.0407967
cg16581142	-0.453178265	3.6335414	-4.212340502	0.000326052	0.0407967
cg03908058	-0.583336272	3.351187415	-4.212161411	0.000326198	0.0407967
cg23492218	-0.487119063	2.933548977	-4.212135371	0.000326219	0.0407967
cg01321637	-0.653175213	0.812937534	-4.212130705	0.000326223	0.0407967
cg26988523	-0.488830044	3.551938355	-4.211975504	0.00032635	0.0407967
cg11813512	-0.749852186	1.874727126	-4.211938552	0.00032638	0.0407967
cg17654020	-0.474250241	4.253287649	-4.211816007	0.00032648	0.0407967
cg08917664	-0.47624433	4.375205742	-4.211582778	0.000326671	0.0407967
cg16257860	-0.476511957	2.961282652	-4.211435421	0.000326791	0.0407967
cg11998916	-0.872268485	2.540546667	-4.211431516	0.000326794	0.0407967
cg23294704	-0.581897421	2.881096571	-4.21135204	0.000326859	0.0407967
cg02429590	-0.398529826	4.24381583	-4.21132574	0.000326881	0.0407967
cg05603252	-0.401002849	-0.59339344	-4.211256638	0.000326937	0.0407967
cg04865531	-0.545111276	-0.638531037	-4.211212048	0.000326974	0.0407967
cg26913033	-0.714456709	2.44196776	-4.211211383	0.000326974	0.0407967
cg16131342	-0.817058628	2.611827043	-4.211181631	0.000326999	0.0407967
cg11890752	-0.539805977	4.00426174	-4.211145793	0.000327028	0.0407967
cg04257171	-0.568780941	2.961979962	-4.211087244	0.000327076	0.0407967
cg14623269	-0.536533784	3.329485082	-4.210971288	0.000327171	0.0407967
cg17481023	-0.61155144	3.462462923	-4.210861621	0.000327261	0.0407967
cg23171019	-0.459023217	3.840639431	-4.210847431	0.000327272	0.0407967
cg22229496	-0.721435259	1.524870612	-4.210791833	0.000327318	0.0407967
cg02325313	-0.489998806	3.549712188	-4.210775165	0.000327332	0.0407967
cg09840721	-0.405063926	3.864188797	-4.210731836	0.000327367	0.0407967
cg12690035	-0.538526565	3.521855622	-4.210629796	0.000327451	0.0407967
cg00822308	-0.478959974	4.038805516	-4.210547645	0.000327518	0.0407967

cg01709798	-0.725020121	2.609226902	-4.210462938	0.000327587	0.0407967
cg03095319	-0.55309531	0.814621861	-4.210443324	0.000327603	0.0407967
cg17698631	-0.637014074	1.932296198	-4.210362463	0.00032767	0.0407967
cg26274929	-0.478205839	-1.441167083	-4.210297709	0.000327723	0.0407967
cg02160026	-0.510548469	3.835445865	-4.210261982	0.000327752	0.0407967
cg09497113	-0.664799727	0.354647335	-4.210154269	0.00032784	0.040800527
cg05709402	-0.446371859	4.050131647	-4.209928723	0.000328026	0.040813076
cg22056102	0.747191778	-2.914681124	4.209879142	0.000328066	0.040813076
cg17646610	-0.440620219	3.683692097	-4.209820786	0.000328114	0.040813076
cg07638261	-0.959975499	1.553390773	-4.209400413	0.000328459	0.040845726
cg00533841	-0.608471257	3.582220644	-4.209360866	0.000328492	0.040845726
cg14965524	-0.350351542	-0.988447363	-4.209189264	0.000328633	0.040856097
cg13437084	-0.87754118	-3.39925464	-4.208831851	0.000328927	0.040885475
cg04977991	-0.390461221	0.110041893	-4.208667235	0.000329063	0.040885807
cg25332475	-0.626503367	3.55489007	-4.208643627	0.000329082	0.040885807
cg03966785	-0.827722097	-3.26307102	-4.208618282	0.000329103	0.040885807
cg18516397	-0.440518634	3.886968381	-4.208380507	0.000329299	0.040902965
cg23955417	-0.451673658	-0.933108368	-4.20823606	0.000329418	0.040905137
cg08644058	-0.941758988	2.597812783	-4.208219152	0.000329432	0.040905137
cg14254374	0.627030711	-0.229869477	4.208075238	0.00032955	0.040912698
cg22883453	-0.443686874	-0.940499334	-4.20790843	0.000329688	0.040918245
cg13787135	-0.516767103	-0.90003739	-4.207738148	0.000329828	0.040918245
cg25757164	-0.656067113	-0.993781083	-4.207645337	0.000329905	0.040918245
cg17512240	-0.797432728	2.427952035	-4.207564093	0.000329972	0.040918245
cg25815893	-0.620806785	2.252124171	-4.207524951	0.000330004	0.040918245
cg07677319	-0.715232535	2.747258148	-4.207518863	0.000330009	0.040918245
cg19399805	-0.731336261	2.173657337	-4.207426891	0.000330085	0.040918245
cg10784679	-0.406318347	4.026728998	-4.207365185	0.000330136	0.040918245
cg12639453	-0.716896133	-0.805087516	-4.207343861	0.000330154	0.040918245
cg07792873	-0.494230502	3.668282538	-4.207321242	0.000330173	0.040918245
cg09923796	-0.561633097	3.750359656	-4.207157112	0.000330308	0.040921548
cg18824896	-0.496768335	4.215453785	-4.207055673	0.000330392	0.040921548
cg02497921	-0.490891698	4.445222825	-4.206964276	0.000330468	0.040921548
cg12665787	0.449193555	1.401373941	4.206960618	0.000330471	0.040921548
cg03748210	-0.416865602	4.051457104	-4.206873508	0.000330543	0.040921548
cg04132713	-0.580973602	1.818375196	-4.206869684	0.000330546	0.040921548
cg14507476	-0.500271051	3.227417695	-4.206739345	0.000330654	0.040927743
cg16785000	-0.521651874	3.11631303	-4.206532688	0.000330825	0.040937548
cg16717626	-0.610893761	3.453255607	-4.206464947	0.000330881	0.040937548
cg13048334	-0.53821546	3.376301912	-4.206434155	0.000330906	0.040937548
cg18323984	-0.663057044	3.493751401	-4.206299925	0.000331017	0.040937978
cg14304326	-0.491999487	-0.506566555	-4.206177678	0.000331119	0.040937978
cg25481874	-0.429305522	-0.510867344	-4.20615628	0.000331136	0.040937978
cg08404082	-0.53028377	4.044166119	-4.20612916	0.000331159	0.040937978
cg26054943	-0.489248486	3.116492717	-4.206057563	0.000331218	0.040937978
cg12270633	-0.423167562	3.440288943	-4.205959029	0.0003313	0.040937978
cg03046538	-0.688604485	2.470613606	-4.20594166	0.000331314	0.040937978

cg07612694	-0.465033712	2.765294664	-4.205805805	0.000331427	0.040944754
cg04680436	-0.435670257	-0.868568174	-4.205689154	0.000331524	0.040949565
cg20860425	-0.478735871	3.311635936	-4.205446228	0.000331725	0.04095659
cg13861461	-0.762116954	1.311106222	-4.205314842	0.000331834	0.04095659
cg12853418	-0.559728449	3.336779634	-4.205251643	0.000331887	0.04095659
cg11017613	-0.527359858	-2.617277406	-4.205250926	0.000331887	0.04095659
cg01701297	-0.561995185	3.840595468	-4.205212312	0.000331919	0.04095659
cg15791088	-0.497978277	3.098513677	-4.205149753	0.000331971	0.04095659
cg02840399	-0.585426688	2.311312124	-4.205133053	0.000331985	0.04095659
cg20683802	-0.487672144	-0.249076894	-4.20486184	0.000332211	0.040975022
cg15670074	-0.497272427	-0.985259762	-4.204814044	0.00033225	0.040975022
cg09486032	-0.733423492	3.090909827	-4.204606623	0.000332423	0.040984403
cg07563943	-0.597237824	3.47539329	-4.204583408	0.000332442	0.040984403
cg10587254	-0.517939667	3.415898234	-4.204388321	0.000332604	0.040996365
cg17275844	-0.566598906	3.622415596	-4.204287972	0.000332688	0.040996365
cg12413263	-0.419814946	4.156909752	-4.204216095	0.000332748	0.040996365
cg23280807	-0.576311277	1.353291388	-4.204188637	0.000332771	0.040996365
cg09881355	-0.547028394	2.951415937	-4.203746862	0.000333139	0.041020776
cg21399344	-0.454684373	-0.644492184	-4.203716545	0.000333164	0.041020776
cg23258033	-0.601649092	2.993824671	-4.203684198	0.000333191	0.041020776
cg22648168	-0.636068645	0.482553643	-4.203656022	0.000333214	0.041020776
cg27267820	-0.682875328	2.411625071	-4.203555258	0.000333298	0.041020776
cg16905092	-0.792371731	2.220247247	-4.203533882	0.000333316	0.041020776
cg24654737	-0.53337347	3.502478784	-4.203398973	0.000333429	0.041023664
cg02322889	-0.423546254	-1.094848109	-4.203320117	0.000333495	0.041023664
cg24172166	-0.52331925	3.184261771	-4.203228047	0.000333571	0.041023664
cg22429706	-0.551878559	1.347076701	-4.203179057	0.000333612	0.041023664
cg19964927	-0.390100512	-0.288370292	-4.203158705	0.000333629	0.041023664
cg16953816	-0.599196153	-1.985752698	-4.20302565	0.00033374	0.041030201
cg08992854	-0.540898676	-0.171828234	-4.202882884	0.00033386	0.041031457
cg26182421	-0.681346221	2.793079002	-4.202874711	0.000333866	0.041031457
cg00823843	-0.387917047	-0.567885004	-4.202669292	0.000334038	0.041045429
cg11411509	-0.610044167	1.531492536	-4.202478856	0.000334197	0.041046757
cg25015733	-0.409593389	-0.307710834	-4.202318055	0.000334332	0.041046757
cg14432578	-0.586602364	1.665286729	-4.20220091	0.00033443	0.041046757
cg24958091	-0.834479585	2.241071962	-4.202152063	0.000334471	0.041046757
cg13207938	-0.566790336	3.5330889	-4.202102157	0.000334512	0.041046757
cg05503824	-0.403764238	4.183509155	-4.201992806	0.000334604	0.041046757
cg22995149	-0.593915654	3.662177015	-4.201969401	0.000334624	0.041046757
cg16558333	-0.551037323	2.054060125	-4.201895006	0.000334686	0.041046757
cg22317498	-0.609445131	3.095646278	-4.201763746	0.000334796	0.041046757
cg21921964	-0.4517522	3.227687174	-4.201665286	0.000334878	0.041046757
cg00494544	0.52803879	1.264213863	4.201627187	0.00033491	0.041046757
cg19123916	0.68052001	3.249884907	4.201599294	0.000334934	0.041046757
cg01645869	-0.424189343	3.480631647	-4.201476769	0.000335036	0.041046757
cg06669062	-0.517492609	0.339513232	-4.201459053	0.000335051	0.041046757
cg24976686	-0.412900217	3.41070448	-4.201455389	0.000335054	0.041046757

cg21526530	-0.624980461	2.417670606	-4.201434209	0.000335072	0.041046757
cg15428654	-0.587316659	3.192552671	-4.201404457	0.000335097	0.041046757
cg16175009	-0.618242779	3.22319639	-4.201349864	0.000335143	0.041046757
cg09972632	-0.616989617	2.623509767	-4.201234504	0.000335239	0.041046757
cg07754644	-0.546661309	3.532250222	-4.201171764	0.000335292	0.041046757
cg09678751	-0.603146168	3.760550565	-4.201125517	0.000335331	0.041046757
cg15337497	-0.468556703	3.774195916	-4.201102069	0.00033535	0.041046757
cg02668903	-0.586707618	2.67994511	-4.20103337	0.000335408	0.041046757
cg07300076	0.673183114	2.36043777	4.200995886	0.00033544	0.041046757
cg02378074	-0.449970962	3.733078984	-4.200695592	0.000335692	0.041053443
cg09489980	-0.418202877	0.548758417	-4.200643577	0.000335735	0.041053443
cg13960999	-0.494688434	-1.012933739	-4.200583104	0.000335786	0.041053443
cg20871164	-0.709474917	0.161560319	-4.200513342	0.000335845	0.041053443
cg13619387	-0.842159079	0.439844613	-4.200448205	0.0003359	0.041053443
cg10163136	-0.447837894	0.2479954	-4.200423345	0.00033592	0.041053443
cg24830559	0.453351677	1.629104225	4.200417784	0.000335925	0.041053443
cg19881032	-0.406856334	0.381579013	-4.200295212	0.000336028	0.041053443
cg17951086	-0.424523107	-0.178786677	-4.20015964	0.000336142	0.041053443
cg11123720	-0.410674381	0.149845832	-4.200142928	0.000336156	0.041053443
cg21107200	-0.531374614	2.493853762	-4.199984044	0.00033629	0.041053443
cg06582679	-0.708505113	2.873559692	-4.199952791	0.000336316	0.041053443
cg14629655	-0.461477783	3.518962798	-4.199952607	0.000336316	0.041053443
cg15187789	-0.527330395	2.802956932	-4.199737356	0.000336497	0.041053443
cg13240984	-0.519726261	-3.551065357	-4.199731465	0.000336502	0.041053443
cg25065440	-0.471911289	3.840532108	-4.199633386	0.000336585	0.041053443
cg22593669	-3.987835219	-6.108512717	-4.199608755	0.000336606	0.041053443
cg19435810	-0.630147813	2.899772132	-4.19950142	0.000336696	0.041053443
cg08313893	-0.795684862	0.96249058	-4.199500682	0.000336697	0.041053443
cg16753209	-0.627225152	2.643953104	-4.199396177	0.000336785	0.041053443
cg08177293	-0.692597672	-0.947401847	-4.19935515	0.000336819	0.041053443
cg00760014	-0.596831517	-0.97542464	-4.199191612	0.000336957	0.041053443
cg21094405	-0.450582962	-2.29826358	-4.199079557	0.000337052	0.041053443
cg17321561	-0.603458521	4.34434463	-4.198941842	0.000337168	0.041053443
cg07855639	-0.598254523	-1.17016641	-4.198866889	0.000337231	0.041053443
cg24857560	-0.674196503	2.590046414	-4.198760143	0.000337321	0.041053443
cg08088989	0.537408234	1.673753291	4.198720697	0.000337354	0.041053443
cg18151254	-0.710240921	2.46260035	-4.198554927	0.000337494	0.041053443
cg13626642	-0.418832953	0.040622556	-4.198505561	0.000337536	0.041053443
cg00569010	-0.52819943	-0.922835034	-4.198496607	0.000337544	0.041053443
cg26224473	-0.761240256	2.754809492	-4.19849004	0.000337549	0.041053443
cg01414738	-0.621468599	1.245262453	-4.198428272	0.000337601	0.041053443
cg01125152	-0.503705017	0.250317907	-4.198383561	0.000337639	0.041053443
cg10276517	0.732356669	2.7756325	4.198303957	0.000337706	0.041053443
cg22720392	-0.418797452	-0.046562384	-4.198168992	0.00033782	0.041053443
cg19224886	-0.555020595	3.21137002	-4.198137534	0.000337847	0.041053443
cg27374686	-0.578441861	3.146358276	-4.198121378	0.000337861	0.041053443
cg17149676	-0.365725595	4.405828693	-4.198101416	0.000337877	0.041053443

cg10278690	-0.484812663	3.55293023	-4.198089802	0.000337887	0.041053443
cg04405052	-0.569166808	3.48428647	-4.198071785	0.000337903	0.041053443
cg18516802	-0.471005018	3.432032115	-4.19801347	0.000337952	0.041053443
cg09583036	-0.41069435	4.34928333	-4.197980151	0.00033798	0.041053443
cg09795809	-0.655630798	-1.960065812	-4.197876406	0.000338068	0.041053443
cg18019938	-0.702575989	2.58587287	-4.197691002	0.000338225	0.041053443
cg01545328	-0.514256003	2.574652801	-4.197650287	0.000338259	0.041053443
cg19246654	-0.408407161	-0.620013724	-4.197600787	0.000338301	0.041053443
cg03504913	-0.640271851	2.384509654	-4.197526359	0.000338364	0.041053443
cg20696493	-0.545897191	3.640623214	-4.197384034	0.000338484	0.041053443
cg25800638	-0.719243428	-1.877074638	-4.197335205	0.000338526	0.041053443
cg20724307	-0.616299001	2.098725127	-4.197300386	0.000338555	0.041053443
cg05171729	-0.389446411	0.231332456	-4.197215966	0.000338627	0.041053443
cg10434261	-0.812979832	-2.745834633	-4.196980571	0.000338826	0.041053443
cg08993143	-0.801479376	3.168836637	-4.196967381	0.000338837	0.041053443
cg23079226	-0.44019709	0.336926385	-4.196857409	0.000338931	0.041053443
cg13083004	-0.859692308	-4.460570664	-4.1968208	0.000338962	0.041053443
cg09680719	-0.644548496	4.645746215	-4.196811649	0.000338969	0.041053443
cg05715201	-0.912766032	1.190884356	-4.196779019	0.000338997	0.041053443
cg23353813	-0.600548466	3.525462415	-4.196766924	0.000339007	0.041053443
cg13442966	-0.499025939	0.053221688	-4.19672142	0.000339046	0.041053443
cg11277483	-0.802268724	2.055254311	-4.196620927	0.000339131	0.041053443
cg09548880	-0.547362096	-1.981189799	-4.196587762	0.000339159	0.041053443
cg05784098	-0.712858055	1.329390735	-4.196482872	0.000339248	0.041053443
cg14446516	-0.336262185	4.120778718	-4.196468117	0.000339261	0.041053443
cg04945860	-0.634331212	3.377032446	-4.196398981	0.00033932	0.041053443
cg05258937	-0.486946401	3.84019548	-4.196334514	0.000339374	0.041053443
cg11331992	-0.766886848	1.838321237	-4.196323041	0.000339384	0.041053443
cg06746836	-0.614009636	3.216658783	-4.196322161	0.000339385	0.041053443
cg00352369	-0.473840656	3.929234586	-4.196148149	0.000339533	0.041053443
cg16935258	-0.496561212	3.222005496	-4.196107778	0.000339567	0.041053443
cg23514107	-0.547355311	2.601384807	-4.196104735	0.000339569	0.041053443
cg07246187	-0.422040111	2.911755391	-4.19600321	0.000339656	0.041053443
cg10214820	-0.876352596	2.592550395	-4.195962493	0.00033969	0.041053443
cg02498227	-0.41549736	0.358339152	-4.195892145	0.00033975	0.041053443
cg19724485	-0.547888848	3.519209713	-4.195808349	0.000339821	0.041053443
cg07322117	-0.832889809	-2.342785559	-4.195785593	0.000339841	0.041053443
cg17787809	-0.5748899	3.216741158	-4.195580175	0.000340015	0.041067542
cg05148404	-0.507241444	2.591289513	-4.195322923	0.000340234	0.041071624
cg02567958	-0.419828986	-0.05411744	-4.19531101	0.000340244	0.041071624
cg08809982	-0.46118021	3.951798967	-4.195303681	0.000340251	0.041071624
cg05623594	-0.641868572	2.860591603	-4.195267931	0.000340281	0.041071624
cg06871149	-0.644100081	3.518758952	-4.194967105	0.000340537	0.041080593
cg07921765	-0.472995295	3.7813851	-4.194924691	0.000340573	0.041080593
cg01680081	-0.481690156	3.587443399	-4.194917839	0.000340579	0.041080593
cg11017498	-0.576959617	3.219741876	-4.194908312	0.000340587	0.041080593
cg25706713	-0.470562479	3.359318334	-4.194803797	0.000340676	0.041083769

cg13451417	-0.590373285	3.153743484	-4.194741307	0.00034073	0.041083769
cg08059631	-0.667920442	2.373010587	-4.194660945	0.000340798	0.041085036
cg24524946	-0.453787812	-0.468197046	-4.194583938	0.000340864	0.04108596
cg21857660	-0.536068962	4.046111133	-4.194281914	0.000341122	0.041103251
cg11520644	0.618883082	1.730042928	4.194279743	0.000341123	0.041103251
cg08372346	-0.382172666	0.720043295	-4.19388388	0.000341461	0.041132506
cg12319001	-0.527555663	3.300036696	-4.193859373	0.000341482	0.041132506
cg10349233	0.489693016	1.708598128	4.193754339	0.000341572	0.041136321
cg22927244	-0.572921677	2.813379964	-4.193679297	0.000341636	0.041136613
cg04934757	-0.754359056	3.381483978	-4.193593486	0.00034171	0.041136613
cg08121550	-0.474659412	-1.111645793	-4.193541659	0.000341754	0.041136613
cg20019897	1.214417958	2.99883123	4.193479764	0.000341807	0.041136613
cg21621104	-0.806589489	-0.574188997	-4.193252637	0.000342001	0.041144338
cg12724546	-0.546464022	3.263065379	-4.193179762	0.000342063	0.041144338
cg07810650	-0.522148585	3.70452825	-4.193029704	0.000342192	0.041144338
cg18000566	-0.480837701	2.763656043	-4.19300551	0.000342213	0.041144338
cg21989765	-0.409120721	4.094813136	-4.192969971	0.000342243	0.041144338
cg02632227	-0.522753552	4.29463094	-4.192961864	0.00034225	0.041144338
cg04468444	-0.724985786	2.244298416	-4.192929597	0.000342278	0.041144338
cg17423978	-0.452470383	3.857303737	-4.192595591	0.000342564	0.041169724
cg01114088	-0.659235543	-1.999577167	-4.192523923	0.000342625	0.041169724
cg22126976	-0.525661289	-1.209083073	-4.192309695	0.000342809	0.041169724
cg02423711	-0.43060758	3.954504393	-4.192193896	0.000342908	0.041169724
cg10205695	-0.522903418	0.322670643	-4.192190101	0.000342911	0.041169724
cg14727763	-0.502687289	3.177833997	-4.192157358	0.000342939	0.041169724
cg09831558	-0.505730929	3.516231899	-4.192141863	0.000342953	0.041169724
cg05438991	-0.572156549	3.200813532	-4.192036492	0.000343043	0.041169724
cg15878839	-0.547829641	3.586351803	-4.191995727	0.000343078	0.041169724
cg15383186	-0.635694438	2.347218072	-4.191946405	0.00034312	0.041169724
cg00217584	-0.396187352	-0.3350359	-4.191906572	0.000343155	0.041169724
cg02974628	0.840837443	-4.40236779	4.191869872	0.000343186	0.041169724
cg27633762	-0.464625003	3.013104789	-4.191725185	0.00034331	0.041177655
cg27582081	-0.474936287	3.383316479	-4.191607031	0.000343412	0.0411821
cg05248368	-0.422116861	-0.046889039	-4.191546692	0.000343464	0.0411821
cg12964970	-0.461827961	1.118754585	-4.191406831	0.000343584	0.041186526
cg04919190	0.856641051	-4.76689243	4.191368427	0.000343617	0.041186526
cg03186232	-0.977074012	2.136824841	-4.191292199	0.000343682	0.041187412
cg02173098	-0.540126388	0.358333815	-4.191218472	0.000343746	0.041187486
cg15459243	-0.680707862	2.937651027	-4.191035253	0.000343903	0.041187486
cg03435698	-0.582313297	3.448309676	-4.191031767	0.000343906	0.041187486
cg21039221	-0.947158787	2.086678151	-4.190950826	0.000343976	0.041187486
cg15261730	-0.433579182	-0.983467553	-4.190753843	0.000344146	0.041187486
cg16653509	-0.50044059	-1.122238732	-4.190731524	0.000344165	0.041187486
cg11815363	-0.501379858	-4.293816955	-4.190628669	0.000344253	0.041187486
cg19725321	-0.632252271	3.54937042	-4.190556872	0.000344315	0.041187486
cg15631184	-0.678484292	2.985254089	-4.190537366	0.000344332	0.041187486
cg18799867	-0.594005932	0.35519648	-4.190469328	0.000344391	0.041187486

cg26725047	-0.712888272	1.38509526	-4.190403447	0.000344447	0.041187486
cg20637405	-0.473301169	-0.138703202	-4.190378076	0.000344469	0.041187486
cg08093568	-0.704291714	0.841141608	-4.190292065	0.000344543	0.041187486
cg23340538	-0.4615373	3.655065828	-4.190281413	0.000344553	0.041187486
cg27080043	-0.627263357	2.866853506	-4.190278354	0.000344555	0.041187486
cg13833040	0.452526125	1.785962666	4.19014922	0.000344667	0.041193842
cg26101468	-0.494421635	-0.722696518	-4.190012551	0.000344784	0.041200977
cg07452406	-0.665706112	3.357608199	-4.189664627	0.000345085	0.041209512
cg27329737	-0.62069466	2.919444223	-4.189650361	0.000345097	0.041209512
cg26223788	-0.597570487	-1.485374605	-4.189622477	0.000345121	0.041209512
cg21460868	-0.621426425	3.459371318	-4.18955019	0.000345183	0.041209512
cg12518626	-0.442338964	3.527208906	-4.189403061	0.00034531	0.041209512
cg11131376	-0.385466499	0.041659773	-4.189342913	0.000345362	0.041209512
cg06358270	-0.420506171	-0.639508039	-4.189218258	0.00034547	0.041209512
cg26438665	-0.724804368	2.991096209	-4.189218069	0.00034547	0.041209512
cg19993014	-0.477624882	4.41104526	-4.18917396	0.000345508	0.041209512
cg22843684	-0.497030499	0.133464644	-4.189098332	0.000345574	0.041209512
cg01331033	-0.561205493	1.195225204	-4.189063166	0.000345604	0.041209512
cg03813291	-0.467745087	3.517687875	-4.189017894	0.000345643	0.041209512
cg15341354	-0.803821347	2.365697227	-4.18879226	0.000345838	0.041209512
cg01347035	-1.001545444	2.662563934	-4.188717354	0.000345903	0.041209512
cg06951009	-0.700737117	2.454253229	-4.188691843	0.000345925	0.041209512
cg02182794	-0.458857397	3.866362671	-4.188641288	0.000345969	0.041209512
cg11499149	-0.895077078	0.755784705	-4.18860617	0.000346	0.041209512
cg16611213	-0.550709293	3.504652244	-4.188489395	0.000346101	0.041209512
cg13998543	-0.639413469	2.061929558	-4.18835249	0.000346219	0.041209512
cg26945894	-0.563945878	3.821545047	-4.188195227	0.000346355	0.041209512
cg13761806	-0.625809309	3.133791054	-4.188123195	0.000346418	0.041209512
cg17807806	-0.51316153	3.678020108	-4.18811676	0.000346423	0.041209512
cg13268387	-0.662280703	3.669468459	-4.188055485	0.000346476	0.041209512
cg05878107	-0.765218289	2.809037226	-4.18782672	0.000346675	0.041209512
cg02849953	0.896701432	-3.012661825	4.187749824	0.000346741	0.041209512
cg22175254	-0.720424893	1.368899073	-4.187707439	0.000346778	0.041209512
cg13258697	-0.830894748	2.225145639	-4.187624308	0.00034685	0.041209512
cg04252513	1.045973856	-3.726266408	4.187622181	0.000346852	0.041209512
cg20803301	-0.503240262	3.885296565	-4.187587406	0.000346882	0.041209512
cg19726363	-0.721801944	1.423769063	-4.187569194	0.000346898	0.041209512
cg19404354	-0.639221179	-1.043796374	-4.187556269	0.000346909	0.041209512
cg13974501	-0.4744591	3.696987116	-4.187550938	0.000346914	0.041209512
cg23959567	-0.475968596	3.598048845	-4.187506048	0.000346953	0.041209512
cg10206184	-0.418414601	-0.592453504	-4.187466868	0.000346987	0.041209512
cg19985362	-0.604081312	-0.021204883	-4.18745373	0.000346998	0.041209512
cg14530362	-0.543344855	3.210198862	-4.187310106	0.000347123	0.041209512
cg13627319	-0.626207902	3.523532554	-4.187275543	0.000347153	0.041209512
cg05243899	-0.640839287	0.831954933	-4.187192047	0.000347226	0.041209512
cg21954465	-0.62661788	2.963337181	-4.187188855	0.000347228	0.041209512
cg11108065	-0.66249119	3.113971581	-4.187140377	0.000347271	0.041209512

cg21619224	-0.476908337	3.876661598	-4.187065258	0.000347336	0.041209512
cg27345065	-0.782639481	2.558511284	-4.187045278	0.000347353	0.041209512
cg05592260	-0.467341392	3.131309032	-4.18704049	0.000347357	0.041209512
cg27573576	-0.459797938	-0.414007554	-4.186788032	0.000347577	0.041216654
cg04007892	-0.708317686	0.700710603	-4.186655648	0.000347692	0.041216654
cg20991003	-0.468573648	-1.253083709	-4.186596181	0.000347744	0.041216654
cg24347251	-0.558953461	3.002970543	-4.186590951	0.000347748	0.041216654
cg21421270	-0.558765632	-0.312448727	-4.186535166	0.000347797	0.041216654
cg05634486	-0.48168481	4.327690078	-4.186502869	0.000347825	0.041216654
cg20436206	-0.758570108	0.38007476	-4.186495957	0.000347831	0.041216654
cg25435020	0.519246141	0.940264785	4.186394453	0.000347919	0.041216654
cg07718844	-0.577827712	3.592936281	-4.186369108	0.000347941	0.041216654
cg07829075	-0.515922196	0.611401654	-4.18616472	0.000348119	0.041224363
cg11515284	-0.426020527	0.279971715	-4.186139252	0.000348141	0.041224363
cg09428934	-0.501161886	3.236738442	-4.186033327	0.000348234	0.041224363
cg18695750	-0.452760741	2.659647242	-4.186027027	0.000348239	0.041224363
cg10599446	-0.482274545	3.351898272	-4.185927296	0.000348326	0.041227759
cg20964248	-0.656480046	2.714351341	-4.185853831	0.00034839	0.041228448
cg15724607	-0.500631355	2.48946255	-4.185545951	0.000348658	0.04124384
cg25616540	-0.630775541	2.770551763	-4.185513542	0.000348687	0.04124384
cg06911270	-0.659166233	-4.180584235	-4.185464695	0.000348729	0.04124384
cg23963351	-0.52970123	-3.328774623	-4.185437553	0.000348753	0.04124384
cg10502424	1.13910606	2.65969484	4.18516787	0.000348988	0.041264784
cg11103623	-0.519160365	3.678566937	-4.184943597	0.000349184	0.041278616
cg21371900	-0.387833294	4.05411251	-4.184900448	0.000349222	0.041278616
cg18138085	-1.092194223	1.088715064	-4.18469123	0.000349405	0.04128294
cg12714415	-0.536740205	0.935538892	-4.18422323	0.000349814	0.04128294
cg17176209	-0.520311612	4.026942784	-4.184215275	0.000349821	0.04128294
cg25936567	-0.499052927	3.610347173	-4.184177702	0.000349854	0.04128294
cg23821480	-0.480463376	3.120578056	-4.183974866	0.000350031	0.04128294
cg20907586	-0.900383841	1.21000886	-4.183890623	0.000350105	0.04128294
cg02838443	-0.445680994	4.331299197	-4.183889655	0.000350106	0.04128294
cg09795127	-0.467160774	3.876519365	-4.183888655	0.000350107	0.04128294
cg19009962	-0.460630464	-0.02318208	-4.183887288	0.000350108	0.04128294
cg17709076	-0.659359343	3.170259189	-4.183782653	0.0003502	0.04128294
cg02227331	0.522128958	2.504185891	4.183677221	0.000350292	0.04128294
cg24914185	-0.478984158	-1.414065728	-4.183624219	0.000350338	0.04128294
cg02154304	-0.458772531	-1.487594836	-4.183573906	0.000350382	0.04128294
cg00889791	-0.485097192	-0.68928259	-4.183421308	0.000350516	0.04128294
cg10912062	-0.618871556	2.199717623	-4.183326239	0.0003506	0.04128294
cg06045746	-0.610262939	2.920813254	-4.183273948	0.000350645	0.04128294
cg05848045	-0.412478948	3.608224206	-4.183249188	0.000350667	0.04128294
cg17445690	-0.784950064	-1.134281917	-4.183200437	0.00035071	0.04128294
cg26331354	-0.502474461	4.148859991	-4.183184204	0.000350724	0.04128294
cg03598921	-0.766725981	1.894086382	-4.183128876	0.000350773	0.04128294
cg20260904	-0.928582567	2.054415881	-4.18312729	0.000350774	0.04128294
cg18330784	-0.461249304	3.657937795	-4.183121176	0.000350779	0.04128294

cg12706884	-0.851809951	1.149119457	-4.183053888	0.000350839	0.04128294
cg11433207	-0.534773374	3.34269828	-4.182984388	0.0003509	0.04128294
cg13674088	-0.430303484	3.949390717	-4.182958127	0.000350923	0.04128294
cg01440285	-0.605158063	3.261630828	-4.1829096	0.000350965	0.04128294
cg00675121	-0.563978331	-0.315327615	-4.182851104	0.000351017	0.04128294
cg04540118	-0.588310667	2.197420057	-4.182842555	0.000351024	0.04128294
cg07926928	-0.492706442	3.121430987	-4.182839003	0.000351027	0.04128294
cg09449123	-0.662372677	2.909931653	-4.182801087	0.00035106	0.04128294
cg06211378	-0.640615661	2.964694483	-4.182795959	0.000351065	0.04128294
cg25982713	-0.624369205	2.590627408	-4.1826334	0.000351208	0.041292875
cg07516823	-0.514287098	3.718439107	-4.182467001	0.000351354	0.041301941
cg01245722	-0.499570123	2.829063222	-4.182380529	0.00035143	0.041301941
cg07371693	-0.531774409	2.212075115	-4.182216102	0.000351574	0.041301941
cg11908291	-0.611720595	2.570367136	-4.182175345	0.00035161	0.041301941
cg09924241	-0.734719836	2.800135997	-4.182153727	0.000351629	0.041301941
cg00808785	-0.384102783	-0.334644672	-4.182147699	0.000351635	0.041301941
cg00890658	0.422306005	1.068065315	4.182032341	0.000351736	0.041307011
cg18066158	-0.503037707	3.924228946	-4.181634633	0.000352086	0.041309992
cg17673954	-0.502410167	3.181831802	-4.181610052	0.000352108	0.041309992
cg04184257	-0.459401594	3.605803351	-4.181576361	0.000352138	0.041309992
cg24982017	-0.581624831	3.772284248	-4.181413375	0.000352281	0.041309992
cg08127694	-0.593864699	2.961075221	-4.181383044	0.000352308	0.041309992
cg06287079	-0.829608133	1.55056661	-4.181213747	0.000352457	0.041309992
cg23914026	-0.614952355	3.236900302	-4.181174728	0.000352491	0.041309992
cg11198284	-0.573083894	3.524077239	-4.18087489	0.000352756	0.041309992
cg01738604	-0.514658983	3.191633934	-4.180819434	0.000352805	0.041309992
cg19876983	-0.622078766	3.167817101	-4.180779694	0.00035284	0.041309992
cg14893932	-0.352679794	0.638442778	-4.180767257	0.000352851	0.041309992
cg12831869	-0.611431076	3.429882492	-4.180749661	0.000352866	0.041309992
cg12245706	-0.699883625	-0.696871135	-4.180737634	0.000352877	0.041309992
cg24222959	-0.499794647	3.164768414	-4.180710491	0.000352901	0.041309992
cg04685798	-0.385008062	3.888009647	-4.180708994	0.000352902	0.041309992
cg26896818	-0.839953524	1.356208882	-4.180672456	0.000352935	0.041309992
cg14281922	-0.412519997	0.300004399	-4.180654788	0.00035295	0.041309992
cg15832270	-0.613372373	2.358459072	-4.180587581	0.00035301	0.041309992
cg18211633	-0.595654151	-1.919103091	-4.180566873	0.000353028	0.041309992
cg07592535	-0.487463919	3.154941631	-4.180513642	0.000353075	0.041309992
cg13337966	-0.448826951	4.149859982	-4.180508903	0.000353079	0.041309992
cg23704369	-0.6860044	2.193948345	-4.180460691	0.000353122	0.041309992
cg24962297	0.706246507	2.691637009	4.18038102	0.000353192	0.041309992
cg08278453	-0.897487303	1.916759169	-4.180379103	0.000353194	0.041309992
cg10203207	-0.616267965	3.116032549	-4.180349939	0.000353219	0.041309992
cg06278181	-0.750055322	1.387793742	-4.180009501	0.00035352	0.041334754
cg17333416	-0.484978392	3.354913642	-4.179923603	0.000353596	0.041334754
cg25503618	-0.522667101	3.847875498	-4.179854081	0.000353658	0.041334754
cg14026451	-0.473066752	0.860994883	-4.179829551	0.000353679	0.041334754
cg04398526	-0.473224997	0.236934778	-4.179780468	0.000353723	0.041334754

cg16317713	-0.471615632	3.96219067	-4.179645708	0.000353842	0.041341869
cg03978137	-0.694093088	2.497813779	-4.179472682	0.000353995	0.041348861
cg06172089	-0.692938996	2.323201129	-4.17938324	0.000354075	0.041348861
cg23840467	-0.522691891	3.737364865	-4.179268345	0.000354176	0.041348861
cg08207566	-0.597587727	2.918585795	-4.179247921	0.000354194	0.041348861
cg18574470	0.464127027	3.012595288	4.179196563	0.00035424	0.041348861
cg07711911	-0.498981279	2.876741802	-4.179182646	0.000354252	0.041348861
cg05039282	-0.658927368	1.921197712	-4.179077863	0.000354345	0.041352885
cg16088539	-0.531611605	-4.489801896	-4.178998535	0.000354415	0.041354278
cg04530445	-1.105517168	0.438600949	-4.178848261	0.000354549	0.041362147
cg16313577	-0.503642989	-0.26526959	-4.178684524	0.000354694	0.041362147
cg13595739	-0.511859739	3.731816281	-4.178664818	0.000354711	0.041362147
cg24184547	-0.424552318	4.082121542	-4.178651722	0.000354723	0.041362147
cg22164539	-0.389863326	3.55444204	-4.178593324	0.000354775	0.041362147
cg04774139	-0.698133581	1.773454283	-4.178493895	0.000354863	0.041365627
cg10597566	-0.629453746	-0.771426908	-4.178367098	0.000354976	0.04137009
cg15435730	-0.86492317	-3.679878491	-4.178319204	0.000355018	0.04137009
cg17010038	-0.527999992	3.708165067	-4.178226006	0.000355101	0.041372929
cg02243883	-0.582715013	2.416688467	-4.17813221	0.000355184	0.041375831
cg08274821	-0.650403232	3.237270742	-4.178018315	0.000355285	0.04137798
cg05760126	-0.430339529	3.78990329	-4.177965916	0.000355332	0.04137798
cg23266130	-0.581033344	2.660527129	-4.177646342	0.000355616	0.04137798
cg17936344	-0.862942094	-1.716213655	-4.177585272	0.00035567	0.04137798
cg01605949	-0.737241212	3.066278456	-4.177496016	0.00035575	0.04137798
cg00990020	-0.524945897	3.322008053	-4.177481326	0.000355763	0.04137798
cg03597418	-0.811200743	1.739703881	-4.177378397	0.000355854	0.04137798
cg23957144	0.466374189	-3.76981067	4.177354204	0.000355876	0.04137798
cg05070931	-0.463762555	3.730784977	-4.177297343	0.000355927	0.04137798
cg12550998	-0.596665398	2.727594891	-4.177237963	0.000355979	0.04137798
cg19343186	-0.56839145	-0.458327262	-4.177158469	0.00035605	0.04137798
cg00904407	-0.608370628	2.21064289	-4.177139756	0.000356067	0.04137798
cg25916662	-0.499615536	3.713001048	-4.177023062	0.000356171	0.04137798
cg27280279	-0.793103133	-0.108166372	-4.176910725	0.000356271	0.04137798
cg12607269	-0.539626317	-0.424896176	-4.176900759	0.00035628	0.04137798
cg17671035	-0.647866811	3.063069047	-4.17678563	0.000356382	0.04137798
cg15890078	-0.488019463	4.204217494	-4.1766184	0.000356532	0.04137798
cg11697592	-0.574629888	2.850718058	-4.176605256	0.000356543	0.04137798
cg12503797	-0.529252191	-0.79231714	-4.176594677	0.000356553	0.04137798
cg00101169	-0.557077849	0.495826203	-4.176528604	0.000356612	0.04137798
cg17288879	-0.526307686	3.895068282	-4.176486197	0.000356649	0.04137798
cg12937925	-0.674356991	2.819115004	-4.176437316	0.000356693	0.04137798
cg24726121	-0.463706853	0.019506011	-4.176368682	0.000356754	0.04137798
cg19954278	-0.562002496	3.954380393	-4.17633796	0.000356782	0.04137798
cg24345902	-0.719818057	3.380028146	-4.17624039	0.000356869	0.04137798
cg03158090	-0.431180004	-0.170335916	-4.176145502	0.000356953	0.04137798
cg22815658	-0.571986621	3.398343878	-4.176008284	0.000357076	0.04137798
cg19717919	-0.587371106	3.466127245	-4.175989997	0.000357092	0.04137798

cg02988476	-0.566775935	2.610978779	-4.175971411	0.000357109	0.04137798
cg22156852	-0.431356087	4.231347836	-4.17595924	0.00035712	0.04137798
cg08946400	-0.452666807	3.208906058	-4.175844843	0.000357222	0.04137798
cg24960875	-0.474074687	3.591739336	-4.175780467	0.00035728	0.04137798
cg19248958	-0.585945583	3.553917277	-4.175718763	0.000357335	0.04137798
cg16445639	-0.554730495	2.977533356	-4.175666576	0.000357381	0.04137798
cg00618096	-0.52759797	3.755542091	-4.175613393	0.000357429	0.04137798
cg17264818	-0.544695378	4.564044667	-4.175588106	0.000357451	0.04137798
cg17682313	-0.506689057	3.612071025	-4.175493178	0.000357536	0.04137798
cg14123942	-1.126778659	-5.259782273	-4.175439258	0.000357585	0.04137798
cg06824186	-0.398866	0.407412867	-4.175336159	0.000357677	0.04137798
cg23190719	-0.496546046	-0.738049706	-4.175309677	0.0003577	0.04137798
cg07369422	-0.887546427	2.71290816	-4.175289619	0.000357718	0.04137798
cg05245416	-0.609137581	0.719017124	-4.175171508	0.000357824	0.04137798
cg26576836	-0.678687439	2.50614493	-4.175100138	0.000357888	0.04137798
cg19987965	-0.819229643	0.807410985	-4.175071926	0.000357913	0.04137798
cg17158405	-0.537524805	3.380205476	-4.174960966	0.000358013	0.04137798
cg12843510	-0.44966537	3.852031719	-4.174853954	0.000358108	0.04137798
cg04388215	-0.613068551	3.365159536	-4.174839843	0.000358121	0.04137798
cg03904639	-0.547661589	3.522172867	-4.17468677	0.000358258	0.04137798
cg10300252	-0.614771108	3.315296533	-4.174666779	0.000358276	0.04137798
cg17427751	-0.623561915	3.148138715	-4.174549983	0.000358381	0.04137798
cg19126679	-0.61993485	2.520405731	-4.174534675	0.000358394	0.04137798
cg07260806	-0.718086926	1.092878254	-4.174472039	0.000358451	0.04137798
cg27478030	-0.438534101	3.322803918	-4.174403672	0.000358512	0.04137798
cg14918582	-0.620681441	0.457880609	-4.174264427	0.000358637	0.04137798
cg23233807	-0.657175567	-1.699360666	-4.174247507	0.000358652	0.04137798
cg01016521	-0.796385075	-3.086733395	-4.174191744	0.000358702	0.04137798
cg00919068	-0.546782012	2.647647645	-4.174156621	0.000358734	0.04137798
cg20252653	-0.507537475	4.160394434	-4.174139858	0.000358749	0.04137798
cg05454830	-0.460764293	1.277102915	-4.174073381	0.000358808	0.04137798
cg09035546	-0.500010633	-0.143516355	-4.174041618	0.000358837	0.04137798
cg20519415	-0.571499716	3.657281833	-4.174011142	0.000358864	0.04137798
cg18718937	-0.584463393	2.490564692	-4.173914079	0.000358951	0.04137798
cg09886564	-0.415755734	3.684985883	-4.173801951	0.000359052	0.04137798
cg11111322	-0.437590056	0.558565447	-4.173762035	0.000359088	0.04137798
cg14359645	-0.603833837	2.246604715	-4.173721945	0.000359124	0.04137798
cg05568839	-0.54297002	3.337092763	-4.173677656	0.000359164	0.04137798
cg01808053	-0.528007962	3.194383471	-4.173626726	0.000359209	0.04137798
cg15104443	-0.596235713	2.645778211	-4.173625778	0.00035921	0.04137798
cg01525935	-0.857950081	2.042811099	-4.173600268	0.000359233	0.04137798
cg27169846	-0.7041338	0.853862002	-4.173407586	0.000359406	0.041383277
cg25349900	0.321681558	-0.222938549	4.173374015	0.000359436	0.041383277
cg02262990	-0.453879506	-0.8210478	-4.17329636	0.000359506	0.041383277
cg26693313	-0.386716049	4.023694627	-4.17324303	0.000359554	0.041383277
cg12586042	-0.491304707	3.612517422	-4.173201323	0.000359592	0.041383277
cg14469601	-0.646341014	2.677915426	-4.173159198	0.00035963	0.041383277

cg20667778	-0.630123896	2.652773607	-4.173063248	0.000359716	0.041386486
cg02963907	-0.471130816	3.50133656	-4.172938294	0.000359828	0.041390043
cg26160660	-0.573318694	1.324617668	-4.172899026	0.000359864	0.041390043
cg08001199	-0.408750602	-0.874411327	-4.172769955	0.00035998	0.041396684
cg13725340	-0.874894561	3.220309347	-4.172599361	0.000360133	0.041399363
cg04258934	-0.523666003	3.222763333	-4.172420791	0.000360294	0.041399363
cg13473795	-0.688898253	1.838733087	-4.172360121	0.000360349	0.041399363
cg19010136	-0.51242768	1.65348569	-4.172288749	0.000360413	0.041399363
cg06441457	-0.548496059	3.623302859	-4.172225938	0.00036047	0.041399363
cg06860015	-0.506970281	3.888410156	-4.172194327	0.000360498	0.041399363
cg18244255	-0.639803603	3.319210101	-4.1721584	0.000360531	0.041399363
cg21315209	-0.687642872	3.336028577	-4.172131285	0.000360555	0.041399363
cg02097398	-0.483252739	-0.609867322	-4.172115262	0.00036057	0.041399363
cg04288607	-0.548206903	3.74723273	-4.17209552	0.000360588	0.041399363
cg25342325	-0.613037806	4.036400656	-4.171941223	0.000360727	0.04140863
cg10725843	-0.557726093	3.008761199	-4.171728549	0.000360919	0.041418097
cg15791366	-0.469096044	-0.205185238	-4.171720251	0.000360926	0.041418097
cg14860676	-0.477968099	-0.206239072	-4.171560855	0.00036107	0.041422151
cg06840703	-0.726472084	2.282278411	-4.171551593	0.000361078	0.041422151
cg05527782	-0.457183154	4.155756352	-4.171395385	0.000361219	0.041431627
cg01737547	-0.76280419	2.479775911	-4.171226643	0.000361372	0.041442405
cg02992714	-0.609602902	-1.526063659	-4.171115487	0.000361472	0.041446089
cg16890762	-0.395697382	4.165259221	-4.171061665	0.000361521	0.041446089
cg22699768	-0.581928699	0.254321432	-4.170899805	0.000361667	0.04144992
cg09926548	-0.593550566	3.119849634	-4.170895298	0.000361672	0.04144992
cg27178956	-0.555035802	3.431347082	-4.170577218	0.000361959	0.041469456
cg05020517	-0.517811656	3.249544521	-4.170518235	0.000362013	0.041469456
cg07627190	-0.495148838	0.096234345	-4.170019549	0.000362465	0.041469456
cg01235387	-0.655109106	3.631793314	-4.170003358	0.000362479	0.041469456
cg22744433	-0.499255147	3.497918424	-4.169979885	0.0003625	0.041469456
cg17672311	-0.612315522	2.690058419	-4.169945992	0.000362531	0.041469456
cg22914031	-0.573902945	2.900314541	-4.169859752	0.000362609	0.041469456
cg08202265	-0.411614178	3.634446588	-4.169856492	0.000362612	0.041469456
cg18911229	-0.59455409	2.90784245	-4.169565121	0.000362877	0.041469456
cg21987970	-0.508703563	-0.535329758	-4.169534121	0.000362905	0.041469456
cg26619416	-0.526938395	3.33122359	-4.16950932	0.000362927	0.041469456
cg18565132	-0.628370158	-2.698930481	-4.169238536	0.000363173	0.041469456
cg04542977	-0.547344665	-1.161813238	-4.169224468	0.000363186	0.041469456
cg00328782	-0.465308906	0.143903794	-4.169220697	0.000363189	0.041469456
cg03012374	-0.672821948	-1.243009283	-4.169191701	0.000363216	0.041469456
cg13078965	-0.964661789	1.06689987	-4.169152307	0.000363252	0.041469456
cg08660064	-0.595317861	2.992427038	-4.169147323	0.000363256	0.041469456
cg05860426	-0.523635645	-0.329283194	-4.169114731	0.000363286	0.041469456
cg00898142	-0.558918442	3.249847681	-4.168962846	0.000363424	0.041469456
cg15179595	-0.554640415	3.255186655	-4.168958254	0.000363428	0.041469456
cg17450853	-0.785736248	3.098430572	-4.16889344	0.000363487	0.041469456
cg26683194	-0.338289913	3.977704927	-4.168714919	0.000363649	0.041469456

cg08665312	-0.672091317	2.660664013	-4.168700144	0.000363663	0.041469456
cg19548172	0.623441473	-2.775709571	4.168694902	0.000363667	0.041469456
cg04743106	-0.581130367	3.565632168	-4.168669072	0.000363691	0.041469456
cg04645521	-0.503311594	0.431116456	-4.168644616	0.000363713	0.041469456
cg02774705	-0.51132924	0.044974962	-4.168643737	0.000363714	0.041469456
cg22951773	0.6939081	-2.676754645	4.168594655	0.000363758	0.041469456
cg26103797	-0.52105433	3.711411545	-4.168559328	0.000363791	0.041469456
cg03554522	-0.532551919	2.381904377	-4.168474906	0.000363867	0.041469456
cg07477852	-0.549130204	-0.339626328	-4.168471096	0.000363871	0.041469456
cg22598885	-0.531737413	-1.220049936	-4.168464543	0.000363877	0.041469456
cg18925478	-0.552799299	1.096951855	-4.168448447	0.000363891	0.041469456
cg22017544	-0.433254013	-1.78949572	-4.168369567	0.000363963	0.041469456
cg20861649	-0.540705369	-1.12686513	-4.168363127	0.000363969	0.041469456
cg06538448	-0.65551185	2.435741729	-4.168307697	0.00036402	0.041469456
cg14530541	-0.871028893	-5.167646113	-4.16826705	0.000364057	0.041469456
cg09922092	-0.608152865	3.693158782	-4.168239564	0.000364082	0.041469456
cg16242080	-0.432514449	3.215230809	-4.168078047	0.000364229	0.041469456
cg19506005	-0.504147652	4.157765198	-4.167908776	0.000364383	0.041469456
cg23416417	-0.786955319	2.794449101	-4.16790479	0.000364387	0.041469456
cg02567119	-0.73335426	-3.227153721	-4.167820419	0.000364463	0.041469456
cg05991333	-0.545376535	0.301242634	-4.167614145	0.000364652	0.041469456
cg00597982	-0.6452147	0.376604853	-4.167564614	0.000364697	0.041469456
cg08840929	-0.514865696	3.191606267	-4.167467317	0.000364785	0.041469456
cg12383447	-0.606636818	3.197305386	-4.167349847	0.000364893	0.041469456
cg05327295	-0.468025359	3.584524998	-4.16729406	0.000364944	0.041469456
cg20759228	-0.809428707	-2.697245994	-4.167217226	0.000365014	0.041469456
cg12416902	-0.784153446	2.164847416	-4.167199504	0.00036503	0.041469456
cg02458997	0.520950679	2.053039506	4.167082822	0.000365136	0.041469456
cg18759850	-0.468166711	2.885445634	-4.167063387	0.000365154	0.041469456
cg08022044	-0.430197367	0.623010641	-4.167034778	0.00036518	0.041469456
cg24053914	0.752767083	2.097173872	4.166970724	0.000365239	0.041469456
cg14200842	-0.566925242	0.645544003	-4.166923172	0.000365282	0.041469456
cg12880059	-0.424211887	-0.11772525	-4.166770354	0.000365422	0.041469456
cg05325989	-0.477058213	-0.447872635	-4.166761612	0.00036543	0.041469456
cg08962392	-0.953576345	2.306482253	-4.1667579	0.000365433	0.041469456
cg20138055	-0.466304233	3.848813695	-4.166592838	0.000365584	0.041469456
cg00381583	-0.467033071	3.713141555	-4.166490752	0.000365677	0.041469456
cg04521633	-0.935023837	0.436139395	-4.166437452	0.000365726	0.041469456
cg23773384	-0.675314162	1.880908339	-4.166409818	0.000365751	0.041469456
cg11997749	-0.595094942	2.799859883	-4.166346134	0.00036581	0.041469456
cg02138557	-0.529886435	0.08729557	-4.166211247	0.000365933	0.041469456
cg01900995	-0.747350308	0.017217278	-4.166181615	0.00036596	0.041469456
cg02747184	-0.391133008	4.183976857	-4.166086492	0.000366047	0.041469456
cg00923632	-0.656130253	2.592129164	-4.165967217	0.000366156	0.041469456
cg06855506	-0.789882256	3.181283226	-4.16595151	0.000366171	0.041469456
cg22948672	-0.448021945	-0.382246453	-4.165893591	0.000366224	0.041469456
cg08206358	-0.678840568	3.562408267	-4.165886137	0.000366231	0.041469456

cg00558019	-0.573054467	3.816238599	-4.165831921	0.00036628	0.041469456
cg04371212	-0.5727027	-0.224315526	-4.165828734	0.000366283	0.041469456
cg20645416	-0.609182868	2.896881107	-4.165806546	0.000366304	0.041469456
cg26503038	-0.459277418	-0.982861372	-4.165751991	0.000366354	0.041469456
cg16888434	-0.603664761	3.171644928	-4.165716064	0.000366387	0.041469456
cg08661003	0.577737905	2.806845389	4.165647297	0.00036645	0.041469456
cg08796240	-0.841187457	3.978282845	-4.165427501	0.000366651	0.041469456
cg09113310	-0.35975327	-0.603003495	-4.165349666	0.000366722	0.041469456
cg26348847	-0.406592781	-0.408604434	-4.165329042	0.000366741	0.041469456
cg15881486	0.406564096	1.602462494	4.165327743	0.000366743	0.041469456
cg05108467	-0.651738435	-3.182447756	-4.165266412	0.000366799	0.041469456
cg24596368	-0.504661584	-1.24807109	-4.165181849	0.000366876	0.041469456
cg18335535	-0.538929863	0.11864052	-4.165124871	0.000366929	0.041469456
ch.2.1546374F	0.506533353	-4.173665699	4.165099255	0.000366952	0.041469456
cg22556677	-0.460751043	-0.241127005	-4.165087732	0.000366963	0.041469456
cg00021123	-0.733144094	1.117248898	-4.165075525	0.000366974	0.041469456
cg25942381	-0.630194309	3.693495586	-4.165003272	0.00036704	0.041469456
cg03938978	0.637428339	3.068901953	4.164997023	0.000367046	0.041469456
cg02452974	-0.47128662	3.906881552	-4.164986725	0.000367055	0.041469456
cg01930524	-0.452383541	0.10525857	-4.164948775	0.00036709	0.041469456
cg14655607	-0.447136764	4.064914994	-4.16492651	0.000367111	0.041469456
cg16589705	-0.453703399	3.57923948	-4.164845054	0.000367185	0.041471291
cg27505472	-0.520878349	0.364586313	-4.164646852	0.000367368	0.041474862
cg00412262	-0.390154779	4.102084545	-4.16462451	0.000367388	0.041474862
cg15036157	-0.539048688	2.996932278	-4.164619399	0.000367393	0.041474862
cg05381290	-0.442225759	4.164674113	-4.164374623	0.000367618	0.041489004
cg11118271	-0.593332155	3.488939415	-4.164355642	0.000367635	0.041489004
cg07209987	-0.464848114	-1.047525345	-4.16425131	0.000367731	0.041493219
cg25925248	-0.416244258	-0.758829683	-4.164075812	0.000367892	0.041504503
cg14656245	-0.67411481	0.29065995	-4.163876516	0.000368076	0.041504503
cg16838554	-0.552033543	3.66630422	-4.16379648	0.00036815	0.041504503
cg15226255	-0.506078056	-0.812816521	-4.163761621	0.000368182	0.041504503
cg19076258	-0.547451188	-0.497575609	-4.163756721	0.000368186	0.041504503
cg22790986	-0.477339231	4.213088839	-4.16373021	0.000368211	0.041504503
cg22899538	-0.466538839	3.550121426	-4.163571085	0.000368357	0.041504503
cg03707696	-0.558446712	1.101765567	-4.163566994	0.000368361	0.041504503
cg14824740	-0.513616297	3.788190975	-4.163408303	0.000368507	0.041504503
cg02210151	-0.393900572	-0.680450946	-4.163312742	0.000368595	0.041504503
cg08531998	-0.356655388	-1.172414245	-4.163289737	0.000368616	0.041504503
cg02724374	-0.532935848	3.992272158	-4.163260421	0.000368643	0.041504503
cg21963073	-0.352042481	3.50069092	-4.163234724	0.000368667	0.041504503
cg16257215	-0.411139235	-0.978310697	-4.163222187	0.000368679	0.041504503
cg23648926	-0.433432017	-1.852633066	-4.163188265	0.00036871	0.041504503
cg04527083	-0.532055139	-0.300141978	-4.162991684	0.000368891	0.041517923
cg14787753	-0.710367848	0.280330431	-4.162912211	0.000368965	0.041517923
cg01188509	-0.498390516	1.121175714	-4.162868387	0.000369005	0.041517923
cg10643316	-0.725589365	2.338152933	-4.162798795	0.000369069	0.041518555

cg16824024	-0.491042206	3.893431426	-4.16240725	0.000369431	0.041552627
cg02594168	-0.719854207	2.316338312	-4.162308332	0.000369522	0.041552895
cg21340024	-0.720625314	1.664352693	-4.162277706	0.00036955	0.041552895
cg18088768	-0.491167074	3.917652519	-4.16216585	0.000369654	0.041557923
cg07668586	-0.642735643	2.239445002	-4.161945835	0.000369857	0.041566069
cg19945323	-0.406288875	-0.042843966	-4.161916217	0.000369885	0.041566069
cg17070800	-0.504062192	-1.233596461	-4.161846965	0.000369949	0.041566069
cg20386818	-0.538568403	3.420435496	-4.161823634	0.00036997	0.041566069
cg08986465	-0.950282082	2.594095784	-4.16175789	0.000370031	0.041566069
cg12441919	-0.434852283	0.180119192	-4.161700697	0.000370084	0.041566069
cg18789472	-0.501920542	3.450778047	-4.161643449	0.000370137	0.041566069
cg00305629	-0.538615123	-0.740813472	-4.161300624	0.000370454	0.041588466
cg20995510	-0.617958185	3.251554316	-4.161230196	0.00037052	0.041588466
cg18496447	0.714792289	-3.129031171	4.161172112	0.000370574	0.041588466
cg11884689	-0.727227433	2.093212174	-4.161151914	0.000370592	0.041588466
cg06834177	-0.759753579	-0.924110081	-4.161111176	0.00037063	0.041588466
cg21645572	-0.918568808	1.390144907	-4.160927585	0.0003708	0.041589957
cg06494746	-0.668527926	2.655426982	-4.160924228	0.000370803	0.041589957
cg11209549	-0.718308368	1.651369115	-4.160906843	0.000370819	0.041589957
cg26023902	-0.582070199	2.441963496	-4.160707123	0.000371005	0.041597059
cg03965333	-0.514629414	3.161579048	-4.160693826	0.000371017	0.041597059
cg00855102	-0.650046894	2.87119856	-4.160648658	0.000371059	0.041597059
cg03100044	-0.494924732	-1.26860533	-4.160319484	0.000371364	0.041615266
cg18661413	-0.846743169	3.084100552	-4.160262985	0.000371417	0.041615266
cg15078322	-0.484916165	3.417335537	-4.160220993	0.000371456	0.041615266
cg14503204	-0.620887037	0.406643882	-4.160190636	0.000371484	0.041615266
cg12394642	-0.451204895	4.038204216	-4.16011068	0.000371558	0.041615266
cg01291631	-0.498550049	3.394274852	-4.160094113	0.000371574	0.041615266
cg16656819	-0.668288294	3.068370744	-4.160017906	0.000371645	0.041616618
cg03004597	-0.533593037	1.17453603	-4.159814198	0.000371834	0.041624025
cg01438012	-0.691638626	2.540900555	-4.159805922	0.000371842	0.041624025
cg05140984	-0.688757193	3.140810706	-4.159701656	0.000371939	0.041624025
cg14437092	-0.637261217	2.507154385	-4.15969394	0.000371946	0.041624025
cg26801880	-0.817888327	1.896007208	-4.159523688	0.000372104	0.041632542
cg26235836	-0.456426623	4.157013098	-4.159485784	0.000372139	0.041632542
cg14214214	-0.425275431	3.606598231	-4.15923122	0.000372376	0.041639971
cg06935085	-0.492055496	4.458361514	-4.159183193	0.000372421	0.041639971
cg04399872	-0.417059267	4.159894205	-4.159149632	0.000372452	0.041639971
cg18025831	-0.45521958	-0.789549535	-4.159084062	0.000372513	0.041639971
cg17929152	-0.51940539	3.750375201	-4.159063421	0.000372533	0.041639971
cg16342597	-0.523375413	3.174310807	-4.158990202	0.000372601	0.041639971
cg19294736	0.44822853	3.657079704	4.158936034	0.000372651	0.041639971
cg24480555	-1.016582664	-1.28694789	-4.158909493	0.000372676	0.041639971
cg07394127	-0.432890811	-0.270446217	-4.158801151	0.000372777	0.041641489
cg12496673	-0.528340104	3.508981969	-4.15873724	0.000372837	0.041641489
cg07821112	-0.531111668	3.282331266	-4.158705716	0.000372866	0.041641489
cg01221418	-0.759225074	2.667836324	-4.158517797	0.000373041	0.041647197

cg02417082	-0.800591209	2.395084691	-4.158372728	0.000373177	0.041647197
cg23506331	-0.644232916	2.49309174	-4.158208959	0.000373329	0.041647197
cg23857154	-0.539768522	-0.656711727	-4.15820688	0.000373331	0.041647197
cg26915779	-0.530621672	3.013335604	-4.158086659	0.000373444	0.041647197
cg05686542	-0.565666871	3.826755783	-4.158085763	0.000373445	0.041647197
cg06888156	-0.599685544	3.450485153	-4.158068998	0.00037346	0.041647197
cg27208573	-0.469807288	3.761322643	-4.15797404	0.000373549	0.041647197
cg19540546	-0.487119202	4.003211573	-4.157767711	0.000373742	0.041647197
cg19391667	-0.363843236	-0.680901332	-4.157690413	0.000373814	0.041647197
cg15026820	-0.605291922	3.623570334	-4.157654639	0.000373847	0.041647197
cg25351763	-0.445141891	3.745219024	-4.15760283	0.000373896	0.041647197
cg03095964	-0.574363498	3.488529287	-4.157557307	0.000373938	0.041647197
cg20817899	-0.64294336	3.292887138	-4.157535485	0.000373959	0.041647197
cg00533202	0.784271313	2.242808521	4.15752827	0.000373965	0.041647197
cg13226797	-0.535979133	0.395984721	-4.15743301	0.000374055	0.041647197
cg20631820	-0.567356899	-0.825594342	-4.157404334	0.000374081	0.041647197
cg02091207	-0.504069044	0.028898776	-4.157358951	0.000374124	0.041647197
cg03361504	-0.441853669	3.086400484	-4.157261052	0.000374215	0.041647197
cg12035144	2.573602585	-1.01548935	4.157245353	0.00037423	0.041647197
cg26834504	-0.482278411	3.779328517	-4.15718699	0.000374285	0.041647197
cg11632652	-0.634977812	3.043090205	-4.157163692	0.000374307	0.041647197
cg19185791	-0.666816021	3.048513982	-4.157134004	0.000374334	0.041647197
cg03448362	-0.728302129	2.178359041	-4.15707174	0.000374393	0.041647197
cg06725965	-0.535274139	2.755825938	-4.157035145	0.000374427	0.041647197
cg14275148	-0.773017637	1.260162519	-4.156942449	0.000374514	0.041647197
cg11841894	-0.496622907	-0.480579016	-4.156737349	0.000374706	0.041647197
cg24950356	-0.695012074	2.847190087	-4.156730877	0.000374712	0.041647197
cg00775011	-0.362765848	4.245425589	-4.156689118	0.000374751	0.041647197
cg09533013	-0.53796914	3.786385894	-4.156646892	0.000374791	0.041647197
cg24801835	-0.451617113	-0.265220418	-4.15664534	0.000374792	0.041647197
cg13729177	-0.502333546	0.339523667	-4.156574703	0.000374858	0.041647197
cg00710180	-0.525639125	-1.631509885	-4.156506723	0.000374922	0.041647197
cg26222498	-0.429512108	0.364882512	-4.156434995	0.000374989	0.041647197
cg15408793	-0.480640828	0.627879131	-4.156382232	0.000375039	0.041647197
cg11550618	-0.428627299	3.468950053	-4.156370128	0.00037505	0.041647197
cg07909630	-0.490641693	0.555331628	-4.156324839	0.000375092	0.041647197
cg06417305	-0.529855501	3.147675112	-4.156059803	0.000375341	0.041659668
cg00746293	-0.645991303	3.27668814	-4.156047651	0.000375353	0.041659668
cg27627854	-0.575221331	3.533214443	-4.155956805	0.000375438	0.041659668
cg26679004	-0.668321418	-1.289252935	-4.155942464	0.000375451	0.041659668
cg15135286	-0.439421552	-0.963419885	-4.155856749	0.000375532	0.041659668
cg01802916	-0.8963405	2.363980174	-4.15582916	0.000375558	0.041659668
cg20584801	-0.42227535	0.488049619	-4.155655242	0.000375721	0.041671263
cg24076968	-0.53118772	-1.31090339	-4.155482868	0.000375883	0.041677567
cg02912281	-0.479803589	-0.357127398	-4.155469531	0.000375895	0.041677567
cg19554547	-0.460483948	4.105805452	-4.155339437	0.000376018	0.04167961
cg03275611	-0.42392003	3.816996723	-4.155276112	0.000376077	0.04167961

cg08374732	-0.617254933	3.750537739	-4.155262175	0.00037609	0.04167961
cg26296901	-0.491207017	-0.369939406	-4.155172656	0.000376175	0.04168242
cg24911730	-0.514428399	3.32695711	-4.154994257	0.000376342	0.041694495
cg20934892	-0.514275687	2.538909179	-4.154912037	0.00037642	0.041694977
cg18881778	-0.758723879	-2.89881968	-4.154864558	0.000376465	0.041694977
cg19801817	-0.730023814	1.416124224	-4.154790077	0.000376535	0.041696223
cg00226580	-0.541848667	3.399427714	-4.154581631	0.000376731	0.041707287
cg26232857	-0.584697817	3.394706843	-4.154558949	0.000376752	0.041707287
cg08761611	-0.60442164	3.257575241	-4.154349444	0.00037695	0.04172262
cg02018291	-0.487325442	-1.719349385	-4.154246322	0.000377047	0.041723939
cg19071098	-0.567773922	0.463452941	-4.154211837	0.000377079	0.041723939
cg16184413	-0.576658501	2.483307726	-4.153860322	0.000377411	0.041750872
cg01591526	-0.569663156	3.689469666	-4.153828837	0.000377441	0.041750872
cg10373803	-0.675895644	0.444468343	-4.153716281	0.000377547	0.041754834
cg02483911	-0.43638106	3.562866635	-4.153616781	0.000377641	0.041754834
cg15817287	-0.534397653	2.957694804	-4.153603568	0.000377653	0.041754834
cg11995282	-0.525851066	3.715356789	-4.153453996	0.000377795	0.041757081
cg23350224	-0.653229917	-2.226704581	-4.153303604	0.000377937	0.041757081
cg26555801	-0.864570074	2.281927284	-4.153257224	0.000377981	0.041757081
cg14219156	-0.419348043	2.847410321	-4.153104945	0.000378124	0.041757081
cg08670568	-0.839755458	1.907600687	-4.153059612	0.000378167	0.041757081
cg11195910	-0.531926892	0.294946037	-4.152980311	0.000378242	0.041757081
cg24032621	-0.545481722	3.078755989	-4.152978492	0.000378244	0.041757081
cg06094379	-0.467887627	3.109368003	-4.152845531	0.00037837	0.041757081
cg06918474	-1.091724068	-2.752414146	-4.152841283	0.000378374	0.041757081
cg09556817	-0.576961945	2.88181248	-4.152835076	0.00037838	0.041757081
cg13932501	-0.551146218	-0.121796958	-4.152772433	0.000378439	0.041757081
cg24315653	-0.833696085	2.680001919	-4.152745292	0.000378465	0.041757081
cg00431924	-0.575849021	3.619442866	-4.152685438	0.000378521	0.041757081
cg26704011	-0.606221878	3.097159488	-4.152425651	0.000378767	0.041757081
cg22365834	-0.5585594	3.059564322	-4.152404688	0.000378787	0.041757081
cg23461824	-0.435986804	3.038191703	-4.15238902	0.000378802	0.041757081
cg04790830	-0.43638764	4.258358677	-4.152323912	0.000378864	0.041757081
cg04280449	-0.59461582	3.446463429	-4.152321066	0.000378866	0.041757081
cg16221513	0.501351392	3.869054904	4.152207735	0.000378974	0.041757081
cg25457886	-0.689108761	-2.054119792	-4.152129582	0.000379048	0.041757081
cg02697491	-0.35830113	3.93226538	-4.152102004	0.000379074	0.041757081
cg22372731	-0.470959891	3.760154491	-4.152095702	0.00037908	0.041757081
cg01306069	-0.688073066	0.764299173	-4.152004136	0.000379167	0.041757081
cg20714968	-0.537601392	2.872194354	-4.151913881	0.000379252	0.041757081
cg08446512	-0.630501869	3.252941029	-4.151855176	0.000379308	0.041757081
cg24937275	-0.350882006	-2.434490256	-4.151842743	0.00037932	0.041757081
cg04217583	-0.461837019	3.659320952	-4.151791825	0.000379368	0.041757081
cg05566777	-0.386758107	-0.167674475	-4.151770453	0.000379388	0.041757081
cg02889469	-0.660855113	3.131179783	-4.151744945	0.000379412	0.041757081
cg13263079	-0.541376345	3.688898856	-4.15168898	0.000379466	0.041757081
cg06873805	-0.438059726	3.728056891	-4.15163412	0.000379518	0.041757081

cg20636756	-0.508238932	3.328045046	-4.151588864	0.000379561	0.041757081
cg18603679	-0.617203678	3.68024652	-4.151527328	0.000379619	0.041757081
cg12171165	-0.536784794	3.152643465	-4.15134538	0.000379792	0.041769595
cg16372086	-0.457146063	-0.024282869	-4.15113089	0.000379995	0.041772682
cg06596991	-0.548739741	-2.423551691	-4.151112297	0.000380013	0.041772682
cg13634760	-0.365527914	4.133883172	-4.151101692	0.000380023	0.041772682
cg02208176	-0.53845777	-3.11279164	-4.151067518	0.000380056	0.041772682
cg12676686	-0.462847423	0.84365047	-4.150942478	0.000380174	0.041776897
cg07292161	-0.495546799	3.830288759	-4.150891357	0.000380223	0.041776897
cg25503044	-0.451171895	-1.544988435	-4.150792161	0.000380317	0.041776897
cg01510688	-0.40258864	0.164232936	-4.150779004	0.00038033	0.041776897
cg12511749	-0.786000637	1.793168806	-4.150237826	0.000380845	0.041826976
cg25531090	-0.529438563	-0.747691026	-4.149758216	0.000381302	0.041855827
cg10316372	0.485909425	3.252848177	4.149734466	0.000381324	0.041855827
cg03883098	-0.452260399	-0.561686523	-4.149729871	0.000381329	0.041855827
cg24034459	-0.484280746	2.447670081	-4.149668588	0.000381387	0.041855827
cg05464822	-0.625949497	1.707653466	-4.149652105	0.000381403	0.041855827
cg26312017	-0.499544965	3.383431129	-4.149564294	0.000381487	0.041857345
cg21245909	-0.519174328	3.151465104	-4.149513688	0.000381535	0.041857345
cg00483582	-0.915644879	2.879095976	-4.14941405	0.00038163	0.041861291
cg14819352	-0.631979222	3.060938172	-4.149136298	0.000381895	0.041872687
cg15127832	0.541444195	3.707967191	4.149114877	0.000381916	0.041872687
cg12653348	-0.655629637	2.001227437	-4.149041078	0.000381986	0.041872687
cg23044403	-0.72988918	3.29513585	-4.149033345	0.000381993	0.041872687
cg00244111	-0.633492919	1.308488095	-4.148995619	0.000382029	0.041872687
cg08723742	-0.487619651	0.045713183	-4.148843823	0.000382174	0.041877822
cg08856463	-0.507034391	3.635943463	-4.148809106	0.000382208	0.041877822
cg27094531	-0.611170292	2.500382246	-4.148760955	0.000382254	0.041877822
cg21985296	-0.508049871	-0.405399615	-4.148532461	0.000382472	0.041883575
cg11606295	-0.668613634	3.441465042	-4.148507955	0.000382496	0.041883575
cg23078159	-0.597111554	1.856297579	-4.148503828	0.000382499	0.041883575
cg04217564	-0.47665004	2.878982912	-4.148458653	0.000382543	0.041883575
cg03264356	-0.414727006	4.032423874	-4.148292404	0.000382702	0.041891164
cg06024252	-0.596096499	2.280009242	-4.148262568	0.00038273	0.041891164
cg13818168	-0.817207467	2.253898926	-4.148131964	0.000382855	0.041898371
cg01085399	-0.72340267	2.711570148	-4.147819806	0.000383154	0.041921572
cg04852926	-0.605977118	3.729278086	-4.147786979	0.000383186	0.041921572
cg16044482	-0.413192647	0.332622728	-4.147643166	0.000383323	0.041926285
cg07072293	-0.440180918	3.878249467	-4.147618484	0.000383347	0.041926285
cg06519782	-0.445819078	3.814744947	-4.147380625	0.000383575	0.041943941
cg11206607	-0.553477873	3.330851471	-4.147326587	0.000383627	0.041943941
cg19904411	0.632908166	-4.121106664	4.14715987	0.000383787	0.041954952
cg24109955	-0.457328883	3.736695937	-4.147071029	0.000383872	0.041957796
cg11750768	-0.652601578	3.768733759	-4.146933723	0.000384004	0.041958057
cg19163398	-0.516897962	3.631299364	-4.146933326	0.000384004	0.041958057
cg12831960	-0.624229902	0.387575026	-4.146821835	0.000384111	0.041958057
cg23277985	-0.400167665	4.147459459	-4.146821755	0.000384111	0.041958057

cg17660041	-0.747488574	2.335072165	-4.146664419	0.000384263	0.041968093
cg17868542	-0.706006614	2.670915509	-4.146567712	0.000384355	0.04197177
cg15837235	-0.767871479	-1.75121242	-4.146378234	0.000384538	0.04197813
cg18355146	-0.449771815	1.843119314	-4.146275484	0.000384636	0.04197813
cg16669842	-0.595933747	2.904214367	-4.146256574	0.000384655	0.04197813
cg06517794	-0.553986394	3.76767465	-4.146245914	0.000384665	0.04197813
cg18009959	-0.598328684	1.087661963	-4.146124461	0.000384782	0.04197813
cg00484899	-0.66853411	1.791910941	-4.146103958	0.000384801	0.04197813
cg23821206	0.572858413	2.748775705	4.146075718	0.000384829	0.04197813
cg13974400	-0.516188076	4.177172629	-4.145951484	0.000384948	0.041983472
cg20181180	-0.523514189	3.419908245	-4.145857131	0.000385039	0.041983472
cg15609069	-0.669821862	2.206821181	-4.145751787	0.00038514	0.041983472
cg23491895	-0.579253528	3.186511163	-4.145716987	0.000385174	0.041983472
cg02725420	-0.781915745	1.900980306	-4.145716958	0.000385174	0.041983472
cg05981211	-0.776292207	2.883860111	-4.145562385	0.000385323	0.041991358
cg09619064	-0.824623851	-1.719320079	-4.145433422	0.000385447	0.041991358
cg08338739	-0.49198441	4.079207103	-4.145318736	0.000385557	0.041991358
cg10171170	-0.967773851	2.450832992	-4.145270367	0.000385604	0.041991358
cg14478731	-0.329472257	3.856480834	-4.14525879	0.000385615	0.041991358
cg21963629	-0.454405938	3.438907704	-4.145241372	0.000385632	0.041991358
cg05003554	-0.502308017	2.801218004	-4.14521124	0.000385661	0.041991358
cg20557603	-0.668676153	0.788890537	-4.145138106	0.000385732	0.041992581
cg05608754	-0.534711041	0.457383893	-4.145057315	0.00038581	0.04199461
cg05192138	-0.495063441	-0.81439429	-4.144827457	0.000386031	0.042006231
cg02670908	-0.592552349	3.263688091	-4.144807721	0.00038605	0.042006231
cg05207135	-0.378406185	3.776851378	-4.1447623	0.000386094	0.042006231
cg27090679	-0.561691653	2.346321711	-4.144679704	0.000386174	0.042008453
cg12641286	-0.577718922	1.389444457	-4.144566495	0.000386283	0.042013893
cg12930149	-0.595038726	-0.523505119	-4.144390542	0.000386453	0.042019034
cg11418308	-0.517296135	2.855486234	-4.144385412	0.000386458	0.042019034
cg16336436	-0.552344523	-0.32650743	-4.144333346	0.000386508	0.042019034
cg18362986	-0.564046937	3.545259203	-4.144220939	0.000386617	0.042024394
cg00356464	-0.491119628	-0.392391967	-4.143999171	0.000386831	0.042025329
cg15479699	-0.492242043	-1.239769934	-4.143953116	0.000386876	0.042025329
cg05987787	-0.596407195	1.740158937	-4.143929636	0.000386899	0.042025329
cg23613051	-0.434818772	-0.41052649	-4.143909895	0.000386918	0.042025329
cg15248672	-0.476498165	2.699960783	-4.143840718	0.000386985	0.042025329
cg07606842	-0.494098479	3.746829904	-4.143801933	0.000387022	0.042025329
cg04458547	-0.594102352	4.026420685	-4.143782617	0.000387041	0.042025329
cg20384939	-0.572982254	3.536407629	-4.143583067	0.000387234	0.042034368
cg09095223	-0.582882426	3.178968479	-4.143573987	0.000387243	0.042034368
cg12820481	-0.432554777	-1.354324288	-4.143439745	0.000387373	0.042042035
cg00866321	-0.552260172	3.689905802	-4.143314871	0.000387494	0.042048719
cg04907802	-0.57817826	0.436984276	-4.143242609	0.000387564	0.042049875
cg14583365	-0.483805708	3.612295751	-4.143019095	0.00038778	0.042061282
cg09642525	-0.336266056	-0.498647706	-4.143011591	0.000387788	0.042061282
cg18780243	-0.491591344	3.973683706	-4.142839133	0.000387955	0.042066462

cg02404197	-0.515683311	-0.329254328	-4.142781285	0.000388011	0.042066462
cg10866060	-0.928339752	-1.141924662	-4.142778634	0.000388014	0.042066462
cg10922442	-0.680184069	2.770734681	-4.142638384	0.00038815	0.042072536
cg05477480	-0.332498963	4.250401368	-4.142456982	0.000388326	0.042072536
cg15177986	-0.634095809	-1.269316886	-4.14237381	0.000388407	0.042072536
cg00723553	-0.520495024	0.064835807	-4.142364748	0.000388415	0.042072536
cg22186533	0.762802386	-4.997136326	4.142209375	0.000388566	0.042072536
cg15086872	-0.803308481	2.594086103	-4.142198349	0.000388577	0.042072536
cg20202792	-0.707800777	3.311439353	-4.14210884	0.000388664	0.042072536
cg10093761	-0.424910089	4.30283658	-4.142056506	0.000388715	0.042072536
cg01139101	-0.540251467	3.556027096	-4.142053083	0.000388718	0.042072536
cg01087690	-0.725836137	-1.516229691	-4.14204615	0.000388725	0.042072536
cg16663033	-0.748059554	-2.227797352	-4.141887039	0.000388879	0.042072536
cg01057132	-0.478623227	-0.613780608	-4.141874948	0.000388891	0.042072536
cg20097219	-0.426272058	-0.668776947	-4.141781596	0.000388982	0.042072536
cg22268309	-0.544406786	-0.369835101	-4.141746742	0.000389016	0.042072536
cg14956284	-0.473103536	3.53381236	-4.14169318	0.000389068	0.042072536
cg18520696	-0.497498922	3.484930814	-4.14166275	0.000389098	0.042072536
cg26172050	-0.505628669	-0.492570329	-4.141605603	0.000389153	0.042072536
cg08857436	-0.484707086	3.680849919	-4.141533711	0.000389223	0.042072536
cg14060279	-0.71314324	2.039148714	-4.141531708	0.000389225	0.042072536
cg12283086	-0.665295989	2.181289622	-4.141366998	0.000389385	0.042072536
cg18151425	-0.523232512	3.467196981	-4.141322686	0.000389428	0.042072536
cg25714963	-0.826957515	1.725438087	-4.141319578	0.000389431	0.042072536
cg15127607	-0.363664661	3.827577642	-4.141315278	0.000389436	0.042072536
cg17491589	-0.537491402	3.503238952	-4.141151754	0.000389595	0.042073563
cg12612154	-0.523862541	4.168844257	-4.140982261	0.00038976	0.042073563
cg04258944	-0.464414581	-1.046282872	-4.140958791	0.000389783	0.042073563
cg26661319	-0.563080626	3.134546656	-4.14090667	0.000389834	0.042073563
cg02374917	-0.63670953	-1.087142308	-4.140900792	0.000389839	0.042073563
cg18198065	-0.484906301	-1.302855179	-4.140861528	0.000389878	0.042073563
cg11392655	-0.45645037	-0.503263874	-4.140827374	0.000389911	0.042073563
cg23780731	-0.512057221	-0.584239824	-4.14081775	0.00038992	0.042073563
cg09459202	-0.425621911	2.235751892	-4.140635292	0.000390098	0.042086347
cg00740721	-0.533314973	2.915414246	-4.140527155	0.000390204	0.042091315
cg27126251	-0.410644925	3.727078751	-4.140443796	0.000390285	0.042091706
cg01466237	-0.867881582	3.568960222	-4.140365366	0.000390362	0.042091706
cg20009671	0.560923965	-4.670621197	4.140340743	0.000390386	0.042091706
cg21130374	0.514283318	2.180831923	4.140230892	0.000390493	0.042095638
cg12491257	-0.501116649	-0.138338632	-4.140181617	0.000390541	0.042095638
cg24511950	-0.815796739	2.307849173	-4.140050254	0.000390669	0.042098814
cg24741685	-0.363084117	-1.315068114	-4.140029724	0.000390689	0.042098814
cg01688202	-0.79392	-1.138676534	-4.139935338	0.000390781	0.042102344
cg04426028	-0.506080206	3.389725197	-4.139843871	0.000390871	0.042105568
cg19961913	-0.505627101	0.033638094	-4.139491591	0.000391215	0.042128047
cg12365951	0.320227601	-0.112459606	4.139453178	0.000391253	0.042128047
cg22021234	-0.536921729	3.561934423	-4.139447902	0.000391258	0.042128047

cg11682700	-0.633941484	-0.366892106	-4.139329965	0.000391373	0.042128071
cg13751683	-0.600653585	1.14523733	-4.139326081	0.000391377	0.042128071
cg26743898	-0.474263512	3.34774096	-4.139234965	0.000391466	0.042131266
cg21943268	-0.480823953	-1.912937551	-4.139145632	0.000391554	0.042134274
cg02032984	-0.562183987	3.21109788	-4.138815581	0.000391877	0.04214728
cg11580118	-0.496076418	-0.28758666	-4.138770425	0.000391921	0.04214728
cg14011076	-0.505691506	3.576595565	-4.138767871	0.000391924	0.04214728
cg03730519	-0.74544087	2.770360924	-4.138717343	0.000391973	0.04214728
cg23533270	-0.575769515	3.500368194	-4.138710524	0.00039198	0.04214728
cg17355742	-0.682844709	-1.679455684	-4.138611235	0.000392077	0.04214728
cg19922558	-0.470082618	2.342024589	-4.138504078	0.000392182	0.04214728
cg03398156	-0.506266836	-0.873561215	-4.138478388	0.000392207	0.04214728
cg17711067	-0.540471245	-1.413315061	-4.138475631	0.00039221	0.04214728
cg00939357	-0.484660085	-2.554992577	-4.138350011	0.000392333	0.042148531
cg08324750	-0.500066621	-0.582265005	-4.13819465	0.000392486	0.042148531
cg04972459	-0.402291064	-0.476267327	-4.138053865	0.000392624	0.042148531
cg17724789	-0.431024043	3.485607742	-4.137891389	0.000392783	0.042148531
cg06544693	-0.45527133	-0.109504924	-4.137836554	0.000392837	0.042148531
cg11634214	-0.456566975	3.216182074	-4.13773315	0.000392939	0.042148531
cg16171480	-0.612319617	3.246270815	-4.137620788	0.000393049	0.042148531
cg05716202	-0.444832296	3.477803706	-4.137609913	0.00039306	0.042148531
cg12064202	-0.581350301	-0.512531351	-4.137516142	0.000393152	0.042148531
cg02411244	-0.774613437	0.886416338	-4.137459139	0.000393208	0.042148531
cg24874191	0.578412408	2.267285849	4.137452929	0.000393214	0.042148531
cg24578615	-0.431433157	1.933177525	-4.137446132	0.000393221	0.042148531
cg15577401	-0.522125323	1.929392706	-4.137368904	0.000393297	0.042148531
cg25140773	-0.516551582	3.149340638	-4.137351556	0.000393314	0.042148531
cg26066413	-0.677110755	2.833686003	-4.137155587	0.000393506	0.042148531
cg07514797	-0.58934831	2.114487857	-4.137138734	0.000393523	0.042148531
cg06328127	-0.453437844	3.195320023	-4.137100271	0.000393561	0.042148531
cg09779837	-0.704790709	3.09570826	-4.136947225	0.000393711	0.042148531
cg24692147	-0.51688576	3.308154	-4.136910088	0.000393748	0.042148531
cg04366343	-0.524047957	3.237941011	-4.13673549	0.00039392	0.042148531
cg04532638	-0.457697204	0.086821916	-4.136732653	0.000393923	0.042148531
cg06084585	-0.431233698	-1.223094707	-4.136554276	0.000394098	0.042148531
cg01318459	-0.46040184	3.793066219	-4.13654408	0.000394108	0.042148531
cg02869156	-0.499674678	3.009371012	-4.136522445	0.00039413	0.042148531
cg09841362	-0.525131568	0.376618589	-4.136476081	0.000394175	0.042148531
cg26684581	-0.349611782	4.047724661	-4.136416001	0.000394234	0.042148531
cg09039874	-0.683840658	0.949082983	-4.13636665	0.000394283	0.042148531
cg19938167	-0.717669132	0.61091924	-4.136320174	0.000394329	0.042148531
cg15340018	-0.574988289	3.753269262	-4.136295852	0.000394353	0.042148531
cg11631011	-0.465090928	3.370074335	-4.136231209	0.000394417	0.042148531
cg10735621	-0.573859576	0.538085132	-4.136200963	0.000394446	0.042148531
cg00866289	-0.471588902	3.361723473	-4.136195692	0.000394452	0.042148531
cg03469437	-0.530612139	3.312325656	-4.13611779	0.000394528	0.042148531
cg16003494	-0.352065131	4.198745738	-4.136078055	0.000394568	0.042148531

cg00342233	-0.798981066	2.519661743	-4.135973638	0.000394671	0.042148531
cg21939447	-0.619998406	2.995435909	-4.135908895	0.000394735	0.042148531
cg06376357	-0.864559284	1.789087147	-4.135715806	0.000394925	0.042148531
cg20024250	-0.409555003	3.82906016	-4.135707233	0.000394934	0.042148531
cg10111305	-0.375924164	3.748601724	-4.135695723	0.000394945	0.042148531
cg05062902	-0.800285192	2.078563775	-4.135579435	0.00039506	0.042148531
cg07341778	-0.388238652	-0.892134155	-4.135579318	0.00039506	0.042148531
cg00387090	0.543462992	3.012967031	4.13557382	0.000395065	0.042148531
cg09451457	-0.490432278	-1.984188999	-4.135528157	0.00039511	0.042148531
cg13780053	-0.333191904	-0.603960599	-4.135525104	0.000395113	0.042148531
cg02583183	-0.52968536	2.893480773	-4.135488969	0.000395149	0.042148531
cg00260499	-0.515351775	-1.064161482	-4.135470177	0.000395168	0.042148531
cg09501518	-0.719625195	4.428134986	-4.135436663	0.000395201	0.042148531
cg10504392	-0.680565059	-2.417721233	-4.13541832	0.000395219	0.042148531
cg15397033	-0.485532152	3.472504308	-4.135287976	0.000395348	0.042148531
cg23182633	-0.535661921	-0.714070438	-4.13526423	0.000395371	0.042148531
cg02098227	-0.392818941	0.128952655	-4.135241225	0.000395394	0.042148531
cg20826755	-0.668909231	-0.478226464	-4.135178405	0.000395456	0.042148531
cg16086945	-0.488672049	3.636024867	-4.135173963	0.00039546	0.042148531
cg13676008	0.584497123	-3.593345637	4.135056842	0.000395576	0.042148531
cg19318707	-0.852740838	1.754106673	-4.135022688	0.00039561	0.042148531
cg27298566	-0.633362064	3.14846999	-4.135001119	0.000395631	0.042148531
cg01005439	-0.50998817	-1.630832824	-4.134977994	0.000395654	0.042148531
cg26632897	-0.475107496	-1.078361574	-4.134959308	0.000395673	0.042148531
cg09922745	-0.643429139	2.414450687	-4.13487166	0.000395759	0.042151427
cg10852664	-0.434255478	-0.495489817	-4.134761155	0.000395869	0.042156734
cg07635303	-0.38432488	0.208947064	-4.134667303	0.000395961	0.042158574
cg07593004	0.559986919	2.938060531	4.134567703	0.00039606	0.042158574
cg10101533	-0.609935211	1.476892796	-4.134563305	0.000396064	0.042158574
cg25223716	-0.700946111	3.435059728	-4.134401781	0.000396224	0.042166149
cg01291285	-0.547324053	3.751246508	-4.134371223	0.000396255	0.042166149
cg05055322	-0.524817413	0.593082306	-4.134211023	0.000396413	0.042176701
cg12770187	-0.607163656	2.111872509	-4.134111403	0.000396512	0.04218087
cg06511330	-0.395424352	-0.889544796	-4.133861282	0.00039676	0.042193203
cg12429457	-0.472824114	-0.747524612	-4.13385005	0.000396771	0.042193203
cg25841553	-0.569328172	3.399979585	-4.133744852	0.000396876	0.042193203
cg00932250	-0.36853658	-0.45872485	-4.133704625	0.000396915	0.042193203
cg03422137	-0.926743747	1.390668296	-4.133612021	0.000397007	0.042193203
cg27307421	-0.558155299	3.559694549	-4.133548874	0.00039707	0.042193203
cg21766191	0.558175704	2.270058817	4.133457639	0.000397161	0.042193203
cg26154808	-0.469100276	-1.751693332	-4.133421842	0.000397196	0.042193203
cg00002979	-0.790343546	2.456574458	-4.133361833	0.000397256	0.042193203
cg00632379	-0.718922397	0.662744215	-4.133222106	0.000397394	0.042193203
cg20860792	-0.517366767	3.149192151	-4.133184652	0.000397432	0.042193203
cg12056547	-0.449298425	2.22825937	-4.133168573	0.000397448	0.042193203
cg08575688	-0.595528381	2.803735119	-4.133113711	0.000397502	0.042193203
cg06069280	-0.678359745	1.185452165	-4.133111865	0.000397504	0.042193203

cg14672084	-0.724600233	-0.640797109	-4.133094268	0.000397521	0.042193203
cg20847165	-0.801594236	2.38899559	-4.132974484	0.00039764	0.042199515
cg08815036	-0.443952695	4.198786448	-4.132876938	0.000397737	0.042203482
cg17096881	-0.453167752	0.386258796	-4.132616488	0.000397996	0.042224641
cg06989803	-0.539670154	3.676665452	-4.132498272	0.000398114	0.042225904
cg24120602	-0.441744816	0.814572194	-4.132461806	0.00039815	0.042225904
cg26461905	-0.471033856	3.9676898	-4.132424811	0.000398187	0.042225904
cg21321372	0.612727414	2.013446373	4.132317977	0.000398294	0.042230859
cg05347723	-0.563635787	3.187842165	-4.132134718	0.000398476	0.042237041
cg06996186	-0.671994606	3.122184979	-4.132113057	0.000398498	0.042237041
cg10208640	-0.576419793	3.216793494	-4.132079813	0.000398531	0.042237041
cg24132146	-0.64447217	1.930835144	-4.131962508	0.000398648	0.042237109
cg07138764	-0.496729869	3.942000124	-4.131959475	0.000398651	0.042237109
cg07107046	-0.799033295	2.757990297	-4.131889179	0.000398721	0.042238213
cg09918110	-0.593639659	-1.370170928	-4.13179819	0.000398811	0.042241503
cg14120436	0.734655361	2.061617033	4.131718101	0.000398891	0.042243644
cg05556038	-0.471408042	4.206374441	-4.131517274	0.000399091	0.042248433
cg26892308	-0.371738276	-0.298690295	-4.131442689	0.000399166	0.042248433
cg01912692	-0.528221625	3.755266323	-4.131400162	0.000399208	0.042248433
cg05105889	-0.497496244	3.600541543	-4.131387691	0.000399221	0.042248433
cg21620078	-0.74167055	1.598237166	-4.131235791	0.000399372	0.042248433
cg09221363	0.6366624	2.692178333	4.131200883	0.000399407	0.042248433
cg09994301	-0.555200679	3.304338824	-4.131147584	0.00039946	0.042248433
cg18504013	-0.413635729	-0.666946076	-4.131099454	0.000399508	0.042248433
cg00280394	-0.420431435	3.959632946	-4.131099275	0.000399509	0.042248433
cg27368181	-0.525564648	3.209138406	-4.131075073	0.000399533	0.042248433
cg19298400	-0.557737665	-0.716680283	-4.130873219	0.000399734	0.042263445
cg15441351	-0.657633336	2.989959551	-4.130748149	0.000399859	0.04227035
cg22553703	-0.365436009	-0.157856285	-4.130584757	0.000400023	0.042281307
cg25513499	-0.809257821	2.610879216	-4.130491541	0.000400116	0.042282996
cg04107175	-0.462500792	-1.058995262	-4.1304494	0.000400158	0.042282996
cg12222932	-0.356131947	-0.715967747	-4.130387454	0.00040022	0.042283236
cg05723953	-0.480750912	3.983137027	-4.130238299	0.000400369	0.042292694
cg22906451	0.620216855	2.325486618	4.129925303	0.000400683	0.042310259
cg08972835	-0.366840957	0.129858068	-4.129905511	0.000400702	0.042310259
cg16721177	-0.47681116	-1.234928912	-4.129893226	0.000400715	0.042310259
cg19702675	-0.562208447	-1.624534101	-4.129563221	0.000401045	0.042319357
cg10093768	-0.804842967	2.554875139	-4.12956262	0.000401046	0.042319357
cg26671936	-0.547721225	3.517763514	-4.129434373	0.000401175	0.042319357
cg22990850	0.566798514	-1.385104454	4.129339633	0.00040127	0.042319357
cg24597564	-0.457015714	3.407016668	-4.129298241	0.000401311	0.042319357
cg04266607	-0.751201013	-0.522560148	-4.129259774	0.00040135	0.042319357
cg07353077	-0.57154121	3.150328567	-4.129246094	0.000401363	0.042319357
cg26621805	-0.665072526	2.972461316	-4.129158159	0.000401452	0.042319357
cg12229979	-0.647137241	-1.134725657	-4.129059225	0.000401551	0.042319357
cg13517781	-0.417767423	3.581050797	-4.129019906	0.00040159	0.042319357
cg21576107	-0.866963902	2.262164812	-4.129019083	0.000401591	0.042319357

cg12759516	-0.488073104	2.844590573	-4.128945592	0.000401665	0.042319357
cg16100721	-0.388037812	-0.341733178	-4.128900288	0.00040171	0.042319357
cg21613620	-0.506739971	3.023064794	-4.12889728	0.000401713	0.042319357
cg17940367	-0.458738895	-0.003884122	-4.128820648	0.00040179	0.042319357
cg27420255	-0.710069883	2.586946684	-4.128814813	0.000401796	0.042319357
cg07262682	-0.464731583	3.59420874	-4.12879468	0.000401817	0.042319357
cg20232747	0.624848458	2.672439815	4.12872829	0.000401883	0.042320088
cg17107289	-0.699046212	2.546537595	-4.128454896	0.000402158	0.04233583
cg06419219	0.581802549	1.225526422	4.128342949	0.00040227	0.04233583
cg25438652	-0.594903525	3.309750714	-4.128216924	0.000402397	0.04233583
cg21514373	-0.524561057	-1.900935646	-4.128191035	0.000402423	0.04233583
cg18304458	-0.554474724	2.290100547	-4.128175296	0.000402439	0.04233583
cg12381675	-0.753163346	2.457987612	-4.128160142	0.000402454	0.04233583
cg15847240	-0.696555801	2.190709548	-4.128068636	0.000402546	0.04233583
cg13818192	-0.501161941	3.34145408	-4.128053239	0.000402562	0.04233583
cg14005037	-0.553876531	2.983040138	-4.128044554	0.000402571	0.04233583
cg00066512	-0.482496653	4.075051491	-4.127927441	0.000402688	0.042340781
cg04833985	-0.576155323	2.825892115	-4.127878993	0.000402737	0.042340781
cg09360099	-0.573728754	3.658290942	-4.127779144	0.000402838	0.042341797
cg06492744	-0.599096675	-2.482462183	-4.127705649	0.000402912	0.042341797
cg10095082	-0.513771844	2.389126749	-4.127691289	0.000402926	0.042341797
cg17296678	4.215176852	1.090570544	4.127461508	0.000403158	0.04234937
cg02770728	-1.022728079	1.990066475	-4.127447496	0.000403172	0.04234937
cg11146884	-0.730750592	-2.076283074	-4.127370924	0.000403249	0.04234937
cg12551908	5.151858819	1.546569595	4.127347049	0.000403273	0.04234937
cg21025352	-0.650171269	2.306780603	-4.127323073	0.000403297	0.04234937
cg09359521	-0.563654469	-1.226838942	-4.127195468	0.000403426	0.042354821
cg14669747	-0.4692159	-0.346053743	-4.127142055	0.00040348	0.042354821
cg17110599	-0.569667672	2.930265679	-4.126920618	0.000403703	0.042354821
cg21902739	-0.734103395	3.192906531	-4.126868885	0.000403755	0.042354821
cg01840281	-0.617878037	-0.089575984	-4.12662066	0.000404006	0.042354821
cg01072388	-0.356347704	-1.073393967	-4.126531418	0.000404096	0.042354821
cg14843013	-0.532288216	-0.086765594	-4.126486503	0.000404141	0.042354821
cg01291592	-0.665746093	3.208743643	-4.126412209	0.000404216	0.042354821
cg12201681	-0.626196571	1.006821062	-4.126403018	0.000404226	0.042354821
cg03646234	-0.568800133	-3.382706521	-4.126381855	0.000404247	0.042354821
cg14917721	0.425548962	1.845216352	4.126312535	0.000404317	0.042354821
cg26094549	-0.496902492	3.746125463	-4.126288831	0.000404341	0.042354821
cg20431441	-0.54862408	2.493710663	-4.126282639	0.000404347	0.042354821
cg11755269	-0.837181421	1.808014143	-4.126277182	0.000404353	0.042354821
cg00362062	-0.517434154	2.067474784	-4.126271606	0.000404358	0.042354821
cg23518214	-0.47831011	3.724568044	-4.12626213	0.000404368	0.042354821
cg11948328	-0.568605447	-2.654391361	-4.126210004	0.000404421	0.042354821
cg22803214	-0.810386147	2.613227406	-4.126205399	0.000404425	0.042354821
cg03469476	-0.516666765	3.336711356	-4.126073641	0.000404559	0.042362508
cg19261968	-0.565536695	3.180543864	-4.125862404	0.000404772	0.04237684
cg03610550	-0.419797293	3.779919041	-4.125796302	0.000404839	0.04237684

cg26522423	-0.555148001	3.182314478	-4.125632764	0.000405005	0.04237684
cg06220761	-0.817520785	1.725558821	-4.125516927	0.000405122	0.04237684
cg02234375	-0.438282416	0.362201358	-4.125502571	0.000405136	0.04237684
cg11974199	-0.613343413	2.440467521	-4.125496101	0.000405143	0.04237684
cg25654517	-0.600402986	0.354337992	-4.125419697	0.00040522	0.04237684
cg11824099	-0.730497029	2.540169262	-4.125394303	0.000405246	0.04237684
cg15115224	-0.66619166	2.762041999	-4.125282344	0.000405359	0.04237684
cg27415655	-0.544602614	-0.906590514	-4.125238769	0.000405404	0.04237684
cg01021743	-0.471319432	3.372270735	-4.125037378	0.000405608	0.04237684
cg11955290	-0.583334553	-0.287647487	-4.125035567	0.000405609	0.04237684
cg08092966	-0.639650155	-0.33334196	-4.125002378	0.000405643	0.04237684
cg10318484	-0.546162841	2.996357685	-4.124925999	0.000405721	0.04237684
cg18745642	-0.5303099	-0.196912997	-4.124923749	0.000405723	0.04237684
cg12658495	-0.492027044	-0.748285767	-4.124899936	0.000405747	0.04237684
cg04058837	-0.523630862	1.408235299	-4.124858699	0.000405789	0.04237684
cg01081456	0.622499226	2.583754648	4.124826523	0.000405821	0.04237684
cg07502417	-0.433335114	-1.420395676	-4.124783148	0.000405865	0.04237684
cg26212586	-0.482040673	3.068197888	-4.124630569	0.00040602	0.04237684
cg14287557	-0.566869955	-2.324340755	-4.124573852	0.000406078	0.04237684
cg04567669	-0.377191437	-0.660778861	-4.124553197	0.000406099	0.04237684
cg09993711	-0.94313665	2.382265838	-4.124530844	0.000406121	0.04237684
cg01512876	-0.431707418	3.550325457	-4.124477096	0.000406176	0.04237684
cg17186139	-0.59509429	2.677453906	-4.124462375	0.000406191	0.04237684
cg18736800	-0.444918547	3.609008475	-4.124369938	0.000406285	0.04238039
cg10773619	-0.503377923	3.74175972	-4.124284826	0.000406371	0.042380524
cg05400732	-0.610395069	-0.2105119	-4.124216264	0.000406441	0.042380524
cg05307536	-0.888034035	1.738088358	-4.124147741	0.00040651	0.042380524
cg18213786	-0.672292262	-2.447479475	-4.124097166	0.000406562	0.042380524
cg23340218	-0.419107552	-1.204240737	-4.124074178	0.000406585	0.042380524
cg17207065	-0.684385883	2.983072255	-4.124004125	0.000406656	0.042381708
cg15465569	-0.492502569	3.709286571	-4.12384026	0.000406823	0.042392831
cg21465372	-0.493939978	4.14699505	-4.123733971	0.000406931	0.042397857
cg10313121	-0.435168076	4.298302551	-4.123616061	0.000407051	0.042404116
cg14110193	-0.493936886	3.427993429	-4.123513181	0.000407156	0.042408783
cg08581297	-0.577384689	2.637504202	-4.123351629	0.00040732	0.042419672
cg00911593	-0.455511917	3.557682162	-4.123281184	0.000407392	0.042420904
cg13520631	-0.61486854	-0.317493264	-4.123032969	0.000407645	0.042440989
cg21556924	-0.44564683	4.475641784	-4.122948906	0.00040773	0.042442336
cg20104235	-0.504082231	0.474790436	-4.122892181	0.000407788	0.042442336
cg17342772	-0.638546386	3.575800464	-4.122824877	0.000407857	0.042442336
cg08993690	-0.606742087	-0.339534623	-4.122698376	0.000407986	0.042442336
cg11255484	-0.722374269	2.481055408	-4.122653879	0.000408031	0.042442336
cg21722370	-0.444485447	3.994378155	-4.122629358	0.000408056	0.042442336
cg25031476	0.452603575	2.53022347	4.122521657	0.000408166	0.042442336
cg02782052	-0.4694763	3.991281076	-4.122459882	0.000408229	0.042442336
cg02123534	-0.474519602	0.996026733	-4.122448385	0.000408241	0.042442336
cg06599983	-0.577368054	2.336580668	-4.122432605	0.000408257	0.042442336

cg15566571	-0.533873591	3.129002862	-4.12236923	0.000408321	0.04244283
cg25259755	-0.453202148	1.854059023	-4.122137866	0.000408557	0.042459986
cg21580043	-0.546503606	0.274244387	-4.122090113	0.000408606	0.042459986
cg17605713	0.647092346	3.089726305	4.121987643	0.000408711	0.042464631
cg13959830	-0.457400927	3.454071034	-4.121889731	0.000408811	0.042468794
cg03133777	-0.383488966	-1.687580922	-4.121648414	0.000409058	0.042486134
cg08866868	-0.430153828	-0.035631184	-4.121595916	0.000409111	0.042486134
cg21573231	-0.768384951	3.073440489	-4.121550386	0.000409158	0.042486134
cg08745685	-0.49257982	3.994278283	-4.12137205	0.00040934	0.042498844
cg00983158	-0.489802208	3.82317083	-4.121116192	0.000409602	0.042519036
cg18735920	0.642120428	2.984302173	4.121064703	0.000409655	0.042519036
cg04273674	-0.469722632	0.496720906	-4.120926339	0.000409796	0.042527509
cg06647446	-0.691225701	2.834691614	-4.1208487	0.000409876	0.042527519
cg01820107	-0.495114891	-0.241306388	-4.120809019	0.000409917	0.042527519
cg10966629	-0.429946044	4.054055218	-4.120699266	0.000410029	0.042532956
cg16743226	-0.402151298	3.632708396	-4.120560408	0.000410171	0.042541489
cg12072024	-0.703696571	1.523357477	-4.120449031	0.000410285	0.042547102
cg22132013	-0.538532501	3.698616419	-4.120280867	0.000410458	0.042549125
cg09874796	-0.412023761	0.112079166	-4.120245666	0.000410494	0.042549125
cg00427847	-0.453030072	3.969843179	-4.120213668	0.000410527	0.042549125
cg19960991	-0.609724929	3.197036815	-4.120085194	0.000410659	0.042549125
cg11465609	-0.422167583	4.340002518	-4.120076182	0.000410668	0.042549125
cg20876170	0.647568002	2.524378524	4.120041714	0.000410703	0.042549125
cg00213082	-0.644910379	-2.665184244	-4.119750711	0.000411002	0.042549125
cg00022510	0.576494922	3.415041543	4.119658841	0.000411096	0.042549125
cg04941054	-0.50055834	-2.718306873	-4.119528849	0.00041123	0.042549125
cg04865726	-0.689618906	-3.183567872	-4.119412146	0.00041135	0.042549125
cg04249612	-0.524476499	3.951593584	-4.119349774	0.000411414	0.042549125
cg11104667	-0.403496338	3.236448474	-4.119344667	0.000411419	0.042549125
cg25350798	-0.706313656	2.308996227	-4.119334845	0.000411429	0.042549125
cg14920328	-0.430682812	2.932522499	-4.119297264	0.000411468	0.042549125
cg04693634	-0.527242974	3.612795171	-4.119265557	0.000411501	0.042549125
cg25479742	0.856228407	-2.294988457	4.119172957	0.000411596	0.042549125
cg03910871	-0.480827071	4.268958758	-4.11916826	0.000411601	0.042549125
cg09160330	-0.624725195	2.49112433	-4.11916724	0.000411602	0.042549125
cg01095395	-0.546968282	3.462129084	-4.119116746	0.000411654	0.042549125
cg04320377	-0.669440905	3.059141474	-4.119045305	0.000411727	0.042549125
cg01762919	-0.43237424	4.182917398	-4.119037174	0.000411736	0.042549125
cg10175712	-0.492970966	3.50146715	-4.119020298	0.000411753	0.042549125
cg05506809	-0.454353076	3.330261594	-4.118973815	0.000411801	0.042549125
cg24937356	-0.464950682	3.678303271	-4.118959485	0.000411816	0.042549125
cg19186275	-0.509100741	3.953744777	-4.118921917	0.000411854	0.042549125
cg12410824	-0.473324705	3.677869055	-4.118909823	0.000411867	0.042549125
cg20216309	-0.45480664	-1.243357903	-4.118808488	0.000411971	0.042552544
cg23568361	0.536579954	2.986975508	4.118745956	0.000412035	0.042552544
cg11145160	-0.612780476	-3.23743903	-4.118649799	0.000412134	0.042552544
cg08034991	-0.504385433	3.44912603	-4.118547648	0.00041224	0.042552544

cg26275419	-0.794431967	1.857347712	-4.118544454	0.000412243	0.042552544
cg16300329	-0.33026827	0.404482404	-4.118527674	0.00041226	0.042552544
cg03208889	-0.629341125	3.266418594	-4.118224901	0.000412572	0.042566807
cg11178985	-0.631712692	-1.798264964	-4.118073823	0.000412728	0.042566807
cg13449430	-0.664985009	-1.824325587	-4.117978684	0.000412826	0.042566807
cg04128222	-0.522089796	0.322835945	-4.11795554	0.00041285	0.042566807
cg25304867	-0.450406381	0.29292028	-4.117896361	0.000412911	0.042566807
cg08917980	-0.502345717	3.789665975	-4.117894732	0.000412913	0.042566807
cg13420393	-0.436380207	-0.287708084	-4.117791564	0.000413019	0.042566807
cg06452993	-0.506555	3.736627297	-4.117790092	0.000413021	0.042566807
cg13003350	-0.511822411	-0.457128365	-4.117782133	0.000413029	0.042566807
cg12927498	-0.771721349	-2.830899196	-4.117754188	0.000413058	0.042566807
cg18220699	-0.467195617	3.829394017	-4.11775269	0.000413059	0.042566807
cg10714061	-0.491195065	-0.061611857	-4.117637893	0.000413178	0.042572827
cg03838156	-0.42377352	-0.934435873	-4.117511043	0.000413309	0.04257557
cg06298666	-0.386336394	3.111607211	-4.117495717	0.000413325	0.04257557
cg00296011	-0.450634258	1.139364833	-4.117405841	0.000413418	0.042578941
cg23012243	-0.726732041	-2.949965696	-4.117081167	0.000413753	0.042591719
cg07442260	-0.687564645	1.6612114	-4.117065285	0.00041377	0.042591719
cg07226746	-0.553871194	2.545169743	-4.11705381	0.000413781	0.042591719
cg17987646	-0.369146731	3.881242963	-4.116931323	0.000413908	0.042591719
cg14383403	0.66399806	1.468275372	4.116910087	0.00041393	0.042591719
cg02757223	-0.904944442	1.956469725	-4.11690656	0.000413934	0.042591719
cg16829732	-0.595990035	3.097098021	-4.116878709	0.000413962	0.042591719
cg04120969	-0.510275123	2.967736714	-4.116781627	0.000414063	0.042591858
cg07161381	-0.561026738	3.299008261	-4.116662009	0.000414187	0.042591858
cg11074301	-0.481484825	-0.49219842	-4.116642659	0.000414207	0.042591858
cg10493186	-0.368032358	-0.829860652	-4.116572195	0.00041428	0.042591858
cg27662750	-0.441268552	4.132019728	-4.116532667	0.000414321	0.042591858
cg09795160	-0.457431129	-0.798873459	-4.116528817	0.000414325	0.042591858
cg26072483	-0.646632269	-0.127505104	-4.116382865	0.000414476	0.042597216
cg02583132	-0.928092317	-0.302136576	-4.116316272	0.000414545	0.042597216
cg27191700	-0.582221433	3.723073191	-4.116304291	0.000414557	0.042597216
cg19633581	-0.461888713	4.166022252	-4.116236745	0.000414627	0.042598228
cg22226821	0.552133197	1.388728783	4.116129007	0.000414739	0.042603519
cg25828097	-0.88852375	2.039752747	-4.115962411	0.000414911	0.04261199
cg10453803	-0.36130613	-1.225963978	-4.115931041	0.000414944	0.04261199
cg21853067	-0.486230082	-0.921786039	-4.115803007	0.000415077	0.04261199
cg15450246	-0.522808378	3.652843152	-4.115800777	0.000415079	0.04261199
cg26077359	-0.49358529	3.65012348	-4.115759399	0.000415122	0.04261199
cg22902342	-0.44247364	3.089968223	-4.115518156	0.000415372	0.042619508
cg27079346	-0.529742998	3.404165605	-4.115438609	0.000415455	0.042619508
cg10679152	-0.412354692	-0.628492689	-4.115402617	0.000415492	0.042619508
cg18232313	0.98848566	3.149112028	4.115341084	0.000415556	0.042619508
cg11121337	-0.695031858	3.140634739	-4.115265743	0.000415634	0.042619508
cg10246038	-0.749099137	2.496587376	-4.115255446	0.000415645	0.042619508
cg06676271	-0.664898732	2.839192871	-4.115138099	0.000415767	0.042619508

cg02542806	-0.496055653	3.475515803	-4.115085319	0.000415822	0.042619508
cg09992387	-0.475916628	-0.082989578	-4.115047017	0.000415862	0.042619508
cg03370998	-0.918884055	-0.015203452	-4.115031037	0.000415878	0.042619508
cg23691040	-0.57856023	2.858919901	-4.115018799	0.000415891	0.042619508
cg08811663	-0.584914878	3.089904535	-4.114781315	0.000416138	0.042619508
cg27324308	-0.602717981	-2.271918326	-4.114775958	0.000416143	0.042619508
cg16112545	-0.3909682	3.517750672	-4.114771199	0.000416148	0.042619508
cg24428529	-0.47965347	0.344012768	-4.114536313	0.000416393	0.042619508
cg22756142	-0.691829424	2.887157577	-4.114534801	0.000416394	0.042619508
cg08474576	-0.495638618	3.247541034	-4.114507682	0.000416422	0.042619508
cg08025318	-0.534387311	-2.664713179	-4.11436224	0.000416574	0.042619508
cg04594619	-0.702058444	-1.972789289	-4.11434005	0.000416597	0.042619508
cg09455542	-0.481066344	3.288113681	-4.114242484	0.000416698	0.042619508
cg25023829	-0.430811105	3.869571958	-4.114218044	0.000416724	0.042619508
cg21273746	-0.708996404	3.976002628	-4.114206417	0.000416736	0.042619508
cg20422212	-0.483005571	3.563267123	-4.114105374	0.000416841	0.042619508
cg11217193	-0.49429111	0.461290979	-4.114069739	0.000416878	0.042619508
cg02810826	-0.488801687	2.888037085	-4.114055318	0.000416893	0.042619508
cg26246536	-0.947440746	1.592457022	-4.1140253	0.000416925	0.042619508
cg22979140	-0.585331012	3.306317679	-4.113941013	0.000417012	0.042619508
cg26801159	-0.623387768	4.069812723	-4.1139129	0.000417042	0.042619508
cg02696067	-0.854502122	-1.007605662	-4.113876954	0.000417079	0.042619508
cg06543730	-0.606207588	-0.264350793	-4.113858925	0.000417098	0.042619508
cg16062094	-0.63512903	1.308200185	-4.113830105	0.000417128	0.042619508
cg07148634	-0.471064778	3.327141951	-4.113793203	0.000417167	0.042619508
cg06962436	-0.618330295	-0.719773061	-4.113779685	0.000417181	0.042619508
cg12650829	-0.504268627	3.795155545	-4.11372034	0.000417243	0.042619682
cg12382372	-0.557383427	3.02261959	-4.113593305	0.000417375	0.042627066
cg13408795	-0.483050765	-1.503256526	-4.113405685	0.000417571	0.042635614
cg04032578	-0.480148815	-2.798921925	-4.113313046	0.000417667	0.042635614
cg11338734	-0.798894995	1.996363892	-4.11323878	0.000417745	0.042635614
cg12543807	-0.624252487	3.29236004	-4.11308524	0.000417905	0.042635614
cg15392819	-0.388542594	-0.025017305	-4.11294603	0.000418051	0.042635614
cg11942327	-0.466533366	3.751471221	-4.112910049	0.000418088	0.042635614
cg00522212	-0.423277385	0.266323597	-4.11289891	0.0004181	0.042635614
cg13208063	-0.415942055	-0.969912413	-4.112873285	0.000418127	0.042635614
cg21481620	-0.742175528	0.857417651	-4.112814989	0.000418187	0.042635614
cg18114468	-0.485107925	3.39062829	-4.112795739	0.000418208	0.042635614
cg08051105	-0.373851758	0.373166659	-4.112719306	0.000418287	0.042635614
cg06722397	-0.461008003	3.778353413	-4.112644243	0.000418366	0.042635614
cg27511206	-0.634377197	3.219601485	-4.112627798	0.000418383	0.042635614
cg00503646	-0.507298186	3.892946549	-4.112605607	0.000418406	0.042635614
cg02586689	-0.460980272	4.268603806	-4.112404079	0.000418617	0.042635614
cg24667405	-0.659717935	2.642717941	-4.112378287	0.000418644	0.042635614
cg14247180	-0.473588888	3.645795037	-4.112353066	0.00041867	0.042635614
cg16411062	-0.479866664	2.992013871	-4.112345327	0.000418679	0.042635614
cg08217593	-0.574481189	3.625140364	-4.112337487	0.000418687	0.042635614

cg16360861	-0.57473965	-0.767782441	-4.112307644	0.000418718	0.042635614
cg13173204	-0.593363904	1.138937114	-4.11230321	0.000418723	0.042635614
cg02421824	-0.478383603	3.294554088	-4.112140117	0.000418893	0.042646863
cg10514890	-0.464253826	4.272031914	-4.112040154	0.000418998	0.042650337
cg04601296	-0.425759368	-2.375410996	-4.1119925	0.000419048	0.042650337
cg12155841	-0.453608038	3.722229106	-4.111932841	0.00041911	0.042650567
cg03516705	-0.541871897	0.015170157	-4.111821024	0.000419227	0.042651028
cg10425754	-0.353956804	-1.285864789	-4.111813545	0.000419235	0.042651028
cg06056936	-0.488058834	3.667593096	-4.111737981	0.000419314	0.042652956
cg17572348	-0.502855315	3.823573213	-4.111574305	0.000419486	0.042656608
cg01612766	0.566704719	0.850664884	4.111552792	0.000419508	0.042656608
cg05882529	-0.585217816	1.383194123	-4.111531351	0.000419531	0.042656608
cg22512093	-0.587506079	3.283813393	-4.111301689	0.000419772	0.04266627
cg15128801	0.556191898	-3.818147144	4.111222445	0.000419855	0.04266627
cg16710342	-0.566015489	3.362121736	-4.111181246	0.000419898	0.04266627
cg20516004	-0.443628586	-0.058298646	-4.111160362	0.00041992	0.04266627
cg01962517	-0.73159237	2.812573886	-4.111069836	0.000420015	0.04266627
cg02240370	-0.515916104	2.885560594	-4.111050769	0.000420035	0.04266627
cg03337349	-0.49279921	0.329036021	-4.110914222	0.000420178	0.04266627
cg27327073	-0.47436907	3.653778554	-4.110849434	0.000420246	0.04266627
cg14176755	-0.486944715	3.628415081	-4.110828037	0.000420269	0.04266627
cg25681427	-0.670958369	2.345313527	-4.110787309	0.000420312	0.04266627
cg07483629	-0.440738668	3.400152433	-4.110786586	0.000420312	0.04266627
cg12089483	-0.512908655	4.274072994	-4.110722673	0.00042038	0.04266627
cg10605404	-0.441177261	0.766326732	-4.110669763	0.000420435	0.04266627
cg05084043	-0.51290165	-0.637005718	-4.11055685	0.000420554	0.04266627
cg15135422	-0.401496377	-1.222206224	-4.11053171	0.00042058	0.04266627
cg04906352	-0.436013839	-0.320933989	-4.110503149	0.00042061	0.04266627
cg20112453	-0.781968986	2.32112991	-4.110463453	0.000420652	0.04266627
cg02504447	-0.559066609	3.830500443	-4.11040804	0.00042071	0.04266627
cg09185679	-0.488071698	0.335350022	-4.110275165	0.00042085	0.042674329
cg00810228	-0.494262565	-0.270136636	-4.110080613	0.000421054	0.042686176
cg07220201	0.525949946	1.835523279	4.109997119	0.000421142	0.042686176
cg00590630	-0.700688795	-2.312703238	-4.109992239	0.000421147	0.042686176
ch.7.2714749F	0.531919727	-3.536178141	4.109875299	0.000421271	0.042692542
cg21299721	-0.51900355	3.717071777	-4.109778708	0.000421372	0.042696738
cg18573663	-0.556864682	3.247798406	-4.109630967	0.000421528	0.042704439
cg19543867	-0.528018844	0.345123689	-4.109592051	0.000421569	0.042704439
cg18389339	-0.477561972	3.366499569	-4.109433419	0.000421736	0.042704491
cg14683962	-0.623147197	1.962310894	-4.109268263	0.00042191	0.042704491
cg14058077	-0.378029231	3.896018442	-4.109247496	0.000421932	0.042704491
cg16109645	-0.433381914	3.695305721	-4.109240798	0.000421939	0.042704491
cg01750261	-0.695389754	-2.452657197	-4.109211344	0.00042197	0.042704491
cg23139122	-0.433388635	3.984138267	-4.109208061	0.000421973	0.042704491
cg10689011	-0.627099913	2.977321103	-4.109109856	0.000422077	0.042704491
cg08282468	-0.419203685	3.561750648	-4.109079644	0.000422109	0.042704491
cg13564462	-0.664897698	0.681776277	-4.109071113	0.000422118	0.042704491

cg03255440	-0.490711176	0.567877715	-4.109010171	0.000422182	0.042704491
cg01438216	-0.684026116	2.696965872	-4.108951887	0.000422244	0.042704491
cg09188595	-0.44878146	3.624031026	-4.10890536	0.000422293	0.042704491
cg09919522	-0.441682233	3.09923472	-4.108826475	0.000422376	0.042706813
cg06080121	-0.576516809	0.033850382	-4.108736985	0.00042247	0.042708299
cg15681886	-0.406011506	4.02793756	-4.108642614	0.00042257	0.042708299
cg21585975	-0.850540231	1.698638566	-4.108641206	0.000422571	0.042708299
cg03320743	-0.512890731	-0.153355968	-4.108563295	0.000422654	0.04271052
cg07063673	-0.475958931	3.697106319	-4.108404216	0.000422822	0.042710627
cg13328114	-0.520949535	3.74441256	-4.108337034	0.000422893	0.042710627
cg24795721	-0.741085861	2.470132286	-4.10833378	0.000422896	0.042710627
cg20015583	-0.394740373	-0.785941172	-4.108283842	0.000422949	0.042710627
cg02249950	-0.635507506	3.382104742	-4.108244134	0.000422991	0.042710627
cg15648303	-0.64287809	1.349012124	-4.108219866	0.000423017	0.042710627
cg13847977	-0.47092014	4.137551357	-4.108120764	0.000423121	0.042712129
cg06556391	-0.476686909	4.355825401	-4.108055055	0.000423191	0.042712129
cg04275030	-0.521854314	3.156799988	-4.107887803	0.000423368	0.042712129
cg07700524	-0.531535099	-0.427910439	-4.107881376	0.000423374	0.042712129
cg17045284	-0.645968853	2.887113838	-4.107735261	0.000423529	0.042712129
cg12819676	-0.462241245	3.600128843	-4.107732848	0.000423532	0.042712129
cg15551981	-0.500193216	-0.567981124	-4.107732601	0.000423532	0.042712129
cg02095456	-0.647170375	2.42111824	-4.107678285	0.000423589	0.042712129
cg05528187	-0.501744535	-0.31270943	-4.10764852	0.000423621	0.042712129
cg11714647	-0.517654875	3.361802654	-4.107635742	0.000423634	0.042712129
cg06533329	-0.48499183	3.983017241	-4.107486454	0.000423792	0.042718834
cg03957984	-0.475125887	3.322002399	-4.107458998	0.000423822	0.042718834
cg20201959	-0.402359695	0.746323184	-4.10726282	0.000424029	0.042731355
cg14114835	-0.443891291	0.309646046	-4.107200688	0.000424095	0.042731355
cg27459437	-0.439878045	0.712727353	-4.107170892	0.000424127	0.042731355
cg17283455	-0.708787743	0.999375469	-4.107110516	0.000424191	0.042731724
cg10493585	-0.349383082	4.124963382	-4.107015635	0.000424291	0.042735778
cg27168858	-0.491095911	0.055973154	-4.106742262	0.000424581	0.042755923
cg12938890	-0.718586787	1.501889306	-4.106713212	0.000424612	0.042755923
cg15102731	-0.601793017	3.139220829	-4.106607493	0.000424724	0.042761139
cg25555505	-0.538234762	0.920507268	-4.106509045	0.000424829	0.04276558
cg21740232	-0.623137115	-1.067903056	-4.106308374	0.000425042	0.042780948
cg24987667	-0.40671358	0.190954321	-4.106003507	0.000425366	0.042800757
cg24926591	-0.62766457	1.855339924	-4.105987037	0.000425383	0.042800757
cg05802416	-0.439882549	4.012061304	-4.105952509	0.00042542	0.042800757
cg19144912	-0.52083552	2.924624408	-4.105831899	0.000425548	0.042805439
cg20498763	-0.696919202	-2.358091657	-4.105795052	0.000425587	0.042805439
cg22714601	-0.466482915	2.939086709	-4.10552576	0.000425874	0.042813873
cg03974400	-0.682769969	2.02688699	-4.105493601	0.000425908	0.042813873
cg08051321	0.614953116	2.545170024	4.10548466	0.000425917	0.042813873
cg16523078	-0.571253548	2.532069124	-4.10542423	0.000425982	0.042813873
cg20343312	-0.47161818	-0.358494054	-4.10533838	0.000426073	0.042813873
cg06923767	0.662853436	2.287745652	4.105274048	0.000426142	0.042813873

cg10847656	-0.520641961	0.032008961	-4.105266877	0.000426149	0.042813873
cg15501187	-0.582486374	3.580124038	-4.105261823	0.000426155	0.042813873
cg02777922	-0.713172099	3.400280347	-4.104985906	0.000426448	0.04283554
cg09247657	-0.666688748	2.9884256	-4.104767136	0.000426682	0.04283554
cg06029792	-0.558368997	-1.421607443	-4.104652138	0.000426804	0.04283554
cg10152497	-0.546566498	3.118115273	-4.104585207	0.000426876	0.04283554
cg09327714	-0.518757133	-2.627291135	-4.10456903	0.000426893	0.04283554
cg15926677	-0.490535271	-0.321801361	-4.10451173	0.000426954	0.04283554
cg07164606	-0.43177703	3.815141064	-4.10446141	0.000427008	0.04283554
cg14593420	-0.473096734	3.887436496	-4.104455628	0.000427014	0.04283554
cg24301771	-0.560043998	3.102886688	-4.104418441	0.000427053	0.04283554
cg19703248	-0.443390697	3.505992629	-4.104401971	0.000427071	0.04283554
cg12709344	-0.523941524	3.589060689	-4.104352873	0.000427123	0.04283554
cg20196728	-0.684298555	3.044873762	-4.104340178	0.000427137	0.04283554
cg09380122	-0.482991405	-0.107602712	-4.104285174	0.000427196	0.04283554
cg07209034	-0.848380651	-2.095609561	-4.104242226	0.000427241	0.04283554
cg13480079	-0.582614494	3.953091674	-4.104096736	0.000427397	0.04283554
cg01722868	-0.501918596	3.474507324	-4.104060094	0.000427436	0.04283554
cg15664541	-0.508367173	3.501002089	-4.103883449	0.000427625	0.04283554
cg00804078	-0.351012126	-0.557709681	-4.103869259	0.00042764	0.04283554
cg17517534	-0.44637707	-1.150778395	-4.103848661	0.000427662	0.04283554
cg13573178	-0.49051759	3.658010035	-4.103793348	0.000427721	0.04283554
cg15573623	-0.479207709	3.602489222	-4.103727507	0.000427791	0.04283554
cg13982340	0.419550353	1.962179978	4.103726819	0.000427792	0.04283554
cg19572076	-0.472039419	3.567276827	-4.103699781	0.000427821	0.04283554
cg10605767	-0.598126022	2.793990037	-4.103646954	0.000427877	0.04283554
cg05286528	-0.473863975	3.36034973	-4.103532567	0.000428	0.04283554
cg06371799	-0.603026015	2.372145367	-4.103448217	0.00042809	0.04283554
cg02170400	-0.501347487	2.91479168	-4.103357401	0.000428187	0.04283554
cg24493323	-0.626947104	3.490891259	-4.103322001	0.000428225	0.04283554
cg20469837	-0.367749335	-1.726010733	-4.103295183	0.000428254	0.04283554
cg19001273	-0.554654723	-0.316229792	-4.103259868	0.000428291	0.04283554
cg02242424	0.499886066	1.98279158	4.103212514	0.000428342	0.04283554
cg25303774	-0.512357613	3.985189453	-4.103212391	0.000428342	0.04283554
cg21915523	-0.899205447	1.491612724	-4.103185135	0.000428371	0.04283554
cg23286493	-0.671746086	2.809791114	-4.103133892	0.000428426	0.04283554
cg02998566	-0.68635022	2.567030654	-4.102958118	0.000428614	0.042842875
cg17185495	-0.512213787	3.599125034	-4.102949025	0.000428624	0.042842875
cg08735444	-0.551625175	3.852759615	-4.102895901	0.000428681	0.042842875
cg03262503	-0.482213308	0.829604538	-4.102795147	0.000428789	0.042845938
cg25803107	-0.443632381	-1.60261348	-4.102754362	0.000428833	0.042845938
cg08178005	0.399248367	2.043129517	4.102504696	0.0004291	0.042862155
cg14727131	-0.604997729	3.443680395	-4.102411834	0.0004292	0.042862155
cg00882967	-0.472831497	-0.868544191	-4.102404055	0.000429208	0.042862155
cg14622659	-0.622115597	3.302894923	-4.102377132	0.000429237	0.042862155
cg14625829	-0.619249309	1.548347487	-4.102280481	0.000429341	0.042866463
cg01605174	-0.584704321	3.914497563	-4.102153788	0.000429476	0.04287399

cg05698996	-0.443858176	0.054475063	-4.101976725	0.000429666	0.042877164
cg17876463	-0.487547583	0.277843563	-4.101951811	0.000429693	0.042877164
cg12811033	-0.585863122	0.08547665	-4.101846283	0.000429807	0.042877164
cg08846482	0.744755094	-3.726136905	4.101820623	0.000429834	0.042877164
cg03595855	-0.7976317	0.334643741	-4.101789707	0.000429867	0.042877164
cg13707793	-0.436679141	-0.182634013	-4.101785845	0.000429871	0.042877164
cg15628639	-0.736688856	2.728887941	-4.101665804	0.00043	0.042877729
cg02923217	-0.803150797	2.171557044	-4.101616566	0.000430053	0.042877729
cg06737942	-0.818254936	1.716902084	-4.101596362	0.000430075	0.042877729
cg21706705	-0.540462462	3.616775359	-4.101555187	0.000430119	0.042877729
cg20128671	-0.685046785	-3.404893454	-4.101444228	0.000430238	0.042883582
cg15561329	-0.443383692	4.087885054	-4.10132999	0.000430361	0.042886227
cg09196233	-0.368249167	4.356193465	-4.101306913	0.000430386	0.042886227
cg07760853	-0.770218938	1.050440851	-4.101215007	0.000430485	0.042886649
cg25470419	-0.542636285	3.374376853	-4.101190374	0.000430511	0.042886649
cg23616233	-0.500435664	3.722099809	-4.101017816	0.000430697	0.042895403
cg02936601	-0.650220101	2.61698759	-4.100934864	0.000430786	0.042895403
cg27376583	-0.494310591	3.922351206	-4.100932656	0.000430789	0.042895403
cg05322837	-0.601583921	-1.879463368	-4.100883581	0.000430841	0.042895403
cg18749563	-0.466352347	-0.204938431	-4.100746129	0.000430989	0.042901472
cg00097715	-0.485073072	-1.131564547	-4.100709349	0.000431029	0.042901472
cg24303737	-0.550636693	3.932052261	-4.100658206	0.000431084	0.042901472
cg07429817	-0.458228765	3.76451667	-4.10055075	0.0004312	0.042904432
cg18738273	0.368437168	1.364352993	4.100482723	0.000431273	0.042904432
cg15956434	-0.77713094	1.690477637	-4.100461924	0.000431296	0.042904432
cg08053263	-0.43889513	4.061872011	-4.100371555	0.000431393	0.042904861
cg16475806	-0.815078637	2.266256448	-4.100345514	0.000431421	0.042904861
cg12752882	-0.412588413	4.005243279	-4.099859286	0.000431945	0.042936149
cg01421512	-0.494232885	0.966440856	-4.099817473	0.000431991	0.042936149
cg13338877	-0.696231993	-4.059552961	-4.09980221	0.000432007	0.042936149
cg02576930	-0.516401882	3.648100462	-4.09980128	0.000432008	0.042936149
cg25387466	-0.364466697	3.973952886	-4.099772827	0.000432039	0.042936149
cg11559048	-0.44673691	-0.13963322	-4.099700185	0.000432117	0.042937917
cg04799167	-0.512310887	3.663291814	-4.09959582	0.00043223	0.04294309
cg12913502	-0.563951178	-1.28030119	-4.099513439	0.000432319	0.042943145
cg14692284	-0.632562277	3.429142318	-4.099480766	0.000432354	0.042943145
cg09584702	-0.676329761	-0.950811013	-4.099388599	0.000432454	0.042943145
cg22152887	-0.56846591	3.354031149	-4.099326847	0.00043252	0.042943145
cg20875397	-0.485287031	3.990459544	-4.099314695	0.000432533	0.042943145
cg05321683	-0.656751878	3.282691112	-4.099255182	0.000432598	0.042943511
cg26328240	-0.7613436	-0.125942397	-4.098978346	0.000432897	0.04294773
cg04135846	-0.444229581	-0.665052707	-4.098947757	0.00043293	0.04294773
cg18327319	-0.62257871	0.381392536	-4.098899335	0.000432982	0.04294773
cg18727010	-0.454190175	-0.231741918	-4.098886743	0.000432996	0.04294773
cg02451467	-0.416719992	3.216192423	-4.098659062	0.000433242	0.04294773
cg02951021	-0.449406611	-2.790774751	-4.098644136	0.000433259	0.04294773
cg11008003	-0.547389383	-0.038677235	-4.098596724	0.00043331	0.04294773

cg10394623	-0.512309752	3.529983475	-4.098594488	0.000433312	0.04294773
cg13655566	-0.561240315	2.597863545	-4.098560025	0.00043335	0.04294773
cg05434126	-0.836699812	1.514644534	-4.098521013	0.000433392	0.04294773
cg24822086	-0.657666266	1.90615396	-4.098514907	0.000433399	0.04294773
cg19595102	-0.963433685	1.294199366	-4.098416302	0.000433505	0.04294773
cg26953075	-0.492431779	3.604638836	-4.098361166	0.000433565	0.04294773
cg07007537	-0.855494927	1.881134027	-4.098343052	0.000433585	0.04294773
cg14408605	-0.526864752	2.969976211	-4.098336202	0.000433592	0.04294773
cg05153029	-0.571891014	3.311676575	-4.09831933	0.00043361	0.04294773
cg07515071	-0.604003661	2.64115129	-4.098024621	0.00043393	0.042968936
cg10523370	-0.490983995	-0.889702878	-4.098009801	0.000433946	0.042968936
cg00490240	-0.444652236	3.581731506	-4.09783335	0.000434137	0.042981872
cg07732037	-0.607287113	3.613510731	-4.097665177	0.000434319	0.042989837
cg02068832	-0.458581376	3.759707668	-4.097610447	0.000434379	0.042989837
cg01062470	-0.582047329	3.519396337	-4.097591374	0.0004344	0.042989837
cg14630843	-0.512325803	0.486795055	-4.097356057	0.000434655	0.043009107
cg17734182	-0.377780799	3.853871613	-4.097283213	0.000434734	0.043010926
cg06491878	-0.374795222	-0.223706891	-4.097156983	0.000434871	0.043018483
cg13837837	-0.63717129	3.224450176	-4.096987651	0.000435055	0.043030674
cg06925645	-0.507221186	-0.660376067	-4.096857286	0.000435197	0.043032019
cg12359854	-0.781379129	2.599396549	-4.096829873	0.000435227	0.043032019
cg06847917	-0.406419246	3.710820516	-4.096759917	0.000435303	0.043032019
cg15230057	-0.416915871	0.327407246	-4.096751627	0.000435312	0.043032019
cg05954699	-0.407396264	3.961665554	-4.096657329	0.000435414	0.043036151
cg11259184	-0.518416743	2.583285614	-4.096368867	0.000435728	0.043061166
cg21700582	-0.494419592	-0.698051499	-4.096249915	0.000435858	0.043064676
cg24732078	-0.495002644	4.239223425	-4.096173902	0.00043594	0.043064676
cg23291401	-0.819290882	1.134579702	-4.096137228	0.00043598	0.043064676
cg02931266	-0.937775317	2.970966043	-4.096112903	0.000436007	0.043064676
cg16305018	-0.521955478	0.229495406	-4.095943707	0.000436191	0.043072514
cg23330845	-0.69858448	1.685506081	-4.095725228	0.000436429	0.043072514
cg12455465	-0.546550017	3.884253812	-4.0957242	0.00043643	0.043072514
cg02022289	-0.473727102	4.048091305	-4.095655872	0.000436505	0.043072514
cg13983673	-0.502941215	4.149938809	-4.095630746	0.000436532	0.043072514
cg21617644	-0.656451807	1.112346603	-4.095620442	0.000436544	0.043072514
ch.13.32987270F	-0.713806246	-1.586237188	-4.095604682	0.000436561	0.043072514
cg10539170	-0.516733747	3.43837782	-4.095593767	0.000436573	0.043072514
cg23054456	-0.624525002	-1.137797329	-4.095215081	0.000436986	0.043103802
cg07967176	-0.450982703	3.139272342	-4.095191581	0.000437011	0.043103802
cg22348843	-0.543312891	-1.32595871	-4.095053719	0.000437162	0.04310933
cg26965476	-0.431107383	0.33178866	-4.095028777	0.000437189	0.04310933
cg11903239	-0.777997904	2.233633935	-4.094951167	0.000437274	0.043111688
cg13788000	-0.586131806	0.280821665	-4.094850855	0.000437384	0.043115986
cg22909333	-0.45840469	3.575170655	-4.094799839	0.000437439	0.043115986
cg20442289	-0.490390131	-0.374960511	-4.094673289	0.000437578	0.0431201
cg20950529	-0.465125975	4.066788865	-4.094650257	0.000437603	0.0431201
cg13319203	-0.562507772	3.16235385	-4.094418374	0.000437856	0.043138748

cg26132229	-0.543685393	3.133635318	-4.094307526	0.000437978	0.043138748
cg27486909	-0.981566697	1.027026727	-4.094294362	0.000437992	0.043138748
cg19538677	0.451462233	1.475573592	4.09415055	0.000438149	0.043138748
cg23960652	-0.374988438	-0.607559148	-4.094149814	0.00043815	0.043138748
cg24531558	-0.4525399	3.764991774	-4.094063075	0.000438245	0.043138748
cg20904375	-0.522787375	2.572877336	-4.094037421	0.000438273	0.043138748
cg09657446	-0.40355689	-0.15837408	-4.094031914	0.000438279	0.043138748
cg22146131	-0.598354445	2.249646878	-4.093968908	0.000438348	0.043139495
cg08213575	-0.719744277	2.398422881	-4.09391375	0.000438409	0.043139495
cg23339720	-0.64229653	-1.729497881	-4.093811432	0.000438521	0.043144529
cg10164398	-0.439809593	0.052226928	-4.093653005	0.000438694	0.043155613
cg10587366	-0.535640514	0.469957679	-4.093580325	0.000438774	0.043156968
cg14725631	-0.746272476	2.122793658	-4.093529261	0.00043883	0.043156968
cg05629639	-0.570178547	3.657308845	-4.093327374	0.000439051	0.043164
cg21066558	-0.636708142	2.922767691	-4.093262432	0.000439122	0.043164
cg04249541	-0.503732212	-0.393446379	-4.093234464	0.000439153	0.043164
cg19368394	-0.604767809	3.727230268	-4.093233313	0.000439154	0.043164
cg14718242	-0.557870788	2.790819621	-4.093186234	0.000439206	0.043164
cg09183033	-0.474058888	3.814792613	-4.093010694	0.000439399	0.043169623
cg10477313	-0.478033363	3.687251999	-4.092977526	0.000439435	0.043169623
cg20213005	-0.487702119	3.237015368	-4.09294505	0.000439471	0.043169623
cg04897932	-0.501693101	3.724760141	-4.092911982	0.000439507	0.043169623
cg03827087	-0.566092936	-1.915208224	-4.092740532	0.000439695	0.043177838
cg22307099	-0.648593854	3.02634047	-4.092724799	0.000439713	0.043177838
cg08517093	0.651334411	1.778541526	4.092598951	0.000439851	0.043184144
cg15208393	-0.349529731	-0.548994136	-4.092555364	0.000439899	0.043184144
cg07128741	-0.434764591	-0.759260128	-4.092346066	0.000440129	0.043200739
cg22874407	-0.449461087	-0.315607516	-4.092028174	0.000440478	0.043229067
cg08000231	-0.767034443	3.089349937	-4.09160326	0.000440946	0.043268978
cg05937573	-0.597264072	3.155219215	-4.091496141	0.000441064	0.043274562
cg15829152	-0.41870039	4.132931749	-4.091407509	0.000441162	0.043278149
cg08773573	-0.671555977	3.464114263	-4.091316647	0.000441262	0.043281979
cg09935357	-0.444393845	-2.09768067	-4.091217586	0.000441371	0.043286696
cg22325435	-0.399795537	3.621123459	-4.091160733	0.000441434	0.04328685
cg15560854	-0.597901528	3.07404371	-4.090971277	0.000441643	0.043295298
cg22642710	0.45814158	1.873531666	4.090945476	0.000441671	0.043295298
cg03523917	-0.630169409	2.065705076	-4.090910149	0.00044171	0.043295298
cg13530091	-0.566697073	2.935764838	-4.090860989	0.000441764	0.043295298
cg19721993	-0.485763188	3.332420513	-4.090702395	0.000441939	0.043301442
cg16265403	-0.56017269	-0.533256431	-4.090660074	0.000441986	0.043301442
cg23484721	-0.489647818	4.5215632	-4.090638049	0.000442011	0.043301442
cg21648691	0.600905237	2.760809563	4.090461092	0.000442206	0.043314537
cg27473162	-0.703145484	2.495664909	-4.090406278	0.000442266	0.043314537
cg15847996	-0.535861737	-1.615933379	-4.090281164	0.000442405	0.043320541
cg22592142	-0.520487567	-1.581082711	-4.090240097	0.00044245	0.043320541
cg06027629	-0.493792343	3.405337963	-4.089998524	0.000442717	0.043340698
cg13486491	-0.901864632	1.108262457	-4.089926267	0.000442797	0.04334253

cg26019259	-0.500573015	1.189134156	-4.089855678	0.000442875	0.043344184
cg05073720	-0.674123974	-0.789582756	-4.089648976	0.000443104	0.043360577
cg05344920	-0.629280228	3.683151526	-4.089572841	0.000443188	0.043362833
cg03315407	-0.883267169	-2.383003242	-4.089450387	0.000443324	0.043367856
cg16345899	-0.462733766	-0.236580636	-4.089405229	0.000443374	0.043367856
cg14789501	-0.509458356	0.330269098	-4.089360604	0.000443423	0.043367856
cg08193398	-0.526430134	3.562799199	-4.08921353	0.000443586	0.04337235
cg10023565	-0.574593137	3.563143212	-4.089190349	0.000443612	0.04337235
cg13335775	-0.499648587	-0.787676144	-4.089131579	0.000443677	0.04337235
cg25204176	-0.486909543	3.86385463	-4.089069268	0.000443746	0.04337235
cg10089827	-0.461643762	2.619323592	-4.089042837	0.000443775	0.04337235
cg21820921	0.766249417	-1.248823645	4.08865208	0.000444209	0.043408712
cg22810156	-0.435206126	3.799631116	-4.088486426	0.000444392	0.043414808
cg06255989	-0.517387708	3.607537202	-4.088485417	0.000444393	0.043414808
cg00414868	-0.824827442	2.650201685	-4.088425456	0.00044446	0.043415323
cg18838642	-0.725532731	-1.117672628	-4.088244118	0.000444661	0.043429003
cg09965499	-0.438292023	4.054917951	-4.088127816	0.000444791	0.043435631
cg26318645	-0.468589674	-1.14038586	-4.088071012	0.000444854	0.043435806
cg22884551	-0.503229775	3.676995073	-4.087888861	0.000445056	0.043445187
cg14552100	-0.554043727	3.290445198	-4.087874193	0.000445072	0.043445187
cg09750510	-0.51444747	2.825680039	-4.087737284	0.000445225	0.043448244
cg01028925	-0.360683025	0.217074707	-4.087676815	0.000445292	0.043448244
cg18418238	-0.373536954	-0.957593314	-4.087675715	0.000445293	0.043448244
cg14277677	-0.416398191	-0.508504959	-4.087625396	0.000445349	0.043448244
cg00704909	0.562468575	-4.459856318	4.087452553	0.000445541	0.043451546
cg02561103	-0.635271717	2.458194396	-4.087443603	0.000445551	0.043451546
cg09418354	-0.503080679	3.573521631	-4.087399383	0.000445601	0.043451546
cg02725654	-0.776931063	-3.032275521	-4.087374474	0.000445628	0.043451546
cg10717869	-0.433575245	-1.925422668	-4.087205739	0.000445816	0.043461277
cg10898833	-0.708013545	2.730918166	-4.087174609	0.000445851	0.043461277
cg16060330	-0.382123361	3.900052029	-4.087091644	0.000445943	0.043464303
cg04227743	-0.422342561	3.979892791	-4.08695127	0.0004461	0.043469052
cg24834960	-0.78046359	3.24865098	-4.086937736	0.000446115	0.043469052
cg00935662	-0.48952241	3.685571114	-4.086756096	0.000446317	0.043482796
cg24693376	-0.64972731	2.603503289	-4.086650671	0.000446435	0.043487342
cg05461259	-0.585648187	-0.657988544	-4.08660412	0.000446486	0.043487342
cg15819225	-0.497432763	3.41289855	-4.08626047	0.00044687	0.043501852
cg00670420	-0.617003225	3.199180659	-4.086190975	0.000446947	0.043501852
cg06874985	-0.677985948	2.960072412	-4.08610801	0.00044704	0.043501852
cg05058787	-0.535044782	-0.279940372	-4.086098541	0.000447051	0.043501852
cg04719462	-0.383501018	0.166828617	-4.08607907	0.000447072	0.043501852
cg20010714	0.72574884	-4.501979001	4.086050377	0.000447104	0.043501852
cg21585707	-0.474447333	-1.75025996	-4.085970374	0.000447194	0.043501852
cg17459911	-0.525863501	3.09151308	-4.085932736	0.000447236	0.043501852
cg14438279	-0.398453664	4.022879638	-4.085851577	0.000447326	0.043501852
cg16332112	0.89735761	-1.931583171	4.085835359	0.000447345	0.043501852
cg04513913	-0.560983979	3.380681802	-4.085757553	0.000447432	0.043501852

cg02869163	-0.606486678	-1.662832545	-4.085723652	0.000447469	0.043501852
cg04551914	-0.704866215	1.6087669	-4.085654979	0.000447546	0.043501852
cg27255072	-0.452404005	3.722503316	-4.085542937	0.000447671	0.043501852
cg25985873	-0.554493361	3.246836983	-4.085522013	0.000447695	0.043501852
cg24179646	-0.615240894	-3.207282507	-4.085463084	0.000447761	0.043501852
cg24089612	-0.583773447	3.1680264	-4.085444233	0.000447782	0.043501852
cg05628259	-0.786506259	1.618160497	-4.085374338	0.00044786	0.043501852
cg06488165	-0.681668979	2.986898726	-4.085357676	0.000447879	0.043501852
cg14200954	-0.440009296	3.254741169	-4.085329825	0.00044791	0.043501852
cg20457544	-0.397557437	0.108374212	-4.085268564	0.000447978	0.043501852
cg20090869	-0.45126378	-3.166968188	-4.085261239	0.000447986	0.043501852
cg04260016	-0.488903589	3.972962974	-4.085124191	0.00044814	0.043505666
cg17374726	-0.423425006	-0.583711478	-4.08511638	0.000448149	0.043505666
cg09567485	-0.643528491	-2.86528105	-4.085020816	0.000448256	0.043510088
cg26777029	-0.587895866	2.96962297	-4.084823668	0.000448476	0.043520264
cg03618571	-0.554911655	0.253611302	-4.084813682	0.000448488	0.043520264
cg12982932	-0.57732906	2.775018852	-4.084762626	0.000448545	0.043520264
cg03933149	-0.59274509	2.762890845	-4.084640234	0.000448682	0.043527607
cg05560630	0.616014411	-3.4394416	4.084572228	0.000448758	0.04352904
cg14727845	-0.497962277	3.022449817	-4.084410936	0.000448939	0.043540616
cg18980440	-0.542532326	-1.206056635	-4.08434986	0.000449007	0.043541297
cg18556587	-0.957299516	-0.378113021	-4.08399814	0.000449402	0.043573596
cg09000385	-0.488151235	-1.431443569	-4.083848317	0.00044957	0.043581639
cg15884546	-0.784297253	1.582762459	-4.083814643	0.000449608	0.043581639
cg27207530	-0.678482565	4.047305501	-4.083640459	0.000449804	0.043586953
cg02335671	-0.459184985	3.757961164	-4.083611972	0.000449836	0.043586953
cg19685672	-0.38962135	-0.437854691	-4.08356193	0.000449892	0.043586953
cg26576576	-0.554136769	3.161159762	-4.083546725	0.000449909	0.043586953
cg15416770	-0.434934252	3.620581652	-4.083463604	0.000450002	0.04358711
cg22137273	-0.556459117	2.896209511	-4.083435773	0.000450034	0.04358711
cg14261568	-0.416967565	4.205351829	-4.083307925	0.000450177	0.043589269
cg12762474	-0.438611129	-0.140073388	-4.083082936	0.00045043	0.043589269
cg26557253	-0.57294531	2.820059629	-4.082926242	0.000450607	0.043589269
cg10642175	-0.669160766	-1.333786285	-4.082878761	0.00045066	0.043589269
cg13962718	-0.365200374	-0.290296785	-4.08287703	0.000450662	0.043589269
cg06900068	-0.482667417	0.098406507	-4.082870823	0.000450669	0.043589269
cg20306285	-0.590302876	1.792775937	-4.082855233	0.000450687	0.043589269
cg20443763	-0.348301235	4.050680276	-4.082828719	0.000450716	0.043589269
cg24530557	-0.533436313	3.381116718	-4.082683721	0.00045088	0.043589269
cg27384047	-0.503629935	3.231622038	-4.082648113	0.00045092	0.043589269
cg18861810	-0.550321035	1.199700788	-4.082617781	0.000450954	0.043589269
cg15537567	-0.583705418	3.11950404	-4.082585254	0.000450991	0.043589269
cg07992435	-0.458247014	-0.647265727	-4.082556291	0.000451023	0.043589269
cg03311477	-0.636110425	2.718948104	-4.082443532	0.00045115	0.043589269
cg19487905	-0.455901966	0.18864751	-4.082379348	0.000451222	0.043589269
cg07599507	-0.671732801	3.330483853	-4.082348922	0.000451257	0.043589269
cg03923587	-0.396375938	3.867393664	-4.082345885	0.00045126	0.043589269

cg02514895	-0.56578732	-0.416593824	-4.082344342	0.000451262	0.043589269
cg14261898	-0.7478536	2.739170401	-4.082340027	0.000451267	0.043589269
cg01275262	-0.573739976	2.133495593	-4.082284099	0.00045133	0.043589269
cg11265104	-0.616237408	2.565997713	-4.082267927	0.000451348	0.043589269
cg13256716	-0.436752565	3.521472525	-4.082136222	0.000451497	0.043597665
cg15626796	-0.518150239	3.974568496	-4.082037223	0.000451608	0.043602501
cg25550220	-0.666006932	1.728649416	-4.081803583	0.000451872	0.043615949
cg21292476	-0.751616921	-0.879203779	-4.081795972	0.00045188	0.043615949
cg27528971	-0.542310102	2.287927077	-4.081666291	0.000452027	0.043615949
cg13992856	0.733449802	-3.407706707	4.081640028	0.000452056	0.043615949
cg26548574	-0.55291399	3.498898828	-4.081631253	0.000452066	0.043615949
cg06452331	-0.49163107	3.719822311	-4.081586423	0.000452117	0.043615949
cg11556949	-0.402676111	-0.450120042	-4.081345252	0.000452389	0.043621548
cg01796107	-0.478960245	4.004416363	-4.081331942	0.000452404	0.043621548
cg21243848	-0.509852572	-0.752106574	-4.081327778	0.000452409	0.043621548
cg05309177	-0.450484287	-0.422928507	-4.081199153	0.000452554	0.043621548
cg25530288	-0.729206068	-0.111490204	-4.081174125	0.000452583	0.043621548
cg21510305	-0.464241949	-0.457281407	-4.08108221	0.000452687	0.043621548
cg03982568	-0.627381286	3.196445264	-4.081059179	0.000452713	0.043621548
cg08124561	-0.581683654	2.696476699	-4.081049809	0.000452723	0.043621548
cg18123675	-0.671569457	2.985280253	-4.080988668	0.000452792	0.043621548
cg10989629	-0.561197663	2.321129133	-4.08098619	0.000452795	0.043621548
cg16859636	-0.62051255	0.62633776	-4.080935633	0.000452852	0.043621548
cg25444696	-0.537459311	3.389345701	-4.080834401	0.000452967	0.043626645
cg10095431	-0.453142012	4.328457644	-4.080700021	0.000453119	0.043634157
cg11768833	-0.669412408	-0.281427153	-4.080656588	0.000453168	0.043634157
cg02326750	-0.897116707	2.25680484	-4.080521512	0.000453321	0.043642946
cg16109455	-0.389881707	-2.49494709	-4.080349269	0.000453516	0.04365579
cg06241057	-0.446964827	3.487498474	-4.080143167	0.000453749	0.043661768
cg16719077	-0.572272008	2.983424867	-4.080080871	0.00045382	0.043661768
cg14953704	-0.397427443	-0.522059512	-4.080016551	0.000453893	0.043661768
cg08120114	-0.5009586	3.611130296	-4.080012222	0.000453898	0.043661768
cg20456954	-0.515781951	3.761137426	-4.079870152	0.000454059	0.043661768
cg10032230	-0.575649294	2.937599024	-4.079822421	0.000454113	0.043661768
cg12278688	-0.389069656	3.962843356	-4.079738465	0.000454208	0.043661768
cg20889322	-0.540831602	-1.045519407	-4.079700694	0.000454251	0.043661768
cg18769171	-0.423310954	0.402718532	-4.07963163	0.000454329	0.043661768
ch.7.54842076F	-0.819652632	-2.953882039	-4.079569837	0.0004544	0.043661768
cg24825242	-0.53155342	4.155793233	-4.079551758	0.00045442	0.043661768
cg09607047	-0.471218406	-1.328978164	-4.079543548	0.000454429	0.043661768
cg12832726	-0.36009736	0.072383646	-4.079516685	0.00045446	0.043661768
cg26854360	-0.506591878	3.242422538	-4.079487669	0.000454493	0.043661768
cg00205746	-0.448670232	3.420927096	-4.07947906	0.000454503	0.043661768
cg11101522	-0.437918023	3.636928971	-4.079230309	0.000454785	0.043682975
cg11337413	-0.652517281	-1.958546173	-4.079008033	0.000455037	0.043694289
cg22263379	0.659404244	3.249575056	4.078937783	0.000455117	0.043694289
cg16830520	-0.732855636	2.905339225	-4.078865263	0.0004552	0.043694289

cg15387055	-0.404063417	3.702149336	-4.078863303	0.000455202	0.043694289
cg20599420	-0.550706115	0.129816474	-4.078794512	0.00045528	0.043694289
cg09580485	0.598556571	2.660906119	4.078791515	0.000455284	0.043694289
cg09549824	-0.439903347	3.931081744	-4.078724489	0.00045536	0.043694289
cg07356096	-0.462550263	4.0775118	-4.078477533	0.000455641	0.043694289
cg11440344	-0.505058097	3.505805947	-4.078473285	0.000455646	0.043694289
cg04009868	-0.44890733	4.141880915	-4.078416817	0.00045571	0.043694289
cg01022336	-0.503807963	3.963114099	-4.078282533	0.000455863	0.043694289
cg01355046	-0.476642236	-0.852098809	-4.078196015	0.000455961	0.043694289
cg10618461	-0.512402557	3.067865247	-4.07818051	0.000455979	0.043694289
cg22990971	-0.548189387	3.816280093	-4.078167386	0.000455994	0.043694289
cg02579241	-0.663903003	-0.934332299	-4.078134458	0.000456031	0.043694289
cg03245139	-0.634221038	1.30849369	-4.078022249	0.000456159	0.043694289
cg11964006	-0.594222627	2.751252875	-4.0780195	0.000456162	0.043694289
cg04992665	-0.81302039	0.000926308	-4.077909348	0.000456288	0.043694289
cg21557493	0.576661164	2.979764186	4.077842268	0.000456364	0.043694289
cg22985373	-0.662025751	2.776428998	-4.077829494	0.000456379	0.043694289
cg03561638	-0.638188722	2.079438209	-4.077686488	0.000456542	0.043694289
cg10904156	-0.409102555	-0.512872759	-4.077681929	0.000456547	0.043694289
cg26387338	-0.424354167	3.905568016	-4.077495368	0.00045676	0.043694289
cg13670883	-0.549241148	1.649356892	-4.07748125	0.000456776	0.043694289
cg04755864	-0.490817921	3.609160972	-4.077426152	0.000456839	0.043694289
cg07210017	-0.467115505	-0.554565262	-4.077426021	0.000456839	0.043694289
cg15346493	-0.425840244	0.201099561	-4.077400005	0.000456868	0.043694289
cg23239870	-0.398169928	3.187028982	-4.077377035	0.000456895	0.043694289
cg00925393	-0.469595252	2.400775203	-4.077311044	0.00045697	0.043694289
cg14970554	-0.424693936	3.778900293	-4.077275386	0.000457011	0.043694289
cg09766512	0.553753726	2.276632977	4.077240546	0.00045705	0.043694289
cg25250661	-0.439597499	3.54740396	-4.077186831	0.000457112	0.043694289
cg27579505	0.447968629	3.159286034	4.077168908	0.000457132	0.043694289
cg08649765	-0.492142815	3.734671294	-4.077095773	0.000457216	0.043694289
cg20839257	-0.685460642	2.93221671	-4.077091561	0.00045722	0.043694289
cg24375958	-0.413876274	4.015464964	-4.076987168	0.00045734	0.043694289
cg19275995	-0.600437122	3.235921999	-4.076963548	0.000457367	0.043694289
cg04861934	-0.452313351	1.486880949	-4.07696295	0.000457367	0.043694289
cg20087253	-0.464655813	3.724492	-4.076961572	0.000457369	0.043694289
cg03045325	-0.492944086	0.897646979	-4.076883008	0.000457459	0.043694289
cg06196600	-0.819842577	2.926814176	-4.076845005	0.000457502	0.043694289
cg07267942	-0.617674165	2.704228781	-4.076792828	0.000457562	0.043694289
cg25113053	-0.567429513	3.748099312	-4.076764743	0.000457594	0.043694289
cg20083463	-0.796496744	2.47704529	-4.076625678	0.000457753	0.043694289
cg11270940	-0.586533643	2.528373359	-4.076620422	0.000457759	0.043694289
cg17348714	-0.38739251	-1.289431361	-4.076595879	0.000457787	0.043694289
cg03648054	-0.461988476	0.529843281	-4.076570723	0.000457815	0.043694289
cg15419852	-0.57200029	-4.410631576	-4.076497034	0.0004579	0.043694289
cg18754578	-0.519322518	3.484611372	-4.076434487	0.000457971	0.043694289
cg05376034	-0.375353854	-0.469420955	-4.076409993	0.000457999	0.043694289

cg14332927	-0.432316652	3.644948737	-4.076366918	0.000458049	0.043694289
cg04207869	-0.409681432	0.675610447	-4.076153779	0.000458292	0.043707144
cg23111190	-0.544144567	2.986300485	-4.076141283	0.000458307	0.043707144
cg25970454	-0.528225095	3.565887401	-4.076055686	0.000458405	0.043710603
cg04156643	-0.495522291	3.334677803	-4.075976299	0.000458496	0.043713384
cg01380884	-0.413004609	0.697871973	-4.07570222	0.000458809	0.04373225
cg01195099	-0.658461291	3.217677525	-4.075651324	0.000458868	0.04373225
cg23101005	-0.596007433	3.058159503	-4.075514716	0.000459024	0.04373225
cg09171763	0.509037008	2.860973979	4.075434207	0.000459117	0.04373225
cg04788432	-0.545779403	3.361349294	-4.075429175	0.000459122	0.04373225
cg26743136	-0.542121242	0.280429429	-4.075408295	0.000459146	0.04373225
cg05443459	-0.644018287	1.020665746	-4.075265726	0.00045931	0.04373225
cg11000285	-0.554125654	3.140070482	-4.075233871	0.000459346	0.04373225
cg17825378	-0.365196683	4.005314944	-4.075225027	0.000459356	0.04373225
cg23216498	-0.428709668	4.139159809	-4.07520983	0.000459374	0.04373225
cg25707465	-0.519515373	3.634854917	-4.075161575	0.000459429	0.04373225
cg26094195	-0.513555506	3.178466294	-4.075041878	0.000459567	0.04373225
cg10581117	-0.62424648	3.349809614	-4.074946965	0.000459676	0.04373225
cg01609275	-0.398818273	-1.631644508	-4.07492383	0.000459702	0.04373225
cg03201367	-0.459901871	3.473169134	-4.074913248	0.000459714	0.04373225
cg09753013	-0.566136874	-1.470291604	-4.074902203	0.000459727	0.04373225
cg26400275	-0.519215557	-1.257500061	-4.074777175	0.00045987	0.04373225
cg21071116	-0.620049725	2.582920672	-4.074768478	0.00045988	0.04373225
cg15844645	-0.623795858	-0.238267656	-4.07474751	0.000459905	0.04373225
cg25262782	-0.573752246	0.755405482	-4.07472698	0.000459928	0.04373225
cg05872758	-0.41590755	-0.828791162	-4.074631514	0.000460038	0.043732721
cg16098469	-0.558131675	3.874731552	-4.074544958	0.000460137	0.043732721
cg03613730	-0.419992089	-0.770507436	-4.074528361	0.000460156	0.043732721
cg10673648	-0.635369937	3.188422036	-4.07448994	0.0004602	0.043732721
cg00339609	-0.4274032	3.990292335	-4.074453983	0.000460242	0.043732721
cg02909136	-0.68152891	-3.011402414	-4.074201386	0.000460532	0.043749574
cg14035807	0.5566127	3.527787208	4.074032493	0.000460726	0.043749574
cg21801252	-0.494808588	3.236709058	-4.07402996	0.000460729	0.043749574
cg03633706	-0.564089517	2.315466926	-4.074000388	0.000460763	0.043749574
cg07376297	-0.884540185	2.550429084	-4.07396095	0.000460809	0.043749574
cg20848130	-0.474313087	0.501651215	-4.073929393	0.000460845	0.043749574
cg23516227	-0.445235747	-0.357052618	-4.07392382	0.000460851	0.043749574
cg13164535	-0.595884089	2.583696248	-4.073727813	0.000461077	0.043765127
cg05297237	-0.503138725	3.312869438	-4.073602334	0.000461222	0.043765726
cg12266167	-0.466461218	3.675315569	-4.073572123	0.000461256	0.043765726
cg13195254	-0.642402523	-3.697065066	-4.073561379	0.000461269	0.043765726
cg02050968	-0.675027172	1.981210504	-4.073346923	0.000461516	0.043783304
cg00243323	-0.430857068	3.787450338	-4.073287927	0.000461584	0.043783891
cg04977222	-0.522073331	0.248386909	-4.073179752	0.000461708	0.043789855
cg05309567	-0.516296876	3.446146435	-4.073060997	0.000461845	0.04379037
cg03518664	-0.475346414	3.593611132	-4.072978806	0.00046194	0.04379037
cg13924134	-0.33295432	-0.387338318	-4.072973317	0.000461947	0.04379037

cg09365107	-0.67998373	2.089849268	-4.072925592	0.000462002	0.04379037
cg24036612	-0.522905199	3.290855563	-4.072869179	0.000462067	0.04379037
cg15264162	-0.761197338	-0.761614371	-4.072853454	0.000462085	0.04379037
cg04452536	-0.643252781	2.729444554	-4.072726468	0.000462231	0.043794512
cg16951984	0.646074664	2.49310151	4.072631607	0.000462341	0.043794512
cg11411535	-0.598878488	0.49478461	-4.072628557	0.000462344	0.043794512
cg04305062	-0.415978289	-0.784852425	-4.072596401	0.000462381	0.043794512
cg15913873	-0.556983397	3.085232768	-4.07249977	0.000462493	0.043794512
cg21313498	-0.742979808	2.855914359	-4.072494238	0.000462499	0.043794512
cg12026235	-0.561180032	3.988625606	-4.072380828	0.00046263	0.043796777
cg20681704	-0.833960604	2.64138822	-4.072366471	0.000462647	0.043796777
cg20065463	-0.581795387	-2.88132212	-4.07226564	0.000462764	0.043801952
cg18395637	-0.691086597	2.192252916	-4.071987907	0.000463085	0.043812681
cg12254216	-0.525082231	2.316376634	-4.071854144	0.000463239	0.043812681
ch.3.3564705F	0.831424481	-1.158173268	4.071773952	0.000463332	0.043812681
cg06303119	-0.409602223	4.305493867	-4.071771842	0.000463334	0.043812681
cg12824929	-0.761967715	2.460414454	-4.071763941	0.000463344	0.043812681
cg18336878	-0.65508123	3.286081175	-4.071760031	0.000463348	0.043812681
cg05565176	-0.659458546	2.530258357	-4.071753309	0.000463356	0.043812681
cg00922044	-0.430584832	3.676798545	-4.071702308	0.000463415	0.043812681
cg04302055	0.432932014	1.707697101	4.071642895	0.000463484	0.043812681
cg12357497	-0.617194961	-1.007132992	-4.071632774	0.000463495	0.043812681
cg13250654	-0.568458155	2.087348855	-4.071520032	0.000463626	0.043815119
cg20539321	1.035614248	-4.751679503	4.071408702	0.000463755	0.043815119
cg03423916	-0.579847033	-0.571678155	-4.071391119	0.000463775	0.043815119
cg03718509	-0.469061932	-0.822870643	-4.07136508	0.000463805	0.043815119
cg14312334	-0.424953465	-0.915804052	-4.071188729	0.00046401	0.043815119
cg17648977	-0.435882435	3.72703299	-4.071180027	0.00046402	0.043815119
cg14608759	-0.602465109	3.277938152	-4.071177768	0.000464022	0.043815119
cg01606156	-0.456719398	3.503345706	-4.071149328	0.000464055	0.043815119
cg06852605	-0.304264956	0.796951993	-4.071045104	0.000464176	0.043815119
cg01261954	-0.862240575	-0.512471601	-4.07103433	0.000464188	0.043815119
cg16449987	-0.48457029	4.08810256	-4.071023052	0.000464202	0.043815119
cg10312522	-0.627643093	-1.046565622	-4.070805447	0.000464454	0.043833091
cg15534018	-0.348899378	4.217034458	-4.070638056	0.000464648	0.043845574
cg04571183	0.541989051	2.160395839	4.070235667	0.000465115	0.043883802
cg11436333	-0.859912657	2.752327504	-4.070167517	0.000465194	0.043885425
cg27050584	-0.78438438	0.716674215	-4.070043777	0.000465338	0.04389314
cg23400942	0.645361437	1.90265885	4.069969649	0.000465424	0.04389542
cg21312906	-0.47419242	0.998385554	-4.069801556	0.000465619	0.043908001
cg03134111	-0.505920786	1.104786429	-4.069679786	0.000465761	0.043910732
cg06125996	-0.581796548	0.718258962	-4.069657161	0.000465787	0.043910732
cg13717684	-0.453249789	-1.385042029	-4.069616731	0.000465834	0.043910732
cg22315577	-0.758592162	2.786264341	-4.069539191	0.000465925	0.04391339
cg21710060	-0.434382771	2.819492685	-4.069383361	0.000466106	0.043916437
cg04338621	-0.611128972	3.102134432	-4.069361605	0.000466131	0.043916437
cg16917158	-0.445266163	3.84786542	-4.069309517	0.000466192	0.043916437

cg08697117	-0.342554829	0.350753897	-4.069298305	0.000466205	0.043916437
cg23102501	-0.597874575	2.443275668	-4.069240429	0.000466272	0.043916944
cg03560685	-0.51098382	-0.09424028	-4.069159769	0.000466366	0.043917007
cg12248457	-0.578589964	-0.600191712	-4.069069831	0.000466471	0.043917007
cg25661961	-0.986535569	1.385945424	-4.069054923	0.000466488	0.043917007
cg12118446	-0.742024716	2.207874514	-4.069026896	0.000466521	0.043917007
cg09676602	-0.567166542	3.204493047	-4.068901342	0.000466667	0.043924939
cg26314722	-0.533571525	-0.43635731	-4.068789584	0.000466797	0.043930581
cg02724067	-0.429766109	-1.441035142	-4.068743485	0.000466851	0.043930581
cg09591065	-0.615452699	0.001682407	-4.068427272	0.00046722	0.043947053
cg02318560	-0.671801182	3.349658827	-4.068386327	0.000467268	0.043947053
cg09445158	-0.594008711	2.224413739	-4.068317854	0.000467348	0.043947053
cg17290573	-0.597805773	2.537657448	-4.06828172	0.00046739	0.043947053
cg04520189	-0.672783157	2.848988712	-4.068211255	0.000467472	0.043947053
cg23982392	-0.695715919	1.408960343	-4.068148863	0.000467545	0.043947053
cg10438236	-0.555193598	3.697771058	-4.068007095	0.00046771	0.043947053
cg24139233	-0.503982632	3.143266802	-4.068003206	0.000467715	0.043947053
cg12950971	-0.471165798	2.95367478	-4.067938512	0.00046779	0.043947053
cg07438778	-0.62187891	2.853280791	-4.067910874	0.000467823	0.043947053
cg19818294	-0.461776261	4.10724339	-4.067910541	0.000467823	0.043947053
cg08162687	-0.521265138	3.241913418	-4.067866582	0.000467874	0.043947053
cg19083007	-0.805450922	-1.413051398	-4.067858252	0.000467884	0.043947053
cg22967111	-0.664861414	3.098505277	-4.067849167	0.000467895	0.043947053
cg22428623	-0.626623031	3.35328217	-4.067716926	0.000468049	0.043955737
cg04331604	-0.639902704	3.01838626	-4.067473999	0.000468333	0.043976575
cg26294369	-0.536982099	3.037738009	-4.067287063	0.000468552	0.043982808
cg00006090	-0.40938815	3.507531616	-4.067274173	0.000468567	0.043982808
cg12209494	-0.4932065	0.096769908	-4.067169853	0.000468689	0.043982808
cg08904630	-0.458241829	0.088218599	-4.067045646	0.000468834	0.043982808
cg05990479	-0.5859706	3.190862405	-4.067033463	0.000468849	0.043982808
cg11554388	0.664037212	3.843330132	4.066967342	0.000468926	0.043982808
cg17167159	-0.513723247	2.721781723	-4.066966104	0.000468927	0.043982808
cg15130073	-0.583124872	3.155717599	-4.066941235	0.000468957	0.043982808
cg25759466	-0.626896283	2.755711585	-4.066939692	0.000468958	0.043982808
cg09349466	-0.440290374	3.534526901	-4.066744016	0.000469188	0.043998475
cg04562396	-0.928868053	-5.212961045	-4.066591643	0.000469366	0.044003896
cg22181120	-0.65771837	2.34915596	-4.066588632	0.00046937	0.044003896
cg14633252	-0.41696052	-1.23067712	-4.066487101	0.000469489	0.044009228
cg07433533	-0.574251257	3.793349277	-4.066392138	0.0004696	0.044012684
cg14873739	-0.497351224	-2.68348136	-4.066349655	0.00046965	0.044012684
cg16343129	-0.511092907	-1.988521736	-4.066271888	0.000469741	0.044014357
cg19173502	-0.483491828	2.358837996	-4.066228468	0.000469792	0.044014357
cg06340382	-0.505633442	2.649850826	-4.066016427	0.000470041	0.044023215
cg16202611	-0.48452306	-1.571827899	-4.066001528	0.000470058	0.044023215
cg17502527	-0.413506653	3.679886309	-4.065988983	0.000470073	0.044023215
cg17979458	-0.480549125	4.065565839	-4.065869284	0.000470213	0.044025369
cg18090431	-0.607030015	3.25381702	-4.06586349	0.00047022	0.044025369

cg26346574	-0.426689882	3.515376812	-4.065697528	0.000470415	0.044034397
cg16890093	0.533165705	1.788123233	4.065606665	0.000470522	0.044034397
cg16805805	-0.626029077	3.676767716	-4.065603235	0.000470526	0.044034397
cg14349667	-0.61947621	-2.475343117	-4.065569661	0.000470565	0.044034397
cg12969533	-0.901090394	2.760795903	-4.065257847	0.000470932	0.044051676
cg17612310	-0.531346095	3.966790571	-4.06519106	0.00047101	0.044051676
cg05698652	-0.532159371	3.871821601	-4.065168144	0.000471037	0.044051676
cg04011825	-0.875025637	1.001963187	-4.06515421	0.000471054	0.044051676
cg20599009	-0.512313515	3.297147805	-4.065148046	0.000471061	0.044051676
cg11691124	-0.421401138	4.086999892	-4.065013962	0.000471218	0.044051967
cg26610739	-0.516624659	-0.597404407	-4.065001288	0.000471233	0.044051967
cg08568970	-0.84270056	2.491324319	-4.064986797	0.00047125	0.044051967
cg19697042	-0.410729664	-1.847109536	-4.064932037	0.000471315	0.044052025
cg01526173	-0.649413347	1.792046235	-4.064880569	0.000471375	0.044052025
cg05564186	-0.697473172	2.324764334	-4.064701307	0.000471586	0.044058964
cg07689123	-0.447408501	-0.394090148	-4.064694166	0.000471595	0.044058964
cg23879937	-0.346936265	0.645265401	-4.06464961	0.000471647	0.044058964
cg11576590	-0.54720413	2.609378058	-4.06460617	0.000471698	0.044058964
cg09126203	-0.622342737	-1.404922803	-4.064525886	0.000471793	0.044061986
cg11950460	-0.673305145	4.096160576	-4.064325197	0.000472029	0.044075622
cg04251347	-0.73767321	1.983599416	-4.064285921	0.000472076	0.044075622
cg16833110	-0.60517056	3.619042106	-4.064243551	0.000472126	0.044075622
cg10971048	-0.324383954	3.979633436	-4.064127624	0.000472262	0.04408257
cg26467471	-0.820966116	0.485017873	-4.064056488	0.000472346	0.044084591
cg02662281	-0.452691347	0.023761395	-4.063984763	0.000472431	0.044086677
cg21407701	-0.495122247	3.195929888	-4.063877863	0.000472557	0.044089248
cg02582978	-0.491323455	3.455935619	-4.063757536	0.000472699	0.044089248
cg22917995	-0.6168436	2.873513625	-4.063707753	0.000472758	0.044089248
cg14491890	-0.479786164	3.851854934	-4.06365122	0.000472824	0.044089248
cg19084281	-0.558147717	3.524208727	-4.063634308	0.000472844	0.044089248
cg00456581	-0.452706544	2.725530132	-4.063582241	0.000472906	0.044089248
cg04711454	-0.732223258	-1.574619347	-4.063468379	0.00047304	0.044089248
cg18657579	-0.404907846	3.034058623	-4.063446902	0.000473066	0.044089248
cg15390010	-0.676643296	2.799183235	-4.06336792	0.000473159	0.044089248
cg05424932	-0.745249605	-1.084386491	-4.063293558	0.000473247	0.044089248
cg06214653	-0.474641353	-1.790694592	-4.0632176	0.000473337	0.044089248
cg09523859	-0.625520057	-0.843021001	-4.063094198	0.000473482	0.044089248
cg11679609	-0.470858356	-1.292663437	-4.06302893	0.00047356	0.044089248
cg03451760	-0.304370323	3.72536677	-4.063018916	0.000473571	0.044089248
cg04141574	-0.506445889	1.017693303	-4.063009524	0.000473582	0.044089248
cg25926515	-0.450210562	-0.980470956	-4.062980681	0.000473617	0.044089248
cg03539481	-0.697379174	2.416285846	-4.062886353	0.000473728	0.044089248
cg20325185	-0.506901259	3.823819737	-4.062879609	0.000473736	0.044089248
cg24549158	-0.754914537	3.182264558	-4.062813211	0.000473815	0.044089248
cg22934258	-0.400071464	4.160782403	-4.062797413	0.000473833	0.044089248
cg00884047	-0.651582794	3.351235105	-4.062787555	0.000473845	0.044089248
cg10643493	-0.888969856	3.45725245	-4.062750712	0.000473889	0.044089248

cg21342536	-0.80335063	2.263771334	-4.062730638	0.000473912	0.044089248
cg10137381	-0.437897596	3.439511248	-4.062696978	0.000473952	0.044089248
cg18970250	-0.471150058	3.514663322	-4.062576561	0.000474095	0.044092318
cg05821075	-0.517614449	1.99967596	-4.062563899	0.00047411	0.044092318
cg19254118	-0.532105399	-0.064347451	-4.062438188	0.000474258	0.044092385
cg08014100	-0.482285022	3.978378131	-4.062429498	0.000474269	0.044092385
cg22828592	-0.65666447	2.280541147	-4.062347184	0.000474366	0.044092385
cg09655887	-0.497761266	3.639574255	-4.062318179	0.000474401	0.044092385
cg03226511	-0.492186749	3.544946438	-4.062300434	0.000474422	0.044092385
cg27386033	-0.618603644	3.525454318	-4.061979363	0.000474802	0.044110924
cg24702873	-0.623166157	2.859995293	-4.061937156	0.000474852	0.044110924
cg01546857	-0.58194632	3.173631896	-4.061851013	0.000474954	0.044110924
cg17844001	-0.459478723	0.421309803	-4.061632878	0.000475213	0.044110924
cg24093964	-0.437087877	3.685275916	-4.061574164	0.000475282	0.044110924
cg10024916	-0.437063395	3.525474976	-4.061565228	0.000475293	0.044110924
cg02613338	-0.434841967	3.780911912	-4.061554165	0.000475306	0.044110924
cg07112755	-0.631065075	2.576438727	-4.061551056	0.00047531	0.044110924
cg14623600	-0.747889519	3.39457993	-4.061548694	0.000475313	0.044110924
cg05697394	-0.560990666	3.234181518	-4.06154143	0.000475321	0.044110924
cg21877464	-0.459532431	-0.128279517	-4.061518142	0.000475349	0.044110924
cg20598190	-0.6453878	1.228475222	-4.06150188	0.000475368	0.044110924
cg07358196	-0.390931003	3.756736914	-4.061425192	0.000475459	0.044113592
cg10581876	-0.511888394	1.952118407	-4.061223551	0.000475699	0.044121292
cg17144988	-0.560039451	3.52750988	-4.061205334	0.00047572	0.044121292
cg13848990	-0.492893353	3.896056482	-4.061151666	0.000475784	0.044121292
cg07729325	-0.525126103	2.649351752	-4.061098771	0.000475847	0.044121292
cg26238679	-0.424768762	3.114948905	-4.061017263	0.000475944	0.044121292
cg19367193	-0.536140664	3.466641943	-4.060975411	0.000475993	0.044121292
cg15569988	-0.508839762	3.05922732	-4.06097049	0.000475999	0.044121292
cg07230676	-0.429823857	3.580473104	-4.060882205	0.000476104	0.044121292
cg12316559	-0.665057248	1.989991516	-4.060841408	0.000476153	0.044121292
cg19361615	-0.632951001	2.811146562	-4.060830956	0.000476165	0.044121292
cg24776427	-0.433519113	3.912488576	-4.060712624	0.000476306	0.044128556
cg12014113	-0.414848345	3.786961631	-4.060598865	0.000476441	0.044135317
cg15839831	-0.497988742	3.241807725	-4.060539345	0.000476512	0.044136103
cg21649848	-0.590898409	3.454307126	-4.060336384	0.000476753	0.044152698
cg23936512	-0.764574593	2.253609824	-4.060002133	0.000477151	0.044169395
cg03770549	-0.563487553	3.286003302	-4.05992818	0.000477239	0.044169395
cg05552343	-0.491199888	3.094456723	-4.059908847	0.000477262	0.044169395
cg21820610	-0.815420751	1.923152205	-4.059808257	0.000477382	0.044169395
cg25211941	-0.471565887	-1.136968391	-4.059730762	0.000477475	0.044169395
cg23443115	-0.499963416	3.326582026	-4.059728885	0.000477477	0.044169395
cg23420064	-0.499491193	3.977820613	-4.059719232	0.000477488	0.044169395
cg06646906	-0.548988891	3.043317485	-4.059713656	0.000477495	0.044169395
cg13309716	-0.521613269	3.311540943	-4.05965193	0.000477569	0.044169395
cg12959520	-0.650171953	0.417730217	-4.059618128	0.000477609	0.044169395
cg22508905	-0.668241743	3.511290702	-4.059492114	0.000477759	0.044169395

cg11788233	-0.643220509	2.99962104	-4.059461285	0.000477796	0.044169395
cg16292885	-0.358324839	-0.767701467	-4.05934646	0.000477933	0.044169395
cg17315703	-0.546876459	-0.07851646	-4.059341194	0.000477939	0.044169395
cg21649668	-1.217352578	2.715243039	-4.059314718	0.000477971	0.044169395
cg02631785	-0.568162266	3.354640969	-4.059282536	0.000478009	0.044169395
cg11945297	-0.335595452	0.348847544	-4.059244602	0.000478055	0.044169395
cg13406593	-0.311346952	-0.342943374	-4.059243355	0.000478056	0.044169395
cg12446447	-0.585608537	1.046255853	-4.059113429	0.000478211	0.044172963
cg16262572	0.487762012	1.754737602	4.059106511	0.000478219	0.044172963
cg06622408	-0.459268381	-2.15927178	-4.058977699	0.000478373	0.044181408
cg20153175	-0.55410698	3.965858439	-4.058843304	0.000478534	0.044185597
cg20676047	0.733004541	1.951409342	4.058806414	0.000478578	0.044185597
cg05157351	-0.496390274	2.825058997	-4.058745204	0.000478651	0.044185597
cg22351426	-0.487505738	3.455432567	-4.058722304	0.000478678	0.044185597
cg21883245	-0.554964776	3.457594751	-4.058674057	0.000478736	0.044185597
cg11371382	-0.65893887	3.072246411	-4.058626468	0.000478793	0.044185597
cg25732399	-0.588077033	1.295727197	-4.058564664	0.000478867	0.04418631
cg14063129	-0.445844102	-0.832755224	-4.05851564	0.000478925	0.04418631
cg27247219	-1.083792523	1.299648521	-4.058383219	0.000479084	0.044195163
cg09119416	-0.412812935	4.470652646	-4.058094684	0.000479429	0.044212895
cg27455595	-0.500245967	-0.164515539	-4.058092246	0.000479432	0.044212895
cg19745314	-0.876505486	-3.33071862	-4.05789137	0.000479672	0.044212895
cg02438965	-0.582563548	3.565120817	-4.057861373	0.000479708	0.044212895
cg22457860	-0.75377576	3.164963289	-4.057827406	0.000479749	0.044212895
cg00793072	-0.331768225	4.242516707	-4.057825342	0.000479751	0.044212895
cg01791398	-0.476542666	3.695406093	-4.05782534	0.000479751	0.044212895
cg26889408	-0.357481007	0.337890915	-4.057768539	0.000479819	0.044212895
cg27447296	0.584294498	-3.255662218	4.057753277	0.000479838	0.044212895
cg23934163	-0.502359414	0.058140361	-4.057686591	0.000479917	0.044214505
cg24169875	-0.330048544	0.066874099	-4.057563686	0.000480065	0.04422232
cg16754631	-0.51788408	1.235453625	-4.057483797	0.00048016	0.044225389
cg27235888	-0.445544962	4.019429955	-4.057335116	0.000480339	0.044231351
cg22784060	-0.420665246	4.40580668	-4.057325634	0.00048035	0.044231351
cg21050025	-0.42542042	4.266633295	-4.057195877	0.000480506	0.044237744
cg09256180	-0.380375748	0.647880276	-4.057130079	0.000480585	0.044237744
cg14085797	-0.467936298	3.743280773	-4.057004812	0.000480735	0.044237744
cg20182292	-0.710769184	2.539059782	-4.056895584	0.000480866	0.044237744
cg07269756	-0.422787523	2.219076914	-4.056679859	0.000481125	0.044237744
cg15145767	-0.422444124	-0.971624276	-4.056630628	0.000481184	0.044237744
cg01238359	-0.624479719	2.393423397	-4.056617753	0.0004812	0.044237744
cg25051180	-0.687299708	2.00683543	-4.056500799	0.00048134	0.044237744
cg06244510	-0.456870928	3.593083729	-4.056392962	0.00048147	0.044237744
cg25502267	-0.731307164	-0.437081943	-4.056359581	0.00048151	0.044237744
cg11213655	-0.763431674	2.457794513	-4.056318965	0.000481559	0.044237744
cg10114550	-0.469811547	-0.049893191	-4.056313903	0.000481565	0.044237744
cg22000795	-0.541248185	2.647034914	-4.056263844	0.000481625	0.044237744
cg27039868	-0.664706867	3.377760684	-4.056147148	0.000481765	0.044237744

cg17276002	0.549759394	0.041125484	4.056147071	0.000481765	0.044237744
cg24929479	-0.588689897	2.968044536	-4.056009076	0.000481931	0.044237744
cg08025214	-0.453261946	1.863834542	-4.055935443	0.00048202	0.044237744
cg17808825	-0.547404357	3.420447781	-4.055911621	0.000482048	0.044237744
cg08171081	-0.440741777	3.910125029	-4.055860188	0.00048211	0.044237744
cg07169618	-0.669380787	3.138110944	-4.055858375	0.000482113	0.044237744
cg07838100	0.452782276	3.982518499	4.055814074	0.000482166	0.044237744
cg03103533	-0.42872572	-1.23878593	-4.055813057	0.000482167	0.044237744
cg03039050	-0.468161748	4.06008212	-4.055809796	0.000482171	0.044237744
cg03426349	-0.932302205	2.417954069	-4.055789413	0.000482196	0.044237744
cg12295892	-0.633206267	3.452301794	-4.055671107	0.000482338	0.044237744
cg11050480	-0.488587008	2.821831879	-4.055638787	0.000482377	0.044237744
cg22891390	-0.665976798	2.211962956	-4.055590546	0.000482435	0.044237744
cg17956002	-0.379369516	3.719073892	-4.055434982	0.000482622	0.044237744
cg14412164	-0.390268197	-1.401736202	-4.055366899	0.000482704	0.044237744
cg12352038	-0.562396127	-2.891357754	-4.055329463	0.000482749	0.044237744
cg06725035	-0.66918567	-1.207237018	-4.055325202	0.000482755	0.044237744
cg07078958	-0.492957701	-1.028458014	-4.055290932	0.000482796	0.044237744
cg11330512	-0.515045957	0.717694767	-4.055261912	0.000482831	0.044237744
cg21630292	-0.509480135	3.888012981	-4.055103259	0.000483022	0.044237744
cg11920406	-0.358345935	0.723582103	-4.055058826	0.000483076	0.044237744
cg06179710	-0.560605874	1.989904214	-4.054999026	0.000483148	0.044237744
cg18855269	-0.830087615	-0.812464793	-4.054958358	0.000483197	0.044237744
cg05722371	-0.762395443	3.30294202	-4.054903093	0.000483264	0.044237744
cg24205793	-0.660657947	0.128138997	-4.05486469	0.00048331	0.044237744
cg13655908	-0.375616043	2.907033742	-4.054824858	0.000483358	0.044237744
cg05970496	-0.677890048	2.849746356	-4.054812233	0.000483373	0.044237744
cg19768372	-0.311289237	-0.048099061	-4.054711891	0.000483494	0.044237744
cg07763787	-0.441097829	4.294832351	-4.05468059	0.000483532	0.044237744
cg15721764	-0.492937627	-0.932703755	-4.054605266	0.000483623	0.044237744
cg04544858	-0.625489672	-1.726747183	-4.054563393	0.000483673	0.044237744
cg17565632	-0.412276451	3.664400043	-4.054558413	0.000483679	0.044237744
cg00584885	-0.732493522	3.095059436	-4.054482426	0.000483771	0.044237744
cg17264563	-0.368662673	-0.162726967	-4.054420954	0.000483845	0.044237744
cg25255802	-0.464291462	3.568097051	-4.054233739	0.000484072	0.044237744
cg06736024	-0.788599092	2.468444242	-4.054179589	0.000484137	0.044237744
cg19861486	-0.499658428	0.88514839	-4.054116772	0.000484213	0.044237744
cg13606421	-0.64208788	-2.706575276	-4.054067925	0.000484272	0.044237744
cg27137836	-0.469864173	0.555745655	-4.054003485	0.00048435	0.044237744
cg26810925	-0.5375127	3.341939487	-4.053939638	0.000484427	0.044237744
cg20324207	-0.686258748	2.846818624	-4.053931742	0.000484437	0.044237744
cg02520133	-0.943195581	1.780503781	-4.053894124	0.000484482	0.044237744
cg04727302	-0.498639184	3.464926507	-4.053831953	0.000484557	0.044237744
cg19205544	-0.415639473	3.429798807	-4.053826832	0.000484564	0.044237744
cg26922511	-0.537510587	3.613604316	-4.053782902	0.000484617	0.044237744
cg10011182	-0.487832531	0.103010379	-4.053768506	0.000484634	0.044237744
cg10387662	-0.604800524	1.182610083	-4.053763361	0.00048464	0.044237744

cg14471330	-0.37684407	-0.368301084	-4.053756457	0.000484649	0.044237744
cg26855406	-0.517141982	3.465376164	-4.053744662	0.000484663	0.044237744
cg14956679	-0.539666025	2.852283093	-4.053699593	0.000484717	0.044237744
cg08657541	-0.799418878	2.51191934	-4.053698067	0.000484719	0.044237744
cg09977452	-0.432368833	2.97538814	-4.053659182	0.000484766	0.044237744
cg19031301	-0.586347195	3.711740235	-4.05361525	0.000484819	0.044237744
cg17687451	-0.525943447	3.396618813	-4.053565187	0.00048488	0.044237744
cg12589188	-0.667440442	-1.00577005	-4.053333582	0.00048516	0.044237744
cg17445948	-0.543038595	3.427884624	-4.053328891	0.000485166	0.044237744
cg13419549	-0.459554479	3.374690125	-4.053317828	0.00048518	0.044237744
cg27142924	0.761233976	-1.590285257	4.053303446	0.000485197	0.044237744
cg03658834	-0.538367822	2.987068768	-4.053264445	0.000485244	0.044237744
cg13997626	-0.461769825	-0.457998886	-4.053056126	0.000485497	0.044237744
cg19582233	-0.726697085	2.901360235	-4.052951858	0.000485623	0.044237744
cg08017142	-0.552485183	3.638229389	-4.052917747	0.000485664	0.044237744
cg03741623	-0.496382149	0.197397237	-4.052868265	0.000485724	0.044237744
cg00618626	-0.474579026	0.369819795	-4.052848029	0.000485749	0.044237744
cg09665844	-0.684262205	1.891552389	-4.052809311	0.000485796	0.044237744
cg00413403	-0.462049007	3.772851779	-4.052751681	0.000485866	0.044237744
cg07405806	-0.450366889	3.759329996	-4.052726096	0.000485897	0.044237744
cg04216778	-0.53865567	2.832830412	-4.052680775	0.000485952	0.044237744
cg09996077	-0.792585439	2.305027043	-4.052644029	0.000485996	0.044237744
cg25613101	0.517773783	-3.825387504	4.052573061	0.000486082	0.044237744
cg18376817	-0.415618235	-0.488314655	-4.052529905	0.000486135	0.044237744
cg02100965	-0.461139988	3.103649855	-4.052466893	0.000486211	0.044237744
cg15264601	-0.609715633	0.369278457	-4.052438936	0.000486245	0.044237744
cg19257200	-0.452487537	-0.444006571	-4.052380186	0.000486316	0.044237744
cg17388924	-0.478233793	-0.09982665	-4.052339186	0.000486366	0.044237744
cg16768966	-0.46717957	-0.802207024	-4.052320227	0.000486389	0.044237744
cg20178757	-0.628950134	3.195817734	-4.052311723	0.000486399	0.044237744
cg06470440	-0.559425953	4.044955967	-4.052193914	0.000486542	0.044237744
cg18496318	0.375897162	0.909857915	4.052082214	0.000486678	0.044237744
cg18453665	-0.656961619	3.115091454	-4.052050455	0.000486717	0.044237744
cg22936208	-0.392467024	-0.803555645	-4.052028013	0.000486744	0.044237744
cg07604117	-0.581875986	-1.290096798	-4.05196235	0.000486824	0.044237744
cg14588738	-0.420733645	3.837088055	-4.051952165	0.000486836	0.044237744
cg22851295	-0.32496419	-0.694442465	-4.051878354	0.000486926	0.044237744
cg07036677	-0.693642219	3.198258881	-4.05186276	0.000486945	0.044237744
cg22507167	-0.449202905	3.575147751	-4.0517753	0.000487051	0.044237744
cg22646785	-0.771006648	2.823777344	-4.051762595	0.000487067	0.044237744
cg14129217	-0.877281432	-1.351378212	-4.051756613	0.000487074	0.044237744
cg01364654	-0.526242878	3.411356635	-4.051745467	0.000487087	0.044237744
cg23992560	-0.348161397	3.9382976	-4.051722013	0.000487116	0.044237744
cg17417595	-0.507524712	3.817777979	-4.051719472	0.000487119	0.044237744
cg00988881	-0.407694658	-0.406054301	-4.051635833	0.000487221	0.044237744
cg10519935	-0.767406266	-0.842170557	-4.051558171	0.000487315	0.044237744
cg08911536	-0.535330926	2.78376098	-4.051530126	0.000487349	0.044237744

cg05381829	-0.958372832	2.286420942	-4.05145478	0.000487441	0.044237744
cg15927031	0.591367431	-4.592273224	4.051418463	0.000487485	0.044237744
cg18734657	-0.477456538	0.567215036	-4.051411647	0.000487493	0.044237744
cg18824724	-0.545204766	1.300626908	-4.051261181	0.000487677	0.044237744
cg11869049	-0.553905765	2.961890983	-4.051219566	0.000487727	0.044237744
cg01249024	-0.39506147	-0.16514521	-4.051141327	0.000487822	0.044237744
cg06059069	-0.372736344	-0.193645333	-4.051120569	0.000487848	0.044237744
cg01039257	-0.470397274	3.427202014	-4.051102688	0.000487869	0.044237744
cg27427522	-0.644654538	2.533420526	-4.051085986	0.00048789	0.044237744
cg14413614	0.453084489	2.607110513	4.051062544	0.000487918	0.044237744
cg04270337	-0.566983048	3.394038525	-4.051049538	0.000487934	0.044237744
cg02642549	-0.667416119	1.550383203	-4.051013195	0.000487978	0.044237744
cg23915591	-0.382853452	3.260142493	-4.050974109	0.000488026	0.044237744
cg04454215	-0.628656368	2.743091616	-4.050964136	0.000488038	0.044237744
cg11356706	-0.578180839	-1.955084172	-4.05087745	0.000488144	0.044241655
cg00734395	-0.544796576	3.560928499	-4.050812729	0.000488223	0.044242972
cg06499214	-0.623446342	2.601036481	-4.050749938	0.000488299	0.044242972
cg07478210	-0.440911833	-1.566704901	-4.05069529	0.000488366	0.044242972
cg26124136	-0.590244525	3.50345662	-4.05065177	0.000488419	0.044242972
cg25163933	-0.330170614	3.78602239	-4.050609297	0.000488471	0.044242972
cg19416290	-0.433471592	3.19970685	-4.050540123	0.000488555	0.044244954
cg25966829	-0.431139007	4.154432493	-4.050475505	0.000488634	0.044246434
cg21534931	-0.465553775	-0.180469874	-4.050371135	0.000488761	0.044247596
cg06419268	-0.439912955	-0.840244238	-4.050358754	0.000488776	0.044247596
cg03810769	-0.388203313	-0.448171795	-4.050311364	0.000488834	0.044247596
cg16313815	-0.367750305	4.432579293	-4.050149694	0.000489031	0.044252133
cg23738173	-0.573982196	3.0986062	-4.050095511	0.000489097	0.044252133
cg07183577	-0.415453938	3.989289559	-4.050088963	0.000489105	0.044252133
cg18105749	-0.417425791	-1.637992285	-4.050065548	0.000489134	0.044252133
cg20628003	-0.42636144	-0.346327982	-4.049986035	0.000489231	0.044255264
cg10900504	-0.496640311	3.448593743	-4.049907524	0.000489327	0.044256607
cg15627300	-0.677257404	1.98550393	-4.049825763	0.000489427	0.044256607
cg19608409	-0.78855515	1.998382857	-4.049770386	0.000489494	0.044256607
cg05217047	-0.426325851	-0.644310167	-4.049769274	0.000489496	0.044256607
cg00145303	-0.532202657	4.076771152	-4.049472573	0.000489858	0.044261258
cg16228716	-0.526907728	3.068770893	-4.049377885	0.000489974	0.044261258
cg01343936	-0.570597822	1.235628244	-4.049355083	0.000490002	0.044261258
cg20760676	-0.49009223	4.516499915	-4.049306768	0.000490061	0.044261258
cg07536419	-0.541289663	3.313581764	-4.049299115	0.000490071	0.044261258
cg03122695	-0.484833765	3.263523716	-4.049246138	0.000490135	0.044261258
cg08585862	-0.824179682	2.483913687	-4.049203111	0.000490188	0.044261258
cg01207650	-0.437282291	0.27994312	-4.049177815	0.000490219	0.044261258
cg00651876	-0.429021471	3.958135376	-4.049044299	0.000490382	0.044261258
cg13543189	-0.535821216	3.094851612	-4.049040483	0.000490387	0.044261258
cg08161491	-0.562246278	-0.183486892	-4.049030101	0.0004904	0.044261258
cg13380325	-0.486152346	3.284478424	-4.048969017	0.000490474	0.044261258
cg14484885	-0.613693627	-0.726641924	-4.048968012	0.000490476	0.044261258

cg03603035	-0.705320915	2.814741489	-4.048923475	0.00049053	0.044261258
cg20496695	-0.696575163	3.431304366	-4.048796216	0.000490686	0.044261258
cg16104831	-0.376641294	-1.919434829	-4.048792053	0.000490691	0.044261258
cg03012734	-0.565637256	2.987141655	-4.048700736	0.000490803	0.044261258
cg08148659	-0.76899063	2.424500786	-4.048675353	0.000490834	0.044261258
cg12206840	-0.547207782	0.302984922	-4.048661898	0.000490851	0.044261258
cg09837995	-0.552160783	0.573645245	-4.048649525	0.000490866	0.044261258
cg15229245	-0.791705002	3.169536811	-4.048625046	0.000490896	0.044261258
cg12551751	-0.576489055	3.430610551	-4.048572829	0.00049096	0.044261258
cg04981608	-0.591555845	3.938008246	-4.048552699	0.000490984	0.044261258
cg13433596	-0.360227474	4.189403064	-4.048359905	0.000491221	0.044276926
cg19800801	-0.483932454	3.410951929	-4.048279173	0.00049132	0.044280214
cg10602561	-0.54724895	3.299065098	-4.047998459	0.000491664	0.044295356
cg15720112	-0.551169868	0.396507175	-4.047972087	0.000491696	0.044295356
cg15027190	-0.405506748	3.585166345	-4.047944162	0.000491731	0.044295356
cg07688771	-0.470534109	3.949592233	-4.047869224	0.000491823	0.044295356
cg20895750	-0.596177303	3.32113462	-4.04780855	0.000491897	0.044295356
cg16784006	-0.555816161	3.066100729	-4.04770561	0.000492023	0.044295356
cg23381267	-0.454113364	3.69610151	-4.047675023	0.000492061	0.044295356
cg16448423	-0.615441939	3.749633074	-4.047465195	0.000492319	0.044295356
cg01425718	-0.670784874	2.766350099	-4.047368237	0.000492438	0.044295356
cg16991351	-0.510129162	3.499436357	-4.047333656	0.00049248	0.044295356
cg00929655	-0.399551798	3.841330253	-4.047276209	0.000492551	0.044295356
cg10455053	-0.339066416	4.060982957	-4.04714564	0.000492712	0.044295356
cg20398091	-0.520709537	-1.543346399	-4.047140753	0.000492718	0.044295356
cg01967537	-0.707895894	2.959842545	-4.047135722	0.000492724	0.044295356
cg21410960	-0.686165247	-0.352550081	-4.047132848	0.000492727	0.044295356
cg07483620	-0.450600869	3.970621939	-4.047122826	0.00049274	0.044295356
cg19375044	0.644124015	-4.592478617	4.047094165	0.000492775	0.044295356
cg10100266	-0.604840516	2.780199658	-4.047052909	0.000492826	0.044295356
cg21761538	-0.442250764	3.595702209	-4.046989989	0.000492903	0.044295356
cg18978531	-0.550982883	-2.079432434	-4.04694884	0.000492954	0.044295356
cg17186418	0.618342535	1.151090514	4.046889757	0.000493026	0.044295356
cg14492992	0.63253967	2.732326444	4.046874977	0.000493044	0.044295356
cg12199057	-0.60073506	0.382046327	-4.046849442	0.000493076	0.044295356
cg06409742	-0.723905915	2.634363499	-4.046840995	0.000493086	0.044295356
cg15895268	-0.521910653	3.942446911	-4.046821466	0.00049311	0.044295356
cg00162517	-0.535956353	-0.494187833	-4.046818942	0.000493113	0.044295356
cg25508419	-0.482615392	0.736927383	-4.046565954	0.000493425	0.044302334
cg16692757	-0.358806902	-0.612078475	-4.046561101	0.000493431	0.044302334
cg17318406	-0.98697346	1.620607915	-4.046557275	0.000493436	0.044302334
cg07788038	-0.572860944	0.020917914	-4.04654491	0.000493451	0.044302334
cg20224780	-0.839646352	2.003386586	-4.046501857	0.000493504	0.044302334
cg27560898	-0.754006992	2.239057089	-4.046421654	0.000493603	0.044304643
cg01512757	-0.863974729	-1.773940952	-4.046352446	0.000493688	0.044304643
cg03979788	-0.696767316	-0.023626612	-4.04632866	0.000493717	0.044304643
cg00449798	-0.584794114	-3.395127562	-4.046081656	0.000494022	0.044322147

cg22349718	-0.754079202	2.363007802	-4.046068881	0.000494037	0.044322147
cg14153437	-0.819235132	3.747624909	-4.045831628	0.00049433	0.044342782
cg08641302	0.6515151	2.325858929	4.045694642	0.000494499	0.044352328
cg06415313	-0.746167264	3.159528682	-4.045546859	0.000494681	0.044363071
cg04882147	-0.746898135	2.342395208	-4.045466092	0.000494781	0.044366363
cg01323312	-0.478704148	-2.652301486	-4.045415685	0.000494843	0.044366363
cg01958256	-0.451669987	4.051859444	-4.045275662	0.000495016	0.044367901
cg22416262	-0.557840506	3.001741517	-4.045263797	0.000495031	0.044367901
cg21329660	-0.767515391	2.043941294	-4.045249679	0.000495048	0.044367901
cg15536175	-0.482617108	4.139854585	-4.044909949	0.000495468	0.044399916
cg19300124	-0.796828706	2.639899766	-4.044707614	0.000495719	0.044402442
cg19638398	0.420864349	-3.05630507	4.044698337	0.00049573	0.044402442
cg15552882	-0.702142855	1.29714213	-4.044670462	0.000495765	0.044402442
cg10778005	-0.385192546	3.725331295	-4.044664941	0.000495771	0.044402442
cg12899614	-0.447669062	4.246460771	-4.044633776	0.00049581	0.044402442
cg15189349	-0.495888921	3.525780776	-4.044395477	0.000496105	0.04442324
cg14701415	-0.369806115	4.101241229	-4.043995541	0.0004966	0.044442807
cg26644914	-0.396808992	3.31152635	-4.04397241	0.000496629	0.044442807
cg13028856	-0.505799789	-1.023064049	-4.043916441	0.000496698	0.044442807
cg16255988	-0.729861285	2.204459352	-4.043898009	0.000496721	0.044442807
cg12430137	-0.499428504	-0.939487438	-4.043895955	0.000496724	0.044442807
cg10564180	-0.516398972	0.097525444	-4.04388966	0.000496731	0.044442807
cg27109284	-0.731282349	1.242487636	-4.043850806	0.00049678	0.044442807
cg12881238	-0.526325171	3.837598184	-4.043814	0.000496825	0.044442807
cg15655888	-0.526013778	3.157918088	-4.043660469	0.000497016	0.0444445
cg11566441	-0.397782247	-0.685042172	-4.043658883	0.000497018	0.0444445
cg14458472	-0.607411705	3.056329061	-4.043646953	0.000497032	0.0444445
cg22432612	-0.394371622	4.241902044	-4.043448523	0.000497279	0.044460902
cg22450878	-0.70455217	0.730276937	-4.043379035	0.000497365	0.044463
cg05885717	-0.512338847	3.804478383	-4.043305	0.000497457	0.044465604
cg18346544	-0.523165019	3.070788654	-4.04314059	0.000497661	0.044469614
cg02020454	-0.493983315	3.040245965	-4.04301131	0.000497821	0.044469614
cg11422445	-0.444433907	3.75300174	-4.042953077	0.000497894	0.044469614
cg07877645	-0.508784735	3.992501203	-4.042935791	0.000497915	0.044469614
cg11612555	-0.392513659	3.591945095	-4.042907583	0.00049795	0.044469614
cg10211049	-0.488301918	-0.332894523	-4.042846665	0.000498026	0.044469614
cg05494565	-0.474904219	-1.363150986	-4.042831865	0.000498044	0.044469614
cg06936779	-0.862530542	-1.440979438	-4.042797657	0.000498087	0.044469614
cg02673129	-0.45461667	4.172758496	-4.042783167	0.000498105	0.044469614
cg26579307	-0.614212154	2.969303654	-4.042763547	0.000498129	0.044469614
cg11454877	-0.440909423	-0.877400985	-4.042576924	0.000498361	0.044470259
cg05050991	-0.446442884	3.505826277	-4.042569294	0.000498371	0.044470259
cg07092233	-0.504994357	3.401428124	-4.042547023	0.000498399	0.044470259
cg11335155	-0.666771665	2.27531507	-4.04251298	0.000498441	0.044470259
cg13485721	0.909586098	3.875304096	4.042471977	0.000498492	0.044470259
cg06432753	-0.434035846	0.508751843	-4.042454842	0.000498513	0.044470259
cg14465148	-0.56256443	2.86246671	-4.042258821	0.000498757	0.044486415

cg04646255	-0.427113455	3.899247732	-4.042011047	0.000499066	0.044490358
cg07451928	0.577123794	3.663729481	4.041998172	0.000499082	0.044490358
cg12829325	-0.749808198	-2.508480022	-4.041944693	0.000499148	0.044490358
cg27171704	-0.488688419	-0.31176173	-4.041901887	0.000499202	0.044490358
cg07273847	-0.398199219	3.913292896	-4.041858475	0.000499256	0.044490358
cg00661155	-0.602890802	3.101669053	-4.041822452	0.000499301	0.044490358
cg01386104	-0.473548357	3.943830839	-4.041735197	0.000499409	0.044490358
cg10226546	0.435706311	-0.557860287	4.041714096	0.000499436	0.044490358
cg02671779	-0.562275143	3.216135531	-4.041713797	0.000499436	0.044490358
cg05839425	-0.586442008	2.181053308	-4.0416176	0.000499556	0.044490358
cg06814641	-0.501845708	3.390798903	-4.041599674	0.000499578	0.044490358
cg11247129	-0.467090892	0.275464121	-4.041510986	0.000499689	0.044490358
cg00024937	-0.440859366	-0.175095474	-4.041499741	0.000499703	0.044490358
cg16280552	-0.451391686	3.373364883	-4.041498964	0.000499704	0.044490358
cg18297105	-0.534969198	2.925981604	-4.04146716	0.000499743	0.044490358
cg27509153	-0.537334118	4.238474051	-4.041406184	0.00049982	0.044491537
cg11987700	-0.934562231	1.21605798	-4.041305273	0.000499945	0.044495251
cg00444581	-0.760223655	2.97432134	-4.041272032	0.000499987	0.044495251
cg02736573	-0.432277121	3.456474995	-4.041204201	0.000500072	0.044497193
cg12357419	-0.457035811	3.912190155	-4.041080169	0.000500226	0.044505377
cg17068512	-0.748522122	2.780648677	-4.040903446	0.000500447	0.044507053
cg09484322	-0.786258405	1.336280715	-4.040898422	0.000500453	0.044507053
cg15911263	-0.49884671	4.086044239	-4.040820822	0.00050055	0.044507053
cg09970231	-0.600060797	3.28117452	-4.0408114	0.000500562	0.044507053
cg26971256	-0.503620689	3.496664341	-4.040748434	0.000500641	0.044507053
cg15797527	-0.499242869	3.206323509	-4.040727897	0.000500666	0.044507053
cg11191979	-0.579172861	2.561612149	-4.040712913	0.000500685	0.044507053
cg03592135	-0.628291138	2.911619479	-4.040641949	0.000500774	0.044508146
cg23539837	0.517626766	3.471102868	4.040602514	0.000500823	0.044508146
cg24491045	-0.605647203	2.828734534	-4.040480404	0.000500976	0.04451141
cg13982505	-0.519894785	-0.792547684	-4.040451667	0.000501011	0.04451141
cg00693166	-0.771784267	-1.187866962	-4.040402485	0.000501073	0.04451141
cg23421347	-0.563061459	3.455526779	-4.040364317	0.000501121	0.04451141
cg22106847	-0.543419377	3.511633423	-4.040321827	0.000501174	0.04451141
cg14820240	-0.760202018	2.290676287	-4.040059374	0.000501502	0.044534987
cg00084608	-0.563470218	4.082753278	-4.040000579	0.000501576	0.044535317
cg25642974	-0.480969328	3.438318301	-4.039955945	0.000501632	0.044535317
cg21228301	-0.427447977	0.574165093	-4.039901244	0.0005017	0.044535814
cg27653683	-0.674115927	2.93864	-4.039703151	0.000501948	0.044546682
cg17859594	-0.536687561	3.298765995	-4.039657193	0.000502006	0.044546682
cg04988103	-0.673098088	-2.279191424	-4.039647201	0.000502018	0.044546682
cg06726154	-0.546206116	-2.129862967	-4.039569221	0.000502116	0.044546682
cg12986110	-0.540879941	2.277392783	-4.039476255	0.000502232	0.044546682
cg11417701	-0.450008477	1.093818276	-4.039449687	0.000502266	0.044546682
cg13855435	-0.591578233	-1.808553183	-4.039446606	0.00050227	0.044546682
cg21422983	-0.596830538	2.886963576	-4.039356956	0.000502382	0.044546682
cg25678184	-0.629126594	0.107269511	-4.03930293	0.00050245	0.044546682

cg11572845	-0.670178831	3.620838581	-4.039301585	0.000502451	0.044546682
cg05239887	-0.870771366	2.412534766	-4.039018494	0.000502806	0.044559199
cg16875108	-0.791134547	1.950971888	-4.039015504	0.00050281	0.044559199
cg15686183	-0.875865678	2.024182256	-4.038990061	0.000502842	0.044559199
cg26680851	-0.49053251	3.590030818	-4.03894245	0.000502902	0.044559199
cg27225476	-0.595025456	3.084409268	-4.038938286	0.000502907	0.044559199
cg07775832	-0.627424806	2.613785793	-4.038861468	0.000503003	0.044561944
cg05162236	-0.428293588	3.636158069	-4.038811315	0.000503066	0.044561944
cg22356541	-0.437835607	3.441399761	-4.038691334	0.000503217	0.044561944
cg08754015	-0.687131985	2.858018075	-4.038687343	0.000503222	0.044561944
cg06734985	-0.547219032	3.378433775	-4.038663053	0.000503253	0.044561944
cg02666070	-0.58945566	3.209927407	-4.038547684	0.000503397	0.044567829
cg26629625	-0.507952727	3.045563871	-4.038509955	0.000503445	0.044567829
cg19209911	-0.711068321	4.304712379	-4.038448584	0.000503522	0.044569084
cg22441882	-0.410098752	-0.698692884	-4.038052937	0.000504019	0.044604367
cg25031844	-0.68589824	2.915841113	-4.037982197	0.000504108	0.044604367
cg18338736	-0.632507313	3.184702197	-4.037981247	0.000504109	0.044604367
cg08474147	-0.497974176	3.837602761	-4.037838764	0.000504289	0.044614656
cg18002615	-0.586352984	2.027946238	-4.037680177	0.000504488	0.044624674
cg17716034	-0.860430572	1.838976719	-4.037648691	0.000504528	0.044624674
cg21944455	-0.545889446	-0.301102222	-4.037414363	0.000504823	0.044643602
cg10407763	-0.642296283	2.189581799	-4.037378667	0.000504868	0.044643602
cg17829746	-0.47126	-0.036599927	-4.037280464	0.000504992	0.044648969
cg20948699	-0.544208298	-0.70209208	-4.037096711	0.000505223	0.044651972
cg00477011	-0.538794982	3.460688916	-4.037049843	0.000505282	0.044651972
cg14857363	-0.464825652	4.214129376	-4.037005214	0.000505339	0.044651972
cg15626670	-0.610992082	2.140980028	-4.036978511	0.000505372	0.044651972
cg24373531	-0.63573659	-0.12047358	-4.036719218	0.000505699	0.044651972
cg25748441	0.708977573	0.442515318	4.036712503	0.000505708	0.044651972
cg09995161	0.541688053	4.021592362	4.03666009	0.000505774	0.044651972
cg02794786	-0.750680402	3.037001662	-4.036629247	0.000505813	0.044651972
cg07508304	-0.475028686	-0.396752534	-4.036565663	0.000505893	0.044651972
cg04233620	-0.440522767	3.297083651	-4.036558701	0.000505902	0.044651972
cg07781445	-0.443190093	-1.658229489	-4.036499141	0.000505977	0.044651972
cg15990211	-0.539079034	-3.380013502	-4.036263606	0.000506275	0.044651972
cg13801381	0.784609459	-4.33176097	4.03621037	0.000506342	0.044651972
cg20074593	-0.398702269	-0.435346239	-4.036166192	0.000506398	0.044651972
cg09014910	-0.492336738	4.306730999	-4.036120037	0.000506456	0.044651972
cg03846072	-0.362456276	4.126903976	-4.036005498	0.000506601	0.044651972
cg12381840	-0.502746499	3.235776552	-4.035982814	0.000506629	0.044651972
cg27102995	-0.489690696	2.992144391	-4.03597138	0.000506644	0.044651972
cg07513789	-0.492991354	2.637825575	-4.035955128	0.000506664	0.044651972
cg00277233	-0.535051502	3.346946288	-4.035834319	0.000506817	0.044651972
cg06070290	-0.622578226	2.037028595	-4.035822254	0.000506832	0.044651972
cg05205351	0.354476844	-0.053935477	4.03576489	0.000506905	0.044651972
cg23353544	-0.65552393	3.345427149	-4.035753342	0.00050692	0.044651972
cg14211983	-0.466180907	2.802236286	-4.035704226	0.000506982	0.044651972

cg22571336	-0.624850281	3.339903552	-4.035696276	0.000506992	0.044651972
cg03877689	-0.40943874	-1.95637973	-4.03564003	0.000507063	0.044651972
cg04076713	-0.592982083	3.220263584	-4.035531363	0.0005072	0.044651972
cg21781492	-0.588903409	2.492073741	-4.035445567	0.000507309	0.044651972
cg00359599	-0.500594048	0.283945358	-4.035419096	0.000507343	0.044651972
cg04442913	-0.43093215	2.9327865	-4.035398649	0.000507368	0.044651972
cg24207849	-0.639766713	3.509667924	-4.035398091	0.000507369	0.044651972
cg11520988	-0.594092588	3.908186373	-4.035355745	0.000507423	0.044651972
cg16255513	-0.37975576	-0.26861305	-4.035326122	0.00050746	0.044651972
cg25838204	-0.583891802	3.227718498	-4.035290546	0.000507505	0.044651972
cg02746640	-0.489840664	3.688894407	-4.035286755	0.00050751	0.044651972
cg17115619	-0.467417706	-0.672953137	-4.035283424	0.000507514	0.044651972
cg22127825	-0.61399269	3.345176707	-4.035259617	0.000507544	0.044651972
cg09228052	-0.310549211	4.013205331	-4.03522922	0.000507583	0.044651972
cg20012224	-0.621067534	2.629954078	-4.035217284	0.000507598	0.044651972
cg25377514	-0.623529942	3.038995407	-4.035160494	0.00050767	0.044651972
cg25314989	-0.693788553	2.362235873	-4.035146658	0.000507688	0.044651972
cg15566921	-0.858935785	1.677956043	-4.035131585	0.000507707	0.044651972
cg17828237	-0.440019024	4.217546155	-4.035108307	0.000507736	0.044651972
cg12629189	-0.526593418	-0.23209484	-4.034645636	0.000508323	0.044689041
cg20162468	-0.578078974	2.472831235	-4.034636265	0.000508334	0.044689041
cg01474902	-0.597792292	3.289548569	-4.034626198	0.000508347	0.044689041
cg07483152	-0.766478731	1.060147474	-4.034508187	0.000508497	0.044689041
cg11271406	-0.531539775	3.101491726	-4.034352766	0.000508694	0.044689041
cg22696982	-0.581318549	-4.088635643	-4.0343483	0.0005087	0.044689041
cg25147576	-0.416311105	4.201895506	-4.034286683	0.000508778	0.044689041
cg18864880	-0.653239407	2.853357635	-4.034226533	0.000508854	0.044689041
cg04515572	-0.546283907	0.540645298	-4.034200461	0.000508888	0.044689041
cg15058210	-0.551786923	-1.355047674	-4.03417038	0.000508926	0.044689041
cg22966369	-0.47508234	3.491826696	-4.034160751	0.000508938	0.044689041
cg08260775	-0.353268764	0.973613399	-4.034136868	0.000508968	0.044689041
cg03462706	-0.551600785	3.294416225	-4.034129398	0.000508978	0.044689041
cg03304589	-0.493451399	3.809589028	-4.034049035	0.00050908	0.044692463
cg01104334	-0.465932305	3.526702932	-4.033999106	0.000509143	0.044692492
cg00702126	-0.407868412	-1.203349989	-4.033719433	0.000509499	0.044711265
cg14448116	-0.411486953	0.038834963	-4.033696209	0.000509528	0.044711265
cg10241039	-0.617055664	2.272574146	-4.03363717	0.000509603	0.044711265
cg21842842	-0.708557777	1.454303364	-4.033632179	0.00050961	0.044711265
cg15128842	0.529265434	0.733878669	4.033448828	0.000509843	0.044726184
cg13300939	-0.492666889	-1.014050318	-4.033275966	0.000510063	0.044739937
cg18019037	-0.611179491	3.220801549	-4.033217001	0.000510138	0.04474098
cg09394516	-0.43356204	0.268357267	-4.033089588	0.0005103	0.044742567
cg02046984	0.44077497	1.007267832	4.033069739	0.000510325	0.044742567
cg16622085	-0.581151947	2.851138293	-4.033037466	0.000510366	0.044742567
cg22386425	-0.356680499	0.292445939	-4.032992121	0.000510424	0.044742567
cg11863255	-0.482937322	-0.416476882	-4.032933208	0.000510499	0.044742567
cg04907014	0.588933408	3.176434207	4.032905159	0.000510535	0.044742567

cg00710515	-0.447470209	-0.462923167	-4.032784852	0.000510688	0.044750462
cg22061957	-0.40217826	3.207054642	-4.032654954	0.000510854	0.044756283
cg13195435	-0.627241973	3.111999187	-4.032593449	0.000510932	0.044756283
cg23123128	-0.594494202	3.502901851	-4.032584006	0.000510944	0.044756283
cg05987644	-0.401225733	-0.508508118	-4.032455508	0.000511108	0.044760523
cg26347632	-0.57775834	-1.107428442	-4.032406238	0.000511171	0.044760523
cg14427795	-0.355918928	-1.253192272	-4.03238925	0.000511192	0.044760523
cg08098951	-0.619465617	3.214956825	-4.032347845	0.000511245	0.044760523
cg17705053	-0.792052957	2.198782015	-4.032094658	0.000511568	0.044783071
cg17633463	-0.405975452	-0.774160208	-4.032046921	0.000511629	0.044783071
cg04195625	-0.585322015	3.008189387	-4.031717982	0.000512049	0.044792651
cg14548911	-0.498202374	3.103023777	-4.031711953	0.000512057	0.044792651
cg21144941	-0.666165468	-0.876866369	-4.031644065	0.000512144	0.044792651
cg24053383	0.459162541	1.46735435	4.031570511	0.000512238	0.044792651
cg02253978	-0.487892992	3.048792867	-4.031475336	0.000512359	0.044792651
cg09668627	-0.430713298	-0.241746036	-4.031444248	0.000512399	0.044792651
cg05280088	-0.480107236	3.655944832	-4.031437171	0.000512408	0.044792651
cg22490758	-0.436609665	-0.647040818	-4.031421399	0.000512428	0.044792651
cg07386061	0.486218812	3.020866227	4.031415078	0.000512436	0.044792651
cg03203693	-0.443982629	0.158362466	-4.031373472	0.00051249	0.044792651
cg04997952	-0.523423287	3.733019553	-4.031341413	0.000512531	0.044792651
cg15137282	-0.413815377	-1.867410624	-4.031327498	0.000512548	0.044792651
cg14183988	-0.62993493	3.385705698	-4.031317863	0.000512561	0.044792651
cg08778119	-0.90412562	0.637118044	-4.031111149	0.000512825	0.044797565
cg04018749	-0.7991074	-1.832886744	-4.03108476	0.000512859	0.044797565
cg14042396	-0.542857586	-1.729934855	-4.031044584	0.00051291	0.044797565
cg25213897	-0.48012114	-0.24446509	-4.03103166	0.000512927	0.044797565
cg12239870	-0.555137712	1.643167139	-4.031026728	0.000512933	0.044797565
cg01738164	0.433534956	1.160880475	4.030898942	0.000513097	0.044806325
cg05828609	-0.497776179	3.537993921	-4.030836942	0.000513176	0.044807733
cg05062286	-0.742684833	2.695730934	-4.030748907	0.000513289	0.044812052
cg02989842	-0.471021206	3.47931135	-4.030640528	0.000513428	0.044818647
cg04044188	-0.469449246	-0.852496129	-4.030565478	0.000513524	0.044821516
cg13199219	-0.491662632	3.399585373	-4.03036383	0.000513782	0.044830377
cg04144816	-0.516583248	3.886161396	-4.030310161	0.000513851	0.044830377
cg12469110	-0.400470047	4.263044246	-4.030246391	0.000513933	0.044830377
cg00774579	0.521603293	1.522362915	4.030197095	0.000513996	0.044830377
cg11537479	-0.396599911	-0.530612322	-4.030196362	0.000513997	0.044830377
cg10630028	0.56687354	-4.068694006	4.030154563	0.00051405	0.044830377
cg00872086	-0.649638744	-1.679731325	-4.030140682	0.000514068	0.044830377
cg00242096	-0.665696973	-1.040010176	-4.030050727	0.000514184	0.04483492
cg17555102	-0.456104488	3.97014516	-4.029830256	0.000514466	0.044837623
cg08390352	-0.727341394	-2.523521936	-4.029830069	0.000514467	0.044837623
cg20410062	0.799565668	2.630478023	4.029785733	0.000514524	0.044837623
cg19561607	-0.414814085	3.604211718	-4.029777042	0.000514535	0.044837623
cg19025736	0.480084516	-4.154404952	4.0297125	0.000514618	0.044837623
cg05065559	-0.898525284	2.973800214	-4.029633278	0.000514719	0.044837623

cg10298301	-0.529798925	3.907195337	-4.0296018	0.00051476	0.044837623
cg03185894	-0.686072742	2.861401215	-4.029598206	0.000514764	0.044837623
cg11257888	0.514128569	3.282470624	4.029570664	0.0005148	0.044837623
cg24939330	-0.422729186	4.147818479	-4.029533471	0.000514848	0.044837623
cg09657431	-0.516130625	3.794171218	-4.029436047	0.000514973	0.044837672
cg04557582	-0.477528482	3.032898044	-4.029405253	0.000515012	0.044837672
cg16605030	-0.42838634	-1.008143425	-4.029319339	0.000515123	0.044837672
cg08986516	-0.766698604	2.715614397	-4.029305246	0.000515141	0.044837672
cg16000321	-0.53738921	-1.435618432	-4.029267122	0.00051519	0.044837672
cg02012014	-0.455647153	3.324091536	-4.029164028	0.000515322	0.044837672
cg12554282	-0.536689627	2.818766262	-4.029114392	0.000515386	0.044837672
cg05435671	-0.378035417	3.969155641	-4.029046555	0.000515473	0.044837672
cg14363533	-0.450417192	-1.409402846	-4.029032639	0.000515491	0.044837672
cg11680772	-0.422312694	2.702191133	-4.029019955	0.000515508	0.044837672
cg06158684	-0.412853055	3.393010933	-4.028883764	0.000515683	0.044837672
cg26054396	-0.391859429	4.155007418	-4.028883499	0.000515683	0.044837672
cg02409150	-0.50879097	3.276364984	-4.028867785	0.000515703	0.044837672
cg27480659	-0.464111332	-0.670205839	-4.028808897	0.000515779	0.044837672
cg17911589	-0.759078987	2.295719887	-4.028788315	0.000515806	0.044837672
cg15696658	-0.448221589	0.516364409	-4.028730845	0.000515879	0.044837672
cg20641391	-0.460642544	3.561618401	-4.028696153	0.000515924	0.044837672
cg26519740	-0.578448342	-0.605382929	-4.028452168	0.000516238	0.044859469
cg15241069	0.716209924	3.320470077	4.028384509	0.000516325	0.044861538
cg27060387	-0.353310175	0.597655749	-4.028307405	0.000516425	0.044863582
cg11521979	-0.476152103	3.741323787	-4.028219571	0.000516538	0.044863582
cg13906954	-0.596010609	2.945541413	-4.02821878	0.000516539	0.044863582
cg07077971	-0.441387259	3.015236439	-4.028046962	0.00051676	0.044874613
cg15761531	-0.553377569	3.65565342	-4.027972478	0.000516856	0.044874613
cg22728989	-0.624347444	3.370616123	-4.027933655	0.000516906	0.044874613
cg15573998	-0.41909132	0.095398562	-4.027923691	0.000516919	0.044874613
cg08708750	-0.524828896	3.458583455	-4.027741022	0.000517155	0.044889566
cg14161821	-0.624834089	-1.159871009	-4.027676799	0.000517238	0.044891258
cg27279968	-0.472610657	3.875602825	-4.027592541	0.000517347	0.044895195
cg04951476	-0.429535808	0.144856509	-4.027519301	0.000517441	0.044895877
cg14752861	-0.40189791	3.535823254	-4.027442197	0.000517541	0.044895877
cg16047868	-0.43507752	3.447061825	-4.027384779	0.000517615	0.044895877
cg23167790	-0.414747928	3.762483924	-4.027349659	0.00051766	0.044895877
cg15167212	-0.715361626	3.750597687	-4.027340995	0.000517671	0.044895877
cg14036821	0.717725809	3.238268791	4.027122436	0.000517954	0.044914863
cg17933531	-0.524654448	-0.809678475	-4.027062418	0.000518031	0.04491609
cg24052475	-0.396491221	-0.51574082	-4.027005664	0.000518104	0.044916952
cg14981482	-0.474507924	3.589306528	-4.02685381	0.000518301	0.044923148
cg17328719	0.563649582	2.563515908	4.026848843	0.000518307	0.044923148
cg19805450	-0.456063809	-0.41780835	-4.026796605	0.000518375	0.044923148
cg24376337	-0.4129274	3.464664496	-4.026754193	0.00051843	0.044923148
cg00431235	-0.777934732	-4.983987161	-4.026472234	0.000518794	0.044949259
cg19237818	-0.586394496	3.430592181	-4.026422649	0.000518859	0.044949321

cg05785311	-0.488053508	3.136598847	-4.026330989	0.000518977	0.044954103
cg20263643	-0.485792118	1.270353349	-4.026184088	0.000519167	0.044963736
cg15894182	-0.737408433	2.918576915	-4.02611736	0.000519254	0.044963736
cg20118250	-0.676651973	1.068762309	-4.026098069	0.000519279	0.044963736
cg06149333	-0.581231493	3.274025008	-4.025977029	0.000519436	0.044971818
cg02611881	-0.507560587	0.221126936	-4.025705517	0.000519788	0.044992883
cg11945452	0.542993483	1.619600624	4.02569133	0.000519806	0.044992883
cg01245018	-0.549095701	3.568080741	-4.025530751	0.000520014	0.045003787
cg09328470	-0.408393522	3.759489937	-4.02549624	0.000520059	0.045003787
cg08999478	-0.435693917	4.108918508	-4.025400742	0.000520183	0.045004674
cg13885610	-0.549813777	4.003782115	-4.025358682	0.000520238	0.045004674
cg18070504	-0.572959282	2.867157512	-4.025341459	0.00052026	0.045004674
cg06157895	-0.377967078	0.522857789	-4.025286699	0.000520331	0.045005326
cg03151980	-0.477536061	3.661566062	-4.025222705	0.000520414	0.045007016
cg03414932	-0.455426613	0.335967562	-4.02514014	0.000520521	0.045010791
cg07892720	-0.345229183	0.535262293	-4.024905061	0.000520827	0.045026065
cg23891633	-0.747311996	2.345185793	-4.024901717	0.000520831	0.045026065
cg12578955	-0.48247203	-0.567880348	-4.024825544	0.00052093	0.045026065
cg26871185	-0.669848463	3.547057347	-4.024714318	0.000521074	0.045026065
cg25084014	-0.404633404	-0.440520838	-4.024564988	0.000521269	0.045026065
cg10463419	-0.450198369	3.637176942	-4.024546841	0.000521292	0.045026065
cg05969496	-0.550182455	3.701065876	-4.024511143	0.000521339	0.045026065
cg06043840	-0.557824515	2.105964692	-4.024497158	0.000521357	0.045026065
cg10453606	-0.483028562	-1.06110565	-4.024468124	0.000521395	0.045026065
cg02192541	-0.493642291	3.713665914	-4.024428808	0.000521446	0.045026065
cg17120273	-0.627745262	0.68839772	-4.024362139	0.000521532	0.045026065
cg13651876	-0.561219445	-0.283922853	-4.02434677	0.000521552	0.045026065
cg26888315	-0.797360787	1.959418125	-4.024337015	0.000521565	0.045026065
cg10551504	-0.586701044	3.811912151	-4.0242176	0.000521721	0.045026065
cg03740148	-0.4361868	4.165193063	-4.02420965	0.000521731	0.045026065
cg02264871	-0.575127115	4.087364808	-4.024203066	0.000521739	0.045026065
cg23728398	-0.468307064	3.661901523	-4.024173099	0.000521778	0.045026065
cg04330597	-0.485170893	0.45404573	-4.024117357	0.000521851	0.045026633
cg05730301	0.621875959	-4.291473493	4.023998263	0.000522006	0.045026633
cg10896077	-0.430767762	-1.243555843	-4.023918356	0.00052211	0.045026633
cg07212543	-0.527109545	-0.700905794	-4.023869075	0.000522174	0.045026633
cg07850407	-0.475432557	3.835520146	-4.023860222	0.000522186	0.045026633
cg01591738	-0.734159481	2.496782232	-4.02379894	0.000522266	0.045026633
cg03135014	-0.476787581	3.697866512	-4.023762497	0.000522313	0.045026633
cg02723908	-0.690135838	1.863405964	-4.023730223	0.000522355	0.045026633
cg01051240	0.592802101	2.295529741	4.023728773	0.000522357	0.045026633
cg15540554	-0.647561093	-2.690950807	-4.023678169	0.000522423	0.045026838
cg01012540	-0.477730485	3.687152334	-4.023562794	0.000522573	0.045034319
cg16430407	-0.450159128	3.742543931	-4.023383926	0.000522807	0.04504057
cg09638873	-0.738145224	2.56539066	-4.023088592	0.000523192	0.04504057
cg03566001	-0.584659216	2.847616078	-4.023051212	0.000523241	0.04504057
cg21047884	-0.460760267	3.676998007	-4.023047593	0.000523245	0.04504057

cg01178188	-0.508470578	0.437984062	-4.023037605	0.000523258	0.04504057
cg23015614	-0.460136959	-0.421517816	-4.022999146	0.000523309	0.04504057
cg02057311	-0.366535301	3.950239125	-4.022957234	0.000523363	0.04504057
cg15798716	-0.597806496	3.635729474	-4.022942376	0.000523383	0.04504057
cg12543245	-0.551976565	2.896488276	-4.022919154	0.000523413	0.04504057
cg15528793	-0.502691112	2.413258305	-4.022913936	0.00052342	0.04504057
cg01200327	-0.440161482	3.820322383	-4.022819101	0.000523544	0.04504057
cg25255839	-0.450229859	-0.978605241	-4.022801781	0.000523566	0.04504057
cg04679284	-0.370483073	-1.19806763	-4.02274858	0.000523636	0.04504057
cg05795436	-0.795194883	3.045037076	-4.022723361	0.000523669	0.04504057
cg16716514	-0.63421553	3.827494106	-4.022716362	0.000523678	0.04504057
cg21088208	-0.398368704	0.705287446	-4.022688084	0.000523715	0.04504057
cg18653405	-0.623253197	2.453845956	-4.022553049	0.000523891	0.04504057
cg03092343	0.338846194	0.848646159	4.022545036	0.000523902	0.04504057
cg00880288	-0.563307051	3.523497466	-4.022516287	0.000523939	0.04504057
cg07312504	-0.48777646	4.368087844	-4.02249932	0.000523962	0.04504057
cg04928124	-0.496556585	-1.725038992	-4.022484291	0.000523981	0.04504057
cg27209729	-0.449997347	-1.163619352	-4.022348385	0.000524159	0.045050375
cg17617848	-0.539262784	3.127287197	-4.022269409	0.000524262	0.045050582
cg26249168	-0.628921857	1.631589691	-4.022249266	0.000524288	0.045050582
cg04015361	-0.50630888	-0.512228988	-4.022117613	0.000524461	0.045059914
cg18829969	-0.45795629	3.774510108	-4.021972897	0.00052465	0.045067661
cg11999718	-0.46352976	2.772463758	-4.021925876	0.000524712	0.045067661
cg07408183	-0.485801961	0.605780669	-4.021902843	0.000524742	0.045067661
cg09111201	-0.529734431	2.211753122	-4.021848689	0.000524813	0.045068237
cg19142141	0.45709469	2.730962767	4.021776117	0.000524908	0.045068237
cg15247809	-0.421194487	3.968996667	-4.021707519	0.000524997	0.045068237
cg16557154	-0.439593628	-0.052066002	-4.021672671	0.000525043	0.045068237
cg26517670	-0.591118193	3.105372832	-4.021647469	0.000525076	0.045068237
cg05884205	-0.380038069	-0.104006553	-4.021498743	0.000525271	0.045068237
cg06073714	-0.466839148	4.00685207	-4.021491659	0.00052528	0.045068237
cg02036387	-0.474113405	-2.191905499	-4.021380013	0.000525426	0.045068237
cg20825814	-0.59550881	2.496073304	-4.021325063	0.000525498	0.045068237
cg07458761	-0.355186617	-0.007391354	-4.021314575	0.000525512	0.045068237
cg20778952	-0.489874484	3.337020997	-4.021218613	0.000525638	0.045068237
cg14944127	-0.476538377	3.635429819	-4.021160262	0.000525715	0.045068237
cg26432088	-0.57995284	2.702951606	-4.021046515	0.000525864	0.045068237
cg07232612	-0.483642371	-1.783306352	-4.021000257	0.000525924	0.045068237
cg22358628	-0.443281647	3.922755774	-4.020957511	0.00052598	0.045068237
cg18620140	-0.399761608	3.585096054	-4.020957328	0.000525981	0.045068237
cg12588678	-0.485416833	3.546257656	-4.0209511	0.000525989	0.045068237
cg12557626	-0.597572943	4.890071917	-4.020916248	0.000526035	0.045068237
cg07211215	-0.485739119	3.780560428	-4.020842248	0.000526132	0.045068237
cg21185075	-0.787533092	1.695443185	-4.020777234	0.000526217	0.045068237
cg22998101	-0.729085493	2.379747061	-4.020734454	0.000526273	0.045068237
cg15074057	-0.402866537	3.991121547	-4.020672406	0.000526355	0.045068237
cg19511335	-0.749096209	2.274803362	-4.020654244	0.000526378	0.045068237

cg26009260	-0.58858965	2.971364021	-4.020624527	0.000526418	0.045068237
cg26915370	-0.523285996	-1.554963793	-4.020588164	0.000526465	0.045068237
cg26806179	-0.899644995	2.488696716	-4.02049986	0.000526581	0.045068237
cg17279142	-0.793345526	2.079136939	-4.020457924	0.000526636	0.045068237
cg13918223	-0.461709801	3.365494901	-4.020449297	0.000526648	0.045068237
cg07957007	-0.390933376	-0.767113383	-4.020443344	0.000526655	0.045068237
cg02675394	-0.652397019	-3.323459447	-4.020430071	0.000526673	0.045068237
cg05664352	-0.688520708	-0.212781571	-4.020393744	0.000526721	0.045068237
cg07691634	-0.669989285	3.468612566	-4.020341936	0.000526789	0.045068618
cg08440635	-0.574357154	3.291893821	-4.020239067	0.000526924	0.045074739
cg14799392	0.440726351	2.071442566	4.020066408	0.000527151	0.04508599
cg11374145	-0.828555844	1.154253045	-4.020038573	0.000527187	0.04508599
cg11559967	-0.385908189	0.601788389	-4.019993791	0.000527246	0.04508599
cg15901190	-0.413766537	3.732899321	-4.019895518	0.000527376	0.045089043
cg09605312	-0.493541932	-1.540351305	-4.019866452	0.000527414	0.045089043
cg06758255	-0.537722722	-1.428330767	-4.019820656	0.000527474	0.045089043
cg19585214	-0.530773379	3.882416949	-4.019773106	0.000527537	0.045089043
cg25145282	-0.556863203	2.59626125	-4.019718281	0.000527609	0.045089769
cg18489232	-0.739165737	1.979412758	-4.019633998	0.00052772	0.045093809
cg26737169	-0.492660395	1.639100093	-4.01955016	0.00052783	0.0450978
cg04804139	-0.532722454	0.469034902	-4.019479234	0.000527923	0.045100339
cg21206339	-0.642663428	3.010645613	-4.01929813	0.000528162	0.045106064
cg09537792	-0.448189791	0.036189391	-4.019284279	0.00052818	0.045106064
cg06326646	-0.674742633	-1.526285127	-4.019206968	0.000528282	0.045106064
cg20261167	-0.505191539	-0.43766186	-4.019189786	0.000528305	0.045106064
cg04253799	-0.671985279	0.351612395	-4.01918666	0.000528309	0.045106064
cg07856295	0.535636071	-4.020036905	4.019053187	0.000528485	0.045115278
cg06423716	-0.421706619	-0.680792922	-4.018980606	0.00052858	0.045115278
cg13458914	-0.536658067	-1.455180655	-4.018959838	0.000528608	0.045115278
cg08544021	-0.613571768	2.97655603	-4.018825842	0.000528785	0.045121457
cg14649369	-0.48321556	3.626076683	-4.018717646	0.000528927	0.045121457
cg17824222	-0.502938007	2.313225954	-4.018581487	0.000529107	0.045121457
cg07213065	-0.434037134	3.969465388	-4.018520245	0.000529188	0.045121457
cg06634586	-0.748223752	0.55788059	-4.018517021	0.000529192	0.045121457
cg17109283	-0.672384973	3.469188501	-4.018463618	0.000529262	0.045121457
cg19724701	-0.380871706	3.099466356	-4.01843328	0.000529303	0.045121457
cg24885556	-0.33563819	-0.413739918	-4.018370009	0.000529386	0.045121457
cg02079411	-0.60006702	2.504172803	-4.018338095	0.000529428	0.045121457
cg14882291	-0.475685235	3.610220806	-4.018300779	0.000529477	0.045121457
cg24015175	-0.585903632	3.953333183	-4.018281531	0.000529503	0.045121457
cg01428454	-0.489735464	3.69191968	-4.018261529	0.000529529	0.045121457
cg21278889	-0.515602197	3.610351464	-4.018163286	0.000529659	0.045121457
cg25078519	-0.453684386	0.79490891	-4.018155305	0.00052967	0.045121457
cg20940424	-0.433366519	-0.781582348	-4.018110053	0.000529729	0.045121457
cg16295514	-0.667678075	3.151277445	-4.018098123	0.000529745	0.045121457
cg03859589	-0.444462286	3.850582575	-4.018084582	0.000529763	0.045121457
cg25703051	-0.460654635	3.67647909	-4.017809725	0.000530126	0.045140384

cg16887521	-0.424680652	-0.839508612	-4.017787492	0.000530156	0.045140384
cg03582793	-0.446870317	3.834652005	-4.017712047	0.000530256	0.045140384
cg00783142	-0.402507207	3.444624746	-4.017682701	0.000530294	0.045140384
cg19556780	-0.629192668	3.628953046	-4.017599807	0.000530404	0.045140384
cg22200270	-0.716804249	2.244787151	-4.017519219	0.000530511	0.045140384
cg22277689	-0.592596638	3.22675309	-4.017512187	0.00053052	0.045140384
cg12927926	-0.521547065	3.088850815	-4.017486631	0.000530554	0.045140384
cg18956798	-0.584302095	-1.319557778	-4.01748281	0.000530559	0.045140384
cg00710972	0.386190311	0.618671358	4.017303198	0.000530797	0.045146827
cg21852792	-0.424044187	2.747979827	-4.017257901	0.000530857	0.045146827
cg04022586	0.567509217	2.56401335	4.017228286	0.000530896	0.045146827
cg09762920	-0.50634588	3.240684744	-4.017176977	0.000530964	0.045146827
cg03126058	-0.505839118	-0.704277568	-4.017158711	0.000530988	0.045146827
cg06196142	-0.529410818	2.882105441	-4.017113867	0.000531047	0.045146827
cg02101876	-0.446748494	-1.190499587	-4.017077095	0.000531096	0.045146827
cg00356764	-0.461972322	3.854375756	-4.017040535	0.000531144	0.045146827
cg07238710	-0.490745389	4.502500018	-4.016917462	0.000531307	0.045148399
cg13491045	-0.646826021	2.775795365	-4.016895882	0.000531336	0.045148399
cg09838499	-0.532768458	3.569996991	-4.016882275	0.000531354	0.045148399
cg03245423	-0.653528354	2.944996332	-4.016763627	0.000531511	0.045156199
cg16913343	-0.766959719	2.45417853	-4.016716834	0.000531573	0.045156199
cg21803847	0.449174471	1.607847532	4.016615033	0.000531708	0.04516225
cg09733719	-0.528266703	3.515612364	-4.016256909	0.000532183	0.045193138
cg05547059	-0.592800865	3.521133649	-4.016244768	0.0005322	0.045193138
cg10110884	-0.637786244	3.486691241	-4.016155053	0.000532319	0.045195889
cg11474334	-0.517061308	3.457616502	-4.016087444	0.000532408	0.045195889
cg03983122	-0.695259923	3.160084676	-4.016076198	0.000532423	0.045195889
cg02187860	-0.455190766	0.5496872	-4.016015825	0.000532504	0.045197278
cg27058077	-0.403309887	0.01261114	-4.015893496	0.000532666	0.045202056
cg11180750	-0.47175809	2.801302326	-4.015875928	0.000532689	0.045202056
cg18349154	-0.660118843	3.685653547	-4.015783721	0.000532812	0.045202056
cg06487334	-0.675406114	2.288833762	-4.015779286	0.000532818	0.045202056
cg01482969	-0.429928881	-0.958802697	-4.015729045	0.000532885	0.045202056
cg10432360	-0.459042043	-1.009816076	-4.015685302	0.000532943	0.045202056
cg01589629	-0.497679839	2.705228904	-4.015544553	0.00053313	0.045212513
cg18605642	-0.70090188	1.954911565	-4.015436668	0.000533273	0.045216897
cg11823151	-0.527339808	3.244242915	-4.015409685	0.000533309	0.045216897
cg24077400	-0.452044574	4.098646657	-4.015341862	0.000533399	0.045219134
cg00981945	-0.488373599	4.209887031	-4.015206505	0.00053358	0.045223739
cg25968937	-0.48039164	4.924271167	-4.015068787	0.000533763	0.045223739
cg04554838	-0.388833971	3.884053246	-4.015032188	0.000533812	0.045223739
cg23248168	-0.573537637	3.147575887	-4.015000538	0.000533854	0.045223739
cg23684410	-0.61564161	-1.675599562	-4.014986932	0.000533872	0.045223739
cg03583510	-0.546653078	3.65675728	-4.014981869	0.000533879	0.045223739
cg07788061	-0.339336184	-1.127808401	-4.014965279	0.000533901	0.045223739
cg16254339	0.61420134	3.719067315	4.014797024	0.000534125	0.045237311
cg03262130	-0.630175545	2.902225607	-4.014493821	0.000534529	0.04525664

cg07705604	-0.403692238	1.266544743	-4.014455308	0.00053458	0.04525664
cg13515351	-0.51601369	3.995948043	-4.014364997	0.000534701	0.04525664
cg13419231	-0.432943644	4.045486925	-4.014364749	0.000534701	0.04525664
cg09522962	0.313357493	3.611564138	4.014340585	0.000534733	0.04525664
cg01787421	-0.660754125	2.776321462	-4.014338201	0.000534736	0.04525664
cg03128890	-0.562038239	-1.02347274	-4.014216957	0.000534898	0.045264919
cg04407215	-0.497566716	-1.865319586	-4.014167609	0.000534964	0.045265083
cg10515131	-0.518465941	-1.947751342	-4.014105663	0.000535046	0.045266525
cg08608240	-0.497449816	0.401222553	-4.014013428	0.00053517	0.045266525
cg14522536	-0.463076186	3.895978717	-4.014011172	0.000535173	0.045266525
cg11358988	0.40725606	2.135384442	4.013913606	0.000535303	0.045270679
cg09132846	-0.744750275	-1.474811164	-4.013836865	0.000535405	0.045270679
cg02969716	-0.626042164	2.474999914	-4.013830776	0.000535413	0.045270679
cg15937191	-0.525586958	2.909947926	-4.013493489	0.000535864	0.045293493
cg25393234	-0.588767686	3.186178601	-4.013486962	0.000535873	0.045293493
cg10705422	0.670029476	2.027067278	4.013412677	0.000535972	0.045293493
cg19450119	-0.528154185	-1.184787227	-4.013404958	0.000535982	0.045293493
cg11900250	-0.429660752	3.361651692	-4.013389525	0.000536003	0.045293493
cg25182919	-0.642613069	3.637558099	-4.013312709	0.000536106	0.045296768
cg21124875	-0.42605969	3.862879628	-4.013211772	0.000536241	0.045299565
cg09301222	-1.047723784	-4.06047639	-4.01306949	0.000536431	0.045299565
cg03129732	-0.591135368	3.613487523	-4.013038358	0.000536473	0.045299565
cg18894214	-0.411335984	-1.190303673	-4.01297617	0.000536556	0.045299565
cg07290249	-0.358674728	3.970928013	-4.012958015	0.00053658	0.045299565
cg09056639	-0.475524275	4.186257609	-4.012932644	0.000536614	0.045299565
cg01485362	-0.440404497	3.871947022	-4.012929487	0.000536618	0.045299565
cg26101183	-0.435058556	-1.433840934	-4.012847821	0.000536728	0.045299565
cg12883491	-0.390077596	3.61825545	-4.012840401	0.000536737	0.045299565
cg20455874	-0.446214394	3.763512818	-4.012809964	0.000536778	0.045299565
cg19543234	-0.677015586	2.882452019	-4.012723033	0.000536895	0.045300402
cg04436544	-0.429260486	3.378275326	-4.012613296	0.000537042	0.045300402
cg10511701	0.80344485	-4.956272288	4.012573615	0.000537095	0.045300402
cg24504942	-0.655778848	0.441106054	-4.01256634	0.000537104	0.045300402
cg09937950	-0.46279793	3.967397246	-4.0124929	0.000537203	0.045300402
cg14264355	-0.512770506	-0.907639626	-4.012438586	0.000537276	0.045300402
cg18922320	-0.474552492	4.011394611	-4.01238068	0.000537353	0.045300402
cg12615903	-0.423600865	-0.268102871	-4.01237399	0.000537362	0.045300402
cg08333778	-0.448747787	3.572114221	-4.012317432	0.000537438	0.045300402
cg01231009	-0.632273699	-0.075209095	-4.012292617	0.000537471	0.045300402
cg17601785	-0.751271419	-0.141612108	-4.012277432	0.000537492	0.045300402
cg09078517	-0.768450024	-3.449417888	-4.012173496	0.000537631	0.045306066
cg25379954	-0.65572945	0.187283831	-4.012131894	0.000537687	0.045306066
cg01112477	-0.671362965	1.002788477	-4.012056612	0.000537788	0.045307501
cg01694663	-0.498047471	3.372026551	-4.011989584	0.000537878	0.045307501
cg24330438	-0.646486794	3.226190843	-4.011864346	0.000538046	0.045307501
cg17242751	-0.605706762	1.751366146	-4.011798227	0.000538134	0.045307501
cg05699320	-0.444108198	-0.584481828	-4.01179303	0.000538141	0.045307501

cg01506748	-0.45259988	0.130968296	-4.011762309	0.000538182	0.045307501
cg11698445	-0.448922377	-0.174063171	-4.011682404	0.00053829	0.045307501
cg01959025	-0.447600264	3.855282587	-4.01163949	0.000538347	0.045307501
cg21414363	-0.616896216	3.252992002	-4.011574757	0.000538434	0.045307501
cg17022633	-0.751663892	3.554654555	-4.01155623	0.000538459	0.045307501
cg20172468	-0.859569064	2.586197445	-4.011380335	0.000538695	0.045307501
cg13195296	-0.738433548	1.774295799	-4.01134656	0.000538741	0.045307501
cg05478633	-0.437961369	4.218950377	-4.011303026	0.000538799	0.045307501
cg09949083	-0.479205802	3.603928995	-4.011247225	0.000538874	0.045307501
cg11944284	-0.364607194	1.302408399	-4.011230213	0.000538897	0.045307501
cg16224104	-0.478700546	3.09390818	-4.011158746	0.000538993	0.045307501
cg13285846	-0.414076857	-0.234525937	-4.011152782	0.000539001	0.045307501
cg01118295	-0.465916713	3.476782603	-4.011126957	0.000539036	0.045307501
cg04916611	-0.460519543	0.28029629	-4.011084735	0.000539093	0.045307501
cg01634142	-0.69129641	2.24544996	-4.01108123	0.000539097	0.045307501
cg20332756	-0.689942169	2.280491879	-4.011017822	0.000539183	0.045307501
cg01458041	0.464720336	2.637746388	4.010981516	0.000539231	0.045307501
cg00704422	-0.615810926	3.605909697	-4.010896621	0.000539346	0.045307501
cg14157644	-0.41474379	-0.234400136	-4.010885116	0.000539361	0.045307501
cg12230584	-0.422788279	0.161126682	-4.010707454	0.0005396	0.045307501
cg15017132	-0.414776337	3.660268545	-4.010688845	0.000539625	0.045307501
cg03247360	-0.682297993	2.951986968	-4.01067836	0.000539639	0.045307501
cg17881997	-0.818664765	0.527492819	-4.01061412	0.000539726	0.045307501
cg05800492	-0.361885776	-0.329805461	-4.010602028	0.000539742	0.045307501
cg15651465	-0.621107217	2.787810913	-4.010558678	0.0005398	0.045307501
cg07406728	-0.451550827	3.485363189	-4.010524733	0.000539846	0.045307501
cg02013638	-0.555071163	3.336586711	-4.010474814	0.000539913	0.045307501
cg18528216	-0.411375274	3.994572163	-4.010369201	0.000540055	0.045307501
cg04135106	-0.595757064	-3.52144147	-4.010366549	0.000540059	0.045307501
cg22824748	-0.490408556	3.870330501	-4.010320149	0.000540122	0.045307501
cg15168017	-0.728757249	2.747834703	-4.010314046	0.00054013	0.045307501
cg01592108	-0.6512433	2.309368639	-4.010311023	0.000540134	0.045307501
cg01142564	-0.536187424	3.726818777	-4.01031077	0.000540134	0.045307501
cg06754079	-0.599680416	2.480131726	-4.010230736	0.000540242	0.045311179
cg14472521	-0.382256213	-1.028402776	-4.010155451	0.000540343	0.045314321
cg16636550	-0.419690356	3.973547863	-4.009865125	0.000540735	0.04534177
cg15825897	-0.460817548	-0.530647282	-4.009782489	0.000540846	0.04534177
cg11488708	-0.876677205	0.101003784	-4.009691821	0.000540968	0.04534177
cg14289852	-0.577185076	-0.079139066	-4.009659478	0.000541012	0.04534177
cg04909420	-0.506953397	4.123352961	-4.009635838	0.000541044	0.04534177
cg16412831	-0.600258911	3.447057025	-4.009626475	0.000541057	0.04534177
cg13417891	0.591338949	2.834891487	4.009555886	0.000541152	0.04534177
cg11270278	-0.456699541	3.326834399	-4.009533014	0.000541183	0.04534177
cg17853872	-0.572801104	2.596363718	-4.009463608	0.000541276	0.045344255
cg10703884	-0.724532439	-3.49675218	-4.009391554	0.000541374	0.045347041
cg17034091	-0.373344082	-1.050251813	-4.00921749	0.000541609	0.045349834
cg01685636	-0.602398688	2.851641209	-4.009208985	0.00054162	0.045349834

cg27648216	-0.429216324	-0.975529556	-4.009177701	0.000541662	0.045349834
cg07649718	-0.409263902	4.148600233	-4.009177225	0.000541663	0.045349834
cg22786748	-0.642545507	-0.678897685	-4.008967801	0.000541946	0.0453638
cg24461304	-0.556616932	-2.218106999	-4.008948465	0.000541972	0.0453638
cg04612608	0.561281026	1.084812202	4.008911586	0.000542022	0.0453638
cg10084509	-0.528365977	3.188886441	-4.008858402	0.000542094	0.045364457
cg19746375	-0.561675865	0.141576795	-4.008804287	0.000542167	0.045365036
cg21229979	-0.705084083	-2.553869755	-4.008758554	0.000542229	0.045365036
cg02436602	-0.495979528	3.561537196	-4.008703641	0.000542303	0.04536589
cg13484813	-0.610987122	3.031631116	-4.008637832	0.000542392	0.045367978
cg04747054	-0.481763281	3.702954796	-4.008497274	0.000542582	0.045376685
cg15923705	-0.533798806	2.959263948	-4.008466186	0.000542624	0.045376685
cg15231374	-0.457315989	-0.141124081	-4.008330012	0.000542809	0.045386738
cg15035705	0.44620304	0.387727594	4.008175914	0.000543017	0.045398823
cg18616925	0.381042733	1.683875525	4.008089111	0.000543135	0.045403293
cg13583454	-0.732402615	2.855811679	-4.008038326	0.000543204	0.045403685
cg16029351	-0.466692899	0.210600761	-4.007964634	0.000543303	0.045406671
cg23346161	-0.577572337	0.339063767	-4.007911534	0.000543375	0.045407327
cg27589420	-0.499951387	3.734585262	-4.007801936	0.000543524	0.045414381
cg16715493	-0.70668425	0.957326615	-4.007713152	0.000543644	0.045414389
cg00978634	-0.394902569	-1.277773353	-4.007634545	0.000543751	0.045414389
cg03737475	-0.532675966	3.61538437	-4.007619312	0.000543772	0.045414389
cg01373691	-0.52252132	-2.231317951	-4.007542367	0.000543876	0.045414389
cg04512268	-0.663677289	-2.404729021	-4.007405507	0.000544061	0.045414389
cg02912828	-0.45203389	-1.906568858	-4.007382321	0.000544093	0.045414389
cg06854604	-0.469812771	3.40236606	-4.00737469	0.000544103	0.045414389
cg25709259	-0.73500715	2.475887456	-4.007270055	0.000544245	0.045414389
cg20076301	-0.585999034	1.210051288	-4.007221761	0.000544311	0.045414389
cg00492003	-0.674349029	0.836196816	-4.007194714	0.000544348	0.045414389
cg27315111	-0.515545685	2.966617062	-4.00714779	0.000544411	0.045414389
cg16968297	-0.794673053	0.053421091	-4.00698079	0.000544638	0.045414389
cg22212430	-0.509493074	3.123489146	-4.006954629	0.000544674	0.045414389
cg01727419	-0.725513549	0.030236729	-4.006919663	0.000544721	0.045414389
cg20517149	-0.401030005	4.227370266	-4.006886534	0.000544766	0.045414389
cg08742424	-0.802073493	-3.67991479	-4.006828394	0.000544845	0.045414389
cg13210534	-0.408070481	-1.824583781	-4.006819106	0.000544858	0.045414389
cg12033578	-0.530659744	2.427316378	-4.006804523	0.000544877	0.045414389
cg02524864	-0.755267297	0.924544981	-4.006797646	0.000544887	0.045414389
cg07171886	-0.408570378	0.278021996	-4.006711161	0.000545004	0.045414389
cg05448979	-0.665816713	1.373442489	-4.006707013	0.00054501	0.045414389
cg16886756	-0.484415603	2.975252642	-4.006690463	0.000545032	0.045414389
cg27315303	-0.549050878	4.141563773	-4.006689486	0.000545034	0.045414389
cg02093811	0.364414044	1.561751876	4.006662907	0.00054507	0.045414389
cg11370966	-0.673281319	3.190572793	-4.006451977	0.000545357	0.045414389
cg02251376	-0.452295597	3.321532382	-4.006410126	0.000545414	0.045414389
cg03992638	-0.419036254	-1.605808551	-4.006400079	0.000545427	0.045414389
cg07304175	-0.464537622	-1.914538019	-4.006313649	0.000545545	0.045414389

cg25027882	-0.489159449	3.324677969	-4.006299009	0.000545565	0.045414389
cg13802419	-0.546967618	3.523809578	-4.006278775	0.000545592	0.045414389
cg22303442	-0.563978269	3.219454205	-4.00619855	0.000545701	0.045414389
cg23034245	-0.608614995	3.167476319	-4.006132738	0.000545791	0.045414389
cg02367389	-0.538866514	3.131265094	-4.00610916	0.000545823	0.045414389
cg17181214	0.532106114	1.953802707	4.006069697	0.000545877	0.045414389
cg06270423	-0.584646751	3.778807913	-4.006007107	0.000545962	0.045414389
cg16474890	-0.554784045	2.978294528	-4.005999278	0.000545973	0.045414389
cg03340303	-0.463754438	0.350864678	-4.00591643	0.000546085	0.045414389
cg12280343	-0.555887738	3.650912741	-4.005825328	0.000546209	0.045414389
cg14501977	-0.546515767	0.065738626	-4.005810923	0.000546229	0.045414389
cg22215512	-0.717979343	2.087513141	-4.005767475	0.000546288	0.045414389
cg08805081	-0.634421955	3.621893327	-4.005693104	0.00054639	0.045414389
cg16882323	-0.773022722	2.151909036	-4.005688609	0.000546396	0.045414389
cg24846030	-0.623113499	1.612688163	-4.005684458	0.000546401	0.045414389
cg04443978	-0.637207071	1.677348516	-4.005618502	0.000546491	0.045414389
cg20889490	-0.490916527	3.261735126	-4.005526159	0.000546617	0.045414389
cg14466345	-0.483909645	3.080143644	-4.005516691	0.00054663	0.045414389
cg08814714	-0.37338662	3.934743139	-4.005457914	0.00054671	0.045414389
cg08580087	-0.409351107	3.373640011	-4.005400245	0.000546789	0.045414389
cg01891969	-0.490620179	0.035435761	-4.005391293	0.000546801	0.045414389
cg14634210	0.535223897	-4.035375904	4.005328999	0.000546886	0.045414389
cg23050404	-0.557036836	2.68951438	-4.005322372	0.000546895	0.045414389
cg01247671	-0.460109031	3.244815607	-4.005321865	0.000546896	0.045414389
cg02194900	-0.610048606	3.044731117	-4.005301967	0.000546923	0.045414389
cg08244436	-0.429046193	-0.91127906	-4.005255595	0.000546986	0.045414389
cg03718164	-0.515926789	3.69506616	-4.005003436	0.00054733	0.045425103
cg21944221	-0.413630678	4.158184555	-4.004989681	0.000547349	0.045425103
cg02160482	-0.446770496	3.531685269	-4.004955956	0.000547395	0.045425103
cg06073220	-0.464099072	-0.665292841	-4.004919931	0.000547444	0.045425103
cg11921585	-0.531280349	3.69582271	-4.004900995	0.00054747	0.045425103
cg07388950	0.496992908	0.785286995	4.004836581	0.000547558	0.045425103
cg17918190	-0.783319222	2.491557102	-4.004832086	0.000547564	0.045425103
cg18224734	-0.688365868	2.803024304	-4.004755948	0.000547668	0.045427224
cg12833861	-0.527446695	3.279879622	-4.004719433	0.000547718	0.045427224
cg14364815	-0.52996659	3.033168522	-4.004604342	0.000547875	0.045434943
cg22518770	-0.510286605	3.369699985	-4.004509677	0.000548004	0.045440349
cg27101899	-0.470740012	-1.200203588	-4.004337706	0.000548239	0.045451487
cg10122855	-0.831453154	1.873176415	-4.00431751	0.000548267	0.045451487
cg03877350	0.655837777	2.184221535	4.004233424	0.000548382	0.045455698
cg09714135	0.65950964	2.901185231	4.004144203	0.000548504	0.045460491
cg10072269	-0.434078611	-0.176048722	-4.004091943	0.000548575	0.045461097
cg01125244	-0.432227974	2.304497514	-4.004009098	0.000548689	0.045465169
cg22400303	-0.5005214	3.499202228	-4.003930635	0.000548796	0.045468747
cg15204882	0.415585926	1.498072507	4.003863115	0.000548888	0.045471084
cg12727718	0.709478217	2.689210843	4.003764989	0.000549023	0.045475599
cg13282237	-0.414325819	-0.800219097	-4.003729501	0.000549071	0.045475599

cg25893964	-0.42287753	3.306154926	-4.003491067	0.000549398	0.045497323
cg00676304	-0.485150217	0.01066411	-4.003333528	0.000549614	0.045506142
cg06679851	0.702304297	2.337567415	4.003319569	0.000549633	0.045506142
cg01966553	-0.473859581	3.987106775	-4.00326269	0.000549711	0.045507278
cg09122838	-0.470215858	1.021136519	-4.003189365	0.000549811	0.04551028
cg25574421	-0.581982681	1.690160339	-4.003062829	0.000549985	0.045511131
cg07353255	-0.477905587	4.068053545	-4.003054531	0.000549996	0.045511131
cg19804403	-1.060630207	1.881963369	-4.003026823	0.000550034	0.045511131
cg10935283	-0.465661489	3.435474046	-4.002994444	0.000550079	0.045511131
cg15401505	-0.542273375	3.483307445	-4.002857001	0.000550267	0.045512507
cg12591881	-0.657379487	2.644840886	-4.002852868	0.000550273	0.045512507
cg13846359	-0.75996479	2.071428528	-4.002841821	0.000550288	0.045512507
cg07066859	-0.487443228	3.534096301	-4.002667348	0.000550527	0.04552155
cg17514485	-0.479833461	-1.015991863	-4.002660701	0.000550536	0.04552155
cg23887149	-0.435065448	-0.768842127	-4.002611402	0.000550604	0.04552155
cg07391756	0.754651551	-4.311617324	4.002574872	0.000550654	0.04552155
cg21147683	-0.549886593	3.279783287	-4.002433219	0.000550849	0.045532317
cg05114959	-0.379931036	-0.891704732	-4.002371801	0.000550933	0.045533977
cg23109891	-0.511322196	-1.047263241	-4.002274338	0.000551067	0.045537797
cg12397924	-0.520710595	-0.971609013	-4.002244573	0.000551108	0.045537797
cg16509830	-0.516593559	-2.32276034	-4.002138824	0.000551253	0.045544493
cg09449346	0.457412573	3.511467473	4.002004726	0.000551438	0.045554365
cg03260824	-0.537746425	4.484013325	-4.001958355	0.000551501	0.045554365
cg21308839	-0.482525452	1.789434827	-4.001896831	0.000551586	0.045556041
cg09645993	-0.555300155	-0.724543007	-4.001752515	0.000551785	0.045557717
cg09402329	-0.588128786	3.448794491	-4.001717984	0.000551832	0.045557717
cg16862361	-0.357223645	3.711602285	-4.001692157	0.000551868	0.045557717
cg08612059	-0.545199381	3.460937811	-4.001631117	0.000551952	0.045557717
cg07364026	-0.451540468	3.769653373	-4.001617143	0.000551971	0.045557717
cg14310109	-0.629924193	-0.539215464	-4.001567058	0.00055204	0.045557717
cg16012953	-0.420525828	3.919708616	-4.001518355	0.000552107	0.045557717
cg14360799	-0.432308038	-1.23925976	-4.001508155	0.000552121	0.045557717
cg17496142	-0.537805905	3.123878042	-4.001387033	0.000552288	0.045563921
cg23540272	-0.762121007	2.556935389	-4.0013363	0.000552357	0.045563921
cg16431067	-0.641200517	1.630636017	-4.001313402	0.000552389	0.045563921
cg21932368	-0.543224661	-2.65188168	-4.001211962	0.000552529	0.045567242
cg07803864	-0.459055222	3.919099257	-4.001190778	0.000552558	0.045567242
cg20198095	-0.605072701	3.261118436	-4.001110432	0.000552669	0.045570902
cg04148941	-0.45025078	-0.142905984	-4.001040179	0.000552765	0.045570902
cg02213001	-0.465787899	4.053328598	-4.000950847	0.000552889	0.045570902
cg10784298	-0.307677331	-0.068659746	-4.00093574	0.000552909	0.045570902
cg04670031	-0.538557633	2.908064255	-4.000925166	0.000552924	0.045570902
cg09617135	-0.593511515	-2.264756628	-4.000731089	0.000553192	0.045587654
cg01814991	-0.546201367	3.276965216	-4.000566027	0.000553419	0.045597507
cg04337725	-0.594264558	3.364689834	-4.000551108	0.00055344	0.045597507
cg03143333	-0.441170497	3.522221808	-4.000322061	0.000553756	0.045609043
cg20564679	-0.695337258	2.512199392	-4.000299026	0.000553788	0.045609043

cg10581365	-0.522087576	3.348827993	-4.000268836	0.000553829	0.045609043
cg09673047	-0.473508505	3.475782297	-4.000240871	0.000553868	0.045609043
cg08388039	-0.336770823	3.460703142	-4.000216465	0.000553902	0.045609043
cg21498161	-0.549448224	3.570225116	-4.000110704	0.000554048	0.045610056
cg10181698	-0.739421272	1.515969459	-4.000096483	0.000554067	0.045610056
cg18251108	-0.598830472	-1.971475171	-4.00004122	0.000554144	0.045610056
cg23466241	-0.546448022	3.674185512	-4.000021103	0.000554172	0.045610056
cg18302315	-0.787393583	2.169619683	-3.999953337	0.000554265	0.045612463
cg01975338	-0.56780663	3.271980061	-3.999852321	0.000554405	0.045618653
cg15443628	-0.387363798	-1.204297913	-3.999702326	0.000554612	0.045630415
cg23947043	-0.773253966	0.932318679	-3.999622944	0.000554722	0.045634147
cg06179698	-0.441600622	3.774897891	-3.999407628	0.00055502	0.04564937
cg25185966	-0.684446599	1.790936889	-3.999357561	0.000555089	0.04564937
cg14173642	-0.505220388	3.317852459	-3.999349444	0.0005551	0.04564937
cg06514313	-0.398334874	3.721616267	-3.999287254	0.000555186	0.045651149
cg15612257	-0.327717681	-0.161769309	-3.999198317	0.00055531	0.045655973
cg18113467	-0.466184852	3.289063518	-3.999076826	0.000555478	0.045663131
cg21940302	-0.698099294	2.265493518	-3.999042337	0.000555526	0.045663131
cg26051856	-0.481102595	4.149140113	-3.998967722	0.000555629	0.045666328
cg15678685	-0.92897423	1.245145925	-3.998900357	0.000555722	0.045668699
cg18605135	-0.419877064	3.639621529	-3.998807595	0.000555851	0.045673963
cg24796998	0.377187817	1.316422363	3.998754734	0.000555924	0.045674685
cg03012310	-0.415214334	-1.401550884	-3.998626478	0.000556102	0.045680526
cg07221298	-0.604470212	0.412107078	-3.998540955	0.00055622	0.045680526
cg26166935	-0.439734513	0.605773196	-3.998529967	0.000556236	0.045680526
cg17063127	-0.545078485	-0.433783211	-3.99844295	0.000556356	0.045680526
cg04416045	-0.563263113	-0.250295736	-3.998229387	0.000556653	0.045680526
cg11173332	-0.604845573	3.140067897	-3.998212893	0.000556676	0.045680526
cg14537183	-0.469880071	0.810071915	-3.998182928	0.000556717	0.045680526
cg18452347	-0.373414009	3.826463662	-3.998089606	0.000556847	0.045680526
cg04930925	-0.378145812	-0.960895157	-3.99806579	0.00055688	0.045680526
cg04853218	0.376921604	1.004990394	3.998047611	0.000556905	0.045680526
cg00165429	-0.912477503	1.106829474	-3.99804372	0.00055691	0.045680526
cg04245131	-0.530278219	-2.999901745	-3.998040273	0.000556915	0.045680526
cg16436933	-0.448124728	-0.108310821	-3.997992612	0.000556981	0.045680526
cg14769556	-0.480718543	3.252598532	-3.997990279	0.000556985	0.045680526
cg18324201	-0.657038008	1.970609537	-3.997950267	0.00055704	0.045680526
cg07391920	-0.377922795	3.592623323	-3.997939177	0.000557056	0.045680526
cg08447116	-0.513242051	3.399354458	-3.997879212	0.000557139	0.045680526
cg07063883	0.613732584	-3.972567437	3.997851072	0.000557178	0.045680526
cg22089922	-0.451864638	-0.894810396	-3.997820501	0.00055722	0.045680526
cg06769994	-0.861186181	0.044625649	-3.997598624	0.000557529	0.0456906
cg21051907	-0.550389531	3.832951608	-3.99758654	0.000557545	0.0456906
cg24915620	-0.56586467	3.569573391	-3.997518566	0.00055764	0.0456906
cg00151915	-0.582258617	3.52754491	-3.997466341	0.000557713	0.0456906
cg00251121	-0.457005625	3.645294411	-3.997464869	0.000557715	0.0456906
cg12660963	-0.490807633	-0.920493477	-3.997453625	0.00055773	0.0456906

cg07574216	-0.747365537	1.444018666	-3.997249516	0.000558014	0.045698456
cg24815728	-0.4221004	0.088787425	-3.997239727	0.000558028	0.045698456
cg05133706	-0.452480433	0.013698718	-3.997205431	0.000558075	0.045698456
cg13809026	-0.476903462	3.822177631	-3.997199156	0.000558084	0.045698456
cg13029400	-0.726443435	-1.96560508	-3.997082939	0.000558246	0.045706414
cg19505398	-0.422487397	4.04894946	-3.997003769	0.000558356	0.045710153
cg07446430	-0.449308195	3.457532331	-3.996804443	0.000558634	0.045727588
cg25141441	-0.740519889	2.742676408	-3.996724762	0.000558745	0.045731388
cg22761692	-0.483247193	0.934157778	-3.996652756	0.000558845	0.045734314
cg22695364	-0.645148601	2.134735927	-3.996573607	0.000558955	0.045738055
cg16164062	-0.553014269	3.604766801	-3.996482461	0.000559082	0.045739065
cg24210821	-0.450432882	3.21417114	-3.996468496	0.000559102	0.045739065
cg06959251	-0.447740364	-0.184641813	-3.996390768	0.00055921	0.045739065
cg19863563	-0.454569015	3.080919773	-3.996379452	0.000559226	0.045739065
cg08238317	-0.564115967	3.045975393	-3.996311455	0.000559321	0.045741538
cg18112451	-0.660240278	2.443350643	-3.995917281	0.00055987	0.045769226
cg27474786	-0.580814363	3.400400604	-3.995763411	0.000560085	0.045769226
cg05086580	-0.569073622	2.488190991	-3.995757883	0.000560093	0.045769226
cg15187175	-0.427679738	3.861626661	-3.995709257	0.000560161	0.045769226
cg02485629	-0.439268399	-0.945263123	-3.995707488	0.000560163	0.045769226
cg13910614	-0.474019038	0.536831639	-3.99568848	0.00056019	0.045769226
cg10502602	-0.53884565	3.644722773	-3.995686742	0.000560192	0.045769226
cg08042092	-0.428196881	-2.771675359	-3.995661877	0.000560227	0.045769226
cg06062423	-0.55613371	3.445043714	-3.995606252	0.000560305	0.045769226
cg20235573	-0.530575803	3.284998956	-3.995590008	0.000560327	0.045769226
cg03306142	-0.580078244	0.356509709	-3.995559633	0.00056037	0.045769226
cg03139336	-0.633656825	2.812626205	-3.995265074	0.000560782	0.045795698
cg16842280	-0.567159451	2.333310535	-3.995235194	0.000560823	0.045795698
cg13911576	-0.568262034	2.98830325	-3.995160431	0.000560928	0.045796878
cg06398524	-0.381728188	3.490532403	-3.994998894	0.000561154	0.045796878
cg15068527	-0.442002482	3.456703243	-3.99492187	0.000561262	0.045796878
cg22352214	-0.418525454	3.48720445	-3.994895491	0.000561298	0.045796878
cg18962998	-0.628614072	3.009232098	-3.994869527	0.000561335	0.045796878
cg02168436	-0.393294132	3.179832614	-3.994748001	0.000561505	0.045796878
cg11205298	-0.641627969	2.450777521	-3.994738667	0.000561518	0.045796878
cg24335282	-0.398564669	3.55721375	-3.994726786	0.000561535	0.045796878
cg14699723	-0.606798518	2.67288506	-3.994623326	0.000561679	0.045796878
cg10721491	-0.399861293	4.110747674	-3.994526318	0.000561815	0.045796878
cg21056788	-0.5505043	-0.121746071	-3.994517881	0.000561827	0.045796878
cg01627172	-0.690598889	2.348878391	-3.994320643	0.000562103	0.045796878
cg24866203	-0.431252436	3.61866834	-3.994282957	0.000562156	0.045796878
cg02694395	-0.525344702	0.815573245	-3.994246124	0.000562208	0.045796878
cg22405154	-0.615970428	-0.480510386	-3.994237314	0.00056222	0.045796878
cg07533740	0.729605015	-3.542006875	3.994232057	0.000562227	0.045796878
cg05087600	-0.448823638	3.309552301	-3.994110771	0.000562398	0.045796878
cg15622564	-0.465001587	3.762638797	-3.994105699	0.000562405	0.045796878
cg26544155	-0.429222801	3.310078691	-3.99406873	0.000562456	0.045796878

cg01697003	-0.594075488	2.944837511	-3.994068626	0.000562457	0.045796878
cg26774602	-0.589056474	2.631005586	-3.994063597	0.000562464	0.045796878
cg11929948	-0.450521189	3.527822723	-3.99400805	0.000562542	0.045796878
cg08680528	-0.463171118	3.936364761	-3.993921908	0.000562662	0.045796878
cg20893892	-0.580009433	1.911214132	-3.993909125	0.00056268	0.045796878
cg11660650	-0.465147876	3.717988007	-3.993846522	0.000562768	0.045796878
cg26666286	-0.444844624	3.24589872	-3.993831212	0.00056279	0.045796878
cg26108824	-0.596400599	2.193748406	-3.993751127	0.000562902	0.045796878
cg22673269	-0.407229993	3.684281459	-3.993741145	0.000562916	0.045796878
cg26462420	-0.450742299	2.875757363	-3.993709605	0.00056296	0.045796878
cg02227480	-0.443061298	2.769436008	-3.993702234	0.000562971	0.045796878
cg22801980	-0.749774169	1.423143069	-3.993701809	0.000562971	0.045796878
cg26099899	-0.448139584	2.982628112	-3.99369502	0.000562981	0.045796878
cg09027601	-0.429958515	-0.860488136	-3.993575919	0.000563148	0.045796878
cg23619461	-0.562237644	3.066554818	-3.993486249	0.000563274	0.045796878
cg07242336	-0.460209553	4.138522805	-3.99344538	0.000563331	0.045796878
cg08486595	-0.485550678	3.312574857	-3.993370167	0.000563437	0.045796878
cg04786631	-0.543436784	3.469623358	-3.993324646	0.000563501	0.045796878
cg18484079	-0.414331357	4.277381066	-3.993318195	0.00056351	0.045796878
cg25195897	-0.486024221	3.859091769	-3.993214554	0.000563655	0.045796878
cg26182317	-0.608639433	3.27889617	-3.993148685	0.000563748	0.045796878
cg06975633	-0.39279708	-0.682058225	-3.993091134	0.000563829	0.045796878
cg02814641	-0.633187512	3.469041181	-3.993079425	0.000563845	0.045796878
cg05394000	-0.690525942	1.600232439	-3.993061254	0.000563871	0.045796878
cg02567839	-0.603481584	-1.722726829	-3.993007278	0.000563947	0.045796878
cg19817540	-0.655365086	2.7685607	-3.992989667	0.000563971	0.045796878
cg22974301	-0.575460874	2.893731979	-3.992935383	0.000564048	0.045796878
cg13522684	-0.394559044	4.102472183	-3.992923003	0.000564065	0.045796878
cg13409737	-0.477700427	-0.054770883	-3.992919881	0.00056407	0.045796878
cg13145583	-0.550311707	-0.718903899	-3.992894749	0.000564105	0.045796878
cg10565356	-0.571346248	2.071126994	-3.992800224	0.000564238	0.045796878
cg03528037	0.502649925	2.925509936	3.99270999	0.000564365	0.045796878
cg01547910	-0.657367401	3.440469648	-3.992664944	0.000564428	0.045796878
cg22488426	-0.578918791	0.049403955	-3.992658207	0.000564438	0.045796878
cg04499272	-0.582038735	0.41642532	-3.99265815	0.000564438	0.045796878
cg08583234	-0.523197723	2.839793854	-3.992653746	0.000564444	0.045796878
cg13948824	-0.493324272	0.262383798	-3.992612921	0.000564501	0.045796878
cg21402062	-0.668240184	2.333219099	-3.992597672	0.000564523	0.045796878
cg15353890	-0.5777659	1.945784604	-3.992488946	0.000564676	0.045799576
cg06957256	-0.560620945	3.499348283	-3.992482166	0.000564685	0.045799576
cg14679333	-0.635646545	2.69402741	-3.992426854	0.000564763	0.045800647
cg05812685	-0.546028986	2.756536214	-3.992250806	0.000565011	0.045811992
cg13212982	-0.672175038	2.084393265	-3.992195154	0.000565089	0.045811992
cg11631342	-0.421246704	0.45049106	-3.992161116	0.000565137	0.045811992
cg17932109	0.520155102	1.574367794	3.992109319	0.00056521	0.045811992
cg01028364	1.06372401	2.330394758	3.99201862	0.000565338	0.045811992
cg23537560	-0.438847619	3.620771181	-3.992003847	0.000565359	0.045811992

cg04125003	0.535371883	4.02344897	3.991980271	0.000565392	0.045811992
cg04839706	-0.475098245	-1.902543796	-3.991960255	0.00056542	0.045811992
cg25841008	-0.416344057	0.162969088	-3.99188473	0.000565527	0.045814556
cg27307648	-0.601416133	3.314227403	-3.991846039	0.000565581	0.045814556
cg27276917	-0.521606142	4.339054284	-3.99178635	0.000565666	0.045816134
cg19020398	-0.686123846	-0.369356183	-3.991684256	0.00056581	0.045819941
cg11609914	-0.516747425	3.299151213	-3.991661295	0.000565842	0.045819941
cg21628936	-0.588221535	3.423075275	-3.991586709	0.000565947	0.045823222
cg01013225	-0.616298093	3.357488336	-3.991538974	0.000566015	0.045823437
cg14051567	-0.405604062	0.376552304	-3.991352487	0.000566278	0.045825538
cg00381033	-0.501927206	3.004427847	-3.991345279	0.000566288	0.045825538
cg12150182	-0.643753716	-1.111301637	-3.991342816	0.000566291	0.045825538
cg24648156	-0.563033797	2.438140182	-3.991308982	0.000566339	0.045825538
cg18857889	-0.356002733	-0.95610411	-3.991291409	0.000566364	0.045825538
cg00700389	0.581840938	3.894712622	3.991227225	0.000566455	0.045827635
cg02883332	-0.655715672	2.660921401	-3.991081314	0.000566661	0.045839071
cg10236264	-0.474117758	3.711192409	-3.990985679	0.000566796	0.045843487
cg05731801	0.406473924	1.098495197	3.990951054	0.000566845	0.045843487
cg23395213	-0.539370262	3.85394795	-3.990711108	0.000567184	0.045865678
cg25472458	-0.561026828	3.679911667	-3.990639436	0.000567285	0.045868071
cg27573017	-0.404868732	-0.490124231	-3.990598594	0.000567343	0.045868071
cg08783639	0.688682852	2.327200491	3.990486969	0.000567501	0.045873042
cg01317818	-0.588408909	3.183393766	-3.990438535	0.000567569	0.045873042
cg04605625	-0.681671192	2.115396429	-3.990369596	0.000567667	0.045873042
cg04705669	-0.521264366	-0.177706525	-3.99033788	0.000567712	0.045873042
cg12227863	-0.570104924	2.593524475	-3.990326245	0.000567728	0.045873042
cg06745783	0.440550403	2.515273059	3.99018916	0.000567922	0.04587369
cg22272369	-0.782301235	1.666522101	-3.990185276	0.000567928	0.04587369
cg10134889	-0.603743332	3.08987368	-3.990183323	0.00056793	0.04587369
cg20377881	-0.655427739	0.5568956	-3.990066024	0.000568096	0.04587998
cg19482605	-0.350166252	4.432482673	-3.989999751	0.00056819	0.04587998
cg21602846	-0.501663259	3.031543502	-3.989923078	0.000568299	0.04587998
cg06448442	-0.550358003	4.148366018	-3.98986771	0.000568377	0.04587998
cg27293439	-0.909978611	0.315771158	-3.989849961	0.000568403	0.04587998
cg02062018	-0.528912968	-0.735482121	-3.989790508	0.000568487	0.04587998
cg27536962	-0.476295992	3.598448408	-3.989779269	0.000568503	0.04587998
cg21833419	-0.478943153	3.079795401	-3.989754446	0.000568538	0.04587998
cg02309725	-0.628023294	3.483014899	-3.989673389	0.000568653	0.04587998
cg27543308	-0.566018685	3.636539219	-3.989671121	0.000568656	0.04587998
ch.2.4478726F	-0.487613864	-1.56827649	-3.989531523	0.000568854	0.04589072
cg16946290	-0.578425243	-0.333349418	-3.989379134	0.00056907	0.04589315
cg23880631	0.702216072	-4.523556684	3.989364794	0.00056909	0.04589315
cg04891802	-0.400112034	0.027177942	-3.989359635	0.000569098	0.04589315
cg05448059	-0.545052949	4.13312539	-3.989327568	0.000569143	0.04589315
cg05859039	0.805973587	-2.721808592	3.989151189	0.000569393	0.045904662
cg03447603	-0.676497783	3.08750597	-3.989110375	0.000569451	0.045904662
cg22044207	-0.535405528	2.453229605	-3.989066182	0.000569514	0.045904662

cg22456178	-0.557728107	-1.637047937	-3.989044306	0.000569545	0.045904662
cg26571535	-0.540456096	3.437180134	-3.988979768	0.000569637	0.045906824
cg01386978	-0.742677089	4.500362429	-3.988903501	0.000569745	0.045908167
cg09140833	-0.640589237	3.321176267	-3.988876754	0.000569783	0.045908167
cg01182236	-0.514243296	2.904105501	-3.988768638	0.000569937	0.045915318
cg12912394	-0.478740922	3.486415757	-3.988627115	0.000570138	0.045921801
cg16030275	-0.461473382	4.243977675	-3.988620753	0.000570147	0.045921801
cg12827830	-0.47581067	3.7156186	-3.988480651	0.000570346	0.045925651
cg24288715	-0.552888588	0.627075604	-3.988451935	0.000570387	0.045925651
cg04232209	-0.489088517	3.466921597	-3.988441927	0.000570401	0.045925651
cg17407324	-0.445498257	3.443401711	-3.988404686	0.000570454	0.045925651
cg25325655	-0.562377331	3.728421109	-3.988286184	0.000570622	0.045930387
cg21319916	-0.600240814	0.936297341	-3.988147687	0.000570819	0.045930387
cg16529686	-0.484645874	3.133460383	-3.988110313	0.000570873	0.045930387
cg17576891	-0.573580491	3.610931282	-3.988052404	0.000570955	0.045930387
cg03557445	-0.626019715	-0.924419347	-3.987969077	0.000571074	0.045930387
cg08028739	-0.496870134	4.028255861	-3.987953333	0.000571096	0.045930387
cg16688681	-0.42507597	3.887309071	-3.987911343	0.000571156	0.045930387
cg12470014	-0.382356271	-0.109358365	-3.98786388	0.000571223	0.045930387
cg06037759	-0.63159949	1.961117609	-3.987823414	0.000571281	0.045930387
cg10518811	-0.541999988	3.571780132	-3.987810202	0.0005713	0.045930387
cg01336497	-0.725666718	1.599761865	-3.987771363	0.000571355	0.045930387
cg10912290	-0.438018639	0.484180191	-3.987746124	0.000571391	0.045930387
cg25424279	-0.396263145	-0.74759059	-3.987686964	0.000571475	0.045930387
cg17332071	-0.569909273	3.530708234	-3.987686812	0.000571475	0.045930387
cg05347405	-0.672085829	2.397620431	-3.987666905	0.000571504	0.045930387
cg01070948	-0.40535202	-1.000054712	-3.987634426	0.00057155	0.045930387
cg22313543	-0.420863936	-0.145581087	-3.987580829	0.000571626	0.045931313
cg01111006	-0.682920869	0.004695269	-3.987472566	0.000571781	0.045933226
cg06572642	-0.570775095	3.861193715	-3.987372845	0.000571923	0.045933226
cg11928173	-0.665980137	4.054292657	-3.987367635	0.00057193	0.045933226
cg16434510	-0.823977573	1.485519	-3.987328555	0.000571986	0.045933226
cg27641106	-0.533385406	4.065150455	-3.98726945	0.00057207	0.045933226
cg14208149	-0.573573523	0.239889151	-3.987232443	0.000572123	0.045933226
cg16081992	-0.52020808	2.939852758	-3.987192216	0.00057218	0.045933226
cg08039343	-0.524736555	3.924275966	-3.987138203	0.000572257	0.045933226
cg17326634	-0.385122609	-0.015999928	-3.987033044	0.000572407	0.045933226
cg00755950	-0.491521651	-2.486673546	-3.98700602	0.000572446	0.045933226
cg06671642	-0.444081194	-2.042962702	-3.986951871	0.000572523	0.045933226
cg26355377	-0.596441735	3.234688236	-3.986892018	0.000572609	0.045933226
cg24182798	-0.3782468	-1.239625761	-3.986879077	0.000572627	0.045933226
cg16228077	-0.652924379	-0.199808328	-3.986840762	0.000572682	0.045933226
cg26835826	-0.511836285	-0.143798033	-3.986802092	0.000572737	0.045933226
cg14178821	-0.605076066	3.23078206	-3.986635461	0.000572975	0.045933226
cg00471617	-0.746427919	2.294927425	-3.986604797	0.000573019	0.045933226
cg25529477	-0.395331	-1.226936051	-3.986597649	0.000573029	0.045933226
cg02984631	-0.633997789	3.113351677	-3.986587098	0.000573044	0.045933226

cg20610798	-0.790635233	2.100081331	-3.98658002	0.000573054	0.045933226
cg23729777	-0.461113231	-1.156987304	-3.986567931	0.000573071	0.045933226
cg21383282	-0.495401535	3.435601286	-3.986493899	0.000573177	0.045933226
cg23713090	-0.485292067	3.670416527	-3.986425297	0.000573275	0.045933226
cg25567495	-0.591346752	0.868296815	-3.986422769	0.000573279	0.045933226
cg18081979	-0.425546315	-0.331728593	-3.986412904	0.000573293	0.045933226
cg08805995	0.430650471	1.459797622	3.986382596	0.000573336	0.045933226
cg16397813	-0.471245541	4.096087934	-3.986100075	0.00057374	0.045947321
cg00135874	0.444564213	2.267423312	3.986068877	0.000573785	0.045947321
cg10972392	-0.749207776	1.979407648	-3.986067643	0.000573786	0.045947321
cg19962304	-0.548000017	3.202165919	-3.986050021	0.000573812	0.045947321
cg13728813	-0.526259445	3.319917622	-3.986032671	0.000573836	0.045947321
cg17241937	-0.457981617	3.335717265	-3.985793691	0.000574178	0.045964552
cg22557090	-0.627587594	3.368405491	-3.985766589	0.000574217	0.045964552
cg08717423	-0.638134311	2.936355458	-3.985737744	0.000574258	0.045964552
cg17711252	-0.530140921	2.709778307	-3.985700867	0.000574311	0.045964552
cg15344339	-0.646124968	2.34689954	-3.985641117	0.000574397	0.045966204
cg11419747	0.62405413	3.56947587	3.985501828	0.000574596	0.045976971
cg20711163	0.520738176	3.411700848	3.985294728	0.000574893	0.045994098
cg00806929	-0.391291716	3.809940549	-3.985237623	0.000574975	0.045994098
cg06761094	-0.373062785	3.638337648	-3.985216449	0.000575005	0.045994098
cg02944021	-0.647270112	2.960245824	-3.98516709	0.000575076	0.045994563
cg03932595	0.48974029	2.552644611	3.985038406	0.00057526	0.046004122
cg16237600	-0.414817261	3.533160963	-3.984992437	0.000575326	0.0460042
cg07760898	-0.451866707	3.916097599	-3.984936512	0.000575406	0.046005419
cg02691381	-0.457328566	3.341954083	-3.984820104	0.000575573	0.04600629
cg12330854	-0.448262519	4.124220494	-3.984800058	0.000575602	0.04600629
cg14942024	-0.543566927	3.139127921	-3.984732911	0.000575698	0.04600629
cg04988180	-0.733839846	3.064754587	-3.98466731	0.000575792	0.04600629
cg22324297	-0.422004816	2.646651164	-3.98463917	0.000575833	0.04600629
cg05804899	-0.530083214	1.718360369	-3.984631821	0.000575843	0.04600629
cg11026385	-0.53311985	-0.491384063	-3.984568691	0.000575934	0.04600629
cg11820824	-0.539686162	-0.577304634	-3.984566784	0.000575937	0.04600629
cg17871838	-0.529317163	2.945595468	-3.984408347	0.000576164	0.046014535
cg25716314	-0.522121878	3.107754546	-3.984404408	0.00057617	0.046014535
cg22099696	-0.597514079	0.998560387	-3.98432935	0.000576278	0.04601652
ch.13.58694637R	-0.803146357	-2.799872958	-3.984296638	0.000576325	0.04601652
cg06137032	-0.397513613	1.102037054	-3.984176726	0.000576497	0.046025086
cg11793126	-0.626791136	2.956374805	-3.983864255	0.000576946	0.046038099
cg11021000	-0.974515533	2.926780708	-3.983770255	0.000577081	0.046038099
cg00430036	-0.463319245	-1.695939864	-3.983752027	0.000577107	0.046038099
cg12213818	-0.628611195	3.230914664	-3.983724168	0.000577147	0.046038099
cg13498479	-0.488343229	3.945301044	-3.983661615	0.000577237	0.046038099
cg15148028	-0.533676788	3.942080372	-3.983586931	0.000577345	0.046038099
cg24705158	-0.405065619	0.267773506	-3.983575696	0.000577361	0.046038099
cg09750940	-0.57690816	3.111870886	-3.983564561	0.000577377	0.046038099
cg04421286	-0.451769936	4.025991889	-3.983553811	0.000577393	0.046038099

cg11485456	-0.443806376	-0.245037604	-3.983362486	0.000577668	0.046038099
cg14871333	-0.48006661	-1.102697038	-3.983354378	0.00057768	0.046038099
cg19153028	-0.753356451	2.537084489	-3.983324813	0.000577722	0.046038099
cg05963712	-0.904285133	-4.172358781	-3.983261066	0.000577814	0.046038099
cg12339425	-0.643270239	2.561230842	-3.983211802	0.000577885	0.046038099
cg14877963	-0.548392751	3.268897828	-3.983211259	0.000577886	0.046038099
cg16642408	-0.612394633	2.672215217	-3.983197431	0.000577906	0.046038099
cg16387372	0.569835305	3.802640059	3.983160465	0.000577959	0.046038099
cg11750794	-0.383847608	3.926974642	-3.983126362	0.000578008	0.046038099
cg01483119	-0.449029278	-2.114304787	-3.983098853	0.000578048	0.046038099
cg04138103	-0.829911509	1.960219062	-3.983054925	0.000578111	0.046038099
cg07813432	-0.540709739	3.748814883	-3.98303632	0.000578138	0.046038099
cg16415433	-0.714137262	1.954023747	-3.983017778	0.000578164	0.046038099
cg04799120	-0.608914805	1.078548119	-3.982955773	0.000578254	0.046038099
cg11056578	0.594104311	2.992594896	3.982913854	0.000578314	0.046038099
cg00235058	-0.484092832	3.800986125	-3.982890199	0.000578348	0.046038099
cg14946212	-0.572287781	3.308768497	-3.98285356	0.000578401	0.046038099
cg18977081	-0.583415968	-1.414992657	-3.982844164	0.000578415	0.046038099
cg25403930	-0.478302024	0.871479267	-3.982744198	0.000578559	0.046041101
cg13899538	-0.602968058	2.937567817	-3.982727841	0.000578582	0.046041101
cg00866811	0.654306875	2.897632435	3.982569766	0.00057881	0.046041962
cg12096406	-0.544651968	0.559777507	-3.98247881	0.000578942	0.046041962
cg07113653	0.640055207	2.064110273	3.982243001	0.000579282	0.046041962
cg12905869	-0.437077932	4.043066911	-3.982038092	0.000579578	0.046041962
cg15371881	-0.449900235	-1.264526199	-3.98201273	0.000579614	0.046041962
cg08266202	-0.424711393	-0.332577615	-3.981971037	0.000579675	0.046041962
cg13996609	-0.436789078	3.415214634	-3.981931676	0.000579731	0.046041962
cg15791948	-0.587986333	2.163553086	-3.981923573	0.000579743	0.046041962
cg11399974	-0.561387401	2.916792188	-3.981858657	0.000579837	0.046041962
cg12479973	-0.676197208	2.473825963	-3.981855826	0.000579841	0.046041962
cg04111054	-0.455727607	3.875603305	-3.981844339	0.000579858	0.046041962
cg20676956	-0.475968989	3.557153415	-3.981763202	0.000579975	0.046041962
cg15390650	-0.559945476	2.716190037	-3.981703042	0.000580062	0.046041962
cg00566369	-0.430384747	-0.985956549	-3.981658739	0.000580126	0.046041962
cg11634137	-0.674992118	2.780273947	-3.981631517	0.000580165	0.046041962
cg22595870	-0.412462222	4.086799639	-3.981475762	0.000580391	0.046041962
cg12557133	-0.419762231	3.447389497	-3.981468524	0.000580401	0.046041962
cg14214233	-0.707263775	1.665680691	-3.981376207	0.000580535	0.046041962
cg11809668	-1.067153707	0.355211734	-3.981370862	0.000580542	0.046041962
cg19030091	-0.423835211	0.273277654	-3.981316342	0.000580621	0.046041962
cg02480692	-0.720246816	1.901193267	-3.981284721	0.000580667	0.046041962
cg04703535	0.524401683	3.346905824	3.981216008	0.000580766	0.046041962
cg20147750	-0.646396878	3.349505316	-3.981208136	0.000580778	0.046041962
cg26650684	0.690298563	3.006290826	3.981194175	0.000580798	0.046041962
cg03208284	-0.605086516	2.10712954	-3.98115962	0.000580848	0.046041962
cg05878952	-0.775212736	1.260036695	-3.981142342	0.000580873	0.046041962
cg09681244	-0.614596529	3.29336251	-3.981031789	0.000581033	0.046041962

cg17969071	-0.491341357	3.452812783	-3.981028885	0.000581037	0.046041962
cg25518366	-0.489020561	3.404195856	-3.981001763	0.000581077	0.046041962
cg15972506	-0.641000544	-2.128306433	-3.980976764	0.000581113	0.046041962
cg15156059	-0.52503777	0.142080452	-3.980967539	0.000581126	0.046041962
cg15053915	-0.396240203	-1.69580789	-3.980902609	0.00058122	0.046041962
cg01455299	-0.5416931	4.271934514	-3.980877266	0.000581257	0.046041962
cg23961757	-0.626794075	2.698729365	-3.980766643	0.000581417	0.046041962
cg03670952	-0.565006425	2.914179128	-3.980743986	0.00058145	0.046041962
cg15001258	-0.575200142	3.698720864	-3.980687898	0.000581531	0.046041962
cg19399473	-0.515024141	2.8956704	-3.980682689	0.000581539	0.046041962
cg00755765	-0.691478974	-0.976181248	-3.980623217	0.000581625	0.046041962
cg18582759	-0.433857854	3.534255027	-3.980595901	0.000581665	0.046041962
cg12202171	-0.497085119	-1.276054253	-3.980542712	0.000581742	0.046041962
cg13520053	-0.425709049	0.107049176	-3.980537424	0.000581749	0.046041962
cg12092888	1.148838765	-3.965622209	3.980527816	0.000581763	0.046041962
cg14641092	-0.573074299	2.921419749	-3.980488772	0.00058182	0.046041962
cg01861257	-0.494387072	3.867668125	-3.980399453	0.000581949	0.046041962
cg09543693	0.59004806	1.461256726	3.980396219	0.000581954	0.046041962
cg11886905	-0.514210463	3.580971084	-3.980385244	0.00058197	0.046041962
cg24831850	-0.732263062	3.229889417	-3.980342475	0.000582032	0.046041962
cg10568409	-0.705754545	2.893954298	-3.980322078	0.000582062	0.046041962
cg11963753	-0.475411399	2.416582393	-3.980256828	0.000582156	0.046041962
cg26548251	-0.936650492	-0.555137591	-3.98024802	0.000582169	0.046041962
cg16387550	-0.446830828	3.740121699	-3.980200205	0.000582238	0.046041962
cg14802408	-0.399975344	3.923231772	-3.980195382	0.000582245	0.046041962
cg00856485	-0.490760191	3.579583649	-3.980165958	0.000582288	0.046041962
cg19765196	-0.710689423	2.623325587	-3.980144955	0.000582319	0.046041962
cg22926528	-0.458802146	3.222038842	-3.980113232	0.000582365	0.046041962
cg04380955	-0.535638179	2.628925589	-3.980076984	0.000582417	0.046041962
cg19082864	-0.532283595	0.146898779	-3.980060434	0.000582441	0.046041962
cg19313607	-0.557978851	3.603412898	-3.980032088	0.000582482	0.046041962
cg01279418	-0.821942574	0.427774173	-3.979781122	0.000582847	0.046041962
cg17100848	-0.595706881	3.889021109	-3.979767369	0.000582867	0.046041962
cg06828431	-0.432688197	3.838415654	-3.9797627	0.000582874	0.046041962
cg26338159	-0.621237426	1.694403705	-3.979754596	0.000582885	0.046041962
cg12078642	-0.372219509	4.033185419	-3.979703207	0.00058296	0.046041962
cg23519967	-0.611267207	2.005335639	-3.979700701	0.000582964	0.046041962
cg27622325	-0.476535114	3.703837678	-3.979696994	0.000582969	0.046041962
cg23562011	0.570563049	2.494910645	3.979695374	0.000582971	0.046041962
cg07807095	-0.741834177	3.184990469	-3.979683868	0.000582988	0.046041962
cg19881270	-0.693475918	2.24060816	-3.979596689	0.000583115	0.046041962
cg08893293	-0.520042992	3.907557278	-3.979592349	0.000583121	0.046041962
cg00184005	-0.675836511	3.10263858	-3.979553747	0.000583177	0.046041962
cg23772033	-0.620813689	3.044652452	-3.979532589	0.000583208	0.046041962
cg10809143	-0.55684404	3.650971797	-3.979392774	0.000583411	0.046052874
cg16253976	-0.552359144	1.772133679	-3.979212234	0.000583674	0.046068464
cg16848930	-0.513481916	2.791204657	-3.97902169	0.000583951	0.046075655

cg17441559	0.820284278	-3.332158444	3.978998947	0.000583984	0.046075655
cg05629821	-0.46859376	-0.512180414	-3.978983474	0.000584006	0.046075655
cg02908404	-0.7485661	0.713756157	-3.978926449	0.000584089	0.046075655
cg23260111	-0.719843826	2.378603784	-3.978896319	0.000584133	0.046075655
cg18080303	-0.574177682	-0.758424477	-3.978859076	0.000584188	0.046075655
cg12048536	-0.685831871	-3.515661552	-3.978805732	0.000584265	0.046075655
cg09447621	0.752158324	4.035522188	3.978792058	0.000584285	0.046075655
cg09630123	-0.485749328	3.063167498	-3.978652529	0.000584488	0.046079091
cg01347048	-0.698056964	-0.664765523	-3.978642637	0.000584503	0.046079091
cg14260162	0.45947022	2.621684966	3.978614992	0.000584543	0.046079091
cg11329853	-0.582309548	0.545090744	-3.978550426	0.000584637	0.046079091
cg24652205	-0.509321484	3.428672443	-3.97849497	0.000584718	0.046079091
cg27174137	-0.401615357	3.54368278	-3.97838056	0.000584885	0.046079091
cg10195698	-0.457746152	3.109532873	-3.978361944	0.000584912	0.046079091
cg17601661	-0.450187164	0.034174284	-3.978347573	0.000584933	0.046079091
cg06299718	-0.513126911	-0.328670638	-3.978315836	0.000584979	0.046079091
cg22792232	-0.568160225	2.643785951	-3.978267062	0.00058505	0.046079091
cg11094976	-0.718052635	1.850636038	-3.978221702	0.000585116	0.046079091
cg19144949	-0.552837619	3.104774967	-3.978157579	0.00058521	0.046079091
cg06581465	-0.439346357	-0.579960505	-3.978145363	0.000585227	0.046079091
cg09703616	-0.729040652	3.262012776	-3.97807245	0.000585334	0.046079091
cg18129781	-0.712329957	1.593760424	-3.978058258	0.000585354	0.046079091
cg22159954	-0.506791891	3.004895361	-3.978048005	0.000585369	0.046079091
cg12613238	-0.50509613	3.78180066	-3.977858597	0.000585646	0.046087097
cg11733738	-0.702147466	2.309423399	-3.977850165	0.000585658	0.046087097
cg00610000	-0.49875494	3.429634001	-3.977844521	0.000585666	0.046087097
cg13744967	-0.691028634	3.181244874	-3.977710991	0.000585861	0.046092223
cg07942500	-0.413884952	-0.32965847	-3.977710644	0.000585862	0.046092223
cg24327724	-0.444544011	3.77801446	-3.977666175	0.000585927	0.046092223
cg21067632	-0.371547962	4.218199572	-3.977557884	0.000586085	0.04609444
cg15907018	-0.46624717	3.581624717	-3.977557756	0.000586085	0.04609444
cg25780632	-0.469872007	3.359265447	-3.977423755	0.000586281	0.046104715
cg14585118	-0.641642268	3.514524354	-3.977248025	0.000586537	0.046118285
cg14970299	-0.539110973	3.661408943	-3.977198719	0.00058661	0.046118285
cg09258479	-0.477189325	-0.786675352	-3.977149764	0.000586681	0.046118285
cg10792660	-0.553861696	3.569843169	-3.977127493	0.000586714	0.046118285
cg25772185	-0.442699955	-0.525660714	-3.977064146	0.000586806	0.046120447
cg19950499	0.516217955	1.373853812	3.976976038	0.000586935	0.046125456
cg07245150	-0.392178062	-1.126060119	-3.976855923	0.000587111	0.046132125
cg16376447	-0.553288545	2.370554815	-3.976828749	0.000587151	0.046132125
cg12783569	-0.437054152	4.243129272	-3.97678448	0.000587215	0.046132125
cg18108818	-0.644322165	-0.657285652	-3.976613053	0.000587466	0.046146718
cg02925687	-0.472342215	3.528996468	-3.976522038	0.000587599	0.046152067
cg11023224	-0.458434272	0.153689157	-3.976387916	0.000587796	0.046152952
cg16674905	-0.547840077	3.538913867	-3.976367235	0.000587826	0.046152952
cg13375202	0.445395928	-4.390462315	3.976351998	0.000587849	0.046152952
cg23791245	-0.317531403	3.946753773	-3.976319189	0.000587897	0.046152952

cg00650911	-0.438541791	-1.307333752	-3.976288407	0.000587942	0.046152952
cg16034426	-0.584713354	-1.693967323	-3.976197808	0.000588074	0.046152952
cg17014647	-0.766382707	1.174215622	-3.976114245	0.000588197	0.046152952
cg23303554	-0.496435531	3.016438271	-3.976114235	0.000588197	0.046152952
cg07000559	-0.440493053	3.770505582	-3.976106841	0.000588208	0.046152952
cg14848333	-0.653886544	0.996948782	-3.976069619	0.000588262	0.046152952
cg10532188	0.715964892	-3.272245568	3.975960399	0.000588422	0.046156053
cg03024055	-0.614214859	2.04221564	-3.975864378	0.000588563	0.046156053
cg03108921	-0.423743354	3.726966199	-3.975846244	0.00058859	0.046156053
cg20686257	-0.432128562	3.124540185	-3.975758656	0.000588718	0.046156053
cg11374703	-0.441204624	0.606263168	-3.975748004	0.000588734	0.046156053
cg19846707	-0.517009568	3.790287605	-3.975722351	0.000588772	0.046156053
cg04982052	-0.526233719	3.153010078	-3.975692487	0.000588815	0.046156053
cg20284961	-0.633063803	-1.372794596	-3.975687221	0.000588823	0.046156053
cg09855125	-0.46159765	3.026021474	-3.975567822	0.000588998	0.046164679
cg14752911	-0.627217547	2.385617753	-3.97547847	0.000589129	0.046165084
cg26767237	-0.677392316	1.776198608	-3.975428749	0.000589202	0.046165084
cg20917551	-0.383964654	4.103207549	-3.975360853	0.000589302	0.046165084
cg21348937	-0.5108878	2.929879811	-3.975341891	0.00058933	0.046165084
cg26340657	-0.482642235	3.488480166	-3.97528819	0.000589409	0.046165084
cg00909286	-0.49228897	-0.741931169	-3.975286074	0.000589412	0.046165084
cg23210151	-0.379473897	3.823065921	-3.975253589	0.00058946	0.046165084
cg18901581	-0.605286418	2.591755285	-3.975208741	0.000589526	0.046165139
cg17897699	-0.496150248	3.437236211	-3.975116558	0.000589661	0.04617064
cg24122068	-0.423146868	3.70601985	-3.974989413	0.000589848	0.0461749
cg05976516	-0.590273977	3.50662345	-3.974984021	0.000589856	0.0461749
cg05028569	-0.384572469	3.345188008	-3.974946467	0.000589911	0.0461749
cg08142099	0.38588895	1.424372756	3.974795067	0.000590133	0.046175274
cg03586483	-0.643437956	3.046952733	-3.974781537	0.000590153	0.046175274
cg12289749	-0.757544496	1.609707494	-3.974772004	0.000590167	0.046175274
cg06444648	-0.813195635	3.049914386	-3.974741621	0.000590212	0.046175274
cg17228586	-0.441521101	2.9355099	-3.974721545	0.000590242	0.046175274
cg13984157	0.49649505	3.472095631	3.974664658	0.000590325	0.04617672
cg25958750	-0.497500656	3.165505191	-3.974369892	0.000590759	0.046196008
cg12026822	-0.475498371	3.144644969	-3.974325786	0.000590824	0.046196008
cg02202052	-0.534429391	-1.174232279	-3.974289141	0.000590878	0.046196008
cg06692153	-0.335690835	-1.650059975	-3.974225158	0.000590972	0.046196008
cg07718041	-0.811582575	1.956343636	-3.974198729	0.000591011	0.046196008
cg14344348	-0.485556647	3.738926094	-3.974168301	0.000591056	0.046196008
cg10797197	-0.440259843	-1.359543404	-3.974145101	0.00059109	0.046196008
cg05840756	0.445708677	2.449388322	3.974126163	0.000591118	0.046196008
cg11326420	-0.710061798	2.665843527	-3.974095242	0.000591163	0.046196008
cg24902067	-0.565670719	3.01625048	-3.974054131	0.000591224	0.046196008
cg20003613	-0.797073667	2.187343737	-3.973958071	0.000591365	0.04620197
cg26440297	-0.511811437	-3.013994321	-3.973884019	0.000591475	0.046205044
cg11006945	-0.402317858	3.739521134	-3.9738293	0.000591555	0.046205044
cg06636020	-0.461955869	0.026529547	-3.973798597	0.000591601	0.046205044

cg16541031	-0.597416537	-1.758410185	-3.973667625	0.000591794	0.046215029
cg21007509	-0.517971358	-0.643113802	-3.973604982	0.000591886	0.046217148
cg19868031	-0.606591793	2.298701503	-3.973523652	0.000592006	0.046219513
cg09304854	-0.447753662	0.954634132	-3.97349401	0.00059205	0.046219513
cg04065304	-0.467218044	-0.143495597	-3.973451747	0.000592112	0.046219513
cg27636962	-0.397955578	-0.10681904	-3.973329362	0.000592293	0.04622725
cg15600238	-0.479379235	3.670765089	-3.97328613	0.000592356	0.04622725
cg20930294	-0.41412231	0.011587958	-3.973251909	0.000592407	0.04622725
cg18590281	-0.582154035	3.502053827	-3.9730294	0.000592735	0.046247791
cg13893720	-0.705820338	1.082859371	-3.972881147	0.000592954	0.046251946
cg17248909	0.672944942	3.443854507	3.97282018	0.000593044	0.046251946
cg05115233	-0.534339814	4.718973523	-3.972765653	0.000593125	0.046251946
cg16435002	-0.494572927	3.278903365	-3.972744024	0.000593157	0.046251946
cg03317802	-0.561882158	2.465868589	-3.972704169	0.000593216	0.046251946
cg20431838	-0.574327547	3.116188574	-3.9726086	0.000593357	0.046251946
cg08843804	-0.787901323	2.561917673	-3.972605001	0.000593363	0.046251946
cg21415215	-0.562327062	3.472187728	-3.97258398	0.000593394	0.046251946
cg13916603	-0.587557672	2.626890636	-3.972556596	0.000593434	0.046251946
cg18850418	-0.511052277	-0.989126321	-3.972525301	0.00059348	0.046251946
cg06676366	-0.721004545	2.575288461	-3.972507367	0.000593507	0.046251946
cg06020767	-0.493674578	3.76488772	-3.972290279	0.000593828	0.04626281
cg04882739	-0.456411608	3.886150486	-3.972284975	0.000593836	0.04626281
cg22120395	-0.432432536	3.364585756	-3.972261506	0.000593871	0.04626281
cg21941987	-0.756873703	2.965847415	-3.972205988	0.000593953	0.04626281
cg20162200	-0.494245821	0.056773622	-3.972168992	0.000594007	0.04626281
cg16774355	-0.488786385	3.422262334	-3.972128688	0.000594067	0.04626281
cg23630568	-0.360128778	3.799784357	-3.972078565	0.000594141	0.04626281
cg01338834	-0.407865837	-1.717124062	-3.972060024	0.000594169	0.04626281
cg00728876	-0.479779263	3.424704062	-3.97191485	0.000594384	0.046265648
cg07790889	-0.507405412	3.183482209	-3.971910857	0.00059439	0.046265648
cg21682408	-0.565357371	3.037517537	-3.971777765	0.000594587	0.046265648
cg20732570	-0.48280301	3.39737039	-3.971743492	0.000594638	0.046265648
cg09096879	-0.408297538	-0.047428875	-3.971715147	0.00059468	0.046265648
cg05377560	-0.435490894	-0.143337388	-3.971653037	0.000594772	0.046265648
cg11173824	-0.491509328	3.002441411	-3.971614751	0.000594828	0.046265648
cg17161750	-0.579689831	2.389173343	-3.971600024	0.00059485	0.046265648
cg22075956	-0.591621461	3.1390517	-3.971599139	0.000594851	0.046265648
cg08480475	-0.823958322	2.656435778	-3.971594492	0.000594858	0.046265648
cg19693717	-0.441946372	3.96382929	-3.971501766	0.000594996	0.046271258
cg11148078	-0.428065052	3.76094126	-3.971337679	0.000595239	0.046274264
cg04625407	-0.543630571	4.314201102	-3.971292516	0.000595306	0.046274264
cg14688178	-0.474491231	2.60698915	-3.971255318	0.000595361	0.046274264
cg06370069	-0.51256509	-1.197325748	-3.971091023	0.000595605	0.046274264
cg08114979	-0.505333528	3.740288317	-3.970992454	0.000595751	0.046274264
cg18438515	-0.390661915	0.107171879	-3.97097774	0.000595773	0.046274264
cg04197554	-0.426392542	3.94830447	-3.970922978	0.000595854	0.046274264
cg04788706	-0.4837826	3.851352536	-3.970845645	0.000595969	0.046274264

cg26253981	-0.692465108	-1.511919362	-3.970831522	0.00059599	0.046274264
cg27378282	-0.711260983	2.653561122	-3.970813962	0.000596016	0.046274264
cg25436970	-0.611738756	2.448795863	-3.970802928	0.000596033	0.046274264
cg15204232	-0.583785189	3.293360871	-3.970753467	0.000596106	0.046274264
cg04902851	0.515250842	-4.153000221	3.970752967	0.000596107	0.046274264
cg25533247	0.361383025	-1.049602395	3.970730801	0.00059614	0.046274264
cg02426644	-0.481262303	3.191380497	-3.970600779	0.000596333	0.046274264
cg01950536	-0.536186838	2.701974648	-3.970569371	0.00059638	0.046274264
cg19009417	-0.487492133	-0.964774927	-3.970544037	0.000596417	0.046274264
cg21624833	-0.520749717	3.330159431	-3.970538603	0.000596425	0.046274264
cg20105652	-0.718787716	3.109230066	-3.970510377	0.000596467	0.046274264
cg01893462	-0.429951586	3.605025019	-3.970482814	0.000596508	0.046274264
cg20311843	-0.713301851	2.457250132	-3.970474262	0.000596521	0.046274264
cg09208162	-0.56164748	0.248093211	-3.970456327	0.000596548	0.046274264
cg01686553	-0.455587782	3.530097121	-3.970430375	0.000596586	0.046274264
cg09579094	-0.526245541	3.889009093	-3.970386342	0.000596652	0.046274264
cg11045529	-0.409187838	4.035936136	-3.970336521	0.000596726	0.046274264
cg15056638	-0.38573448	3.59002021	-3.970331674	0.000596733	0.046274264
cg24054272	-0.450807076	-1.84793605	-3.970265832	0.000596831	0.046276422
cg01403126	-0.468772636	2.639754281	-3.970207133	0.000596918	0.046276422
cg04893513	-0.354997169	1.121031939	-3.970181161	0.000596957	0.046276422
cg06868432	-0.503194992	-1.503157561	-3.970090541	0.000597091	0.046280055
cg06343233	-0.413829605	-0.328500132	-3.970025245	0.000597189	0.046280055
cg15970736	-0.532478624	3.236673171	-3.970017898	0.0005972	0.046280055
cg13417396	-0.519275473	4.036851059	-3.969973392	0.000597266	0.046280124
cg22154992	-0.411971565	0.541190027	-3.969663058	0.000597728	0.046286863
cg23270022	-0.38987309	0.276112001	-3.969659494	0.000597733	0.046286863
cg01181514	-0.450886242	4.244009292	-3.969645317	0.000597754	0.046286863
cg13978794	-0.563246961	3.518556538	-3.969620687	0.000597791	0.046286863
cg07701579	-0.513258714	1.066647	-3.969597084	0.000597826	0.046286863
cg07074138	0.530938505	-3.607920896	3.969589063	0.000597838	0.046286863
cg10390072	-0.686594291	2.684363671	-3.969586887	0.000597841	0.046286863
cg17726889	0.495666739	1.421585476	3.969563859	0.000597875	0.046286863
cg14380574	-0.55714628	-0.072316978	-3.969514741	0.000597949	0.046287007
cg10630035	-0.64261366	2.937568814	-3.969474888	0.000598008	0.046287007
cg10622920	-0.585433692	3.180208375	-3.969250979	0.000598342	0.04629564
cg25034709	-0.585867965	3.201105232	-3.969211916	0.0005984	0.04629564
cg09781747	-0.781610587	2.482306746	-3.969182887	0.000598443	0.04629564
cg24909753	-0.579391391	2.697484035	-3.969120795	0.000598536	0.04629564
cg19529621	-0.441801645	-1.225321611	-3.969119345	0.000598538	0.04629564
cg14278774	-0.512327453	0.562258808	-3.969096982	0.000598571	0.04629564
cg18126840	-0.476785103	3.257030592	-3.969044192	0.00059865	0.04629564
cg22347126	-0.65994781	-0.574875733	-3.969007703	0.000598704	0.04629564
cg17410410	-0.675498539	3.022976755	-3.968948253	0.000598793	0.04629564
cg05199232	-0.405042938	2.930098625	-3.968828764	0.000598971	0.04629564
cg24321566	-0.527681884	-0.022075672	-3.968819323	0.000598985	0.04629564
cg11533825	-0.461991989	0.216082745	-3.968741114	0.000599102	0.04629564

cg16393928	-0.476912726	-1.327919544	-3.968723477	0.000599129	0.04629564
cg10363549	-0.605453794	3.55035562	-3.968697693	0.000599167	0.04629564
cg19034038	-0.446384757	-1.926963112	-3.968642422	0.00059925	0.04629564
cg15003147	-0.432100489	4.189488543	-3.968618729	0.000599285	0.04629564
cg24721517	-0.629906548	2.90807415	-3.968526529	0.000599423	0.04629564
cg27574608	-0.593947769	2.587551915	-3.968522325	0.000599429	0.04629564
cg27185183	-0.426130408	4.036716702	-3.968455756	0.000599528	0.04629564
cg15218862	-0.53966996	3.084689029	-3.968454946	0.000599529	0.04629564
cg14808684	-0.502351251	2.764823677	-3.96843919	0.000599553	0.04629564
cg10784597	-0.522415329	2.669630161	-3.968342524	0.000599697	0.04629564
cg14398342	-0.705653632	1.980988111	-3.968325197	0.000599723	0.04629564
cg22624306	-0.680926769	0.910708835	-3.96832012	0.000599731	0.04629564
cg13540735	-0.477143362	3.352202209	-3.968230793	0.000599864	0.04629564
cg01682915	-0.835362661	1.679923474	-3.968190942	0.000599924	0.04629564
cg13514049	-0.963574025	-2.543596681	-3.968163151	0.000599965	0.04629564
cg27551597	-0.535756182	3.148802004	-3.968157686	0.000599974	0.04629564
cg20088961	-0.424873983	0.427250536	-3.968103648	0.000600054	0.04629564
cg02286506	0.430622257	2.366357609	3.968071078	0.000600103	0.04629564
cg21403156	-0.471132051	3.391507308	-3.96804271	0.000600145	0.04629564
cg25421357	-0.769893093	2.434068797	-3.967978185	0.000600242	0.046298041
cg24677005	1.007216478	-2.778245272	3.967773192	0.000600549	0.046314412
cg12739137	-0.674017832	1.512901611	-3.967748869	0.000600585	0.046314412
cg21577159	-0.4456798	4.139279728	-3.967597913	0.000600811	0.046320898
cg25594307	-0.371182163	-1.717209425	-3.967463486	0.000601012	0.046320898
cg16017521	0.322531867	3.119428085	3.96746025	0.000601017	0.046320898
cg03803806	-0.446428166	-1.255145626	-3.967443275	0.000601042	0.046320898
cg02928644	-0.554281776	-1.342643533	-3.9674413	0.000601045	0.046320898
cg17850883	-0.581459734	2.751545344	-3.96743056	0.000601061	0.046320898
cg05516950	-0.475708962	4.103376234	-3.967339021	0.000601199	0.046326423
cg25975347	-0.588323199	3.150313455	-3.967281806	0.000601284	0.046327987
cg22312091	-0.583167658	3.204518211	-3.967160609	0.000601466	0.046335784
cg15593510	-0.602151033	3.013673265	-3.967126941	0.000601516	0.046335784
cg21504730	0.416695749	2.656608569	3.967037996	0.00060165	0.046341013
cg13986884	-0.753505532	0.804304398	-3.966889857	0.000601872	0.046342982
cg19760004	-0.417647752	0.056509263	-3.966880702	0.000601885	0.046342982
cg00688819	-0.80542286	-1.816798642	-3.966870488	0.000601901	0.046342982
cg07009098	-0.444614256	3.713646165	-3.966846394	0.000601937	0.046342982
cg07711025	-0.53412623	-0.855558103	-3.966789469	0.000602022	0.046344518
cg09487891	-0.679285202	-0.860557249	-3.966745027	0.000602089	0.046344613
cg10823956	-0.327590912	-0.109569936	-3.966663328	0.000602211	0.04634901
cg16550959	-0.568528924	2.051677788	-3.966566041	0.000602357	0.046355208
cg10423842	-0.368492587	0.678717699	-3.966414554	0.000602585	0.046367668
cg10518961	-0.678871396	2.688810048	-3.966366137	0.000602657	0.046368224
cg06573795	-0.416009545	3.151319893	-3.96620713	0.000602896	0.046377455
cg16481285	-0.535893135	1.198886356	-3.966199043	0.000602908	0.046377455
cg00752601	-0.587423477	2.529727573	-3.966145805	0.000602988	0.04637857
cg18708661	-0.526443043	3.45340046	-3.966085824	0.000603078	0.046380464

cg02931208	-0.339387925	-0.183221085	-3.965875257	0.000603395	0.046395168
cg26627593	-0.541623577	3.258678526	-3.965871432	0.000603401	0.046395168
cg09323275	-0.389801509	4.259911905	-3.965764404	0.000603561	0.046395806
cg12702873	-0.580486934	3.806756557	-3.965763388	0.000603563	0.046395806
cg04365182	-0.374328276	-0.268598946	-3.965735223	0.000603605	0.046395806
cg21864318	-0.454678562	2.250328495	-3.96560755	0.000603797	0.046400696
cg15775787	-0.452159785	4.315813756	-3.965605809	0.0006038	0.046400696
cg05027035	-0.341959285	3.169750903	-3.965540774	0.000603898	0.04640318
cg08858295	-0.536582829	0.371496784	-3.965321287	0.000604228	0.046412852
cg09804944	-0.751572827	3.012393917	-3.965310891	0.000604244	0.046412852
cg08982224	-0.386098603	-0.238897251	-3.965285326	0.000604282	0.046412852
cg04794159	-0.417729017	0.460997781	-3.965282976	0.000604286	0.046412852
cg03450107	-0.363062	-0.612397217	-3.965187435	0.00060443	0.046418867
cg07054763	-0.479926025	0.946417775	-3.96512878	0.000604518	0.046420617
cg13675329	-0.480786202	3.42375318	-3.965080872	0.00060459	0.046421125
cg27099480	0.57576983	1.728588613	3.965033752	0.000604661	0.046421543
cg00711743	-0.438484239	3.805813776	-3.964935229	0.000604809	0.046422441
cg26203738	-0.437596362	3.139378403	-3.964925786	0.000604824	0.046422441
cg12904680	0.509317877	2.428425034	3.96479969	0.000605014	0.046422441
cg06781086	-0.583670365	2.520269187	-3.964744384	0.000605097	0.046422441
cg23245586	-0.435974601	3.273087833	-3.964722463	0.00060513	0.046422441
cg15828403	0.791514175	-2.717101947	3.964716621	0.000605139	0.046422441
cg21630243	-0.558516121	3.575344711	-3.964695962	0.00060517	0.046422441
cg02317746	-0.481774262	3.687378074	-3.964637494	0.000605258	0.046422441
cg05816006	-0.693552815	2.181970281	-3.964634621	0.000605262	0.046422441
cg14123750	-0.473403581	0.129450422	-3.964586643	0.000605335	0.046422963
cg02479671	-0.462508995	3.209405653	-3.964300992	0.000605766	0.046449376
cg00216760	-0.519845284	-0.372832522	-3.964271396	0.00060581	0.046449376
cg00183590	-0.404578301	4.109403939	-3.9641399	0.000606009	0.046459565
cg06152865	-0.421812598	-1.601369942	-3.963909711	0.000606356	0.046478927
cg06706898	0.432411767	3.347758312	3.963885752	0.000606393	0.046478927
cg21802773	-0.613959523	3.526706473	-3.963742879	0.000606608	0.046482007
cg15224626	-0.68826241	1.723738993	-3.963708179	0.000606661	0.046482007
cg00480115	-0.488163944	0.267884883	-3.963674716	0.000606712	0.046482007
cg02418175	-0.419264753	-0.419517543	-3.963645981	0.000606755	0.046482007
cg13279848	-0.874489125	2.036206501	-3.963642022	0.000606761	0.046482007
cg01158239	-0.490695809	0.429318598	-3.963528661	0.000606932	0.046483417
cg13054321	0.610565753	2.869045286	3.963508116	0.000606963	0.046483417
cg17657636	-0.366954503	-1.050751691	-3.963472985	0.000607016	0.046483417
cg11778756	-0.637755639	2.930148362	-3.963456233	0.000607042	0.046483417
cg20126910	0.740669671	3.205203257	3.963357499	0.000607191	0.046489825
cg23913078	-0.459887343	3.904642065	-3.963175874	0.000607466	0.046502745
cg01620602	-0.632989609	-1.490666993	-3.963159172	0.000607491	0.046502745
cg07712738	-0.593734548	2.812984177	-3.962973566	0.000607772	0.046519223
cg16290930	-0.476077954	2.142798567	-3.962917171	0.000607857	0.046520732
cg08371406	-0.590315996	3.691951326	-3.962810688	0.000608019	0.046524007
cg20886194	-0.46485311	3.062698283	-3.962721769	0.000608153	0.046524007

cg04833516	-0.453148716	0.629380663	-3.96269987	0.000608187	0.046524007
cg17345620	-0.456523013	3.144138222	-3.962670053	0.000608232	0.046524007
cg04952263	-0.500089187	4.026819648	-3.962629949	0.000608293	0.046524007
cg16321672	-0.583844926	2.365809966	-3.962563633	0.000608393	0.046524007
cg12975061	-0.436186415	3.102135097	-3.962531228	0.000608442	0.046524007
cg11737757	-0.411726234	-0.468222439	-3.962444207	0.000608574	0.046524007
cg17335387	-0.511750256	-0.231734453	-3.962424925	0.000608603	0.046524007
cg02110475	-0.717766653	0.684845337	-3.962364468	0.000608695	0.046524007
cg21408351	-0.617239002	2.234780927	-3.962353195	0.000608712	0.046524007
cg06162198	-0.635806573	2.55058287	-3.962340654	0.000608731	0.046524007
cg03260563	-0.661606771	2.849981733	-3.962325469	0.000608754	0.046524007
cg21722227	-0.427452979	4.065191573	-3.962201587	0.000608942	0.046524817
cg18314607	-0.431798434	3.827239872	-3.962141729	0.000609033	0.046524817
cg09370849	-0.778224418	-2.32128271	-3.962130311	0.00060905	0.046524817
cg17888482	-0.535268964	3.425052114	-3.962126698	0.000609056	0.046524817
cg08384843	-0.51424589	3.529990828	-3.962101972	0.000609093	0.046524817
cg25479340	-0.653327171	2.715126518	-3.961940214	0.000609339	0.046532112
cg07249433	-0.818107378	-1.293982354	-3.961920581	0.000609368	0.046532112
cg25741748	-0.731935665	2.49903447	-3.961841256	0.000609489	0.046532112
cg12838902	-0.416099801	-0.58289844	-3.961833935	0.0006095	0.046532112
cg13385408	-0.442999017	-0.008508884	-3.961822643	0.000609517	0.046532112
cg02102635	-0.616290494	2.299002269	-3.96171964	0.000609673	0.046539037
cg22601570	-0.578298193	0.684473138	-3.961587982	0.000609873	0.046549286
cg25158649	-0.393829173	3.728656402	-3.961494325	0.000610016	0.046550797
cg10476585	-0.543352876	3.73297822	-3.961488444	0.000610025	0.046550797
cg11373806	-0.630754113	3.557728019	-3.961366033	0.000610211	0.046551554
cg12291247	-0.499595883	2.958606318	-3.961319172	0.000610282	0.046551554
cg25756406	-0.396354833	-0.573198296	-3.961310612	0.000610295	0.046551554
cg18502070	-0.455545759	3.588378324	-3.96128369	0.000610336	0.046551554
cg24757533	-0.521722517	-1.823325564	-3.96121015	0.000610448	0.046551554
cg06901621	-0.445764664	-0.288542885	-3.961199213	0.000610464	0.046551554
cg26053175	-0.470539678	-0.681945739	-3.961179117	0.000610495	0.046551554
cg17544079	0.614681829	2.106731371	3.961136079	0.00061056	0.046551554
cg18606115	-0.556738803	2.545119385	-3.961025789	0.000610728	0.046555878
cg18537721	-0.58999	3.35578401	-3.961012378	0.000610748	0.046555878
cg13259711	-0.854681288	0.116395187	-3.960833007	0.000611021	0.046570138
cg25842677	-0.409907338	4.045375901	-3.960777532	0.000611106	0.046570138
cg19759064	-0.42648274	-2.422969605	-3.960661625	0.000611282	0.046570138
cg10575686	-0.575092447	-3.29056134	-3.960661072	0.000611283	0.046570138
cg14920359	-0.550595366	-0.362873	-3.96065533	0.000611292	0.046570138
cg11760345	0.642508133	3.267869651	3.960630305	0.00061133	0.046570138
cg27402766	-0.597251825	3.88861506	-3.960377304	0.000611715	0.046570295
cg20238589	-0.46181293	0.380995473	-3.960317441	0.000611806	0.046570295
cg03779603	-0.426003928	-1.745234365	-3.960284023	0.000611857	0.046570295
cg05867832	-0.487807936	3.632432843	-3.960282503	0.00061186	0.046570295
cg01352906	0.391476029	1.830987855	3.960258072	0.000611897	0.046570295
cg27011350	-0.379457065	3.96983013	-3.960237178	0.000611929	0.046570295

cg00943191	-0.594086158	2.698882519	-3.96022595	0.000611946	0.046570295
cg06651848	0.580674962	-3.100443771	3.960217802	0.000611958	0.046570295
cg10144863	-0.874235931	-0.145940654	-3.960212878	0.000611966	0.046570295
cg09165129	-0.510810063	3.341082447	-3.959996568	0.000612296	0.046570295
cg08415118	-0.527291317	3.45784249	-3.959980995	0.000612319	0.046570295
cg24953956	-0.398304508	-0.484708126	-3.959970864	0.000612335	0.046570295
cg09236445	-0.424833936	3.546242721	-3.959956494	0.000612357	0.046570295
cg06161933	-0.901893966	2.061396578	-3.959901081	0.000612441	0.046570295
cg22901975	-0.515366112	3.344065746	-3.959842617	0.00061253	0.046570295
cg24509697	-0.32327392	-0.756393053	-3.959834143	0.000612543	0.046570295
cg10902693	-0.716798842	2.376104026	-3.959804676	0.000612588	0.046570295
cg21179803	-0.521805934	4.337933754	-3.959798811	0.000612597	0.046570295
cg17338470	-0.466370864	4.303559623	-3.959775323	0.000612633	0.046570295
cg01166827	0.574597807	2.688238605	3.959766389	0.000612647	0.046570295
cg03523626	0.619225667	2.830796816	3.95962305	0.000612866	0.046570982
cg13197256	-0.401943585	3.449543378	-3.959530503	0.000613007	0.046570982
cg14552514	-0.701548975	-0.975547502	-3.959493093	0.000613064	0.046570982
cg26551200	-0.633816613	-3.799141733	-3.959469332	0.0006131	0.046570982
cg16681471	0.68225335	2.265369	3.95937941	0.000613238	0.046570982
cg06755448	-0.470382491	0.270308846	-3.959372169	0.000613249	0.046570982
cg14837557	-0.500781503	2.975957605	-3.959344309	0.000613291	0.046570982
cg11611320	-0.553165639	4.03308394	-3.95931283	0.000613339	0.046570982
cg11207705	-0.619033	2.929268836	-3.959302321	0.000613355	0.046570982
cg09080729	-0.573852196	-3.180572615	-3.959297949	0.000613362	0.046570982
cg08030248	-0.460829126	0.866409599	-3.959286836	0.000613379	0.046570982
cg22945467	-0.364197297	0.364345873	-3.959243669	0.000613445	0.046570998
cg05921271	-0.483370277	2.641484257	-3.959195231	0.000613519	0.046571006
cg20301210	-0.694427427	2.502111649	-3.959157552	0.000613577	0.046571006
cg16678355	-0.523645122	3.570258533	-3.959072834	0.000613706	0.04657101
cg21931839	0.635976874	-2.463874324	3.959059043	0.000613727	0.04657101
cg02327109	-0.344344948	1.153032612	-3.959005793	0.000613809	0.04657101
cg19329529	0.611431466	1.697714069	3.958985485	0.00061384	0.04657101
cg15125476	-0.488418443	3.154492672	-3.958837503	0.000614066	0.04657103
cg03289219	0.431960958	1.394358462	3.958829166	0.000614079	0.04657103
cg20079886	-0.513112146	4.006629091	-3.958793566	0.000614133	0.04657103
cg17018569	-0.446716308	3.117952131	-3.958688752	0.000614293	0.04657103
cg02606200	-0.520642904	-0.499432352	-3.95867584	0.000614313	0.04657103
cg10592033	-0.609488247	0.110218463	-3.958650343	0.000614352	0.04657103
cg18773571	-0.609107454	3.020662396	-3.958610251	0.000614414	0.04657103
cg24726571	-0.659038465	-2.205542657	-3.958606884	0.000614419	0.04657103
cg00503760	-0.609577817	2.529271982	-3.958579393	0.000614461	0.04657103
cg08176140	-0.471218172	0.273861617	-3.958548691	0.000614508	0.04657103
cg16758272	-0.622328061	2.733070221	-3.958466349	0.000614634	0.04657103
cg07151059	-0.535810622	3.140357875	-3.958437475	0.000614678	0.04657103
cg15877831	-0.840788378	1.511038699	-3.958426695	0.000614694	0.04657103
cg15844374	-0.470015142	-0.531005026	-3.958357495	0.0006148	0.04657176
cg20019217	0.536635365	-3.882769465	3.958300553	0.000614888	0.04657176

cg15827202	-0.581294687	3.479494442	-3.958291601	0.000614901	0.04657176
cg24341059	-0.63623315	2.571708563	-3.958195046	0.000615049	0.046572452
cg20483374	-0.622627288	-1.176468798	-3.958188136	0.00061506	0.046572452
cg13372624	-0.537856769	3.043387507	-3.9581226	0.00061516	0.046572452
cg01869804	-0.579798091	3.238405941	-3.958095795	0.000615201	0.046572452
cg14428628	-0.427573077	3.059756009	-3.958018562	0.00061532	0.046572452
cg14798255	-0.440707906	3.472658787	-3.957946608	0.00061543	0.046572452
cg13705556	-0.364897635	0.397216986	-3.957938571	0.000615442	0.046572452
cg11522044	-0.446303497	3.542638325	-3.957937987	0.000615443	0.046572452
cg01843489	-0.569609816	3.420801845	-3.957899471	0.000615502	0.046572452
cg19957506	-0.530174725	3.937597592	-3.957674251	0.000615848	0.046592767
cg06787622	-0.61783425	2.99464857	-3.957593629	0.000615971	0.046592767
cg04905719	-0.573893779	-1.672677474	-3.957581048	0.000615991	0.046592767
cg08446321	-0.439589692	3.901276165	-3.957552866	0.000616034	0.046592767
cg00920244	-0.412446818	0.148152704	-3.957331719	0.000616373	0.046603618
cg02182592	-0.344064525	-0.631158303	-3.957309579	0.000616407	0.046603618
cg21683459	-0.492049135	-1.781522509	-3.957273821	0.000616462	0.046603618
cg15604733	-0.47878117	4.113738394	-3.95726093	0.000616482	0.046603618
cg27240008	-0.395101828	0.337464988	-3.957233837	0.000616523	0.046603618
cg05949913	-0.814759941	1.872791665	-3.957202236	0.000616572	0.046603618
cg19953253	-0.854186723	1.014672875	-3.957039748	0.000616822	0.046617508
cg09799441	-0.645924348	2.963399767	-3.956969168	0.00061693	0.046620729
cg17987666	-0.603222365	2.806133911	-3.956741312	0.00061728	0.046638742
cg04924893	-0.412361745	3.227589147	-3.95672841	0.0006173	0.046638742
cg12544989	-0.744985365	1.501813358	-3.956673321	0.000617385	0.046640167
cg06207961	0.727455069	1.649920109	3.956617457	0.000617471	0.046641683
cg21910634	-0.517027858	3.759070987	-3.956501605	0.000617649	0.046646779
cg02700878	2.835114327	0.875559447	3.95648797	0.00061767	0.046646779
cg20655237	-0.425765914	4.01824248	-3.956180339	0.000618143	0.046669435
cg12453582	-0.499930236	-0.683975723	-3.956114595	0.000618244	0.046669435
cg15999997	-0.494270715	3.735166249	-3.956087648	0.000618286	0.046669435
cg16360576	-0.628513812	2.433602923	-3.956082565	0.000618294	0.046669435
cg18603613	0.48840992	1.634540205	3.956079029	0.000618299	0.046669435
cg01314530	-0.565921134	0.764226069	-3.955876652	0.000618611	0.046675026
cg22890747	-0.504054234	-1.304997477	-3.955786596	0.00061875	0.046675026
cg01874151	-0.538236463	3.631311703	-3.955779643	0.00061876	0.046675026
cg06281719	0.552521303	-4.340199997	3.955730641	0.000618836	0.046675026
cg07805644	-0.744778641	2.025891716	-3.955727611	0.000618841	0.046675026
cg17282875	-0.604426146	1.262778954	-3.955698617	0.000618885	0.046675026
cg02015348	-0.512041042	3.215170069	-3.955693299	0.000618893	0.046675026
cg11480684	-0.464649742	-0.464654779	-3.955688804	0.0006189	0.046675026
cg07489153	-0.417962927	3.65618895	-3.955587789	0.000619056	0.0466818
cg10411143	-0.810287149	2.626040892	-3.955533695	0.00061914	0.04668312
cg02413776	-0.496795822	-0.913862679	-3.955386409	0.000619367	0.046693147
cg16087867	-0.727571065	2.516132065	-3.955334825	0.000619446	0.046693147
cg04829348	-0.523392564	3.523835239	-3.955319267	0.00061947	0.046693147
cg01950921	-0.91625166	2.359487093	-3.955235106	0.0006196	0.046697966

cg23040173	-0.420955445	4.115029422	-3.955095877	0.000619815	0.046699936
cg06683316	-0.380807371	0.203327294	-3.954995749	0.00061997	0.046699936
cg11401011	-0.485965875	3.094343955	-3.954904536	0.00062011	0.046699936
cg25485092	-0.729556988	2.632138671	-3.954897738	0.000620121	0.046699936
cg18151410	-0.528747996	-0.556259878	-3.954868061	0.000620167	0.046699936
cg01248567	-0.563469615	3.436691742	-3.954865524	0.000620171	0.046699936
cg15018620	-1.252246417	1.961019437	-3.954851798	0.000620192	0.046699936
cg16595750	-0.393934688	-1.167433223	-3.954731606	0.000620378	0.046699936
cg14464434	-0.567146117	3.30580669	-3.954722234	0.000620392	0.046699936
cg09346655	-0.494418941	3.833822276	-3.954676651	0.000620462	0.046699936
cg10903555	-0.635800081	-1.142729401	-3.954671639	0.00062047	0.046699936
cg14308311	-0.662961055	1.12696954	-3.954592453	0.000620593	0.046699936
cg13585861	-0.460190823	0.068520683	-3.954564942	0.000620635	0.046699936
cg14685886	-0.4857907	2.901717315	-3.95455545	0.00062065	0.046699936
cg07654202	-0.319049145	0.228664294	-3.954525147	0.000620697	0.046699936
cg23165274	-0.432196026	0.716269264	-3.95450541	0.000620727	0.046699936
cg18006647	-0.826262201	0.447921599	-3.954409513	0.000620875	0.046699936
cg19062940	-0.471442067	3.413253679	-3.954407957	0.000620878	0.046699936
cg15659713	0.527179698	-1.032304631	3.954368255	0.000620939	0.046699936
cg22097004	-0.50983178	3.718936507	-3.954332232	0.000620995	0.046699936
cg04251828	-0.456674271	0.124469731	-3.95418399	0.000621224	0.046699936
cg19543969	-0.693119283	2.755957061	-3.954137087	0.000621297	0.046699936
cg03189545	-0.416099196	3.625678675	-3.954123316	0.000621318	0.046699936
cg05389236	-0.551971296	-2.116337438	-3.954046095	0.000621438	0.046699936
cg15707816	-0.7098201	1.707646596	-3.954014823	0.000621486	0.046699936
cg10778880	-0.426810017	1.090836498	-3.95401358	0.000621488	0.046699936
cg11780323	-0.535086554	2.970232193	-3.953969087	0.000621557	0.046699936
cg07492055	-0.636135153	2.790622678	-3.953940158	0.000621602	0.046699936
cg05937392	-0.416012936	4.051976099	-3.953882017	0.000621692	0.046699936
cg22871336	-0.502341728	-0.807929079	-3.953851033	0.00062174	0.046699936
cg09756225	-0.502620688	-1.84457235	-3.953848382	0.000621744	0.046699936
cg09912548	-0.540692455	3.414096605	-3.953756796	0.000621885	0.046699936
cg00684101	-0.430823681	3.2615932	-3.953728923	0.000621929	0.046699936
cg16008536	-0.47779789	-2.836221886	-3.953656053	0.000622041	0.046699936
cg10244313	-0.637547717	3.885656754	-3.953645836	0.000622057	0.046699936
cg02057729	-0.420605709	4.128523055	-3.953590139	0.000622144	0.046699936
cg03331935	-0.408622637	0.488794292	-3.953564795	0.000622183	0.046699936
cg05468433	-0.506916948	0.862148246	-3.953550734	0.000622205	0.046699936
cg25813582	0.641920481	-4.552255716	3.953516808	0.000622257	0.046699936
cg07364962	-0.450622328	-0.004302758	-3.953512934	0.000622263	0.046699936
cg01075966	-0.421580566	3.793067998	-3.953318476	0.000622565	0.046701811
cg03436208	-0.501070449	0.255279595	-3.953251709	0.000622668	0.046701811
cg23072419	-0.50021806	-0.167141524	-3.953238787	0.000622688	0.046701811
cg05563740	-0.442376661	2.940627041	-3.953201092	0.000622747	0.046701811
cg17834425	-0.605918852	4.396686467	-3.953193755	0.000622758	0.046701811
cg04576764	-0.334013842	0.446903705	-3.953184046	0.000622773	0.046701811
cg03775901	-0.48209626	-0.003671524	-3.953138198	0.000622844	0.046701811

cg25178388	-0.53368248	3.447605177	-3.953107555	0.000622892	0.046701811
cg19356612	-0.436366162	3.625697934	-3.953097164	0.000622908	0.046701811
cg09506001	-0.533316456	-0.988170074	-3.953049385	0.000622982	0.046701811
cg18106800	-0.393641838	-2.931980022	-3.952994797	0.000623067	0.046701811
cg11888928	-0.387224935	4.263027201	-3.95295551	0.000623128	0.046701811
cg05907504	0.466761642	3.89756899	3.95294415	0.000623145	0.046701811
cg13874439	-0.561305377	2.985708374	-3.952808723	0.000623355	0.046707856
cg22030501	-0.398488257	3.699817474	-3.952721883	0.00062349	0.046707856
cg04337594	-0.529587102	0.141556293	-3.9527154	0.0006235	0.046707856
cg15242517	-0.312751428	-1.327585488	-3.952713241	0.000623504	0.046707856
cg23975646	-0.509521186	-0.010232507	-3.952679816	0.000623556	0.046707856
cg11513378	-0.470076719	3.758226623	-3.952578187	0.000623713	0.046714739
cg15129362	-0.479643011	-0.178942208	-3.95249016	0.00062385	0.046720041
cg21580456	-0.388064751	-0.573502365	-3.952397756	0.000623994	0.046723045
cg01323835	-0.411940274	-0.637858928	-3.952372928	0.000624032	0.046723045
cg12597033	-0.404823282	-1.09482562	-3.952304375	0.000624139	0.046723045
cg14037182	-0.373913209	-0.561197569	-3.952276724	0.000624182	0.046723045
cg15553770	-0.576416462	3.745787448	-3.952139168	0.000624396	0.046723045
cg26910920	-0.52858045	3.673815432	-3.952123484	0.00062442	0.046723045
cg18708810	-0.689801086	1.43307674	-3.952090981	0.000624471	0.046723045
cg14102434	-0.466305137	3.164755999	-3.952044703	0.000624543	0.046723045
cg21516718	-0.448880194	-0.647749978	-3.95204191	0.000624547	0.046723045
cg23450912	-0.549548369	3.497169481	-3.952040038	0.00062455	0.046723045
cg24321676	-0.579613314	3.457050781	-3.951983076	0.000624638	0.046724739
cg04334746	-0.837345446	1.270068963	-3.951925144	0.000624729	0.046726546
cg01861885	-0.436967112	1.197680261	-3.951799489	0.000624924	0.046734896
cg24651908	-0.539852103	-0.309170595	-3.951757064	0.00062499	0.046734896
cg04990703	-0.446595497	-0.273439845	-3.951726215	0.000625038	0.046734896
cg20656595	0.322557989	0.944216815	3.951627547	0.000625192	0.046741447
cg19586112	-0.430086999	-0.756731055	-3.951522058	0.000625356	0.046742963
cg05710512	0.404825107	2.058608314	3.951513756	0.000625369	0.046742963
cg01215511	-0.382033704	0.282384783	-3.95148739	0.00062541	0.046742963
cg26737948	-0.464301193	3.960220077	-3.951352733	0.00062562	0.046744176
cg10523399	0.521809328	2.9328242	3.951317114	0.000625675	0.046744176
cg06719000	-0.411593322	4.332857193	-3.951312471	0.000625682	0.046744176
cg07073321	-0.41231401	3.912923015	-3.951274538	0.000625742	0.046744176
cg10959591	-0.444268766	3.040905105	-3.951211376	0.00062584	0.046744176
cg26217053	-0.480205786	3.601516308	-3.951185783	0.00062588	0.046744176
cg09052865	-0.384145897	0.645708868	-3.951138694	0.000625953	0.046744176
cg12371147	-0.79092867	1.270841895	-3.951124948	0.000625975	0.046744176
cg19023847	-0.415689565	-0.195183731	-3.951095835	0.00062602	0.046744176
cg15053979	-0.590579585	2.448239684	-3.950863732	0.000626382	0.046763119
cg06731295	-0.474204028	-0.593055343	-3.950736617	0.00062658	0.046763119
cg18331083	-0.452258016	3.59261378	-3.950690341	0.000626653	0.046763119
cg16776331	-0.547550549	4.131886954	-3.950685383	0.00062666	0.046763119
cg02346077	-0.407223752	4.044665046	-3.950645113	0.000626723	0.046763119
cg06109846	-0.445535837	3.894996407	-3.950555657	0.000626863	0.046763119

cg27371451	-0.696955902	2.044198995	-3.950551708	0.000626869	0.046763119
cg17592363	-0.421136596	0.452615046	-3.950536538	0.000626893	0.046763119
cg18152167	-0.530755641	3.444320639	-3.950513206	0.000626929	0.046763119
cg15218589	-0.485934137	3.083585466	-3.950510062	0.000626934	0.046763119
cg15862420	-0.546147915	-1.13699171	-3.950355692	0.000627175	0.046767295
cg00146085	-0.451865868	3.892665994	-3.95032006	0.000627231	0.046767295
cg11772665	-0.567768842	0.219912826	-3.95031452	0.000627239	0.046767295
cg05944877	-0.446416618	3.783362011	-3.950268833	0.000627311	0.046767295
cg21888059	-0.431036031	0.253958734	-3.950246495	0.000627346	0.046767295
cg10011976	-0.368232935	3.53933853	-3.950190375	0.000627433	0.046767295
cg02593601	-0.572621547	3.713314655	-3.95014954	0.000627497	0.046767295
cg07482226	-0.514971417	3.464039098	-3.95013607	0.000627518	0.046767295
cg27377225	-0.7848794	1.949183902	-3.950059332	0.000627638	0.046771312
cg08636240	-0.478034785	3.456014877	-3.94997221	0.000627774	0.046771843
cg02450267	-0.39981174	0.364590653	-3.94997029	0.000627777	0.046771843
cg03636048	-0.510490997	3.0877213	-3.949922715	0.000627852	0.046772465
cg04415451	-0.327477903	4.167439206	-3.949838391	0.000627983	0.046777368
cg11035003	-0.423329775	4.18047755	-3.949763427	0.000628101	0.046778357
cg22346500	-0.363288753	-0.832163053	-3.949714246	0.000628178	0.046778357
cg10006393	-0.586210317	2.889996029	-3.949611077	0.000628339	0.046778357
cg02731032	-0.433010465	-0.41934115	-3.949597634	0.00062836	0.046778357
cg25387578	-0.620638377	2.337098622	-3.949577572	0.000628391	0.046778357
cg22223182	-0.577520338	1.300760204	-3.949501062	0.000628511	0.046778357
cg06508320	-0.737288767	-3.839868947	-3.949454509	0.000628584	0.046778357
cg26277433	-0.439392131	-0.645207532	-3.949439674	0.000628607	0.046778357
cg01049355	-0.512331113	3.728550913	-3.949416033	0.000628644	0.046778357
cg02312317	-0.405323612	3.416452936	-3.949407835	0.000628657	0.046778357
cg26454724	-0.475130558	-1.155454317	-3.949294598	0.000628834	0.046784399
cg27075810	0.533634441	1.929590694	3.949271611	0.00062887	0.046784399
cg17439694	-0.546674942	3.479440622	-3.949127494	0.000629096	0.046787494
cg02447471	-0.592629705	3.534038263	-3.949086902	0.00062916	0.046787494
cg09951596	-0.718056125	-0.967812319	-3.94900664	0.000629285	0.046787494
cg16154816	0.450772295	-4.352682869	3.948993638	0.000629306	0.046787494
cg02908609	0.814369996	4.110155712	3.948975074	0.000629335	0.046787494
cg14153852	-0.45284218	-2.428774985	-3.94897078	0.000629342	0.046787494
cg08527837	-0.409620733	4.459827302	-3.948949925	0.000629374	0.046787494
cg11251728	-0.521206302	-1.082899784	-3.948786172	0.000629631	0.046801665
cg21258370	-0.445955297	4.020413998	-3.948732926	0.000629714	0.046802959
cg01471491	-0.825034874	1.440519068	-3.948612973	0.000629903	0.046812029
cg23190353	-0.464576101	-0.579542767	-3.948468005	0.00063013	0.046819797
cg18123581	-0.792903195	0.943982588	-3.948349298	0.000630316	0.046819797
cg21388345	-0.498587578	1.082794945	-3.948320492	0.000630361	0.046819797
cg24911588	-0.480135229	3.825023525	-3.948232091	0.0006305	0.046819797
cg03779434	-0.55490125	3.570592662	-3.948149192	0.00063063	0.046819797
cg25797455	-0.354531482	-0.396045241	-3.948140624	0.000630644	0.046819797
cg27310031	-0.516978361	3.570525192	-3.948082704	0.000630735	0.046819797
cg13711143	-0.430398101	4.012191028	-3.948067014	0.000630759	0.046819797

cg25755875	-0.478115917	1.680233809	-3.948033928	0.000630811	0.046819797
cg12850049	0.460316472	2.845099888	3.948020561	0.000630832	0.046819797
cg00723563	-0.380245153	0.364982379	-3.948001607	0.000630862	0.046819797
cg01708543	-0.433205524	0.225826564	-3.947762881	0.000631237	0.046819797
cg13538320	-0.483273581	3.972255725	-3.947673157	0.000631378	0.046819797
cg03247301	-0.37361616	-0.359540122	-3.947664713	0.000631392	0.046819797
cg12927248	-0.432914282	-0.550973362	-3.947651527	0.000631412	0.046819797
cg18637843	-0.631644933	2.551941058	-3.947591695	0.000631506	0.046819797
cg06089892	-0.920177272	1.328989271	-3.947589629	0.00063151	0.046819797
cg23877385	-0.344460812	0.45846982	-3.947587816	0.000631513	0.046819797
cg07665134	-0.410636669	-0.079984368	-3.947542758	0.000631583	0.046819797
cg22430379	-0.537895624	3.11854821	-3.9475419	0.000631585	0.046819797
cg06437892	-0.416501503	0.572088551	-3.947517013	0.000631624	0.046819797
cg05668902	0.557754198	2.647663151	3.947491474	0.000631664	0.046819797
cg11284545	-0.415935299	-0.867164536	-3.94748606	0.000631673	0.046819797
cg03270175	-0.406697465	0.034617761	-3.94747462	0.000631691	0.046819797
cg18110553	-0.415297263	3.306097176	-3.947353273	0.000631881	0.046819797
cg21460047	-1.217897025	0.038272961	-3.947321796	0.000631931	0.046819797
cg09713815	-0.447352959	3.92910537	-3.947306965	0.000631954	0.046819797
cg11168621	-0.485311968	3.930491098	-3.947271321	0.00063201	0.046819797
cg16322006	-0.545123185	2.981169668	-3.947227161	0.00063208	0.046819797
cg25091766	-0.487983647	3.638504129	-3.947121495	0.000632246	0.046819797
cg17176472	-0.476850381	-0.405888862	-3.947119925	0.000632249	0.046819797
cg24141882	0.570153842	-1.921813017	3.947115826	0.000632255	0.046819797
cg00176043	-0.46906703	-0.132413164	-3.947086051	0.000632302	0.046819797
cg15307476	-0.465444499	3.789996591	-3.947003629	0.000632432	0.046819797
cg26486112	0.460169233	2.060631292	3.947002128	0.000632434	0.046819797
cg13958215	-0.720620632	3.316947107	-3.946999963	0.000632438	0.046819797
cg08343926	-0.443279295	-0.527429735	-3.946990458	0.000632453	0.046819797
cg13375696	-0.517518188	2.736811022	-3.94692208	0.00063256	0.046820559
cg12337562	-0.421511659	3.456616633	-3.946891583	0.000632608	0.046820559
cg03496850	-0.467865431	3.816505574	-3.946858028	0.000632661	0.046820559
cg25908655	-0.487439623	3.102595939	-3.94670368	0.000632904	0.046831722
cg12098787	-0.47337471	3.912928142	-3.946678381	0.000632944	0.046831722
cg23933554	-0.510950208	3.427625798	-3.946545428	0.000633154	0.046841354
cg18927521	-0.414333147	-1.460717463	-3.946493793	0.000633235	0.046841354
cg18669406	-0.376503207	0.072972464	-3.946345597	0.000633469	0.046841354
cg07261924	-0.798465242	2.268049442	-3.946295431	0.000633548	0.046841354
cg22761813	-0.511340913	2.8614162	-3.946219074	0.000633668	0.046841354
cg12111740	-0.635478737	2.535002161	-3.946166284	0.000633752	0.046841354
cg25224852	-4.057342278	-5.949261207	-3.946161675	0.000633759	0.046841354
cg10212055	-0.491321839	3.428896806	-3.946143169	0.000633788	0.046841354
cg17345703	-0.371423485	-0.525648558	-3.946075098	0.000633896	0.046841354
cg01148098	-0.841619383	0.061217615	-3.946023548	0.000633977	0.046841354
cg07155495	-0.579830662	3.238839837	-3.945950003	0.000634093	0.046841354
cg16189218	-0.591526329	3.246553358	-3.945932201	0.000634121	0.046841354
cg07134622	-0.531879349	2.056260001	-3.945851105	0.000634249	0.046841354

cg03335929	-0.696925902	2.618368625	-3.945825454	0.00063429	0.046841354
cg05764531	-0.460591069	3.877760118	-3.94582	0.000634298	0.046841354
cg11197318	-0.337417409	-0.203847891	-3.945693262	0.000634499	0.046841354
cg17579867	-0.784308426	3.000090104	-3.94568839	0.000634506	0.046841354
cg11758853	-0.437233704	4.117052967	-3.945629889	0.000634599	0.046841354
cg25132536	-0.761146111	-2.708326153	-3.945596477	0.000634652	0.046841354
cg15378328	0.587902271	3.503751248	3.945566057	0.0006347	0.046841354
cg26688079	-0.530077363	3.641910024	-3.945561662	0.000634707	0.046841354
cg24501330	0.589966926	2.571729911	3.945516167	0.000634778	0.046841354
cg06492821	-0.454024329	-1.672071666	-3.945493063	0.000634815	0.046841354
cg25665011	-0.541965259	3.562299104	-3.945442231	0.000634895	0.046841354
cg27226883	-0.575393543	3.023776381	-3.94541018	0.000634946	0.046841354
cg22949732	-0.400821205	-0.770630302	-3.945342454	0.000635053	0.046841354
cg13155823	-0.580526414	2.0390888	-3.945313608	0.000635099	0.046841354
cg07871717	-0.500358501	-0.155993037	-3.945289294	0.000635137	0.046841354
cg18515872	-0.367614975	-0.499567225	-3.945268958	0.000635169	0.046841354
cg08123673	0.390362073	2.989729053	3.945237059	0.00063522	0.046841354
cg18321827	-0.534300759	1.183565554	-3.945235304	0.000635223	0.046841354
cg22601108	-0.425597285	3.254867975	-3.945229218	0.000635232	0.046841354
cg22969666	-0.440802469	3.755707067	-3.945147268	0.000635362	0.046841354
cg25714134	-0.409074324	4.144578413	-3.94513383	0.000635383	0.046841354
cg23575320	-0.521906007	-1.995662777	-3.945088213	0.000635455	0.046841354
cg20071972	-0.804618413	3.655787001	-3.945024739	0.000635556	0.046841354
cg12659587	-0.495148284	2.78940941	-3.945016198	0.000635569	0.046841354
cg01627483	-0.469451866	-1.800177244	-3.945004846	0.000635587	0.046841354
cg12262507	-0.581370747	-0.682326505	-3.944910272	0.000635737	0.046847512
cg10699291	-0.394401322	3.120159972	-3.944785695	0.000635934	0.046847555
cg15518464	0.566479938	3.237566097	3.944755317	0.000635982	0.046847555
cg00540067	-0.549532806	-0.670380086	-3.944717468	0.000636042	0.046847555
cg06753153	-0.416325705	-0.535243704	-3.944611898	0.000636209	0.046847555
cg01750729	-0.441359514	0.330995497	-3.944609981	0.000636212	0.046847555
cg21161126	-0.54678532	0.562471601	-3.944555139	0.000636299	0.046847555
cg07810469	-0.771434568	1.648422594	-3.944515506	0.000636362	0.046847555
cg23967863	-0.476334197	4.262881706	-3.944495276	0.000636394	0.046847555
cg20019881	-0.414440022	0.108264483	-3.944492652	0.000636398	0.046847555
cg09152824	-0.760628918	-0.278356267	-3.944466382	0.00063644	0.046847555
cg17648096	-0.558815581	3.437454013	-3.944435864	0.000636488	0.046847555
cg00675001	-0.751345069	2.988863439	-3.944408841	0.000636531	0.046847555
cg06818764	-0.384769775	4.118373658	-3.944217905	0.000636834	0.046864962
cg09245683	-0.404112631	3.94420785	-3.944141581	0.000636955	0.046867659
cg07389750	-0.699430978	2.325408596	-3.944111351	0.000637003	0.046867659
cg08343347	-0.809532194	2.522079674	-3.944061727	0.000637081	0.046868582
cg04037841	-0.531756733	-0.83500126	-3.943891392	0.000637352	0.046869781
cg18743575	-0.520389392	3.481568215	-3.943888257	0.000637357	0.046869781
cg11416650	-0.47958971	0.713475827	-3.943879472	0.00063737	0.046869781
cg25204768	-0.496510971	4.001064771	-3.943845322	0.000637425	0.046869781
cg14208334	0.536440594	3.32135279	3.943802439	0.000637493	0.046869781

cg04071398	-0.432265448	4.204157692	-3.94380126	0.000637495	0.046869781
cg27417749	-0.530447835	0.015058726	-3.94365597	0.000637725	0.046881479
cg18537456	-0.936674308	0.195336188	-3.943617659	0.000637786	0.046881479
cg08537563	-0.530489627	2.878772978	-3.943546459	0.000637899	0.046883376
cg03107817	0.682922489	1.064456716	3.94350421	0.000637966	0.046883376
cg02241799	-0.387400847	-0.161413558	-3.943427869	0.000638088	0.046883376
cg05273780	-0.567047513	-0.969529913	-3.943340834	0.000638226	0.046883376
cg11146081	-0.404389818	3.454610152	-3.943301861	0.000638288	0.046883376
cg15130894	-0.519517839	0.289793383	-3.943284712	0.000638315	0.046883376
cg15466227	-0.601086127	3.573453333	-3.943235003	0.000638394	0.046883376
cg17162767	-0.595569535	2.81721048	-3.943222805	0.000638413	0.046883376
cg27468753	-0.423128168	-1.627042346	-3.943212328	0.00063843	0.046883376
cg03857931	-0.700587825	1.149680898	-3.943184844	0.000638474	0.046883376
cg26531879	-0.406304419	-0.334853553	-3.943102349	0.000638605	0.046888146
cg15844777	-0.356594576	-1.178921746	-3.943013198	0.000638747	0.046891008
cg06440678	-0.446998529	-0.929020431	-3.942994576	0.000638776	0.046891008
cg15131345	-0.445805631	3.387664497	-3.942815975	0.00063906	0.046906142
cg01167177	-0.433291594	4.042863534	-3.942766331	0.000639139	0.046906142
cg12248186	-0.456357084	3.544962423	-3.942671054	0.000639291	0.046906142
cg22626683	-0.45410533	2.889600608	-3.942654497	0.000639317	0.046906142
cg21919819	-0.485135828	1.03623109	-3.94263608	0.000639347	0.046906142
cg23321781	-0.37769222	4.079886959	-3.942615305	0.00063938	0.046906142
cg00060866	-0.400839397	-1.610817433	-3.942517107	0.000639536	0.046908572
cg21420536	-0.477227732	4.153592601	-3.942511311	0.000639545	0.046908572
cg23352491	-0.486582173	-0.019727407	-3.942437242	0.000639663	0.046912366
cg24993739	-0.446951569	3.849018816	-3.942273152	0.000639925	0.046922257
cg01696784	-0.728164584	-1.5904075	-3.942269403	0.000639931	0.046922257
cg04005840	-0.399593389	4.199073607	-3.942078497	0.000640235	0.046927428
cg06433834	-0.537414546	3.217782902	-3.942059563	0.000640265	0.046927428
cg00247803	-0.392122448	3.57391304	-3.942054897	0.000640272	0.046927428
cg11454270	-0.413822881	-0.687881128	-3.94205002	0.00064028	0.046927428
cg25399071	-0.486876698	3.137998071	-3.942017309	0.000640332	0.046927428
cg27109426	-0.443371596	0.010508347	-3.941894285	0.000640528	0.046936948
cg18770029	-0.594201488	0.970334418	-3.941767351	0.000640731	0.046944627
cg25872320	-0.56121665	-0.939185802	-3.941745479	0.000640766	0.046944627
cg02603284	-0.428939201	-0.34771566	-3.941581163	0.000641028	0.046954597
cg02631153	-0.569387992	3.338708242	-3.941502305	0.000641154	0.046954597
cg26662029	-0.536243251	3.403487287	-3.941494253	0.000641167	0.046954597
cg20004888	-0.89131989	-4.376618079	-3.941458972	0.000641223	0.046954597
cg05533210	-0.593900557	0.25586757	-3.941452529	0.000641233	0.046954597
cg18688077	-0.440324904	3.441566873	-3.941313119	0.000641456	0.046964763
cg16521460	-0.473151514	3.35917395	-3.941247983	0.00064156	0.046964763
cg10407055	-0.497464045	4.172572183	-3.941225501	0.000641596	0.046964763
cg11672854	-0.480518233	3.64115092	-3.941178936	0.00064167	0.046964763
cg18864437	-0.668085645	2.092051925	-3.941158017	0.000641704	0.046964763
cg14210811	-0.425726287	0.87502088	-3.941033511	0.000641903	0.04696696
cg01406862	-0.70993601	1.053467253	-3.941016919	0.000641929	0.04696696

cg25513853	-0.844646169	-2.953702445	-3.941014753	0.000641933	0.04696696
cg15211328	-0.678419675	2.977005748	-3.940941659	0.000642049	0.046970656
cg07127468	-0.822817935	2.233665495	-3.940771738	0.000642321	0.04697441
cg07182076	-0.447707322	0.205410904	-3.940734148	0.000642381	0.04697441
cg05684005	-0.644332226	2.675860859	-3.940702565	0.000642432	0.04697441
cg11427341	-0.458706416	-0.633660702	-3.940701541	0.000642433	0.04697441
cg19119365	-0.695263099	2.213481638	-3.940630292	0.000642547	0.04697441
cg18461278	-1.033374848	1.783022942	-3.940588052	0.000642615	0.04697441
cg17430032	-0.433899765	3.617507277	-3.940495693	0.000642763	0.04697441
cg18197376	-0.463358724	4.02246838	-3.94034479	0.000643004	0.04697441
cg00468670	-0.572165653	0.026860681	-3.940299021	0.000643077	0.04697441
cg13424484	-0.47379993	3.020545878	-3.940262606	0.000643136	0.04697441
cg04250878	-0.833158362	-0.403365449	-3.940139375	0.000643333	0.04697441
cg10028175	-0.504843054	-0.557546892	-3.940114804	0.000643372	0.04697441
cg04255090	-0.393749605	-0.770784785	-3.940109303	0.000643381	0.04697441
ch.20.12629947R	-0.510000613	-3.499831091	-3.940089691	0.000643413	0.04697441
cg07141215	-0.432548756	2.03316132	-3.940046124	0.000643482	0.04697441
cg16373144	-0.411684901	2.957584045	-3.939906333	0.000643706	0.04697441
cg13690169	-0.516929989	0.198278906	-3.939804463	0.00064387	0.04697441
cg20934713	-0.452767121	3.50159141	-3.939771746	0.000643922	0.04697441
cg03223456	-0.590318073	2.852649667	-3.939743801	0.000643967	0.04697441
cg25768367	-0.596204004	0.269762683	-3.939739591	0.000643974	0.04697441
cg12658792	-0.582154996	1.16341322	-3.93972736	0.000643993	0.04697441
cg01720935	-0.447499433	3.431649896	-3.939707528	0.000644025	0.04697441
cg24847709	0.462681976	3.276634264	3.939692982	0.000644048	0.04697441
cg12128262	-0.46315135	3.074446284	-3.939692846	0.000644049	0.04697441
cg24981837	-0.593114097	3.433119566	-3.939672715	0.000644081	0.04697441
cg04170325	-0.541687075	0.679127008	-3.939660879	0.0006441	0.04697441
cg07029411	0.729160496	2.60768014	3.939597458	0.000644202	0.04697441
cg01000642	-0.544168494	0.591077882	-3.939532815	0.000644305	0.04697441
cg15974460	-0.510920746	3.248987992	-3.939508718	0.000644344	0.04697441
cg27399763	-0.391818978	3.537072746	-3.939503659	0.000644352	0.04697441
cg02845018	0.457178933	1.913903719	3.939450348	0.000644437	0.04697441
cg25885914	-0.583345715	3.018508846	-3.939410859	0.000644501	0.04697441
cg10618992	-0.522133127	3.873708409	-3.939373064	0.000644561	0.04697441
cg01255782	-0.400040195	-0.962349936	-3.939348869	0.0006446	0.04697441
cg05740655	-0.640106645	3.446440411	-3.93933214	0.000644627	0.04697441
cg06130176	0.58286833	2.616904612	3.939275859	0.000644717	0.04697441
cg04867011	-0.881467848	1.289861517	-3.939275814	0.000644718	0.04697441
cg16250865	-0.543287274	-1.125610238	-3.939271308	0.000644725	0.04697441
cg23689579	-0.612361209	2.97165175	-3.939246362	0.000644765	0.04697441
cg06206229	-0.672109501	1.844585423	-3.939203951	0.000644833	0.04697441
cg25577099	-0.396122715	-0.576695196	-3.939198287	0.000644842	0.04697441
cg24104437	-0.47631404	-0.034865887	-3.939138329	0.000644938	0.04697441
cg26736446	-0.517701989	3.278005426	-3.939091786	0.000645013	0.04697441
cg12507814	0.49392576	3.269037066	3.939071373	0.000645046	0.04697441
cg18072384	-0.542137084	3.279782567	-3.939047106	0.000645085	0.04697441

cg03318340	-0.846202526	2.191935178	-3.93880314	0.000645477	0.046993476
cg27421037	-0.666955691	1.603233366	-3.938801518	0.000645479	0.046993476
cg19517051	-0.498823385	0.138443117	-3.938685005	0.000645666	0.046998156
cg00411569	-0.763862495	2.428996792	-3.938672251	0.000645687	0.046998156
cg03501807	-0.45775571	3.946075083	-3.938637678	0.000645743	0.046998156
cg14545620	-0.837261824	1.349287647	-3.938558844	0.000645869	0.047002551
cg06969118	-0.534150173	3.524443457	-3.938491452	0.000645978	0.047005608
cg16362271	-0.645838939	3.150214429	-3.938374288	0.000646166	0.047014491
cg04128804	-0.529749174	2.726697831	-3.938302634	0.000646281	0.047018048
cg19370065	-0.439924516	-0.538477687	-3.938069377	0.000646657	0.047037086
cg08763666	-0.571670767	2.820257204	-3.938057513	0.000646676	0.047037086
cg05669151	-0.432703329	0.241852549	-3.937959718	0.000646833	0.047040374
cg21666801	-0.49533427	3.747247116	-3.937946946	0.000646854	0.047040374
cg09581644	-0.420681621	4.276765795	-3.937856683	0.000646999	0.047041285
cg24438974	-0.610962374	2.870536237	-3.937830926	0.000647041	0.047041285
cg10732087	-0.330323817	-0.071825675	-3.937815465	0.000647066	0.047041285
cg23882545	0.81266527	-0.02465671	3.937696687	0.000647257	0.047045625
cg01114124	-0.417131093	-0.4525722	-3.937695965	0.000647258	0.047045625
cg20562586	-0.416327515	3.740544661	-3.93747827	0.000647609	0.047066297
cg23007368	-0.365785964	-0.259966551	-3.937314323	0.000647873	0.047080679
cg17797848	-0.604512619	0.097051227	-3.937239491	0.000647994	0.047080889
cg07877113	-0.523560088	2.978574527	-3.937182595	0.000648086	0.047080889
cg07993134	-0.420797538	0.566491252	-3.937168327	0.000648109	0.047080889
cg03111727	-0.451106007	3.811922274	-3.937071478	0.000648265	0.047080889
cg27279920	-0.363992472	-0.434751914	-3.937071325	0.000648265	0.047080889
cg14123840	-0.753337799	2.411438705	-3.937048516	0.000648302	0.047080889
cg01393579	-0.507005552	2.168875095	-3.937024169	0.000648341	0.047080889
cg19516263	-0.605079023	2.557917805	-3.936931825	0.000648491	0.047086885
cg12306949	-0.523589369	3.080645718	-3.936807427	0.000648691	0.047096641
cg07629517	-0.546555055	3.367838389	-3.936657459	0.000648934	0.047098677
cg17628023	-0.456125983	3.39016033	-3.936648986	0.000648947	0.047098677
cg19998423	-0.530094458	-0.615914438	-3.936627402	0.000648982	0.047098677
cg25146976	-0.527846533	-3.304609971	-3.936613658	0.000649004	0.047098677
cg07925606	-0.402007341	3.32009764	-3.936508415	0.000649174	0.047098677
cg13794390	-0.673712523	1.945598999	-3.93650565	0.000649179	0.047098677
cg27093379	-0.482980758	0.600927016	-3.93644802	0.000649272	0.047098677
cg08546075	-0.555428287	2.770439596	-3.936434472	0.000649294	0.047098677
cg10211209	-0.565561898	3.244773431	-3.936419675	0.000649318	0.047098677
cg19414302	-0.368582327	-0.067134067	-3.936283005	0.000649539	0.047109879
cg05183668	-0.551349641	-0.33685987	-3.936135117	0.000649778	0.047117501
cg05598481	0.653221766	3.340452632	3.936112785	0.000649814	0.047117501
cg12481734	-1.28250275	-5.811149648	-3.936017192	0.000649969	0.047117501
cg22992794	-0.816912955	0.515229325	-3.935966778	0.00065005	0.047117501
cg06721868	-0.587229675	2.677611123	-3.935944248	0.000650087	0.047117501
cg11434762	-0.596420141	3.396983043	-3.935937538	0.000650098	0.047117501
cg25699273	-0.569456341	2.697327965	-3.935930203	0.000650109	0.047117501
cg12980830	-0.473658505	3.488859714	-3.935805088	0.000650312	0.047119427

cg01024504	-0.454863165	-0.986318907	-3.935758492	0.000650387	0.047119427
cg05088463	-0.608898041	-1.731583486	-3.935722296	0.000650446	0.047119427
cg14094884	-0.573758299	2.683948969	-3.935688591	0.000650501	0.047119427
cg25024515	-0.590139705	1.237438565	-3.935680843	0.000650513	0.047119427
cg26398907	-0.5813165	4.109651739	-3.93563081	0.000650594	0.047119427
cg18773150	-0.497261247	-0.757707262	-3.935626171	0.000650602	0.047119427
cg05925857	-0.477480724	3.675162083	-3.93540916	0.000650953	0.047140067
cg08411567	-0.425668358	2.768755357	-3.935302568	0.000651126	0.047145141
cg23344412	-0.453897949	3.61496429	-3.935283786	0.000651156	0.047145141
cg07107053	-0.555902306	2.850181029	-3.935086371	0.000651476	0.047157567
cg09856470	-0.461089671	3.649070133	-3.9350309	0.000651566	0.047157567
cg20618663	-0.497405265	1.641941483	-3.935014718	0.000651593	0.047157567
cg07647993	-0.447850952	3.5117612	-3.935013709	0.000651594	0.047157567
cg07795228	-0.450732545	-0.44968513	-3.934913854	0.000651756	0.047164471
cg26380316	0.391502532	2.89829273	3.934864261	0.000651837	0.047165475
cg08135718	-0.532396852	-0.159582243	-3.93480039	0.00065194	0.047166073
cg15075580	-0.357248448	3.7320856	-3.934743522	0.000652033	0.047166073
cg15417798	-0.477091655	-1.596496046	-3.934736077	0.000652045	0.047166073
cg22268255	0.601607515	2.625351021	3.934655472	0.000652176	0.047170721
cg16435782	-0.496146649	0.44736029	-3.934534359	0.000652372	0.047176106
cg10391294	-0.608630487	3.393525821	-3.934502224	0.000652424	0.047176106
cg14124869	-0.384160176	4.190808479	-3.934447805	0.000652513	0.047176106
cg25277053	-0.656685627	2.968454606	-3.934343251	0.000652683	0.047176106
cg05951419	-0.48366778	3.732837396	-3.934338802	0.00065269	0.047176106
cg04222494	-0.538191043	3.905808812	-3.934324155	0.000652714	0.047176106
cg19746825	-0.810489132	2.43694825	-3.934322577	0.000652716	0.047176106
cg25528143	0.58158912	-4.067208241	3.934278158	0.000652788	0.047176509
cg24114587	-0.694397253	3.14482437	-3.934223565	0.000652877	0.047176586
cg17599688	-0.500444294	3.11845656	-3.934195538	0.000652923	0.047176586
cg06242416	-0.466093776	2.197565905	-3.934068246	0.000653129	0.047186411
cg19375539	-0.638235773	-2.036387352	-3.934029923	0.000653192	0.047186411
cg02099988	-0.507307625	-0.7778823	-3.933897862	0.000653407	0.047197112
cg12760659	-0.849787174	1.225674312	-3.933726699	0.000653685	0.047200738
cg24954325	-0.491924508	-1.111382833	-3.933636107	0.000653832	0.047200738
cg21056326	-0.808210239	2.42034676	-3.933620119	0.000653858	0.047200738
cg02119693	-0.838884966	1.397150649	-3.933508665	0.00065404	0.047200738
cg05530162	-0.470651688	3.834112269	-3.933366364	0.000654272	0.047200738
cg19853104	-0.449945697	4.07536657	-3.933323173	0.000654342	0.047200738
cg16865793	-0.429252261	-0.249962941	-3.933299514	0.00065438	0.047200738
cg27336073	-0.628800306	-0.74550187	-3.933295516	0.000654387	0.047200738
cg00620746	-0.396608244	4.150474882	-3.933287444	0.0006544	0.047200738
cg24622439	-0.369006107	4.274311895	-3.933184095	0.000654568	0.047200738
cg23894205	-0.529580123	3.966390171	-3.933170167	0.000654591	0.047200738
cg21606102	-0.39663685	-0.383797237	-3.933149428	0.000654625	0.047200738
cg18483750	-0.465490547	3.695552517	-3.933138852	0.000654642	0.047200738
cg11483215	-0.714371834	2.933604238	-3.933134314	0.00065465	0.047200738
cg07317805	-0.47389495	3.553703739	-3.933076286	0.000654744	0.047200738

cg00016814	-0.679093903	1.800608063	-3.93307511	0.000654746	0.047200738
cg00997931	-0.595172212	2.895508171	-3.933067705	0.000654758	0.047200738
cg01571977	-0.421859548	0.426086861	-3.933030015	0.000654819	0.047200738
cg06148656	-0.631185365	1.599478707	-3.932991586	0.000654882	0.047200738
cg06694561	-0.433135071	0.304796325	-3.93297292	0.000654913	0.047200738
cg21707740	-0.69303397	3.057605803	-3.932904807	0.000655024	0.047200738
cg07706880	-0.395739028	4.06549882	-3.932898643	0.000655034	0.047200738
cg10550074	-0.542736784	2.799027005	-3.932824071	0.000655155	0.047200738
cg00179196	-0.389800301	0.952661546	-3.932801504	0.000655192	0.047200738
cg11090342	-0.57729093	3.39584158	-3.932785473	0.000655218	0.047200738
cg16124718	-0.432038753	-1.278053537	-3.932778307	0.00065523	0.047200738
cg16240260	-0.698306529	2.358617989	-3.932762415	0.000655256	0.047200738
cg06429884	-0.362972789	4.422574788	-3.932612519	0.0006555	0.04721355
cg14735704	-0.641412811	3.566632211	-3.932536466	0.000655624	0.047217688
cg12428447	-0.442778767	-0.258713684	-3.932405513	0.000655838	0.047225859
cg06878117	-0.477801254	3.169537731	-3.93235704	0.000655917	0.047225859
cg12382418	-0.429367122	3.46413741	-3.932337526	0.000655949	0.047225859
cg21208943	0.436491875	1.366166214	3.932303585	0.000656005	0.047225859
cg16950503	0.735364952	3.575302863	3.932189596	0.000656191	0.04723446
cg05460965	-0.380364702	-0.774942014	-3.932003983	0.000656494	0.047249567
cg19403610	-0.543636027	-0.062760367	-3.931972801	0.000656545	0.047249567
cg21108691	-0.545797323	3.888670031	-3.931938657	0.000656601	0.047249567
cg21114429	-0.781305194	1.075060783	-3.931864964	0.000656721	0.047253434
cg04318607	-0.401779002	-1.82784833	-3.931797094	0.000656832	0.047256617
cg26855961	-0.307460751	-0.465448344	-3.931650267	0.000657072	0.047266374
cg25833403	-0.543856807	3.651691273	-3.931617693	0.000657125	0.047266374
cg07777524	-0.55957897	-0.176007022	-3.931591748	0.000657168	0.047266374
cg26907167	-0.466432903	-0.101978566	-3.931335904	0.000657586	0.047290632
cg07741592	-0.377623377	-0.959620466	-3.931304016	0.000657639	0.047290632
cg25496104	-0.507447645	3.194381172	-3.931201702	0.000657806	0.047297875
cg26250120	-0.814640514	2.548473009	-3.931104456	0.000657965	0.047304523
cg26102434	-0.568509094	3.01398521	-3.931024382	0.000658096	0.04730915
cg20597640	-0.652796473	1.575055401	-3.930982296	0.000658165	0.047309305
cg23015434	-0.423132847	-0.762376978	-3.93081666	0.000658437	0.047321255
cg01295999	-0.541009123	0.537426324	-3.93079929	0.000658465	0.047321255
cg24928451	-0.602961812	3.655593715	-3.930499095	0.000658957	0.047348478
cg19884600	-0.387078388	3.57655287	-3.9304867	0.000658978	0.047348478
cg00423403	-0.456222433	4.014899075	-3.930377013	0.000659158	0.047354126
cg22559350	-0.929221384	-2.783945493	-3.93031976	0.000659252	0.047354126
cg23572703	-0.580606305	-1.030040704	-3.930290097	0.0006593	0.047354126
cg01839003	-0.432868098	-0.163038992	-3.930275792	0.000659324	0.047354126
cg10912830	-0.384867765	4.373991645	-3.930132915	0.000659558	0.047354237
cg08869748	-0.474575791	3.113426732	-3.930118932	0.000659581	0.047354237
cg02998321	-0.51761426	3.566582168	-3.930081948	0.000659642	0.047354237
cg18192419	-0.513312164	3.899138941	-3.930079779	0.000659645	0.047354237
cg04850418	0.540443634	2.083701161	3.930071215	0.00065966	0.047354237
cg17506742	-0.440054176	-1.962941683	-3.929981754	0.000659806	0.047359982

cg18993534	0.643082858	2.590502062	3.929890336	0.000659957	0.047361739
cg09974329	-0.444189198	2.31103602	-3.929869079	0.000659991	0.047361739
cg13652386	-0.484815191	3.297109506	-3.92983113	0.000660054	0.047361739
cg26422861	-0.465009117	-2.04289553	-3.929797718	0.000660109	0.047361739
cg11594887	-0.595106107	0.689751509	-3.929707701	0.000660257	0.047361739
cg20162626	-0.540830534	2.832841845	-3.92970184	0.000660266	0.047361739
cg20887544	-0.547066995	3.570069664	-3.92967145	0.000660316	0.047361739
cg26683252	-0.531880004	-0.271664772	-3.92960372	0.000660428	0.047361739
cg03742932	-0.6684668	2.742942298	-3.929562327	0.000660496	0.047361739
cg16646383	-0.452917689	-0.14747297	-3.929550409	0.000660515	0.047361739
cg24486037	-0.70262791	-0.760156338	-3.929519252	0.000660566	0.047361739
cg11363513	-0.569079571	3.743540403	-3.929474942	0.000660639	0.047362169
cg05354105	-0.499253903	3.57434518	-3.929390921	0.000660777	0.047362923
cg13570658	-0.497943982	3.664542286	-3.929369948	0.000660812	0.047362923
cg20606489	-0.43491681	3.720307344	-3.929346568	0.00066085	0.047362923
cg26684238	-0.546980557	3.614537193	-3.929290289	0.000660943	0.047364765
cg06043710	-0.665736915	-0.874930568	-3.929197158	0.000661096	0.047370953
cg17545805	-0.439033028	0.429968925	-3.929098713	0.000661258	0.047375143
cg16435542	-0.616058164	1.027442359	-3.929080017	0.000661289	0.047375143
cg03934478	-0.414959412	0.816002363	-3.929039706	0.000661355	0.047375143
cg12440131	-0.392333452	4.198243975	-3.928990518	0.000661436	0.047376152
cg19811420	-0.732952375	2.296033579	-3.928841832	0.000661681	0.047379346
cg05852845	-0.554535768	1.974375632	-3.928826327	0.000661707	0.047379346
cg19191555	-0.572419926	3.076729673	-3.928794488	0.000661759	0.047379346
cg22914389	0.553970753	-4.846606792	3.928766892	0.000661804	0.047379346
cg03714235	-0.856809187	0.283251787	-3.928740135	0.000661848	0.047379346
cg10993701	-0.807918679	0.835582075	-3.928713305	0.000661893	0.047379346
cg21332135	-0.559827582	3.59400164	-3.928679135	0.000661949	0.047379346
cg27097541	-0.478204819	4.094206769	-3.92849911	0.000662246	0.04739579
cg07040325	-0.53848553	1.61003026	-3.928358741	0.000662477	0.04740756
cg06653974	-0.472698795	3.100943544	-3.928317036	0.000662546	0.047407692
cg14287022	0.53855034	3.267352237	3.928205812	0.000662729	0.047414319
cg15514896	-0.548342872	-1.168100306	-3.928179706	0.000662772	0.047414319
cg17464848	-0.628788683	3.048169727	-3.928097444	0.000662908	0.047419238
cg14675963	0.514630195	2.949588921	3.928026131	0.000663026	0.047420762
cg26554194	0.509600024	2.163777901	3.928003386	0.000663063	0.047420762
cg20409748	-0.588124971	2.669191819	-3.927869183	0.000663285	0.047427119
cg08382193	-0.554526576	-2.33032396	-3.927833456	0.000663344	0.047427119
cg15302530	-0.434726545	-2.946927587	-3.927827843	0.000663353	0.047427119
cg05962169	-0.508078526	3.564622215	-3.927765995	0.000663455	0.047429249
cg02423014	-0.450055001	-0.480484318	-3.927708829	0.000663549	0.047429249
cg06543886	-0.344156111	4.285878474	-3.927688151	0.000663584	0.047429249
cg04753163	0.53349833	0.813277721	3.927545966	0.000663818	0.047440303
cg20659236	-0.463159852	0.186026292	-3.927513427	0.000663872	0.047440303
cg06501109	-0.62411487	-1.486217602	-3.927360707	0.000664124	0.047447474
cg16365421	-0.644019216	-0.996265531	-3.927332	0.000664172	0.047447474
cg21679611	-0.572194538	-1.209889868	-3.927331106	0.000664173	0.047447474

cg02376205	0.560768784	3.130045698	3.927248389	0.00066431	0.04745094
cg16140372	-0.486367593	0.134491981	-3.927163664	0.00066445	0.04745094
cg14107822	-0.449776177	3.092238892	-3.927109619	0.00066454	0.04745094
cg13575698	-0.589424108	2.482699289	-3.92705353	0.000664632	0.04745094
cg05351880	-0.500604258	3.340065715	-3.927037243	0.000664659	0.04745094
cg01003164	-0.492114775	-0.616940109	-3.927016046	0.000664694	0.04745094
cg12210255	-0.354402335	-2.128288239	-3.927003336	0.000664715	0.04745094
cg17567690	-0.593235318	3.169977922	-3.926977708	0.000664758	0.04745094
cg08625747	-0.462837003	3.911688078	-3.926859138	0.000664954	0.047452973
cg12651286	-0.59616722	-0.417017333	-3.92685684	0.000664958	0.047452973
cg03832839	-0.663385752	-2.715739761	-3.926833928	0.000664996	0.047452973
cg20574112	-0.43438334	3.756994252	-3.926798556	0.000665054	0.047452973
cg14276379	-0.660363931	-1.931868548	-3.926735863	0.000665158	0.047455597
cg04208160	-0.652446864	1.586093181	-3.926682154	0.000665247	0.047457161
cg20280631	-0.522504173	3.789151563	-3.926587627	0.000665403	0.047462313
cg16544366	-0.508470519	2.255562339	-3.926533075	0.000665494	0.047462313
cg05277865	-0.465407628	4.382930864	-3.926517157	0.00066552	0.047462313
cg23498445	-1.132780549	-1.865646218	-3.92645748	0.000665619	0.047464583
cg24452303	-0.660252035	-4.005818203	-3.926204436	0.000666038	0.047487296
cg13075444	0.458505403	1.626860285	3.926184345	0.000666072	0.047487296
cg12990093	-0.480754434	3.50354063	-3.926033185	0.000666322	0.047500382
cg07638027	-0.776929688	1.667553746	-3.925869762	0.000666593	0.047503768
cg03449148	-0.684455754	3.130945029	-3.925810903	0.000666691	0.047503768
cg07266249	-0.642610218	3.696898999	-3.925772638	0.000666754	0.047503768
cg08235081	-0.37996329	-2.297815814	-3.925713163	0.000666853	0.047503768
cg09607268	-0.386373397	4.040711263	-3.925661545	0.000666939	0.047503768
cg05807381	-0.534800102	3.758545726	-3.92564104	0.000666973	0.047503768
cg19685106	-0.548215888	2.94142561	-3.925612682	0.00066702	0.047503768
cg22567444	-0.478446535	-0.537032886	-3.925598029	0.000667044	0.047503768
cg11249861	-0.470075724	3.501372374	-3.925579372	0.000667075	0.047503768
cg06304812	-0.449982478	3.670576052	-3.925573057	0.000667086	0.047503768
cg03058578	-0.51286128	3.764444726	-3.925533095	0.000667152	0.047503768
cg19578030	-0.436841186	3.853562189	-3.925403287	0.000667367	0.047503768
cg25437822	-0.587825113	2.343245292	-3.925386146	0.000667396	0.047503768
cg04246253	-0.488172702	2.814679053	-3.925377536	0.00066741	0.047503768
cg26778733	-0.523758962	0.364974786	-3.925323238	0.0006675	0.047503768
cg17769705	-0.422832551	4.138695842	-3.925276781	0.000667578	0.047503768
cg09513224	0.516795673	2.852606652	3.925273326	0.000667583	0.047503768
cg08130444	-0.463165046	3.130804136	-3.925256935	0.000667611	0.047503768
cg11157965	0.669811689	2.680377647	3.925200492	0.000667704	0.047503768
cg00325023	-0.41043644	-0.394058539	-3.925112594	0.00066785	0.047503768
cg02457541	-0.43268647	0.150891476	-3.925064126	0.000667931	0.047503768
cg14117934	-0.422562128	3.287308598	-3.925061662	0.000667935	0.047503768
cg07957619	-0.413171931	-2.091716031	-3.925056956	0.000667943	0.047503768
cg02728979	0.524801556	2.420350274	3.925035172	0.000667979	0.047503768
cg09723881	-0.504110259	3.798327763	-3.924984715	0.000668063	0.047504964
cg20204776	-0.463242715	3.501780764	-3.924868937	0.000668255	0.047508077

cg25522119	-0.707045038	-2.194973065	-3.924826223	0.000668327	0.047508077
cg06137502	-0.360961244	-0.037337694	-3.924799434	0.000668371	0.047508077
cg23186952	-0.455162955	0.457798146	-3.92479706	0.000668375	0.047508077
cg01101448	-0.485073611	2.569426522	-3.92470774	0.000668524	0.047513871
cg09140693	-0.863031759	2.331244952	-3.924660916	0.000668601	0.04751464
cg05700616	-0.538229595	-0.186185139	-3.924583268	0.000668731	0.047518717
cg25961702	-0.456365553	4.088259729	-3.924545812	0.000668793	0.047518717
cg17805210	-0.518257374	3.778252036	-3.924314333	0.000669178	0.047538712
cg17770034	-0.548521897	2.233986501	-3.924265617	0.00066926	0.047538712
cg10772906	-0.487685152	3.558948893	-3.924255861	0.000669276	0.047538712
cg25859670	-0.589810516	4.287272506	-3.924183813	0.000669396	0.047542468
cg22449691	-0.499483182	3.723059289	-3.924000904	0.000669701	0.047557153
cg05753823	-0.631215152	2.729679103	-3.923951712	0.000669782	0.047557153
cg24757467	-0.536535053	3.97109787	-3.923882657	0.000669898	0.047557153
cg08358418	-0.553116086	3.482801269	-3.923869024	0.00066992	0.047557153
cg07542096	0.475732872	2.351185898	3.92380197	0.000670032	0.047557153
cg18885992	-0.513447305	-1.672134506	-3.923754285	0.000670112	0.047557153
cg21218089	-0.379455896	2.046114066	-3.923715642	0.000670176	0.047557153
cg07207367	-0.373316703	-0.852088553	-3.923689796	0.000670219	0.047557153
cg05049790	-0.54836172	3.039228859	-3.923668938	0.000670254	0.047557153
cg13585927	-0.385151576	-0.267339066	-3.923619883	0.000670336	0.047557153
cg13305336	-0.887966409	-0.541210371	-3.923584711	0.000670394	0.047557153
cg15181688	0.436494721	1.126575796	3.923576507	0.000670408	0.047557153
cg06815731	-0.880870654	1.953389295	-3.923516476	0.000670508	0.047558314
cg13232468	-0.431660864	-1.402414147	-3.923439782	0.000670636	0.047558314
cg25458367	-0.69124006	2.002616201	-3.923315523	0.000670844	0.047558314
cg06038180	-0.438742466	0.574417889	-3.923265505	0.000670927	0.047558314
cg13847853	0.41033294	2.370193195	3.92326536	0.000670927	0.047558314
cg16606320	-0.587529424	-1.970211256	-3.923198774	0.000671039	0.047558314
cg16069600	-0.446663662	3.5923387	-3.923159348	0.000671104	0.047558314
cg06376392	-0.629454041	-1.416634534	-3.923158411	0.000671106	0.047558314
cg14743071	-0.749937581	2.740078358	-3.92308002	0.000671237	0.047558314
cg11697861	-0.332365356	2.612625872	-3.92302289	0.000671332	0.047558314
cg13513428	-0.700213674	1.65317609	-3.9230184	0.00067134	0.047558314
cg06736079	-0.472391062	4.297335804	-3.922920609	0.000671503	0.047558314
cg04193083	-0.677067427	-3.386617968	-3.922855473	0.000671612	0.047558314
cg09705456	-0.464700528	1.10975046	-3.922840664	0.000671637	0.047558314
cg02180798	-0.506745068	-1.277763842	-3.922840534	0.000671637	0.047558314
cg23274247	-0.644990173	-3.112791544	-3.922839519	0.000671639	0.047558314
cg09799789	-0.541002954	3.185781368	-3.922834081	0.000671648	0.047558314
cg04947949	-0.655594003	3.23681573	-3.922815762	0.000671678	0.047558314
cg10421214	-0.39381084	4.068343513	-3.922659096	0.00067194	0.047558314
cg02164185	-0.420541619	3.65826556	-3.922578281	0.000672075	0.047558314
cg02094285	-0.617812587	3.735457623	-3.922515493	0.00067218	0.047558314
cg03548062	-1.026567646	4.321822768	-3.92246032	0.000672273	0.047558314
cg20409397	-0.702635507	3.277104778	-3.922413834	0.000672351	0.047558314
cg00194869	-0.642787271	1.516096582	-3.922412643	0.000672353	0.047558314

cg27314569	-0.459674621	-1.796187913	-3.922405112	0.000672365	0.047558314
cg12891971	-0.451544027	3.889177792	-3.922351414	0.000672455	0.047558314
cg15428351	-0.574055511	3.562027996	-3.92233734	0.000672479	0.047558314
cg26284114	-0.736705446	2.429975871	-3.922251458	0.000672622	0.047558314
cg20098883	-0.792115159	2.319280534	-3.922204473	0.000672701	0.047558314
cg26543333	-0.549482215	-1.095537373	-3.922170958	0.000672757	0.047558314
cg17489939	0.542286182	0.650786357	3.922151442	0.00067279	0.047558314
cg03429512	-0.486761658	2.906253823	-3.922095495	0.000672883	0.047558314
cg12511689	-0.503133815	0.360852674	-3.9220748	0.000672918	0.047558314
cg17563034	-0.511353747	-1.866309066	-3.922063486	0.000672937	0.047558314
cg01121564	-0.49234723	4.38718614	-3.92202674	0.000672998	0.047558314
cg00217795	-0.393182772	-0.643476335	-3.921994718	0.000673052	0.047558314
cg02382517	-0.516850354	3.031655437	-3.921953387	0.000673121	0.047558314
cg22336293	-0.474939449	0.490682225	-3.921948886	0.000673129	0.047558314
cg15848380	-0.876931577	2.474371242	-3.921946952	0.000673132	0.047558314
cg18360347	0.649228861	2.599933736	3.921923728	0.000673171	0.047558314
cg23670387	-0.84660694	1.306407148	-3.921794887	0.000673387	0.047558314
cg20618826	-0.495928365	-0.787847294	-3.921786355	0.000673401	0.047558314
cg09365758	-0.683303484	1.63734466	-3.921698293	0.000673549	0.047558314
cg01952742	-0.464973355	3.609367	-3.921656339	0.000673619	0.047558314
cg22529541	-0.397245476	3.456605603	-3.921641752	0.000673643	0.047558314
cg19040427	-0.554048353	3.002068574	-3.921547138	0.000673802	0.047558314
cg04556412	-0.459878321	-0.678827364	-3.921517754	0.000673851	0.047558314
cg04546344	-0.502593532	3.082905725	-3.921498606	0.000673883	0.047558314
cg17047517	-0.479634392	3.372475625	-3.921441612	0.000673979	0.047558314
cg00892294	-0.61465257	0.168272656	-3.921432085	0.000673995	0.047558314
cg18468235	-0.86169702	-1.859561094	-3.921389429	0.000674067	0.047558314
cg05341689	-0.663181845	-0.837886127	-3.921361148	0.000674114	0.047558314
cg02017189	-0.560854132	3.010442575	-3.921347182	0.000674137	0.047558314
cg05162523	-0.374387348	-0.539746981	-3.921307662	0.000674204	0.047558314
cg06805064	-0.932411992	0.259409072	-3.921292217	0.00067423	0.047558314
cg08981757	-0.521877112	3.115172551	-3.921287875	0.000674237	0.047558314
cg20510600	-0.419459879	1.10498012	-3.921279302	0.000674251	0.047558314
cg17010737	-0.526033499	2.358217735	-3.921182753	0.000674413	0.04756285
cg01639509	-0.674382353	3.003635157	-3.921119219	0.00067452	0.04756285
cg20890180	-0.400180822	4.304673896	-3.92105204	0.000674633	0.04756285
cg24070942	-0.478481523	2.008092231	-3.921001235	0.000674718	0.04756285
cg11648522	-0.396797025	-1.517038286	-3.920975592	0.000674761	0.04756285
cg17159459	-0.535901344	-0.262686111	-3.920959521	0.000674788	0.04756285
cg20490064	-0.411041296	1.136222671	-3.920880315	0.000674921	0.04756285
cg06014673	-0.5070498	3.970106441	-3.920729439	0.000675174	0.04756285
cg16211438	-0.5185048	3.926685811	-3.920593246	0.000675403	0.04756285
cg14076729	-0.373400179	-1.017231698	-3.920553489	0.00067547	0.04756285
cg01786360	-0.528610679	2.704921055	-3.920522194	0.000675523	0.04756285
cg26397166	-0.368567198	4.066088378	-3.920505279	0.000675551	0.04756285
cg23246499	-0.70160858	2.621620776	-3.920496116	0.000675566	0.04756285
cg14105637	0.598985585	2.90469883	3.920451433	0.000675642	0.04756285

cg25106671	-0.410458155	-2.693279656	-3.920417094	0.000675699	0.04756285
cg26375587	-0.405440642	3.62698128	-3.92039953	0.000675729	0.04756285
cg19925256	-0.559256081	-0.431723613	-3.920373782	0.000675772	0.04756285
cg18710798	-0.678318344	2.120390103	-3.920332331	0.000675842	0.04756285
cg14849575	-0.489438285	2.909615832	-3.920322373	0.000675859	0.04756285
cg15126137	-0.470810636	3.699736754	-3.920244674	0.000675989	0.04756285
cg12923203	-0.554914734	3.844939463	-3.920221229	0.000676029	0.04756285
cg12253142	-0.617337056	2.154526427	-3.920215524	0.000676038	0.04756285
cg14105271	-0.646585879	-1.837239265	-3.9201756	0.000676105	0.04756285
cg03435439	-0.603358718	-1.239055734	-3.92012969	0.000676183	0.04756285
cg01468523	-0.438783813	-0.026365295	-3.920098999	0.000676234	0.04756285
cg26752870	-0.576695578	0.097214865	-3.92009369	0.000676243	0.04756285
cg17440408	-0.385091969	-0.265124835	-3.920055506	0.000676308	0.04756285
cg03157605	-0.852665826	-4.572800605	-3.91999867	0.000676403	0.04756285
cg04492567	-0.378462929	-1.97118665	-3.919976805	0.00067644	0.04756285
cg10083956	-0.57124863	4.16842247	-3.919971622	0.000676449	0.04756285
cg03609808	-0.405832668	4.16286133	-3.9199467	0.000676491	0.04756285
cg18914866	-0.464319805	3.388869544	-3.919916383	0.000676542	0.04756285
cg23570146	-0.30733555	4.1264416	-3.919910682	0.000676551	0.04756285
cg01496475	-0.432999186	3.408246349	-3.91988266	0.000676598	0.04756285
cg21452088	-0.738429676	2.079434343	-3.919813822	0.000676714	0.047566277
cg25883013	-0.623924147	2.019352806	-3.919600362	0.000677074	0.047582308
cg15124946	-0.409003597	-0.28879679	-3.919538558	0.000677178	0.047582308
cg24769583	0.54759826	3.745958981	3.919527313	0.000677197	0.047582308
cg00296804	-0.439106314	-0.350633833	-3.919518911	0.000677211	0.047582308
cg12624025	-0.368932521	3.34701464	-3.919466608	0.000677299	0.04758378
cg23351707	-0.433717161	3.364593582	-3.919284657	0.000677606	0.047600606
cg06595154	-0.673651055	1.735201069	-3.919075762	0.000677958	0.047601134
cg10642200	-0.436577106	3.473252072	-3.919073161	0.000677963	0.047601134
cg15022173	-0.395883818	-1.309453452	-3.919043771	0.000678012	0.047601134
cg09431505	-0.701376072	1.925176675	-3.919013701	0.000678063	0.047601134
cg26603919	-0.50605493	2.972061334	-3.918979451	0.000678121	0.047601134
cg00935068	-0.361639259	0.413508886	-3.918918017	0.000678224	0.047601134
cg13463538	-0.4480268	3.708948217	-3.918802257	0.00067842	0.047601134
cg04055835	-0.42804476	-0.393272633	-3.918771	0.000678472	0.047601134
cg08613497	-0.636351716	3.113060782	-3.918725647	0.000678549	0.047601134
cg06446577	-0.488705581	0.628805823	-3.918713821	0.000678569	0.047601134
cg24608458	-0.683341224	3.187105459	-3.918676169	0.000678633	0.047601134
cg20004324	-0.49023123	3.594081509	-3.918628697	0.000678713	0.047601134
cg06142324	-0.489575607	-0.346420529	-3.918617662	0.000678731	0.047601134
cg02651217	-0.707174919	1.766234223	-3.918539763	0.000678863	0.047601134
cg08611402	-0.976268973	0.390804707	-3.918361908	0.000679163	0.047601134
cg14854431	0.55641153	1.778588734	3.918360717	0.000679165	0.047601134
cg19875864	-0.63990879	3.316537815	-3.91818755	0.000679458	0.047601134
cg06517521	-0.498064531	3.62013329	-3.918170855	0.000679486	0.047601134
cg01249522	-0.911402095	3.431912205	-3.918154512	0.000679514	0.047601134
cg18100085	-0.538037376	3.16199877	-3.918132065	0.000679552	0.047601134

cg25704289	-0.727561672	2.072087001	-3.91811864	0.000679575	0.047601134
cg06733776	-0.621373065	3.052378245	-3.918096108	0.000679613	0.047601134
cg20738928	-0.472516229	3.288769687	-3.918028729	0.000679727	0.047601134
cg00189927	-0.416321249	3.704324793	-3.918015459	0.000679749	0.047601134
cg00234645	-0.756251154	1.886850897	-3.918012827	0.000679754	0.047601134
cg20827317	-0.394202418	4.104089529	-3.918006956	0.000679763	0.047601134
cg23000806	-0.484043084	0.41452724	-3.917972982	0.000679821	0.047601134
cg11558117	-0.720808387	3.027953339	-3.91796532	0.000679834	0.047601134
cg24812398	-0.692316777	-2.65368962	-3.917924488	0.000679903	0.047601134
cg15790271	-0.500410677	0.549607446	-3.917875433	0.000679986	0.047601134
cg04764612	-0.482164891	4.021850576	-3.917817045	0.000680085	0.047601134
cg13404478	-0.546474476	3.665480374	-3.91781691	0.000680085	0.047601134
cg16947559	-0.465880486	3.038945403	-3.917802865	0.000680109	0.047601134
cg13524267	-0.436466333	-1.804148929	-3.917774468	0.000680157	0.047601134
cg07291387	-0.373601662	2.497393025	-3.917688023	0.000680303	0.047601134
cg02381426	-0.632255217	2.79410302	-3.917656584	0.000680356	0.047601134
cg06289323	-0.508101345	2.691156899	-3.917616312	0.000680424	0.047601134
cg10244128	-0.434999852	3.671681814	-3.917584705	0.000680478	0.047601134
cg08858888	-0.467788282	-0.124142302	-3.917581307	0.000680484	0.047601134
cg09670739	-0.268303505	3.866593703	-3.917557216	0.000680525	0.047601134
cg20583714	-0.642566071	2.543451343	-3.917548025	0.00068054	0.047601134
cg06425791	-0.475439112	4.305034824	-3.917514859	0.000680596	0.047601134
cg03959918	-0.51238054	3.732759927	-3.917449638	0.000680707	0.047601134
cg19311889	0.50404419	-4.333387541	3.917446832	0.000680711	0.047601134
cg22901226	0.568350567	-3.136233698	3.917431745	0.000680737	0.047601134
cg13214630	-0.490653017	-0.184187409	-3.917427772	0.000680744	0.047601134
cg15832408	-0.485765002	3.875426544	-3.917323139	0.000680921	0.047601134
cg19616274	-0.357328163	-1.106134334	-3.917319162	0.000680928	0.047601134
cg09039201	0.593714781	3.081701567	3.917302136	0.000680957	0.047601134
cg05706684	-0.595719075	2.739990588	-3.917279948	0.000680994	0.047601134
cg03893806	-0.477021731	3.474022108	-3.917214055	0.000681106	0.047601134
cg25846864	-0.499217085	3.397735893	-3.917212927	0.000681108	0.047601134
cg12644393	-0.415521429	4.03119483	-3.917107483	0.000681286	0.047606484
cg21151541	-0.479913615	2.833529108	-3.917088455	0.000681319	0.047606484
cg25671025	-0.631578244	2.391363901	-3.917016274	0.000681441	0.04760919
cg09482308	-0.438657344	2.530537095	-3.916982544	0.000681498	0.04760919
cg01774131	-0.528608545	2.993241675	-3.916946692	0.000681559	0.04760919
cg21121742	-0.499623036	-1.716557688	-3.916816359	0.00068178	0.047610432
cg01140244	-0.428042379	-0.394975493	-3.916810056	0.000681791	0.047610432
cg01250446	-0.389205782	-0.206272369	-3.916773138	0.000681853	0.047610432
cg17306004	-0.296128995	-0.567542216	-3.916741186	0.000681908	0.047610432
cg22240492	-0.555801681	3.300729357	-3.916738075	0.000681913	0.047610432
cg12301301	-0.509478647	3.560597624	-3.91666561	0.000682036	0.047611546
cg17769523	-0.517638042	3.21408835	-3.916647067	0.000682067	0.047611546
cg13930766	-0.519575087	1.420882143	-3.916609844	0.00068213	0.047611546
cg19021144	-0.773259495	2.288717893	-3.916563719	0.000682209	0.04761223
cg18865126	-0.437497093	0.47720072	-3.916524872	0.000682275	0.04761223

cg02642363	-0.477579013	1.196140986	-3.916368191	0.000682541	0.047626101
cg17291385	0.583029611	3.302778394	3.916305142	0.000682648	0.047628881
cg17233089	-0.421564301	-1.547828687	-3.916216939	0.000682798	0.047634642
cg07672076	-0.480556422	3.894890836	-3.916144806	0.00068292	0.047636134
cg09389280	-0.50534178	0.691989439	-3.916125182	0.000682953	0.047636134
cg24053811	-0.40965052	4.037408237	-3.916013146	0.000683144	0.047639632
cg10822986	-0.633962958	2.903900428	-3.915979391	0.000683201	0.047639632
cg26127114	0.326470733	1.031368127	3.915976956	0.000683205	0.047639632
cg18688142	-0.438145061	0.315721953	-3.915924297	0.000683295	0.047640821
cg07801265	-0.704224832	3.041706897	-3.915887803	0.000683357	0.047640821
cg13606991	-0.541909721	1.945342057	-3.915656699	0.00068375	0.047653659
cg15158045	-0.322607104	0.837264255	-3.915647381	0.000683766	0.047653659
cg00500227	-0.383504833	3.835453545	-3.915573899	0.000683891	0.047653659
cg24020235	-0.46726396	-0.897345374	-3.915570179	0.000683897	0.047653659
cg17855178	-0.601880704	-1.551936209	-3.915532447	0.000683961	0.047653659
cg10551426	-0.650685847	1.782621576	-3.915326576	0.000684312	0.047653659
cg17510859	-0.503296009	3.226757967	-3.915204187	0.00068452	0.047653659
cg16616370	-0.554357791	3.620202577	-3.915191929	0.000684541	0.047653659
cg16744996	-0.413126718	-1.365037043	-3.915116573	0.000684669	0.047653659
cg07178008	-0.502839628	3.039438062	-3.915101191	0.000684695	0.047653659
cg10958840	-0.553055548	3.396861021	-3.915095909	0.000684704	0.047653659
cg16377738	0.709468011	0.076322341	3.915041687	0.000684797	0.047653659
cg17322500	-0.399736484	3.61321137	-3.915008905	0.000684853	0.047653659
cg27101633	-0.645826628	2.413852328	-3.914997889	0.000684871	0.047653659
cg02721684	-0.528992235	2.924180203	-3.914975934	0.000684909	0.047653659
cg09943288	-0.807411704	1.5225919	-3.914958309	0.000684939	0.047653659
cg25909708	-0.617395766	2.634261771	-3.91485632	0.000685113	0.047653659
cg02377131	-0.453399612	3.429304826	-3.914832071	0.000685154	0.047653659
cg16804814	0.602636379	4.281122086	3.914750824	0.000685293	0.047653659
cg01923616	-0.575160228	3.241588161	-3.914745989	0.000685301	0.047653659
cg05782888	-0.539768745	-1.82478767	-3.914722766	0.00068534	0.047653659
cg18813028	-0.411305472	-0.993739588	-3.914678248	0.000685416	0.047653659
cg08538377	-0.684513109	0.024925361	-3.914673172	0.000685425	0.047653659
cg00997969	-0.457973623	4.003157074	-3.914672706	0.000685426	0.047653659
cg24716039	0.550696245	2.887130745	3.914616595	0.000685521	0.047653659
cg17456775	-0.412689137	4.241905544	-3.914611063	0.000685531	0.047653659
cg03143441	-0.597788445	2.618651574	-3.914543985	0.000685645	0.047653659
cg25342822	-0.560163051	2.552638876	-3.914497919	0.000685724	0.047653659
cg22300252	-0.41940839	4.572227352	-3.914398573	0.000685893	0.047653659
cg24513289	-0.413467701	3.810151962	-3.914358619	0.000685962	0.047653659
cg22998918	-0.55157104	3.063812018	-3.914347288	0.000685981	0.047653659
cg11896546	-0.472905187	-0.649136903	-3.91433628	0.000686	0.047653659
cg13852181	-0.459198612	3.88142573	-3.91432595	0.000686017	0.047653659
cg25524233	-0.462938869	4.114589458	-3.914298595	0.000686064	0.047653659
cg27449976	-0.481822986	3.137754058	-3.914229548	0.000686182	0.047653659
cg16359035	-0.479907712	3.388392026	-3.914210794	0.000686214	0.047653659
cg13447847	-0.483050204	3.939252444	-3.914133247	0.000686346	0.047653659

cg01873305	-0.631363348	2.335784986	-3.914101028	0.000686401	0.047653659
cg07311312	-0.415692838	4.117452175	-3.914099255	0.000686404	0.047653659
cg12494446	-0.376192214	3.678526398	-3.913984085	0.000686601	0.047653659
cg11563307	-0.386478585	-1.586214875	-3.9139658	0.000686632	0.047653659
cg04237003	-0.572763413	0.122802136	-3.913942861	0.000686671	0.047653659
cg01546986	-0.796314804	2.831226693	-3.913923794	0.000686704	0.047653659
cg03997145	-0.448414754	-0.76669964	-3.913855426	0.000686821	0.047653659
cg21055546	-0.583279939	-0.207449873	-3.91383545	0.000686855	0.047653659
cg09426368	-0.404956427	3.014472575	-3.913728471	0.000687038	0.047653659
cg15043605	-0.670450319	3.538599156	-3.913715844	0.000687059	0.047653659
cg00120464	-0.565153106	2.063004614	-3.913714281	0.000687062	0.047653659
cg01379853	-0.626552422	2.445874076	-3.913695849	0.000687093	0.047653659
cg22008144	-0.58577139	3.50580097	-3.913676887	0.000687126	0.047653659
cg12511809	-0.499855001	3.56896576	-3.913658572	0.000687157	0.047653659
cg16004467	-0.490235741	-1.361581845	-3.913631855	0.000687203	0.047653659
cg19092987	-0.519336223	3.739045881	-3.913615778	0.00068723	0.047653659
cg26664137	0.604183763	2.600510843	3.91352464	0.000687386	0.047653659
cg09213389	-0.608343347	2.026131696	-3.913524064	0.000687387	0.047653659
cg10501036	-0.733868202	-2.872936536	-3.913523851	0.000687388	0.047653659
cg00245808	-0.428443722	0.148661897	-3.913484513	0.000687455	0.047653659
cg06944489	-0.724326283	4.624764466	-3.913452686	0.000687509	0.047653659
cg25495195	-0.838274633	-1.992363494	-3.9133605	0.000687667	0.047653659
cg07987728	-0.46707836	-1.103835627	-3.913347607	0.000687689	0.047653659
cg06754876	0.713280374	3.535137881	3.913344287	0.000687695	0.047653659
cg14131777	-0.387567722	4.271241422	-3.913334251	0.000687712	0.047653659
cg05546369	-0.439498181	-1.739780425	-3.913218449	0.00068791	0.047657173
cg07056260	-0.591110384	3.751614792	-3.913193181	0.000687953	0.047657173
cg18155464	-0.470934495	3.75312024	-3.913186665	0.000687964	0.047657173
cg17693122	-0.54274299	3.430660402	-3.913084119	0.00068814	0.047664671
cg04523661	-0.824809066	-1.115644256	-3.912933266	0.000688398	0.04766757
cg03338123	-0.761146056	-0.743094343	-3.912927881	0.000688407	0.04766757
cg00722789	-0.438832848	3.699858033	-3.912921395	0.000688418	0.04766757
cg12796089	-0.512977956	3.747156831	-3.912838246	0.000688561	0.04766757
cg08682559	-0.499093084	3.444398749	-3.912820029	0.000688592	0.04766757
cg04936346	-0.578860164	3.403802038	-3.912743899	0.000688723	0.04766757
cg09022607	-0.339009848	-0.739868014	-3.912719748	0.000688764	0.04766757
cg21159639	-0.443163459	0.721666638	-3.912711997	0.000688777	0.04766757
cg17952474	-0.627997305	2.192813217	-3.912622785	0.00068893	0.04766757
cg15731334	-0.800702227	0.166878563	-3.912590614	0.000688985	0.04766757
cg08695650	-0.572450286	2.648984353	-3.912569918	0.000689021	0.04766757
cg16024801	-0.609689809	2.576218967	-3.912504273	0.000689133	0.04766757
cg02102363	-0.389834571	-0.76383687	-3.912497982	0.000689144	0.04766757
cg25107624	-0.7102073	1.099556669	-3.912455566	0.000689217	0.04766757
cg01744322	-0.513695867	3.780724565	-3.912432728	0.000689256	0.04766757
cg23916451	-0.608636679	2.529283803	-3.912431262	0.000689258	0.04766757
cg00109102	-0.65999884	1.632295993	-3.912386462	0.000689335	0.047668229
cg00320779	-0.488299315	3.084379019	-3.912159301	0.000689725	0.047686715

cg08860879	-0.468369977	2.138500169	-3.91213947	0.000689759	0.047686715
cg01208184	-0.578621613	0.421793305	-3.912070988	0.000689876	0.047686715
cg12708821	-0.569349347	-1.266118075	-3.912063051	0.00068989	0.047686715
cg14076451	-0.736438528	-0.276591508	-3.911995409	0.000690006	0.047686715
cg22008020	-0.564379234	3.231008788	-3.911920213	0.000690135	0.047686715
cg07782924	-0.488233615	4.07601998	-3.911895174	0.000690178	0.047686715
cg02958843	-0.435694602	4.039915701	-3.91187938	0.000690205	0.047686715
cg22441338	-0.489756187	2.745582982	-3.911877574	0.000690208	0.047686715
cg24390932	-0.387063906	0.368478027	-3.91181494	0.000690316	0.047688832
cg23068767	-0.71572288	1.99280618	-3.911733853	0.000690455	0.047688832
cg11847185	-0.545350297	3.141118314	-3.911690561	0.00069053	0.047688832
cg24290487	-0.42614112	-0.011607678	-3.911677654	0.000690552	0.047688832
cg12337336	-0.44781411	4.146632891	-3.911663738	0.000690576	0.047688832
cg04250451	-0.424848747	-0.949766899	-3.911448069	0.000690946	0.047709773
cg15036894	-0.47315143	-1.153214953	-3.911366379	0.000691087	0.047714818
cg20741864	-0.365654858	4.352071748	-3.911119423	0.000691511	0.047732491
cg18162371	-0.460683526	0.20575008	-3.911057193	0.000691618	0.047732491
cg02052536	-0.34793954	-0.346593826	-3.910991862	0.000691731	0.047732491
cg09092489	-0.848972874	2.94035869	-3.910991069	0.000691732	0.047732491
cg27635253	-0.530664395	3.398159007	-3.910956205	0.000691792	0.047732491
cg22419732	-0.514626903	2.777120211	-3.910952957	0.000691798	0.047732491
cg25233203	-0.536041973	3.064401914	-3.910943324	0.000691814	0.047732491
cg15256538	-0.495263996	3.473865762	-3.910811102	0.000692042	0.047743543
cg25686087	-0.312741367	-0.19020679	-3.910648959	0.000692321	0.047756905
cg04854637	-0.368934894	-1.106655519	-3.91059915	0.000692407	0.047756905
cg26289892	-0.552357508	3.403928924	-3.91050843	0.000692563	0.047756905
cg10137450	-0.732486983	3.075347339	-3.91050673	0.000692566	0.047756905
cg16348324	-0.447836769	3.839596866	-3.910361669	0.000692816	0.047756905
cg08046604	-0.55037774	2.875250372	-3.910282236	0.000692953	0.047756905
cg14394740	-0.504199689	2.53429239	-3.910226007	0.00069305	0.047756905
cg06344162	-0.519394496	3.283446177	-3.910224191	0.000693053	0.047756905
cg02583494	-0.443633423	0.210794039	-3.910150982	0.000693179	0.047756905
cg17200441	-0.463895196	-1.549331181	-3.91013767	0.000693202	0.047756905
cg19764295	-0.504168934	0.121006136	-3.910132129	0.000693212	0.047756905
cg13983864	-0.529147766	3.720201346	-3.910105092	0.000693258	0.047756905
cg10080728	-0.366463762	-0.805376175	-3.910094425	0.000693277	0.047756905
cg07150473	-0.517565535	2.033840816	-3.910033931	0.000693381	0.047756905
cg12113758	-0.732982317	-2.818441876	-3.910033072	0.000693383	0.047756905
cg19699200	-0.456243406	0.035454884	-3.90998177	0.000693471	0.047756905
cg22650137	-0.448971797	-0.548992994	-3.909957305	0.000693513	0.047756905
cg14769993	-0.525259451	3.692778054	-3.909936512	0.000693549	0.047756905
cg02808257	-0.446358115	-0.287939953	-3.909924258	0.00069357	0.047756905
cg26314281	-0.36345537	3.728155851	-3.90989962	0.000693613	0.047756905
cg10831683	-0.602941547	3.032900855	-3.909877257	0.000693651	0.047756905
cg19681626	-0.381383285	4.132816909	-3.909760498	0.000693853	0.047762238
cg20430924	-0.479249492	3.899986154	-3.909718278	0.000693926	0.047762238
cg26282025	-0.490902839	2.491206591	-3.909650996	0.000694042	0.047762238

cg19376807	-0.426806767	2.959914416	-3.909645101	0.000694052	0.047762238
cg02138806	-0.540577046	3.657792252	-3.909637049	0.000694066	0.047762238
cg27531731	-0.623398902	-0.926303412	-3.909597113	0.000694135	0.047762343
cg00122781	-0.403005371	4.269543638	-3.909443956	0.000694399	0.04777065
cg20384613	-0.415611342	3.86586445	-3.90939513	0.000694484	0.04777065
cg07565847	-0.432959826	4.338653132	-3.909360093	0.000694544	0.04777065
cg01622124	-0.931123149	1.973374799	-3.909303014	0.000694643	0.04777065
cg27172769	-0.629021878	1.016817112	-3.909257587	0.000694721	0.04777065
cg03284061	-0.494918868	0.140723712	-3.909254777	0.000694726	0.04777065
cg18574396	-0.363101311	4.210647886	-3.909253938	0.000694728	0.04777065
cg26489751	-0.467817389	1.015235951	-3.909211244	0.000694801	0.047771086
cg00475558	-0.421811834	3.644469918	-3.909075988	0.000695035	0.047782523
cg15162597	-0.388617364	-0.911535144	-3.909028998	0.000695116	0.047783471
cg08105174	-0.41342909	4.08088775	-3.908903346	0.000695334	0.047787006
cg12874219	-0.370199754	3.546977308	-3.908903088	0.000695334	0.047787006
cg08920233	-0.38821268	4.101281192	-3.908848541	0.000695429	0.047787006
cg03705558	-0.547889266	-2.226299014	-3.908842113	0.00069544	0.047787006
cg09191958	0.668210496	3.315287805	3.908804239	0.000695505	0.047787006
cg17671100	-0.421978953	-0.217243008	-3.908629141	0.000695808	0.047803187
cg05163650	-0.507011584	3.92299045	-3.908536401	0.000695969	0.047809579
cg21004290	-0.457316236	3.622271059	-3.908474603	0.000696076	0.047812292
cg22542334	-0.579576344	3.550999256	-3.908379852	0.00069624	0.047818925
cg12249234	-0.491491159	-1.895269062	-3.908324382	0.000696336	0.047820886
cg08710911	-0.468145033	-1.404645055	-3.908285294	0.000696404	0.0478209
cg01157470	-0.518085669	3.411341211	-3.908175884	0.000696593	0.047825589
cg01651296	-0.39053751	-0.150256508	-3.908120671	0.000696689	0.047825589
cg17205652	-0.514083067	3.298887054	-3.908053654	0.000696805	0.047825589
cg06121719	-0.528530748	3.558216557	-3.908044098	0.000696821	0.047825589
cg08080919	-0.437714372	3.781138631	-3.908037057	0.000696834	0.047825589
cg01655161	-0.524983014	3.507286904	-3.907896014	0.000697078	0.047825589
cg10151196	-0.376887539	-0.443478326	-3.90785798	0.000697144	0.047825589
cg11048682	-0.577433803	-0.112350093	-3.907853962	0.000697151	0.047825589
cg24094897	-0.571032305	-0.02104053	-3.907848635	0.00069716	0.047825589
cg08397344	-0.371963181	-1.085291494	-3.90775932	0.000697315	0.047825589
cg01725746	0.550321742	2.431729547	3.907664002	0.000697481	0.047825589
cg00608338	-0.42774546	3.37260674	-3.907662435	0.000697483	0.047825589
cg27002247	0.969315144	-0.592284879	3.907563226	0.000697655	0.047825589
cg16098001	-0.427842974	3.795796701	-3.907474355	0.00069781	0.047825589
cg03239746	-0.435271046	3.666410798	-3.907459272	0.000697836	0.047825589
cg23525708	-0.618602616	-2.207562713	-3.907424462	0.000697896	0.047825589
cg25703598	-0.354501466	3.722177601	-3.907385068	0.000697965	0.047825589
cg19533981	-0.614594078	1.458324788	-3.907384044	0.000697966	0.047825589
cg03549129	-0.38704243	4.033878553	-3.90735872	0.00069801	0.047825589
cg21961901	-0.558953131	3.094130406	-3.907355696	0.000698016	0.047825589
cg05876246	-0.738753951	-1.419939232	-3.907290465	0.000698129	0.047825589
cg06487138	-0.347738574	4.145927541	-3.907242197	0.000698213	0.047825589
cg03668686	-0.334288717	-0.015983074	-3.907195985	0.000698293	0.047825589

cg22796480	-0.533956647	3.929828362	-3.907181123	0.000698319	0.047825589
cg19149132	-0.410703555	-0.505499771	-3.907133384	0.000698402	0.047825589
cg13674711	-0.457420424	3.133770829	-3.907076346	0.000698501	0.047825589
cg14724841	-0.553281157	3.838541281	-3.907055492	0.000698537	0.047825589
cg25644284	-0.728993833	-1.034780172	-3.907002652	0.000698629	0.047825589
cg25578013	-0.475177793	0.382534902	-3.906986138	0.000698657	0.047825589
cg19439285	-0.411705993	3.690281718	-3.906985501	0.000698659	0.047825589
cg10533098	-0.634519932	1.89278027	-3.906955166	0.000698711	0.047825589
cg20695627	-0.544477242	-1.320885019	-3.906938174	0.000698741	0.047825589
cg00368292	-0.350487509	4.244639532	-3.906928613	0.000698757	0.047825589
cg19039809	-0.770054828	2.992226733	-3.906896816	0.000698813	0.047825589
cg04481584	-0.826114059	2.391863917	-3.90681448	0.000698956	0.047825589
cg05837928	-0.496565757	3.472286507	-3.906774982	0.000699025	0.047825589
cg08909330	-0.563942989	3.608760826	-3.906632069	0.000699273	0.047825589
cg13693322	-0.48697843	3.768491891	-3.906582176	0.00069936	0.047825589
cg09430020	-0.821350486	0.174899589	-3.906563003	0.000699393	0.047825589
cg00452530	-0.523545488	3.926600569	-3.906554323	0.000699408	0.047825589
cg16840742	-0.615488465	2.955025811	-3.906541807	0.00069943	0.047825589
cg05249959	-0.508082706	-0.97706707	-3.906524492	0.00069946	0.047825589
cg20205515	-0.454078534	3.144139147	-3.906513187	0.00069948	0.047825589
cg21363377	-0.580239639	-1.673328775	-3.906445396	0.000699598	0.047825589
cg16740157	-0.890440799	0.731789026	-3.906423451	0.000699636	0.047825589
cg10433625	-0.510059887	0.511417449	-3.906407643	0.000699663	0.047825589
cg08621778	-0.367269545	-0.745423978	-3.906331379	0.000699796	0.047825589
cg25328718	-0.736829724	2.459460053	-3.906322107	0.000699812	0.047825589
cg23808183	-0.709841876	1.594032788	-3.906256242	0.000699927	0.047825589
cg23982880	-0.458708433	3.058501635	-3.906217177	0.000699995	0.047825589
cg18806033	-0.559224598	3.172102752	-3.906207285	0.000700012	0.047825589
cg07946300	-0.421470388	3.326047554	-3.906191725	0.000700039	0.047825589
cg05503761	-0.478411646	1.256757542	-3.906185582	0.00070005	0.047825589
cg09039287	-0.65388152	-0.046025158	-3.90613554	0.000700137	0.047826929
cg05993202	-0.50672589	2.654377401	-3.906041648	0.000700301	0.047832529
cg26986062	-0.676333013	2.221962999	-3.905987999	0.000700394	0.047832529
cg22051537	-0.744844866	2.244694869	-3.905943904	0.000700471	0.047832529
cg16873385	-0.695184032	2.980593976	-3.905857309	0.000700622	0.047832529
cg17294136	-0.514220665	-1.600932805	-3.905710968	0.000700877	0.047832529
cg10521480	-0.414728804	-0.543115667	-3.90568824	0.000700916	0.047832529
cg05733557	-0.706814548	1.687086196	-3.905674834	0.00070094	0.047832529
cg11797898	-0.453396326	0.355203726	-3.905647607	0.000700987	0.047832529
cg05866917	-0.583789437	3.805868782	-3.905614297	0.000701045	0.047832529
cg21692850	-0.398149967	3.71626474	-3.905611043	0.000701051	0.047832529
cg00425361	-0.485767452	3.412175556	-3.905606463	0.000701059	0.047832529
cg25797507	-0.550549897	3.639724256	-3.905577842	0.000701109	0.047832529
cg02537941	-0.604809991	-0.269663299	-3.905469449	0.000701298	0.047832529
cg27522205	-0.408227162	-0.194639586	-3.905438481	0.000701352	0.047832529
cg07010741	-0.65206225	-0.016771929	-3.905413839	0.000701395	0.047832529
cg20190922	-0.671369083	-0.937729529	-3.905370193	0.000701471	0.047832529

cg18852038	-0.371846035	3.795520242	-3.905360056	0.000701489	0.047832529
cg25799710	-0.444506873	3.987812932	-3.905353945	0.0007015	0.047832529
cg05444288	-0.331876469	-0.465436565	-3.905304122	0.000701586	0.047832529
cg20310415	0.655365007	3.022708409	3.905184432	0.000701795	0.047832529
cg26662324	-0.777308058	-2.600402963	-3.905161824	0.000701835	0.047832529
cg09106769	-0.570577951	2.767764345	-3.905159696	0.000701838	0.047832529
cg16103098	-0.542935579	3.103099376	-3.905152634	0.000701851	0.047832529
cg27628891	-0.916713138	-1.436795145	-3.905087125	0.000701965	0.047832529
cg17356355	-0.639328509	3.171800577	-3.905064441	0.000702005	0.047832529
cg23253006	0.471496674	2.273293706	3.905011022	0.000702098	0.047832529
cg06199909	-0.632536792	3.028378375	-3.904980528	0.000702151	0.047832529
cg01334081	-0.548112007	3.444777205	-3.904973474	0.000702164	0.047832529
cg09931724	-0.653485689	3.416912196	-3.90496564	0.000702177	0.047832529
cg15335334	-0.473762886	-0.750506971	-3.904895754	0.000702299	0.047833136
cg17997285	-0.735338589	2.473887794	-3.904860089	0.000702362	0.047833136
cg12726332	-0.575964507	3.453989459	-3.904751124	0.000702552	0.047833136
cg11459681	-0.547021888	-0.901796926	-3.904740499	0.000702571	0.047833136
cg18263879	-0.494191943	3.954591968	-3.904718291	0.000702609	0.047833136
cg00092916	-0.469004689	3.908769309	-3.904671602	0.000702691	0.047833136
cg17388638	-0.472387155	3.454466623	-3.904656831	0.000702717	0.047833136
cg26748079	-0.490351262	0.069909623	-3.904650535	0.000702728	0.047833136
cg17035787	0.390524596	2.051572512	3.904612703	0.000702794	0.047833136
cg21348908	-0.431047932	-0.125253464	-3.904371546	0.000703216	0.047856082
cg24817763	-0.740137459	-4.574076186	-3.904342617	0.000703266	0.047856082
cg05404441	-0.527209088	3.020756919	-3.904233774	0.000703457	0.04786444
cg12749653	-0.500773405	-0.74428756	-3.904098652	0.000703693	0.047873172
cg15476723	-0.410249244	-1.08290212	-3.904083192	0.00070372	0.047873172
cg25472614	-0.350388437	3.678488246	-3.904013345	0.000703842	0.047873213
cg20594430	-0.434351348	3.849563634	-3.904005629	0.000703856	0.047873213
cg14457499	-0.617909458	4.192774265	-3.903856264	0.000704117	0.047886402
cg18278736	-0.413073446	0.52460857	-3.903706735	0.000704379	0.047895099
cg16338844	0.59577615	3.569277282	3.903618187	0.000704534	0.047895099
cg02266222	-0.662549156	2.751525377	-3.903617332	0.000704536	0.047895099
cg13704331	-0.447296731	4.338201026	-3.903594287	0.000704576	0.047895099
cg00433866	-0.625168566	-0.03087097	-3.903590282	0.000704583	0.047895099
cg17256227	-0.438450832	3.428988132	-3.903492846	0.000704754	0.047899683
cg06807288	-0.54444427	3.425772685	-3.903471072	0.000704792	0.047899683
cg14338472	-0.410550934	3.615221647	-3.903342436	0.000705018	0.047899683
cg21638053	-0.444381286	4.035126339	-3.903316962	0.000705062	0.047899683
cg19010166	-0.566568537	2.999390285	-3.903273081	0.000705139	0.047899683
cg05180880	-0.474083289	0.158542764	-3.90325902	0.000705164	0.047899683
cg06049367	-0.527415685	0.120731034	-3.903240404	0.000705197	0.047899683
cg19671375	-0.455340072	3.453705044	-3.903222025	0.000705229	0.047899683
cg20798124	-0.641780467	2.899437214	-3.903204681	0.000705259	0.047899683
cg17255277	-0.624814632	2.976252138	-3.903156726	0.000705343	0.047900804
cg02843331	-2.075917693	2.499420496	-3.903081361	0.000705476	0.04790519
cg21913528	-0.404057199	0.273607807	-3.902992802	0.000705631	0.047910833

cg19186888	-0.498427682	0.378362723	-3.902951921	0.000705703	0.047910833
cg09388590	-0.478988439	-1.775613552	-3.90288028	0.000705829	0.047910833
cg20311522	-0.695759408	0.956473226	-3.902872881	0.000705842	0.047910833
ch.16.1711323F	-0.810133418	-3.242205564	-3.902829293	0.000705918	0.047910833
cg05053708	-0.465102652	0.52868068	-3.902788215	0.00070599	0.047910833
cg04267686	-0.433742532	4.21112721	-3.90273421	0.000706085	0.047910833
cg00712735	-0.625154434	3.644927874	-3.902706561	0.000706134	0.047910833
cg08360599	-0.488377553	-1.808322424	-3.902687247	0.000706167	0.047910833
cg01998719	-0.466477452	3.897077605	-3.902605268	0.000706311	0.047911537
cg14943012	-0.459284014	3.623839704	-3.902604325	0.000706313	0.047911537
cg24149024	-0.386530324	3.839603938	-3.902531554	0.000706441	0.047913287
cg19388805	-0.552970831	2.280914265	-3.902493431	0.000706508	0.047913287
cg25337965	-0.539809786	-0.588745436	-3.902437976	0.000706605	0.047913287
cg20490341	-0.556043876	-0.377298783	-3.902369966	0.000706725	0.047913287
cg13729541	-0.424112491	-1.986791227	-3.902325004	0.000706804	0.047913287
cg13993183	-0.585334688	-0.413953793	-3.902307634	0.000706834	0.047913287
cg09161656	-0.431586844	-1.249856848	-3.90228732	0.00070687	0.047913287
cg22411162	-0.622890746	2.384923175	-3.902249653	0.000706936	0.047913287
cg14364065	-0.420502279	-0.873639939	-3.902243244	0.000706948	0.047913287
cg06215562	-0.572595069	1.198131205	-3.902174019	0.000707069	0.047916952
cg17495046	0.38603756	1.718720879	3.901993556	0.000707387	0.047927047
cg13762464	0.603575887	2.441956863	3.901948918	0.000707465	0.047927047
cg15843531	-0.453695457	3.370005361	-3.901936226	0.000707488	0.047927047
cg17805581	-0.473349779	3.608025112	-3.901896732	0.000707557	0.047927047
cg10084213	-0.430927931	-0.27795639	-3.901881647	0.000707584	0.047927047
cg03757501	-0.472422516	3.924372311	-3.90185857	0.000707624	0.047927047
cg20073882	-0.471036436	3.077715121	-3.901769265	0.000707781	0.047933109
cg21048924	-0.493438199	3.249009239	-3.901722055	0.000707865	0.047933193
cg09630122	-0.492305882	3.564873008	-3.901685187	0.000707929	0.047933193
cg21089903	0.948181271	-5.115606047	3.901644826	0.000708	0.047933193
cg07372384	-0.488654132	3.35949175	-3.901614824	0.000708053	0.047933193
cg00502062	-0.39291864	-0.858434768	-3.90149335	0.000708267	0.047943093
cg16060302	-0.415124582	-0.766425968	-3.90139492	0.000708441	0.047944215
cg02268535	-0.843144299	-3.022203956	-3.901358197	0.000708505	0.047944215
cg17234605	-0.696671951	2.82316717	-3.901341703	0.000708534	0.047944215
cg21593161	-0.491697572	3.428921758	-3.901329151	0.000708557	0.047944215
cg23400169	-0.532675392	0.625494239	-3.901256877	0.000708684	0.047944215
cg05112948	-0.560754975	3.648163686	-3.90125346	0.00070869	0.047944215
cg26139552	-0.468050273	3.660495524	-3.901208752	0.000708769	0.047944967
cg17480669	-0.47365759	-1.33765507	-3.901018759	0.000709104	0.047955727
cg10214622	-0.543099959	2.996941649	-3.900893785	0.000709324	0.047955727
cg23323395	-0.378963647	3.858250981	-3.900845782	0.000709409	0.047955727
cg20431785	-0.609140307	1.713299746	-3.900834512	0.000709429	0.047955727
cg12063952	-0.421228686	3.646228538	-3.900797838	0.000709493	0.047955727
ch.4.167276096F	-0.668796999	-3.20157045	-3.900796558	0.000709496	0.047955727
cg03722472	-0.431277841	0.220674687	-3.900667065	0.000709724	0.047955727
cg07934264	-1.159046117	2.764381344	-3.900641835	0.000709769	0.047955727

cg14948128	-0.57514517	3.447445481	-3.900620097	0.000709807	0.047955727
cg08823209	0.351986227	0.750734223	3.900615835	0.000709815	0.047955727
cg17668756	-0.437967529	-0.713697142	-3.900612184	0.000709821	0.047955727
cg02646779	-0.500316908	2.530049488	-3.900552921	0.000709926	0.047955727
cg23052520	-0.813567851	1.963992764	-3.900547043	0.000709936	0.047955727
cg19229215	-0.363750217	-1.036220868	-3.900500446	0.000710018	0.047955727
cg08447324	-0.412411462	-2.649490819	-3.900495914	0.000710026	0.047955727
cg22518785	-0.666430119	2.627614944	-3.900457866	0.000710093	0.047955727
cg05301191	-0.464118426	3.763887857	-3.900439752	0.000710125	0.047955727
cg24861413	-0.518603257	3.464512833	-3.900427916	0.000710146	0.047955727
cg13968283	-0.399341661	3.565497935	-3.900180153	0.000710584	0.047971278
cg03228292	-0.558638901	0.789608394	-3.900173625	0.000710596	0.047971278
cg08840857	-0.532216254	3.587851971	-3.900075113	0.00071077	0.047971278
cg16189976	-0.685555605	2.324264365	-3.900060305	0.000710796	0.047971278
cg11152513	-0.410444365	0.014519995	-3.9000572	0.000710801	0.047971278
cg04055345	-0.486756835	-0.608088006	-3.900006661	0.000710891	0.047971278
cg23122032	-0.388718028	-0.725962357	-3.899990535	0.000710919	0.047971278
cg01784549	-0.501201299	3.629461884	-3.899986334	0.000710927	0.047971278
cg14361551	-0.585869067	3.518685995	-3.89994028	0.000711008	0.047971278
cg02137282	-0.41795054	1.104910656	-3.899874361	0.000711125	0.047971278
cg25105316	-0.503053027	3.626575747	-3.899832888	0.000711198	0.047971278
cg14987175	-0.350275266	-0.14330308	-3.899773774	0.000711302	0.047971278
cg16472896	0.484359704	2.943899348	3.899714989	0.000711406	0.047971278
cg17410660	-0.482693344	-0.85420789	-3.89968204	0.000711465	0.047971278
cg14382954	0.490198592	4.200546941	3.89965845	0.000711506	0.047971278
cg18319213	-0.43260144	4.067996283	-3.899633035	0.000711551	0.047971278
cg13780303	0.535615571	3.009813429	3.899557943	0.000711684	0.047971278
cg09309117	0.596526605	2.444714635	3.89955477	0.00071169	0.047971278
cg06055755	-0.606940222	2.476676103	-3.899548476	0.000711701	0.047971278
cg25853406	-0.357684655	3.45072579	-3.899424383	0.000711921	0.047971278
cg04496284	-0.476222773	3.329376633	-3.899369164	0.000712018	0.047971278
cg19275526	-0.582819557	3.252947506	-3.899336847	0.000712076	0.047971278
cg00762672	-0.567479393	2.882500579	-3.899328474	0.000712091	0.047971278
cg05037389	-0.559065061	-1.542454442	-3.899308518	0.000712126	0.047971278
cg25359168	-0.606118089	1.834986289	-3.899241786	0.000712244	0.047971278
cg23266840	-0.410107863	-0.834296494	-3.899187233	0.000712341	0.047971278
cg05001349	-0.812511487	1.313565327	-3.89916257	0.000712384	0.047971278
cg26087524	-0.541544527	1.972086332	-3.899125639	0.00071245	0.047971278
cg15686474	-0.542993374	-0.690835857	-3.899118934	0.000712462	0.047971278
cg16867962	-0.674695895	2.17588093	-3.899064	0.000712559	0.047971278
cg13911398	-0.640398942	2.702772781	-3.899033445	0.000712613	0.047971278
cg16163735	-0.577123739	2.964364757	-3.899016086	0.000712644	0.047971278
cg17597911	0.525570114	2.788904328	3.898926682	0.000712802	0.047971278
cg13199913	-0.362678842	0.295126204	-3.898915832	0.000712822	0.047971278
cg23786205	-0.31930526	0.677763906	-3.898908016	0.000712835	0.047971278
cg21282452	-0.43498104	3.57805002	-3.898870553	0.000712902	0.047971278
cg23795362	-0.442494363	-0.440921508	-3.898870154	0.000712903	0.047971278

cg26142862	0.528874834	2.412552456	3.898819724	0.000712992	0.047971278
cg01858274	-0.429638334	-0.074633554	-3.898734274	0.000713143	0.047971278
cg14077078	-0.478277897	3.163090222	-3.898715487	0.000713177	0.047971278
cg12172295	-0.451605077	-1.107306865	-3.898670271	0.000713257	0.047971278
cg19772199	-0.438566919	3.300396914	-3.898625116	0.000713337	0.047971278
cg05665555	-0.362283567	3.821525416	-3.898614587	0.000713356	0.047971278
cg10369206	-0.600283938	-3.326101934	-3.898614306	0.000713356	0.047971278
cg17498694	-0.513939432	4.26767568	-3.89855071	0.000713469	0.047974311
cg14863830	-0.540158726	3.066729978	-3.898480153	0.000713594	0.047978175
cg07676043	-0.590673791	2.934897167	-3.898328787	0.000713863	0.047985167
cg11352681	-0.38973759	-0.440109991	-3.898316067	0.000713885	0.047985167
cg01437030	-0.642874523	1.41429933	-3.898307058	0.000713901	0.047985167
cg11007201	-0.544892027	3.60053777	-3.898248456	0.000714006	0.047987606
cg23311967	-0.43938535	3.961294998	-3.898178882	0.000714129	0.047987672
cg07628295	-0.405094478	-0.099747111	-3.898171608	0.000714142	0.047987672
cg00251716	-0.625065287	-0.151797001	-3.897961635	0.000714515	0.047988199
cg10064663	-0.460860898	3.346811657	-3.897923811	0.000714582	0.047988199
cg07456586	-0.930610501	1.263158492	-3.897812926	0.000714779	0.047988199
cg12508976	0.562087217	2.4818614	3.897809067	0.000714786	0.047988199
cg04109760	-0.43591664	1.773432993	-3.897759863	0.000714874	0.047988199
cg24264451	-0.558606784	2.646233871	-3.897748974	0.000714893	0.047988199
cg22651048	-0.45268713	3.736515202	-3.897724029	0.000714937	0.047988199
cg10398107	-0.448506396	4.112432297	-3.897699603	0.000714981	0.047988199
cg05579422	0.702252512	2.589523487	3.897667855	0.000715037	0.047988199
cg01171706	-0.456341725	-0.471269239	-3.897650234	0.000715068	0.047988199
cg06387620	-0.502545155	3.442729818	-3.897615793	0.00071513	0.047988199
cg22800743	0.378622343	1.160893174	3.897560746	0.000715228	0.047988199
cg09014062	-0.479668045	3.744081221	-3.897469819	0.000715389	0.047988199
cg23877497	-0.844955122	0.211362253	-3.897410952	0.000715494	0.047988199
cg03314633	-0.714359339	0.005762711	-3.897401184	0.000715511	0.047988199
cg10569553	-0.360312091	0.331762652	-3.897385791	0.000715539	0.047988199
cg16560847	0.502653311	3.160916322	3.897373802	0.00071556	0.047988199
cg20938391	-0.354285721	-0.892576159	-3.897317773	0.00071566	0.047988199
cg13000219	-0.328206108	-0.488478925	-3.897317353	0.000715661	0.047988199
cg08712082	-0.474155272	3.587115611	-3.897302229	0.000715687	0.047988199
cg06363163	-0.586792566	3.121875028	-3.897289354	0.00071571	0.047988199
cg01543641	0.562182994	4.237305285	3.89727424	0.000715737	0.047988199
cg21000520	-0.581547426	2.959152741	-3.897249824	0.000715781	0.047988199
cg23902454	-0.366418434	-0.079000412	-3.897216307	0.00071584	0.047988199
cg04715975	-0.424777546	3.596095437	-3.897214614	0.000715843	0.047988199
cg23655769	0.436377034	-0.054734117	3.897024592	0.000716182	0.048006173
cg06485774	-0.437390236	-0.450580503	-3.896942631	0.000716328	0.048006173
cg25163442	-0.388588909	0.143382373	-3.896934718	0.000716342	0.048006173
cg18084050	-0.518682545	3.380400862	-3.896870217	0.000716457	0.048006173
cg07866798	-0.54311406	-0.15117504	-3.896849688	0.000716493	0.048006173
cg11423891	-0.421525806	0.383223707	-3.896828937	0.00071653	0.048006173
cg08200809	-0.467531014	3.655917109	-3.896797689	0.000716586	0.048006173

cg09969248	-0.393956342	2.926589757	-3.896584886	0.000716965	0.048021607
cg25336779	-0.457648811	3.510199906	-3.896575567	0.000716982	0.048021607
cg18415268	0.371991839	0.591921126	3.896554327	0.00071702	0.048021607
cg13446404	-0.407732869	4.141183755	-3.896451808	0.000717202	0.048029309
cg09929288	0.632847124	3.329508981	3.896311739	0.000717452	0.048035526
cg07476851	-0.488034134	2.70550718	-3.896245097	0.000717571	0.048035526
cg06425843	-0.463530549	-0.213505063	-3.896229714	0.000717599	0.048035526
cg00337703	0.69688708	2.391012526	3.896221638	0.000717613	0.048035526
cg03589085	-0.522611998	2.978363112	-3.896173086	0.0007177	0.048035526
cg23854087	-0.366574481	2.361790659	-3.896169981	0.000717705	0.048035526
cg27585557	-0.584314702	4.647940057	-3.8961337	0.00071777	0.048035526
cg03715182	-0.567096534	2.612682915	-3.89609457	0.00071784	0.048035662
cg09877112	-0.579090318	2.30469399	-3.896043715	0.000717931	0.048037199
cg08276542	-0.880400828	1.199309454	-3.895993616	0.00071802	0.048038646
cg27102138	-0.432689744	3.948148286	-3.895918945	0.000718153	0.048040731
cg23566791	-0.444289919	3.283437631	-3.895850557	0.000718275	0.048040731
cg03821671	-0.42193891	2.482112525	-3.89584159	0.000718291	0.048040731
cg04878939	-0.545154312	0.584716012	-3.895813584	0.000718342	0.048040731
cg03198320	-0.547411452	2.140453581	-3.895786277	0.00071839	0.048040731
cg04055528	-0.685592904	-1.187879452	-3.895720917	0.000718507	0.048044004
cg01980735	-0.464821738	-0.124689211	-3.895682053	0.000718576	0.048044112
cg11429155	-0.629735783	0.407709835	-3.895594699	0.000718733	0.048048274
cg09389615	-0.606557574	0.159623455	-3.895571301	0.000718774	0.048048274
cg08189583	-0.484927248	3.903989589	-3.895513956	0.000718877	0.048050591
cg11708650	-0.39228069	3.951244453	-3.895473775	0.000718949	0.048050858
cg10049835	-0.516312926	3.210706027	-3.895298735	0.000719262	0.04806724
cg17304240	-0.736773804	2.883293872	-3.895247973	0.000719352	0.048068772
cg27522658	-0.447355613	-0.166227683	-3.895144492	0.000719538	0.048076607
cg15926900	-0.638418336	1.913273911	-3.895024932	0.000719752	0.048086365
cg15556990	-0.586673651	2.886072568	-3.894929407	0.000719922	0.048093252
cg20937029	-0.662030841	0.688401246	-3.894856931	0.000720052	0.048097383
cg14197123	-1.045840404	0.151596508	-3.894812218	0.000720132	0.048098196
cg04957377	-0.621635087	-1.782838509	-3.894732408	0.000720275	0.048100741
cg16735351	-0.543803208	3.703574724	-3.8947151	0.000720306	0.048100741
cg15830864	-0.46417928	0.947150937	-3.894632912	0.000720453	0.048106036
cg16452155	-0.440339175	3.634110221	-3.894544205	0.000720612	0.048109535
cg16022692	-0.366457018	4.07838471	-3.89452785	0.000720642	0.048109535
cg04382220	-0.773200926	2.63013846	-3.894432817	0.000720812	0.048110006
cg05303090	-0.440564962	4.067477626	-3.894391281	0.000720886	0.048110006
cg11717660	-0.687706182	3.482402465	-3.894380589	0.000720906	0.048110006
cg17862561	-0.488294196	-1.448183933	-3.894372325	0.00072092	0.048110006
cg01010681	-0.476298951	3.224532183	-3.894256881	0.000721127	0.048112966
cg20526254	-0.700099972	3.109144005	-3.89425445	0.000721132	0.048112966
cg05477331	-0.524445446	3.741280803	-3.894205638	0.000721219	0.048112966
cg24493634	-0.802282663	-0.102345739	-3.894157697	0.000721305	0.048112966
cg07586533	-0.738344759	-3.105820927	-3.894031027	0.000721532	0.048112966
cg13804980	-0.451782556	3.528816549	-3.894028416	0.000721537	0.048112966

cg10632328	-0.63793888	-1.664430449	-3.893990243	0.000721606	0.048112966
cg00250171	-0.474104134	3.525609427	-3.893974682	0.000721633	0.048112966
cg08609766	-0.591775103	2.738322923	-3.893971062	0.00072164	0.048112966
cg24679890	-0.644030886	-3.258303768	-3.893968865	0.000721644	0.048112966
cg08717071	-0.706249448	3.186403085	-3.893905391	0.000721758	0.048116031
cg15114787	-0.519130713	3.558172878	-3.893815255	0.00072192	0.048117882
cg24265943	-0.709121537	2.186147041	-3.893716964	0.000722096	0.048117882
cg07628084	-0.855510794	-1.213014064	-3.893667781	0.000722184	0.048117882
cg05892794	-0.530883516	3.124052319	-3.893653428	0.00072221	0.048117882
cg26672151	-0.534982641	3.660311409	-3.893592935	0.000722319	0.048117882
cg27337540	0.424103752	1.606069624	3.893561307	0.000722376	0.048117882
cg04906629	-0.413324698	-0.240951346	-3.893519953	0.00072245	0.048117882
cg16385939	0.430831541	1.446369253	3.89345932	0.000722559	0.048117882
cg20748012	-0.598216782	3.61658594	-3.893455735	0.000722565	0.048117882
cg07373578	-0.602193534	-0.743055497	-3.893439097	0.000722595	0.048117882
cg02771587	-0.491277899	3.506054155	-3.893426437	0.000722618	0.048117882
cg02100365	-0.489989862	3.473160958	-3.893410369	0.000722647	0.048117882
cg20411258	-0.641443475	2.562886372	-3.89330992	0.000722827	0.048117882
cg22482158	0.313098019	3.00035292	3.893308131	0.00072283	0.048117882
cg01146879	-0.663930973	2.407434243	-3.893298859	0.000722847	0.048117882
cg20864326	-0.489037091	3.432217343	-3.893274949	0.00072289	0.048117882
cg07382923	-0.859030508	-3.38070712	-3.89318257	0.000723056	0.048117882
cg00616552	-0.787927112	-1.813364066	-3.893180508	0.00072306	0.048117882
cg21609584	-0.54969253	3.431641032	-3.893158556	0.000723099	0.048117882
cg23278483	-0.585103236	2.349855318	-3.893090297	0.000723222	0.048117882
cg17430405	-0.503353095	3.401228895	-3.893031213	0.000723328	0.048117882
cg26849049	-0.569752072	2.807348755	-3.893027979	0.000723334	0.048117882
cg07850454	-0.393085925	4.100791594	-3.893020275	0.000723348	0.048117882
cg07139026	-0.61313339	2.730554236	-3.892954284	0.000723467	0.048120324
cg22119466	-0.378192105	-1.580358583	-3.892924334	0.00072352	0.048120324
cg11036631	-0.875868611	-0.545946397	-3.89285711	0.000723641	0.048123849
cg24252287	-0.540073119	-2.278031397	-3.892810355	0.000723725	0.048124925
cg23524283	-0.407054505	2.79048172	-3.892722978	0.000723883	0.048125478
cg07453857	-0.358683881	-0.699086386	-3.892713172	0.0007239	0.048125478
cg05360101	-0.790219043	1.874128724	-3.892692485	0.000723938	0.048125478
cg12962953	-0.516298897	3.544984327	-3.892576197	0.000724147	0.048130937
cg01053396	-0.530296804	-0.185773147	-3.892466343	0.000724345	0.048130937
cg21440166	0.607723337	1.988459633	3.892377492	0.000724505	0.048130937
cg11167615	-0.799512677	2.276947933	-3.892375082	0.000724509	0.048130937
cg01595666	-0.365054084	-0.438411019	-3.892367789	0.000724522	0.048130937
cg13303686	-0.522604496	3.628861668	-3.892366805	0.000724524	0.048130937
cg25412677	-0.462026768	2.258292635	-3.892232965	0.000724765	0.048130937
cg16930925	-0.575768004	1.618741676	-3.892210289	0.000724806	0.048130937
cg07626798	-0.528896106	3.597415197	-3.892166086	0.000724886	0.048130937
cg09813610	-0.886002775	-4.228034262	-3.892144622	0.000724924	0.048130937
cg01048107	-0.52282425	-1.758410815	-3.892141638	0.00072493	0.048130937
cg03973379	-0.581744867	3.033777951	-3.892137867	0.000724937	0.048130937

cg22979314	-0.677531408	2.857027757	-3.892126997	0.000724956	0.048130937
cg01465009	-0.549545406	3.387096804	-3.892041855	0.00072511	0.048130937
cg17139861	-0.558753781	2.830636392	-3.892019942	0.000725149	0.048130937
cg15778925	-0.428029793	-0.685976987	-3.892006463	0.000725173	0.048130937
cg21390684	-0.392170799	3.888427848	-3.891914605	0.000725339	0.048130937
cg21748464	-0.378645619	3.409871758	-3.891908862	0.000725349	0.048130937
cg22144486	-0.544526018	3.547979171	-3.891906093	0.000725354	0.048130937
cg08921133	-0.509012269	3.751586536	-3.891859717	0.000725438	0.048130937
cg24993140	-0.455377096	3.709095905	-3.891842771	0.000725469	0.048130937
cg23063913	-0.436597085	-2.569804739	-3.891816156	0.000725517	0.048130937
cg24412895	-0.407208143	0.813518602	-3.891779641	0.000725582	0.048130937
cg13526596	-0.499225392	3.731855029	-3.891673571	0.000725774	0.048133366
cg10295790	0.50774752	2.513043796	3.891656535	0.000725805	0.048133366
cg01543670	-0.51359456	3.403244088	-3.891634766	0.000725844	0.048133366
cg09768011	-0.446309051	4.161389791	-3.891507834	0.000726073	0.048133366
cg21830726	-0.449752721	3.955203925	-3.891500116	0.000726087	0.048133366
cg19805435	-0.437343853	-0.752653948	-3.891491822	0.000726102	0.048133366
cg02281699	-0.362397526	3.913465658	-3.891466891	0.000726147	0.048133366
cg21833932	-0.541606311	3.595461934	-3.891455161	0.000726168	0.048133366
cg09926562	-0.693167433	2.791577623	-3.891374546	0.000726314	0.048133366
cg05064054	-0.504309598	3.506662577	-3.891286275	0.000726473	0.048133366
cg27211071	-0.756169851	-1.2170503	-3.891249105	0.00072654	0.048133366
cg00900231	-0.569576663	-1.285699897	-3.891248019	0.000726542	0.048133366
cg14376653	-0.514346934	-0.126613648	-3.891148983	0.000726721	0.048133366
cg10890518	0.479124521	-3.161892015	3.891107637	0.000726796	0.048133366
cg07181000	-0.485091316	3.745840011	-3.891051385	0.000726897	0.048133366
cg12349875	0.879123058	-4.49963301	3.89104261	0.000726913	0.048133366
cg10778770	-0.578437419	3.388104796	-3.891036357	0.000726924	0.048133366
cg22290704	-0.464343673	2.948548268	-3.891029777	0.000726936	0.048133366
cg24626843	0.372720784	1.420231566	3.891008344	0.000726975	0.048133366
cg10113589	-0.407536426	-0.079845885	-3.891001985	0.000726987	0.048133366
cg04828658	-0.504318634	1.654198462	-3.890969143	0.000727046	0.048133366
cg16185523	-0.816081228	2.399258339	-3.890847462	0.000727266	0.048142967
cg11327194	-0.425869581	0.392253485	-3.890813741	0.000727327	0.048142967
cg06142662	-0.412377437	3.959507625	-3.890703508	0.000727526	0.048151663
cg13459077	-0.615659476	2.640698949	-3.890610201	0.000727695	0.048154067
cg03244650	-0.799981022	2.65792418	-3.890581864	0.000727746	0.048154067
cg01419164	-0.63188188	4.581141253	-3.890570691	0.000727767	0.048154067
cg12610030	0.639319778	1.729609496	3.89050507	0.000727885	0.048154074
cg23545250	-0.585723365	-2.657412756	-3.89044195	0.000728	0.048154074
cg06586469	-0.57328644	2.564992192	-3.890396467	0.000728082	0.048154074
cg04652932	-0.560872854	-0.988673044	-3.890320202	0.00072822	0.048154074
ch.2.1149235F	-0.680680433	-2.110524549	-3.890262824	0.000728324	0.048154074
cg23991388	-0.538900134	-0.753703335	-3.890201822	0.000728434	0.048154074
cg09712197	-0.329789233	3.767774634	-3.890139345	0.000728547	0.048154074
cg12113345	-0.497465095	3.006281211	-3.889987418	0.000728823	0.048154074
cg12781374	-0.533323951	3.039797484	-3.88995594	0.00072888	0.048154074

cg00231192	0.358827402	1.3466203	3.889925314	0.000728935	0.048154074
cg22547480	-0.428988499	3.047236046	-3.889903706	0.000728974	0.048154074
cg07245447	-0.458036708	0.775776874	-3.889891103	0.000728997	0.048154074
cg03865600	-0.497566898	-0.454639513	-3.889879131	0.000729019	0.048154074
cg17666994	-0.592126405	3.743844224	-3.88985822	0.000729057	0.048154074
cg01445411	-0.428630774	3.179509088	-3.889851304	0.000729069	0.048154074
cg27213210	-0.45375674	2.667173259	-3.88978668	0.000729186	0.048154074
cg11854729	-0.865847184	1.85313779	-3.889701106	0.000729342	0.048154074
cg26824216	-0.517584324	-2.282907364	-3.889661306	0.000729414	0.048154074
cg11390344	-0.433468287	3.7153977	-3.889533431	0.000729646	0.048154074
cg01577070	-0.688505727	2.488711622	-3.889527892	0.000729656	0.048154074
cg06780308	-0.40066741	3.751972709	-3.889491616	0.000729721	0.048154074
cg12475934	-0.607610154	2.598489857	-3.889423712	0.000729845	0.048154074
cg10947584	-0.442269568	1.410720325	-3.889417029	0.000729857	0.048154074
cg05463727	0.653413119	3.292994117	3.889363474	0.000729954	0.048154074
cg00098040	-0.574954143	2.264386382	-3.889332927	0.000730009	0.048154074
cg20215494	-0.409115703	-1.352484158	-3.889300078	0.000730069	0.048154074
cg08941098	-0.494499155	3.162426525	-3.889260838	0.00073014	0.048154074
cg18008653	-0.847363701	2.640061585	-3.889259933	0.000730142	0.048154074
cg19359439	-0.400018763	4.232899846	-3.889194944	0.00073026	0.048154074
cg06133462	-0.630368622	2.689673454	-3.889181909	0.000730284	0.048154074
cg22613262	-0.411871635	3.678012258	-3.889154651	0.000730333	0.048154074
cg10125428	-0.704185482	3.297095432	-3.889086098	0.000730457	0.048154074
cg16300707	-0.405533249	4.206516276	-3.889062021	0.000730501	0.048154074
cg21875190	-0.424106525	3.874939686	-3.889049223	0.000730524	0.048154074
cg09822052	-0.536268663	1.206187333	-3.889033984	0.000730552	0.048154074
cg01204128	-0.533280412	0.457879327	-3.889021411	0.000730575	0.048154074
cg20600038	-0.789131247	1.393165008	-3.889015149	0.000730586	0.048154074
cg00594099	-0.432858414	-1.580822163	-3.888947606	0.000730709	0.048154074
cg26200734	-0.693631825	2.53143159	-3.888923538	0.000730753	0.048154074
cg17821645	-0.415795392	-0.123416638	-3.888918989	0.000730761	0.048154074
cg26163470	-0.762301272	-0.643640007	-3.888897607	0.0007308	0.048154074
cg11723270	-0.561093745	3.627195538	-3.888875679	0.00073084	0.048154074
cg21461805	-0.445493814	3.566253608	-3.88887364	0.000730843	0.048154074
cg23492834	-0.460219388	3.648436803	-3.888870679	0.000730849	0.048154074
cg11001670	-0.512860786	3.43199895	-3.888867559	0.000730854	0.048154074
cg12958046	-0.520258098	3.353130033	-3.888825849	0.00073093	0.048154074
cg11533291	-0.466088035	0.302424148	-3.888808587	0.000730962	0.048154074
cg04980297	-0.581793526	2.194874667	-3.888740178	0.000731086	0.048157786
cg24397939	-0.65314018	2.480342604	-3.888633646	0.00073128	0.048166062
cg09565111	-0.679594788	0.305734957	-3.888519167	0.000731488	0.04816818
cg10671125	-0.880780461	1.887682101	-3.88849979	0.000731523	0.04816818
cg24412662	-0.512750348	-0.240145808	-3.888497358	0.000731527	0.04816818
cg08721652	-0.427849867	3.978879819	-3.888466378	0.000731584	0.04816818
cg07704716	-0.383278348	-2.265458426	-3.888401888	0.000731701	0.048171425
cg22760690	-0.500121718	-0.115258747	-3.888310964	0.000731866	0.048177837
cg23361916	-0.536708195	3.467528814	-3.888222257	0.000732028	0.048178941

cg04181478	-0.559996334	2.854534629	-3.888186264	0.000732093	0.048178941
cg20071505	0.542347642	3.465177839	3.888110826	0.000732231	0.048178941
cg09869357	-0.608848173	3.222058945	-3.888107442	0.000732237	0.048178941
cg05124073	-0.382997108	2.556269832	-3.888105714	0.00073224	0.048178941
cg26032513	-0.558889724	0.402409058	-3.888070875	0.000732303	0.048178941
cg19766369	-0.811898247	0.00289206	-3.888040162	0.000732359	0.048178941
cg06916270	-0.485272013	-0.130852659	-3.8879574	0.00073251	0.048184379
cg00720106	-0.605750892	3.257933712	-3.887902365	0.00073261	0.048184569
cg25121146	-0.423491908	3.505829219	-3.887863922	0.00073268	0.048184569
cg17321617	-0.658923873	-0.867173373	-3.887843767	0.000732717	0.048184569
cg17791030	-0.62338115	2.783014116	-3.887710982	0.000732959	0.048188354
cg26547928	-0.547738297	3.250859326	-3.887702922	0.000732973	0.048188354
cg11895696	-0.500528504	-1.066135841	-3.887700146	0.000732979	0.048188354
cg25335435	-0.436016794	4.056185396	-3.887644482	0.00073308	0.048188703
cg01179137	-0.413233974	0.003050568	-3.887541373	0.000733268	0.048188703
cg05932024	-0.565901349	3.164029506	-3.88752505	0.000733298	0.048188703
cg02650744	-0.45193645	3.40422111	-3.887523903	0.0007333	0.048188703
cg15622655	-0.598867612	2.469608877	-3.887453417	0.000733428	0.048188703
cg16575175	-0.465971998	-0.169273715	-3.887445472	0.000733443	0.048188703
cg11075099	-0.401753178	3.941484847	-3.887414455	0.000733499	0.048188703
cg03178468	-0.700427686	2.350783824	-3.887329128	0.000733655	0.048188703
cg21948803	-0.633332485	1.321536717	-3.887262089	0.000733777	0.048188703
cg12385975	-0.39853423	3.939443394	-3.887250081	0.000733799	0.048188703
cg08388182	-0.508656131	-0.890168328	-3.887241589	0.000733815	0.048188703
cg17492940	-0.419364327	4.044660381	-3.88715576	0.000733971	0.048188703
cg09821991	0.568285573	-4.260039055	3.887113588	0.000734048	0.048188703
cg14461266	-0.374524419	4.27660367	-3.887101891	0.000734069	0.048188703
cg08473660	-0.518642545	-0.678801234	-3.887095976	0.00073408	0.048188703
cg02035061	-0.417174264	3.342905684	-3.887073691	0.000734121	0.048188703
cg01882998	-0.510543923	3.152895592	-3.887063051	0.00073414	0.048188703
cg16501561	-0.599879027	-1.034029276	-3.88697506	0.000734301	0.04819155
cg00559439	-0.395659526	3.24360655	-3.886919519	0.000734402	0.04819155
cg21657781	-0.710718284	2.626394111	-3.88690348	0.000734432	0.04819155
cg19199512	-0.636314231	2.918512175	-3.88689021	0.000734456	0.04819155
cg08714967	-0.353221334	3.69852555	-3.886728167	0.000734752	0.048203871
cg10395657	-0.442077575	4.287363	-3.886678678	0.000734842	0.048203871
cg22949034	-0.489371728	3.556844276	-3.886675597	0.000734848	0.048203871
cg04263658	-0.798196844	1.815318215	-3.886636965	0.000734918	0.048204037
cg11425279	-0.778296568	2.270821101	-3.886561104	0.000735057	0.048204088
ch.12.2406115F	0.67246023	-2.848999603	3.886546285	0.000735084	0.048204088
cg01727128	-0.884259212	1.817260643	-3.886476643	0.000735211	0.048204088
cg22308877	-0.66679253	-0.548873588	-3.886464866	0.000735233	0.048204088
cg16185996	-0.406694794	-0.404034133	-3.886450336	0.000735259	0.048204088
cg20056648	-0.532181608	3.446113648	-3.886402667	0.000735346	0.048205339
cg17586094	-0.454265643	-2.45106463	-3.886311512	0.000735513	0.048211801
cg17363943	-0.470111972	-0.653171816	-3.88626645	0.000735595	0.048212701
cg17230724	-0.37563628	0.493051955	-3.88622956	0.000735663	0.048212701

cg15247955	-0.592325209	3.068895732	-3.886084269	0.000735929	0.048225655
cg06026375	-0.494102929	3.86221804	-3.885944473	0.000736184	0.048237954
cg27632435	-0.504046106	-3.904509555	-3.885771354	0.000736501	0.048254253
cg14061255	-0.561017285	3.305407425	-3.885730351	0.000736576	0.048254708
cg14155724	-0.51470859	3.051063134	-3.885612436	0.000736792	0.04826439
cg20249492	-0.594312228	-1.286128062	-3.885568315	0.000736873	0.048265221
cg05241157	-0.333787726	-1.308363741	-3.885470568	0.000737052	0.048272292
cg23104549	-0.448484295	3.509539518	-3.885429913	0.000737127	0.048272292
cg25820188	-0.376692858	-0.878725583	-3.885397801	0.000737185	0.048272292
cg13126336	-0.504901933	3.051087905	-3.88535449	0.000737265	0.048273027
cg23290350	-0.387707326	3.622629352	-3.885302619	0.00073736	0.048274239
cg17439237	-0.583363196	3.178172803	-3.88527002	0.00073742	0.048274239
cg12349241	-0.792629522	1.574500174	-3.88500425	0.000737907	0.048300623
cg02885259	-0.610897874	2.609962727	-3.884946163	0.000738014	0.048300623
cg11368427	-0.733936296	2.408964358	-3.884906186	0.000738087	0.048300623
cg07937810	-0.795772698	1.150258848	-3.884901521	0.000738095	0.048300623
cg07882678	0.627339343	2.981754572	3.884820131	0.000738245	0.048302402
cg14168996	-0.487785954	2.485786108	-3.884791124	0.000738298	0.048302402
cg26461267	0.661442045	1.386956848	3.884775218	0.000738327	0.048302402
cg12783942	-0.622546491	2.850130822	-3.884659161	0.00073854	0.048311877
cg20785459	-0.34817538	4.127938436	-3.88444111	0.000738941	0.048331428
cg18750643	-0.447142522	4.317928742	-3.884403143	0.00073901	0.048331428
cg02706294	-0.501299243	3.816904248	-3.884384942	0.000739044	0.048331428
cg24704200	-0.4602289	3.851695633	-3.884273242	0.000739249	0.048335939
cg14562983	0.608704201	2.942576036	3.884209246	0.000739367	0.048335939
cg09038666	-0.37970449	-0.521589776	-3.884197245	0.000739389	0.048335939
cg17518244	-0.462627285	-0.408059541	-3.884165362	0.000739447	0.048335939
cg16443053	-0.438484881	3.874667218	-3.884113064	0.000739543	0.048335939
cg21025032	-0.40868119	4.249218445	-3.884098069	0.000739571	0.048335939
cg01561157	0.795215533	2.808939371	3.88408749	0.00073959	0.048335939
cg25842688	-0.524633877	3.026112992	-3.884040694	0.000739676	0.048337102
cg05369810	-0.394613557	-0.915177974	-3.883994864	0.000739761	0.048338149
cg08873673	-0.506324531	2.960753138	-3.88394985	0.000739843	0.048339099
cg02318796	-0.430634568	4.246961678	-3.883874682	0.000739982	0.048339774
cg01021631	-0.576855428	-0.248540741	-3.88374531	0.00074022	0.048339774
cg22810049	-0.454931565	3.397591715	-3.883735904	0.000740237	0.048339774
cg02015219	-0.459902598	3.596723555	-3.883710261	0.000740284	0.048339774
cg26801697	-0.506560691	2.857939755	-3.883511059	0.000740651	0.048339774
cg07913430	-0.49520121	-0.903422384	-3.883491056	0.000740688	0.048339774
cg02736488	-0.591374803	2.8087666	-3.883456135	0.000740752	0.048339774
cg07198944	-0.42038553	0.142718382	-3.883400757	0.000740854	0.048339774
cg13581402	-0.491101853	3.77340208	-3.883348123	0.000740951	0.048339774
cg22006654	-0.447409999	2.881662423	-3.883302986	0.000741034	0.048339774
cg07531569	-0.616015038	3.546811681	-3.883243189	0.000741144	0.048339774
cg01700386	-0.489113884	3.744559	-3.883196659	0.00074123	0.048339774
cg07262001	-0.453656069	3.922229753	-3.883150919	0.000741314	0.048339774
cg22288309	-0.428064308	3.664379918	-3.88314521	0.000741325	0.048339774

cg19638921	-0.388964158	4.239521081	-3.883136434	0.000741341	0.048339774
cg22823462	-0.390371887	0.405574697	-3.883136253	0.000741341	0.048339774
cg08644039	-0.387397814	3.068703438	-3.883112138	0.000741386	0.048339774
cg05466464	-0.396981604	3.666138413	-3.88309076	0.000741425	0.048339774
cg01036371	-0.476769283	-1.570197091	-3.883055506	0.00074149	0.048339774
cg02153245	-0.40583072	-0.186664354	-3.883053916	0.000741493	0.048339774
cg22012333	-0.449105927	-1.620313641	-3.883040932	0.000741517	0.048339774
cg02854030	-0.395499849	-1.002265878	-3.883012108	0.00074157	0.048339774
cg04030141	-0.604091544	-0.881167156	-3.883005865	0.000741581	0.048339774
cg12903024	-0.408783012	0.142709344	-3.882985483	0.000741619	0.048339774
cg02885543	-0.534192081	3.369861315	-3.882980058	0.000741629	0.048339774
cg26544657	-0.393552768	3.164648762	-3.882926661	0.000741727	0.048339774
cg05380885	0.580496818	2.425113933	3.882898709	0.000741779	0.048339774
cg12723492	-0.467336886	-1.212070328	-3.882876651	0.00074182	0.048339774
cg00414536	-0.601287559	-0.069721092	-3.882869534	0.000741833	0.048339774
cg22980648	-0.418802539	4.196957052	-3.882741267	0.000742069	0.048345908
cg10017818	0.726166228	3.219516077	3.88272559	0.000742098	0.048345908
cg25282574	-0.5368812	3.624259163	-3.882697608	0.00074215	0.048345908
cg26367107	-0.448443426	-1.483424737	-3.882631721	0.000742271	0.048345908
cg25176380	-0.322414648	0.532674695	-3.882619905	0.000742293	0.048345908
cg16905911	-0.578349578	-1.018435091	-3.88259649	0.000742336	0.048345908
cg20263570	-0.684890319	2.748724647	-3.882397133	0.000742704	0.048361116
cg18883919	-0.432798583	0.108292415	-3.882364727	0.000742764	0.048361116
cg20720056	-0.460764352	4.024040838	-3.882333641	0.000742821	0.048361116
cg15013315	-0.381124715	-0.107618022	-3.88228322	0.000742915	0.048361116
cg06516465	-0.751750129	2.527213288	-3.882138078	0.000743183	0.048361116
cg08817867	-0.428493138	-0.559607295	-3.8820496	0.000743346	0.048361116
cg13598865	0.778180411	-5.007825104	3.882028461	0.000743385	0.048361116
cg09645743	-0.614698235	2.094714878	-3.882021254	0.000743398	0.048361116
cg03739280	-0.630106384	2.643970002	-3.88199324	0.00074345	0.048361116
cg01168584	-0.500743002	-1.607269214	-3.881986978	0.000743462	0.048361116
cg11556122	-0.539410481	3.496072113	-3.881965701	0.000743501	0.048361116
cg03358351	-0.553243169	3.279939669	-3.881907522	0.000743609	0.048361116
cg14332665	-0.501169155	3.399994368	-3.881891056	0.000743639	0.048361116
cg09232269	0.491082711	1.823440261	3.881850231	0.000743714	0.048361116
cg20516630	0.597634396	-3.570957354	3.881844557	0.000743725	0.048361116
cg07371628	-0.487748709	-0.527713297	-3.881764808	0.000743872	0.048361116
cg15737510	-0.554594766	3.402002952	-3.881748731	0.000743902	0.048361116
cg07962847	-0.487030202	3.529201377	-3.881740597	0.000743917	0.048361116
cg03985228	-0.531485194	-0.729093135	-3.881680071	0.000744029	0.048361116
cg05301965	-0.862692349	2.172956502	-3.881670187	0.000744047	0.048361116
cg18641851	-0.417665666	0.416936227	-3.881653225	0.000744079	0.048361116
cg05747535	-0.507421362	-0.311162027	-3.881627474	0.000744126	0.048361116
cg10419904	-0.494166922	3.457311457	-3.881620069	0.00074414	0.048361116
cg26571814	0.574523157	2.224120453	3.881576473	0.000744221	0.04836192
cg13274014	0.535543138	3.88125029	3.881435527	0.000744481	0.04836549
cg10587895	-0.674237181	2.508592263	-3.881413393	0.000744522	0.04836549

cg01384593	-0.523212235	3.599773746	-3.881404615	0.000744539	0.04836549
cg26557993	-0.679737756	1.580826903	-3.881399161	0.000744549	0.04836549
cg18277507	-0.363645571	0.365332087	-3.881182403	0.00074495	0.048385605
cg08857108	-0.622348649	3.063238022	-3.881158075	0.000744995	0.048385605
cg24200596	-0.478333325	2.601326485	-3.881088703	0.000745123	0.048389511
cg16027308	-0.53694386	3.84514584	-3.880973673	0.000745337	0.048397706
cg15337768	-0.498453139	3.0097408	-3.880946802	0.000745386	0.048397706
cg11986875	-0.784083909	1.608506431	-3.880854568	0.000745557	0.048403085
cg25545609	-0.400893152	0.357048803	-3.880798867	0.00074566	0.048403085
cg15076631	0.763501257	2.487790943	3.880791465	0.000745674	0.048403085
cg20584932	-0.472100337	4.521092783	-3.880708973	0.000745827	0.048403257
cg02676742	-0.602153282	2.902063047	-3.880677647	0.000745885	0.048403257
cg20604021	-0.44023025	3.775559151	-3.88064554	0.000745945	0.048403257
cg15273618	-0.506725521	-0.183457108	-3.880642582	0.00074595	0.048403257
cg02542999	-0.850173203	2.28920521	-3.880393706	0.000746412	0.048420009
cg00476790	0.578196197	2.602298164	3.880334113	0.000746522	0.048420009
cg08351473	0.533754678	1.834800984	3.880253586	0.000746672	0.048420009
cg01001800	-0.439965046	3.447638858	-3.880202931	0.000746766	0.048420009
cg17092532	-0.485205741	4.48873802	-3.880199842	0.000746771	0.048420009
cg17344640	-0.558672692	2.513316326	-3.880193807	0.000746783	0.048420009
cg05719472	-0.549894586	-0.148191106	-3.880172732	0.000746822	0.048420009
cg08912229	-0.456436069	3.129876205	-3.880163163	0.000746839	0.048420009
cg05088386	-0.55437709	3.255511831	-3.880019856	0.000747105	0.048420009
cg17391883	-0.64699333	2.479598975	-3.879972751	0.000747193	0.048420009
cg14797608	-0.526033336	3.490143771	-3.879953206	0.000747229	0.048420009
cg25650474	-0.502355882	3.862646764	-3.879917394	0.000747296	0.048420009
cg15583544	-0.463523992	-0.15611606	-3.879891542	0.000747344	0.048420009
cg07139363	-0.71885471	2.37538095	-3.879890798	0.000747345	0.048420009
cg08916769	-0.65824949	3.3522818	-3.879871564	0.000747381	0.048420009
cg15970584	-0.421138276	3.957231435	-3.879868713	0.000747386	0.048420009
cg03188843	-0.499045512	3.564036716	-3.879784169	0.000747543	0.048420009
cg15723346	-0.47642551	4.038379847	-3.879759634	0.000747589	0.048420009
cg06974053	-0.416459698	-0.613068055	-3.879734551	0.000747635	0.048420009
cg18272304	-0.503091512	-1.022354431	-3.879713437	0.000747675	0.048420009
cg04261164	-0.384305817	3.547530927	-3.879708584	0.000747684	0.048420009
cg21304617	-0.483866938	-0.522356288	-3.879664239	0.000747766	0.048420009
cg08654337	-0.602593845	1.439942962	-3.879656543	0.00074778	0.048420009
cg09477996	-0.38837095	3.169005154	-3.879559878	0.00074796	0.04842288
cg07749266	-0.928312989	-4.017845438	-3.879559127	0.000747961	0.04842288
cg00706240	0.425704466	2.787504865	3.879510835	0.000748051	0.048424267
cg16957502	-0.655409186	1.993207557	-3.879414097	0.000748231	0.048429567
cg27009815	-0.541294153	3.301535171	-3.879344851	0.00074836	0.048429567
cg08268813	-0.697121834	2.74240975	-3.879250883	0.000748535	0.048429567
cg03319889	-0.441959774	4.071561657	-3.879240296	0.000748554	0.048429567
cg05656939	-0.614281848	3.516812496	-3.879222896	0.000748587	0.048429567
cg00048764	-0.39999606	3.930209934	-3.879180913	0.000748665	0.048429567
cg10252804	0.657841327	2.39631972	3.879166694	0.000748691	0.048429567

cg08658287	-0.375578486	3.089624911	-3.879127104	0.000748765	0.048429567
cg05403127	-0.635559128	2.528218579	-3.87910213	0.000748811	0.048429567
cg21972567	-0.528441054	3.115938973	-3.879099264	0.000748817	0.048429567
cg27479021	-0.369529047	0.164759107	-3.878707427	0.000749546	0.048472325
cg00124133	-0.567067775	0.120056814	-3.878510565	0.000749913	0.048488496
cg15064667	-0.446499669	3.390850988	-3.878499744	0.000749933	0.048488496
cg02401589	-0.671250219	1.028631005	-3.878364786	0.000750185	0.048500335
cg18083049	-0.502117962	3.43521668	-3.878305369	0.000750296	0.048503071
cg08560531	-0.395625971	0.628808029	-3.878135103	0.000750613	0.048519171
cg14496654	-0.413088013	-1.408022068	-3.878018526	0.000750831	0.048528801
cg12747588	-0.708768429	1.735692494	-3.877961214	0.000750938	0.048529908
cg20375654	-0.435458923	0.609879756	-3.877830427	0.000751182	0.048529908
cg18078841	-0.622868678	-3.107408554	-3.87781149	0.000751217	0.048529908
cg21158513	-0.605694594	-1.686592326	-3.877742584	0.000751346	0.048529908
cg04821942	-0.650029374	-0.864151076	-3.877735051	0.00075136	0.048529908
cg24070673	-0.564134418	3.147725119	-3.877659836	0.0007515	0.048529908
cg04315069	-0.441015179	-0.184403472	-3.877619283	0.000751576	0.048529908
cg25680215	-0.73208993	3.249817918	-3.877581036	0.000751647	0.048529908
cg11990702	0.699200866	4.231665988	3.877572911	0.000751663	0.048529908
cg23127450	-0.39219601	3.660291592	-3.877556782	0.000751693	0.048529908
cg10622931	-0.91583845	1.773430672	-3.877538267	0.000751727	0.048529908
cg21855225	-0.376542604	3.880589186	-3.877535091	0.000751733	0.048529908
cg00936790	-0.686942753	-2.573365684	-3.877532337	0.000751738	0.048529908
cg14821349	-0.427361628	1.221502881	-3.877411267	0.000751965	0.048533389
cg17474601	-0.597296148	1.807197011	-3.877342143	0.000752094	0.048533389
cg26526503	-0.413899547	0.299637052	-3.877312552	0.000752149	0.048533389
cg13963210	-0.524899237	0.696577425	-3.877276204	0.000752217	0.048533389
cg11555708	-0.534420389	2.462581429	-3.877223683	0.000752315	0.048533389
cg17844840	-0.409884669	-0.091980365	-3.877184404	0.000752389	0.048533389
cg22934824	-0.511756554	1.648287968	-3.87718398	0.000752389	0.048533389
cg00625456	-0.562636076	1.923793672	-3.877170379	0.000752415	0.048533389
cg15655935	-0.541737656	3.276090547	-3.877155805	0.000752442	0.048533389
cg19083479	-0.445588247	3.993287012	-3.877136927	0.000752477	0.048533389
cg12536527	-0.453965428	2.874720868	-3.876970526	0.000752789	0.048549042
cg20007321	0.338673057	1.160335552	3.876928527	0.000752867	0.048549689
cg19841896	-0.575228049	2.6675219	-3.876839895	0.000753033	0.048553346
cg16520685	-0.48893933	2.973546983	-3.876824964	0.000753061	0.048553346
cg20033239	-0.526376452	3.870900766	-3.876723955	0.00075325	0.048559969
cg17376432	-0.506969396	3.34952688	-3.876696831	0.000753301	0.048559969
cg17324872	-0.4453592	3.164578085	-3.876588422	0.000753504	0.048562307
cg02796390	-0.333515172	0.133747683	-3.876515885	0.00075364	0.048562307
cg24726768	0.599548584	-4.696029087	3.876497084	0.000753675	0.048562307
cg16717793	-0.806550417	1.235765952	-3.876495908	0.000753677	0.048562307
cg15708928	-0.607715425	2.7728946	-3.8764944	0.00075368	0.048562307
cg01642152	-0.457850817	-0.737336994	-3.876415944	0.000753827	0.04856509
cg19580003	-0.397991356	3.986149498	-3.87639814	0.00075386	0.04856509
cg06169771	-0.342878246	-1.215081635	-3.876338082	0.000753973	0.048567922

cg21351028	-0.414923698	4.347135003	-3.876151046	0.000754323	0.048582808
cg09418771	-0.433494229	-1.297919318	-3.876050449	0.000754512	0.048582808
cg16188466	-0.437980397	-0.3777542	-3.875930404	0.000754737	0.048582808
cg06875331	-0.603732864	3.594071401	-3.875895876	0.000754802	0.048582808
cg14479617	-0.532867961	3.07884283	-3.87588303	0.000754826	0.048582808
cg15424789	-0.434724876	3.987441882	-3.875867586	0.000754855	0.048582808
cg08683522	-0.35222379	3.435853741	-3.875860865	0.000754867	0.048582808
cg21449015	-0.556741634	2.704955896	-3.875796395	0.000754988	0.048582808
cg02611031	-0.446431453	-4.174662931	-3.87578199	0.000755015	0.048582808
cg16029875	-0.380410853	-1.702019123	-3.875707312	0.000755155	0.048582808
cg03619640	-0.579374712	3.468876637	-3.875679051	0.000755208	0.048582808
cg20964423	-0.383455271	-1.926618258	-3.875635399	0.00075529	0.048582808
cg08244485	-0.658410904	2.25928373	-3.87558309	0.000755389	0.048582808
cg10472962	-0.438294144	3.439092896	-3.87558187	0.000755391	0.048582808
cg17898737	-0.686786086	2.048430582	-3.875570603	0.000755412	0.048582808
cg18625270	-0.440079885	3.918067772	-3.875517901	0.000755511	0.048582808
cg10294164	-0.586944992	3.006679233	-3.875437552	0.000755662	0.048582808
cg22982811	-0.478415102	-0.464847244	-3.875215267	0.000756079	0.048582808
cg21600784	-0.443434642	3.964344591	-3.875188449	0.00075613	0.048582808
cg12306343	-0.321148199	4.065304289	-3.875156572	0.00075619	0.048582808
cg09787089	-0.66199764	-2.48230916	-3.875133708	0.000756233	0.048582808
cg24721309	-0.542795347	2.484718381	-3.875088407	0.000756318	0.048582808
cg25329933	-0.467272572	3.563110051	-3.875082576	0.000756329	0.048582808
cg18192614	-0.413214343	0.378545152	-3.87503583	0.000756417	0.048582808
cg14475381	-0.563678914	2.995831631	-3.875008214	0.000756468	0.048582808
cg19034208	-0.790131131	1.832990463	-3.874983638	0.000756515	0.048582808
cg08085567	-0.59435528	3.78190685	-3.874971951	0.000756537	0.048582808
cg23528105	-0.493115585	2.903389056	-3.874961041	0.000756557	0.048582808
cg12264764	-0.524020683	2.796746613	-3.874940554	0.000756596	0.048582808
cg07273304	0.643317517	3.39585078	3.87490163	0.000756669	0.048582808
cg07581623	-0.573898451	-1.011122795	-3.874897234	0.000756677	0.048582808
cg25043996	-0.469548425	3.759028483	-3.874860985	0.000756745	0.048582808
cg24927211	-0.46538404	3.705259	-3.87484729	0.000756771	0.048582808
cg12892054	-0.453727802	3.557023618	-3.874816048	0.00075683	0.048582808
cg18812058	-0.782437179	1.427354529	-3.874814687	0.000756832	0.048582808
cg02958515	-0.425644322	3.035806357	-3.874810114	0.000756841	0.048582808
cg05056722	-0.547711854	3.620756348	-3.874765629	0.000756925	0.048582808
cg26412531	-0.455547858	0.49034351	-3.874733758	0.000756985	0.048582808
cg20390814	-0.487773845	3.87481334	-3.874702965	0.000757042	0.048582808
cg22955140	-0.432284551	3.428507594	-3.874679086	0.000757087	0.048582808
cg13446288	-0.89805084	-2.791101788	-3.874614242	0.000757209	0.048582808
cg09027156	-0.461861658	3.788989924	-3.874502769	0.000757419	0.048582808
cg10372921	-0.439270593	-1.555022287	-3.874425054	0.000757565	0.048582808
cg20221991	-0.460361374	3.407364155	-3.874414074	0.000757586	0.048582808
cg00483533	-0.607838929	3.385613014	-3.874403904	0.000757605	0.048582808
cg03327816	-0.561085419	0.601634947	-3.874350027	0.000757707	0.048582808
cg15972185	-0.479497595	3.55162091	-3.874336673	0.000757732	0.048582808

cg00972588	-0.496788378	3.550114775	-3.874330092	0.000757744	0.048582808
cg01925002	-0.53066527	2.502594225	-3.874323682	0.000757756	0.048582808
cg16247544	-0.639325718	2.986110981	-3.874297299	0.000757806	0.048582808
cg13024275	-0.631133726	2.363126574	-3.874278932	0.000757841	0.048582808
cg22187085	-0.66859253	-0.433348153	-3.874266224	0.000757864	0.048582808
cg11428818	-0.825990774	2.715631208	-3.874263693	0.000757869	0.048582808
cg05077519	-0.484282517	2.781376007	-3.874243522	0.000757907	0.048582808
cg11465897	-0.454477866	3.971117857	-3.874163214	0.000758059	0.048588107
cg03491687	-0.697010265	-2.506686551	-3.874030106	0.000758309	0.048599783
cg19859759	-0.492277245	0.393871993	-3.873968579	0.000758425	0.048602817
cg08921397	-0.534407472	3.374468827	-3.873925623	0.000758506	0.048603608
cg03627985	-0.713465261	-1.378925476	-3.873879152	0.000758594	0.048604824
cg17224754	-0.634783191	3.40263056	-3.873760029	0.000758818	0.048614816
cg21074003	-0.437546201	0.603215785	-3.873719887	0.000758894	0.048615269
cg11995328	-0.765922934	2.213094525	-3.873650846	0.000759024	0.048616702
cg27033920	-0.406083031	4.055994129	-3.873572024	0.000759173	0.048616702
cg05819240	-0.429980328	0.46258932	-3.873569598	0.000759177	0.048616702
cg00958342	-0.515936712	-0.598279253	-3.873525783	0.00075926	0.048616702
cg21095779	-0.436737036	3.307661439	-3.873504029	0.000759301	0.048616702
cg24546263	-0.639498263	-0.956844356	-3.873489713	0.000759328	0.048616702
cg14765881	-0.454208666	-0.576623632	-3.87340526	0.000759488	0.048622511
cg25953930	-0.53527494	-1.382321447	-3.873364581	0.000759564	0.048623032
cg17070935	-0.440106196	3.185478291	-3.873269953	0.000759743	0.048629909
cg11958644	-0.435391816	-0.215361411	-3.873234936	0.000759809	0.048629909
cg03694857	-0.520294596	3.877280665	-3.873054777	0.000760149	0.048643459
cg03961030	-0.342767601	-0.609853686	-3.872995353	0.000760262	0.048643459
cg25512593	-0.423102013	4.006613214	-3.872992076	0.000760268	0.048643459
cg19893032	-0.402688351	3.929164453	-3.872969801	0.00076031	0.048643459
cg22928497	-0.455228327	-0.994208849	-3.872937163	0.000760371	0.048643459
cg25497545	-0.661574017	2.005183278	-3.872904704	0.000760433	0.048643459
cg06764498	-0.434824155	3.266754868	-3.87275699	0.000760712	0.048656922
cg22982702	-0.497740264	3.500802641	-3.872607409	0.000760995	0.048664368
cg16521454	-0.528768666	-1.646547606	-3.872584808	0.000761037	0.048664368
cg08956208	0.73019194	2.206408858	3.872503109	0.000761192	0.048664368
cg21724450	-0.360365231	0.549131063	-3.872482516	0.000761231	0.048664368
cg24235862	-0.515172295	3.448320515	-3.872479788	0.000761236	0.048664368
cg04036282	-0.420800047	4.220491638	-3.87247743	0.000761241	0.048664368
cg25214376	-0.470321106	-1.23221772	-3.872433681	0.000761323	0.048665266
cg22895640	-0.388818388	3.730897327	-3.87238963	0.000761407	0.048666202
cg22297466	-0.485344361	3.364535995	-3.87229301	0.000761589	0.048668385
cg21250594	0.418001036	2.741316085	3.872281039	0.000761612	0.048668385
cg21514071	-0.51802994	3.399295626	-3.872262648	0.000761647	0.048668385
cg10494894	-0.872717386	-4.090931902	-3.872219692	0.000761728	0.048668623
cg08862452	-0.503858163	1.111475875	-3.872188083	0.000761788	0.048668623
cg13718960	-0.428707825	-0.726393615	-3.872127941	0.000761902	0.048671372
cg08848887	-0.49067283	0.428960149	-3.872092762	0.000761968	0.048671372
cg14676177	-0.701469959	2.465536422	-3.871916544	0.000762302	0.048688102

cg04351597	-0.356987241	-0.356954161	-3.871881869	0.000762368	0.048688102
cg10073280	-0.623735486	2.837898789	-3.871754911	0.000762608	0.048696766
cg22704788	-0.486534041	-1.540965229	-3.871732773	0.00076265	0.048696766
cg11653645	-0.412469143	-0.798477278	-3.871661103	0.000762786	0.048696766
cg00278090	0.407568205	1.958699642	3.8716399	0.000762826	0.048696766
cg24640583	-0.466393634	0.47690492	-3.871628875	0.000762847	0.048696766
cg09720597	-0.68753772	2.759233791	-3.871575004	0.000762949	0.048698896
cg13811867	-0.693557494	0.072105642	-3.871474264	0.00076314	0.048706699
cg19959091	-0.549094295	3.425782857	-3.87139381	0.000763293	0.048711546
cg02463143	-0.411893527	3.467863867	-3.871361692	0.000763354	0.048711546
cg02904800	-0.417482808	0.256165781	-3.871288317	0.000763493	0.048716039
cg19835472	-0.466287573	3.165447495	-3.871229796	0.000763604	0.048718735
cg22788243	-0.716740606	1.016241312	-3.871119475	0.000763813	0.048727702
cg27292074	-0.522944127	3.155460314	-3.870997842	0.000764044	0.048738041
cg08494871	-0.426819625	-1.459609006	-3.870931911	0.000764169	0.048738404
cg22580380	-0.498220987	3.303627098	-3.87092237	0.000764188	0.048738404
cg22718891	-0.735098658	-1.352707984	-3.8707792	0.000764459	0.048750575
cg03035890	-0.569684772	2.874698416	-3.870749404	0.000764516	0.048750575
cg21420351	-0.387118181	-1.034195328	-3.870702859	0.000764604	0.048750589
cg25279093	-0.44614113	3.434679516	-3.870663633	0.000764679	0.048750589
cg10538433	-0.403248027	4.348511604	-3.870640619	0.000764723	0.048750589
cg03945057	-0.425631536	3.268279405	-3.870507477	0.000764976	0.04876233
cg16773813	-0.401909344	4.50987671	-3.870440205	0.000765104	0.048766093
cg15721529	-0.664799644	1.326683104	-3.870357707	0.00076526	0.048766439
cg09533142	0.422096836	1.66597814	3.870338309	0.000765297	0.048766439
cg05267156	-0.773063504	1.758893928	-3.870308478	0.000765354	0.048766439
cg24582377	0.334718951	-0.596890305	3.870260414	0.000765445	0.048766439
cg18134619	-0.72110048	3.166616834	-3.87025632	0.000765453	0.048766439
cg15735620	-0.708134073	-0.620215758	-3.87015932	0.000765638	0.048773806
cg23493020	0.521386278	1.649643617	3.869888478	0.000766153	0.048802249
cg19764731	-0.340003249	-0.124866196	-3.869807037	0.000766308	0.048807736
cg01644962	-0.593843269	0.336485083	-3.869622562	0.000766659	0.048822784
cg14019501	-0.397999799	-0.077222958	-3.869610597	0.000766682	0.048822784
cg06465045	0.48609091	1.935754791	3.869563349	0.000766772	0.048824126
cg26465327	-0.424366176	0.17837953	-3.869470801	0.000766949	0.048828956
cg22301418	-0.464706374	-0.100251774	-3.869431128	0.000767024	0.048828956
cg12098680	-0.70269274	2.321132642	-3.869415016	0.000767055	0.048828956
cg07421887	-0.537816029	3.677003648	-3.869338415	0.000767201	0.048833861
cg18460830	-0.413118392	-0.061335075	-3.869290527	0.000767292	0.048834119
cg13940957	-0.528855516	3.632663465	-3.869263963	0.000767343	0.048834119
cg08209422	-0.43987616	-1.080674536	-3.869031907	0.000767785	0.048851942
cg06324869	-0.682622617	0.516043059	-3.869026068	0.000767796	0.048851942
cg01870690	-0.465818637	-1.634476364	-3.868980954	0.000767883	0.048851942
cg12347442	-0.674484584	0.719636963	-3.868972477	0.000767899	0.048851942
cg07149190	0.643427915	2.938999947	3.868751105	0.000768321	0.048874428
cg00432058	-0.416684734	4.274236706	-3.868661498	0.000768492	0.048874479
cg01061789	-0.538553867	3.433278181	-3.868618899	0.000768574	0.048874479

cg18650693	-0.461176184	3.430530217	-3.868585807	0.000768637	0.048874479
cg10444843	-0.533439736	3.288811651	-3.868568178	0.00076867	0.048874479
cg10671510	-0.473360979	-1.378039442	-3.868566173	0.000768674	0.048874479
cg01021196	-0.531713366	0.982784749	-3.868481967	0.000768835	0.048874479
cg05561696	-0.438633101	-0.480364156	-3.868440305	0.000768915	0.048874479
cg00040659	-0.433934614	-0.516739409	-3.868426234	0.000768942	0.048874479
cg02764523	-0.502694217	-0.23698661	-3.868425516	0.000768943	0.048874479
cg18228109	-0.568740572	2.877252935	-3.868380124	0.00076903	0.048875382
cg10364017	-0.739618788	0.973428148	-3.868295118	0.000769192	0.048875382
cg07341813	-0.532335635	-0.286256897	-3.868227917	0.00076932	0.048875382
cg03725137	-0.488973947	3.984545825	-3.86821724	0.000769341	0.048875382
cg06612660	-0.364895667	3.109721493	-3.868205981	0.000769362	0.048875382
cg13564717	-0.817689032	1.333320057	-3.868164322	0.000769442	0.048875382
cg10876863	-0.566243304	1.175468038	-3.868136196	0.000769496	0.048875382
cg06943561	-0.682017455	-1.037628523	-3.868129256	0.000769509	0.048875382
cg24834791	-0.552190014	0.685575034	-3.868075372	0.000769612	0.048876801
cg25162729	-0.50373921	2.75842467	-3.868031857	0.000769695	0.048876801
cg13078598	-0.817220285	0.960337708	-3.86800931	0.000769738	0.048876801
cg19180698	-0.411114068	-0.036603494	-3.867894497	0.000769958	0.048886363
cg13477226	-0.613620582	3.039935073	-3.86778252	0.000770172	0.048892693
cg09499699	-0.920951569	1.717717616	-3.867770232	0.000770196	0.048892693
cg09413976	-0.74305154	1.909755267	-3.867589873	0.000770541	0.048904503
cg15814750	-0.461101128	3.840298189	-3.867577373	0.000770565	0.048904503
cg08339915	-0.472735581	-0.028079656	-3.867472109	0.000770766	0.048904503
cg23774361	-0.541143474	3.018806836	-3.867430715	0.000770846	0.048904503
cg10652359	-0.516311655	3.444474276	-3.867429909	0.000770847	0.048904503
cg14914706	-0.523619419	3.133256868	-3.867428963	0.000770849	0.048904503
cg07617759	-0.68015971	-0.792382212	-3.867418057	0.00077087	0.048904503
cg17604181	-0.424375581	3.4835797	-3.867321058	0.000771056	0.048904503
cg17333830	-0.630812201	2.827976007	-3.867209433	0.00077127	0.048904503
cg05195377	-0.568057168	2.193577571	-3.867132516	0.000771417	0.048904503
cg00183770	-0.856932646	2.316953243	-3.867116139	0.000771448	0.048904503
cg09856074	-0.577865266	-0.716355525	-3.867104741	0.00077147	0.048904503
cg08179368	-0.557414049	2.509374454	-3.867097524	0.000771484	0.048904503
cg20429911	0.560835672	-3.410993515	3.867054271	0.000771567	0.048904503
cg03490406	0.629876344	2.731559274	3.866949239	0.000771768	0.048904503
cg01829901	-0.446489925	-0.122880692	-3.866921601	0.000771821	0.048904503
cg25100883	-0.458395922	-1.555279237	-3.866915722	0.000771833	0.048904503
cg08201646	0.428595547	2.617233535	3.866906964	0.000771849	0.048904503
cg15460050	-0.526746398	2.956069712	-3.866904653	0.000771854	0.048904503
cg27306581	0.544988279	3.654004014	3.866882127	0.000771897	0.048904503
cg07305485	-0.475812457	3.222733647	-3.866814479	0.000772027	0.048904503
cg15191197	-0.456022313	3.486741918	-3.866786448	0.00077208	0.048904503
cg22900387	-0.455881005	3.459315312	-3.866764917	0.000772122	0.048904503
cg04305183	-0.4900587	3.942583707	-3.866717803	0.000772212	0.048904503
cg18130079	-0.395943835	4.376506194	-3.866649146	0.000772344	0.048904503
cg00063284	0.597499087	1.941422232	3.866646599	0.000772349	0.048904503

cg06305026	-0.453366576	3.739368094	-3.866627185	0.000772386	0.048904503
cg00776173	-0.488495825	3.166169482	-3.866626919	0.000772386	0.048904503
cg23618588	-0.793385521	2.487909226	-3.86650658	0.000772617	0.048904503
cg26808675	-0.495602361	3.354300214	-3.866487227	0.000772655	0.048904503
cg11062835	-0.393470387	-1.26733324	-3.866477954	0.000772672	0.048904503
cg10107839	-0.442856157	0.084716122	-3.866474521	0.000772679	0.048904503
cg12316667	-0.357038328	-0.125927098	-3.866463141	0.000772701	0.048904503
cg12003952	-0.568571501	3.217046375	-3.86644844	0.000772729	0.048904503
cg22643481	-0.587645652	3.516782547	-3.866293766	0.000773026	0.048918928
cg05203346	-0.402392603	3.975582741	-3.866192282	0.000773221	0.048926146
cg10320516	-0.648156617	0.1837531	-3.866113935	0.000773371	0.048926146
cg15337956	-0.560303437	1.004423162	-3.866000991	0.000773588	0.048926146
cg14625947	-0.524996407	-1.885471482	-3.865974513	0.000773639	0.048926146
cg26358141	-0.434926735	3.085759812	-3.865934807	0.000773716	0.048926146
cg04777383	-0.464283207	-1.626261353	-3.865893065	0.000773796	0.048926146
cg17152765	-0.449096337	3.434826304	-3.865879488	0.000773822	0.048926146
cg09495207	-0.422278681	-0.968342279	-3.865874594	0.000773831	0.048926146
cg01691377	-0.498174145	3.079974385	-3.865763285	0.000774045	0.048926146
cg07504127	-0.499607917	3.288924867	-3.865746617	0.000774077	0.048926146
cg01877366	-0.39443412	-0.29268586	-3.865711666	0.000774145	0.048926146
cg09892706	-0.62610465	2.95263951	-3.865688303	0.00077419	0.048926146
cg16978150	-0.474619429	0.607000445	-3.865621035	0.000774319	0.048926146
cg09295413	-0.409660838	0.010869012	-3.86559666	0.000774366	0.048926146
cg23680411	-0.501576031	3.71673357	-3.865583436	0.000774391	0.048926146
cg17758835	-0.457617417	0.611055405	-3.865574933	0.000774408	0.048926146
cg08712461	-0.572350577	1.777523373	-3.86556698	0.000774423	0.048926146
cg06174606	-0.447670492	4.066023931	-3.865561894	0.000774433	0.048926146
cg12068244	-0.553986221	3.215133867	-3.865538629	0.000774478	0.048926146
cg01990257	-0.67837445	3.38921715	-3.865489521	0.000774572	0.048926146
cg13100137	-0.42568334	0.8491722	-3.865435982	0.000774675	0.048926146
cg17571733	-0.747004753	1.103658788	-3.865434224	0.000774678	0.048926146
cg23102768	-0.65359644	1.654368454	-3.865408137	0.000774729	0.048926146
cg09405702	-0.603971077	3.686465013	-3.865339643	0.00077486	0.048930109
cg12338273	-0.464289442	4.336588686	-3.865226836	0.000775078	0.048937887
cg24459984	-0.564222827	-0.401801799	-3.865203897	0.000775122	0.048937887
cg16380846	0.511112237	4.062066575	3.865050293	0.000775418	0.048952202
cg01856048	-0.922040934	2.944177994	-3.864958293	0.000775595	0.048953153
cg18395636	-0.354749229	-1.213229238	-3.864944735	0.000775621	0.048953153
cg08210022	-0.71000035	2.938893175	-3.86493486	0.00077564	0.048953153
cg14762984	-0.469031968	4.10144804	-3.864893474	0.00077572	0.04895324
cg01715799	-0.408250808	-0.905768136	-3.864836418	0.00077583	0.04895324
cg03557135	-0.613402357	2.76555397	-3.86482656	0.000775849	0.04895324
cg10052236	-0.587108449	3.076193858	-3.864678851	0.000776133	0.048965313
cg09495582	-0.661127496	1.724699802	-3.864655587	0.000776178	0.048965313
cg24099990	-0.848976735	2.250372452	-3.864599165	0.000776287	0.048965972
cg09843260	-0.450517234	0.673688111	-3.864558163	0.000776366	0.048965972
cg09899007	-0.4469293	3.46442941	-3.864542635	0.000776396	0.048965972

cg15113229	-0.536724736	3.782161799	-3.864389004	0.000776692	0.048976274
cg13948104	-0.776045191	3.00767265	-3.864386281	0.000776698	0.048976274
cg17021277	-0.439327213	0.039789972	-3.864224036	0.000777011	0.048978994
cg20861386	-0.702395795	3.106736787	-3.864222092	0.000777015	0.048978994
cg12953169	-0.290269575	0.282317927	-3.864201944	0.000777053	0.048978994
cg19756470	-0.32754719	3.6722518	-3.864122598	0.000777207	0.048978994
cg18702026	-0.885088651	2.376536379	-3.864109083	0.000777233	0.048978994
cg01314194	0.513897223	-4.629185741	3.863996748	0.00077745	0.048978994
cg04322162	-0.557924391	2.78840084	-3.863986622	0.000777469	0.048978994
cg04268099	-0.500058487	3.631756219	-3.86395652	0.000777527	0.048978994
cg12794430	-0.390317526	3.878988679	-3.863950959	0.000777538	0.048978994
cg23627301	0.693172353	-0.310616377	3.863949549	0.000777541	0.048978994
cg12097634	0.339552007	0.589580118	3.863910868	0.000777616	0.048978994
cg08278773	-0.549207697	3.8406337	-3.863904363	0.000777628	0.048978994
cg09675506	-0.319912017	3.949482761	-3.863886055	0.000777663	0.048978994
cg07003097	-0.536292659	2.936824812	-3.863862557	0.000777709	0.048978994
cg05225889	-0.39313079	-0.178632577	-3.863786865	0.000777855	0.048979648
cg27331292	-0.523134163	-0.945433919	-3.863761818	0.000777903	0.048979648
cg13518992	-0.490685738	-2.30922055	-3.863749826	0.000777927	0.048979648
cg13489987	-0.815286416	2.588985164	-3.863699346	0.000778024	0.048981437
cg04085335	-0.428286265	4.252522272	-3.863534719	0.000778342	0.048992555
cg22620414	-0.458757178	-1.536794207	-3.86352159	0.000778368	0.048992555
cg19483581	-0.517059272	3.821792579	-3.863500657	0.000778408	0.048992555
cg02121539	-0.818396185	2.467385896	-3.863427741	0.000778549	0.048997077
cg07367585	-0.475453538	3.527111449	-3.8633025	0.000778792	0.049007968
cg01676506	-0.67302195	3.315879029	-3.863202672	0.000778985	0.049014399
cg25432975	-0.632800225	-1.075976267	-3.863162177	0.000779063	0.049014399
cg16010122	-0.695292019	1.831572216	-3.863121789	0.000779141	0.049014399
cg17121950	0.590509143	4.04263176	3.863106652	0.000779171	0.049014399
cg22659664	-0.453015618	3.718099331	-3.863032329	0.000779314	0.049014507
cg04131071	-0.559390828	3.714848057	-3.862996561	0.000779384	0.049014507
cg05086879	-0.425988847	-0.520826743	-3.862976905	0.000779422	0.049014507
cg07344194	-0.500906292	3.406044103	-3.862940694	0.000779492	0.049014507
cg04070200	-0.400025499	4.281349609	-3.862884873	0.0007796	0.049014507
cg24488271	-0.376485605	0.442204919	-3.862827571	0.000779711	0.049014507
cg14897238	-0.363104643	-1.715136759	-3.862766756	0.000779829	0.049014507
cg04229294	-0.56712369	3.411514271	-3.862605162	0.000780142	0.049014507
cg23026699	-0.471923626	4.136655026	-3.862598562	0.000780155	0.049014507
cg24201808	-0.662648656	2.566848328	-3.862596433	0.000780159	0.049014507
cg00105154	-0.685908746	-0.725122392	-3.862583023	0.000780185	0.049014507
cg02677777	-0.401146971	3.09317602	-3.862559108	0.000780231	0.049014507
cg07716832	-0.412249144	3.336037063	-3.862555591	0.000780238	0.049014507
cg16549587	-0.360598204	3.475981735	-3.862535833	0.000780276	0.049014507
cg19839601	-0.442402771	3.548522974	-3.8625003	0.000780345	0.049014507
cg16398882	-0.410442074	3.460213023	-3.862499363	0.000780347	0.049014507
cg25011666	-0.737346705	2.847953542	-3.862466502	0.00078041	0.049014507
cg09226014	-0.599604019	2.610667573	-3.862455263	0.000780432	0.049014507

cg23121335	-0.432554671	-2.065624818	-3.862315179	0.000780704	0.049014507
cg06201087	0.407946868	1.86996029	3.86230469	0.000780724	0.049014507
cg27459020	-0.514142331	3.832675614	-3.862235496	0.000780858	0.049014507
cg26844350	-0.588237598	3.153996319	-3.862233214	0.000780863	0.049014507
cg17749443	-0.91840584	-4.755880183	-3.862222704	0.000780883	0.049014507
cg07356515	-0.536541328	3.561447004	-3.862186187	0.000780954	0.049014507
cg02454885	-0.45867942	3.113168413	-3.862180489	0.000780965	0.049014507
cg00001638	-0.610718184	3.636514974	-3.862150794	0.000781023	0.049014507
cg14797351	-0.387796735	-0.149525304	-3.862141675	0.00078104	0.049014507
cg10908297	-0.461891121	3.947043368	-3.86210561	0.00078111	0.049014556
cg10408737	-0.547075318	3.794850759	-3.862036149	0.000781245	0.049018671
cg20602245	-0.68752634	1.087588023	-3.86199819	0.000781319	0.049018952
cg14603700	-0.439308681	2.729763091	-3.861933095	0.000781445	0.049022536
cg09940040	0.847254494	-3.641264887	3.861772521	0.000781757	0.049036093
cg02785128	-0.455043837	3.444101446	-3.861720102	0.000781859	0.049036093
cg00626312	-0.431786288	-0.786093693	-3.861695626	0.000781906	0.049036093
cg02204085	-0.467634079	4.280894851	-3.861679198	0.000781938	0.049036093
cg16576302	-0.809186486	2.370238261	-3.86148224	0.000782321	0.049050149
cg08166198	-0.378018726	4.195140713	-3.861448465	0.000782386	0.049050149
cg22986376	-0.333594852	-0.811272484	-3.861389529	0.000782501	0.049050149
cg05441830	-0.388210966	1.027900841	-3.861360093	0.000782558	0.049050149
cg04483989	-0.703248808	0.622651687	-3.861329419	0.000782618	0.049050149
cg08546406	-0.682536759	2.666121169	-3.861314414	0.000782647	0.049050149
cg15811976	-0.709735138	2.03395386	-3.861278836	0.000782716	0.049050149
cg07569575	0.503658761	2.97539316	3.861243461	0.000782785	0.049050149
cg03227728	-0.406282145	-0.069134703	-3.861243169	0.000782785	0.049050149
cg04867738	-0.734331735	-3.8293108	-3.860964075	0.000783328	0.049079823
cg02893102	-0.603019941	2.486636851	-3.860866639	0.000783518	0.049086338
cg23624095	-0.626371441	3.541550908	-3.860822703	0.000783603	0.049086338
cg02354872	-0.428492534	0.60235574	-3.86080382	0.00078364	0.049086338
cg16341987	-0.968165038	0.530091759	-3.860660175	0.00078392	0.049095526
cg21305731	-0.517772914	3.243622329	-3.860657272	0.000783925	0.049095526
cg04163795	-0.408874701	3.888693427	-3.860489348	0.000784253	0.049107771
cg15401252	-0.540431796	3.490087559	-3.860485693	0.00078426	0.049107771
cg24452783	-0.749136409	1.729966626	-3.86040739	0.000784412	0.049112981
cg18761380	-0.561659947	2.752486012	-3.860323338	0.000784576	0.049118894
cg15053029	-0.709637671	3.115854512	-3.860251398	0.000784716	0.049119783
cg24487168	-0.689360325	2.845004639	-3.860244892	0.000784729	0.049119783
cg13629022	-0.507388368	2.894774668	-3.860152862	0.000784908	0.049124984
cg09684998	-0.654711412	3.043673375	-3.860125997	0.000784961	0.049124984
cg00712935	-0.389764494	-0.421580565	-3.860081877	0.000785047	0.049124984
cg23587025	-0.528990147	-1.044664708	-3.86004456	0.000785119	0.049124984
cg11697237	-0.462308816	-2.343659561	-3.860024431	0.000785159	0.049124984
cg01119949	-0.565657629	2.560193864	-3.859936104	0.000785331	0.049131422
cg18085407	0.619167175	3.087461064	3.859853664	0.000785492	0.049133501
cg05997115	-0.490281367	3.621733537	-3.85984145	0.000785516	0.049133501
cg09230562	-0.581418881	2.92880953	-3.859776973	0.000785641	0.049133501

cg25046720	-0.438455044	-0.824025143	-3.859776868	0.000785642	0.049133501
cg21211253	-0.410176963	-0.054693921	-3.859648356	0.000785892	0.049144848
cg18267330	-0.591773208	3.073413984	-3.859548411	0.000786088	0.049147723
cg15993920	-0.396805298	0.230700942	-3.859448004	0.000786284	0.049147723
cg25108655	-0.381860441	0.852103088	-3.859389636	0.000786398	0.049147723
cg10201857	0.669242139	1.889776539	3.859269644	0.000786632	0.049147723
cg14101959	-0.428930016	3.803380458	-3.859249426	0.000786672	0.049147723
cg24720604	-0.488749794	-0.501568761	-3.859216824	0.000786735	0.049147723
cg15580665	-0.404583308	2.857610804	-3.859201124	0.000786766	0.049147723
cg04495735	-0.684016685	4.397167479	-3.85919907	0.00078677	0.049147723
cg14409249	-0.469155578	-0.431249983	-3.859191498	0.000786785	0.049147723
cg25848010	-0.481582984	3.451504956	-3.8591849	0.000786798	0.049147723
cg12536786	-0.62197182	1.947089865	-3.859166373	0.000786834	0.049147723
cg12687784	-0.427532653	3.369268169	-3.859165768	0.000786835	0.049147723
cg07197059	-0.445538414	-1.595899134	-3.859128246	0.000786908	0.049147723
cg19210625	-0.542265809	3.417845803	-3.859127522	0.00078691	0.049147723
cg16792014	-0.45734735	3.263442547	-3.859059167	0.000787043	0.049151734
cg11876390	-0.425726778	3.997084803	-3.85901967	0.000787121	0.049152222
cg20307496	-0.308510912	0.545583295	-3.858944094	0.000787268	0.049153947
cg08266464	-0.367126309	0.129953692	-3.85891125	0.000787333	0.049153947
cg00074354	-0.397640047	3.669991268	-3.858762629	0.000787623	0.049153947
cg25840921	-0.671796523	0.185729142	-3.858743005	0.000787662	0.049153947
cg02802179	-0.534005017	0.451091324	-3.85873454	0.000787678	0.049153947
cg18342621	-0.681095578	-5.100408047	-3.858731322	0.000787684	0.049153947
cg23111696	-0.548142832	3.771376339	-3.858633019	0.000787877	0.049153947
cg24360745	-0.479540336	3.446604987	-3.858593116	0.000787955	0.049153947
cg09819395	-0.429428317	3.325015585	-3.85857998	0.000787981	0.049153947
cg12411582	-0.485087565	3.116539958	-3.858554026	0.000788031	0.049153947
cg15744108	-0.507074484	3.059721426	-3.858551509	0.000788036	0.049153947
cg18663886	-0.717900277	2.983275616	-3.85854913	0.000788041	0.049153947
cg15503235	-0.39144214	-1.08967704	-3.858544405	0.00078805	0.049153947
cg12355459	-0.468982027	3.002311917	-3.858354395	0.000788422	0.049169319
cg06072564	-0.746209409	1.834300977	-3.8582796	0.000788569	0.049169319
cg19939925	-0.423648884	-2.014764198	-3.858252149	0.000788622	0.049169319
cg12793314	-0.461245303	3.600010674	-3.858252111	0.000788623	0.049169319
cg23141170	-0.718641676	0.392221486	-3.858241286	0.000788644	0.049169319
cg00232868	-0.836827214	2.348048447	-3.858154788	0.000788813	0.049171977
cg20610155	-0.478536939	3.035169801	-3.858148654	0.000788825	0.049171977
cg07064537	-0.512947126	0.157256068	-3.858054398	0.00078901	0.049173038
cg08051701	-0.779883373	2.17245944	-3.858004676	0.000789107	0.049173038
cg22586956	-0.514937145	3.242576646	-3.857994408	0.000789127	0.049173038
cg10427472	-0.713216655	2.611909667	-3.857834929	0.00078944	0.049173038
cg13068095	0.476664222	-4.421005585	3.857834527	0.000789441	0.049173038
cg19319554	-0.520946138	3.676529796	-3.857761742	0.000789584	0.049173038
cg07230959	-0.49551163	3.413620775	-3.857722482	0.000789661	0.049173038
cg09301057	-0.468555476	4.102695522	-3.857718564	0.000789668	0.049173038
cg03106717	-0.522103396	0.012890108	-3.857708497	0.000789688	0.049173038

cg08691100	-0.573000421	4.035857409	-3.857703807	0.000789697	0.049173038
cg01789048	-0.503790722	0.500210256	-3.857702679	0.000789699	0.049173038
cg00729699	0.364447177	0.977342693	3.857695388	0.000789714	0.049173038
cg15981571	-0.401965857	-0.377232208	-3.857679623	0.000789745	0.049173038
cg15590671	-0.771191055	0.568787725	-3.857634282	0.000789834	0.049174253
cg02936315	-0.30957115	-1.706287447	-3.85753802	0.000790022	0.049181689
cg16367518	-0.708128665	2.745925168	-3.857435894	0.000790223	0.049189842
cg12658587	-0.518860834	3.633620044	-3.857332587	0.000790426	0.049191914
cg19241089	-0.442277719	3.80103263	-3.857330223	0.00079043	0.049191914
cg04300535	-0.785337925	1.609673553	-3.857312798	0.000790464	0.049191914
cg00232903	-0.527201294	3.016228994	-3.857124841	0.000790834	0.049210559
cg12713604	-0.669819283	-2.458657393	-3.857052309	0.000790976	0.049215102
cg24711510	-0.383892033	3.852451607	-3.856973219	0.000791131	0.049217771
cg24685892	-0.477578554	3.301781533	-3.856937737	0.000791201	0.049217771
cg02157083	-0.517503926	2.523625395	-3.856849371	0.000791375	0.049217771
cg13601936	-0.786630973	-0.547203085	-3.856837152	0.000791399	0.049217771
cg00285431	-0.4604109	3.927436699	-3.856833375	0.000791406	0.049217771
cg11103413	-0.488847261	-0.712833203	-3.856796407	0.000791479	0.049217771
cg03324748	-0.424679832	3.978385082	-3.856782975	0.000791505	0.049217771
cg26840689	-0.326675767	-0.008159647	-3.856734304	0.000791601	0.049219401
cg27430465	-0.614494755	2.900194888	-3.856698724	0.000791671	0.04921943
cg05159423	-0.446410906	-1.882763397	-3.856631509	0.000791803	0.04922048
cg08133848	-0.428566281	-1.834324305	-3.856584029	0.000791896	0.04922048
cg11580390	-0.452719225	3.177590887	-3.856558336	0.000791947	0.04922048
cg19642748	-0.390964514	3.465172811	-3.856498076	0.000792065	0.04922048
cg14659840	-0.586082771	0.94703475	-3.856476286	0.000792108	0.04922048
cg12117412	-0.456584653	4.236915087	-3.856438998	0.000792182	0.04922048
cg01750626	-0.5487566	1.247452775	-3.856432823	0.000792194	0.04922048
cg00500047	-0.633009117	2.826487791	-3.856407515	0.000792244	0.04922048
cg05098417	-0.405874893	-1.050403078	-3.85633809	0.00079238	0.049221596
cg03412967	-0.509066002	3.14747081	-3.856327762	0.000792401	0.049221596
cg03402259	-0.475322957	3.964472361	-3.856241005	0.000792571	0.049226346
cg15665483	-0.629621865	2.793432952	-3.856196476	0.000792659	0.049226346
cg16282552	-0.477765844	3.05218583	-3.856165367	0.00079272	0.049226346
cg10072434	-0.484423598	0.062165356	-3.856147698	0.000792755	0.049226346
cg27508486	-0.484746183	3.661504772	-3.855943586	0.000793157	0.049246768
cg02997851	-0.742211771	1.56924856	-3.8559101	0.000793223	0.049246768
cg06640206	-0.771566902	1.540483086	-3.855805973	0.000793428	0.049251873
cg18126442	-0.451027252	-1.887067524	-3.855775502	0.000793488	0.049251873
cg05158146	-0.393417157	-0.109621841	-3.855762504	0.000793514	0.049251873
cg00815869	-0.863970092	2.042683691	-3.855704341	0.000793628	0.049254417
cg21789597	-0.554180833	3.47642526	-3.855671149	0.000793694	0.049254417
cg19031697	-0.460084732	3.706902323	-3.855596042	0.000793842	0.049259289
cg11798066	-0.425635881	3.494630049	-3.855479631	0.000794071	0.049269216
cg03651021	-0.391810706	3.943107236	-3.855425685	0.000794178	0.049270703
cg14386867	-0.367586255	-0.148358816	-3.855354831	0.000794317	0.049270703
cg05876179	-0.442399266	3.546500871	-3.855327468	0.000794371	0.049270703

cg07847737	-0.821621277	2.371579248	-3.855326427	0.000794374	0.049270703
cg18701707	-0.485223194	3.012235694	-3.855258498	0.000794508	0.049272008
cg03467897	-0.556965779	3.255099791	-3.855245253	0.000794534	0.049272008
cg11294276	-0.46403429	-0.384322518	-3.85518637	0.00079465	0.0492749
cg08927935	-0.400574106	4.118391255	-3.8551241	0.000794773	0.049276595
cg11692505	0.703158429	2.316926981	3.85510203	0.000794816	0.049276595
cg25384556	-0.490368851	-1.079739965	-3.855055561	0.000794908	0.049277969
cg09895893	-0.436462908	-1.56439063	-3.854928835	0.000795158	0.049286197
cg13721764	-1.221303644	-5.578931657	-3.854913427	0.000795189	0.049286197
cg05927763	-0.532384188	-1.066305658	-3.854774588	0.000795463	0.049286197
cg21105855	-0.543676005	0.966095696	-3.854767669	0.000795477	0.049286197
cg16359577	-0.543807635	-0.683636801	-3.854729201	0.000795553	0.049286197
cg27399957	-0.520423609	3.964796748	-3.854684094	0.000795642	0.049286197
cg01377475	-0.474843184	2.400945616	-3.854668038	0.000795673	0.049286197
cg18379490	-0.588521303	1.119666579	-3.854647175	0.000795715	0.049286197
cg09333298	-0.568545164	0.165642162	-3.854638461	0.000795732	0.049286197
cg24322535	-0.638942226	1.616178495	-3.8546361	0.000795737	0.049286197
cg22719998	-0.462712844	4.092589728	-3.854384017	0.000796235	0.049298822
cg08677210	0.533125489	1.648227629	3.854313217	0.000796375	0.049298822
cg13083037	-0.479816706	-0.303106221	-3.854310003	0.000796381	0.049298822
cg01515896	-0.380568412	-1.715903683	-3.854263926	0.000796472	0.049298822
cg23238776	-0.670516459	2.434631496	-3.85417668	0.000796645	0.049298822
cg06551685	-0.445216237	-1.664348348	-3.854138717	0.00079672	0.049298822
cg01707127	-0.350747188	-0.168531244	-3.854103451	0.00079679	0.049298822
cg14033979	-0.553847865	3.385780373	-3.854031409	0.000796932	0.049298822
cg17838165	-0.657313101	3.593796283	-3.854028232	0.000796939	0.049298822
cg06595479	0.825724175	2.678780649	3.853964951	0.000797064	0.049298822
cg09075378	-0.635427326	3.072509166	-3.853834135	0.000797323	0.049298822
cg09042437	-0.377613304	0.520379373	-3.853814482	0.000797362	0.049298822
cg10815411	-0.335471994	-1.38551433	-3.853796921	0.000797397	0.049298822
cg07475429	-0.737631769	1.772257162	-3.853794097	0.000797402	0.049298822
cg04064167	-0.672450063	3.118996827	-3.853743402	0.000797503	0.049298822
cg19432198	-0.4397825	3.990804324	-3.853708626	0.000797571	0.049298822
cg19537111	-0.570962576	-0.186806005	-3.853657821	0.000797672	0.049298822
cg05555202	-0.589914783	4.208336628	-3.853638376	0.000797711	0.049298822
cg09171581	-0.558968281	0.399522898	-3.853596619	0.000797793	0.049298822
cg03730738	-0.558606916	1.085971067	-3.853573953	0.000797838	0.049298822
cg13153329	-0.50088298	2.638942547	-3.853504866	0.000797975	0.049298822
cg16915628	0.437269692	1.290074588	3.853453091	0.000798078	0.049298822
cg23165500	-0.417010533	-0.350999998	-3.853435198	0.000798113	0.049298822
cg03871180	-0.5215198	3.738340859	-3.853412585	0.000798158	0.049298822
cg27114354	-0.495531986	3.775199759	-3.853409342	0.000798164	0.049298822
cg24215858	-0.401646272	4.072449994	-3.853406172	0.000798171	0.049298822
cg04663750	-0.46002471	-1.365337753	-3.853389148	0.000798204	0.049298822
cg26339153	-0.437841984	0.065156947	-3.853364353	0.000798254	0.049298822
cg22194948	-0.763063225	-4.071295144	-3.853348131	0.000798286	0.049298822
cg07764158	-0.515824	3.631011211	-3.853319128	0.000798343	0.049298822

cg00019151	-0.472000226	2.943363132	-3.853317817	0.000798346	0.049298822
cg07005706	-0.689078718	2.974352109	-3.853201633	0.000798576	0.049298822
cg03331400	-0.470162485	3.497146125	-3.853192622	0.000798594	0.049298822
cg25431366	-0.411233122	-0.769062886	-3.853141793	0.000798695	0.049298822
cg21499486	-0.563043796	1.614617002	-3.853053314	0.00079887	0.049298822
cg20118596	-0.90269306	0.067226819	-3.853035392	0.000798906	0.049298822
cg04824469	-0.541204844	3.116894377	-3.853019496	0.000798937	0.049298822
cg26729605	-0.762927847	2.301963438	-3.85299955	0.000798977	0.049298822
cg21005226	-0.348834607	0.821995237	-3.85297768	0.00079902	0.049298822
cg07066091	-0.702209117	2.921473276	-3.852969051	0.000799038	0.049298822
cg05085162	-0.521024957	3.117457528	-3.852956987	0.000799061	0.049298822
cg00432221	-0.457617052	4.371379908	-3.852951796	0.000799072	0.049298822
cg25240254	-0.479855778	0.490908995	-3.85294025	0.000799095	0.049298822
cg24373699	-0.334235656	3.759971084	-3.852933566	0.000799108	0.049298822
cg10557125	-0.440758979	-1.911319321	-3.852844801	0.000799284	0.049298822
cg05835326	-0.383205136	-0.667972978	-3.852825385	0.000799323	0.049298822
cg19218072	-0.582360309	-0.081756308	-3.852819455	0.000799334	0.049298822
cg17836805	-0.579461111	3.03100659	-3.852800197	0.000799373	0.049298822
cg00866564	-0.523919155	-1.894745029	-3.85279914	0.000799375	0.049298822
cg23854219	-0.761823011	2.785917635	-3.852776317	0.00079942	0.049298822
cg00783321	-0.609537443	2.924314218	-3.852680699	0.00079961	0.049306236
cg03696028	-0.929529182	0.762176083	-3.852645246	0.00079968	0.049306285
cg00332950	-0.577340666	2.960657921	-3.852527073	0.000799915	0.049316464
cg12533029	-0.655927236	1.04758706	-3.852394099	0.000800179	0.049328457
cg21913301	-0.474488961	3.084998824	-3.852182141	0.0008006	0.049340474
cg05373743	-0.497148714	-0.612183773	-3.852181982	0.000800601	0.049340474
cg01647887	-0.729100167	3.111670863	-3.852163605	0.000800637	0.049340474
cg16143544	-0.501377965	3.42981476	-3.852155854	0.000800653	0.049340474
cg27176425	-0.465781108	-0.680967015	-3.852033841	0.000800895	0.049351132
cg25705306	-0.371747239	0.08564824	-3.851950758	0.000801061	0.049357021
cg17821276	-0.80159988	1.714579401	-3.851869509	0.000801222	0.049359057
cg05043716	-0.50634904	0.569135804	-3.851864098	0.000801233	0.049359057
cg17394888	-0.645804865	2.892080767	-3.851804902	0.000801351	0.04936202
cg14373996	-0.317858651	0.819148487	-3.851700883	0.000801558	0.049370478
cg05889455	-0.569898287	2.22890077	-3.851631377	0.000801696	0.049372728
cg14779557	-0.512058997	3.342239973	-3.851552689	0.000801853	0.049372728
cg11760363	-0.491806955	3.825861595	-3.851535942	0.000801886	0.049372728
cg08673532	-0.425350216	3.61926542	-3.851490803	0.000801976	0.049372728
cg20936142	-0.376587786	-0.980353168	-3.851464646	0.000802028	0.049372728
cg20172156	-0.545165815	3.372037123	-3.851447609	0.000802062	0.049372728
cg03536657	-0.385101244	0.264923608	-3.851411855	0.000802133	0.049372728
cg16134098	-0.474752346	2.909161631	-3.851402504	0.000802152	0.049372728
cg26572921	-0.432102056	0.4290692	-3.851317417	0.000802321	0.04937887
cg05944635	-0.634304086	2.449209903	-3.851259617	0.000802436	0.049380521
cg26269613	-0.573856566	-3.05606989	-3.851233982	0.000802488	0.049380521
cg10875004	-0.489716444	3.913293422	-3.851008182	0.000802938	0.049403221
cg06048782	-0.495095074	3.32303573	-3.850926389	0.000803101	0.049403221

cg03908214	-0.500674631	3.76109024	-3.85085787	0.000803237	0.049403221
cg20642138	-0.701040506	-4.960395689	-3.850855899	0.000803241	0.049403221
cg20325291	-0.660118908	0.175733796	-3.850850253	0.000803253	0.049403221
cg10713220	-0.346083248	-0.512394152	-3.850839046	0.000803275	0.049403221
cg10034782	-0.347564197	-0.490115291	-3.850766517	0.00080342	0.049407829
cg12025418	-0.374868261	3.987856451	-3.850668211	0.000803616	0.049415601
cg04965463	-0.501092822	3.340305031	-3.850621788	0.000803708	0.049417008
cg19926144	-0.562453222	-0.35262972	-3.850570551	0.000803811	0.049419006
cg20992442	0.669475791	1.923393687	3.850523959	0.000803904	0.049420434
cg03874197	-0.420014536	3.707006231	-3.850230043	0.000804491	0.04944441
cg10424968	-0.542353396	2.900804572	-3.850218077	0.000804514	0.04944441
cg02484676	-0.602847914	3.034912137	-3.850131584	0.000804687	0.04944441
cg10697992	-0.33551779	0.166760495	-3.85007034	0.00080481	0.04944441
cg03299182	-0.534819047	-0.312964359	-3.850064246	0.000804822	0.04944441
cg12011406	-0.485372137	3.9578234	-3.850051388	0.000804847	0.04944441
cg02977388	-0.691919454	-1.033545575	-3.850000688	0.000804949	0.04944441
cg02410185	-0.380510691	-0.556506374	-3.849976881	0.000804996	0.04944441
cg25665909	-0.467043358	4.1991954	-3.8499451	0.00080506	0.04944441
cg00404986	0.462930754	2.024464404	3.849931648	0.000805087	0.04944441
cg03600708	-0.69958752	2.060452911	-3.849912857	0.000805124	0.04944441
cg11616028	-0.822685128	1.083215323	-3.849909435	0.000805131	0.04944441
cg06394103	-0.447127975	2.859818599	-3.84981799	0.000805314	0.04944514
cg08528404	-0.558838368	0.028020287	-3.849800915	0.000805348	0.04944514
cg06251348	-0.487014391	3.53308434	-3.849798756	0.000805353	0.04944514
cg11315699	-0.428227883	-0.48583477	-3.849709937	0.00080553	0.049447874
cg00347721	-0.451948555	3.024446836	-3.849576723	0.000805797	0.049447874
cg11532216	0.699954996	3.426341044	3.849572586	0.000805805	0.049447874
cg26448828	-0.590893022	3.538648942	-3.849569522	0.000805811	0.049447874
cg06975390	-0.488710594	3.58378527	-3.849459905	0.00080603	0.049447874
cg19410329	-0.75185612	1.536341497	-3.849458236	0.000806034	0.049447874
cg09957895	-0.623255632	3.41214689	-3.849455844	0.000806039	0.049447874
cg06760466	-0.479671329	3.333845761	-3.849423045	0.000806104	0.049447874
cg22941685	-0.420763887	1.122921811	-3.849414018	0.000806122	0.049447874
cg04164058	-0.473480306	-2.105613625	-3.849333163	0.000806284	0.049447874
cg15665304	-0.34990082	-0.908370157	-3.849309544	0.000806331	0.049447874
cg23480021	-0.517918587	3.553161935	-3.849285912	0.000806379	0.049447874
cg00952642	-0.474604983	1.060673884	-3.849198377	0.000806554	0.049447874
cg25126757	-0.576081697	3.211727097	-3.849192214	0.000806566	0.049447874
cg13765695	-0.338987468	-0.322270457	-3.849178628	0.000806594	0.049447874
cg06382028	0.683185065	4.034960533	3.849141038	0.000806669	0.049447874
cg23489627	0.440487785	-3.5110167	3.84908444	0.000806782	0.049447874
cg00688698	-0.750969468	2.293739084	-3.849081305	0.000806788	0.049447874
cg03662475	-0.491366568	3.89108617	-3.849077773	0.000806796	0.049447874
cg06332842	-0.403576329	0.727131366	-3.849053624	0.000806844	0.049447874
cg09795903	-0.79591002	2.671729265	-3.84902691	0.000806897	0.049447874
cg21628808	-0.510957673	3.571241718	-3.848983944	0.000806983	0.049447874
cg07126979	-0.53490005	1.641208148	-3.848974449	0.000807003	0.049447874

cg18996141	-0.424368173	0.4979087	-3.848735001	0.000807482	0.049469678
cg25659672	-0.408549955	4.154508638	-3.848727224	0.000807498	0.049469678
cg11806439	-0.513995859	0.599355153	-3.848639454	0.000807674	0.049471978
cg27088725	-0.491721843	-1.883859746	-3.848613747	0.000807726	0.049471978
cg26224831	-0.368709811	-0.858503844	-3.848574229	0.000807805	0.049471978
cg19480930	-0.429388168	3.732894539	-3.8485692	0.000807815	0.049471978
cg05713666	-0.619929058	3.102217366	-3.848489413	0.000807975	0.049472824
cg24829633	-0.560345171	2.855771793	-3.848482375	0.000807989	0.049472824
cg24015220	-0.495630338	3.124755643	-3.848457865	0.000808038	0.049472824
cg14924122	-0.576893755	3.184942002	-3.848395989	0.000808162	0.049476149
cg11733879	-0.565370466	2.222523521	-3.848340184	0.000808274	0.049478728
cg25227846	-0.422434908	-1.579901025	-3.848239984	0.000808476	0.049486557
cg07006588	-0.508105991	3.632982721	-3.848206858	0.000808542	0.049486557
cg15136429	-0.449777751	3.721157259	-3.848148723	0.000808659	0.049489425
cg17634975	-0.466449296	-0.010275701	-3.848107724	0.000808741	0.049490187
cg04418091	-0.93385992	-3.387386443	-3.848051067	0.000808855	0.049492224
cg24468939	-0.408211874	-0.962990718	-3.848021566	0.000808914	0.049492224
cg11966136	-0.390206436	-0.850473378	-3.847977756	0.000809002	0.049493333
cg09843907	-0.426278021	3.696323595	-3.847884802	0.000809189	0.049497123
cg16047172	-0.533820685	-0.630581411	-3.847864034	0.000809231	0.049497123
cg19458020	-0.650426032	-1.509499197	-3.847842578	0.000809274	0.049497123
cg07484253	-0.764392872	2.008629293	-3.847727487	0.000809505	0.049506994
cg06498232	-0.371463203	-1.374327189	-3.847606514	0.000809748	0.049509469
cg25396862	-0.451602311	3.419172254	-3.847581386	0.000809799	0.049509469
cg06872313	-0.453641941	-1.763545622	-3.847577399	0.000809807	0.049509469
cg17066841	-0.486380553	3.486503604	-3.847542617	0.000809877	0.049509469
cg10646193	-0.555117299	-0.521262754	-3.84742353	0.000810116	0.049509469
cg18519644	-0.984504713	0.874492277	-3.847418421	0.000810126	0.049509469
cg13951472	-0.428059438	-1.735011491	-3.847306033	0.000810352	0.049509469
cg12773057	-0.712669266	2.270637042	-3.84730501	0.000810354	0.049509469
cg14197875	-0.538992342	2.517179372	-3.847304747	0.000810355	0.049509469
cg18093631	-0.467956221	2.177205606	-3.84715671	0.000810653	0.049509469
cg27642663	-0.472247987	0.602826935	-3.847133386	0.0008107	0.049509469
cg20239485	-0.453108157	3.165315184	-3.847126609	0.000810713	0.049509469
cg16431266	-0.513038812	4.309831301	-3.847095481	0.000810776	0.049509469
cg06420554	-0.463370962	3.998573163	-3.847049149	0.000810869	0.049509469
cg23559078	-0.378666805	3.446291564	-3.84704235	0.000810883	0.049509469
cg09621868	-0.407907939	-0.61812065	-3.847026604	0.000810915	0.049509469
cg05895665	1.105582532	1.345730578	3.847008859	0.000810951	0.049509469
cg16089792	-0.454149074	-0.091044223	-3.846996553	0.000810975	0.049509469
cg02830452	-0.795049688	2.467844321	-3.846983757	0.000811001	0.049509469
cg05398019	-0.741895414	0.256716959	-3.846964811	0.000811039	0.049509469
cg00986598	-0.487707303	3.476549221	-3.846878566	0.000811213	0.049509469
cg11166681	-0.443782167	2.950128179	-3.846754287	0.000811463	0.049509469
cg19840589	-0.603313112	2.961490636	-3.846717775	0.000811537	0.049509469
cg16703780	-0.366447352	-0.566255212	-3.846624648	0.000811724	0.049509469
cg26134095	-0.350588793	3.091967762	-3.846617968	0.000811738	0.049509469

cg06239143	-0.614766931	3.587330379	-3.846496126	0.000811983	0.049509469
cg04259443	0.446133031	1.865220797	3.846492855	0.00081199	0.049509469
cg17925380	-0.663262788	2.987861898	-3.846491805	0.000811992	0.049509469
cg08866162	-0.756990507	2.211850726	-3.846475086	0.000812026	0.049509469
cg25564954	-0.46929713	4.047335966	-3.846458322	0.00081206	0.049509469
cg19402598	-0.603520033	3.072981591	-3.846411816	0.000812153	0.049509469
cg16528452	-0.567174388	3.135112327	-3.846368555	0.000812241	0.049509469
cg00337569	-0.527407367	2.176257389	-3.846313099	0.000812353	0.049509469
cg04262726	-0.607949991	1.889452806	-3.84630924	0.00081236	0.049509469
cg03379064	-0.441634445	3.557594175	-3.846308941	0.000812361	0.049509469
cg12325598	-0.353359592	-0.312687776	-3.846277432	0.000812424	0.049509469
cg15505642	-0.435083976	-0.298499759	-3.846263955	0.000812452	0.049509469
cg19895294	-0.544790473	3.020107482	-3.846105127	0.000812772	0.049509469
cg20541870	-0.636430267	-0.965885358	-3.846081473	0.00081282	0.049509469
cg08599330	0.71430196	2.789680688	3.845967681	0.000813049	0.049509469
cg15458914	-0.496690306	3.71830143	-3.845954355	0.000813076	0.049509469
cg17166397	-0.604975591	3.790780741	-3.845926763	0.000813132	0.049509469
cg22395655	0.429033679	2.063016343	3.845916152	0.000813154	0.049509469
cg06068338	-0.421783255	3.58566851	-3.845892061	0.000813202	0.049509469
cg01945263	-0.460625731	0.610141255	-3.84589092	0.000813204	0.049509469
cg17458556	-0.447389851	4.244860538	-3.845878713	0.000813229	0.049509469
cg00242955	-0.616973912	2.632463593	-3.845850265	0.000813287	0.049509469
cg14563792	-0.468165509	-0.805166018	-3.845789592	0.000813409	0.049509469
cg00272903	-0.765412381	0.735699818	-3.845766772	0.000813455	0.049509469
cg14884491	-0.549024618	3.935099087	-3.845741019	0.000813507	0.049509469
cg19020339	-0.615755921	2.80582915	-3.845735327	0.000813519	0.049509469
cg12378753	-0.798418258	-4.591538531	-3.845712389	0.000813565	0.049509469
cg11570843	-0.486359609	4.08307192	-3.845705931	0.000813578	0.049509469
cg00825168	-0.460914118	-0.294077573	-3.845696009	0.000813598	0.049509469
cg01818102	-0.442271273	3.945153067	-3.84568047	0.000813629	0.049509469
cg11024359	0.489371304	1.037661514	3.845637542	0.000813716	0.049509469
cg11770121	-0.543989811	0.889461638	-3.845630314	0.000813731	0.049509469
cg01089385	-0.4227849	3.551593253	-3.845554056	0.000813885	0.049509469
cg15874770	-0.612928639	1.900148609	-3.845551292	0.00081389	0.049509469
cg10423750	-0.594565088	-1.321717363	-3.845526801	0.00081394	0.049509469
cg17196591	-0.770887303	0.112582664	-3.845521112	0.000813951	0.049509469
cg25511278	-0.796590268	2.472521524	-3.84550999	0.000813974	0.049509469
cg18523810	-0.574835143	2.319387315	-3.845465817	0.000814063	0.049509469
cg02841802	-0.855750374	2.037010628	-3.845464398	0.000814066	0.049509469
cg23765999	-0.409674093	3.640924597	-3.845453388	0.000814088	0.049509469
cg27029473	-0.459781102	1.623495191	-3.845345924	0.000814305	0.049517316
cg02446672	-0.548163503	2.986599813	-3.84532039	0.000814357	0.049517316
cg20412744	-0.627356868	2.460387963	-3.845227533	0.000814545	0.049523605
cg00725345	0.426993479	2.031490545	3.845191209	0.000814618	0.049523605
cg21912448	-0.536197446	2.527966794	-3.845135419	0.000814731	0.049523605
cg21558029	-0.417801498	4.233668306	-3.845124608	0.000814753	0.049523605
cg12721695	0.428505537	1.785281182	3.845096397	0.00081481	0.049523605

cg20142941	-0.40824155	4.120550869	-3.845024008	0.000814957	0.049525497
cg22909949	-0.76021581	1.886812872	-3.845011895	0.000814981	0.049525497
cg24471070	-0.566603536	2.719995564	-3.844917336	0.000815172	0.049532875
cg07333715	-0.443700403	-2.036456292	-3.844782822	0.000815445	0.049535152
cg01904912	-0.751629589	2.157670999	-3.844676371	0.00081566	0.049535152
cg11402099	-0.388489087	3.920465713	-3.84467072	0.000815672	0.049535152
cg20272779	-0.417759379	3.564121724	-3.844664396	0.000815684	0.049535152
cg18064463	-0.412112671	3.875964193	-3.8446456	0.000815722	0.049535152
cg25362900	-0.930464209	1.818170877	-3.844631636	0.000815751	0.049535152
cg11785537	-0.610469882	2.304328319	-3.844583782	0.000815848	0.049535152
cg24656388	-0.475219375	3.411483526	-3.844568523	0.000815879	0.049535152
cg17533626	-0.472047033	2.944997517	-3.844553074	0.00081591	0.049535152
cg22360317	-0.45553548	4.183183447	-3.844522144	0.000815973	0.049535152
cg10760299	-0.535742174	-1.735417935	-3.844518929	0.000815979	0.049535152
cg05388262	-0.604104988	-1.887931211	-3.844443481	0.000816132	0.049537397
cg22049769	-0.422559835	4.03857477	-3.844431639	0.000816156	0.049537397
cg24338003	-0.652920658	3.158423751	-3.844342487	0.000816337	0.04953967
cg25424600	-0.446676954	4.347732908	-3.844314209	0.000816394	0.04953967
cg02660321	-0.544650119	3.589707068	-3.844241188	0.000816542	0.04953967
cg02628445	-0.517530727	3.560742729	-3.844216859	0.000816591	0.04953967
cg20141584	-0.419270403	3.847325815	-3.844206659	0.000816612	0.04953967
cg15719431	-1.007468767	1.902027633	-3.84420613	0.000816613	0.04953967
cg08299687	-0.474237858	0.54250776	-3.84410625	0.000816815	0.049547145
cg10593009	-0.360902413	-0.474900564	-3.844074472	0.00081688	0.049547145
cg19698099	-0.541881202	3.710034391	-3.844041873	0.000816946	0.049547145
cg08354227	-0.759780085	-1.173354957	-3.843908844	0.000817216	0.049552694
cg01563774	0.471574786	2.016946065	3.843896809	0.00081724	0.049552694
cg22687685	-0.533179819	2.406248934	-3.843837024	0.000817362	0.049552694
cg14230256	-0.711404829	1.387554396	-3.843817414	0.000817401	0.049552694
cg08037624	-0.579511938	-0.737025329	-3.843800113	0.000817436	0.049552694
cg17201638	-0.777586978	3.017707754	-3.843789889	0.000817457	0.049552694
cg04058080	-0.395315442	-0.653887268	-3.843643971	0.000817753	0.049554317
cg23655858	-0.557357024	3.948626005	-3.843609035	0.000817824	0.049554317
cg13827361	-0.413052848	0.084662419	-3.843592475	0.000817858	0.049554317
cg25085719	-0.413554012	3.638884759	-3.843572962	0.000817898	0.049554317
cg04656692	0.740173162	2.759799549	3.843552236	0.00081794	0.049554317
cg01858173	-0.563564798	-2.707309054	-3.843545634	0.000817953	0.049554317
cg10504333	-0.382798492	-0.224486425	-3.843487682	0.000818071	0.049554317
cg24789964	-0.546123813	2.788959091	-3.843445166	0.000818157	0.049554317
cg21007691	-0.582348759	3.232396945	-3.843442938	0.000818162	0.049554317
cg10697445	-0.55462682	3.187576249	-3.84342116	0.000818206	0.049554317
cg26926101	-0.520345464	4.31929446	-3.843397698	0.000818253	0.049554317
cg00589833	-0.447669319	3.587413015	-3.843219093	0.000818616	0.049563856
cg11549894	-0.421146869	0.121923554	-3.843208403	0.000818638	0.049563856
cg26598162	-0.43845374	-0.651359047	-3.843155796	0.000818745	0.049563856
cg03294202	-0.666359704	2.757417837	-3.843154151	0.000818748	0.049563856
cg13636978	-0.519932423	3.533435273	-3.843101952	0.000818854	0.049563856

cg26503663	-0.601551807	2.6782322	-3.843096556	0.000818865	0.049563856
cg13944105	-0.425943339	0.039924629	-3.84307852	0.000818902	0.049563856
cg23181900	0.484803465	1.538704188	3.843044753	0.000818971	0.049563856
cg12317021	-0.59983226	-1.924006939	-3.842982721	0.000819097	0.049567254
cg00028135	-0.452594681	-1.454311579	-3.842873828	0.000819318	0.04957642
cg27189374	0.587521956	-2.124693399	3.842670062	0.000819733	0.049597267
cg01364862	-0.853747126	2.086141451	-3.842576201	0.000819924	0.049604588
cg18886916	-0.644205198	3.01593629	-3.842454251	0.000820172	0.04961537
cg11621100	-0.628455591	2.323776183	-3.84239598	0.000820291	0.04961831
cg17675084	0.492403002	1.100201881	3.842195403	0.000820699	0.049631934
cg11997849	-0.427727231	3.942182784	-3.842013598	0.00082107	0.049631934
cg00916056	-0.433255523	4.092546189	-3.842002418	0.000821093	0.049631934
cg24511008	-0.441264056	3.591914929	-3.842000092	0.000821097	0.049631934
cg13022534	-0.525319748	0.908159603	-3.841876034	0.00082135	0.049631934
cg22905603	-0.569806576	3.457897912	-3.84186652	0.00082137	0.049631934
cg13498382	-0.733277055	2.717295555	-3.841844878	0.000821414	0.049631934
cg23178546	-0.436489869	4.211461841	-3.841810994	0.000821483	0.049631934
cg14645599	-0.378636082	-1.075380757	-3.841794589	0.000821516	0.049631934
cg23057621	-0.694139725	1.632614425	-3.841774414	0.000821557	0.049631934
cg15986542	0.799024126	1.530584968	3.841740058	0.000821627	0.049631934
cg26060224	-0.512488361	3.326943797	-3.841733923	0.00082164	0.049631934
cg14115218	-0.490288795	3.095529239	-3.841693086	0.000821723	0.049631934
cg26363272	-0.645250379	3.378226598	-3.841685201	0.000821739	0.049631934
cg01432329	-0.545819447	-0.741754138	-3.841614436	0.000821884	0.049631934
cg01807030	-0.480736304	0.100996787	-3.841610586	0.000821892	0.049631934
cg19769305	-0.472858935	3.262163173	-3.841576057	0.000821962	0.049631934
cg25153629	-0.496140418	0.82797639	-3.841556686	0.000822002	0.049631934
cg18774382	-0.948070128	0.765871077	-3.84154909	0.000822017	0.049631934
cg22855764	-0.463284955	-0.260426555	-3.841484618	0.000822149	0.049631934
cg08140255	0.520892411	2.780472423	3.841483947	0.00082215	0.049631934
cg08912287	-0.492065507	-0.734965202	-3.841474831	0.000822169	0.049631934
cg17074310	-0.398905928	3.800848985	-3.84142506	0.00082227	0.049631934
cg06661901	-0.455331131	3.950751457	-3.84141016	0.000822301	0.049631934
cg11231220	-0.751776713	2.007654092	-3.841404461	0.000822312	0.049631934
cg08411094	-0.535447815	3.356737854	-3.841391235	0.000822339	0.049631934
cg15191330	-0.556051365	3.720796769	-3.84135768	0.000822408	0.049631934
cg07485401	-0.427937797	3.183623321	-3.841320238	0.000822484	0.049632319
cg03206533	0.606356148	2.467522343	3.84126477	0.000822597	0.049634802
cg02136521	-0.687334414	-0.73466479	-3.841175582	0.00082278	0.049634802
cg19128882	-0.516386418	3.754227569	-3.841168496	0.000822794	0.049634802
cg06880028	0.484484965	-0.094902013	3.841129898	0.000822873	0.049634802
cg14310811	-0.50392471	3.143487778	-3.841128533	0.000822876	0.049634802
cg13447820	-0.642365167	1.055816341	-3.84107186	0.000822991	0.049637559
cg21614855	-0.521142513	-1.265511275	-3.840998197	0.000823142	0.049637808
cg01897944	-0.482051951	-0.09547196	-3.84095753	0.000823225	0.049637808
cg06079317	-0.501476454	3.023585837	-3.84094548	0.00082325	0.049637808
cg11169640	-0.613531572	3.678565013	-3.840910839	0.00082332	0.049637808

cg09731767	-0.432509165	0.025671012	-3.840892462	0.000823358	0.049637808
cg04180924	-0.501132693	0.101847407	-3.840828705	0.000823488	0.049637808
cg04314155	-0.42842027	-0.944146467	-3.840825583	0.000823495	0.049637808
cg16243898	-0.519820139	-0.187045465	-3.84077459	0.000823599	0.049637808
cg09024023	-0.64486474	2.494932676	-3.84076125	0.000823626	0.049637808
cg16597778	-0.510091613	0.943651209	-3.840685549	0.000823781	0.049639082
cg05414781	-0.679705598	2.367783489	-3.840673223	0.000823806	0.049639082
cg11366775	-0.453527516	-0.234451975	-3.840648105	0.000823858	0.049639082
cg23481109	-0.536627255	3.798617101	-3.840603997	0.000823948	0.049640295
cg07530596	-0.931011435	2.32205111	-3.840535019	0.000824089	0.049644573
cg18467421	-0.424971234	4.023128941	-3.840497779	0.000824165	0.04964494
cg27525272	-0.468846093	3.459114826	-3.840398257	0.000824369	0.04964506
cg04351607	-0.598018669	0.082599433	-3.840335964	0.000824496	0.04964506
cg22239325	-0.477913862	-0.111161036	-3.840320422	0.000824528	0.04964506
cg09918008	-0.349562871	3.603690154	-3.840316058	0.000824537	0.04964506
cg26646061	-0.492934431	3.673519724	-3.84028258	0.000824605	0.04964506
cg20226593	-0.531398314	3.566156909	-3.84028229	0.000824606	0.04964506
cg04029502	-0.586900141	2.838178408	-3.84025708	0.000824658	0.04964506
cg26787589	-0.393145101	4.046655752	-3.840101531	0.000824976	0.049660012
cg17255005	-0.40045963	4.068468109	-3.84003206	0.000825118	0.049664355
cg08240847	-0.476113119	2.891908176	-3.839933722	0.00082532	0.049670525
cg25103337	-0.325825463	-0.357951526	-3.839913563	0.000825361	0.049670525
cg01854144	-0.410836801	3.424901903	-3.839821967	0.000825549	0.04967189
cg22844232	-0.535821868	-2.095380127	-3.839808556	0.000825576	0.04967189
cg00154367	-0.655385518	1.958508428	-3.839799822	0.000825594	0.04967189
cg26036648	-0.472565599	3.68988301	-3.839583366	0.000826038	0.049684613
cg00071391	-0.463770728	0.246391688	-3.839535701	0.000826136	0.049684613
cg18531903	-0.602494785	-1.087497582	-3.839527197	0.000826153	0.049684613
cg11299856	-0.699369392	1.817624202	-3.839522225	0.000826163	0.049684613
cg17988307	-0.341572581	3.609151197	-3.839493752	0.000826222	0.049684613
cg06209718	-0.461518569	2.71143098	-3.839491395	0.000826226	0.049684613
cg26266991	0.60418085	1.041708018	3.839456493	0.000826298	0.0496847
cg20353685	0.738609142	3.172800231	3.839390526	0.000826433	0.049685607
cg10093067	-0.746732954	0.782779495	-3.839301429	0.000826616	0.049685607
cg02687548	-0.389257417	-0.635484499	-3.839290365	0.000826639	0.049685607
cg14831653	-0.516750819	2.271286424	-3.83928549	0.000826649	0.049685607
cg09916797	-0.559617929	3.362017141	-3.83927819	0.000826664	0.049685607
cg06564376	-0.472960635	2.732390635	-3.839205003	0.000826814	0.049690417
cg22544398	-0.572657536	3.031983597	-3.839164969	0.000826896	0.049691139
cg19983759	-1.089967268	2.631337163	-3.839067009	0.000827097	0.049695066
cg20197715	-0.54633914	3.008915631	-3.839055143	0.000827122	0.049695066
cg08443426	-0.705494425	-0.266400896	-3.839019789	0.000827194	0.049695066
cg07076326	-0.531022352	3.257696596	-3.838996453	0.000827242	0.049695066
cg24560783	-0.822867002	1.96919697	-3.838949951	0.000827338	0.049695788
cg19213762	-0.551891705	2.499934111	-3.838922268	0.000827394	0.049695788
cg10421435	-0.518707275	3.946179698	-3.838870277	0.000827501	0.049697665
cg21449902	-0.751508523	2.098806742	-3.838823297	0.000827598	0.049697665

cg01919377	-0.481824211	-1.190084451	-3.838804585	0.000827636	0.049697665
cg07413140	-0.382801363	0.96240993	-3.838728379	0.000827793	0.049699011
cg05801207	-0.467543083	4.270224713	-3.838725379	0.000827799	0.049699011
cg21410293	-0.49473996	-2.023852846	-3.838643662	0.000827967	0.04970147
cg07758192	-0.571417449	-0.474469138	-3.83863716	0.00082798	0.04970147
cg00378647	-0.497037572	4.013962982	-3.838446401	0.000828372	0.049703661
cg16874710	-0.463781116	4.221895986	-3.838436692	0.000828392	0.049703661
cg00816564	-0.519748027	3.555401357	-3.838401427	0.000828465	0.049703661
cg10508271	-0.386456057	3.395855686	-3.838378023	0.000828513	0.049703661
cg02265748	-0.617449587	-1.03965068	-3.838322899	0.000828626	0.049703661
cg13665998	-1.384289241	-4.01828842	-3.838307402	0.000828658	0.049703661
cg24849633	0.39621705	1.101365475	3.838290833	0.000828692	0.049703661
cg19226975	-0.517887336	3.729468695	-3.838255188	0.000828765	0.049703661
cg01048937	-0.376077148	4.206331388	-3.838247269	0.000828782	0.049703661
cg21401359	-0.81373856	-1.336041957	-3.838236545	0.000828804	0.049703661
cg02465741	-0.537339643	3.16086243	-3.838227961	0.000828822	0.049703661
cg19161353	-0.501684776	3.193070021	-3.83817272	0.000828935	0.049703661
cg03244325	-0.4753987	3.770986012	-3.838140618	0.000829001	0.049703661
cg17534092	-0.457225007	-0.927845943	-3.838081928	0.000829122	0.049703661
cg18342872	-0.445539891	3.361948927	-3.838025282	0.000829239	0.049703661
cg01571633	-0.418518874	3.454149828	-3.837929728	0.000829435	0.049703661
cg08376533	-0.434389244	3.823757201	-3.837916881	0.000829462	0.049703661
cg18991915	-0.591205873	3.370469335	-3.837877845	0.000829542	0.049703661
cg19151455	-0.462553099	3.018263958	-3.837846281	0.000829607	0.049703661
cg24002653	-0.663723357	0.790284832	-3.837845114	0.000829609	0.049703661
cg07597512	-0.498125441	3.580213329	-3.837833458	0.000829633	0.049703661
cg15604158	-0.432044412	3.717156007	-3.837787598	0.000829728	0.049703661
cg00924575	-0.543880212	0.252996082	-3.837787227	0.000829729	0.049703661
cg07476353	-0.546260606	2.72540851	-3.837762039	0.000829781	0.049703661
cg00338116	-0.5060379	-2.792716184	-3.837749354	0.000829807	0.049703661
cg12047361	-0.550362731	3.235499632	-3.837717332	0.000829873	0.049703661
cg14177306	-0.596323883	1.751281542	-3.837698658	0.000829911	0.049703661
cg03442375	-0.486689451	-0.488472345	-3.837610785	0.000830092	0.0497103
cg13120814	-0.529876133	-1.200219085	-3.837449439	0.000830425	0.049723372
cg05478905	-0.496051772	3.126016643	-3.837424072	0.000830477	0.049723372
cg10252369	-0.541162142	1.005156719	-3.837363961	0.000830601	0.049723372
cg25791315	-0.628658859	2.619038331	-3.837348448	0.000830633	0.049723372
cg09024049	-0.472004741	3.159596676	-3.837334581	0.000830661	0.049723372
cg16668903	-0.414436427	0.659007194	-3.837252945	0.00083083	0.049724353
cg25608560	-0.475159158	2.765834957	-3.83721822	0.000830901	0.049724353
cg10673484	-0.760740844	3.153984239	-3.837191077	0.000830957	0.049724353
cg25744776	-0.480550186	3.277279024	-3.837167165	0.000831007	0.049724353
cg08205361	-0.335552675	-1.218424952	-3.837125324	0.000831093	0.049724353
cg12444450	-0.516422739	0.127140332	-3.837094168	0.000831157	0.049724353
cg20929474	-0.389169747	3.15977354	-3.837085577	0.000831175	0.049724353
cg12976965	-0.875349134	1.296561937	-3.83704622	0.000831256	0.049724353
cg09787101	-0.446954581	-0.049523131	-3.837020335	0.000831309	0.049724353

cg23683962	-0.463569661	3.954790933	-3.836928538	0.000831499	0.049729999
cg16633687	-0.368587977	4.513432161	-3.836889141	0.00083158	0.049729999
cg26478333	-0.437880949	3.534323493	-3.836818781	0.000831725	0.049729999
cg15647328	-0.486541675	3.906370408	-3.836811881	0.00083174	0.049729999
cg10074661	-0.477294617	3.323156673	-3.836804517	0.000831755	0.049729999
cg19215206	-0.54107797	2.61952801	-3.836644327	0.000832086	0.049733891
cg08514128	-0.489229754	3.048903092	-3.836629745	0.000832116	0.049733891
cg00832546	-0.533756691	-1.081074159	-3.836617212	0.000832142	0.049733891
cg03759001	-0.525611854	2.617438608	-3.836585707	0.000832207	0.049733891
cg12642773	-0.449656255	3.988057577	-3.836582338	0.000832214	0.049733891
cg20448308	-0.457703101	0.36217572	-3.836569	0.000832241	0.049733891
cg21433612	-0.38926	3.275689875	-3.83646389	0.000832458	0.049741684
cg07356373	-0.480635303	2.971478119	-3.836408964	0.000832572	0.049741684
cg26800107	-0.647899712	2.993580259	-3.836403909	0.000832582	0.049741684
cg19403132	0.550836943	1.47408766	3.836328211	0.000832739	0.049746835
cg11857223	-0.443988275	-1.746251927	-3.836172104	0.000833061	0.049755681
cg08886020	-0.386249346	0.608034946	-3.836169912	0.000833066	0.049755681
cg18579187	-0.585758999	0.666249793	-3.836154644	0.000833098	0.049755681
cg19002094	-0.432711477	3.711839568	-3.836089199	0.000833233	0.049759568
cg10647959	-0.562938051	3.685772742	-3.835993264	0.000833431	0.049767222
cg14690181	-0.350469546	-0.2325667	-3.835943832	0.000833534	0.049769133
cg07055397	-0.45852634	-0.114256404	-3.835896327	0.000833632	0.049770806
cg25975856	-0.675376753	-2.035070263	-3.835837979	0.000833753	0.049772369
cg18421806	-0.582555797	3.268150483	-3.835815763	0.000833799	0.049772369
cg03467567	-0.79814237	1.561019826	-3.83577357	0.000833886	0.049773387
cg07475527	-0.505156983	3.380149589	-3.835674618	0.000834091	0.049779225
cg11843735	0.564125974	2.08233097	3.835643092	0.000834156	0.049779225
cg26980152	-0.447217928	4.199646748	-3.835624469	0.000834194	0.049779225
cg21209212	-0.426965983	3.35712409	-3.835584161	0.000834278	0.049780012
cg13940125	-0.543806915	-0.150833659	-3.835482754	0.000834488	0.049786036
cg15649327	-0.373212008	4.104222722	-3.835467528	0.000834519	0.049786036
cg09156067	0.638624508	-4.445984683	3.835418136	0.000834622	0.049787491
cg10332422	-0.462253582	4.464079354	-3.835387895	0.000834684	0.049787491
cg07687673	-0.41984692	0.312108027	-3.835248706	0.000834973	0.049794229
cg07347534	-0.370040431	3.444545489	-3.835202627	0.000835068	0.049794229
cg09830059	-0.476577791	2.940155479	-3.835171509	0.000835133	0.049794229
cg08213963	-0.661711111	3.322569286	-3.83516784	0.00083514	0.049794229
cg25988105	-0.360728404	3.669936343	-3.835163765	0.000835149	0.049794229
cg08814860	-0.731771892	2.467590729	-3.835018566	0.00083545	0.049805866
cg13258528	-0.466568347	2.679126279	-3.83499454	0.0008355	0.049805866
cg17282466	-0.576926896	2.748907401	-3.834967878	0.000835555	0.049805866
cg19709675	-0.421142295	3.812084226	-3.834894461	0.000835707	0.049810491
cg23464327	-0.472223184	2.75649538	-3.834862661	0.000835773	0.049810491
cg20903136	-0.674371873	3.223413024	-3.834784457	0.000835935	0.049815968
cg08223323	-0.702389397	3.725646549	-3.834719639	0.00083607	0.049819791
cg19759804	-0.354074459	-1.004259462	-3.834516156	0.000836492	0.049829632
cg05303639	-0.504446354	0.975830513	-3.834504096	0.000836517	0.049829632

cg14742187	-0.667517507	0.949709943	-3.834434736	0.000836661	0.049829632
cg02630510	-0.700351554	2.482773488	-3.834415682	0.000836701	0.049829632
cg03330372	-0.454420725	3.639897195	-3.834384038	0.000836766	0.049829632
cg00928751	-0.435913466	0.987559655	-3.834382877	0.000836769	0.049829632
cg14749261	-0.420297844	3.856417595	-3.834372464	0.000836791	0.049829632
cg13712826	-0.729105946	2.287190528	-3.834368984	0.000836798	0.049829632
cg26584329	-0.593595277	2.265885778	-3.834190214	0.000837169	0.049839307
cg13001250	-0.366848985	-0.995511638	-3.834177337	0.000837196	0.049839307
cg07100242	-0.525244362	2.740628904	-3.83415321	0.000837246	0.049839307
cg12543045	0.35489414	1.494813701	3.834150822	0.000837251	0.049839307
cg20268967	-0.598305016	2.235249977	-3.834090375	0.000837377	0.049839307
cg14456629	-0.587498759	0.22606724	-3.834087574	0.000837382	0.049839307
cg22946648	-0.356960403	-0.896897711	-3.83402961	0.000837503	0.049842289
cg02181555	-0.783971685	1.449301753	-3.833936175	0.000837697	0.049848337
cg00721453	-0.442889371	3.058816166	-3.83391302	0.000837745	0.049848337
cg15630394	-0.54642499	2.877800528	-3.833875304	0.000837824	0.049848816
cg15865113	-0.553736717	3.673564059	-3.833830691	0.000837916	0.049850148
cg00937699	-0.539858637	3.351896977	-3.833764212	0.000838055	0.049850918
cg12719585	-0.524040507	3.422989123	-3.833736999	0.000838111	0.049850918
cg03677635	-0.58703643	-0.180963139	-3.833666502	0.000838258	0.049850918
cg04873259	-0.657687278	1.500201673	-3.833646963	0.000838299	0.049850918
cg08594696	0.616781598	2.653131753	3.833603905	0.000838388	0.049850918
cg12934558	-0.490520151	-1.167142444	-3.833538425	0.000838524	0.049850918
cg09884146	0.482044124	1.285909017	3.833535333	0.000838531	0.049850918
cg22350358	-0.533733367	-0.040913551	-3.833516411	0.00083857	0.049850918
cg06924792	-0.49747201	2.935765386	-3.833514312	0.000838575	0.049850918
cg26601267	-0.523539442	3.419603265	-3.833486198	0.000838633	0.049850918
cg11192749	-0.52004421	0.756377052	-3.833341241	0.000838935	0.049857242
cg25560215	-0.354535923	-0.58961034	-3.833305916	0.000839008	0.049857242
cg05119709	-0.61156037	2.243846854	-3.833283826	0.000839054	0.049857242
cg09409261	-0.83236536	1.350523853	-3.833248583	0.000839128	0.049857242
cg22748674	-0.360564204	-0.273384218	-3.833236641	0.000839153	0.049857242
cg07174177	-0.71309845	0.92384886	-3.833177172	0.000839276	0.049857242
cg01162108	-0.44390424	3.994841893	-3.833175802	0.000839279	0.049857242
cg12471732	-0.66224668	2.776571982	-3.833164666	0.000839302	0.049857242
cg27510568	-0.710924147	0.759715235	-3.832916656	0.000839819	0.049882258
cg09796638	-0.471157829	3.542492006	-3.832894955	0.000839864	0.049882258
cg07621982	-0.501252233	-1.245556839	-3.832835542	0.000839988	0.04988543
cg26794346	-0.413824905	4.326908306	-3.8327627	0.00084014	0.049890266
cg11661235	-0.559725437	2.463274561	-3.83270531	0.00084026	0.04989281
cg13542793	-0.468590705	3.262393293	-3.832660028	0.000840354	0.04989281
cg01990013	-0.58080974	3.195778382	-3.832640823	0.000840394	0.04989281
cg26075742	-0.552599229	3.992289429	-3.832545107	0.000840594	0.04990048
cg26772104	-0.369916572	4.34551619	-3.832450335	0.000840792	0.049908035
cg03138863	-0.501506659	3.30208142	-3.832305348	0.000841094	0.049921812
cg11199827	-0.448382481	3.091690091	-3.832235056	0.000841241	0.049922282
cg03430065	-0.395167268	4.227634856	-3.832191865	0.000841331	0.049922282

cg03348965	-0.666787541	3.556972202	-3.83217391	0.000841369	0.049922282
cg16000198	-0.566788888	3.188822865	-3.832166523	0.000841384	0.049922282
cg08086157	-0.653086615	3.092729342	-3.832086388	0.000841551	0.049923894
cg24296196	-0.60468597	3.182520017	-3.832086013	0.000841552	0.049923894
cg00118977	-0.510698336	3.134298198	-3.832047898	0.000841632	0.049924435
cg06866993	-0.479551969	3.469799175	-3.831987835	0.000841757	0.049927696
cg19117630	-0.420470518	4.385846133	-3.831928446	0.000841881	0.049930874
cg15434432	-0.584044172	2.793826216	-3.831861547	0.000842021	0.049934983
cg27287446	-0.563995226	-0.588672042	-3.831722693	0.000842311	0.049944723
cg06731150	-0.51043891	-2.229242831	-3.831691946	0.000842375	0.049944723
cg12656963	-0.623277013	2.142411568	-3.831681757	0.000842397	0.049944723
cg05674944	0.339365259	-3.485701743	3.831639461	0.000842485	0.049945785
cg10874314	-0.375903704	-0.145769796	-3.831487103	0.000842804	0.049960491
cg25764614	-0.55076491	2.507805587	-3.831274257	0.000843249	0.049972631
cg08799790	-0.633973548	3.102392739	-3.831260179	0.000843279	0.049972631
cg11809417	-0.442189525	4.371991469	-3.831254601	0.00084329	0.049972631
cg20635102	-0.787059344	1.882344562	-3.831254332	0.000843291	0.049972631
cg26151727	-0.413016066	-1.455661461	-3.831084469	0.000843646	0.049989517
