# Supplementary figures and images for: Integrative Analysis Unveils the Correlation of Aminoacyl-tRNA Biosynthesis Metabolites with the Methylation of the SEPSECS Gene in Huntington’s Disease Brain Tissue
Source: Genes (Basel). 2023 Sep 2;14(9):1752. doi: 10.3390/genes14091752 (PMC10530570; doi:10.3390/genes14091752)

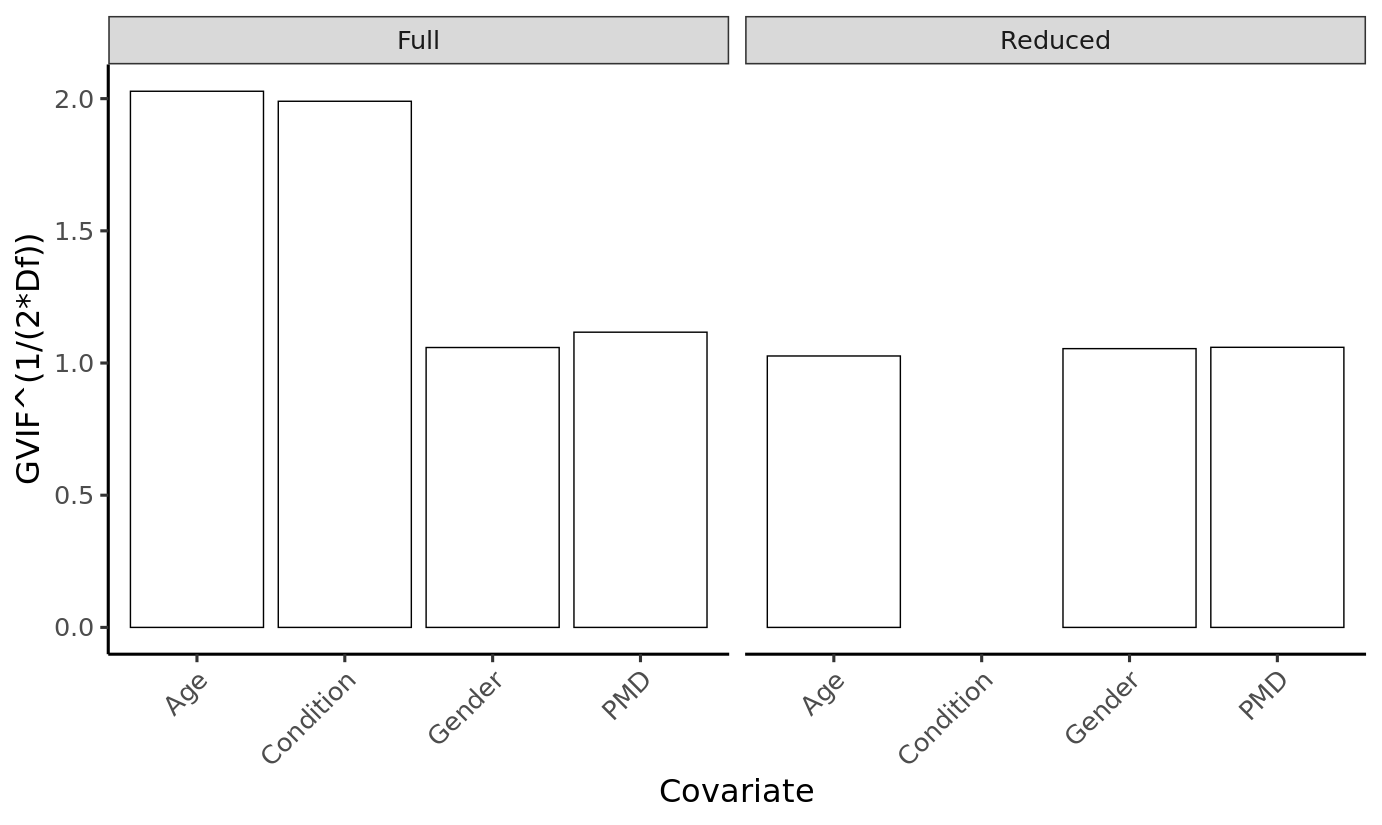

Supplement: Supplementary file 1 [file genes-14-01752-s001.zip › 12-HD-Supple Fig 1- Metabolomics-Striatum-Variance inflation.png]

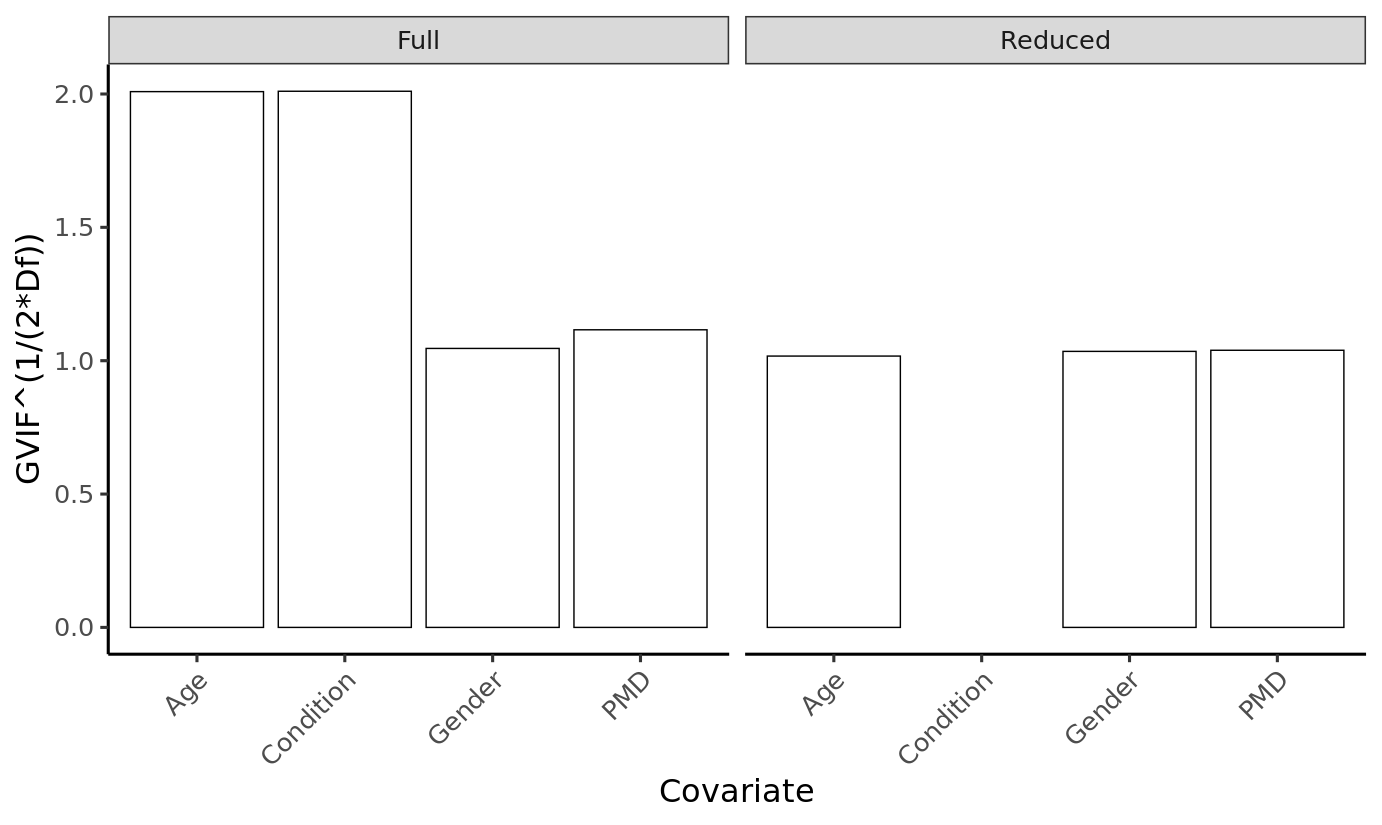

Supplement: Supplementary file 1 [file genes-14-01752-s001.zip › 13-HD-Supple Fig 2- Metabolomics-Frontal-Variance inflation.png]

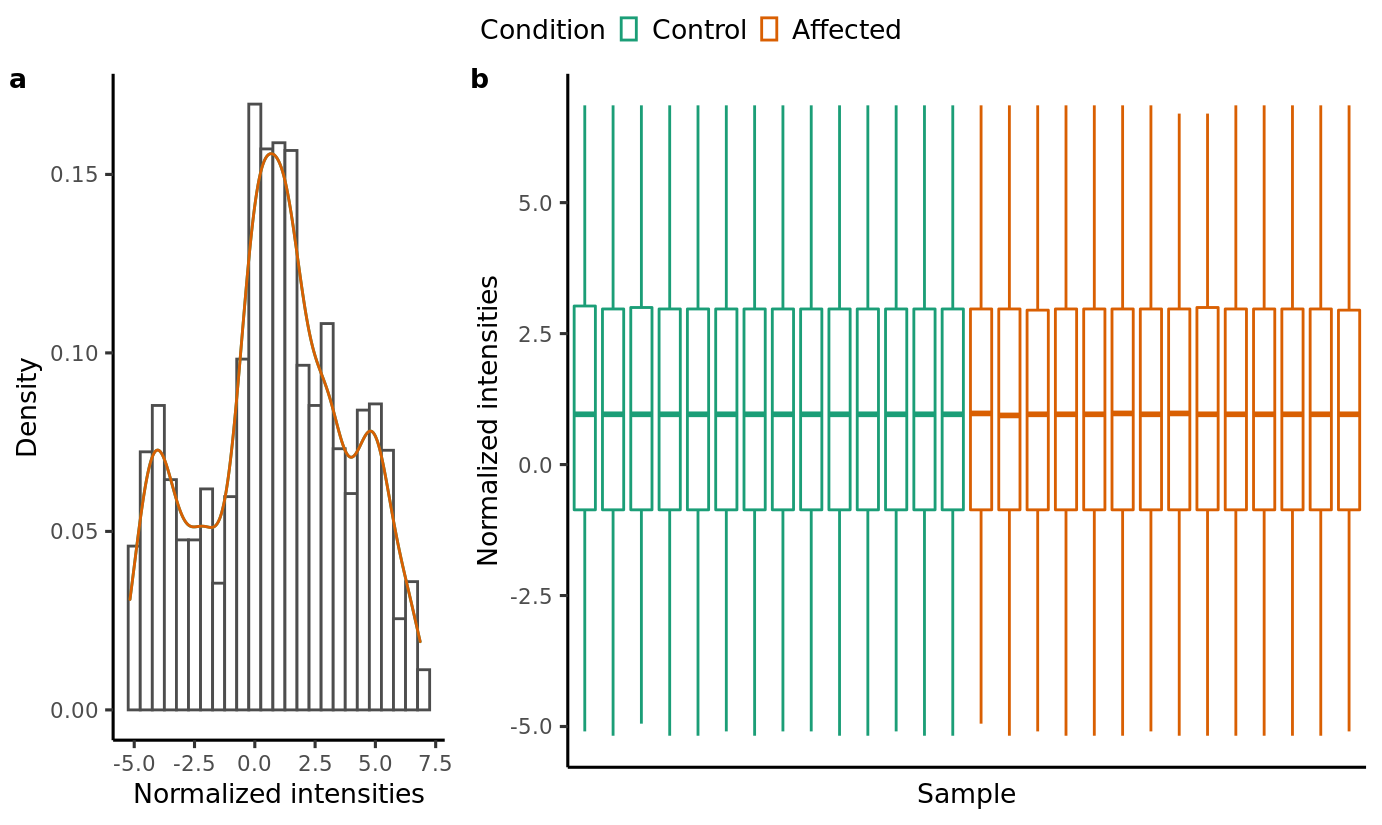

Supplement: Supplementary file 1 [file genes-14-01752-s001.zip › 14-HD-Supple Fig 3- Metabolomics-Striatum-Normalization.png]

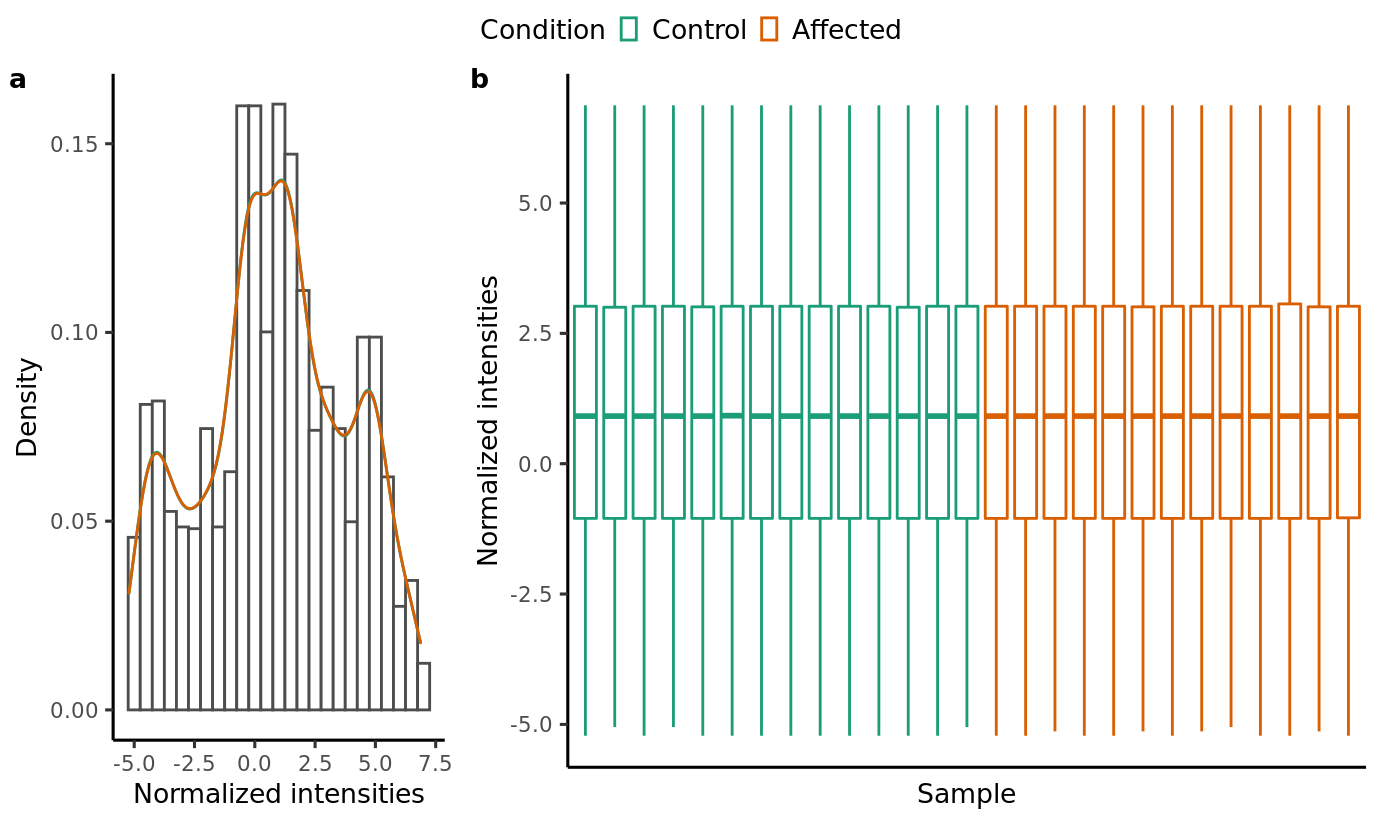

Supplement: Supplementary file 1 [file genes-14-01752-s001.zip › 15-HD-Supple Fig 4- Metabolomics-Frontal-Normalization.png]

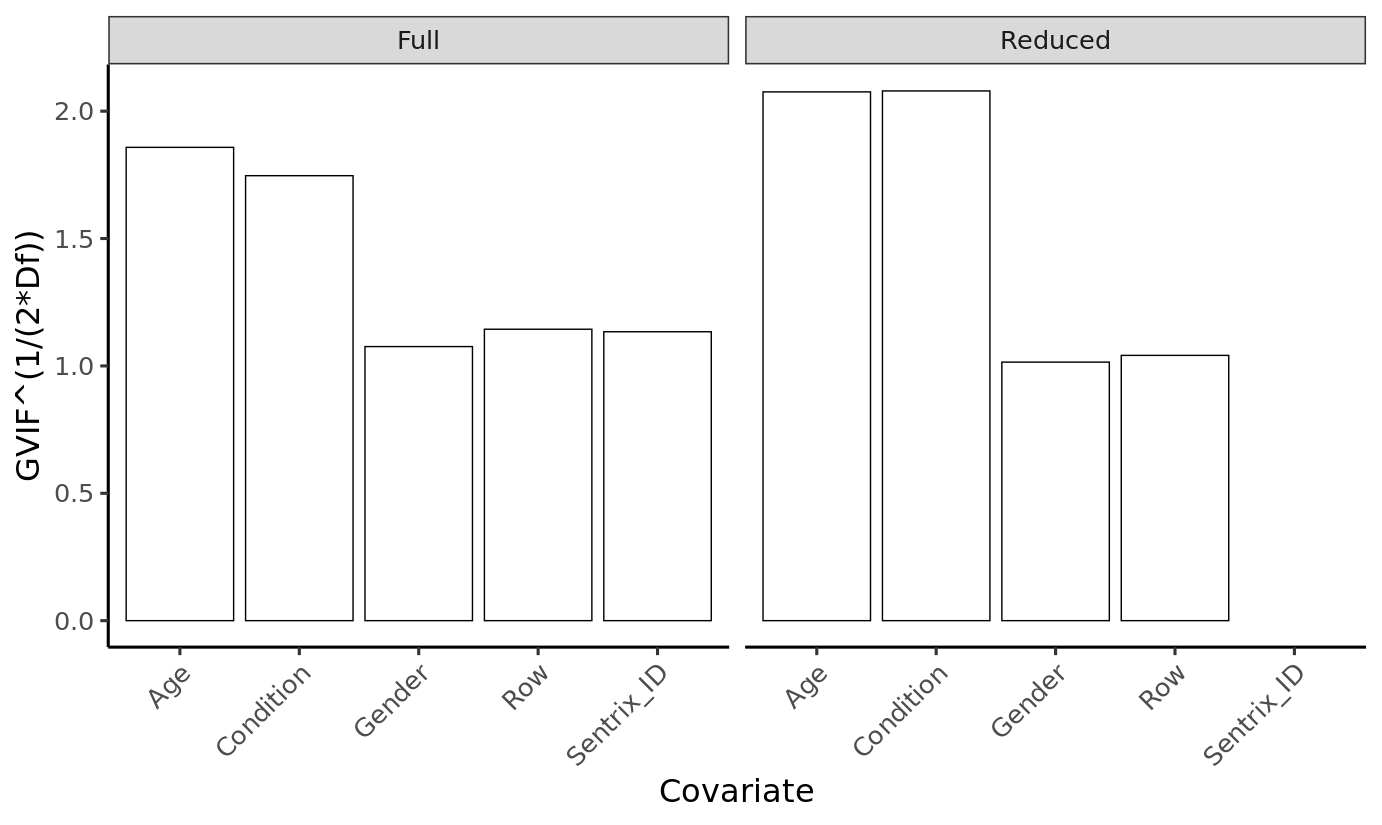

Supplement: Supplementary file 1 [file genes-14-01752-s001.zip › 16-HD-Supple Fig 5- Epic array variance inflation.png]

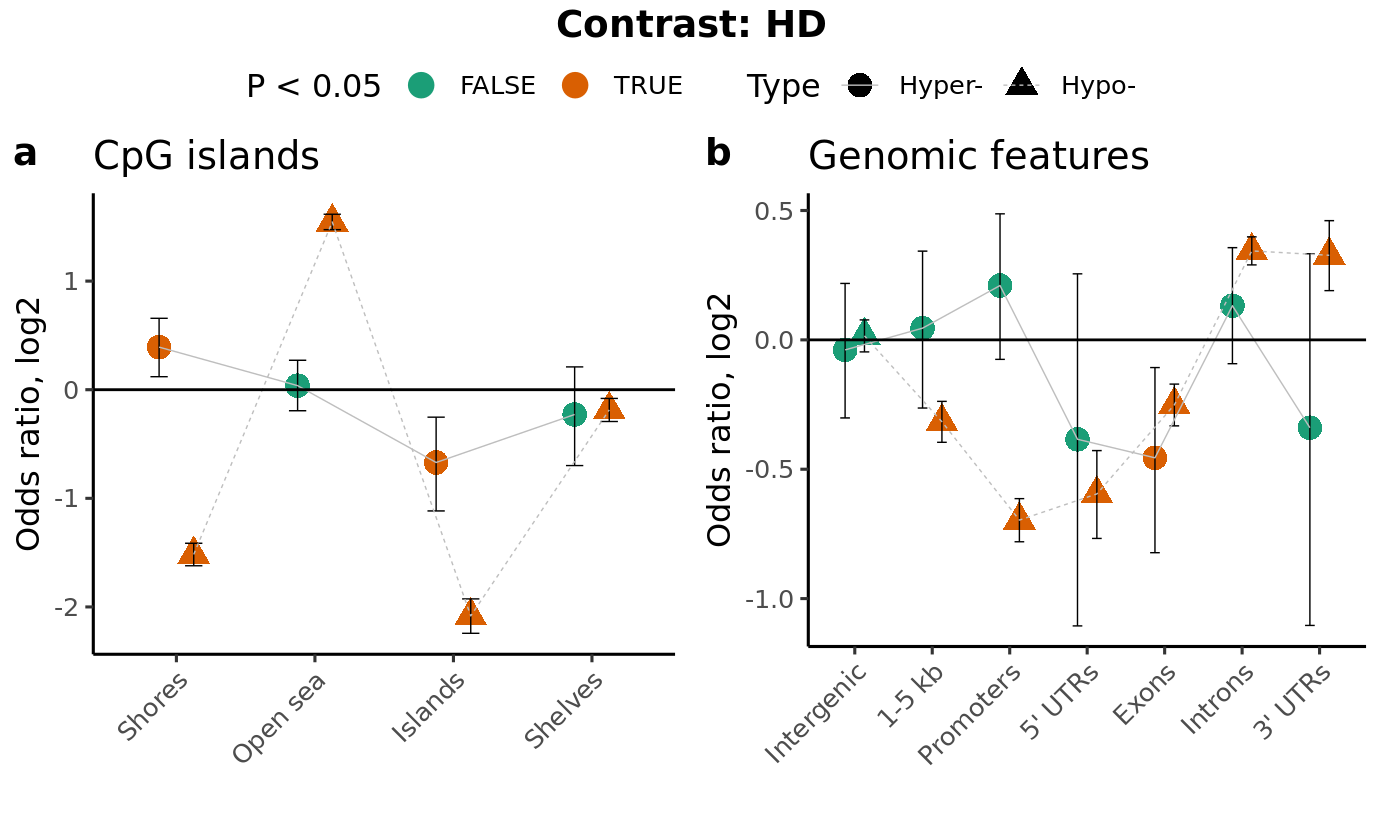

Supplement: Supplementary file 1 [file genes-14-01752-s001.zip › 17-HD-Supple Fig 6- EPIC-Enrichment of genomic region.png]

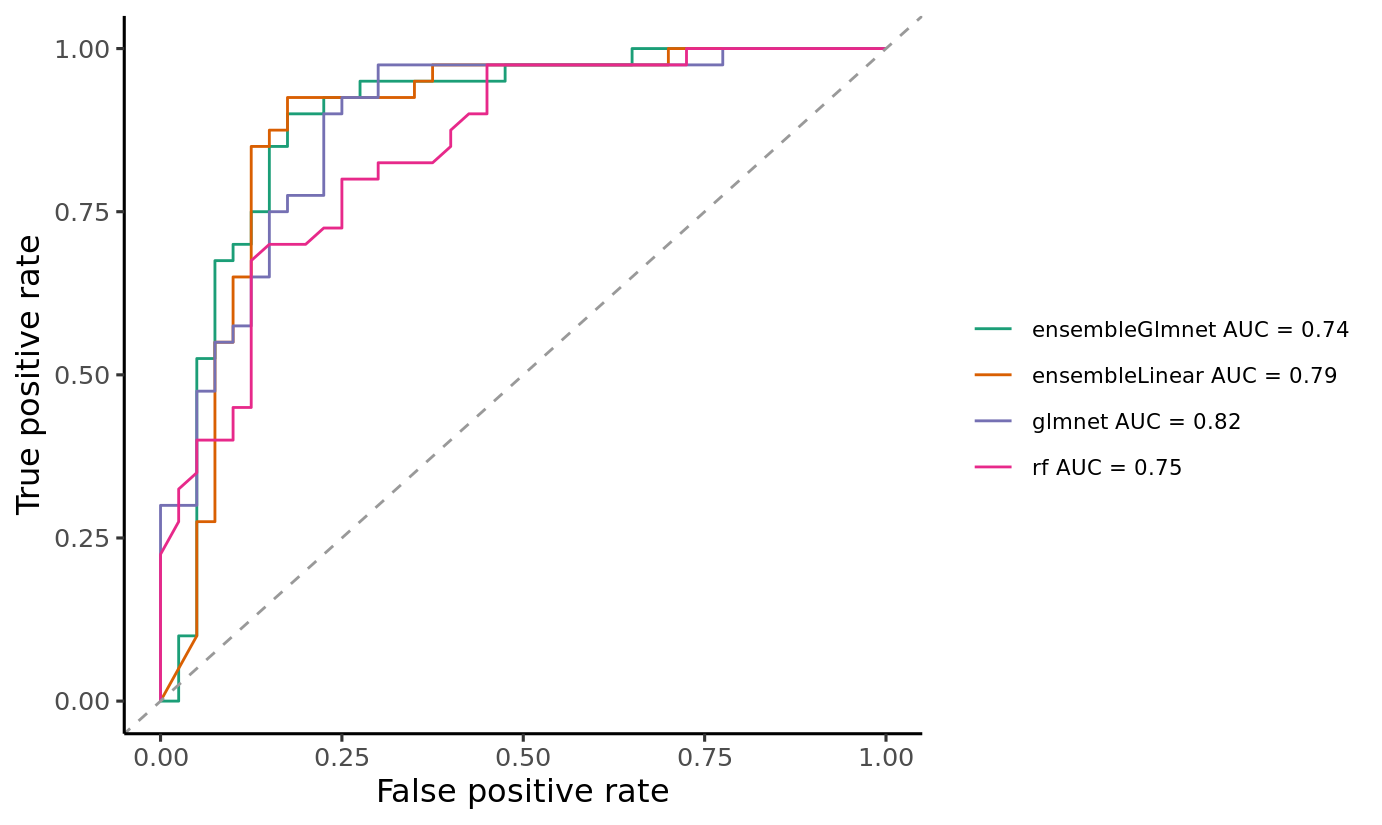

Supplement: Supplementary file 1 [file genes-14-01752-s001.zip › 18-HD-Supple Fig 7- Striatum - Metabolomics - Predictor.png]

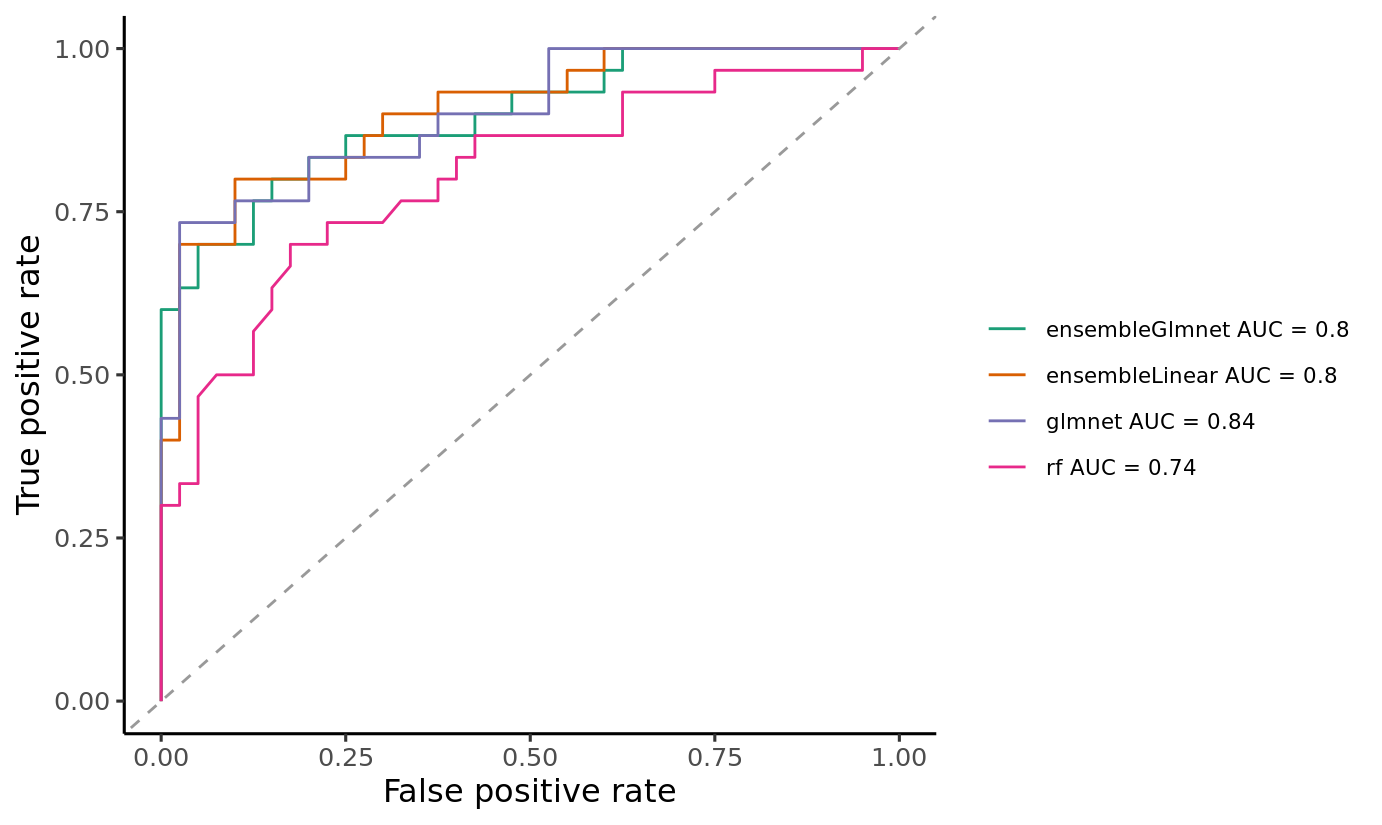

Supplement: Supplementary file 1 [file genes-14-01752-s001.zip › 19-HD-Supple Fig 8- Frontal lobe - Metabolomics - Predictor.png]

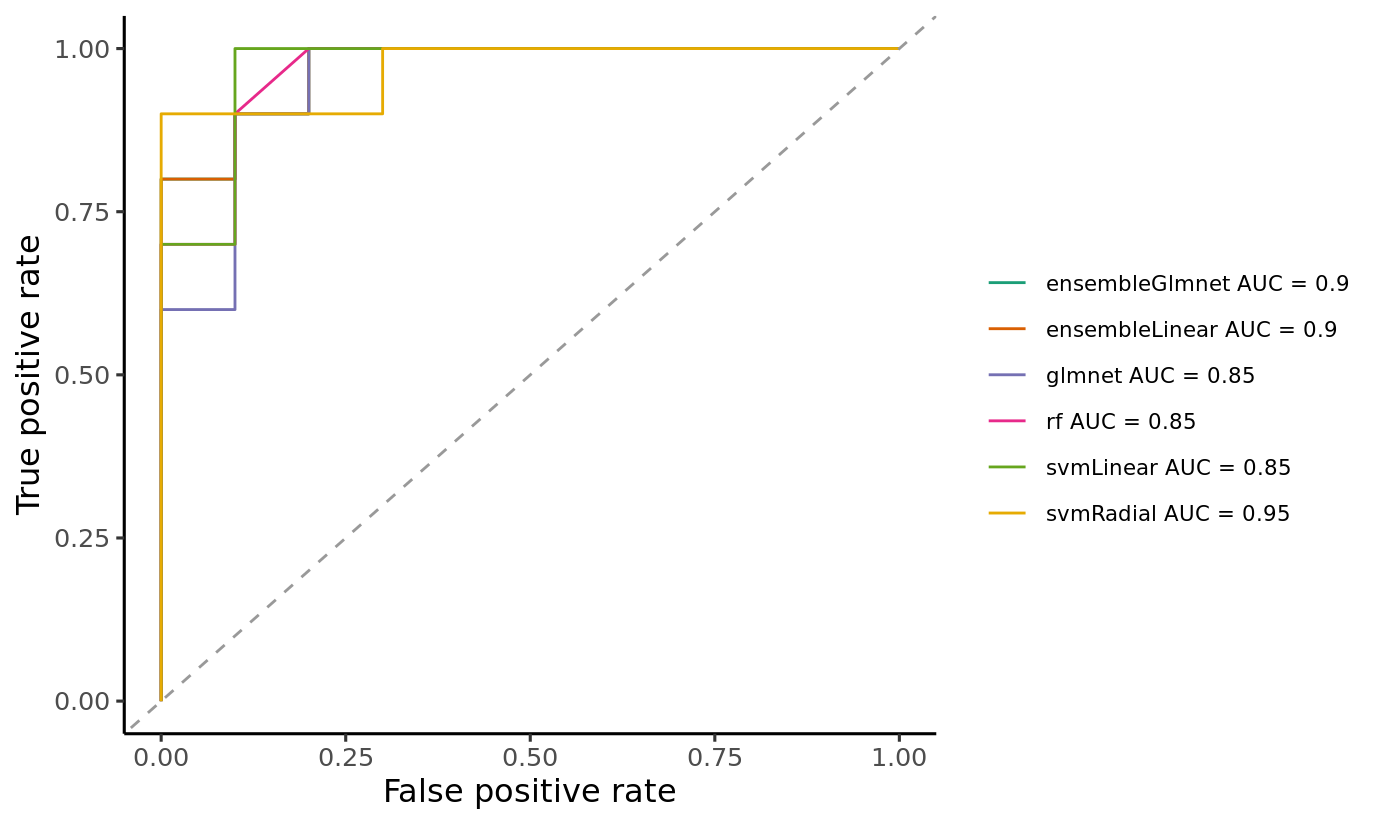

Supplement: Supplementary file 1 [file genes-14-01752-s001.zip › 20-HD-Supple Fig 9- Frontal lobe - Methylation - Predictor.png]
